# Retroviral DNA Integration: Viral and Cellular Determinants of Target-Site Selection

**DOI:** 10.1371/journal.ppat.0020060

**Published:** 2006-06-23

**Authors:** Mary K Lewinski, Masahiro Yamashita, Michael Emerman, Angela Ciuffi, Heather Marshall, Gregory Crawford, Francis Collins, Paul Shinn, Jeremy Leipzig, Sridhar Hannenhalli, Charles C Berry, Joseph R Ecker, Frederic D Bushman

**Affiliations:** 1 Infectious Disease Laboratory, The Salk Institute, La Jolla, California, United States of America; 2 Divisions of Human Biology and Basic Sciences, Fred Hutchinson Cancer Research Center, Seattle, Washington, United States of America; 3 Department of Microbiology, University of Pennsylvania School of Medicine, Philadelphia, Pennsylvania, United States of America; 4 National Human Genome Research Institute, National Institutes of Health, Bethesda, Maryland, United States of America; 5 Genomic Analysis Laboratory, The Salk Institute, La Jolla, California, United States of America; 6 Department of Genetics, University of Pennsylvania School of Medicine, Philadelphia, Pennsylvania, United States of America; 7 Department of Family/Preventive Medicine, University of California San Diego School of Medicine, La Jolla, California, United States of America; King's College London, United Kingdom

## Abstract

Retroviruses differ in their preferences for sites for viral DNA integration in the chromosomes of infected cells. Human immunodeficiency virus (HIV) integrates preferentially within active transcription units, whereas murine leukemia virus (MLV) integrates preferentially near transcription start sites and CpG islands. We investigated the viral determinants of integration-site selection using HIV chimeras with MLV genes substituted for their HIV counterparts. We found that transferring the MLV integrase *(IN)* coding region into HIV (to make HIVmIN) caused the hybrid to integrate with a specificity close to that of MLV. Addition of MLV *gag* (to make HIVmGagmIN) further increased the similarity of target-site selection to that of MLV. A chimeric virus with MLV Gag only (HIVmGag) displayed targeting preferences different from that of both HIV and MLV, further implicating Gag proteins in targeting as well as IN. We also report a genome-wide analysis indicating that MLV, but not HIV, favors integration near DNase I–hypersensitive sites (i.e., +/− 1 kb), and that HIVmIN and HIVmGagmIN also favored integration near these features. These findings reveal that IN is the principal viral determinant of integration specificity; they also reveal a new role for Gag-derived proteins, and strengthen models for integration targeting based on tethering of viral IN proteins to host proteins.

## Synopsis

A required step in the replication cycle of retroviruses is the integration of a DNA copy of the viral genome into a host cell chromosome. Recent studies have shown that human immunodeficiency virus (HIV) and murine leukemia virus (MLV) favor integration near different chromosomal features. HIV preferentially targets active genes, while MLV prefers integration near start sites of gene transcription. The authors investigated integration-target site–selection by HIV derivatives substituted with segments of MLV to determine which viral proteins are responsible for integration-targeting preferences. They found that the viral integrase protein is the dominant determinant of integration-site selection, probably through its tethering to cellular proteins bound near preferred genomic regions. In addition, components of the viral structural polyprotein, Gag, appear to be involved in targeting. These findings provide a functional map of the viral proteins involved in directing integration-site selection.

## Introduction

The selection of target sites for integration of retroviral DNA is central to the biology of retroviruses and the application of retroviral vectors to gene therapy. The recent setbacks in human gene-therapy trials, in which a therapeutic retroviral vector integrated near the *LMO-2* proto-oncogene and caused leukemia-like illness in three patients [1–3], have focused particular attention on the mechanisms responsible for integration targeting. Here we map the retroviral determinants of integration-target site–selection and investigate candidate mechanisms.

**Figure 1:**
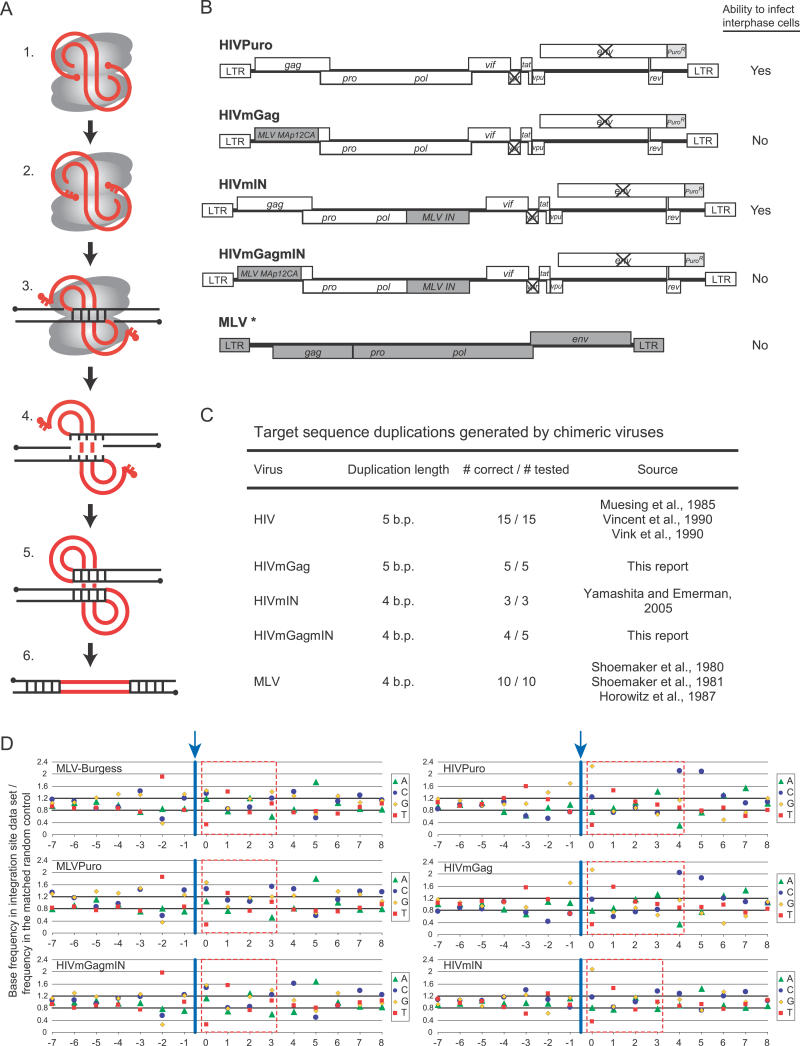
Retroviral DNA Integration and the Chimeric Viruses Used in This Study (A) The DNA breaking and joining reactions mediating integration. Gray ovals represent integrase monomers, thick red lines represent viral DNA, black lines represent target DNA, and dots represent 5′ ends. (1) Linear blunt-ended viral cDNA is bound by integrase as part of the preintegration complex. (2) Integrase removes two nucleotides from the 3′ ends of the viral DNA, exposing recessed 3′ hydroxyl groups. (3) IN joins the recessed 3′ ends of viral DNA to the target DNA. (4) Unpairing of the target DNA between the joined ends of the viral DNA yields gaps in the target DNA. (5) DNA repair enzymes fill in the gaps. (6) The provirus is flanked by repeated segments of the target DNA. (B) Chimeric HIV derivatives containing segments of MLV. At the top is the HIV parent virus, with *vpr* and *env* inactivated and the puromycin resistance gene in place of *nef.* Following that are the chimeras, with substitutions of MLV *gag* gene segments (MA-, p12-, and CA-encoding regions) for HIV MA and CA, or substitution of MLV *IN* for HIV *IN,* or both [20,21]. The MLV genome (indicated by an asterisk) is shown for comparison. The MLV used in this study (MLVPuro) was an MLV-based vector (LPCX) encoding the puromycin resistance gene with Gag, Pol, and Env provided in *trans.* Although we refer to “Gag” in the text, we note that Gag is in fact a polyprotein which is cleaved into individual functional proteins by the action of the viral protease. (C) Target-sequence duplication lengths made by HIV, MLV, and the chimeric viruses. (D) Primary sequences at the site of integration for HIV, MLV, the chimeric viruses, and a previously published MLV dataset (MLV-Burgess). On the *x*-axis are the top strand positions surrounding the point of integration, which is represented by the blue arrow and line (between positions −1 and 0). For each dataset, the proportion of each base at a given location was divided by the proportion of that base in the matched random control set, such that a base with a *y* value >1 is present at an increased frequency, while a base with a *y* value <1 is present at a decreased frequency compared to random sites. A dashed red box surrounds the target sequence that is duplicated upon integration.

The basic DNA cleavage and joining reactions mediating retroviral integration are common among retroviruses (summarized in Figure 1A), but integration in vivo shows pronounced favored and disfavored chromosomal regions that differ among retroviruses. Retroviral integration-site selection is not strongly sequence-specific with respect to the target DNA at the point of joining, though a weakly conserved palindromic sequence can be detected when many integration-target sites are aligned [4–7]. Early studies of murine leukemia virus (MLV) integration targeting led to the suggestion that integration may be favored in open chromatin [8], since a positive correlation was detected between integration frequency and DNase I–hypersensitive sites [9,10]. More recently, the completion of the draft human genome sequence has allowed systematic studies of integration targeting by high-throughput sequencing of integration acceptor sites [11–14]. Human immunodeficiency virus (HIV) integration sites are found predominantly in active transcription units [11,13]. A cellular protein, lens epithelium–derived growth factor (LEDGF/p75), binds HIV IN [15–18] and is partially responsible for favored integration in genes [19]. For MLV, in contrast, roughly 25% of integration events are near transcription start sites and associated CpG islands, while integration within transcription units is only slightly favored [14]. Avian sarcoma-leukosis virus (ASLV) shows the most random pattern of integration-site selection—ASLV favors transcription units only weakly and does not favor transcription start sites [11,12]. Thus for the three retroviruses studied in detail, three different patterns of favored integration sites were found.

Here we investigate the requirements for integration targeting using chimeric viruses in which gene segments of MLV were substituted for the corresponding segments of the HIV genome (Figure 1B). The chimeras contained MLV *gag* gene segments substituted for HIV *gag* (HIVmGag) [20], MLV *IN* substituted for HIV *IN* (HIVmIN) [21], or both MLV *gag* and MLV *IN* substituted for their HIV counterparts (HIVmGagmIN) [21].

Previous characterization has shown that these viruses differ in their ability to infect interphase cells, and that this property maps to the *gag* gene polyprotein precursor [20,21]. MLV integrates only after mitosis, while HIV can integrate at any time during the cell cycle. The chimeric viruses HIVmGag and HIVmGagmIN show the same requirement for cell division as does MLV [20,21], while HIVmIN, like HIV, can infect non-dividing cells [21] (summarized in Figure 1B). Thus MLV Gag imposes the requirement for cell division on HIV. Consequently, tests of integration-target site–selection by these chimeras provide an opportunity to probe the influence of cell-cycle progression on integration-target site–selection.

Integration-site selection by the chimeric and control viruses was assayed by cloning and sequencing 2,440 junctions between human and proviral DNA generated by infection of human cells. We found that HIVmIN and HIVmGagmIN favored integration near transcription start sites and CpG islands, paralleling the preferences of MLV and implicating IN as the main specificity determinant. The resemblance was closest between MLV and HIVmGagmIN, implicating Gag as a cofactor for targeting as well as IN. HIVmGag exhibited a phenotype that did not resemble either parent—it did not favor transcription starts and CpG islands like MLV, and it did not favor integration in transcription units or gene-rich regions as strongly as did HIV, further implicating Gag as well as IN. In addition, we used new genome-wide data on preferential DNase I cleavage sites [22] to analyze the relationship with favored integration sites, and found that MLV favored integration within 1 kb of DNase I cleavage sites, as did the HIVmIN and HIVmGagmIN chimeras. However, HIV, ASLV, and L1 retrotransposons did not favor these sites, indicating that possible open chromatin marked by DNase I cleavage sites was not globally favorable for integration of new DNA sequences. This result is more consistent with models based on specific interactions between MLV IN and cellular proteins bound near DNase I cleavage sites. These data elucidate the viral determinants of integration targeting, disclose a role for Gag in integration, and indicate that models for targeting based solely on open chromatin or cell-cycle effects are unlikely to be correct.

## Results

### Cloning and Analysis of Integration Sites

The chimeric viruses used in this study were deleted for the *env* gene and complemented with the vesicular stomatitis virus G protein (VSV-G) to boost titer and restrict infection to a single round. These chimeras were less infectious than the wild-type virus [20,21], so the puromycin resistance gene was cloned in place of *nef,* allowing infected cells to be selected with puromycin to enrich for provirus-containing cells. *Vpr* was also deleted because of its cellular toxicity [23]. In order to control for possible biases in integration-site recovery due to puromycin selection, control infections were carried out with an HIV derivative transducing the puromycin resistance gene (termed “HIVPuro”) and an MLV vector (LPCX) also transducing the puromycin resistance gene (termed “MLVPuro”). Attempts to make reciprocal constructs (HIV gene segments into an MLV background) did not yield infectious viruses (unpublished data). HeLa cells were chosen as infection target cells because they are highly susceptible to infection and because they had been used in a previous study comparing MLV and HIV integration targeting [14].

To clone integration sites, genomic DNA from infected cells was extracted, digested with *Mse*I and ligated to adapters. The junctions between proviral DNA and genomic DNA were amplified by nested PCR using primers complementary to proviral and adaptor sequences, cloned, sequenced, and mapped to the human genome as described [11,13,14,24]. Newly determined sets of integration sites (a total of 2,440 sites for the five viruses studied) were compared to each other and to previously reported datasets (Table 1). The distribution of integration sites was also compared to random sites in the human genome generated computationally. As is discussed in Protocol S1, a bioinformatic procedure was used to control for potential biases in integration-site recovery due to possible nonrandom distributions of restriction sites in the human genome.

**Table 1:**
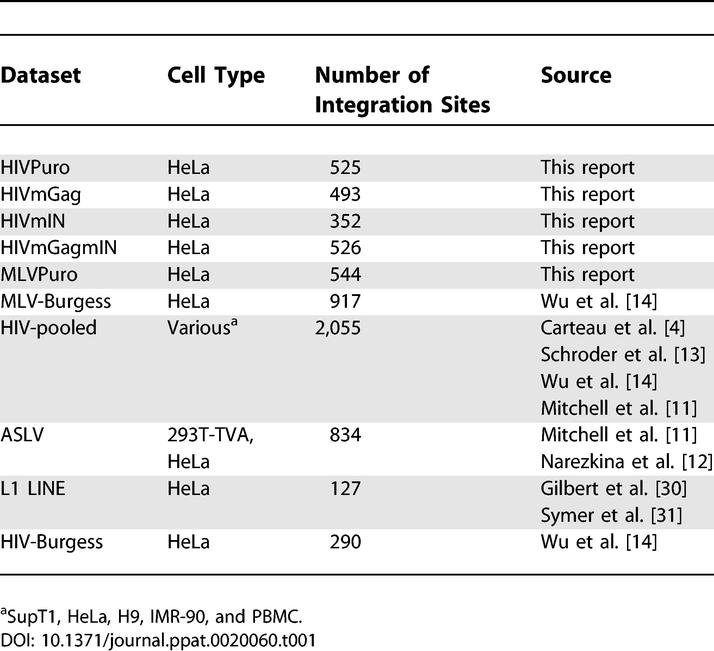
Integration-Site Datasets Used in This Study

As a test for correct integration by the chimeric viruses, we determined the target-site duplication lengths for a few integration events of each chimeric virus (Figure 1C). Each chimeric virus showed mostly the duplication length that is characteristic of the virus donating the *IN* segment—4 bp for MLV and 5 bp for HIV—which is as expected because IN is known to dictate the length of the duplication [25,26]. For unknown reasons, one out of five duplications for the HIVmGagmIN chimera was 5 bp instead of the expected 4 bp; all the others were as expected. In addition, all integration events showed evidence of correct cleavage at the viral DNA 3′ end by integrase. These data support the idea that the IN–DNA complexes of the chimeras generally assembled and functioned normally.

The target DNA sequences at the point of integration were then compared (Figure 1D). Previous studies showed that retroviruses have weak preferences for specific primary DNA sequences at integration sites and, when large numbers of sites are analyzed, these biases become statistically very significant [4–7]. We found that two MLV datasets and the HIVmGagmIN dataset showed the previously determined MLV-favored site, and that the HIVPuro and HIVmGag datasets matched the known HIV sequence. Unexpectedly, the HIVmIN site showed lower information content than the others and was somewhat intermediate in sequence. Pairwise comparisons of selected positions in the consensus sequence showed significant differences (e.g., *p* < 0.0001 for comparison of HIVmIN to HIVPuro at position −3; *p* < 0.0001 for comparison of HIVmIN to MLVPuro at position −2, analyzed by chi-square). This indicates that Gag determinants, as well as IN determinants, can influence the favored primary sequences at integration sites.

### Integration Frequency near Transcription Start Sites and CpG Islands

**Figure 2:**
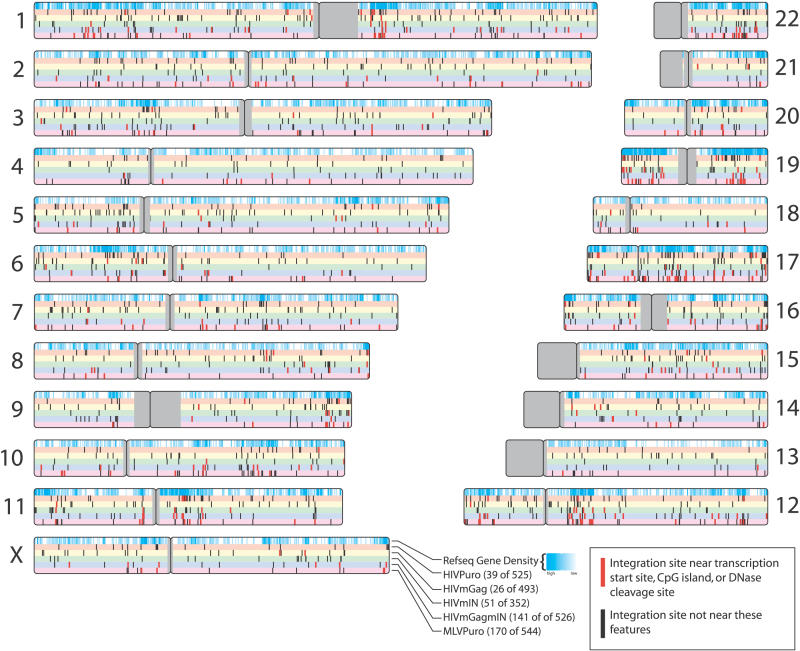
Positions of Retroviral Integration Sites on the Human Chromosomes The human chromosomes are shown numbered. Centromeric regions (which are mostly unsequenced) are shown in gray. Relative gene density is indicated in the top bar on each chromosome by the intensity of the cyan coloration. Integration-site datasets (lower bars) are color-coded as indicated. Sites of integration near transcription start sites (within ± 5 kb), CpG islands (within ± 1 kb of a CpG midpoint), or 2 DNase I cleavage sites are shown as red dashes; other sites are black.

**Figure 3:**
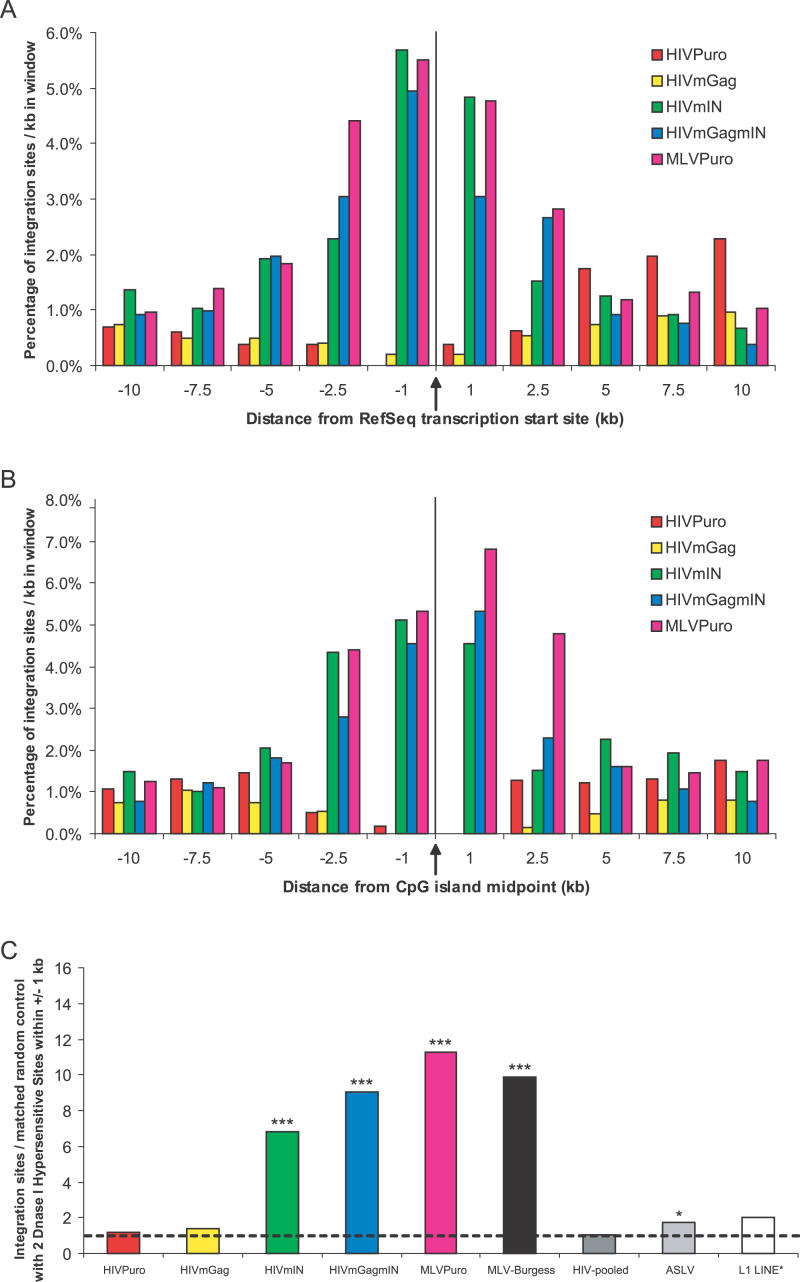
Frequency of Integration near Transcription Start Sites, CpG Islands, and DNase I Cleavage Sites, Illustrating the Contribution of MLV IN to Specificity (A and B) The percentage of integration sites (per kb) within each interval is shown for (A) transcription start sites, and (B) CpG islands. (C) DNase I cleavage sites, For each dataset, the proportion of integration sites found within ± 1 kb of two DNase I cleavage sites was divided by the proportion in the matched random control set. The dotted line represents the expected bar height if the observed data did not differ from the random control set. L1 is displayed as the ratio over an unmatched random set. Three asterisks denote *p* < 0.0001 by chi-square comparison to random sites. Single asterisk denotes *p* = 0.0396.

Integration sites for each of the five viruses were mapped to the human genome, and nearby features were assessed (Figure 2). To begin to compare integration by the chimeras, we evaluated the frequency of integration near transcription start sites and CpG islands (Figure 3A and 3B; Table 2). The MLVPuro control exhibited a strong preference for integration near transcription start sites—26.1% of MLVPuro sites were within ± 5 kb of a RefSeq gene-transcription start site compared to 5.0% of matched random control sites. For the HIVPuro virus, only 6.9% were near transcription start sites. Thus the preferential integration near transcription start sites by MLV, but not HIV, reported previously [13,14] was seen despite the puromycin selection of transduced cells (*p* < 0.0001 for pairwise comparison of HIVPuro and MLVPuro; chi-square test).

**Table 2:**
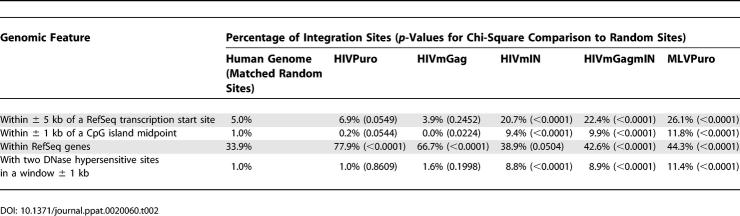
Integration near Genomic Features

The HIVmIN and HIVmGagmIN target-site preferences closely paralleled MLV, showing 20.7% and 22.4% of integration events within ± 5 kb of transcription start sites, respectively. These high frequencies were significantly different from HIVPuro (*p* < 0.0001 for both comparisons; chi-square test), and the random control (Table 2), but not significantly different from MLVPuro (*p* > 0.05 for both comparisons; chi-square test). HIVmGag differed, showing only 3.9% of integration events near transcription start sites, which was significantly lower than HIVPuro (*p* = 0.0342; chi-square test). Thus MLV *IN* is a sufficient determinant for directing favored integration near transcription start sites, and Gag-derived proteins also influence integration near these features.

The integration frequency near CpG islands was then compared. CpG islands are regions rich in the CpG dinucleotide, which are undermethylated and frequently associated with gene regulatory regions [27]. MLV strongly favors integration near CpG islands while HIV does not [11,14]. We quantified integration frequency near CpG islands and found that the MLVPuro, HIVmIN, and HIVmGagmIN viruses all favored integration near these sites. Specifically, 11.8%, 9.4%, and 9.9% of sites, respectively, were within 1 kb of a CpG island midpoint, compared to 1.0% of matched random sites. HIVPuro was not significantly different from random sites (0.2%), while the HIVmGag virus significantly disfavored regions within 1 kb of a CpG island midpoint (0%, *p* = 0.0224 for chi-square comparison to random sites). The MLVPuro, HIVmIN, and HIVmGagmIN datasets showed significantly more frequent integration near CpG islands than did the HIVPuro and HIVmGag datasets (*p* < 0.0001 for any pairwise comparion between the two groups; chi-square test).

In summary, the HIVmIN and HIVmGagmIN chimeras resembled MLV in their strong preferences for integration near transcription start sites and CpG islands. Evidently, MLV IN is sufficient to direct favored integration near these features. HIVPuro showed significant differences from HIVmGag, implicating Gag in integration targeting near these features as well.

Another difference between HIV and MLV is the different frequency of integration within transcription units (Table 2). The HIVPuro virus favored integration in these sequences (77.9% in RefSeq genes), while the MLVPuro virus showed a much weaker trend (44.3% in RefSeq genes), which is only slightly above the frequency for random sites (33.9%). Comparing the frequency between HIVPuro and MLVPuro achieved *p* < 0.0001 (chi-square test). The HIVmIN and HIVmGagmIN viruses did not differ significantly from the MLVPuro virus (*p*-values are 0.1112 and 0.5713, respectively; chi-square test). Both HIVmIN and HIVmGagmIN differed significantly from HIVPuro (*p* < 0.0001 for both comparisons; chi-square test). HIVmGag showed an intermediate phenotype, being down significantly in the frequency of targeting transcription units compared to HIVPuro (reduced 11%; *p* < 0.0001; chi-square test), but still significantly greater than the MLVPuro, HIVmIN, or HIVmGagmIN viruses (*p* < 0.0001 for all comparisons; chi-square test). Thus, analysis of integration frequency in transcription units also indicated that *IN* was the key determinant, but *gag* also contributed.

We next assessed the effects of transcriptional activity on integration frequency using transcriptional profiling data for the HeLa target cells. All viruses tested favored active transcription units for integration compared to randomly selected genes (*p* < 0.0001, Mann-Whitney U-test on signal values). The median expression level of genes targeted for integration was highest for the HIVPuro and HIVmGag viruses but lower for HIVmIN, HIVmGagmIN, and MLVPuro viruses—in that order. The median signals were significantly different between the HIVPuro virus and the HIVmGagmIN and MLVPuro viruses (*p* = 0.0005 and *p* = 0.0241; Mann-Whitney U-test of signal values for genes targeted by HIVPuro versus HIVmGagmIN and by HIVPuro versus MLVPuro, respectively). Thus the chimeras containing MLV *IN* in the HIV background paralleled MLV by this measure as well.

### Integration Frequency near DNase I Cleavage Sites

Early studies of MLV integration targeting suggested that MLV favors DNase I–hypersensitive sites for integration [8–10]. DNase I–hypersensitive sites are believed to be nucleosome-depleted chromosomal regions associated with regulatory elements [28]. Genome-wide mapping of DNase I cleavage sites in chromatin has revealed that they are enriched near the 5′ ends of transcription units, near CpG islands, and near active genes, reinforcing the idea that they are markers for regulatory regions [22,29].

To assess the correlation between retroviral integration and DNase I cleavage frequency genome-wide, we quantified integration sites within 1 kb of two positions of DNase I cleavage mapped by Crawford et al. [22]. We chose to use two cleavage sites in the analysis instead of a single site to better match the experimental definition of DNase I–hypersensitive sites, which relies on multiple cleavage events. The conclusions were similar whether one, two, or three DNase I cleavage sites were used for analysis (unpublished data). For technical reasons, Crawford et al. analyzed cleavage sites in resting T cells, but further analysis showed that 80% of sites were shared between resting T cells and HeLa cells [22]; we therefore extrapolated their data for comparison to integration sites in HeLa cells studied here.

Table 2 shows the percentage of integration sites that were in intervals (plus or minus 1 kb of the integration sites) containing two or more DNase I cleavage sites. The enrichment relative to the matched random control is shown in Figure 3C. We also analyzed previously published datasets from MLV [14], HIV [4,11,13,14], ASLV [11,12], and the L1 retrotransposon [30,31] and plotted these in Figure 3C for comparison.

Of these, MLV showed by far the strongest preference for integration near DNase I cleavage sites. HIV and L1 retrotransposons showed no preference for integration near DNase I cleavage sites, while ASLV showed a weak preference that barely achieved statistical significance. Thus, contrary to the expectation that open chromatin at DNase I cleavage sites is globally favorable for integration, we find that strong favoring of integration near DNase I cleavage sites is specific to MLV.

DNase I cleavage sites are known to be enriched near promoters, raising the question of whether the association of DNase I cleavage sites and MLV integration sites is just a reflection of favored integration near promoters. However, a bioinformatic analysis of this issue (Protocol S2; unpublished data) indicates that proximity to DNase I cleavage sites is favorable for integration independently of proximity to promoters. For example, when promoter locations are approximated as the 1 kb of DNA just upstream from a RefSeq transcription start site, analysis of integration sites outside these regions still reveals increased frequency of MLV integration plus or minus 500 bp from a DNase I cleavage site (*p* < 0.00001).

HIVmIN and HIVmGagmIN were similar to the MLVPuro virus in that they strongly favored DNase I–hypersensitive sites for integration, and all three differed significantly from HIVPuro or HIVmGag (*p* < 0.0001 for any pairwise comparison between the two groups; chi-square test). Like the HIVPuro virus, the HIVmGag virus did not favor these sites for integration above the expectation for random placement (Table 2). Thus substituting MLV *IN* into HIV was sufficient to transfer the tendency to favor integration near DNase I cleavage sites.

### Transcription-Factor Binding Sites near Integration Sites

**Figure 4:**
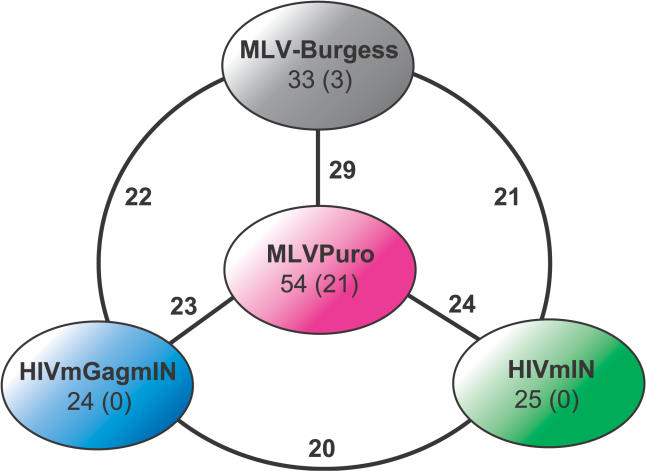
Diagram of the Relationship of Transcription-Factor Binding Sites Enriched in the MLVPuro, MLV-Burgess, HIVmIN, and HIVmGagmIN Integration-Site Datasets The genomic sequences within 1 kb of each integration site were used for analysis. Ten matched random-control integration sites were compared to each experimental integration site. The cut-off value for over-representation was 2.0-fold. All comparisons achieved *p* ≤ 0.001. The number of enriched transcription-factor binding sites in each dataset is shown with the number of factors unique to each in parentheses. The edge labels show the number of commonly enriched sites between pairs of datasets.

Given the favoring of integration by MLV, HIVmIN, and HIVmGagmIN near promoters, we investigated whether transcription-factor binding sites were enriched near integration sites of these viruses. We evaluated possible enrichment of 546 transcription-factor binding-site motifs within plus or minus 1 kb of integration sites compared to matched random control sites. To assess the generality of any findings, we also included in this study a previously published set of MLV integration sites in HeLa cells (termed MLV-Burgess; [14]). The MLVPuro, MLV-Burgess, HIVmIN, and HIVmGagmIN datasets showed by far the highest numbers of significantly (*p* < 0.001) enriched transcription-factor binding-site motifs (54, 33, 25, and 24, respectively). The HIVPuro and HIVmGag returned far fewer (1 and 0). Strikingly, for the MLV group of motifs, many were common to all four datasets, or were shared between multiple group members (Figure 4). Seventeen significantly enriched factors were common to all four, thus specifying a set of cellular factors correlated with MLV (plus HIVmIN and HIVmGagmIN) integration (see Table S1). No single motif was common between the HIVPuro and HIVmGag datasets.

However, many of the sites in Figure 4 were not found to be enriched when promoter sequences were used as controls instead of randomly chosen genomic sites (see Table S1), indicating the general features of promoters correlate most strongly with MLV integration. Nevertheless, a few transcription-factor binding sites were still significantly enriched when promoters were used as controls (requiring 1.5-fold enrichment and *p* ≤ 0.001), suggesting potential specific interactions. Among the datasets in Figure 4, binding sites for the Ap-1 and Bach1 transcription factors were enriched relative to promoter controls in three out of four datasets (HIVmIN was the exceptional dataset). In addition, a regression analysis indicated that the presence of a nearby promoter could not fully account for the favorable effect of transcription-factor binding sites on integration frequency, again indicating a possible effect of the transcription-factor binding sites beyond just marking promoters (Protocol S2).

### Global Comparison of Trends in Integration Targeting

**Figure 5:**
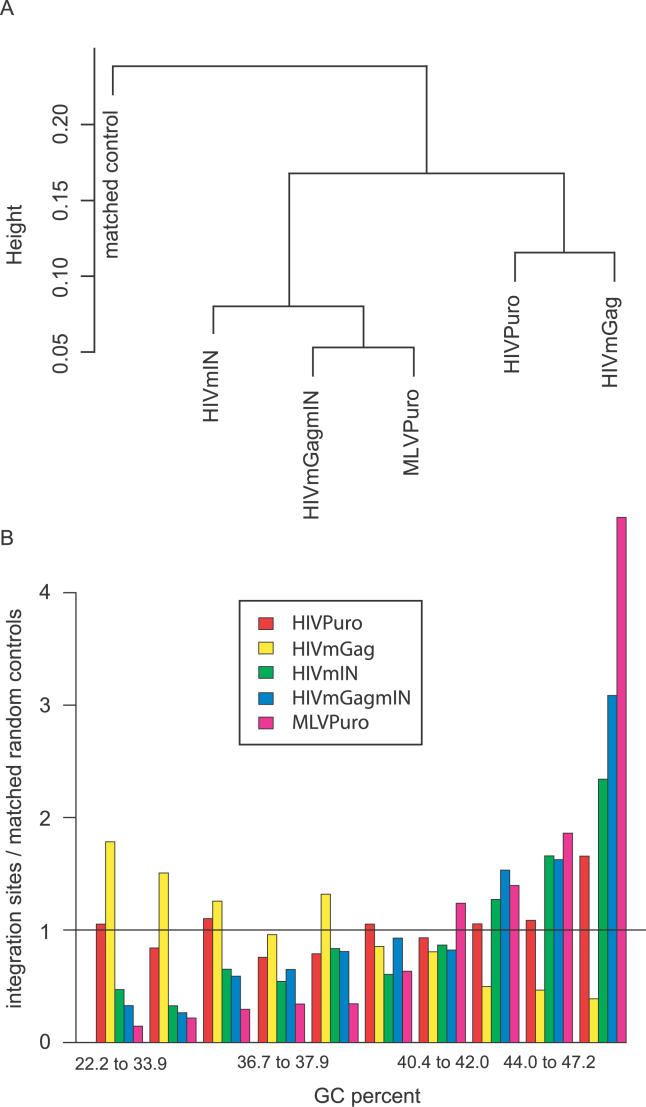
Effects of Gag Proteins on Integration Targeting (A) Clustering by the machine learning algorithm RandomForest, illustrating an influence of Gag determinants as well as IN. For a detailed description of the method, see Protocol S3. (B) An analysis of the G/C percentage at integration sites.

To assess the similarities among integration-site datasets, a machine learning algorithm based on RandomForest was developed to cluster the datasets, taking into account 109 different types of genomic features (Figure 5A; Protocol S3). Examples of genomic features included: gene calls, CpG islands, G/C content, DNase I cleavage sites, and gene boundaries (a detailed list is included in Protocol S3). The MLVPuro, HIVmIN, and HIVmGagmIN integration-site datasets were clustered together by this means. HIVmGagmIN resembled MLV the most closely. HIVPuro and HIVmGag clustered together, though the analysis also emphasized the distinctions between the two datasets. The genomic features most responsible for distinguishing among integration-site datasets could be determined by further analysis of the clustering results (summarized in Protocol S3). Measures of proximity to transcription start sites and gene boundaries were prominent, as were measures of integration in genes and gene density, all as expected from the data in Table 2 and Figure 3.

Another significant feature was the G/C content at integration sites. The effects of G/C content in isolation are presented in Figure 5B. For the highest G/C content category, there are obvious, strong effects (*p* < 0.0005 for each integration complex). In the human genome, regions of high G/C content are also high in transcription units, SINE elements, CpG islands, and a variety of other features. Controlling for these features would be expected to reduce the strength of the relationship shown in Figure 5B. However, after controlling for the presence of a CpG island within ± 2.5 kb, the effects of being in the highest G/C content category are still significant (at *p* < 0.005 for each virus studied). HIVPuro and HIVmGag differed by this measure (particularly in the highest G/C content category where *p* < 1e^−10^), indicating that Gag proteins play a role in integration targeting near these sequences.

### Effects of Selection on Populations of Proviruses

**Figure 6:**
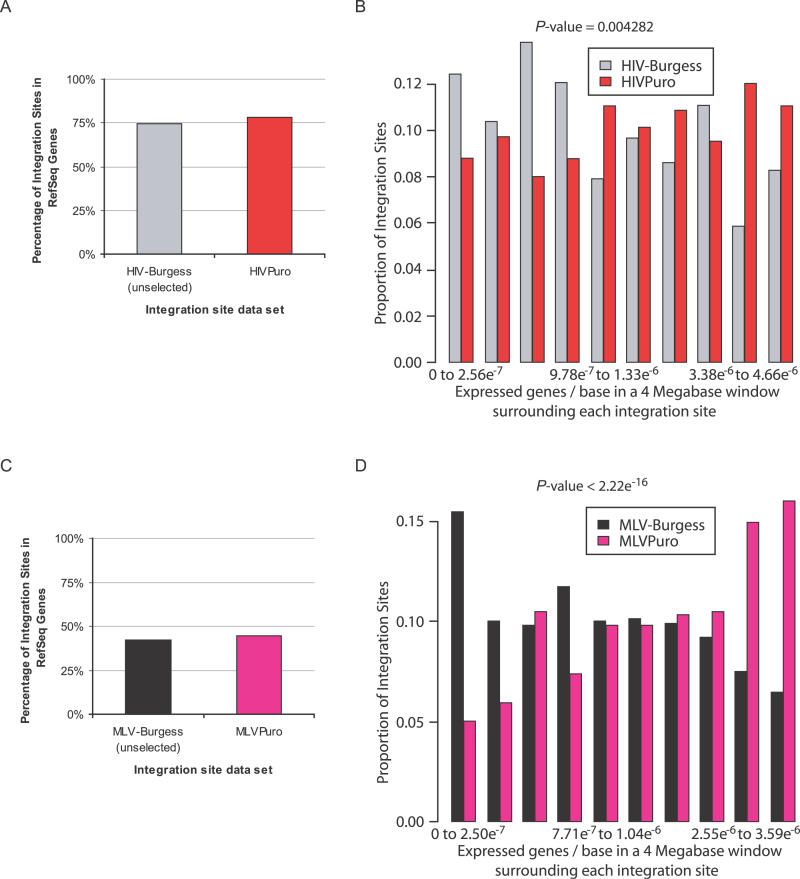
Effects of Selection for Provirus Gene Expression on the Distribution of Integration Sites (A) Frequencies of integration in RefSeq genes in the HIV-Burgess and HIVPuro datasets. (B) Comparison of the relative frequency of integration in the HIV-Burgess and HIVPuro datasets as a function of transcriptional intensity. The proportion of integration sites from each dataset in regions of varying transcriptional intensity was plotted increasing from left to right along the *x*-axis (in groups divided according to deciles of density). For “expressed genes” in this plot, we counted the number of genes whose expression level in HeLa cells was in the upper 1/8th of genes assayed on the HG-U133A microarray. The *p*-value given is the result of fitting a cubic polynomial to the expressed gene-density values. (C) Frequencies of integration in RefSeq genes in the MLV-Burgess and MLVPuro datasets. (D) Comparison of the relative frequency of integration in the MLV-Burgess and MLVPuro datasets as a function of transcriptional intensity. See Protocols S4 and S5 for details and additional plots.

In order to clone a large number of integration sites from the poorly infectious chimeric viruses, it was necessary to select infected cells with puromycin, raising the question of to what extent the selection for proviral gene expression affected the ultimate distribution of integration sites. Previous work showed that selecting for proviral expression can influence the population of integration sites recovered, though the effect was modest [24]. To account for this, puromycin-transducing HIV (HIVPuro) and MLV (MLVPuro) control viruses were used in the present study for comparison to the chimeras. Thus the data from this study, combined with previous work, allows the effects of selection to be analyzed by comparing the HIVPuro and MLVPuro datasets to unselected HIV and MLV datasets (Wu et al. [14]; Table 1), which were also generated by infection of HeLa cells. The pairs of datasets were compared in a semiautomated fashion with respect to many types of genomic annotation. The results are presented in detail in Protocols S4 and S5, and highlights are shown in Figure 6 and Table S2.

The unselected HIV-Burgess dataset did not differ significantly from the HIVPuro dataset over many forms of annotation. For example, the two did not differ in the frequency of integration in RefSeq genes (Figure 6A), the proportion of sites within 1 kb of a CpG island, or the proportion of sites within 1 kb of two DNase I cleavage sites (Table S2). However, the two datasets did differ with respect to the gene density of regions hosting integration sites (*p* = 0.0085 for gene density in a 4-Mb window surrounding each integration site; Protocol S4), as did the response to transcriptional intensity in the surrounding region (Figure 6B). These data suggest that gene-dense regions are more favorable for HIV provirus expression, reinforcing earlier findings that integration within long intergenic regions disfavored subsequent proviral gene expression [24].

The MLVPuro dataset did not differ from the unselected MLV-Burgess dataset in the proportion of integration sites within RefSeq genes (Figure 6C), within 5 kb of a RefSeq gene-transcription start site, within 1 kb of a CpG midpoint, or within 1 kb of two DNase I cleavage sites (Table S2). However, like HIV, the selected and unselected MLV sites did differ in their frequency in gene-dense regions (*p* < 2.22e^−16^ for gene density in a 4-Mb window surrounding each integration site; Protocol S5) and their response to local transcriptional intensity (Figure 6D). Selected and unselected MLV also differed in the G/C content at integration sites (*p* = 4.52e^−11^; see Protocol S5). Evidently, gene-dense regions and correlated regions of high G/C content are favorable for MLV gene expression after integration.

## Discussion

Previous studies of target-site selection by mobile DNA elements have revealed that the determinants of integration targeting can be diverse. The prokaryotic transposons Tn7 and bacteriophage Mu each encode specific proteins, distinct from the element-encoded transposase enzymes that bind to integration-target DNA and direct site selection (reviewed in [32]). For the Saccharomyces cerevisiae Ty retrotransposons, in contrast, there is strong evidence for a tethering mechanism involving direct binding of the Ty integrase enzyme to a cellular protein bound near favored sites on target DNA [33–37]. Here we report that two virus-encoded determinants are involved for retroviruses: the IN protein and components of the Gag polyprotein.

An alternative explanation for the data presented here could have been that the viral nucleic acid sequence, and not the encoded protein, was the determinant of target-site specificity. As shown in Figure 1B, the viral DNAs that become integrated retain the *IN* and *gag* coding regions. Thus it appears possible that a binding site for a cellular protein might exist in the DNA encoding *IN* or *gag,* and that binding of a cellular factor to this DNA site could mediate integration-site selection. However, this model can be ruled out, because integration-site sequence data has been obtained for both HIV and MLV using retroviral vectors that lack the *gag* and *IN* coding regions, and these show the same target-sequence preferences as the viruses that do contain the *IN* and *gag* coding regions studied here (MLVPuro is such a dataset for MLV, and [11,13] report examples for HIV). Thus the IN and Gag-derived proteins are responsible for selecting the integration target, and not a DNA site within the region encoding *gag* or *IN.*

The earliest model for the mechanism of integration-site selection by retroviruses proposed that open chromatin was favored because MLV favored integration near DNase I–hypersensitive sites [8–10]. However, our genome-wide data indicate that DNase I–sensitive regions are not universally favorable. Only MLV—and not HIV, ASLV, or L1—strongly favored integration near to these sites. It is unclear whether relatively greater exposure of DNA at DNase I–hypersensitive sites is involved in integration targeting at all. Binding of MLV integration complexes to specific cellular proteins bound at or near DNase I–hypersensitive sites may fully explain the observations. Contrary to the initial interpretation of the data on integration and DNase I–hypersensitive sites, we conclude that none of the available data require explanations based on DNA accessibility to explain integration targeting near these sites.

Another model for the mechanism of integration targeting invokes effects of the cell cycle. HIV and MLV differ in the cell-cycle dependence of infection. HIV can infect cells regardless of cell-cycle phase [38,39], while MLV infection requires host cells to pass through mitosis [40,41]. The transcriptional state of a cell is known to vary with the cell cycle, so the organization of chromosomal DNA encountered by the MLV and HIV integration complexes should differ. The HIVmGag chimera exhibited cell cycle–restricted infectivity, like that of MLV [20]—thus HIVmGag would likely encounter the chromosomal DNA in the same state as does MLV. The targeting preferences of the HIVmGag chimera did differ from those of HIVPuro, potentially supporting the cell-cycle model, but the HIVmGag integration pattern was very different from that of MLV. Thus cell-cycle effects may have a modest influence on integration, but other factors appear to dominate. Consistent with this, studies of HIV integration targeting in non-dividing cells have not shown large differences from studies of integration in dividing cells [42–43].

The best-supported model at present invokes direct tethering interactions between retroviral proteins and cellular factors. Evidence suggests that HIV IN is one determinant of integration targeting, since it binds LEDGF/p75 protein [15–18], and cells lacking LEDGF/p75 show reduced frequency of integration in transcription units [19]. However, the IN–LEDGF/p75 interaction is not a complete explanation for the HIV integration-target preference, because HIV integration in cells depleted for LEDGF/p75 shows only a modest reduction in integration in transcription units, indicating that other factors may be involved [19].

Data reported here implicate IN as the primary determinant of integration targeting, with Gag-derived proteins playing an auxiliary role. For the MLV case, *IN* is clearly a dominant determinant, because it reprograms HIV integration toward the MLV-like pattern. It is possible that determinants for targeting HIV exist in other HIV genes, but are recessive to MLV *IN*. However, the data with LEDGF suggest that HIV IN is one determinant of HIV target-site selection. The mechanism of MLV targeting is not fully specified by our data, but a direct tethering interaction between MLV IN and transcription factors (Figure 4; Table S1) or other proteins bound at promoters is consistent with our findings. The role of Gag is less clear. It could be that MLV Gag–derived proteins are involved indirectly by acting as cofactors for correct assembly of complexes containing MLV IN. Consistent with this idea is the finding that the target-sequence preference at the point of integration is perturbed in the HIVmIN dataset, but fully matches MLV in HIVmGagmIN (Figure 1D). That is, lack of the matched MLV Gag may cause incorrect assembly of MLV IN, resulting in incorrect recognition of the target DNA. MLV Gag could also interact directly with cellular proteins. A third possibility is that MLV Gag is acting through its ability to regulate the relationship of integration to the cell cycle [20], as is discussed above. Our results also suggest that HIV Gag–derived proteins are involved in integration targeting, because the HIVmGag chimera differed significantly from HIVPuro in target-sequence preferences (see, for example, Figure 5B).

In summary, we found that substitution of MLV *IN* for HIV *IN* reprogrammed HIV integration-site selection towards that of MLV. Furthermore, addition of MLV *gag* resulted in a closer parallel with MLV integration targeting. In addition, we found that favored integration near DNase I–hypersensitive sites was an MLV-specific trend, and this tendency also could be transferred to HIV by substituting MLV *IN* into HIV. These data clarify the viral determinants of integration-site selection, reveal a new role for Gag proteins, and constrain models for the mechanisms directing integration targeting by retroviruses.

## Materials and Methods

### DNA constructions.

To generate the MLVPuro dataset, we used LPCX (Clontech, Palo Alto, California, United States), which is an MLV-based vector that expresses the puromycin resistance gene from the MLV LTR. All other vectors used were based on the full-length HIV clone pLAI [44]. *Vpr* was mutated by the insertion of four bases at the NcoI site at 5,207 bp, and *env* has a deletion between the BglII sites at 6,634 and 7,214 bp [23]. The puromycin resistance gene was cloned in place of *nef.* The MLV *gag* gene segment encoding MA, p12, and CA from pAMS [45] was cloned in place of HIV MA and CA for MHIV-mMA12CA-ΔenvΔvprΔnef-puromycin (for the HIVmGag dataset) and MHIV-mMA12CA-mIN-ΔenvΔvprΔnef-puromycin (for HIVmGagmIN) as described previously [20]. For MHIV-mIN-ΔenvΔvprΔnef-puromycin (HIVmIN) and MHIV-mMA12CA-mIN-ΔenvΔvprΔnef-puromycin (HIVmGagmIN), the MLV *IN*–encoding portion of the pAMS *pol* gene was cloned in place of HIV *IN,* starting at the same position of the 5′ end of the HIV *IN* gene segment. The 3′ end of the HIV *IN*–encoding region with the cPPT remains and is separated from the end of MLV *IN* by two stop codons [21]. (The junction sequence is CGTGGAAGCCCTTAATAGTCTgaattc.)

### Infections.

VSV-G–pseudotyped virus was prepared as described previously [20]. HeLa cells were infected by spinoculation [46] with concentrated viral supernatant and 20 μg/ml DEAE-dextran. Infected cells were selected with 0.7 μg/ml puromycin for 2 wk. Genomic DNA was extracted from pooled colonies.

### Cloning integration sites.

Genomic DNA was digested with MseI and ligated to a linker as described previously [14]. The ligase was heat-inactivated at 65 °C for 15 min, and the genomic DNA was digested with a second restriction enzyme to limit the amplification of an internal viral fragment. SpeI was used for the MLVPuro virus, and SacI was used for the HIV-based viruses. Viral-host DNA junctions were amplified by nested PCR using primers specific for the proviral LTR (reading out from the 3′ end) and the linker essentially as described in the GeneWalker Kit manual (Clontech). Nested-PCR products were cloned using the TOPO TA cloning system (Invitrogen, Carlsbad, California, United States). Clones were sequenced and mapped to the human genome with BLAT (University of California, Santa Cruz, California, United States). The viral genotypes in each genomic DNA sample were confirmed by PCR using primers that detected sequences from HIV *gag,* HIV *IN,* MLV *gag,* and MLV *IN.*

For analysis of the length of target-site duplications, integration-site clones were randomly chosen and genomic sequence-specific primers were designed. The viral-host DNA junction from the 5′ LTR of the provirus was amplified from undigested genomic DNA and cloned using the TOPO TA cloning system (Invitrogen). Oligonucleotides used in this study are listed in Table S3.

A question arises regarding the use of the VSV-G envelope for infection instead of the authentic HIV or MLV envelopes, but a direct study of this issue has failed to reveal any differences [43].

### Bioinformatic analysis.

A detailed statistical analysis is presented in Protocols S1–S5. In order to control for possible biases in the datasets due to the choice of restriction endonuclease used in cloning integration sites, each experimental integration site was paired with ten randomly selected sites in the genome that were exactly the same distance from an MseI site. These matched random control sites were generated in silico and were used for comparison to the integration-site datasets as previously described [11].

The statistical analysis of favored binding-site motifs (Figure 4 and Table S1) was carried out as follows. Transcription-factor binding-site motifs, described as positional-weight matrices, were obtained from the TRANSFAC database. Let X and Y denote sets of significant motifs around the integration sites in two independent experiments, with *c* motifs in common. Assuming a random sampling of *|X|* and *|Y|* distinct factors from a pool of 546 transcription-factor motifs, the hypergeometric *p*-value estimates the probability of sampling *c* or more common motifs.

For the analysis of the effects of host-cell transcription on integration, we acquired a set of HeLa transcriptional profiling data (assayed with Affymetrix HG-U133A microarrays [Santa Clara, California, United States]) from NCBI Gene Expression Omnibus (http://www.ncbi.nlm.nih.gov/projects/geo/index.cgi).

## Supporting Information

Protocol S1 Association of Genomic Features with Integration (650 KB PDF)

Protocol S2 Screening Transcription Factors for Effects on Retroviral Integration (309 KB PDF)

Protocol S3 Similarity of Integration Sites of Different Integration Complexes (451 KB PDF)

Protocol S4 Association of Genomic Features with Integration: Unselected versus Puromycin-Selected HIV (524 KB PDF)

Protocol S5 Association of Genomic Features with Integration: Unselected versus Puromycin-Selected MLV (521 KB PDF)

Table S1 Transcription-Factor Binding-Site Motifs Enriched in Each Integration-Site Dataset (41 KB XLS)

Table S2 Comparison of Selected and Unselected Datasets (14 KB XLS)

Table S3 Oligonucleotides Used in This Study (16 KB XLS)

### Accession Numbers

The NCBI Gene Expression Omnibus (http://www.ncbi.nlm.nih.gov/projects/geo/index.cgi) accession numbers for the publicly available data that we used in our analysis of host-cell transcription effects on integration are GSM23372, GSM23373, GSM23377, and GSM23378*.*

The NCBI GenBank (http://www.ncbi.nlm.nih.gov/Genbank/index.html) accession numbers for integration sites sequenced in this study are: HIVPuro (DX597229–DX598304), HIVmGag (DX588312, DX593208–DX594687), HIVmIN (DX594688–DX597228), HIVmGagmIN (DX590011–DX590615), and MLVPuro (DX598305–DX598906).

## Abbreviations

ASLV: avian sarcoma-leukosis virus
HIV: human immunodeficiency virus
LEDGF: lens epithelium–derived growth factor
MLV: murine leukemia virus
VSV-G: vesicular stomatitis virus G protein
