# Supplementary material for: Retroviral DNA Integration: Viral and Cellular Determinants of Target-Site Selection
Source: PLoS Pathog. 2006 Jun 23;2(6):e60. doi: 10.1371/journal.ppat.0020060 (PMC1480600; doi:10.1371/journal.ppat.0020060)
Supplement: Supplementary file 1 [file ppat.0020060.sd001.pdf]

Association of Genomic Features with Integration

Charles C. Berry

January 17, 2006

Contents

1 Introduction **2**

2 Preference for Genes **4**
2.1 Acembly Genes 4
2.2 refGenes 6
2.3 ensGenes 8
2.4 genScan Genes 9
2.5 uniGenes 11

3 CpG Island Neighborhoods **14**
3.1 1 kilobase neighborhoods 14
3.2 5 kilobase neighborhoods 15
3.3 10 kilobase neighborhoods 15
3.4 25 kilobase neighborhoods 16
3.5 50 kilobase neighborhoods 17

4 Gene Density, Expression 'Density', and CpG Island Density **19**
4.1 25 kilobase Window 19
4.2 50 kilobase Window 24
4.3 100 kilobase Window 29
4.4 250 kilobase Window 34
4.5 500 kilobase Window 39
4.6 1 megabase Window 44
4.7 2 megabase Window 49
4.8 4 megabase Window 54
4.9 8 megabase Window 59
4.10 16 megabase Window 64
4.11 32 megabase Window 69

5 Juxtaposition with Gene Start and End Positions **75**
5.1 Acembly Annotations 75
5.2 RefSeq Annotations 79
5.3 genScan Annotations 83
5.4 uniGene Annotations 87

6 GC content **91**

7 Cytobands **92**

1 Introduction

In this document, I examine the association of integration sites with various genomic features.

The data consist of both actual integration sites and sets of control sites, each set chosen to match the spacing (in bases) from the nearest restriction site (according to the direction in which the sequence was read) to an integration site. The numbers of insertion and matching sites for several data sets are shown below:

```
                  type
Origin.of.data.set insertion match
        HIVPuro          525  5240
        HIVmGag          493  4930
        HIVmGagmIN       526  5260
        HIVmIN           352  3500
        MLVPuro          544  5430
```

The advantage of choosing 'control' sites that match the spacing from the nearest restriction site is that biases due to location and density of restriction sites are eliminated by applying the classical multinomial logit model (reviewed in [2]). This model allows regression procedures to be applied to the study of integration intensity as a function of genomic features. The `clogit` function of the R `survival` library) implements estimation and fitting for such models along with the usual likelihood ratio and Wald tests.

The distribution of relative frequency of insertions across the chromosomes is given in this barplot:

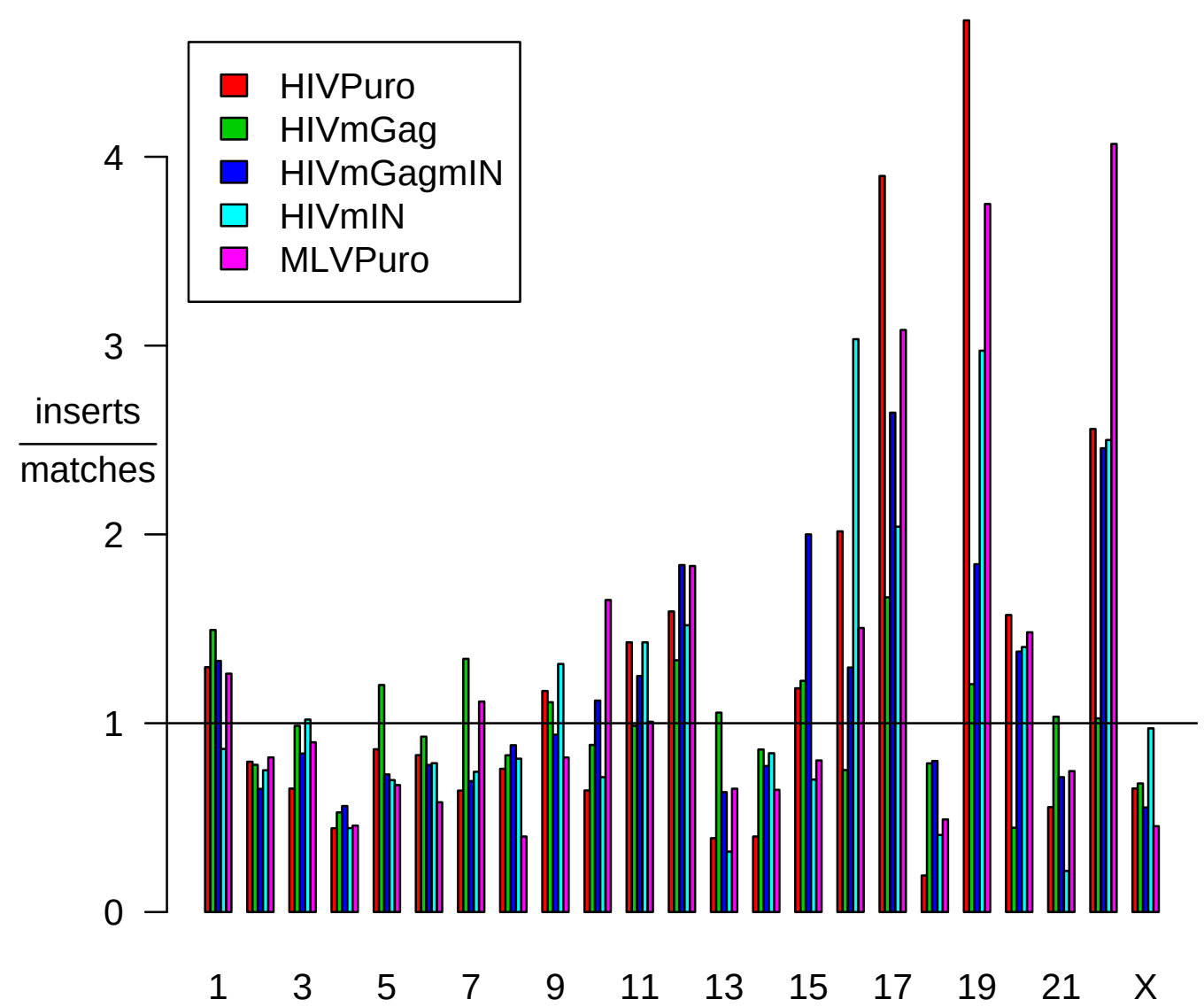

It seems evident that there are some chromosomes that are particularly favored for integration. This is reinforced by a test of statistical significance. The test performed used the likelihood ratio statistic for the multinomial logit model (reviewed in [2]) as implemented by the `clogit` function of the R `survival` library). The null hypothesis tested is that the ratio of true integration events to matched control sites is constant across all chromosomes. This test attains a p-value of $< 2.22e - 16$.

2 Preference for Genes

2.1 Acembly Genes

Here we examine the preference that integration events have for genes. In the following plot we show the relative frequency of integrations in genes according to the 'Acembly' annotation. The bars grouped over the label "In Gene" give the relative frequency of integration events (compared to control sites) between bases located within Acembly gene annotations, while the label "Not in Gene" give the relative frequency of integration events (compared to control sites) between bases not located within Acembly gene annotations.

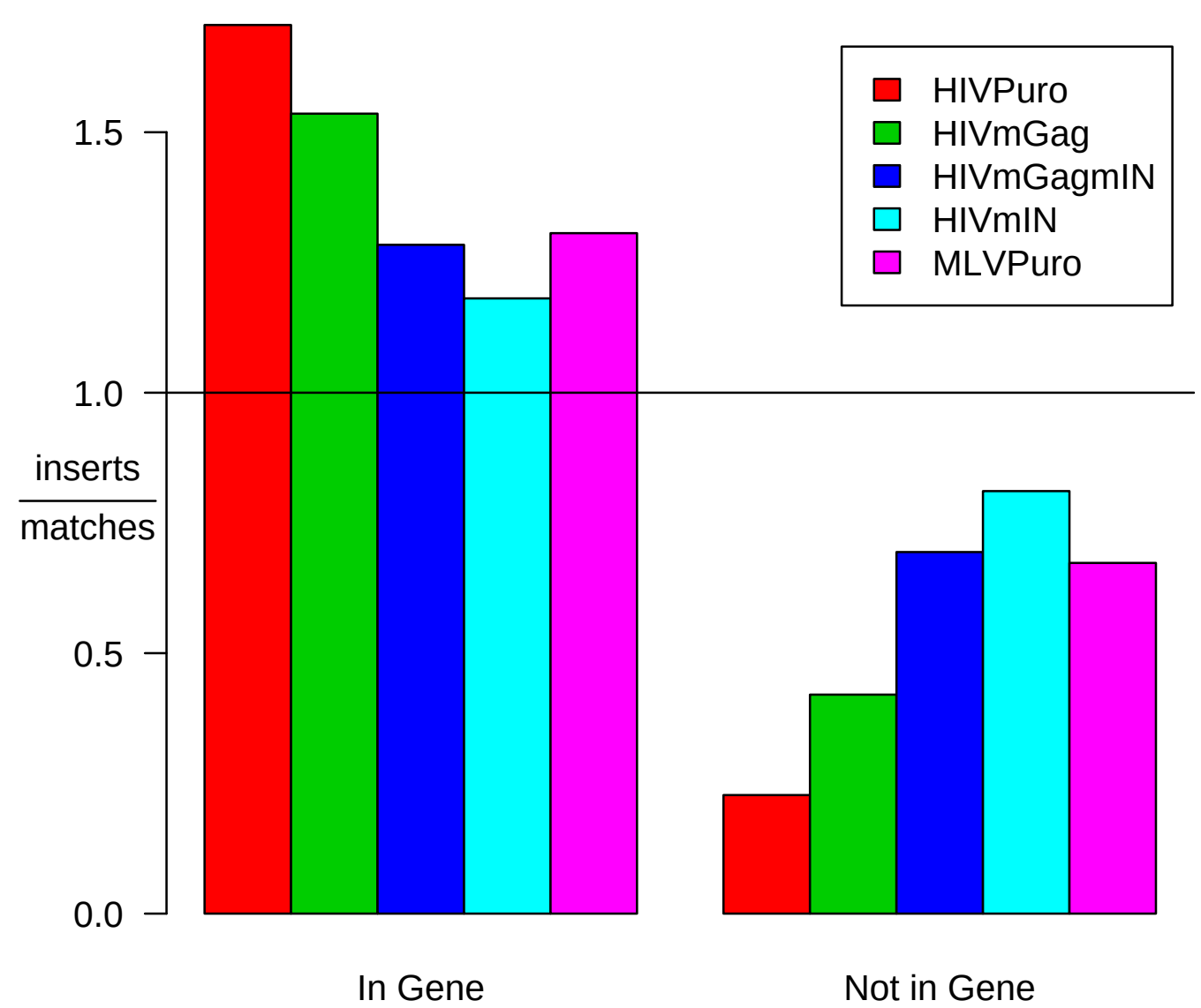

It seems evident that there is a strong tendency for insertions to occur in genes. A formal test of significance bears this out with a p-value of $< 2.22e-16$. Also, it appears that the tendency of different viruses to integrate into genes varies, and a test for this hypothesis attains $< 2.22e - 16$. Here is the table of coefficients of the log ratio of intensities for true insertion sites versus control insertion sites along with their standard errors, z statistics, and p-values for each data set:

```
            coef     se     z        p
HIVPuro    2.010 0.1430 14.10 6.25e-45
HIVmGag    1.300 0.1160 11.20 3.46e-29
HIVmGagmIN 0.615 0.0963  6.38 1.76e-10
```

HIVmIN	0.382	0.1150	3.31	9.39e-04
MLVPuro	0.670	0.0958	7.00	2.59e-12

As is evident, there are some differences in the coefficients. The largest coefficient is seen in the HIVPuro data set, while the smallest is seen in the HIVmIN data set.

In the following plot we show the relative frequency of insertions in exons according to the 'Acembly' annotation. The bars grouped over the label "In Exon" give the relative frequency of integration events (compared to control sites) between bases located in exons according to the Acembly annotation, while the label "Not in Exon" give the relative frequency of integration events (compared to control sites) between bases not located in exons according to the Acembly gene annotation.

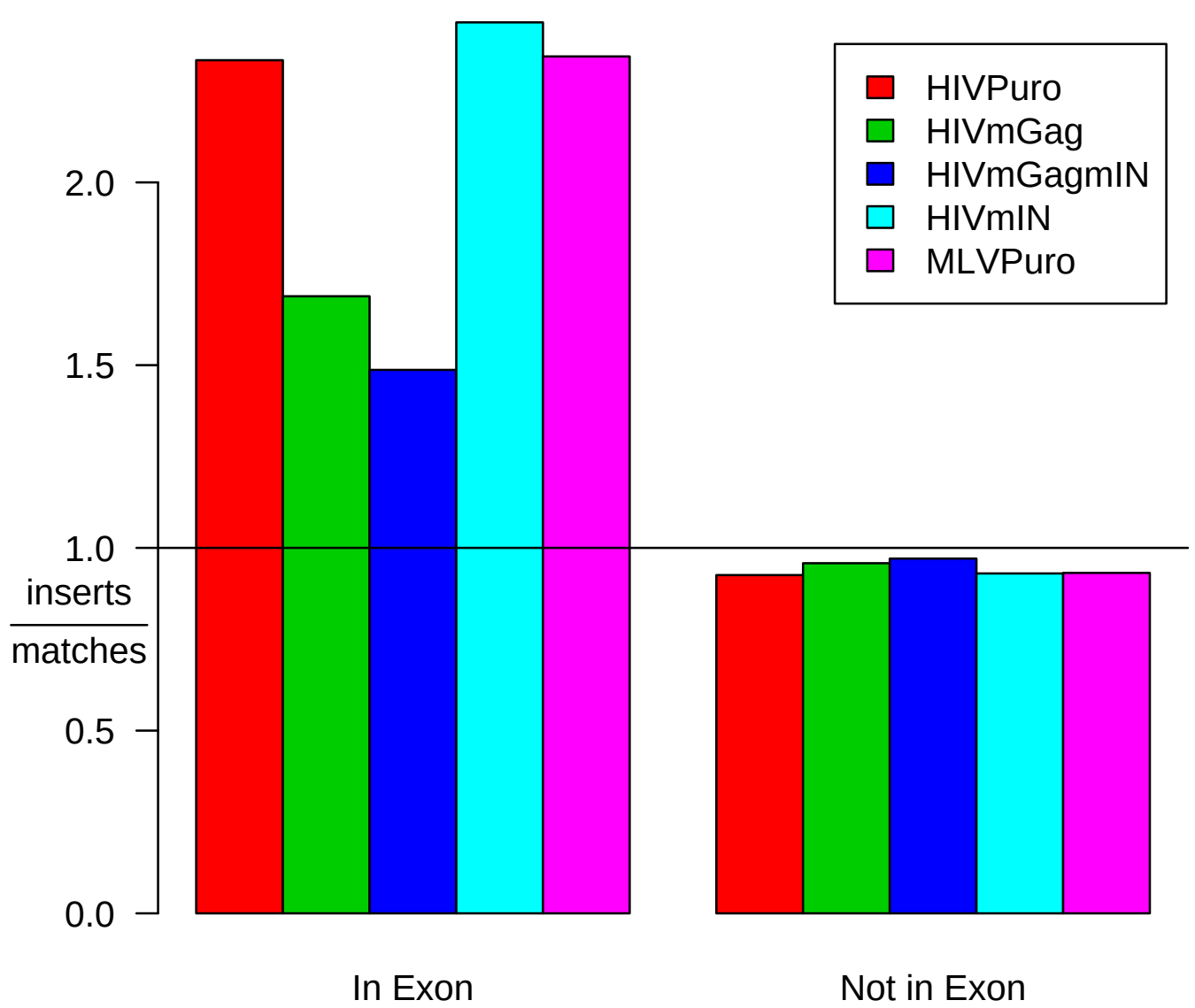

Here is the table of coefficients of the log ratio of intensities for true insertion sites versus control insertion sites along with their standard errors, z statistics, and p-values for each data set:

	coef	se	z	p
HIVPuro	0.367	0.149	2.460	1.37e-02
HIVmGag	0.110	0.170	0.646	5.18e-01
HIVmGagmIN	0.171	0.176	0.968	3.33e-01
HIVmIN	0.844	0.189	4.460	8.15e-06
MLVPuro	0.681	0.155	4.390	1.15e-05

The model on which these coefficients are based include terms for whether the site is in a gene or not. Thus, the effect shown as ”In Exon” is net of that due to being in a gene. Note that in the barplot above the ’Not in Exon’ bars include both the introns and intergenic regions, so the impression given by the table may differ from that for the barplot.

2.2 refGenes

Here we examine the preference that insertions have for genes. In the following plot we show the relative frequency of insertions in genes according to the ’refGene’ annotation.

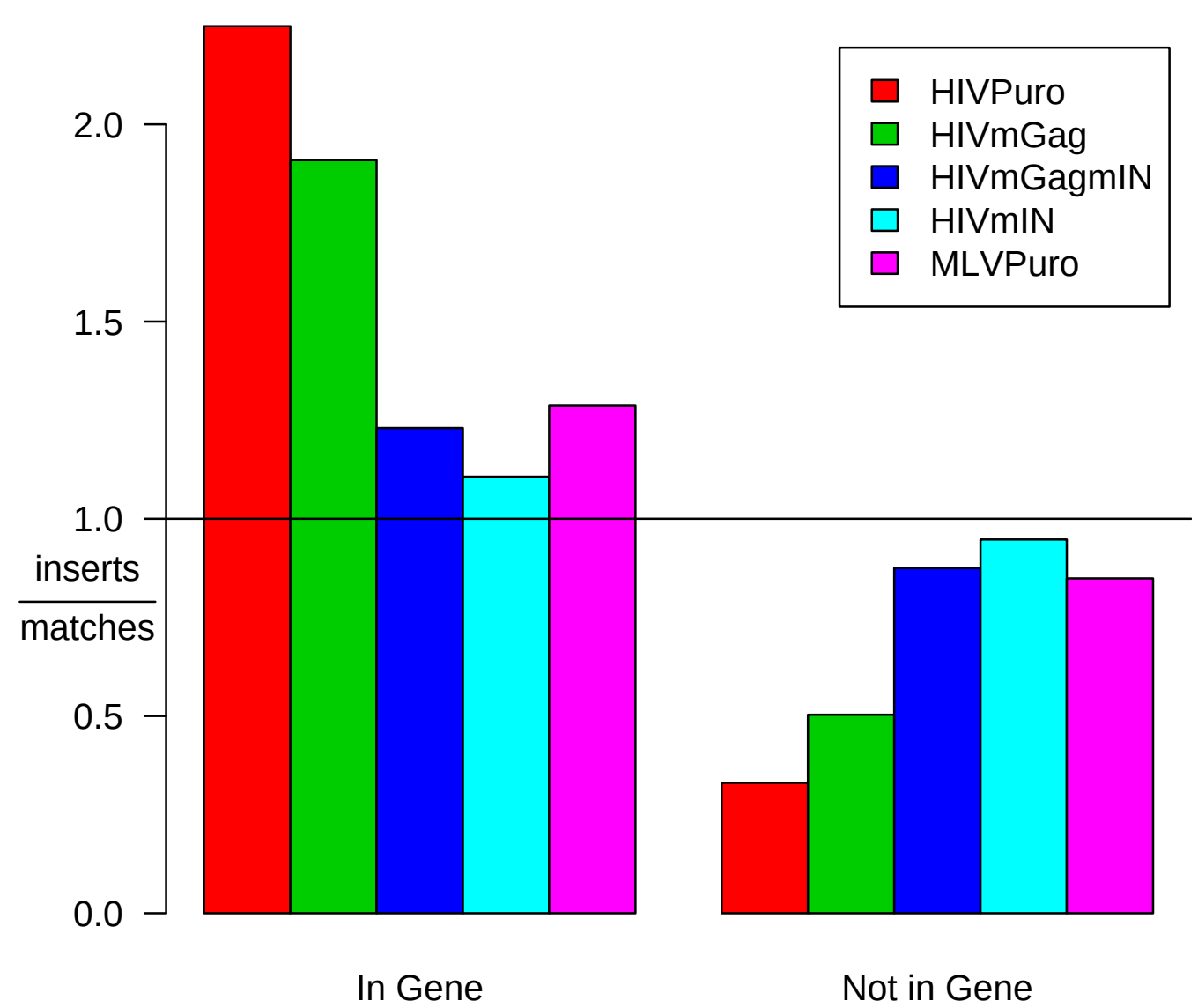

It seems evident that there is a strong tendency for insertions to occur in genes. A formal test of significance bears this out with a p-value of $< 2.22e-16$. Also, it appears that the tendency of different viruses to integrate into genes varies, and a test for this hypothesis attains $< 2.22e-16$. Here is the table of coefficients of the log ratio of intensities for true insertion sites versus control insertion sites along with their standard errors, z statistics, and p-values for each data set:

```
            coef     se     z         p
HIVPuro    1.910 0.1110 17.20 1.79e-66
HIVmGag    1.340 0.1010 13.20 1.21e-39
HIVmGagmIN 0.338 0.0926  3.65 2.58e-04
```

```
HIVmIN      0.169 0.1170  1.45 1.47e-01
MLVPuro     0.417 0.0907  4.60 4.25e-06
```

As is evident, there are some differences in the coefficients. The largest coefficient is seen in the HIVPuro data set, while the smallest is seen in the HIVmIN data set.

In the following plot we show the relative frequency of insertions in exons according to the 'refGene' annotation.

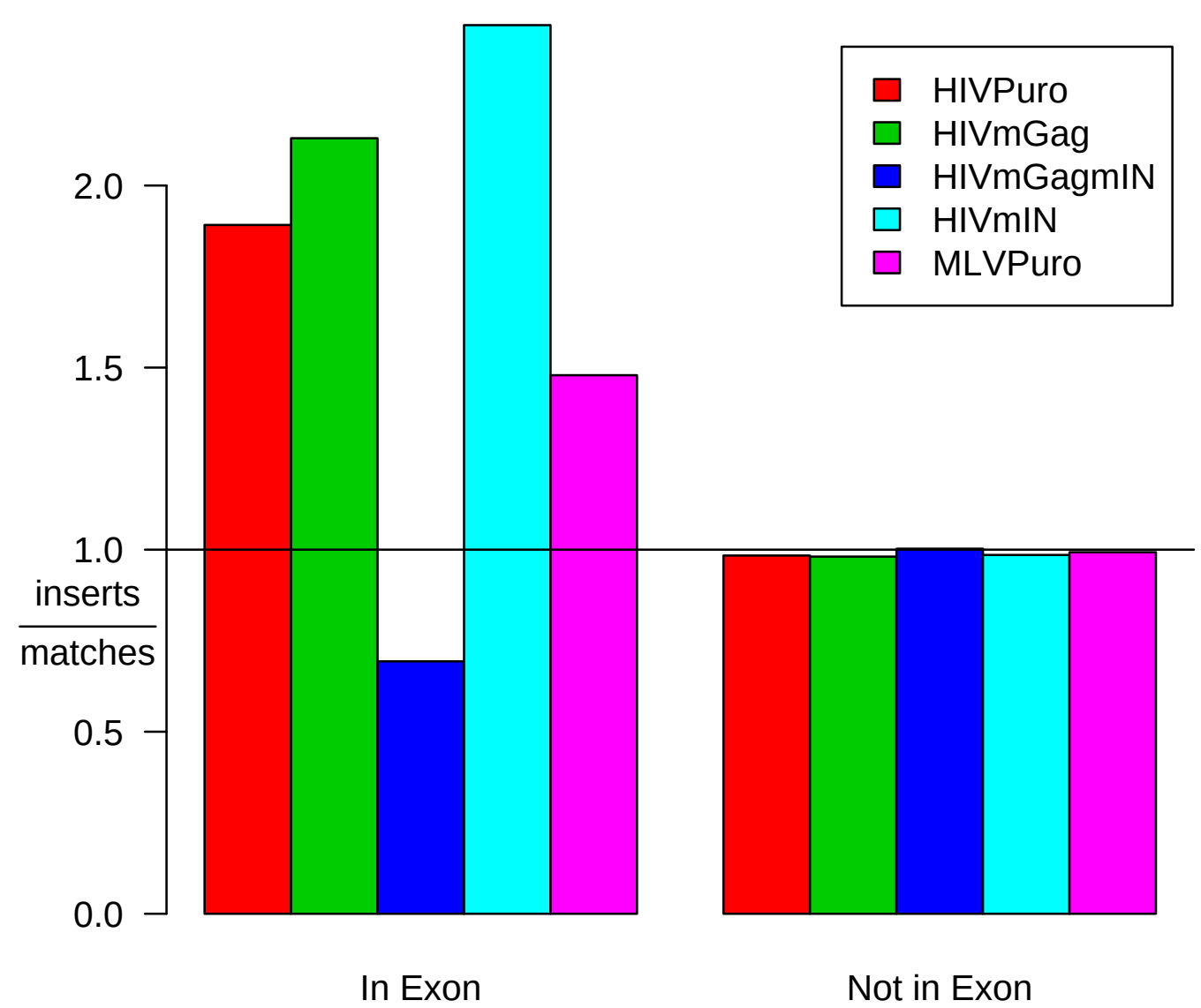

Here is the table of coefficients of the log ratio of intensities for true insertion sites versus control insertion sites along with their standard errors, z statistics, and p-values for each data set:

	coef	se	z	p
HIVPuro	-0.164	0.265	-0.617	0.5370
HIVmGag	0.106	0.281	0.376	0.7070
HIVmGagmIN	-0.595	0.468	-1.270	0.2040
HIVmIN	0.851	0.353	2.410	0.0159
MLVPuro	0.147	0.318	0.462	0.6440

The model on which these coefficients are based include terms for whether the site is in a gene or not. Thus, the effect shown as "In Exon" is net of that due to being in a gene.

2.3 ensGenes

Here we examine the preference that insertions have for genes. In the following plot we show the relative frequency of insertions in genes according to the 'ensGene' annotation.

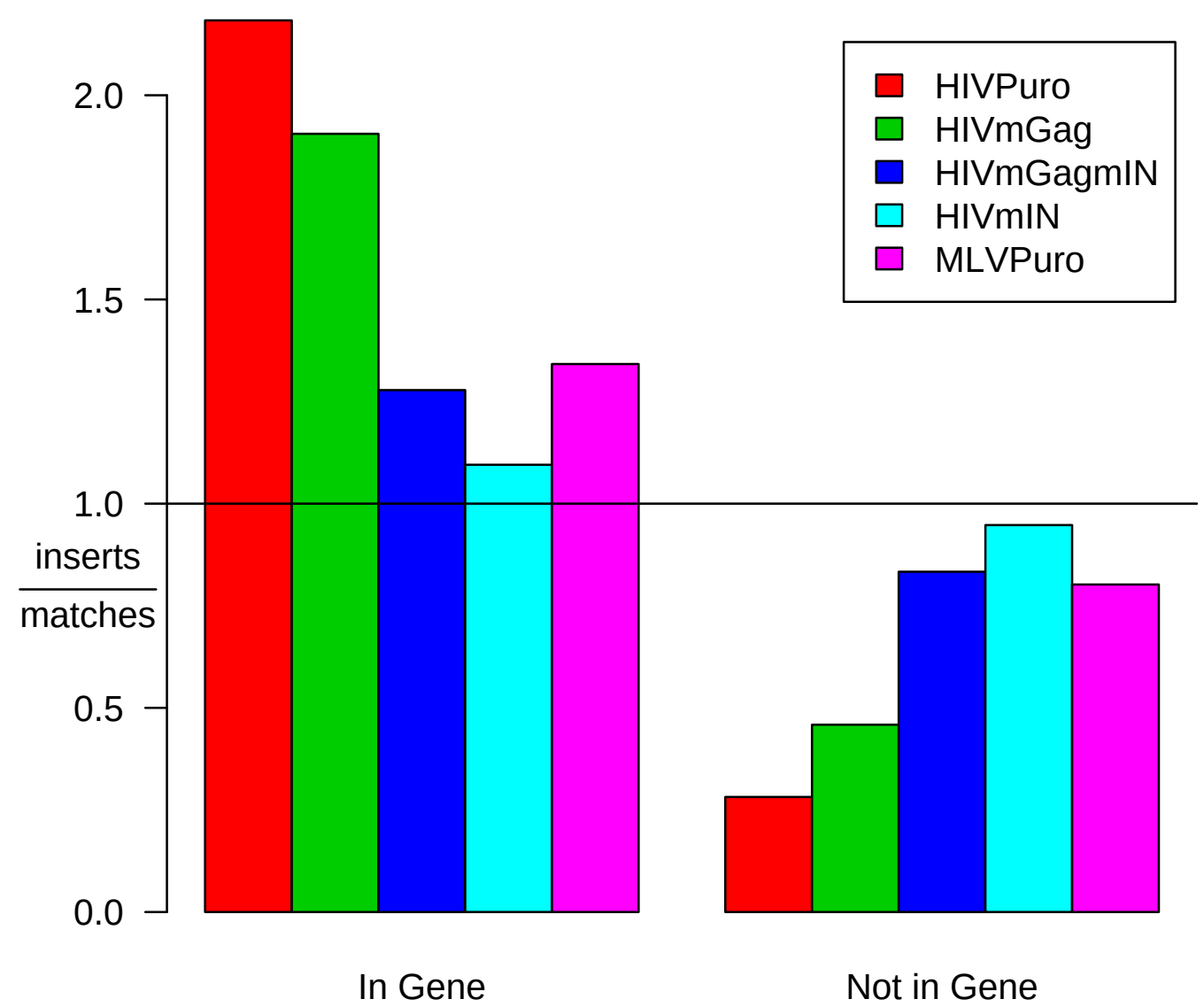

It seems evident that there is a strong tendency for insertions to occur in genes. A formal test of significance bears this out with a p-value of $< 2.22e-16$. Also, it appears that the tendency of different viruses to integrate into genes varies, and a test for this hypothesis attains $< 2.22e-16$. Here is the table of coefficients of the log ratio of intensities for true insertion sites versus control insertion sites along with their standard errors, z statistics, and p-values for each data set:

	coef	se	z	p
HIVPuro	2.050	0.1190	17.20	5.03e-66
HIVmGag	1.420	0.1040	13.70	1.79e-42
HIVmGagmIN	0.426	0.0916	4.65	3.40e-06
HIVmIN	0.157	0.1150	1.37	1.70e-01
MLVPuro	0.515	0.0900	5.72	1.06e-08

As is evident, there are some differences in the coefficients. The largest coefficient is seen in the HIVPuro data set, while the smallest is seen in the HIVmIN data set.

In the following plot we show the relative frequency of insertions in exons according to the 'ensGene' annotation.

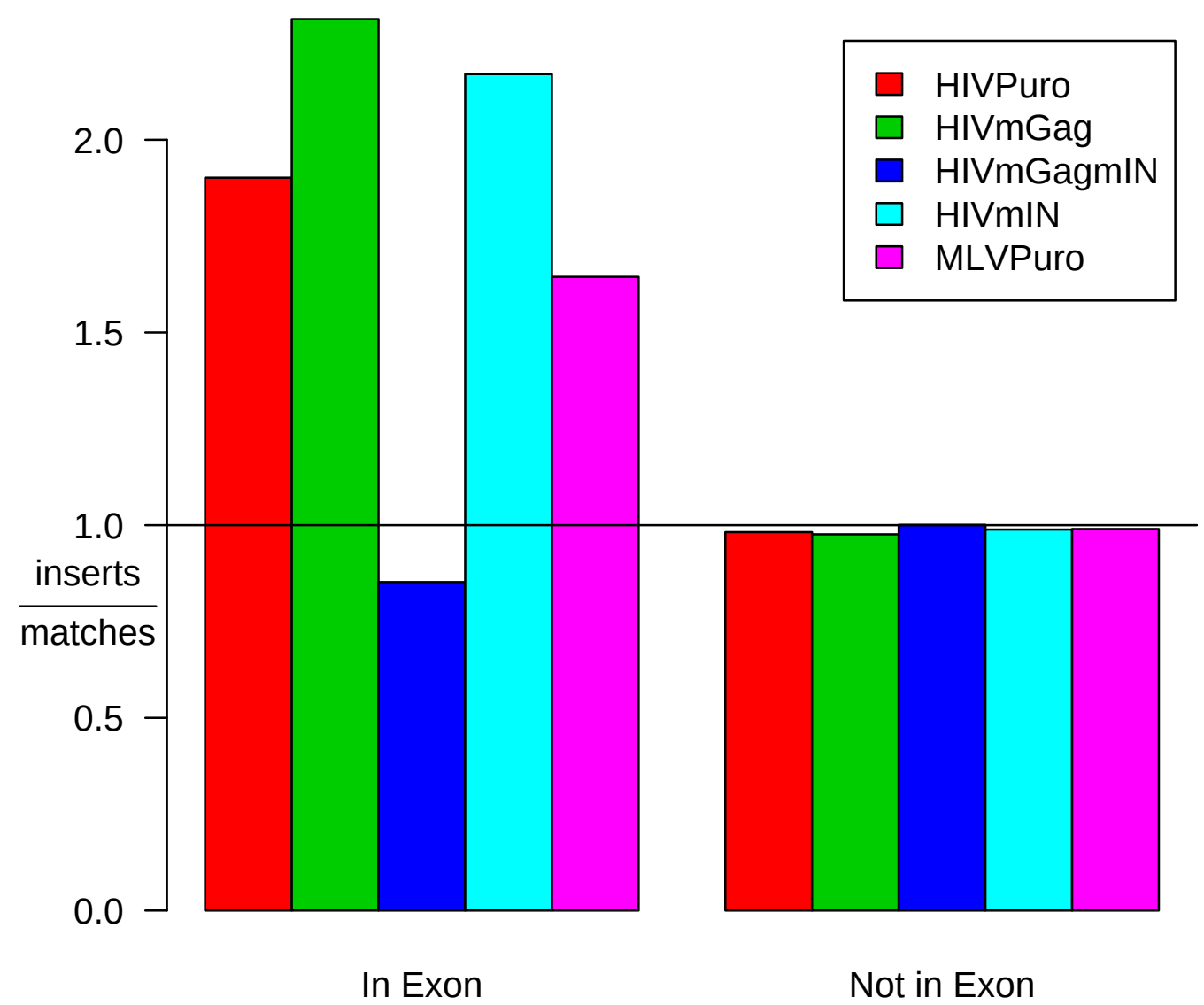

Here is the table of coefficients of the log ratio of intensities for true insertion sites versus control insertion sites along with their standard errors, z statistics, and p-values for each data set:

	coef	se	z	p
HIVPuro	-0.118	0.255	-0.462	0.6440
HIVmGag	0.197	0.260	0.756	0.4500
HIVmGagmIN	-0.422	0.401	-1.050	0.2920
HIVmIN	0.725	0.361	2.010	0.0447
MLVPuro	0.217	0.296	0.733	0.4640

The model on which these coefficients are based include terms for whether the site is in a gene or not. Thus, the effect shown as "In Exon" is net of that due to being in a gene.

2.4 genScan Genes

Here we examine the preference that insertions have for genes. In the following plot we show the relative frequency of insertions in genes according to the 'genScan' annotation.

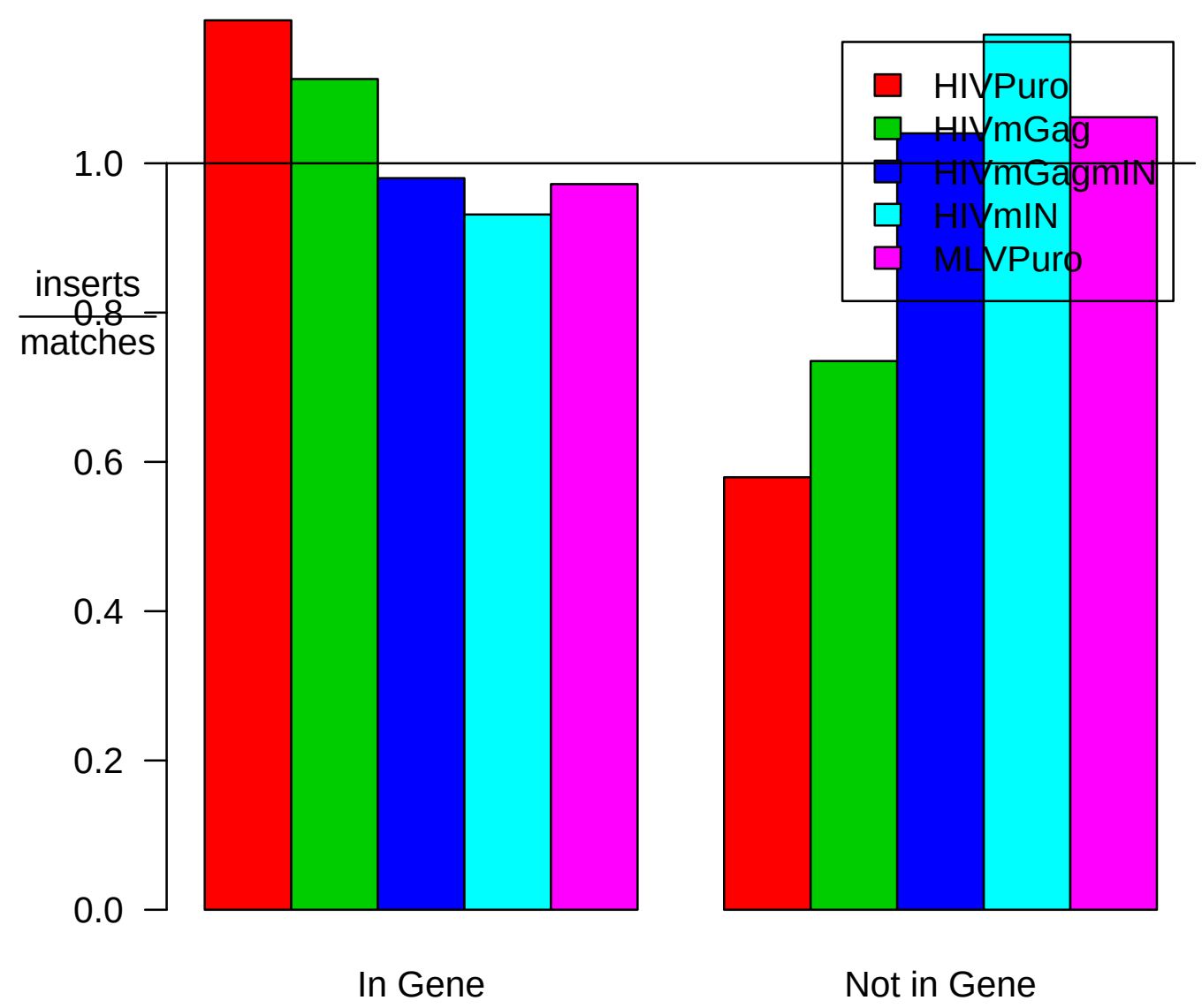

It seems evident that there is a strong tendency for insertions to occur in genes. A formal test of significance bears this out with a p-value of 0.0019923. Also, it appears that the tendency of different viruses to integrate into genes varies, and a test for this hypothesis attains $1.2775e-10$. Here is the table of coefficients of the log ratio of intensities for true insertion sites versus control insertion sites along with their standard errors, z statistics, and p-values for each data set:

	coef	se	z	p
HIVPuro	0.7170	0.1170	6.120	9.33e-10
HIVmGag	0.4150	0.1130	3.690	2.28e-04
HIVmGagmIN	-0.0590	0.0979	-0.602	5.47e-01
HIVmIN	-0.2250	0.1180	-1.920	5.53e-02
MLVPuro	-0.0907	0.0955	-0.949	3.42e-01

As is evident, there are some differences in the coefficients. The largest coefficient is seen in the HIVPuro data set, while the smallest is seen in the HIVmIN data set.

In the following plot we show the relative frequency of insertions in exons according to the 'genScan' annotation.

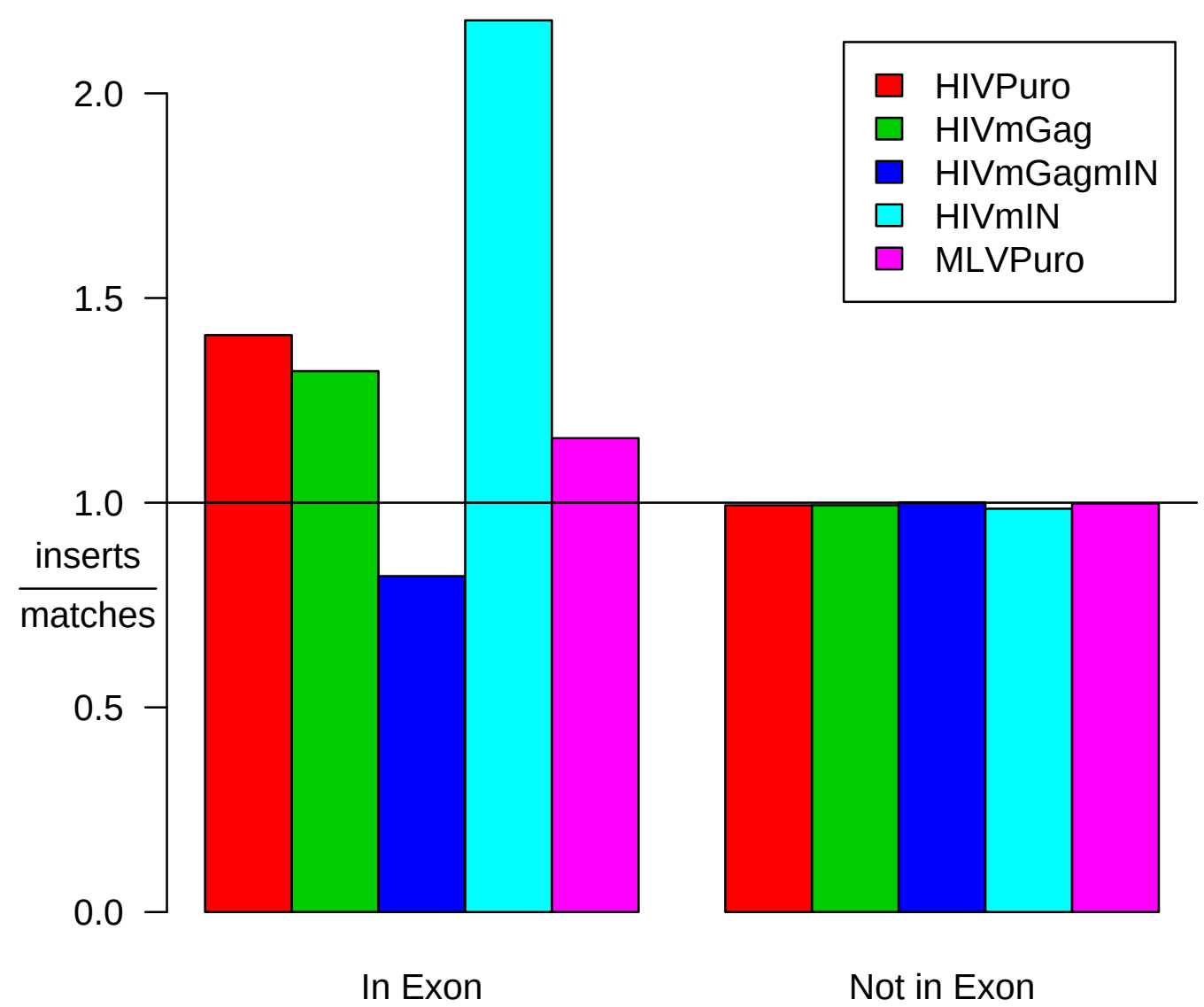

Here is the table of coefficients of the log ratio of intensities for true insertion sites versus control insertion sites along with their standard errors, z statistics, and p-values for each data set:

	coef	se	z	p
HIVPuro	0.182	0.314	0.581	0.56100
HIVmGag	0.181	0.358	0.506	0.61300
HIVmGagmIN	-0.181	0.427	-0.424	0.67200
HIVmIN	0.886	0.327	2.710	0.00671
MLVPuro	0.180	0.378	0.475	0.63500

The model on which these coefficients are based include terms for whether the site is in a gene or not. Thus, the effect shown as ”In Exon” is net of that due to being in a gene.

2.5 uniGenes

Here we examine the preference that insertions have for genes. In the following plot we show the relative frequency of insertions in genes according to the 'uniGene' annotation.

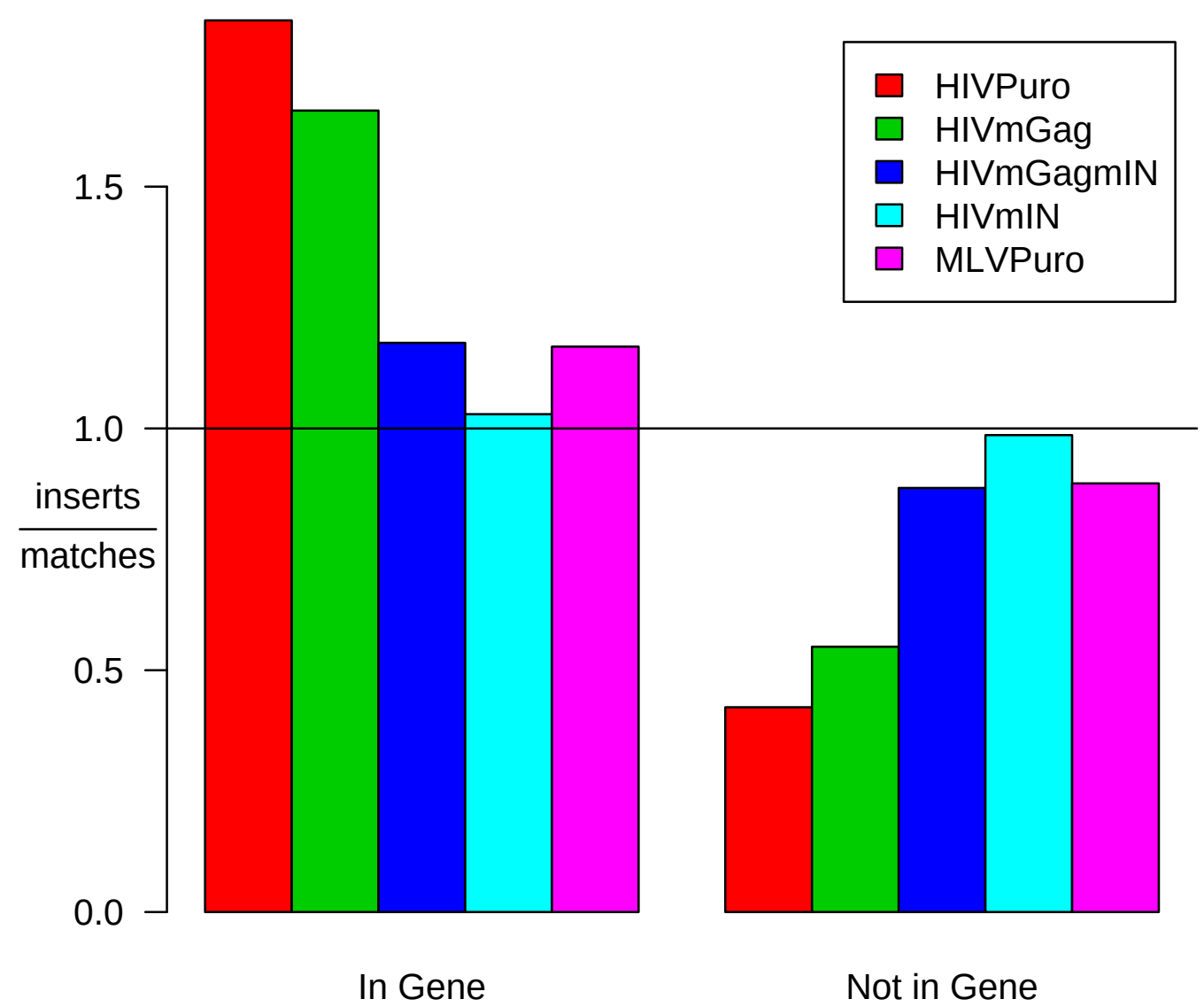

It seems evident that there is a strong tendency for insertions to occur in genes. A formal test of significance bears this out with a p-value of $< 2.22e-16$. Also, it appears that the tendency of different viruses to integrate into genes varies, and a test for this hypothesis attains $< 2.22e-16$. Here is the table of coefficients of the log ratio of intensities for true insertion sites versus control insertion sites along with their standard errors, z statistics, and p-values for each data set:

	coef	se	z	p
HIVPuro	1.4700	0.1050	14.000	1.50e-44
HIVmGag	1.1100	0.1010	11.000	4.83e-28
HIVmGagmIN	0.2930	0.0915	3.200	1.38e-03
HIVmIN	0.0527	0.1130	0.465	6.42e-01
MLVPuro	0.2810	0.0904	3.110	1.87e-03

As is evident, there are some differences in the coefficients. The largest coefficient is seen in the HIVPuro data set, while the smallest is seen in the HIVmIN data set.

In the following plot we show the relative frequency of insertions in exons according to the 'uniGene' annotation.

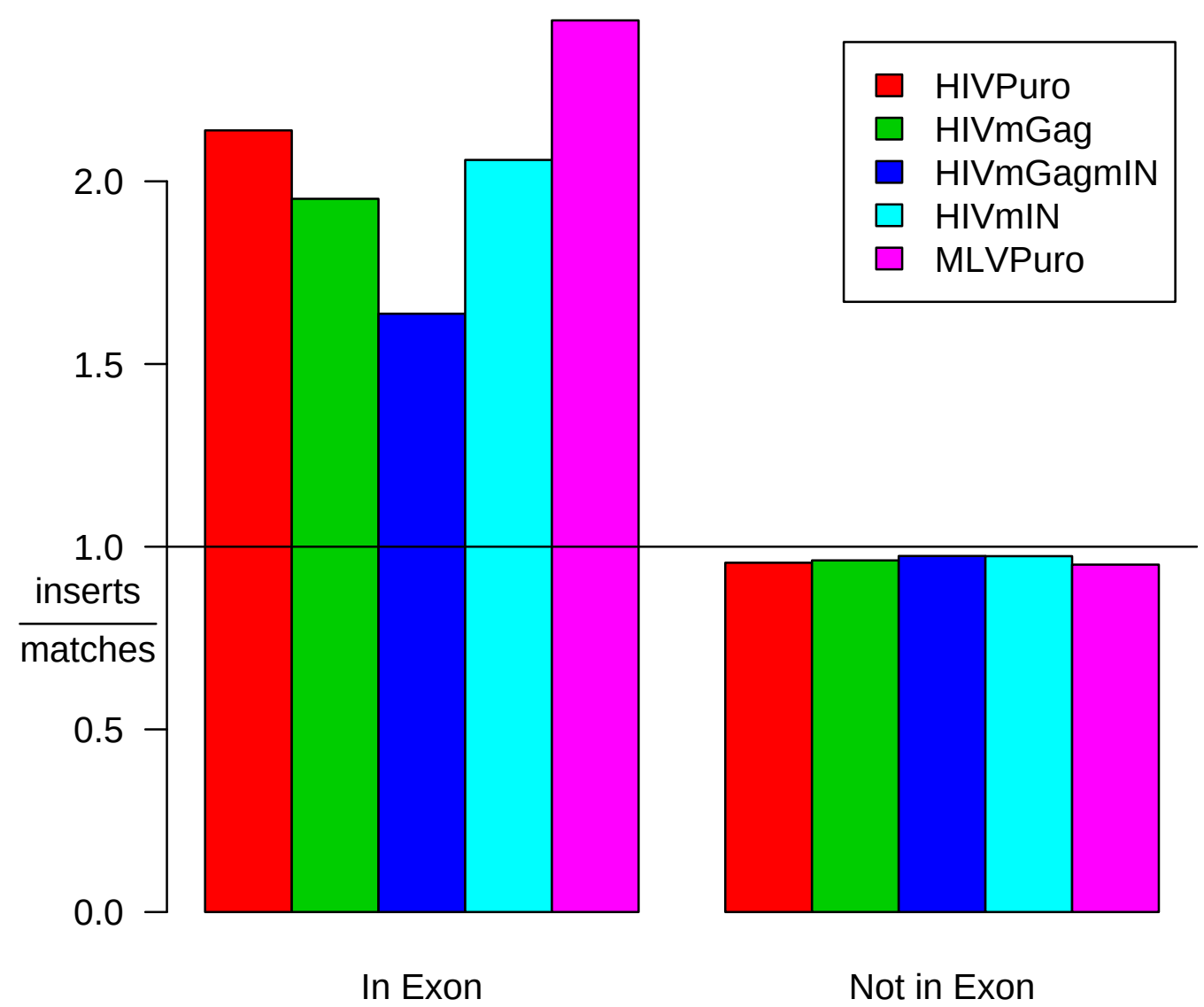

Here is the table of coefficients of the log ratio of intensities for true insertion sites versus control insertion sites along with their standard errors, z statistics, and p-values for each data set:

	coef	se	z	p
HIVPuro	0.156	0.183	0.849	3.96e-01
HIVmGag	0.189	0.195	0.971	3.31e-01
HIVmGagmIN	0.373	0.207	1.800	7.14e-02
HIVmIN	0.773	0.264	2.930	3.38e-03
MLVPuro	0.843	0.184	4.590	4.41e-06

The model on which these coefficients are based include terms for whether the site is in a gene or not. Thus, the effect shown as ”In Exon” is net of that due to being in a gene.

3 CpG Island Neighborhoods

Here we study the effect of being in the neighborhood of CpG Islands. Following Wu et al [3], who found that the neighborhoods within ±1kb of CpG islands are enriched for MLV insertions, we study such neighborhoods.

3.1 1 kilobase neighborhoods

The following plot shows the effect of being in or within ±1kb of a CpG island:

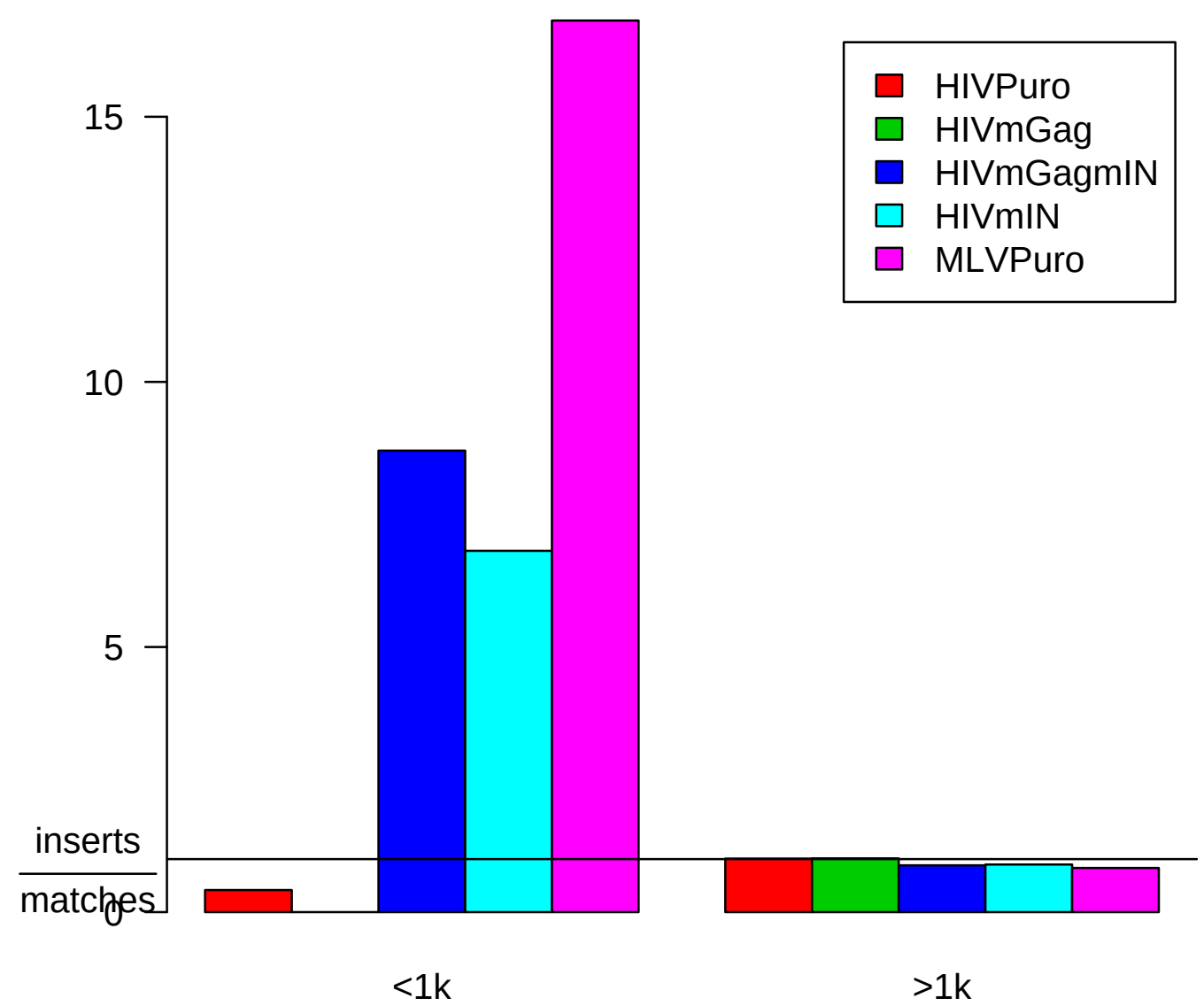

A formal test of significance comparing the difference attains a p-value of $< 2.22e-16$. A test for differences between viruses attains $< 2.22e-16$. Here is the table of coefficients of the log ratio of intensities for true insertion sites versus control insertion sites along with their standard errors, z statistics, and p-values for each data set:

	coef	se	z	p
HIVPuro	-0.877	0.590	-1.4900	1.37e-01
HIVmGag	-15.400	825.000	-0.0187	9.85e-01
HIVmGagmIN	2.300	0.176	13.0000	7.70e-39
HIVmIN	2.090	0.215	9.7400	2.05e-22
MLVPuro	2.990	0.182	16.5000	6.70e-61

The largest coefficient is seen in the MLVPuro data set, while the smallest is seen in the HIVmGag data set.

3.2 5 kilobase neighborhoods

The following plot shows the effect of being in or within ±5kb of a CpG island:

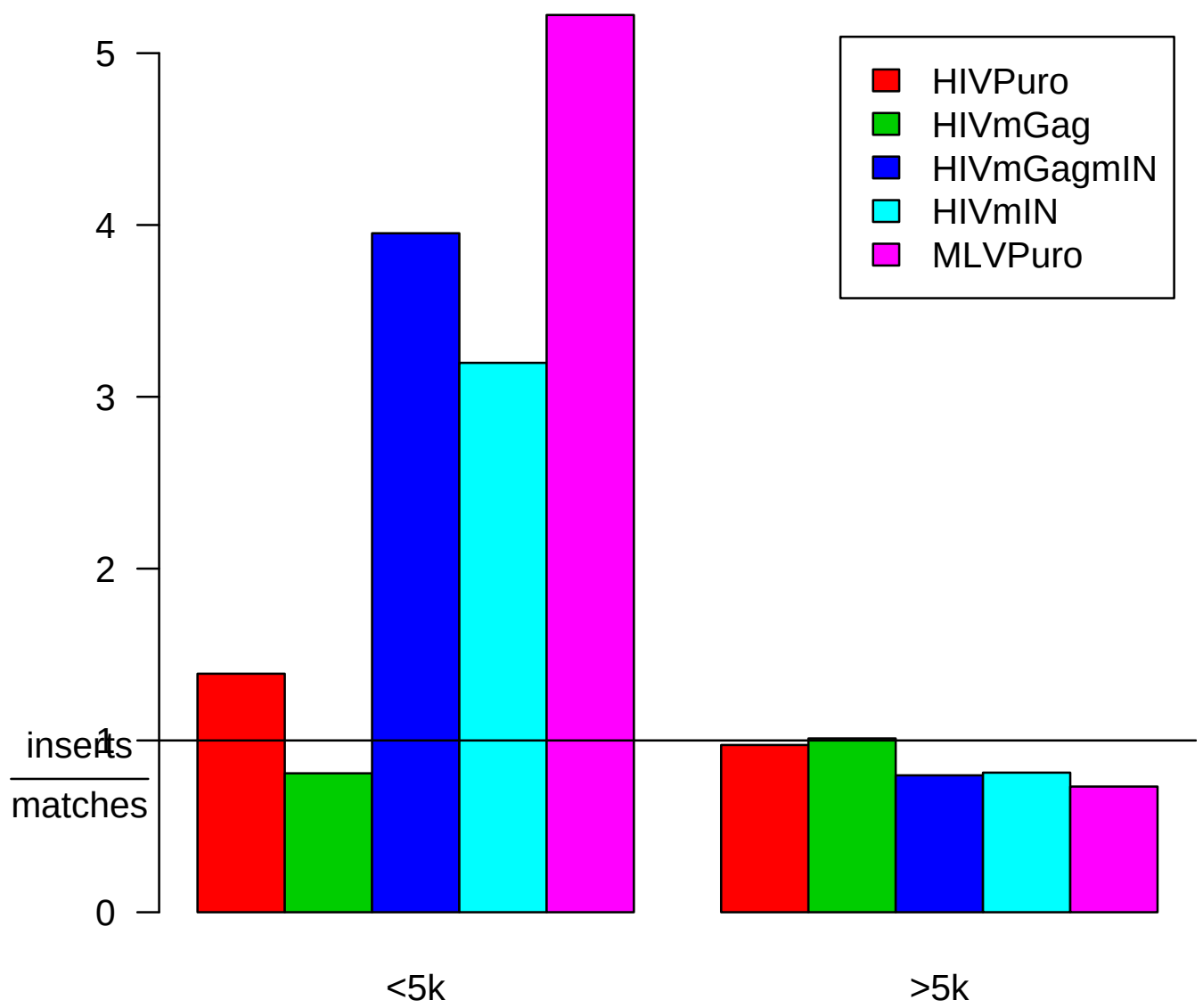

A formal test of significance comparing the difference attains a p-value of $< 2.22e-16$. A test for differences between viruses attains $< 2.22e-16$. Here is the table of coefficients of the log ratio of intensities for true insertion sites versus control insertion sites along with their standard errors, z statistics, and p-values for each data set:

	coef	se	z	p
HIVPuro	0.358	0.163	2.19	2.83e-02
HIVmGag	-0.223	0.209	-1.07	2.86e-01
HIVmGagmIN	1.600	0.116	13.80	2.85e-43
HIVmIN	1.390	0.139	10.00	1.60e-23
MLVPuro	1.960	0.112	17.60	4.78e-69

The largest coefficient is seen in the MLVPuro data set, while the smallest is seen in the HIVmGag data set.

3.3 10 kilobase neighborhoods

The following plot shows the effect of being in or within ±10kb of a CpG island:

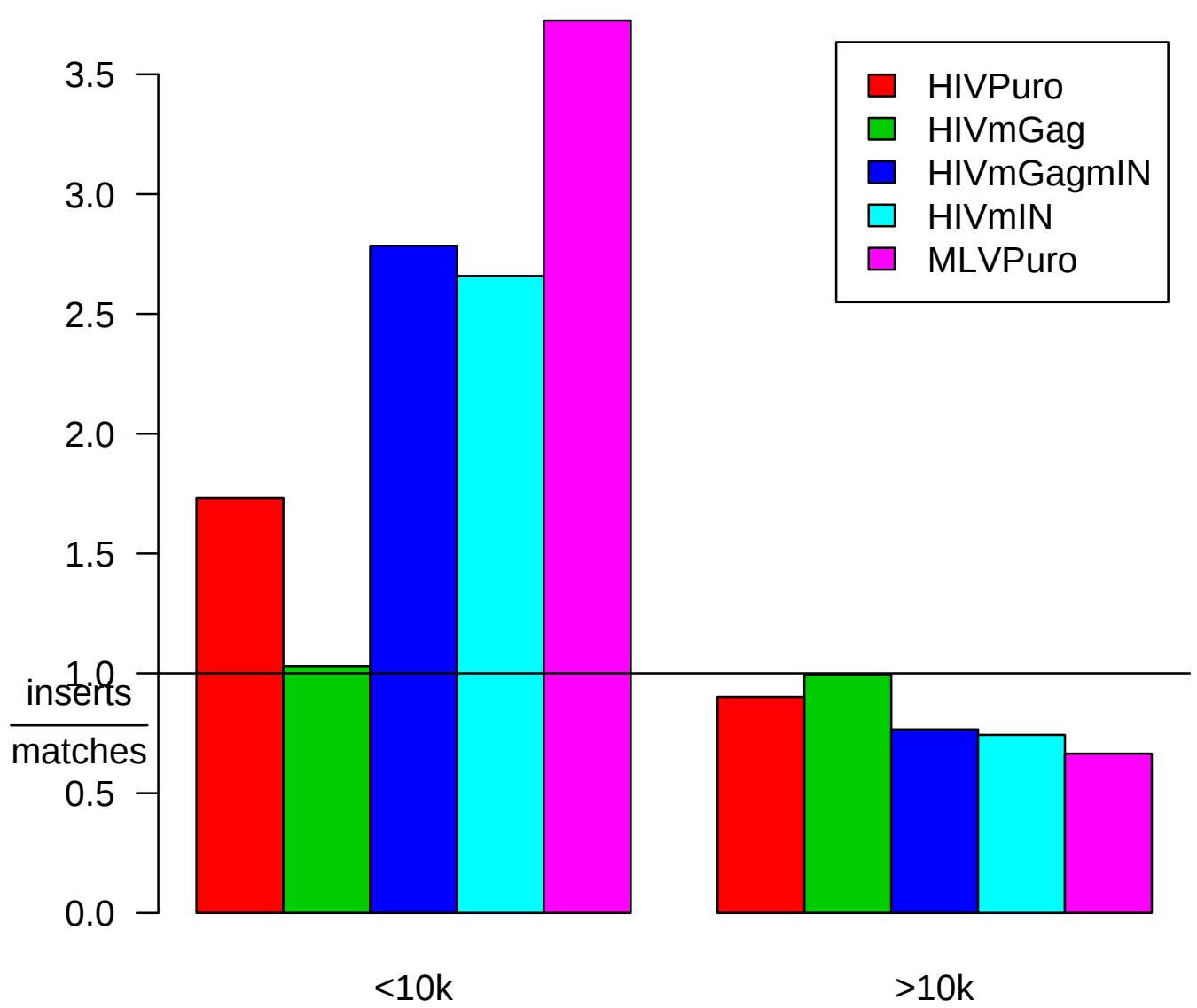

A formal test of significance comparing the difference attains a p-value of $< 2.22e - 16$. A test for differences between viruses attains $< 2.22e - 16$. Here is the table of coefficients of the log ratio of intensities for true insertion sites versus control insertion sites along with their standard errors, z statistics, and p-values for each data set:

	coef	se	z	p
HIVPuro	0.6540	0.1160	5.630	1.83e-08
HIVmGag	0.0352	0.1460	0.241	8.09e-01
HIVmGagmIN	1.3000	0.1040	12.500	8.13e-36
HIVmIN	1.2900	0.1230	10.500	8.17e-26
MLVPuro	1.7100	0.0989	17.300	3.35e-67

The largest coefficient is seen in the MLVPuro data set, while the smallest is seen in the HIVmGag data set.

3.4 25 kilobase neighborhoods

The following plot shows the effect of being in or within ±25kb of a CpG island:

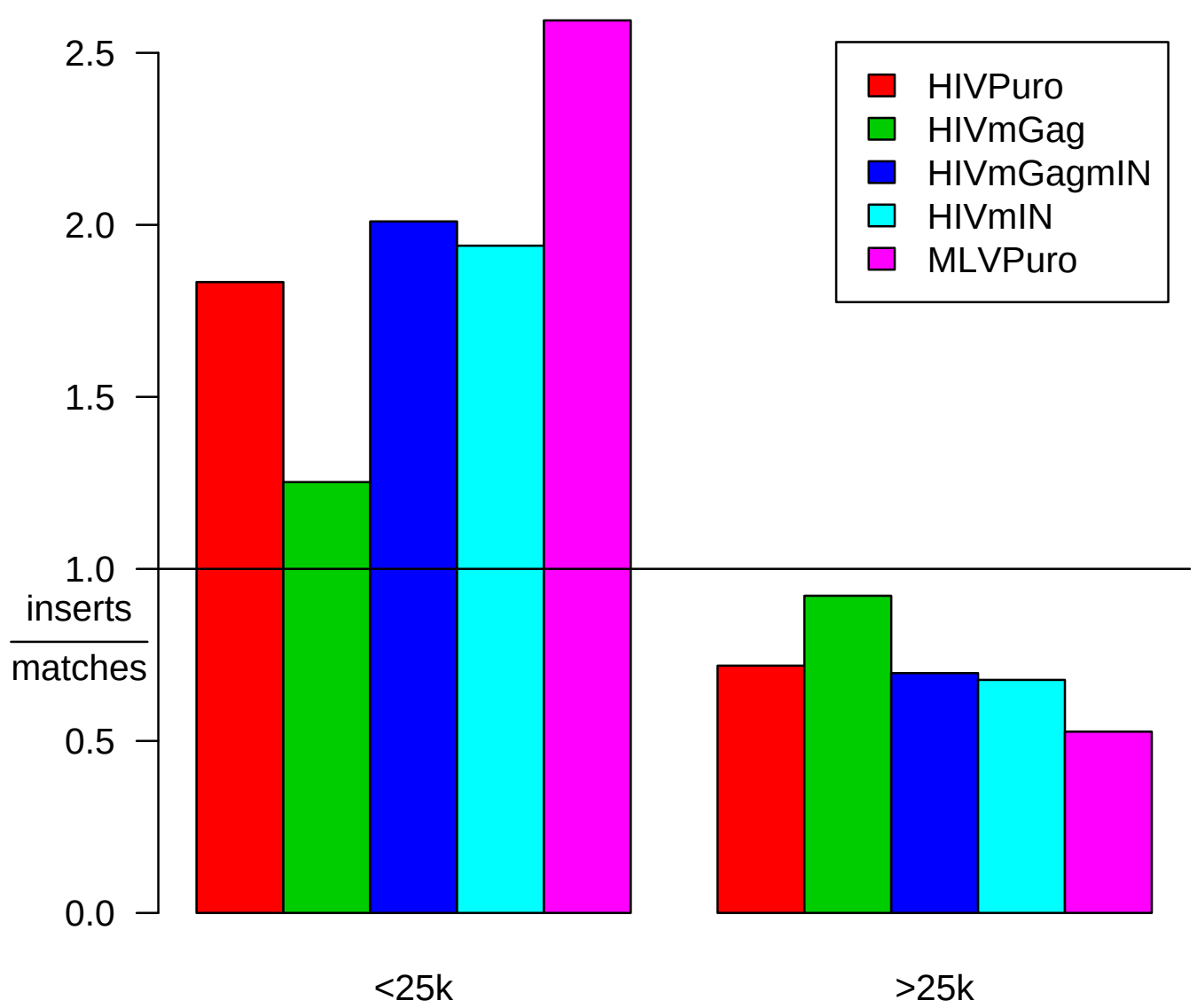

A formal test of significance comparing the difference attains a p-value of $< 2.22e - 16$. A test for differences between viruses attains $< 2.22e - 16$. Here is the table of coefficients of the log ratio of intensities for true insertion sites versus control insertion sites along with their standard errors, z statistics, and p-values for each data set:

	coef	se	z	p
HIVPuro	0.923	0.0930	9.93	3.04e-23
HIVmGag	0.307	0.1050	2.92	3.45e-03
HIVmGagmIN	1.050	0.0932	11.30	1.65e-29
HIVmIN	1.090	0.1160	9.41	4.76e-21
MLVPuro	1.590	0.0944	16.90	4.82e-64

The largest coefficient is seen in the MLVPuro data set, while the smallest is seen in the HIVmGag data set.

3.5 50 kilobase neighborhoods

The following plot shows the effect of being in or within ±50kb of a CpG island:

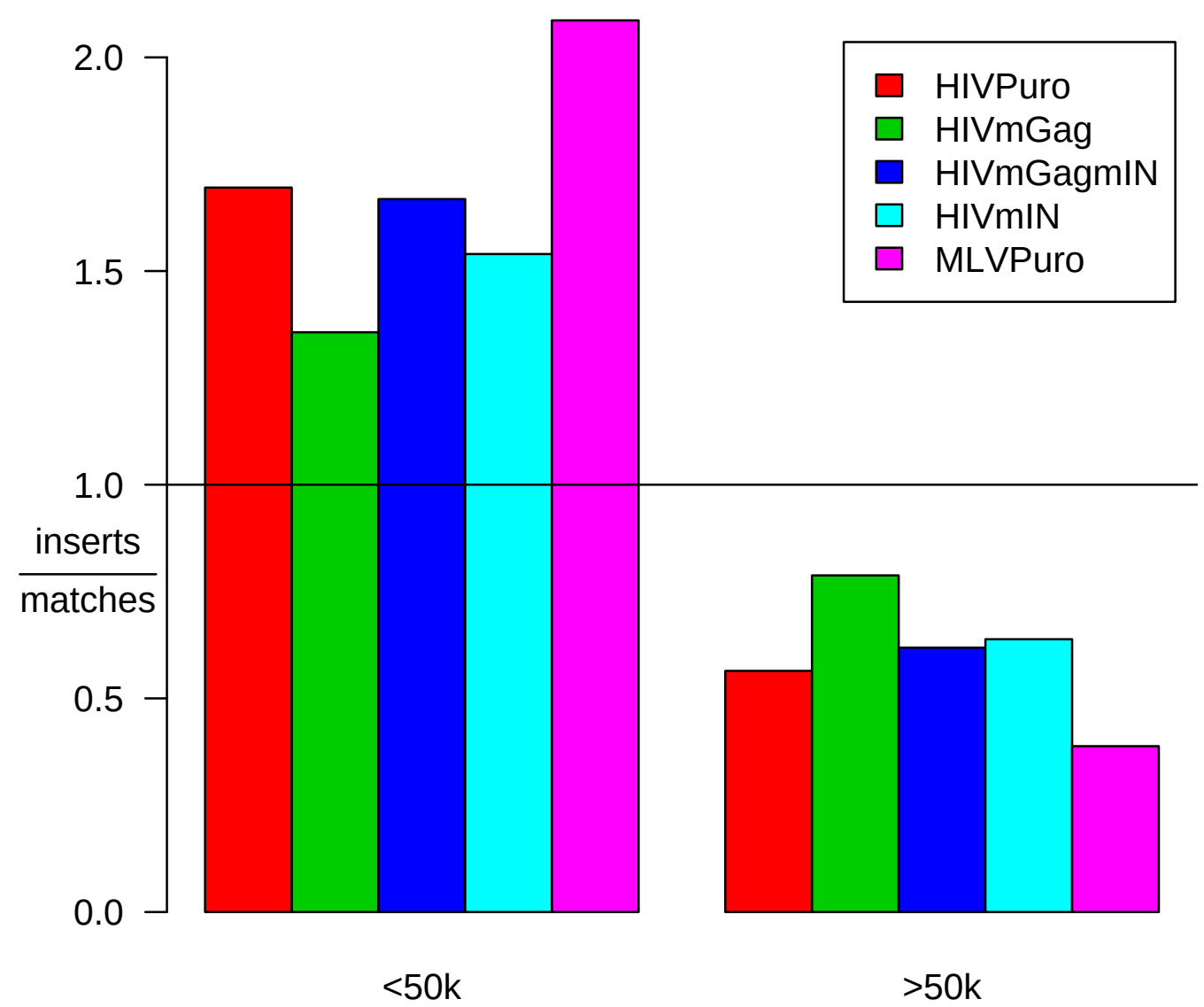

A formal test of significance comparing the difference attains a p-value of $< 2.22e - 16$. A test for differences between viruses attains $2.7179e - 14$. Here is the table of coefficients of the log ratio of intensities for true insertion sites versus control insertion sites along with their standard errors, z statistics, and p-values for each data set:

	coef	se	z	p
HIVPuro	1.100	0.0966	11.40	5.05e-30
HIVmGag	0.541	0.0947	5.72	1.08e-08
HIVmGagmIN	0.997	0.0941	10.60	3.30e-26
HIVmIN	0.909	0.1180	7.74	1.03e-14
MLVPuro	1.670	0.1030	16.20	9.87e-59

The largest coefficient is seen in the MLVPuro data set, while the smallest is seen in the HIVmGag data set.

4 Gene Density, Expression 'Density', and CpG Island Density

In this section the association with gene density is examined. The 'genes' that are counted are the genes represented on the Affymetric Hu133A microarray. In addition, we count the number of such genes expressed at various levels. The levels are

low.ex Count genes whose expression is in the upper half and divide by number of bases

med.ex Count genes whose expression is in the upper $1/8^{th}$ and divide by number of bases

high.ex Count genes whose expression is in the upper $1/16^{th}$ and divide by number of bases

The bolded terms are used as abbreviations in what follows. The abbreviation **dens** is used to indicate gene density as number of genes per base.

4.1 25 kilobase Window

In the barplot that follows we examine the association of insertion sites with gene density in a 25 kilobase window surrounding each locus. More such plots will follow and the method of their construction is always to try to divide the data according to the deciles of density. However, it often happens that there is a very skewed distribution of density and even the 90th percentile is zero. In that case, the barplots simply show the sites for which the density is zero and those for which it is non-zero. If there are fewer than ten groups of bars, the groupings contain ten percent of the sites each except for the leftmost grouping which will contain all of the remaining sites.

Also note that the title of the plot contains clues as to its content; the prefix indicates the type of variable studied while the suffix indicates the window width in the number of bases. The p-value given is the result of fitting a cubic polynomial to the gene density values.

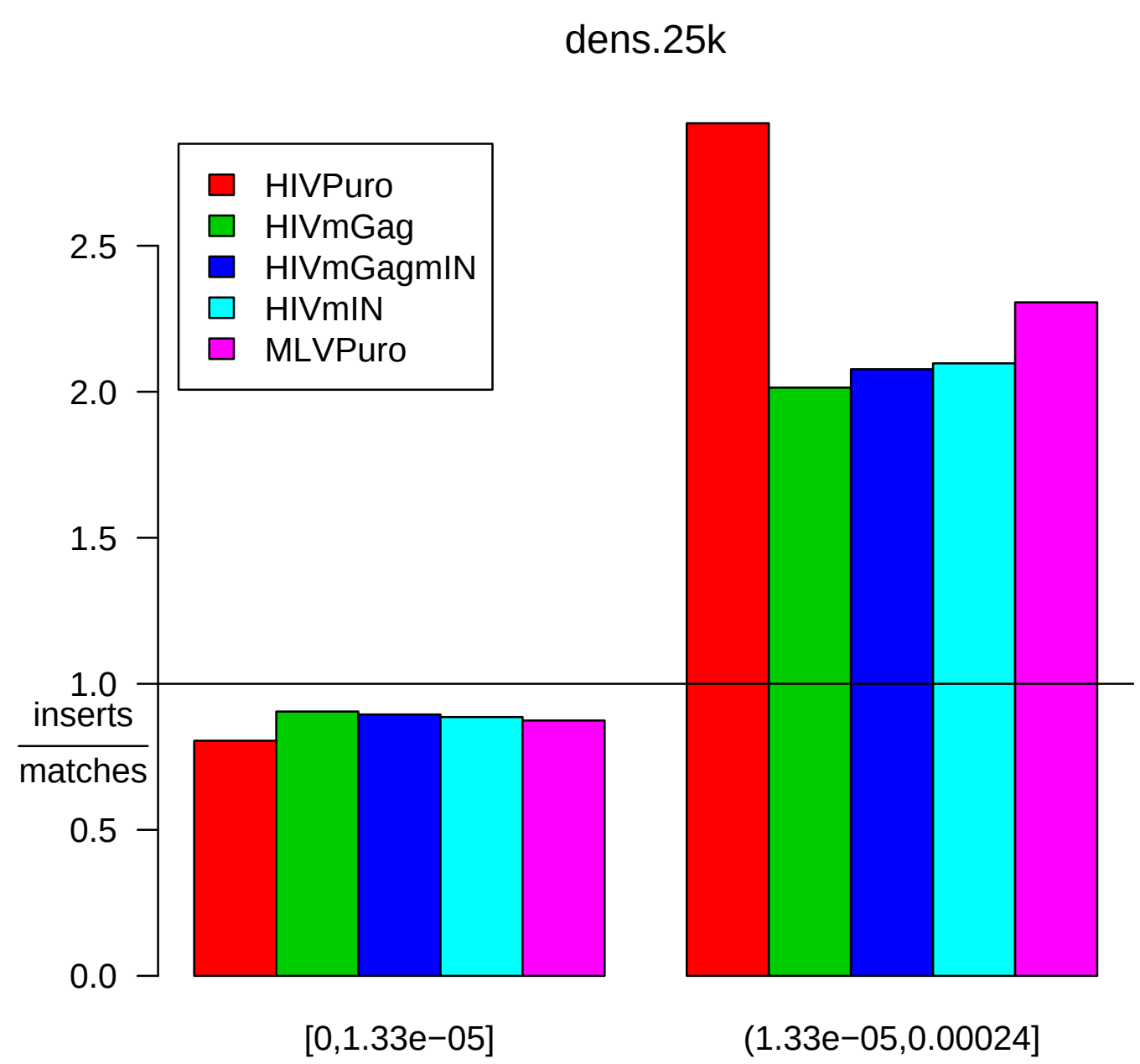

	coef	se	z	p
HIVPuro	1.390	0.104	13.30	2.67e-40
HIVmGag	0.916	0.118	7.78	6.99e-15
HIVmGagmIN	0.759	0.116	6.55	5.58e-11
HIVmIN	0.824	0.138	5.97	2.43e-09
MLVPuro	1.050	0.108	9.71	2.71e-22

Here are the results for expression density. First, we count just genes that are in the upper half.

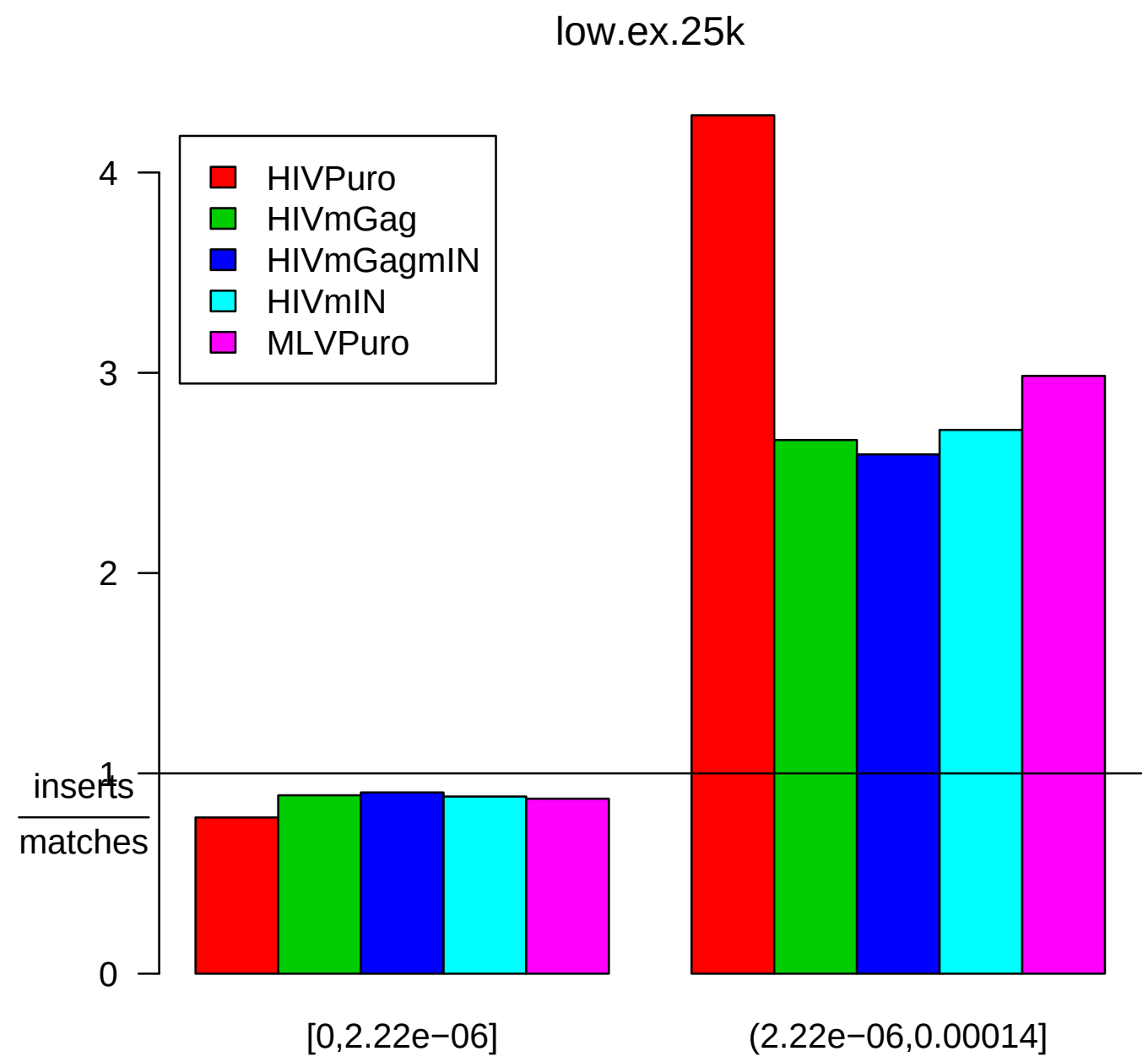

	coef	se	z	p
HIVPuro	1.74	0.117	14.90	6.44e-50
HIVmGag	1.11	0.136	8.11	4.93e-16
HIVmGagmIN	1.06	0.138	7.69	1.52e-14
HIVmIN	1.13	0.159	7.09	1.33e-12
MLVPuro	1.21	0.125	9.68	3.75e-22

Now we count genes in the upper $1/8^{th}$:

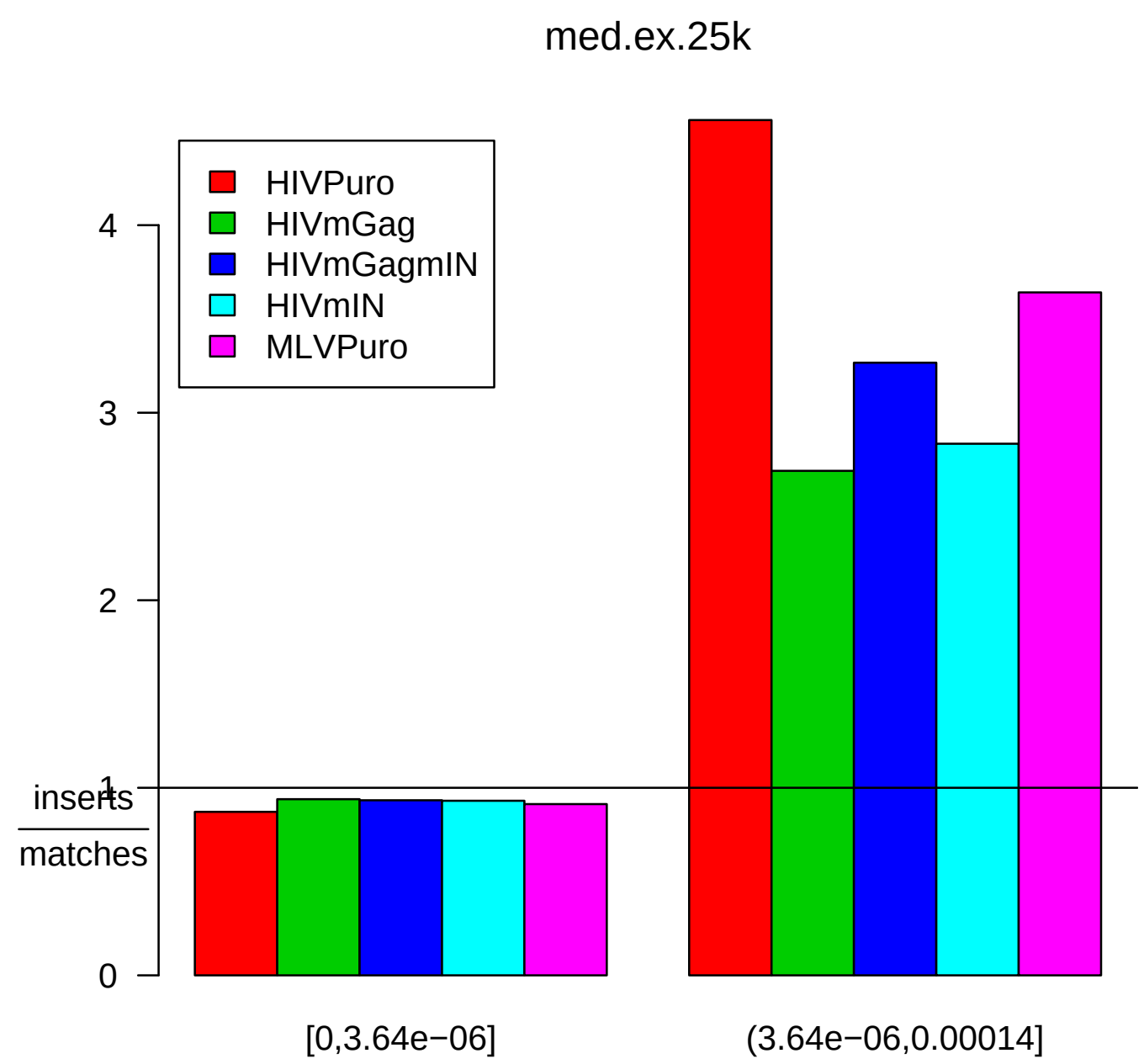

	coef	se	z	p
HIVPuro	1.66	0.142	11.60	2.43e-31
HIVmGag	1.06	0.174	6.08	1.22e-09
HIVmGagmIN	1.28	0.174	7.32	2.50e-13
HIVmIN	1.11	0.198	5.62	1.94e-08
MLVPuro	1.36	0.154	8.86	8.03e-19

And here we count genes in the upper $1/16^{th}$:

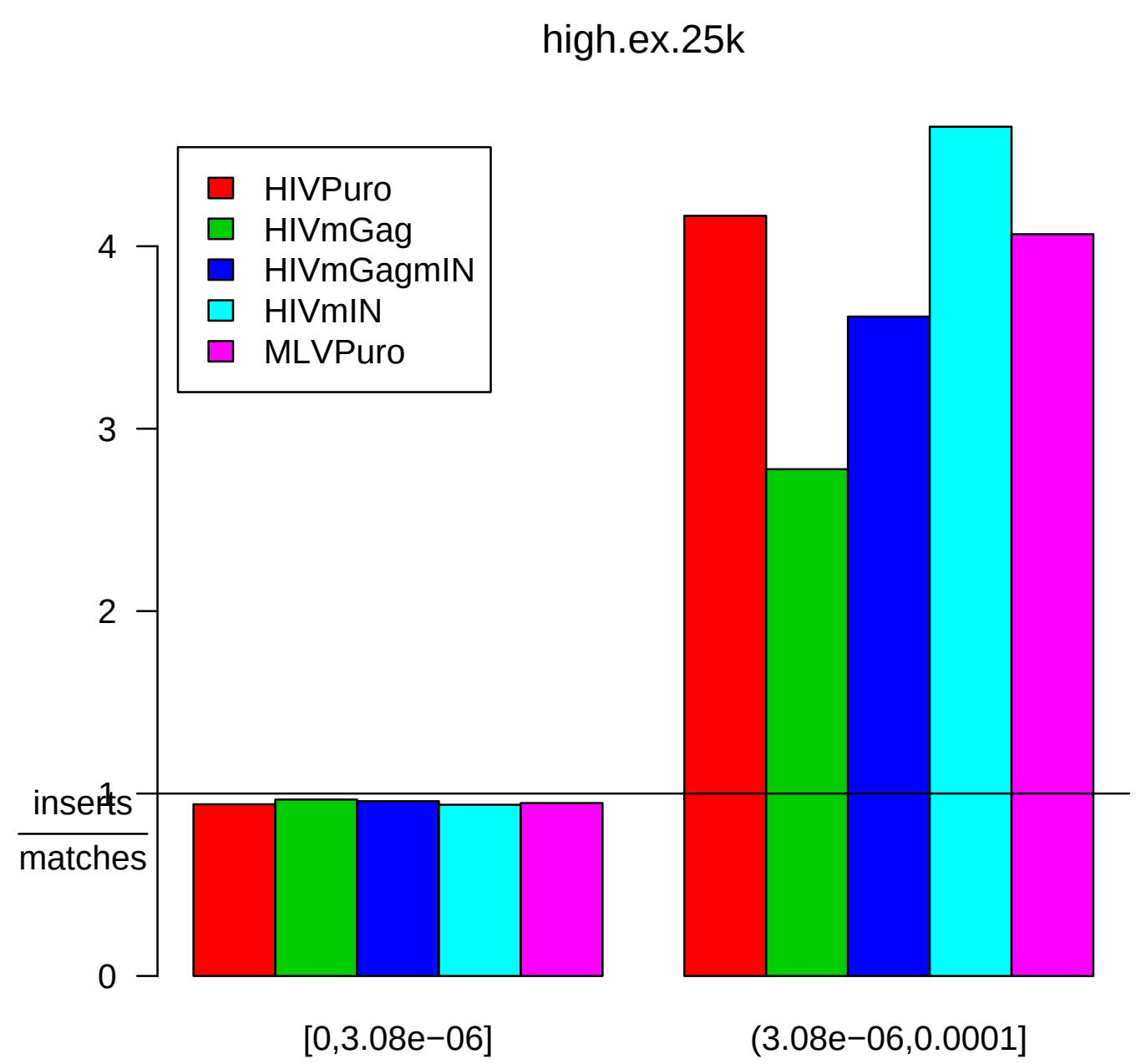

	coef	se	z	p
HIVPuro	1.49	0.195	7.64	2.15e-14
HIVmGag	1.07	0.234	4.59	4.48e-06
HIVmGagmIN	1.32	0.218	6.07	1.30e-09
HIVmIN	1.56	0.238	6.57	4.92e-11
MLVPuro	1.43	0.198	7.22	5.15e-13

Here the effect of density of CpG islands is studied:

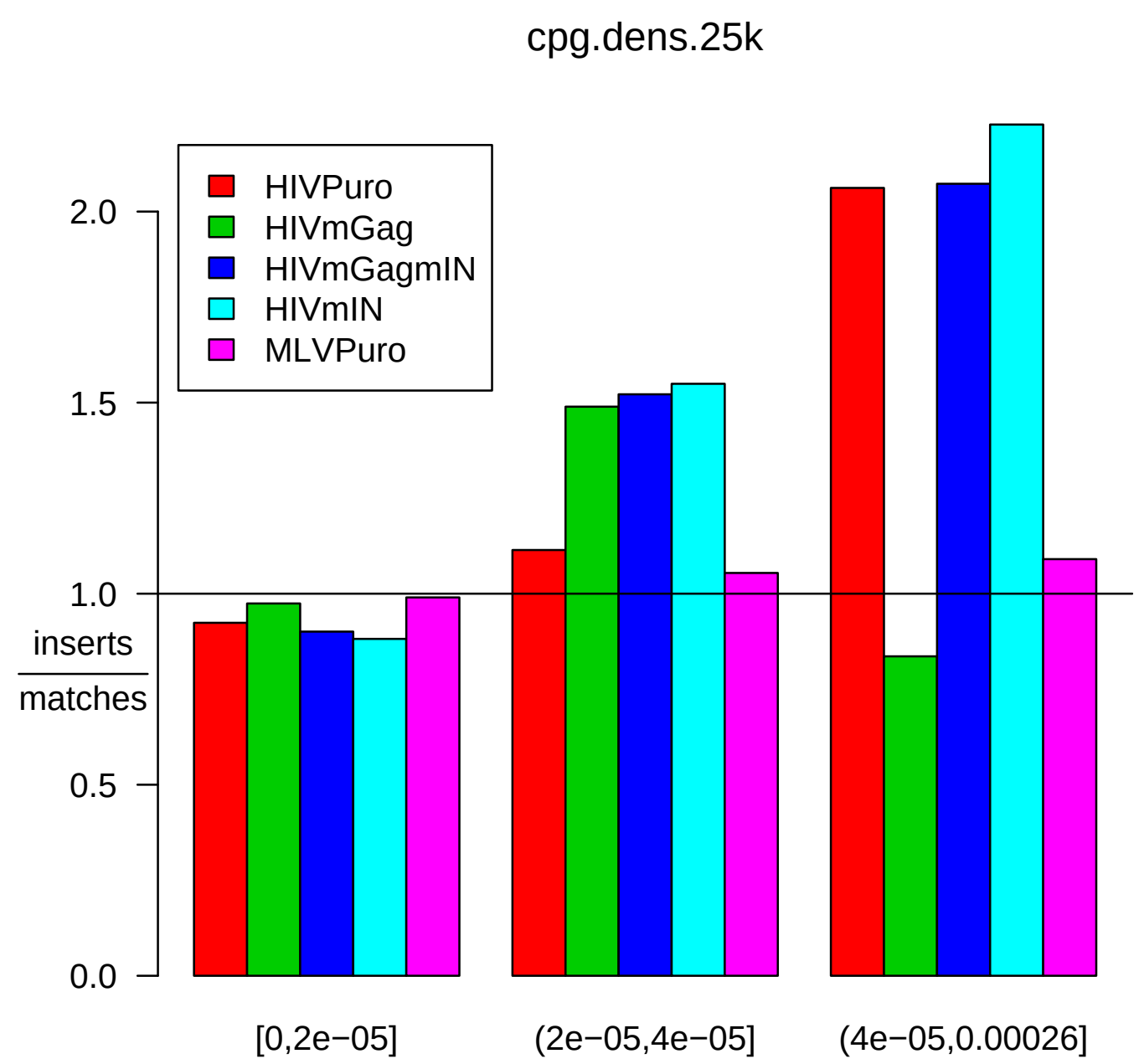

	coef	se	z	p
HIVPuro	0.534	0.0927	5.76	8.30e-09
HIVmGag	0.341	0.0986	3.46	5.37e-04
HIVmGagmIN	0.588	0.0926	6.35	2.13e-10
HIVmIN	0.722	0.1140	6.32	2.70e-10
MLVPuro	0.252	0.0950	2.65	7.98e-03

4.2 50 kilobase Window

In the barplot that follows we examine the association of insertion sites with expression density in a 50 kilobase window surrounding each locus. First, we count just the number of genes represented on the chip.

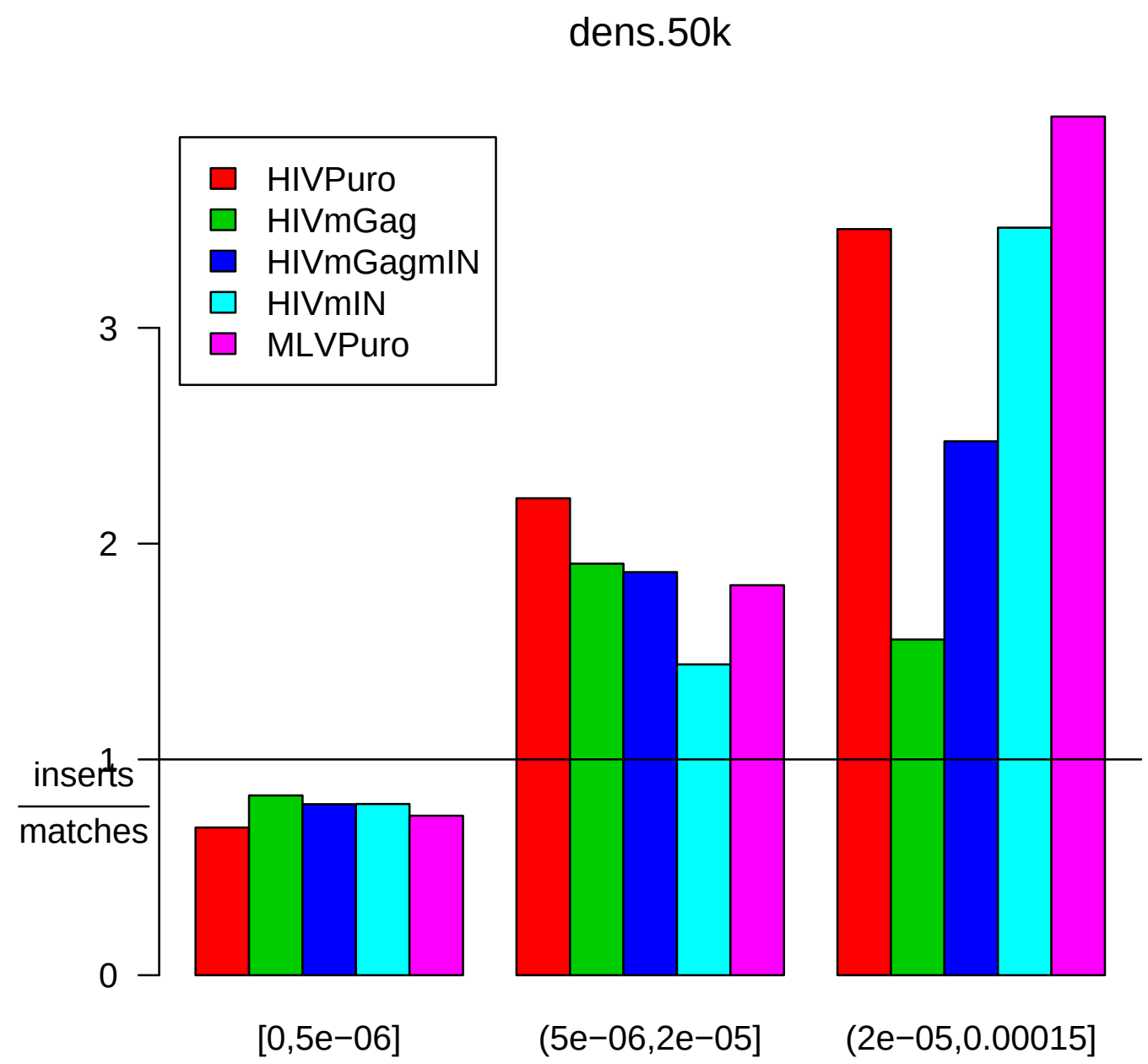

	coef	se	z	p
HIVPuro	1.450	0.0953	15.20	2.47e-52
HIVmGag	0.894	0.1010	8.85	8.63e-19
HIVmGagmIN	0.928	0.0960	9.67	4.01e-22
HIVmIN	0.891	0.1180	7.54	4.79e-14
MLVPuro	1.130	0.0921	12.30	8.86e-35

Here are the results for expression density. First, we count just genes that are in the upper half.

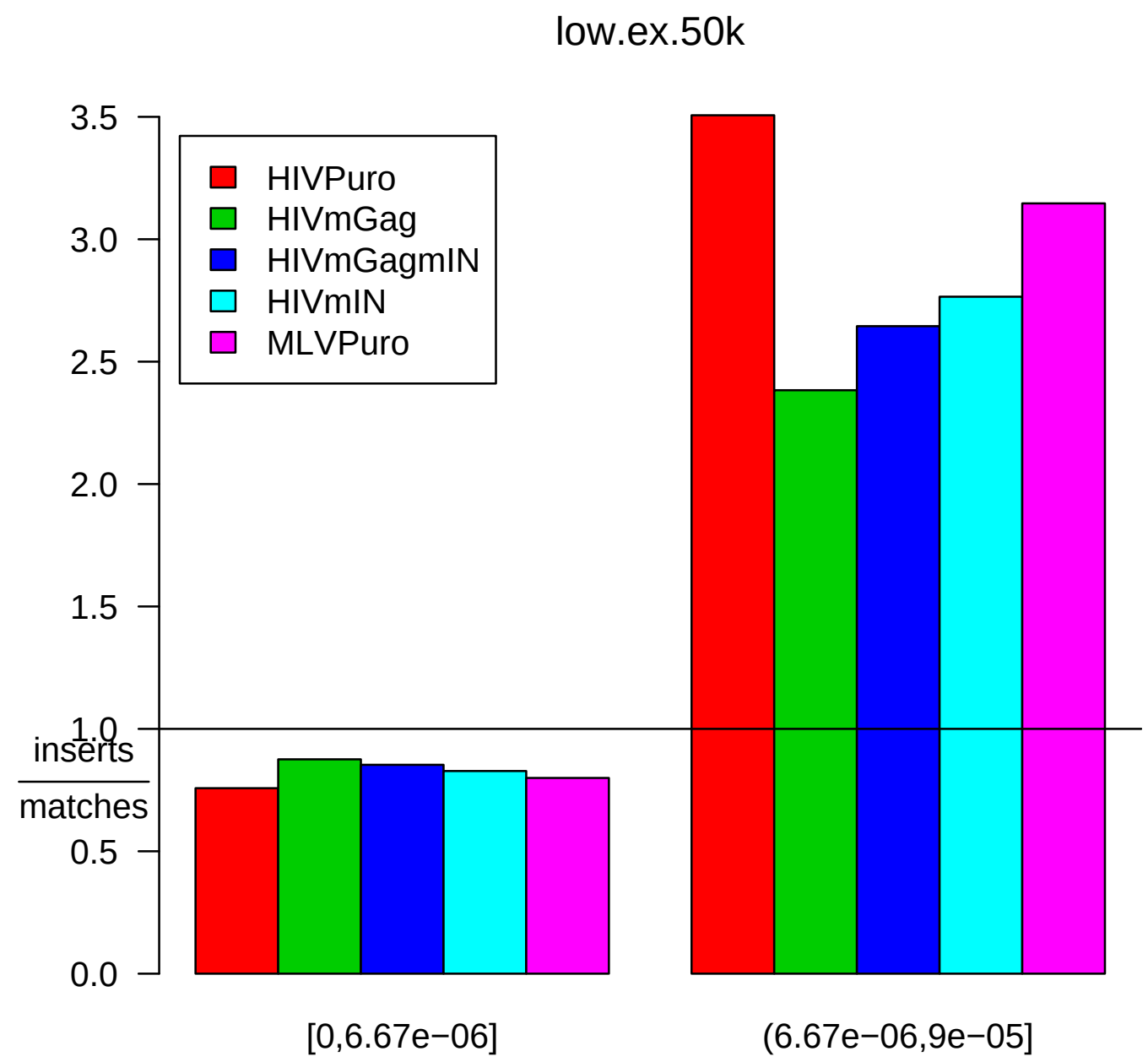

	coef	se	z	p
HIVPuro	1.73	0.102	17.10	2.84e-65
HIVmGag	1.14	0.111	10.30	1.09e-24
HIVmGagmIN	1.09	0.108	10.10	5.32e-24
HIVmIN	1.17	0.131	8.91	5.16e-19
MLVPuro	1.32	0.101	13.00	1.07e-38

Now we count genes in the upper $1/8^{th}$:

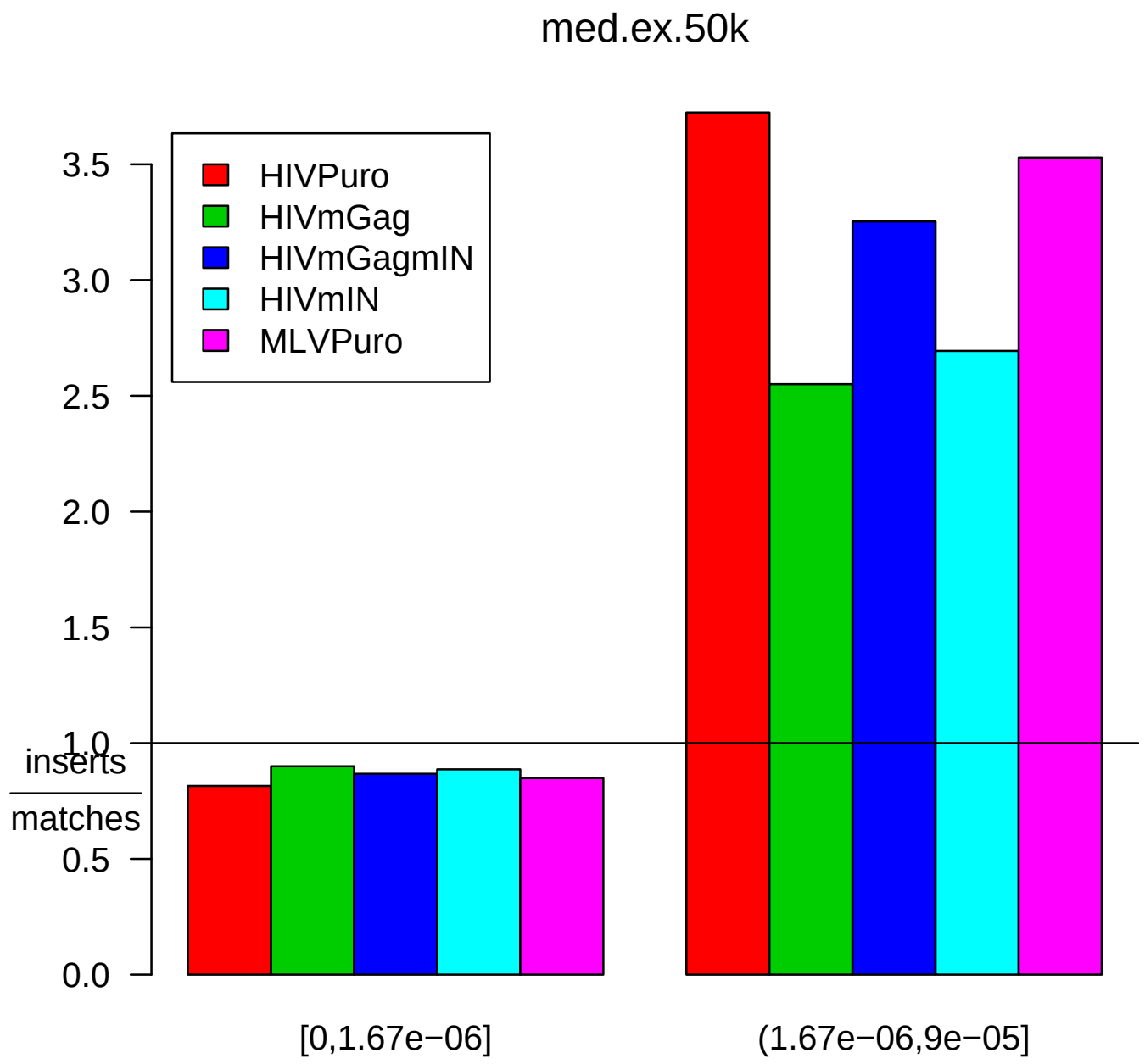

	coef	se	z	p
HIVPuro	1.51	0.118	12.80	1.08e-37
HIVmGag	1.05	0.139	7.52	5.33e-14
HIVmGagmIN	1.32	0.129	10.20	1.35e-24
HIVmIN	1.10	0.158	6.94	3.85e-12
MLVPuro	1.40	0.122	11.50	9.66e-31

And here we count genes in the upper $1/16^{th}$:

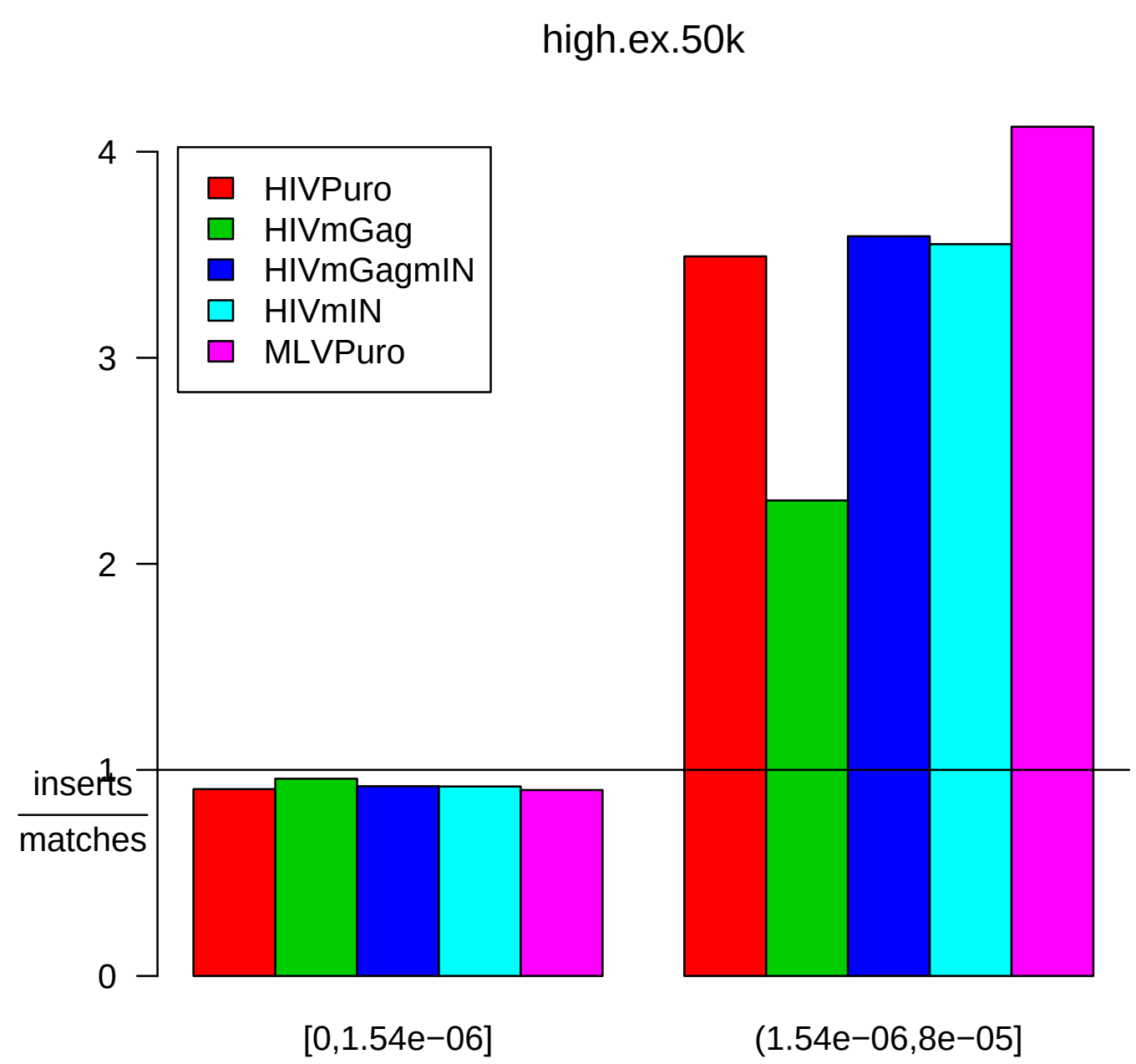

	coef	se	z	p
HIVPuro	1.35	0.152	8.89	6.22e-19
HIVmGag	0.89	0.193	4.62	3.84e-06
HIVmGagmIN	1.35	0.163	8.30	1.06e-16
HIVmIN	1.33	0.197	6.76	1.40e-11
MLVPuro	1.50	0.151	9.94	2.86e-23

Here the effect of density of CpG islands is studied:

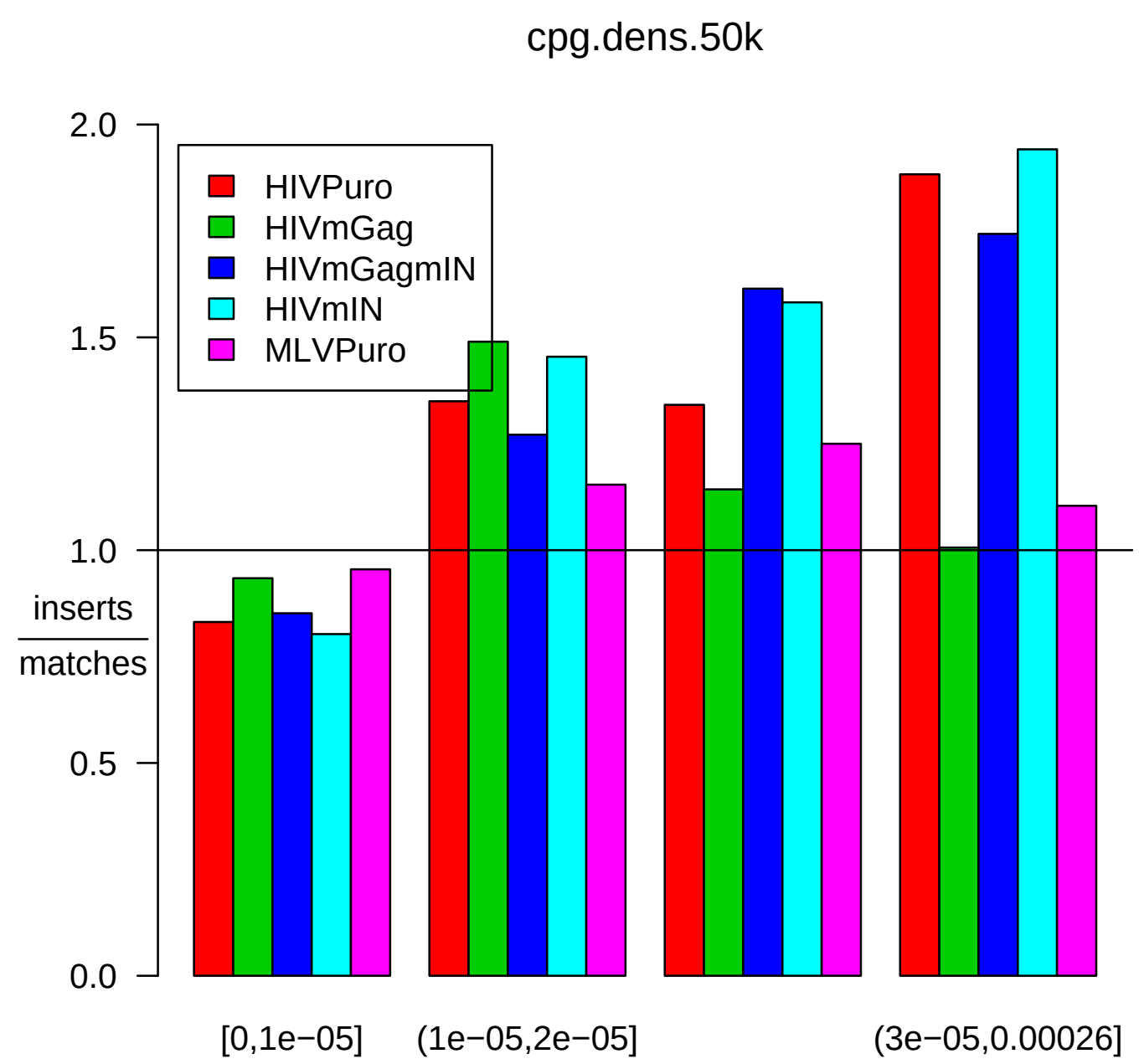

	coef	se	z	p
HIVPuro	0.548	0.0933	5.87	4.24e-09
HIVmGag	0.472	0.0952	4.96	6.93e-07
HIVmGagmIN	0.642	0.0932	6.89	5.64e-12
HIVmIN	0.693	0.1150	6.02	1.70e-09
MLVPuro	0.240	0.0901	2.66	7.77e-03

4.3 100 kilobase Window

In the barplot that follows we examine the association of insertion sites with expression density in a 100 kilobase window surrounding each locus. First, we count just the number of genes represented on the chip.

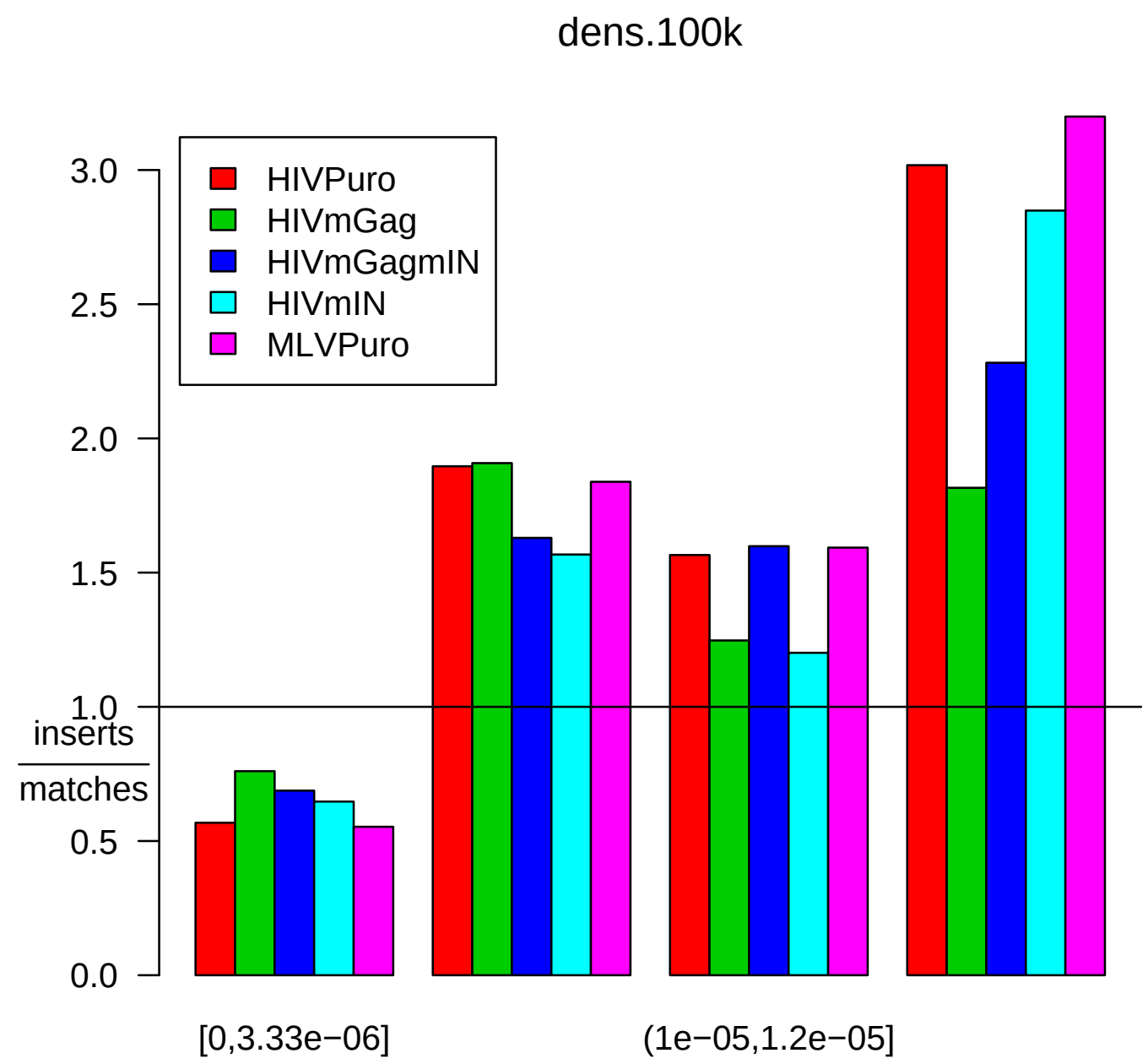

	coef	se	z	p
HIVPuro	1.50	0.0987	15.30	1.64e-52
HIVmGag	0.90	0.0955	9.42	4.38e-21
HIVmGagmIN	1.01	0.0928	10.80	2.41e-27
HIVmIN	1.12	0.1160	9.67	3.87e-22
MLVPuro	1.43	0.0950	15.00	4.60e-51

Here are the results for expression density. First, we count just genes that are in the upper half.

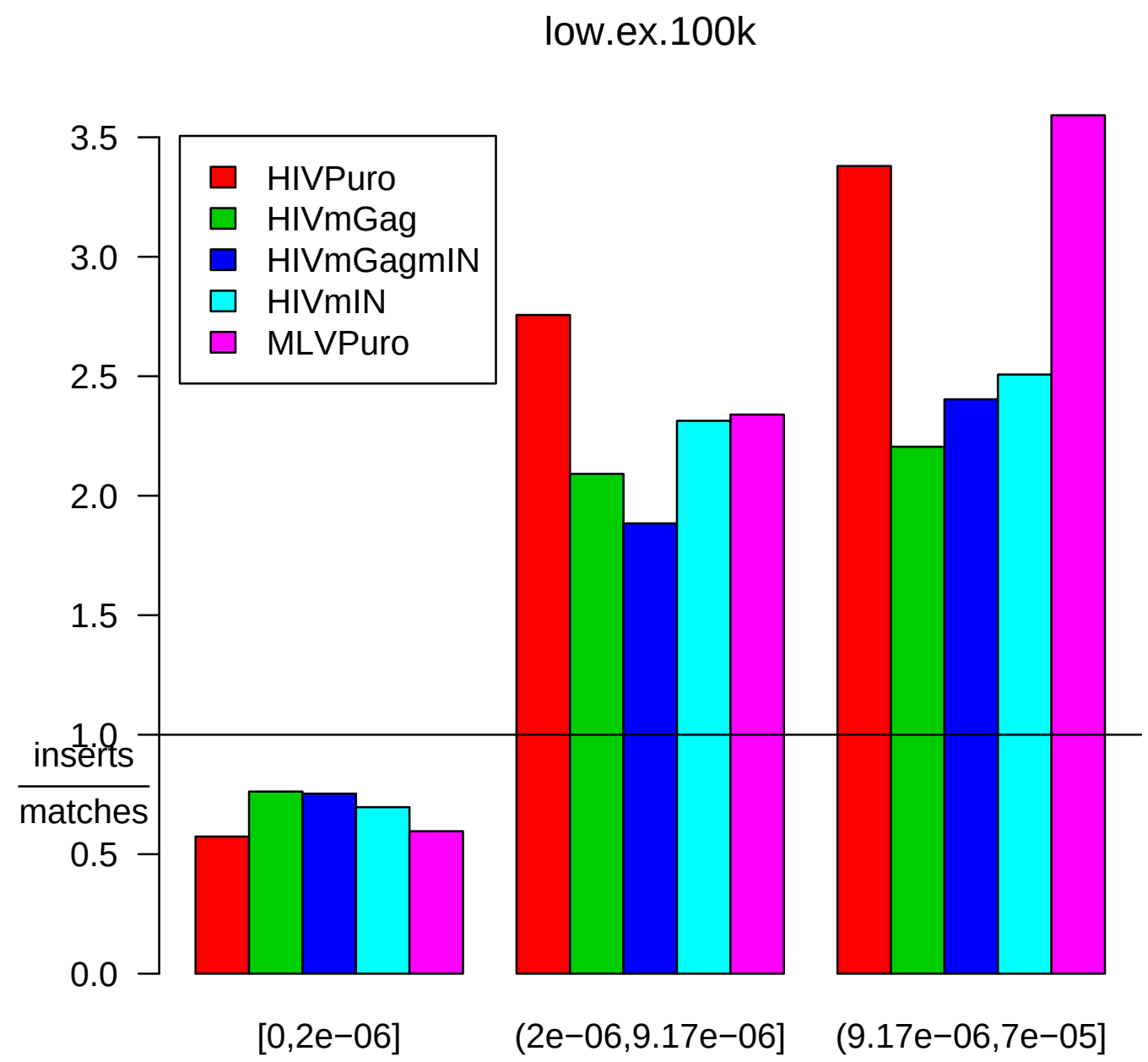

	coef	se	z	p
HIVPuro	1.75	0.0967	18.1	1.94e-73
HIVmGag	1.08	0.0987	10.9	1.16e-27
HIVmGagmIN	1.13	0.0957	11.8	3.10e-32
HIVmIN	1.30	0.1180	11.0	3.74e-28
MLVPuro	1.63	0.0944	17.2	1.91e-66

Now we count genes in the upper $1/8^{th}$:

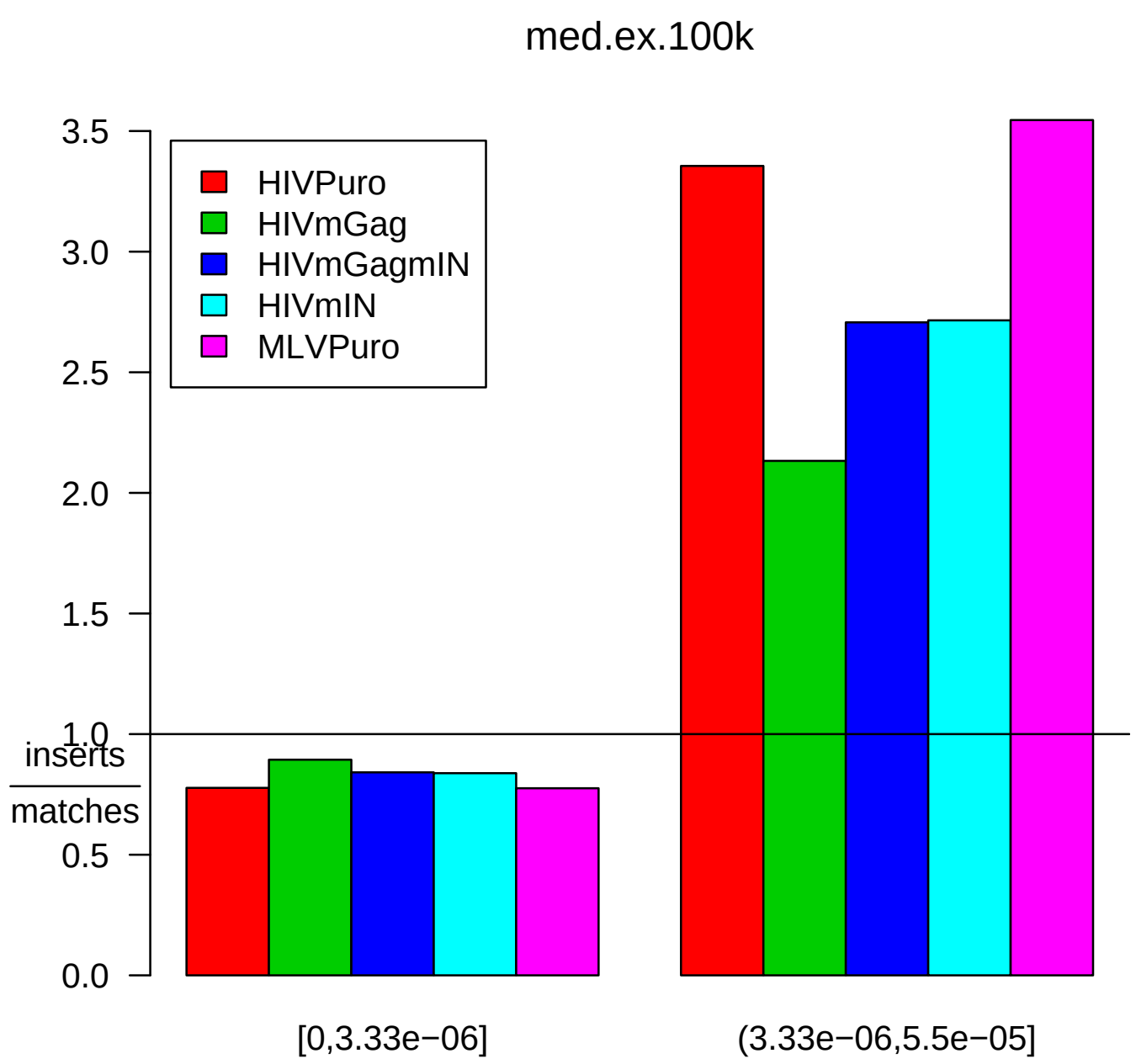

	coef	se	z	p
HIVPuro	1.490	0.102	14.60	1.51e-48
HIVmGag	0.885	0.116	7.66	1.82e-14
HIVmGagmIN	1.160	0.108	10.80	5.75e-27
HIVmIN	1.160	0.131	8.85	9.01e-19
MLVPuro	1.520	0.102	14.90	6.59e-50

And here we count genes in the upper $1/16^{th}$:

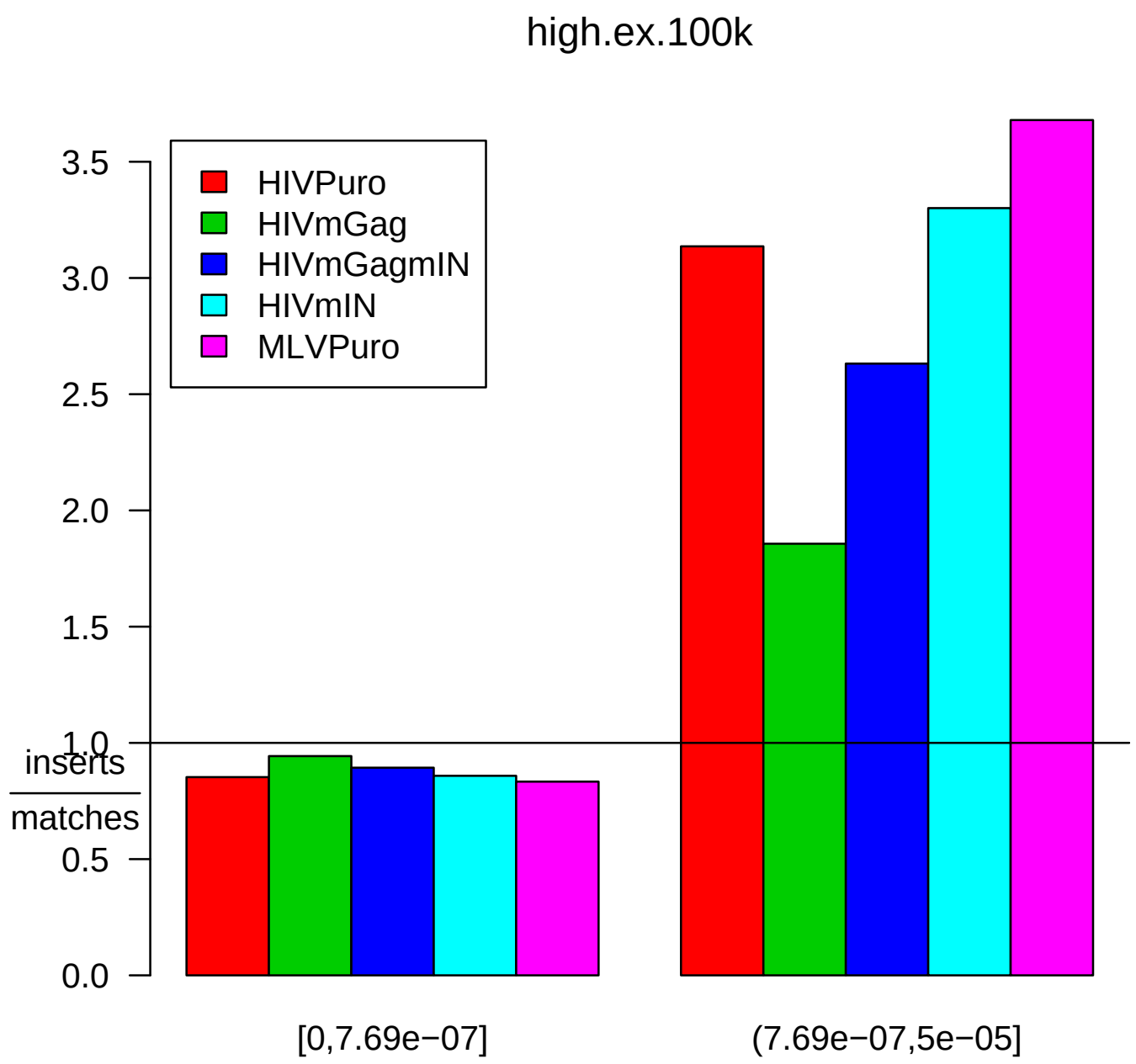


```
            coef    se     z        p
HIVPuro    1.310 0.124 10.60 3.62e-26
HIVmGag    0.679 0.153  4.44 8.97e-06
HIVmGagmIN 1.080 0.132  8.18 2.90e-16
HIVmIN     1.350 0.155  8.70 3.37e-18
MLVPuro    1.500 0.121 12.40 3.51e-35
```

Here the effect of density of CpG islands is studied:

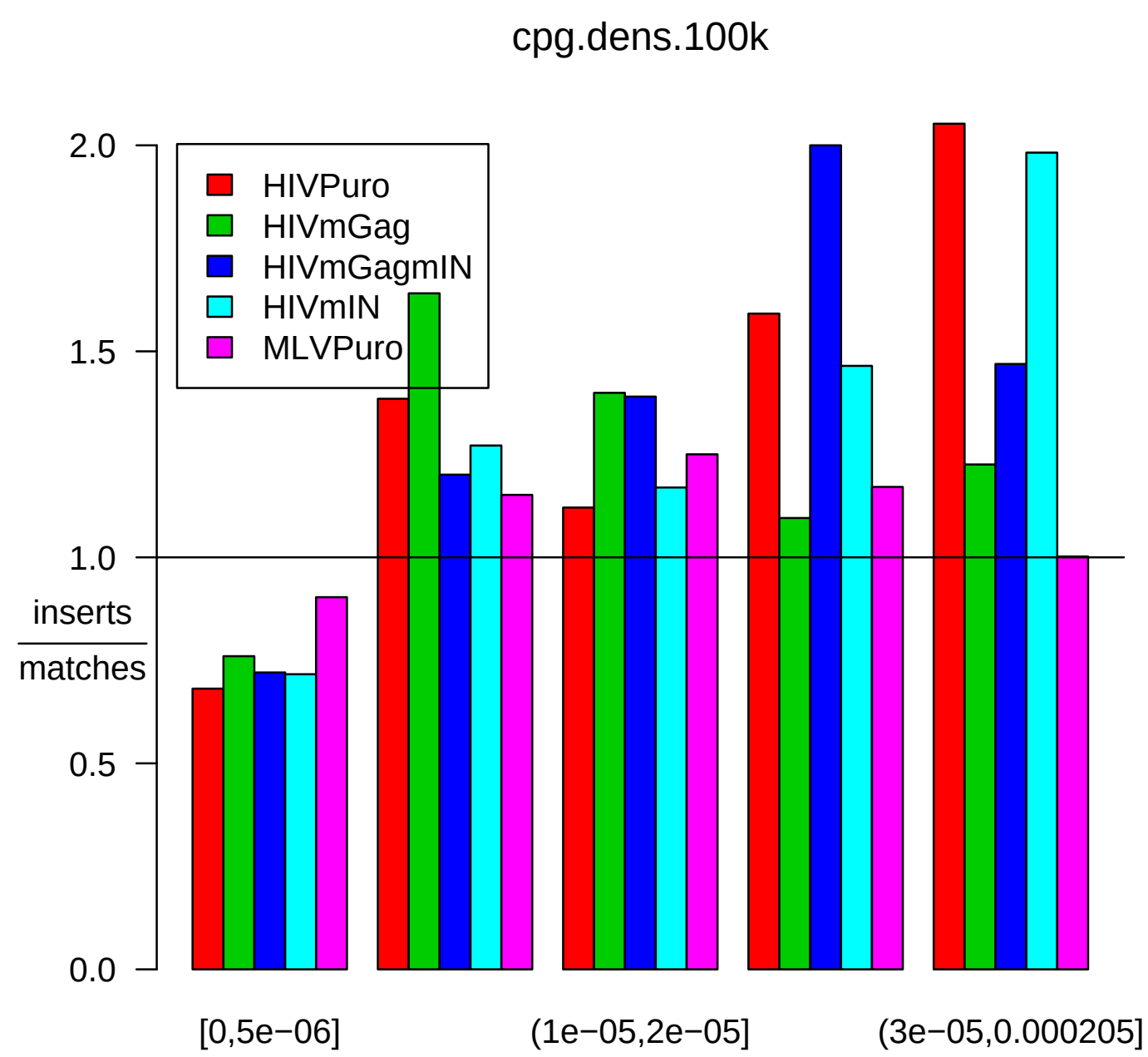

	coef	se	z	p
HIVPuro	0.770	0.0932	8.27	1.37e-16
HIVmGag	0.602	0.0953	6.31	2.79e-10
HIVmGagmIN	0.688	0.0924	7.45	9.62e-14
HIVmIN	0.700	0.1130	6.18	6.41e-10
MLVPuro	0.241	0.0906	2.66	7.84e-03

4.4 250 kilobase Window

In the barplot that follows we examine the association of insertion sites with expression density in a 250 kilobase window surrounding each locus. First, we count just the number of genes represented on the chip.

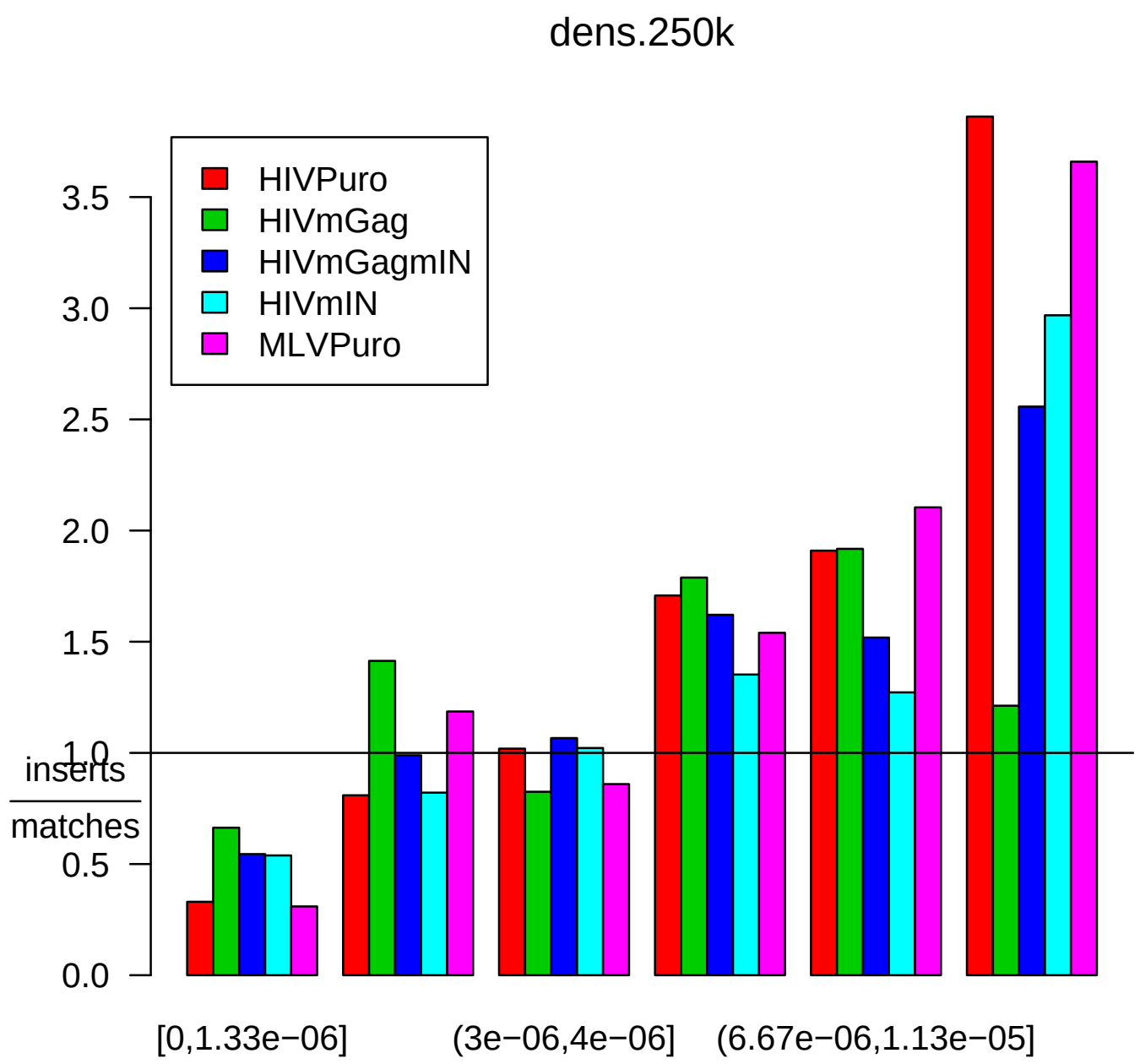

	coef	se	z	p
HIVPuro	1.690	0.1180	14.30	3.29e-46
HIVmGag	0.727	0.0985	7.38	1.53e-13
HIVmGagmIN	1.010	0.1010	10.00	1.45e-23
HIVmIN	1.020	0.1250	8.16	3.35e-16
MLVPuro	1.740	0.1190	14.60	2.10e-48

Here are the results for expression density. First, we count just genes that are in the upper half.

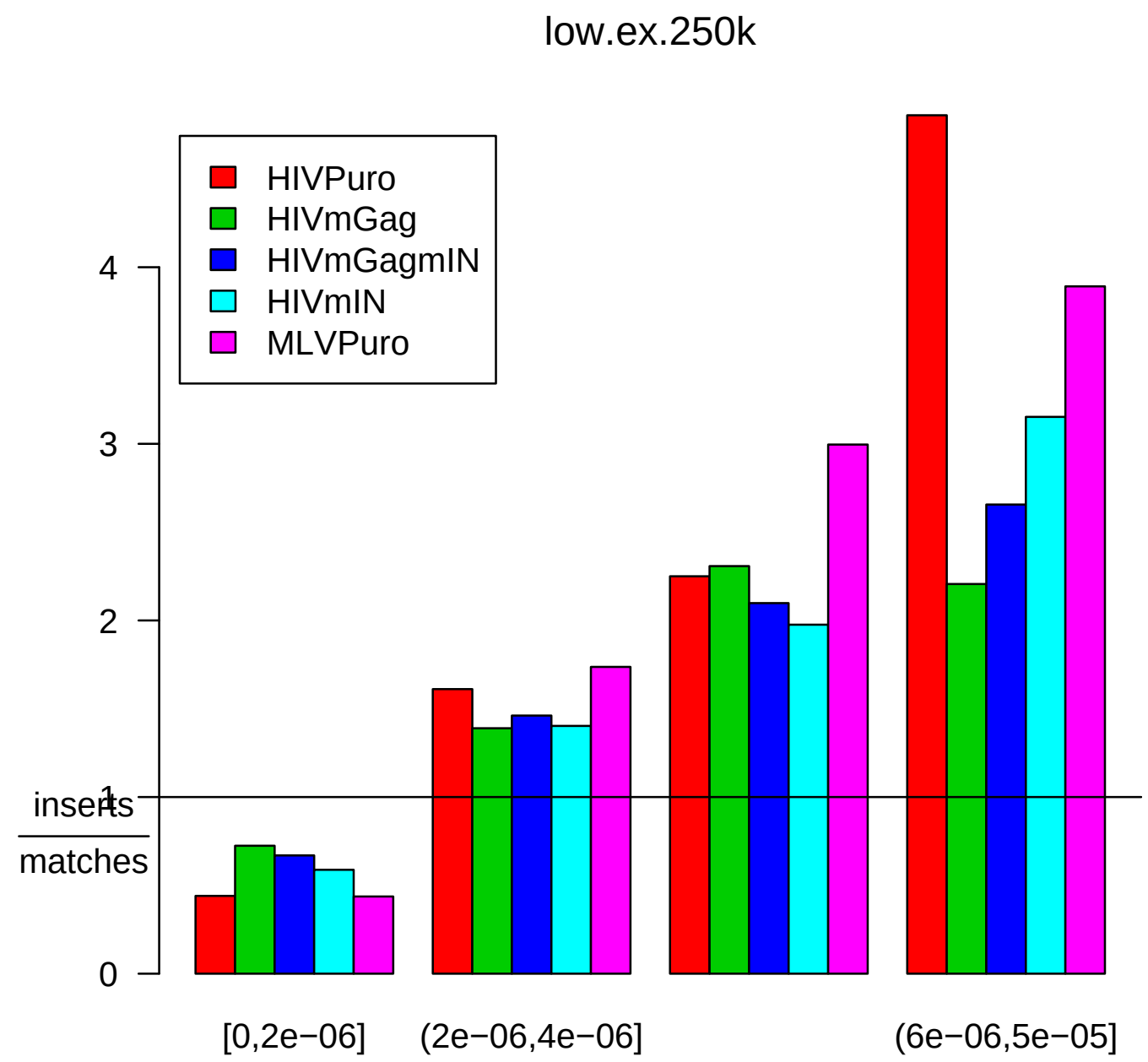

	coef	se	z	p
HIVPuro	1.90	0.1090	17.5	1.55e-68
HIVmGag	1.01	0.0961	10.5	5.26e-26
HIVmGagmIN	1.25	0.0963	13.0	9.70e-39
HIVmIN	1.30	0.1210	10.7	8.59e-27
MLVPuro	1.92	0.1080	17.7	2.34e-70

Now we count genes in the upper $1/8^{th}$:

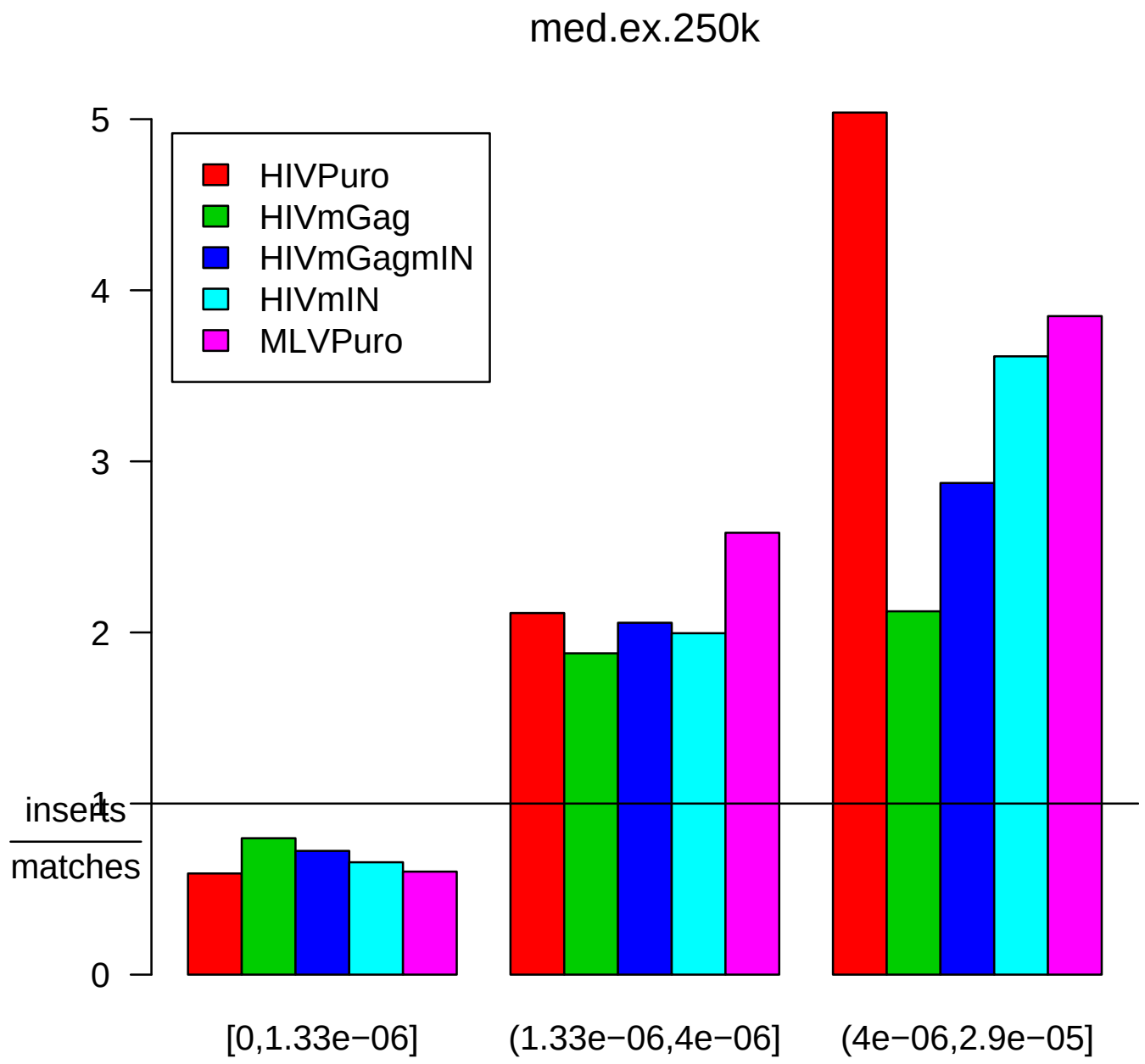

	coef	se	z	p
HIVPuro	1.710	0.0976	17.50	2.09e-68
HIVmGag	0.868	0.0980	8.87	7.59e-19
HIVmGagmIN	1.230	0.0944	13.10	4.95e-39
HIVmIN	1.300	0.1150	11.30	1.27e-29
MLVPuro	1.700	0.0939	18.10	6.02e-73

And here we count genes in the upper $1/16^{th}$:

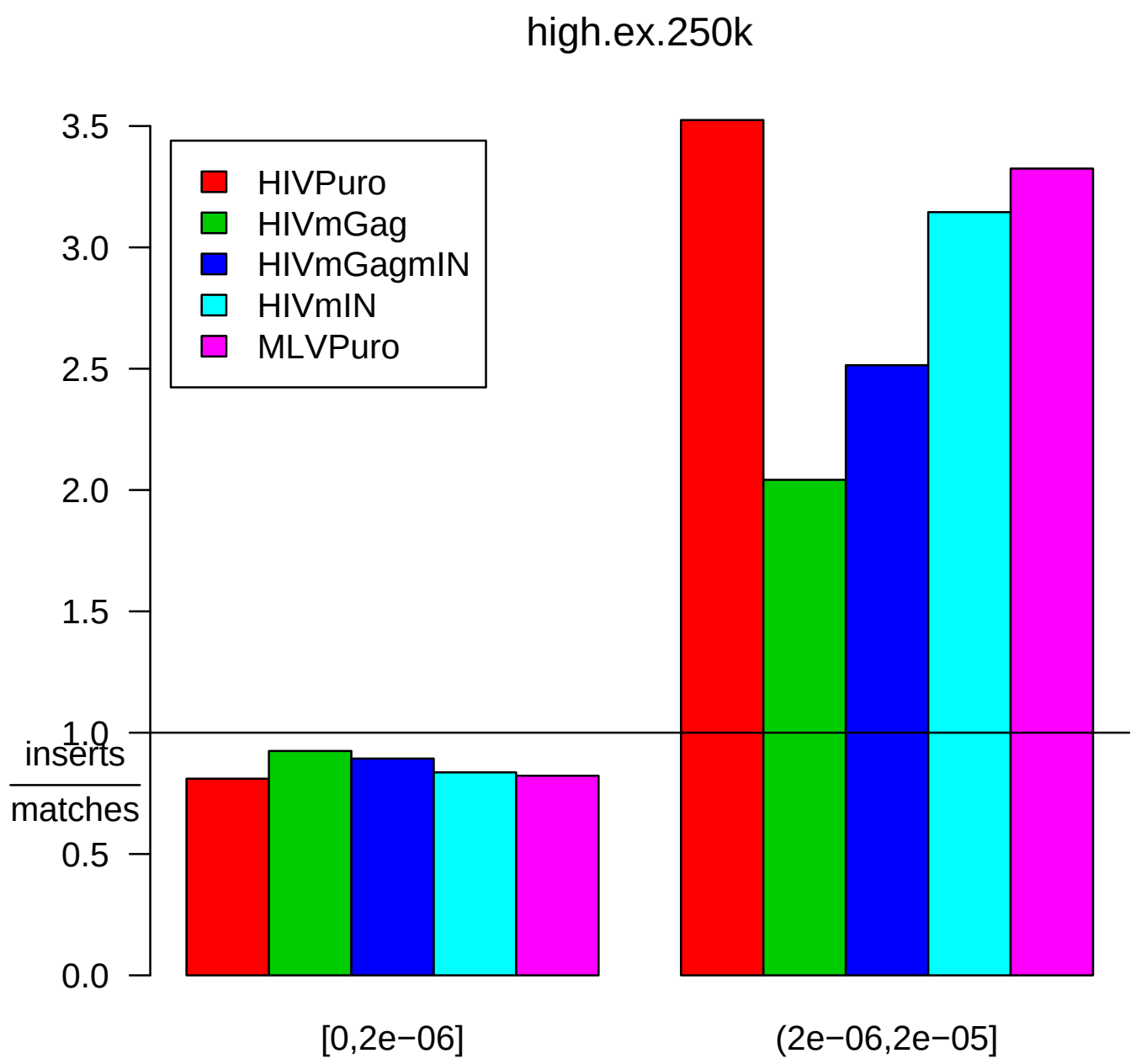

	coef	se	z	p
HIVPuro	1.420	0.0998	14.30	4.05e-46
HIVmGag	0.696	0.1130	6.16	7.09e-10
HIVmGagmIN	1.130	0.1020	11.00	2.38e-28
HIVmIN	1.340	0.1220	11.00	4.94e-28
MLVPuro	1.540	0.0985	15.60	8.56e-55

Here the effect of density of CpG islands is studied:

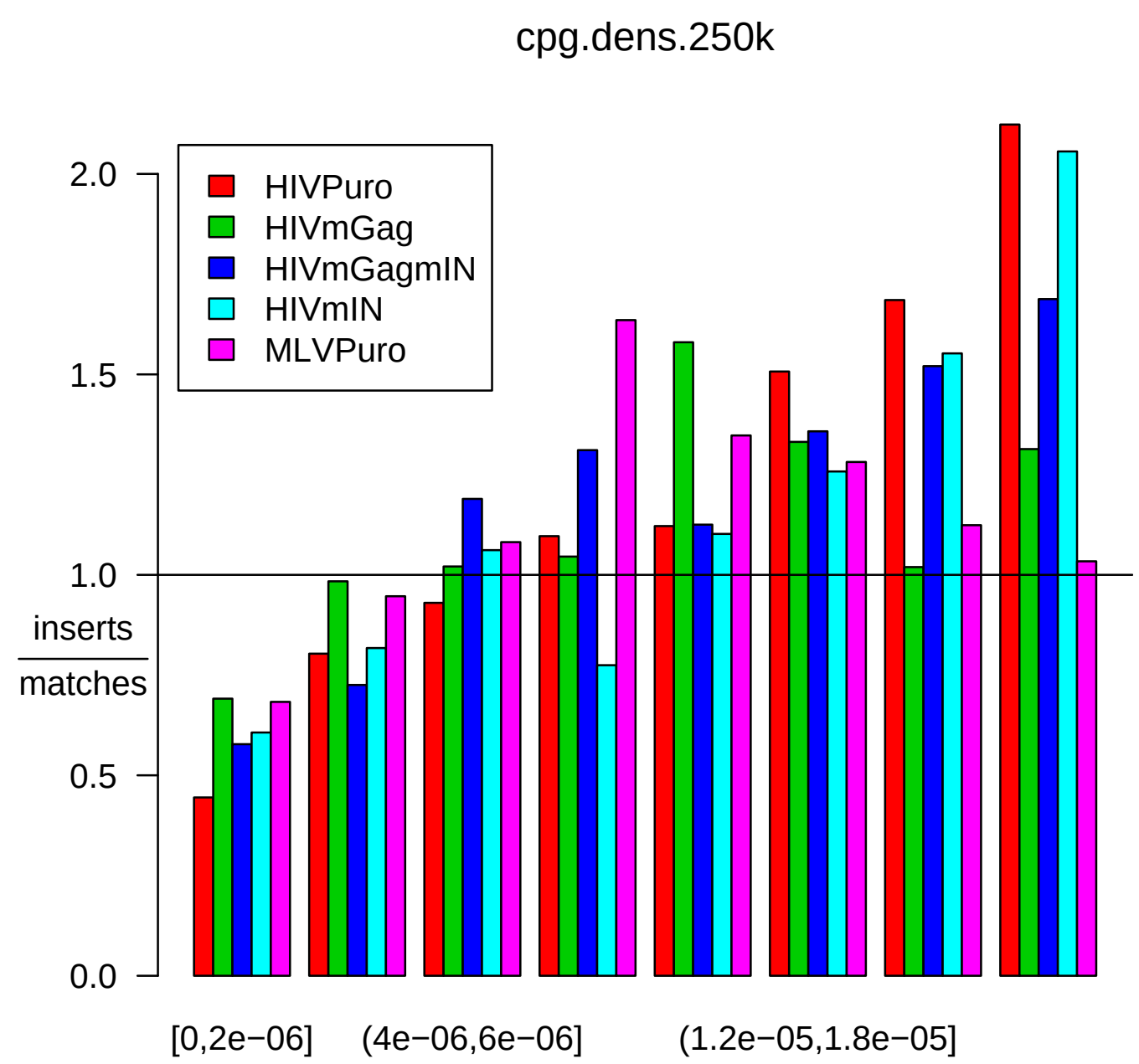

	coef	se	z	p
HIVPuro	0.926	0.0968	9.57	1.08e-21
HIVmGag	0.457	0.0947	4.82	1.42e-06
HIVmGagmIN	0.683	0.0933	7.33	2.35e-13
HIVmIN	0.641	0.1150	5.56	2.76e-08
MLVPuro	0.473	0.0910	5.19	2.07e-07

4.5 500 kilobase Window

In the barplot that follows we examine the association of insertion sites with expression density in a 500 kilobase window surrounding each locus. First, we count just the number of genes represented on the chip.

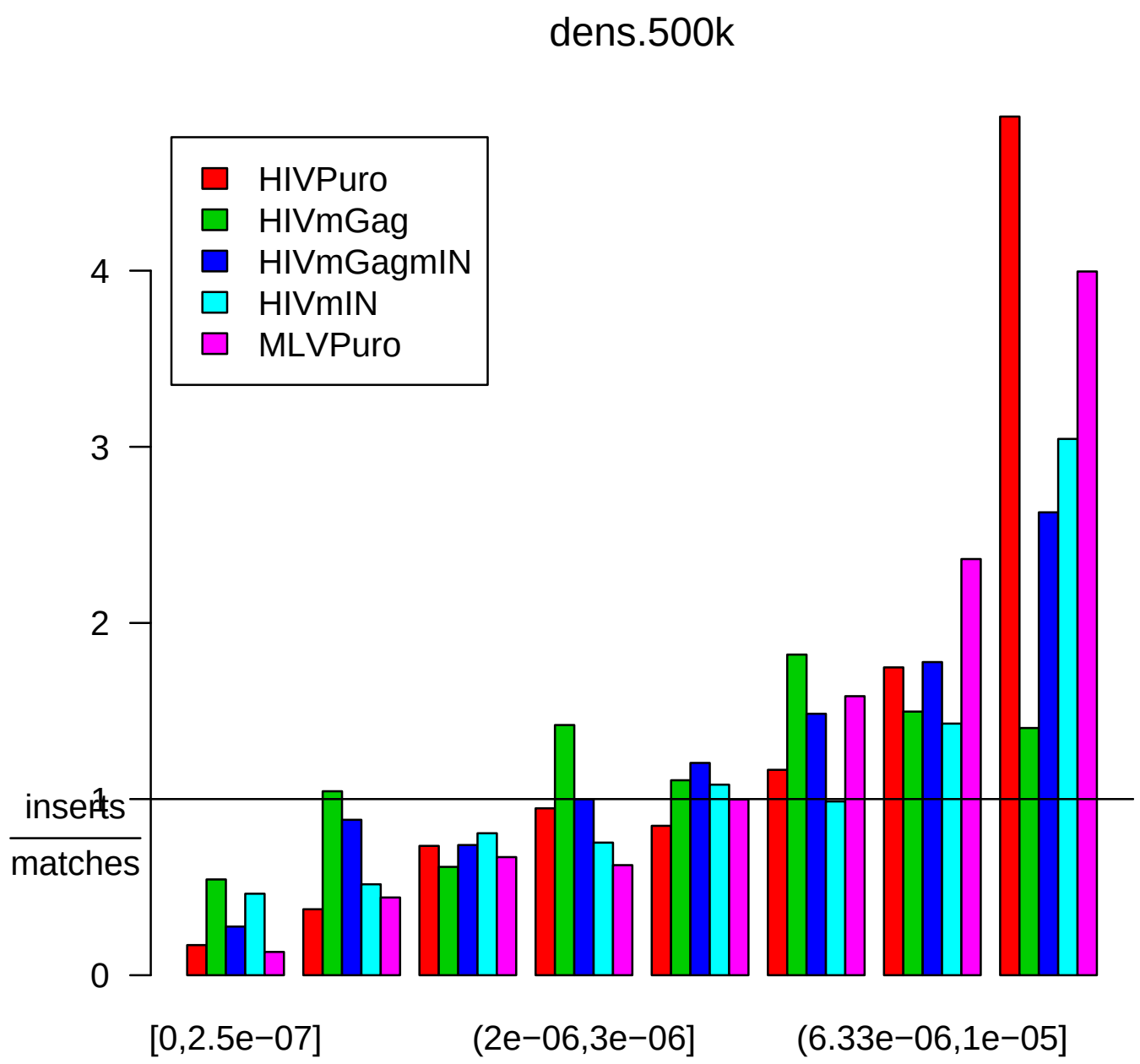

	coef	se	z	p
HIVPuro	1.690	0.1160	14.60	4.15e-48
HIVmGag	0.789	0.0981	8.04	8.85e-16
HIVmGagmIN	1.160	0.1020	11.40	3.74e-30
HIVmIN	1.020	0.1250	8.19	2.61e-16
MLVPuro	1.810	0.1180	15.40	1.02e-53

Here are the results for expression density. First, we count just genes that are in the upper half.

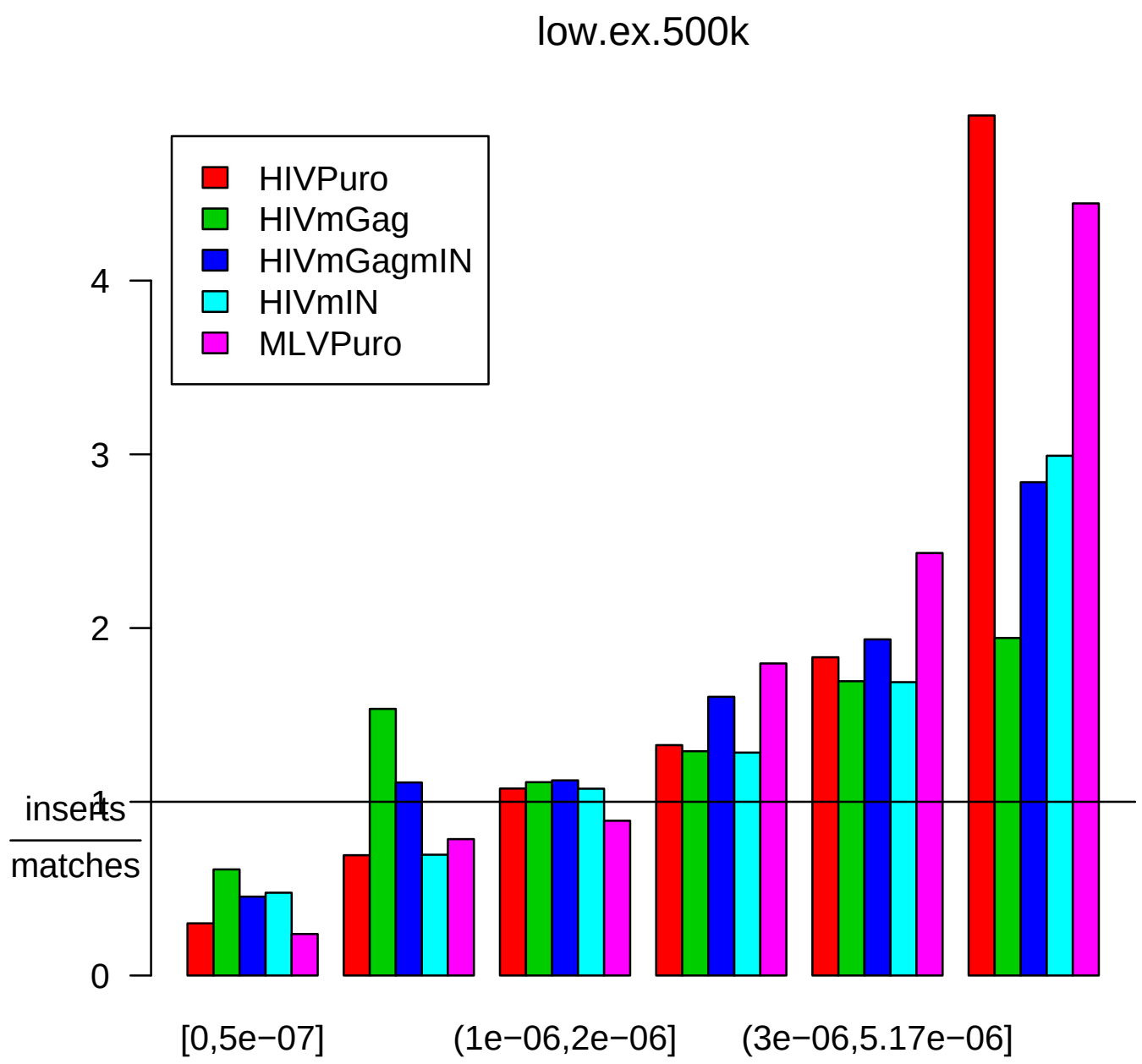

	coef	se	z	p
HIVPuro	1.830	0.1220	14.90	1.78e-50
HIVmGag	0.882	0.0995	8.86	8.10e-19
HIVmGagmIN	1.290	0.1050	12.30	8.91e-35
HIVmIN	1.220	0.1310	9.30	1.37e-20
MLVPuro	2.110	0.1300	16.20	4.85e-59

Now we count genes in the upper $1/8^{th}$:

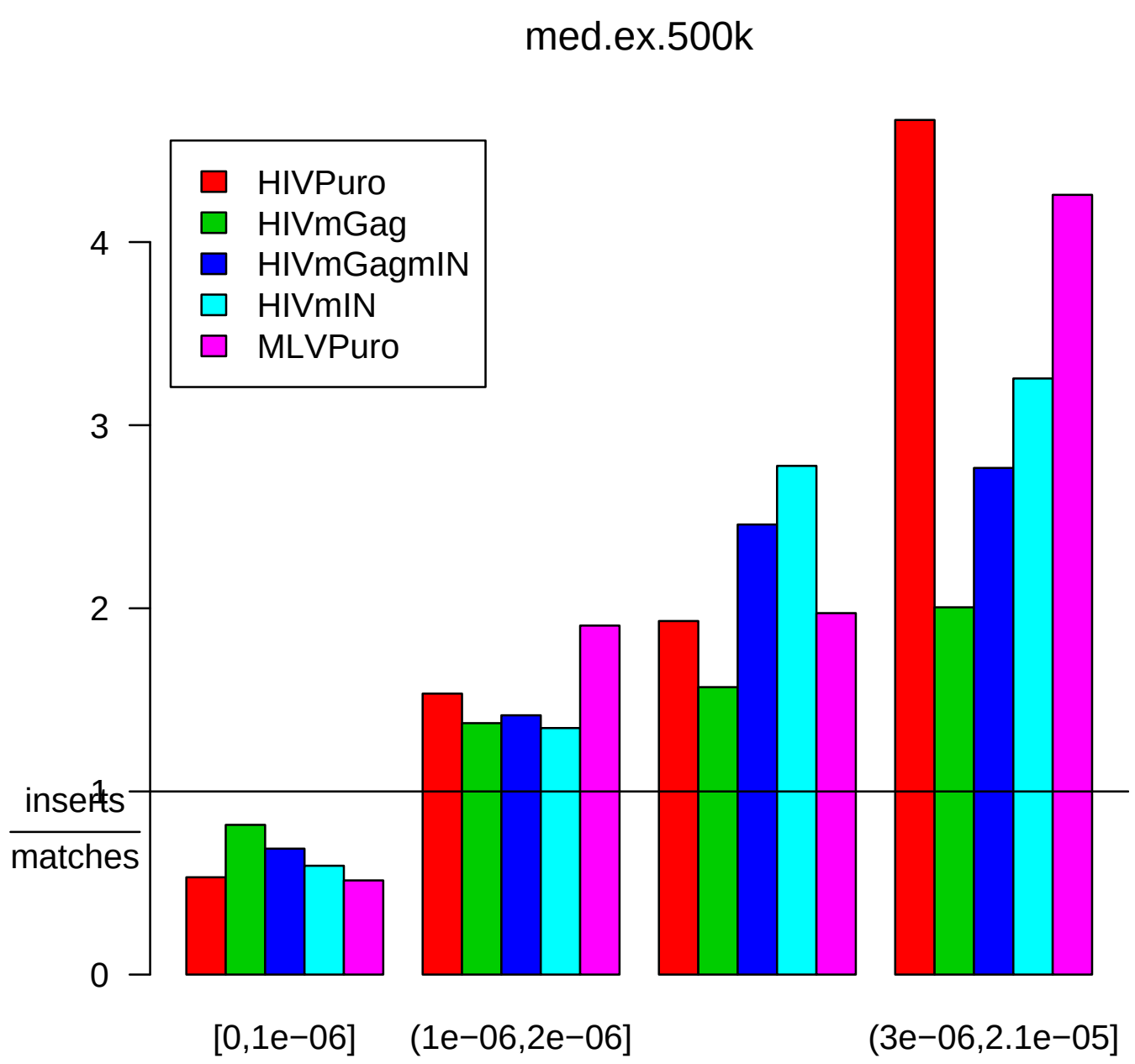

	coef	se	z	p
HIVPuro	1.580	0.1020	15.40	7.89e-54
HIVmGag	0.857	0.0952	9.01	2.10e-19
HIVmGagmIN	1.330	0.0975	13.60	3.43e-42
HIVmIN	1.160	0.1190	9.75	1.84e-22
MLVPuro	1.920	0.1080	17.80	1.27e-70

And here we count genes in the upper $1/16^{th}$:

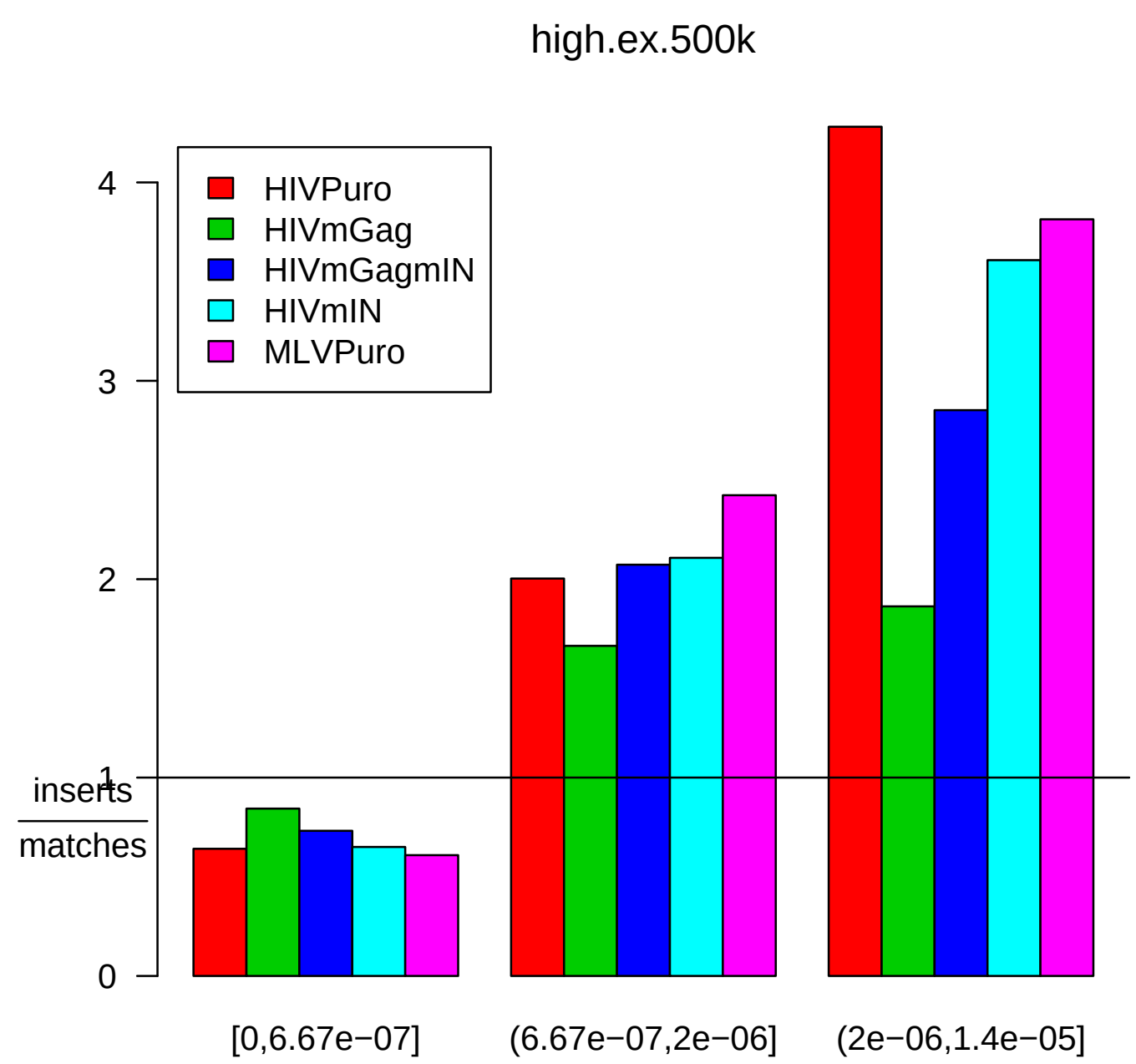

	coef	se	z	p
HIVPuro	1.390	0.0937	14.80	1.36e-49
HIVmGag	0.681	0.0992	6.86	6.70e-12
HIVmGagmIN	1.200	0.0939	12.80	3.03e-37
HIVmIN	1.230	0.1140	10.80	3.56e-27
MLVPuro	1.600	0.0941	17.00	4.87e-65

Here the effect of density of CpG islands is studied:

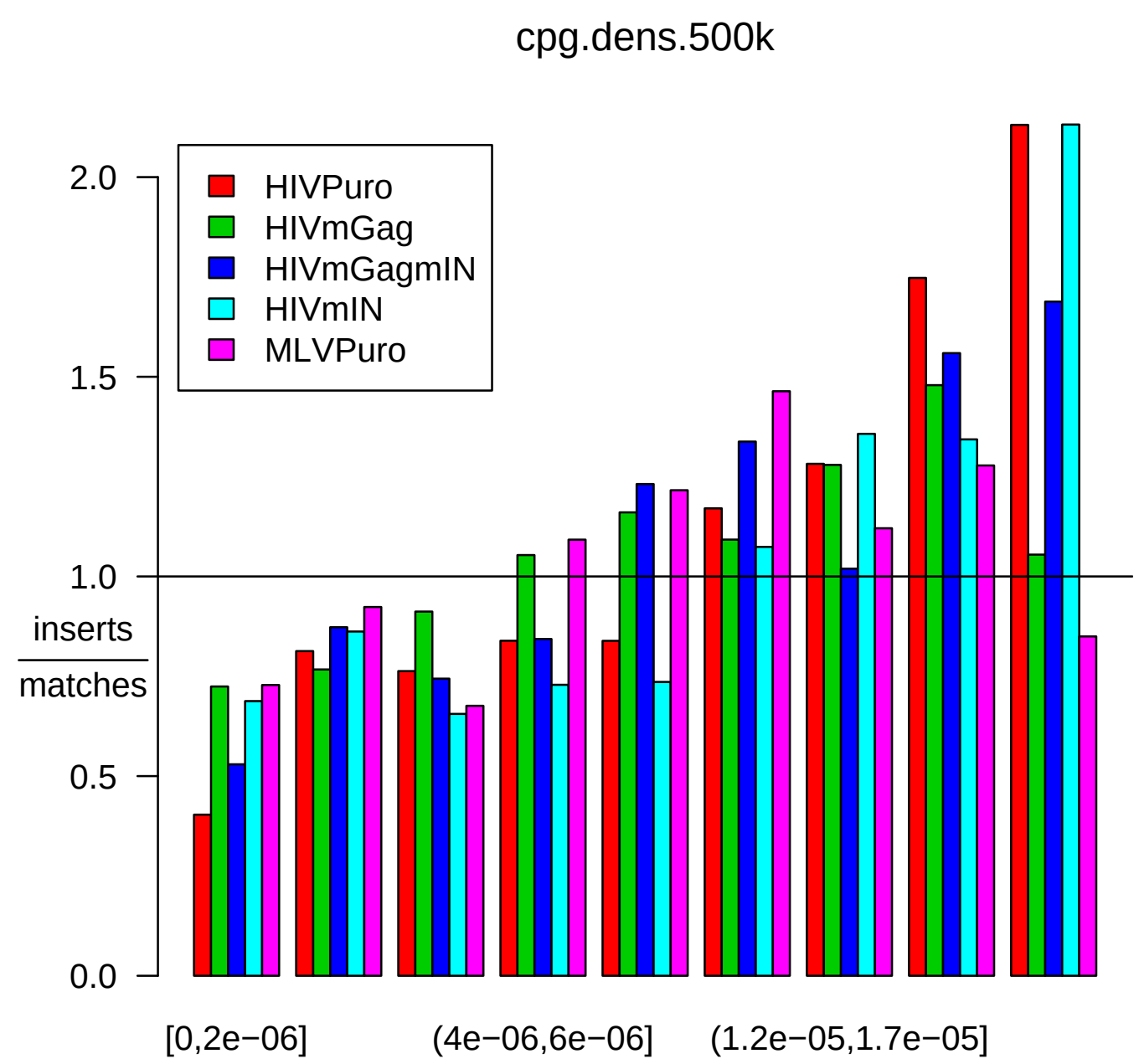

	coef	se	z	p
HIVPuro	0.860	0.0982	8.75	2.10e-18
HIVmGag	0.380	0.0952	3.99	6.63e-05
HIVmGagmIN	0.704	0.0949	7.43	1.12e-13
HIVmIN	0.619	0.1160	5.34	9.34e-08
MLVPuro	0.372	0.0908	4.10	4.15e-05

4.6 1 megabase Window

In the barplot that follows we examine the association of insertion sites with expression density in a 1 megabase window surrounding each locus. First, we count just the number of genes represented on the chip.

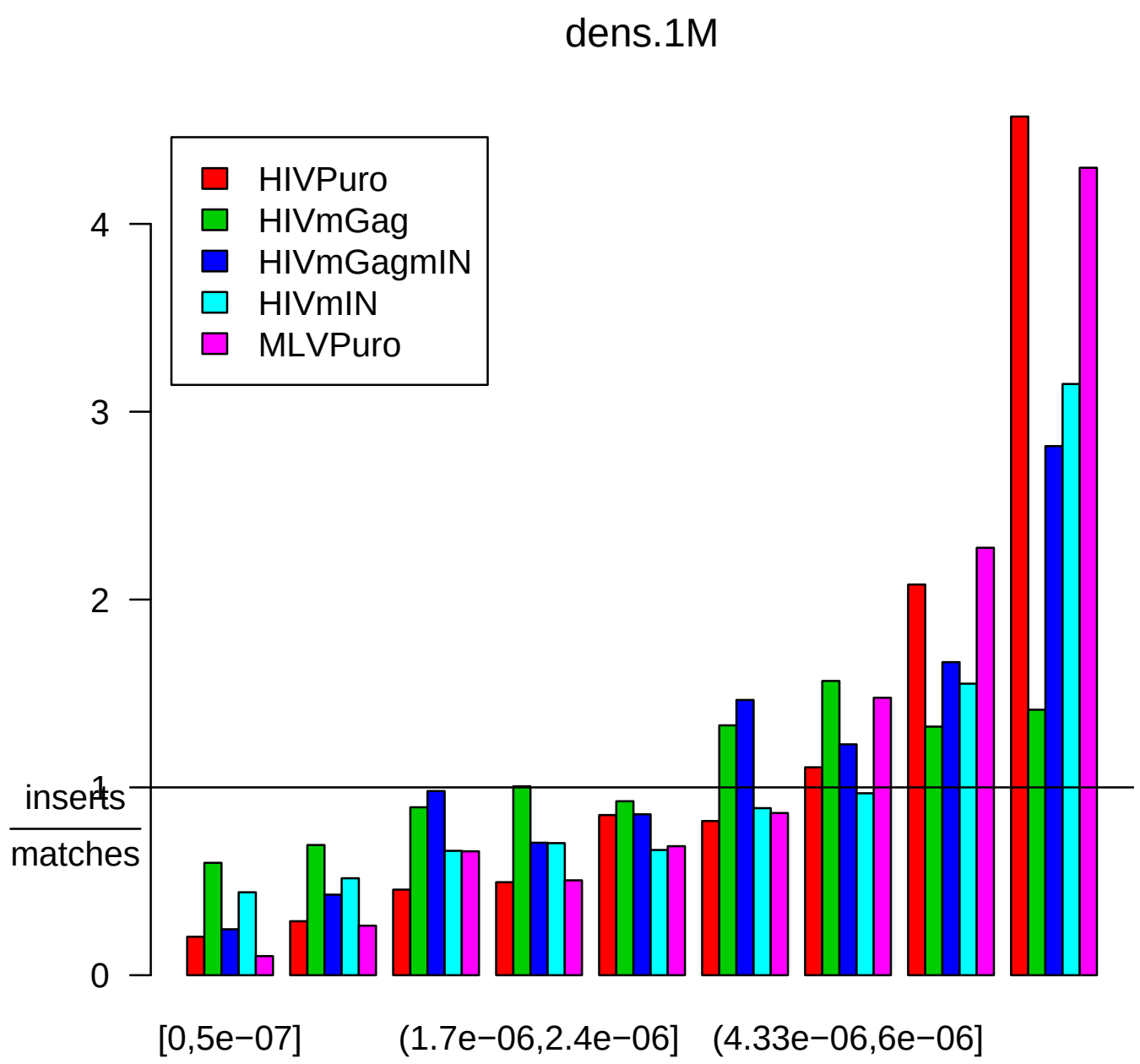

	coef	se	z	p
HIVPuro	1.710	0.1200	14.20	6.05e-46
HIVmGag	0.559	0.0966	5.78	7.36e-09
HIVmGagmIN	1.150	0.1040	11.00	2.73e-28
HIVmIN	0.988	0.1270	7.77	8.13e-15
MLVPuro	1.810	0.1210	15.00	1.01e-50

Here are the results for expression density. First, we count just genes that are in the upper half.

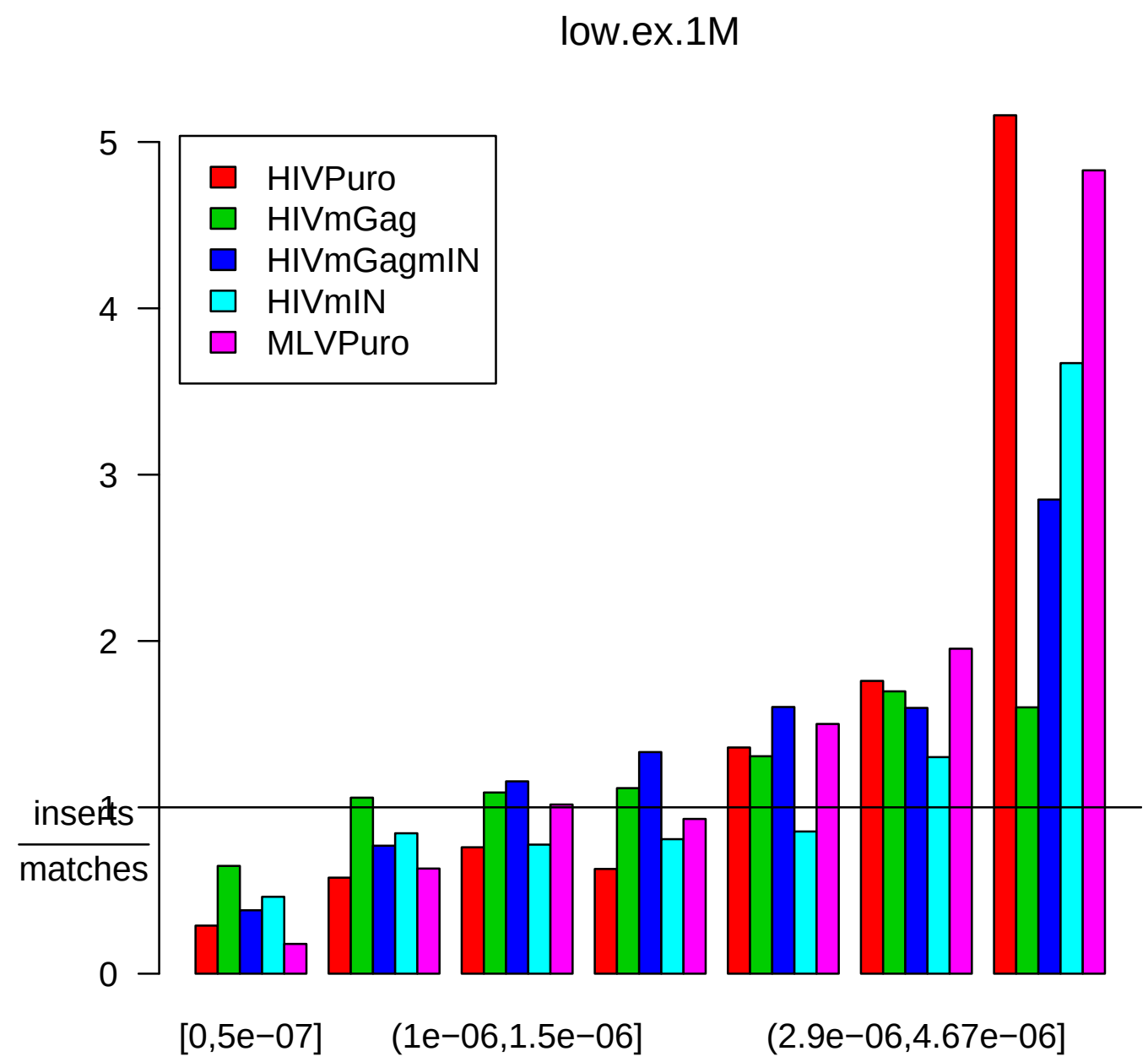

	coef	se	z	p
HIVPuro	1.660	0.1130	14.80	2.89e-49
HIVmGag	0.624	0.0959	6.51	7.50e-11
HIVmGagmIN	1.310	0.1040	12.60	1.81e-36
HIVmIN	1.050	0.1250	8.45	2.83e-17
MLVPuro	2.000	0.1230	16.30	1.52e-59

Now we count genes in the upper $1/8^{th}$:

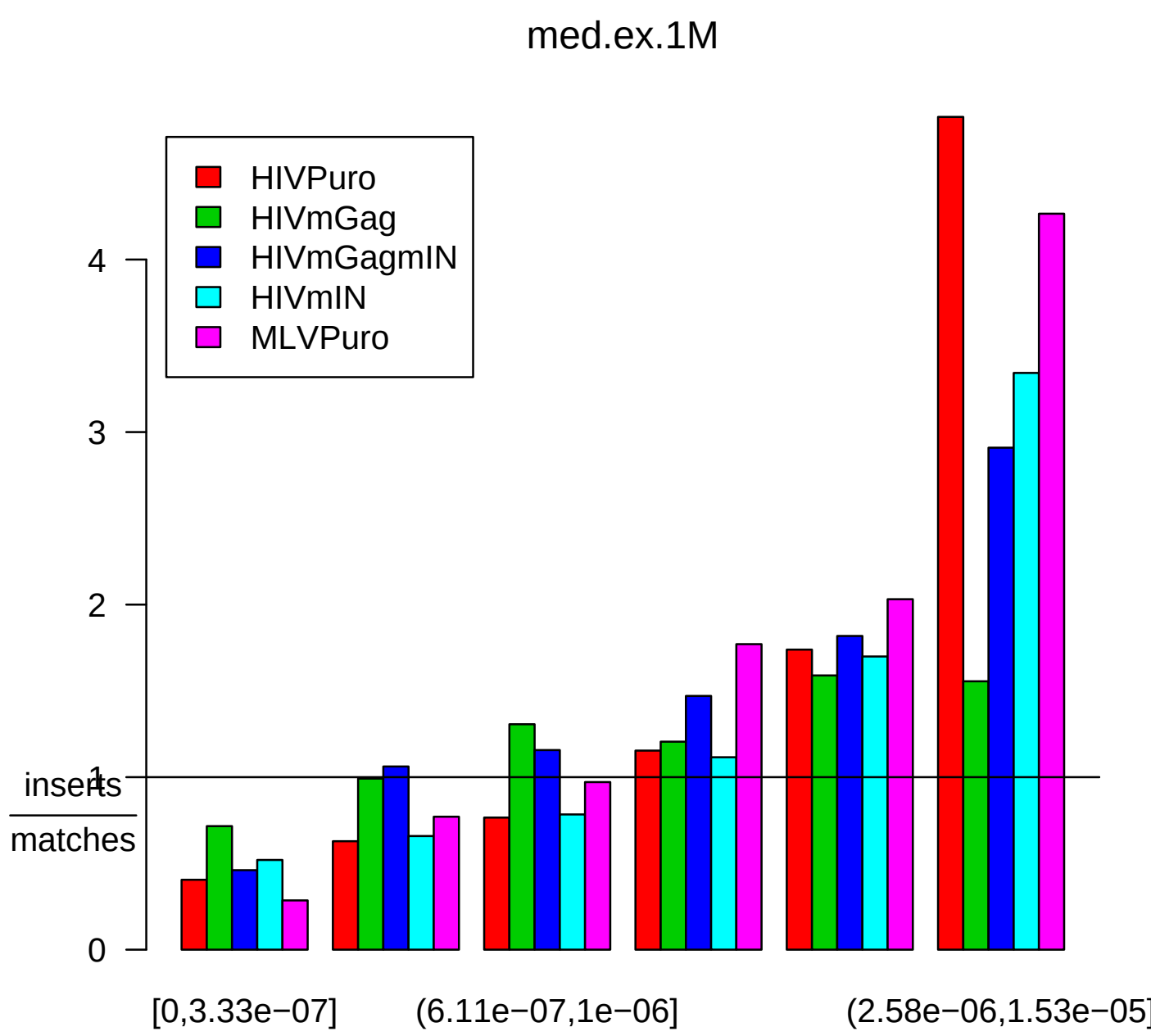

	coef	se	z	p
HIVPuro	1.420	0.1110	12.80	1.54e-37
HIVmGag	0.618	0.0973	6.36	2.08e-10
HIVmGagmIN	1.250	0.1060	11.90	2.12e-32
HIVmIN	1.080	0.1280	8.43	3.60e-17
MLVPuro	1.870	0.1230	15.20	4.01e-52

And here we count genes in the upper $1/16^{th}$:

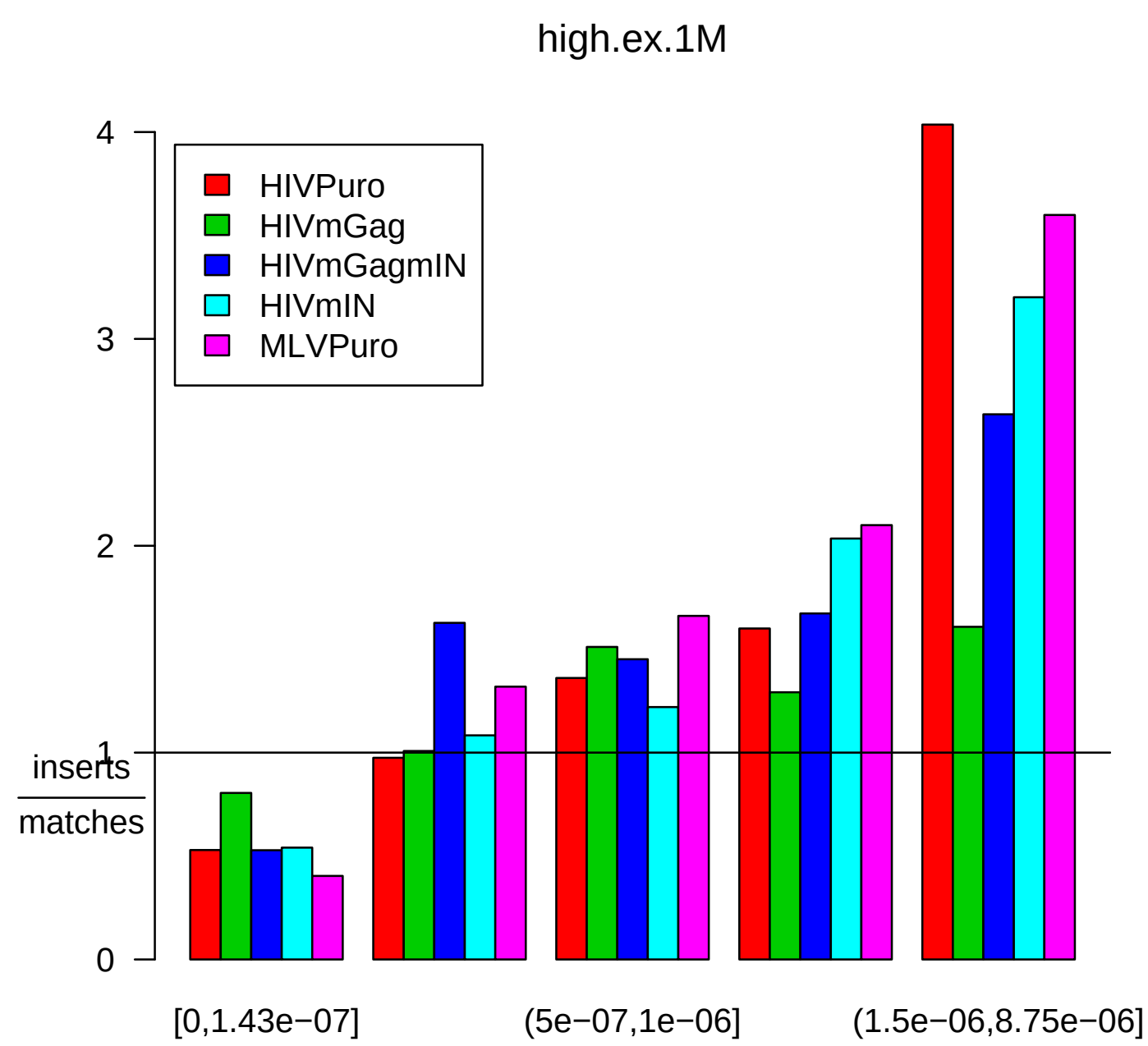

	coef	se	z	p
HIVPuro	1.220	0.0973	12.50	7.20e-36
HIVmGag	0.482	0.0946	5.09	3.55e-07
HIVmGagmIN	1.210	0.0972	12.40	2.38e-35
HIVmIN	1.170	0.1200	9.73	2.27e-22
MLVPuro	1.610	0.1030	15.70	1.97e-55

Here the effect of density of CpG islands is studied:

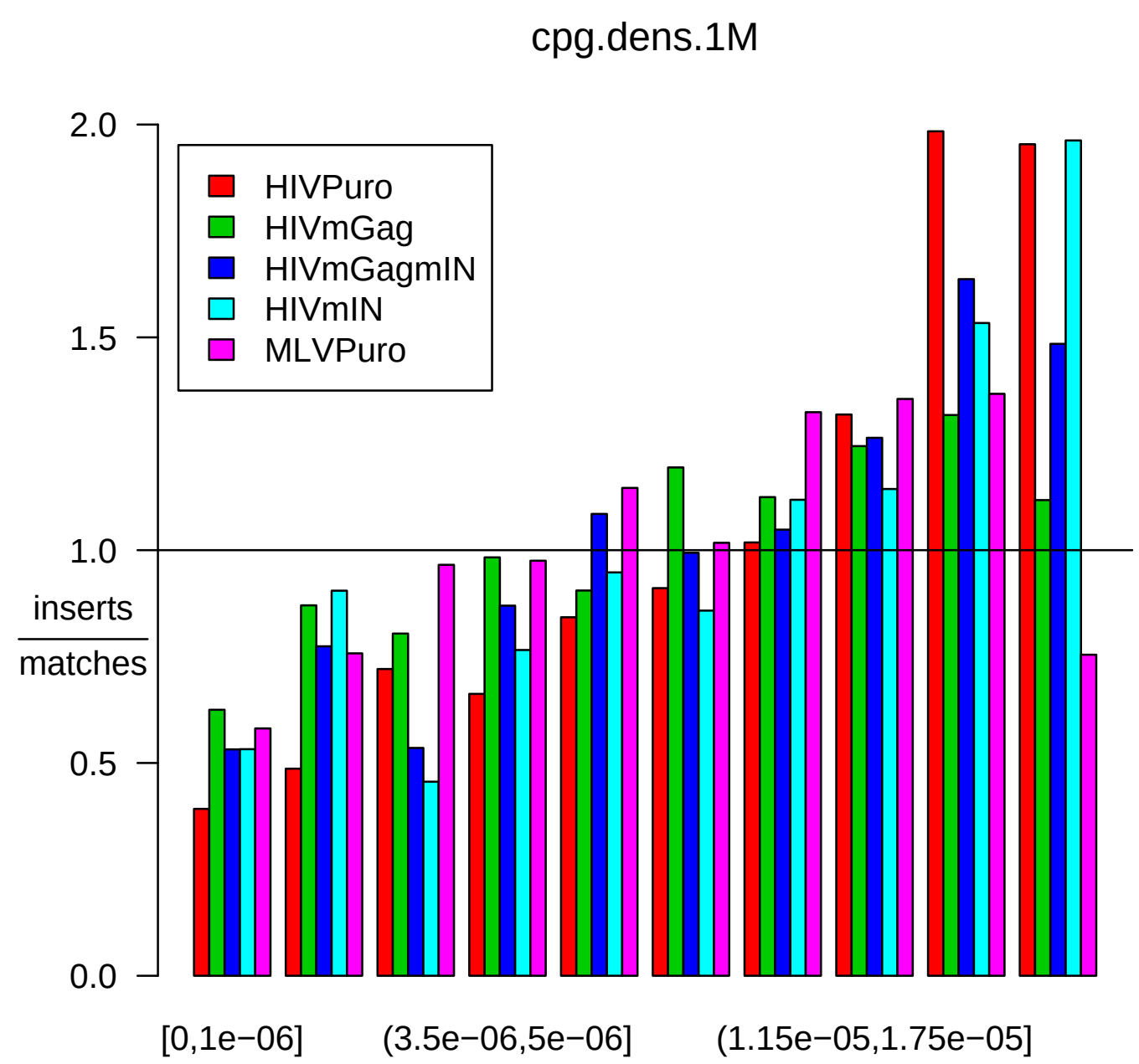

	coef	se	z	p
HIVPuro	0.858	0.0978	8.77	1.84e-18
HIVmGag	0.366	0.0949	3.86	1.14e-04
HIVmGagmIN	0.539	0.0932	5.79	7.16e-09
HIVmIN	0.606	0.1150	5.25	1.50e-07
MLVPuro	0.295	0.0904	3.26	1.11e-03

4.7 2 megabase Window

In the barplot that follows we examine the association of insertion sites with expression density in a 2 megabase window surrounding each locus. First, we count just the number of genes represented on the chip.

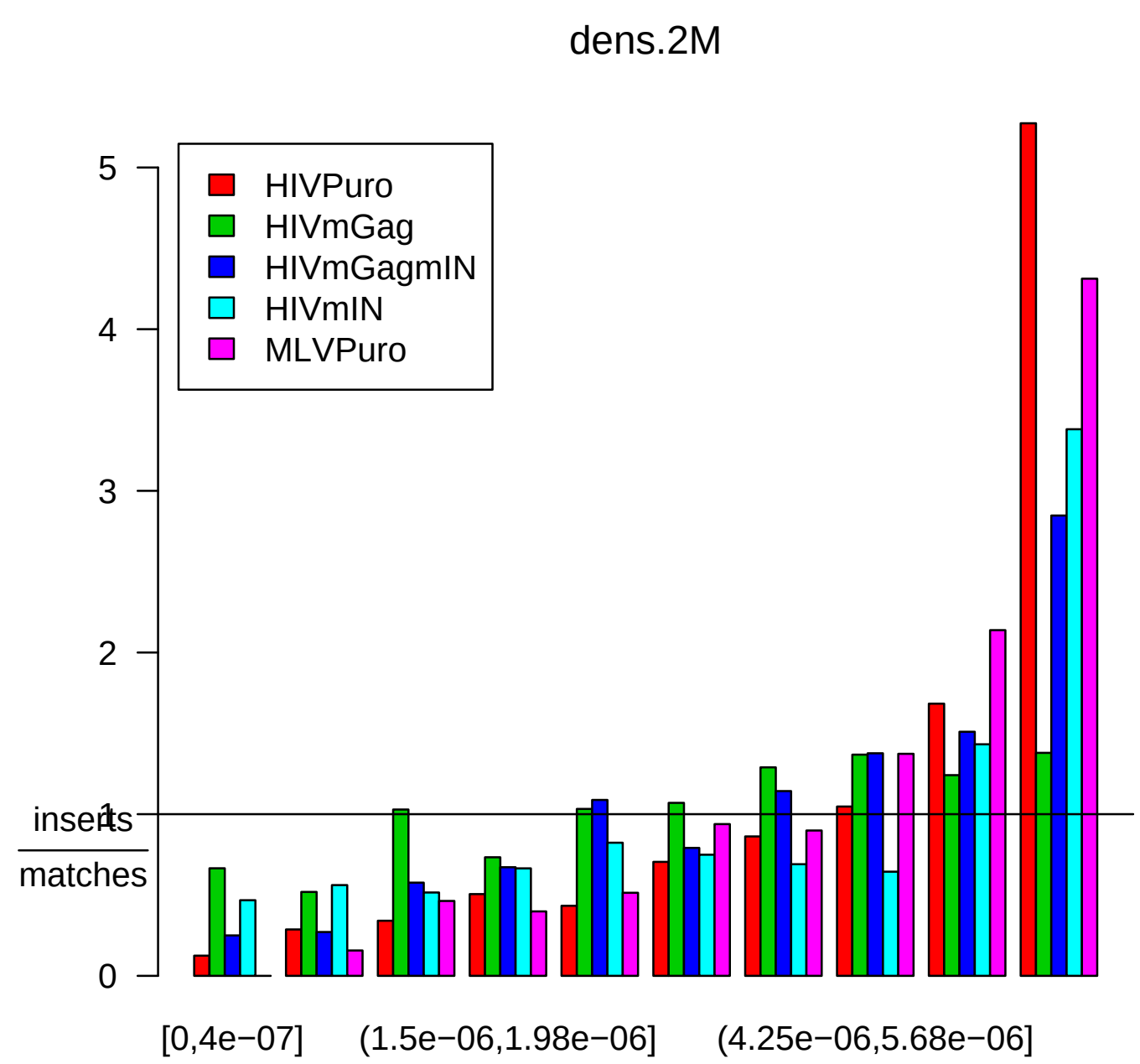

	coef	se	z	p
HIVPuro	1.640	0.1180	14.00	2.98e-44
HIVmGag	0.500	0.0963	5.19	2.09e-07
HIVmGagmIN	0.979	0.1000	9.75	1.83e-22
HIVmIN	0.838	0.1230	6.84	8.13e-12
MLVPuro	1.840	0.1220	15.10	3.14e-51

Here are the results for expression density. First, we count just genes that are in the upper half.

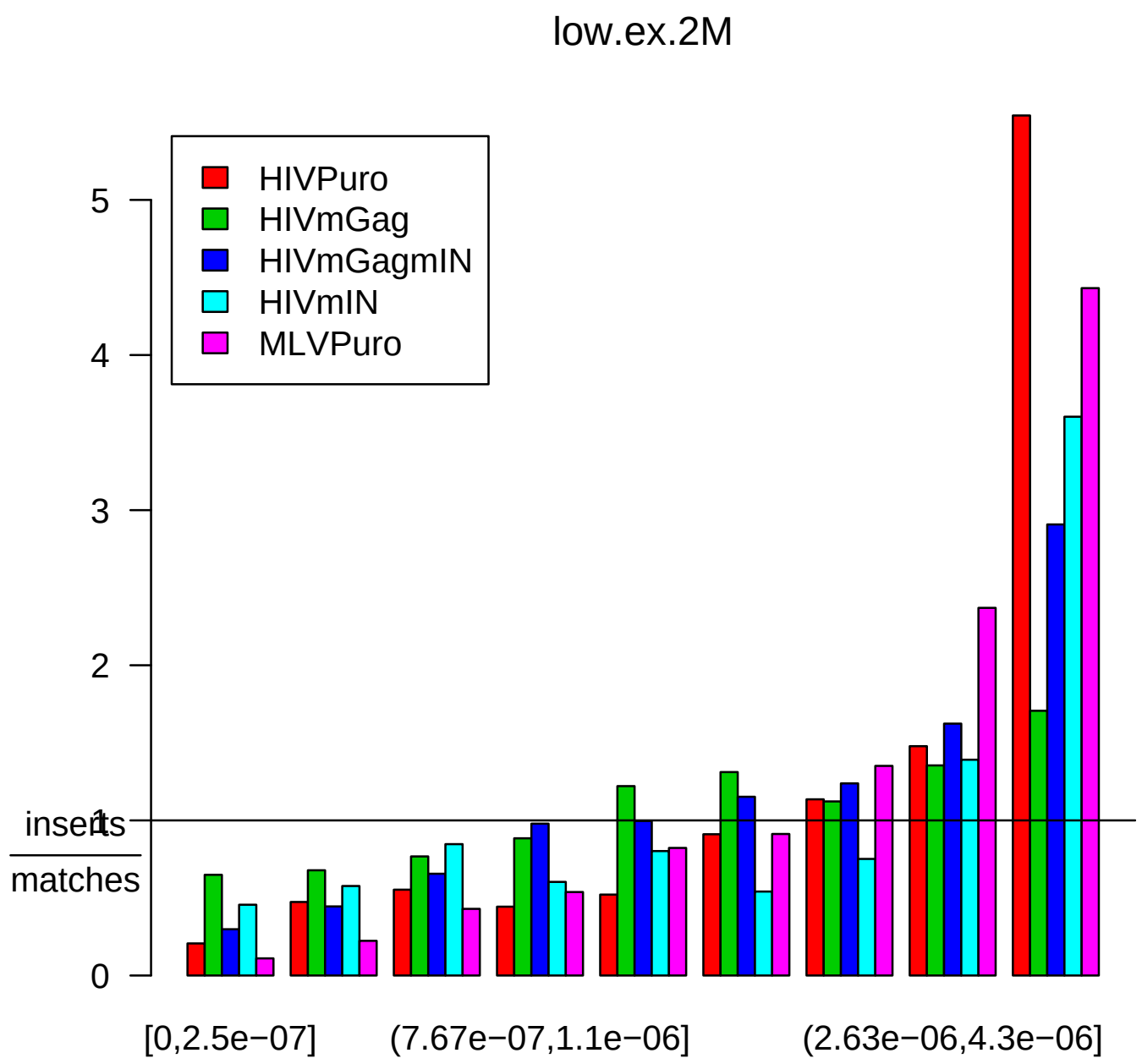

	coef	se	z	p
HIVPuro	1.570	0.1160	13.60	6.00e-42
HIVmGag	0.615	0.0973	6.32	2.61e-10
HIVmGagmIN	1.100	0.1030	10.60	1.78e-26
HIVmIN	0.922	0.1250	7.37	1.75e-13
MLVPuro	1.940	0.1250	15.50	4.24e-54

Now we count genes in the upper $1/8^{th}$:

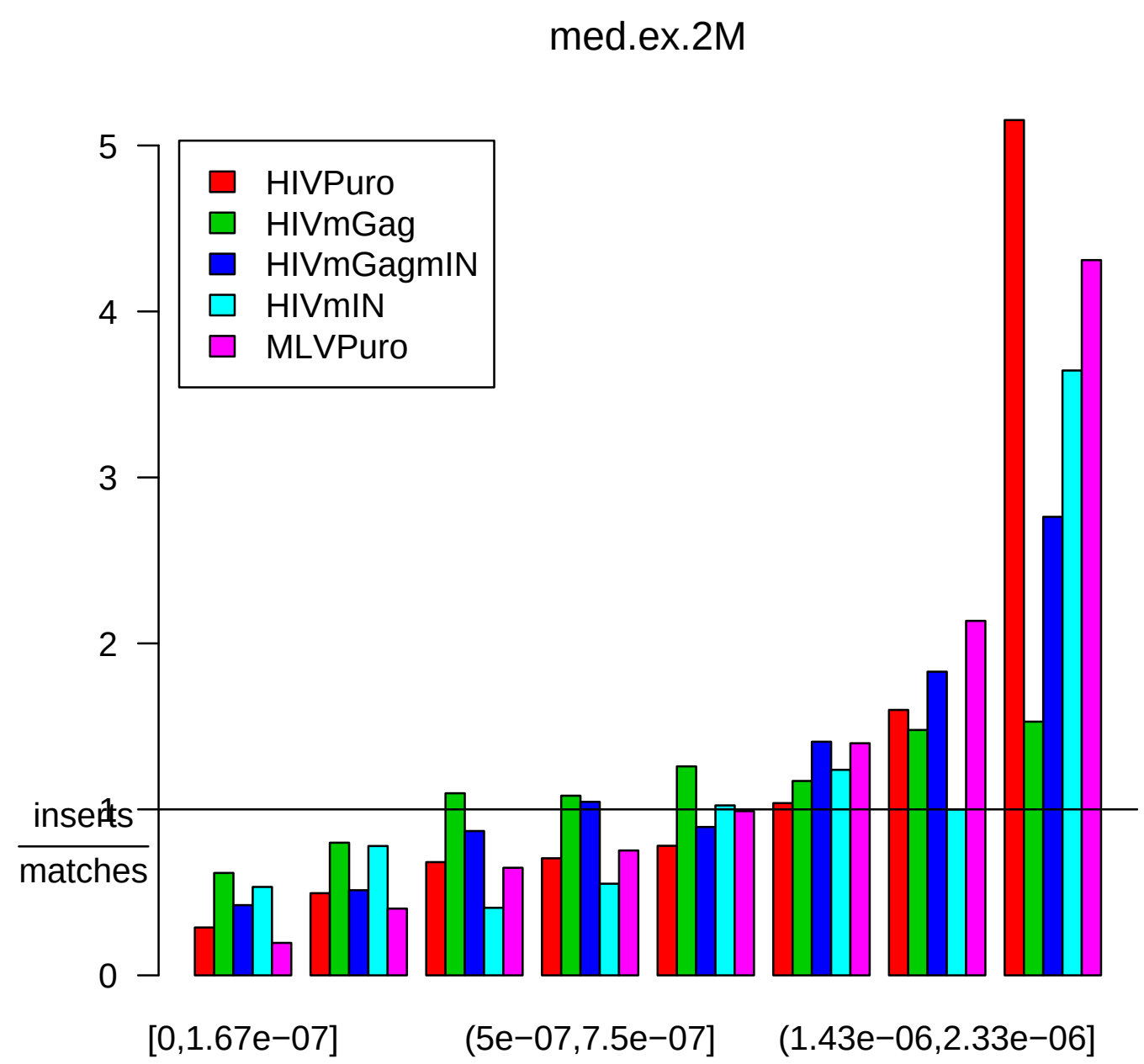

	coef	se	z	p
HIVPuro	1.410	0.108	13.10	2.94e-39
HIVmGag	0.543	0.096	5.65	1.59e-08
HIVmGagmIN	1.070	0.100	10.70	9.02e-27
HIVmIN	1.030	0.124	8.34	7.32e-17
MLVPuro	1.700	0.114	14.90	3.52e-50

And here we count genes in the upper $1/16^{th}$:

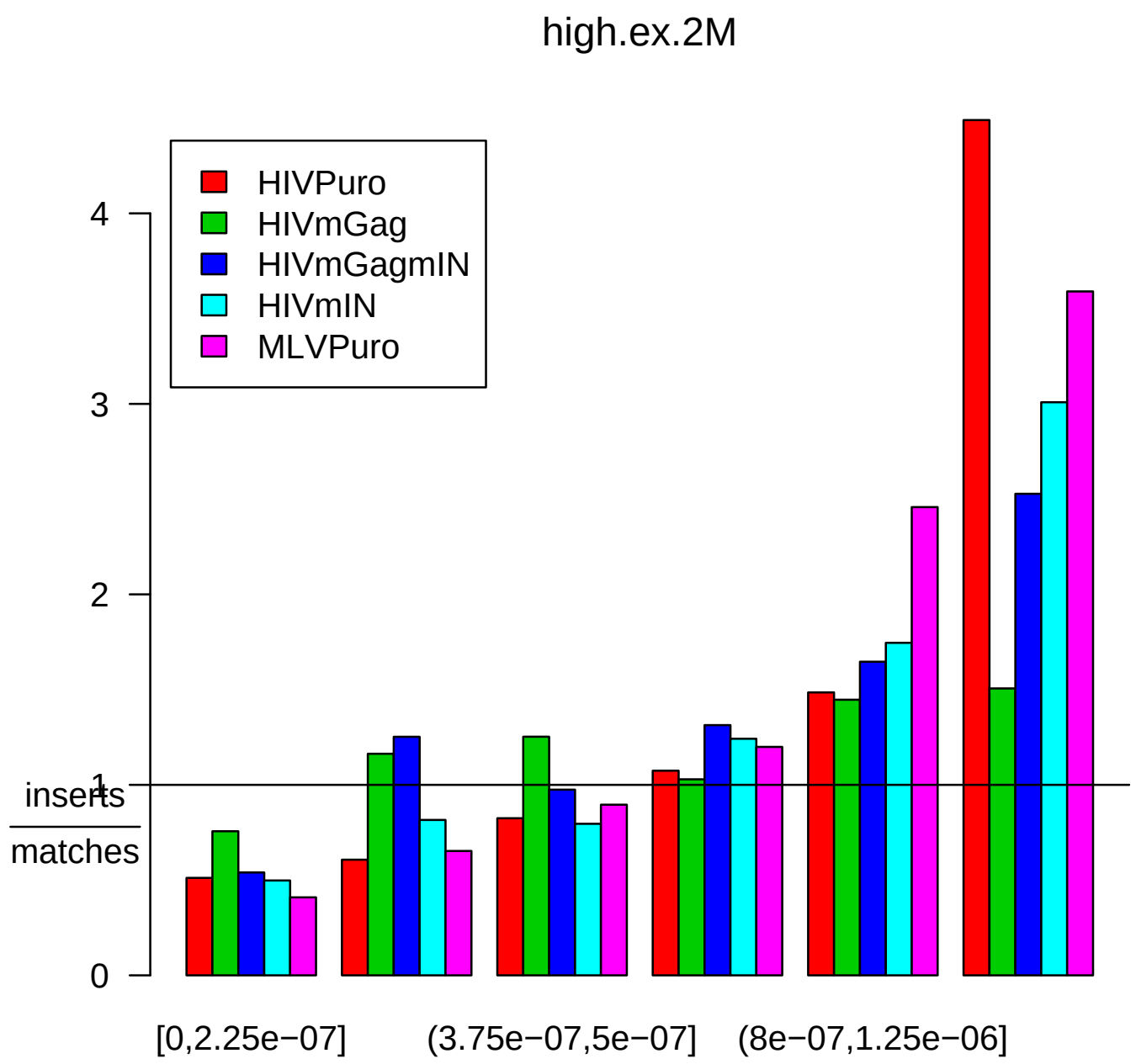


```
             coef     se     z        p
HIVPuro     1.090 0.1030 10.60 3.03e-26
HIVmGag     0.525 0.0967  5.43 5.71e-08
HIVmGagmIN  1.030 0.1010 10.20 1.29e-24
HIVmIN      1.120 0.1280  8.73 2.55e-18
MLVPuro     1.400 0.1090 12.90 2.98e-38
```

Here the effect of density of CpG islands is studied:

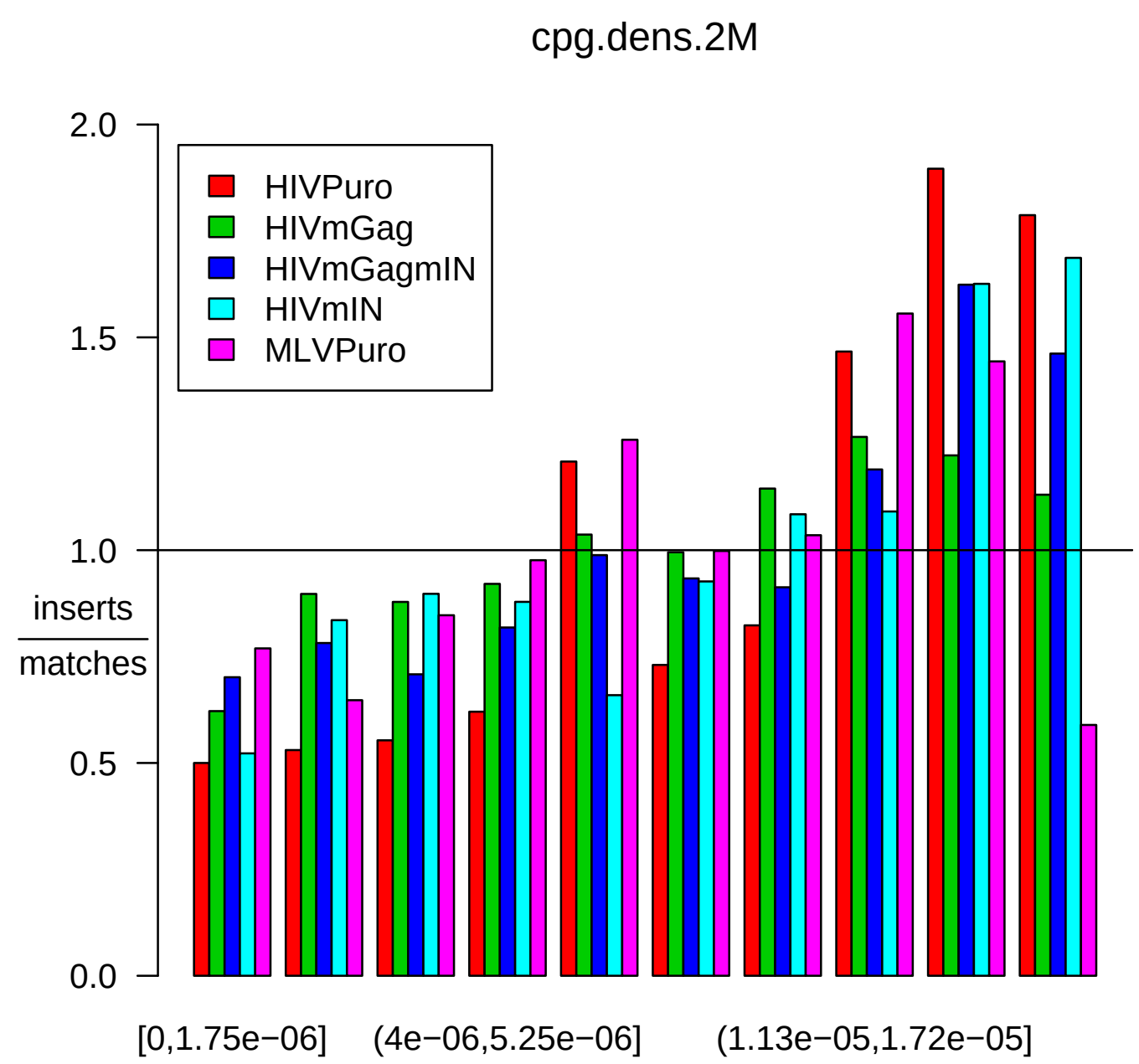

	coef	se	z	p
HIVPuro	0.665	0.0954	6.98	3.05e-12
HIVmGag	0.291	0.0947	3.07	2.11e-03
HIVmGagmIN	0.427	0.0924	4.62	3.78e-06
HIVmIN	0.540	0.1140	4.72	2.38e-06
MLVPuro	0.222	0.0905	2.45	1.42e-02

4.8 4 megabase Window

In the barplot that follows we examine the association of insertion sites with expression density in a 4 megabase window surrounding each locus. First, we count just the number of genes represented on the chip.

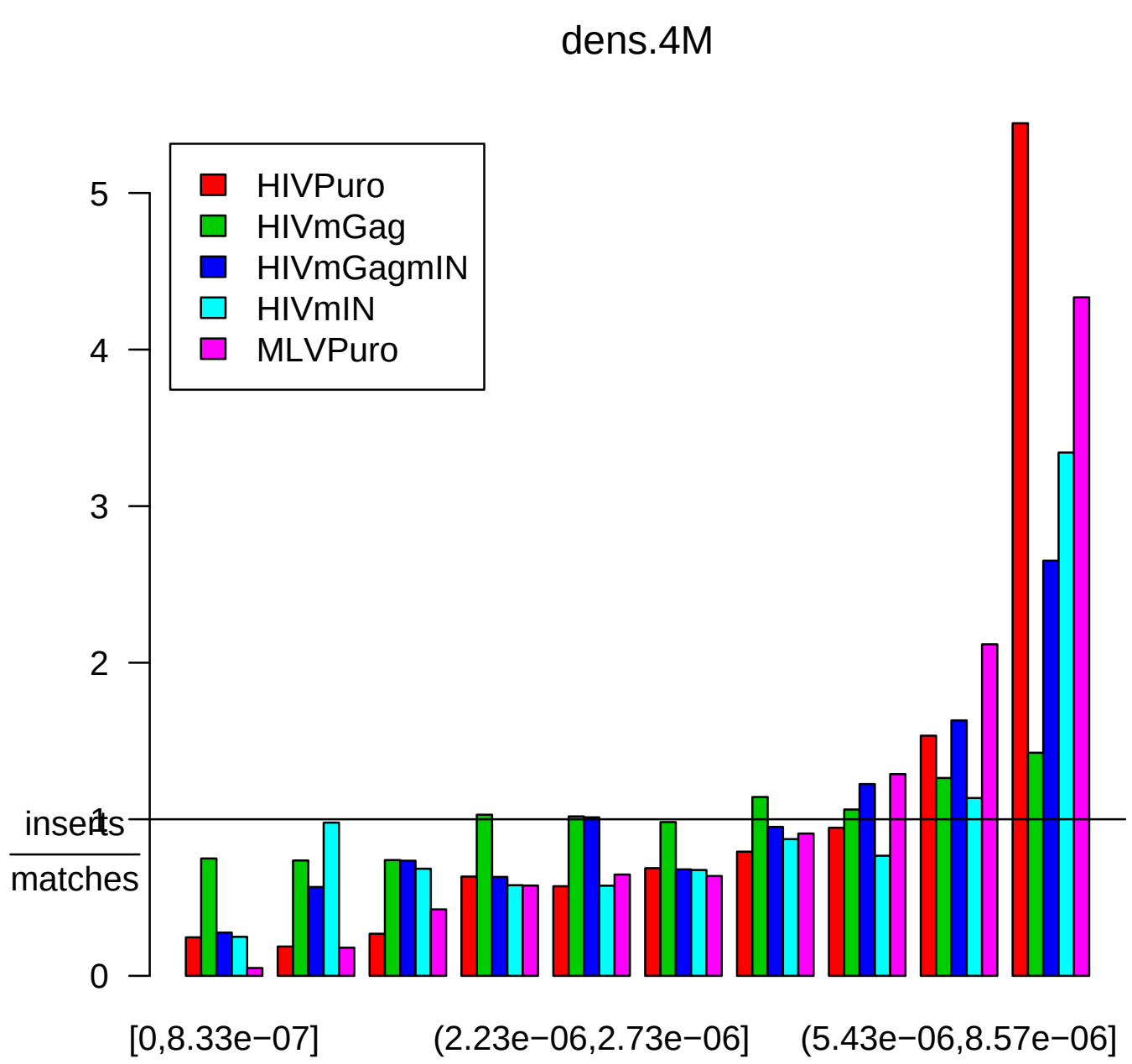

	coef	se	z	p
HIVPuro	1.480	0.1120	13.20	8.13e-40
HIVmGag	0.314	0.0958	3.28	1.05e-03
HIVmGagmIN	0.769	0.0963	7.99	1.38e-15
HIVmIN	0.831	0.1230	6.75	1.50e-11
MLVPuro	1.540	0.1120	13.70	8.51e-43

Here are the results for expression density. First, we count just genes that are in the upper half.

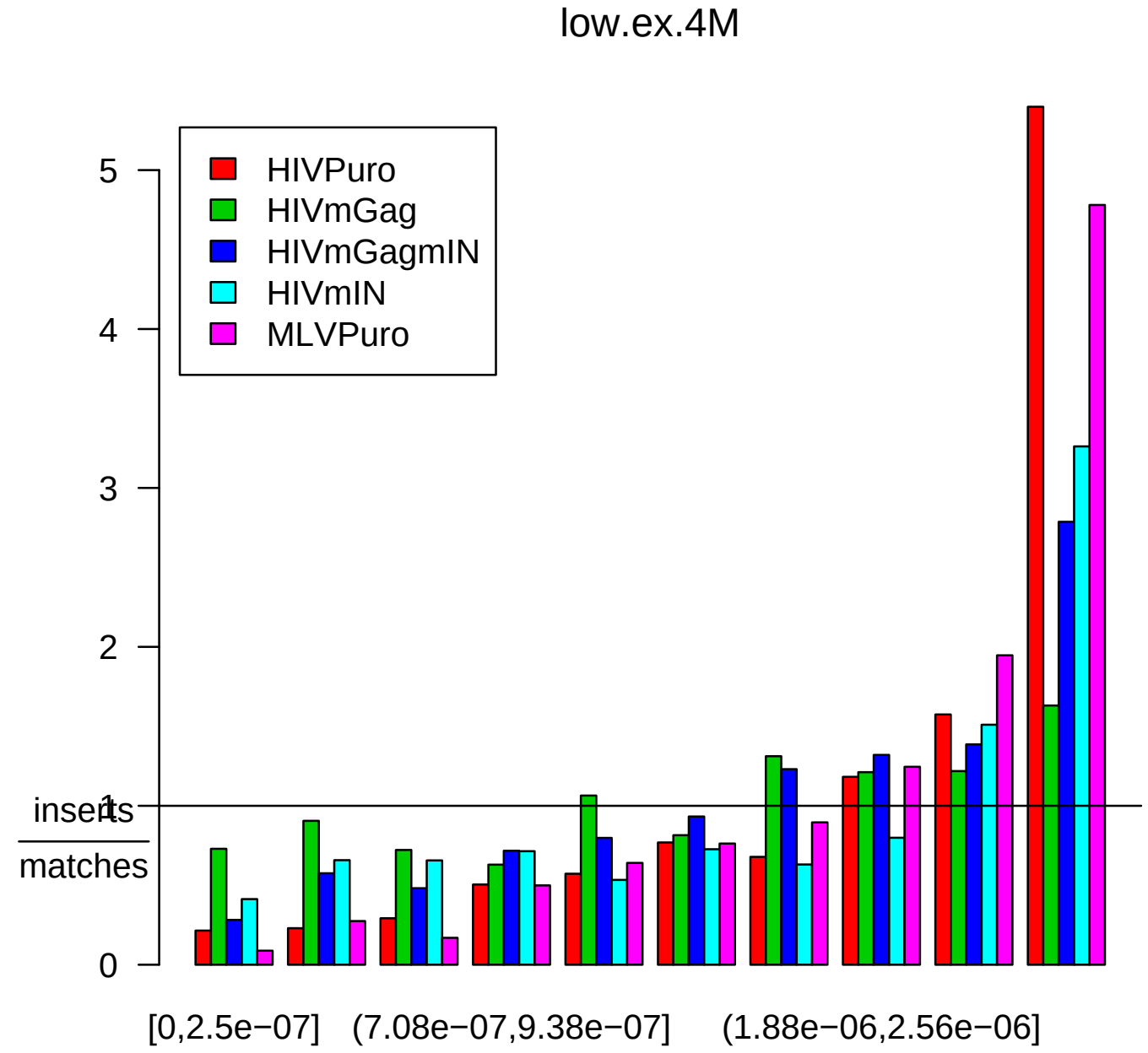

	coef	se	z	p
HIVPuro	1.550	0.1150	13.50	1.88e-41
HIVmGag	0.415	0.0956	4.34	1.45e-05
HIVmGagmIN	0.984	0.1000	9.84	7.66e-23
HIVmIN	0.910	0.1250	7.31	2.70e-13
MLVPuro	1.690	0.1160	14.50	1.37e-47

Now we count genes in the upper $1/8^{th}$:

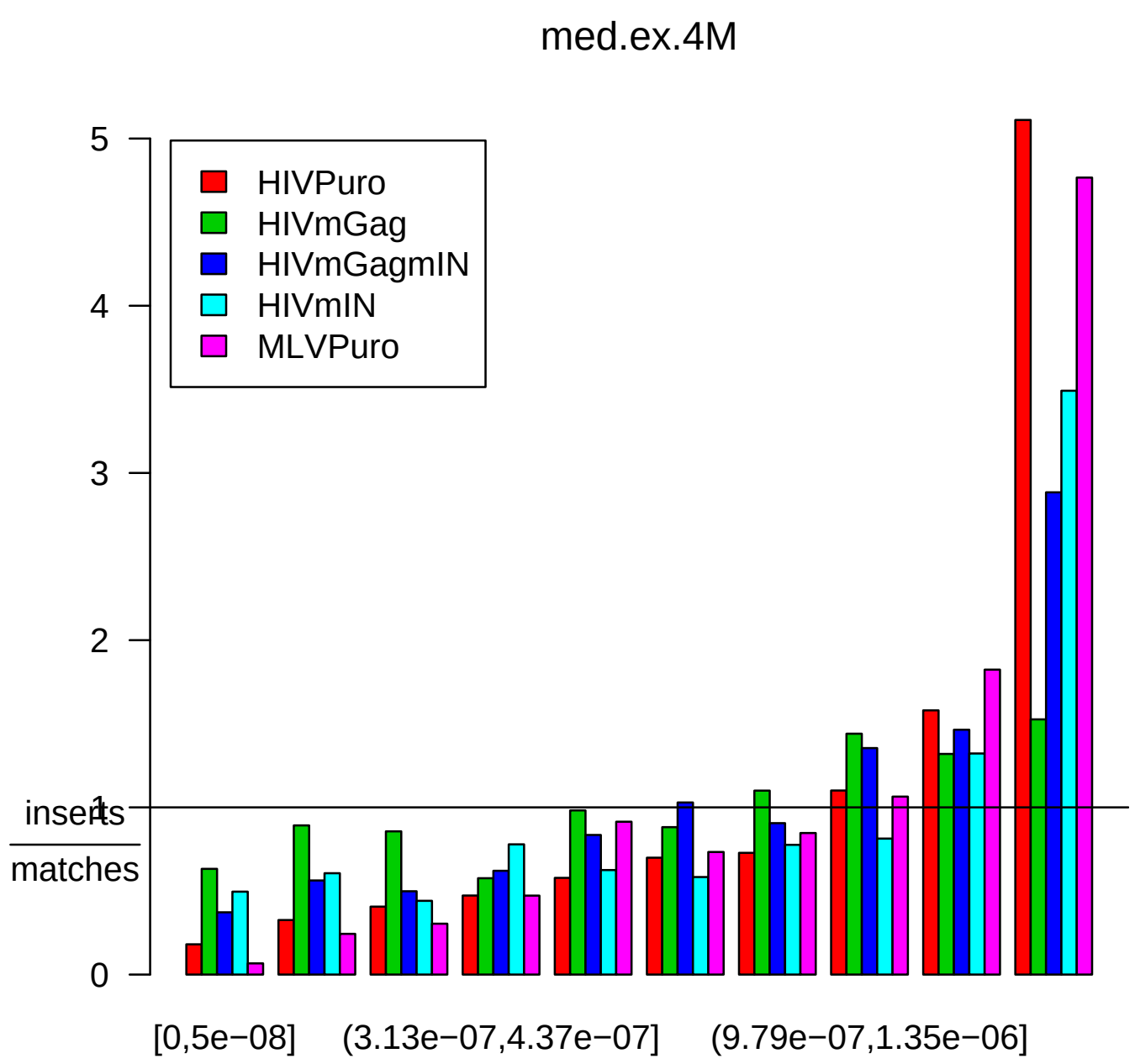

	coef	se	z	p
HIVPuro	1.450	0.1120	13.00	1.63e-38
HIVmGag	0.456	0.0959	4.76	1.94e-06
HIVmGagmIN	0.945	0.0989	9.55	1.32e-21
HIVmIN	0.890	0.1230	7.21	5.64e-13
MLVPuro	1.450	0.1090	13.30	2.47e-40

And here we count genes in the upper $1/16^{th}$:

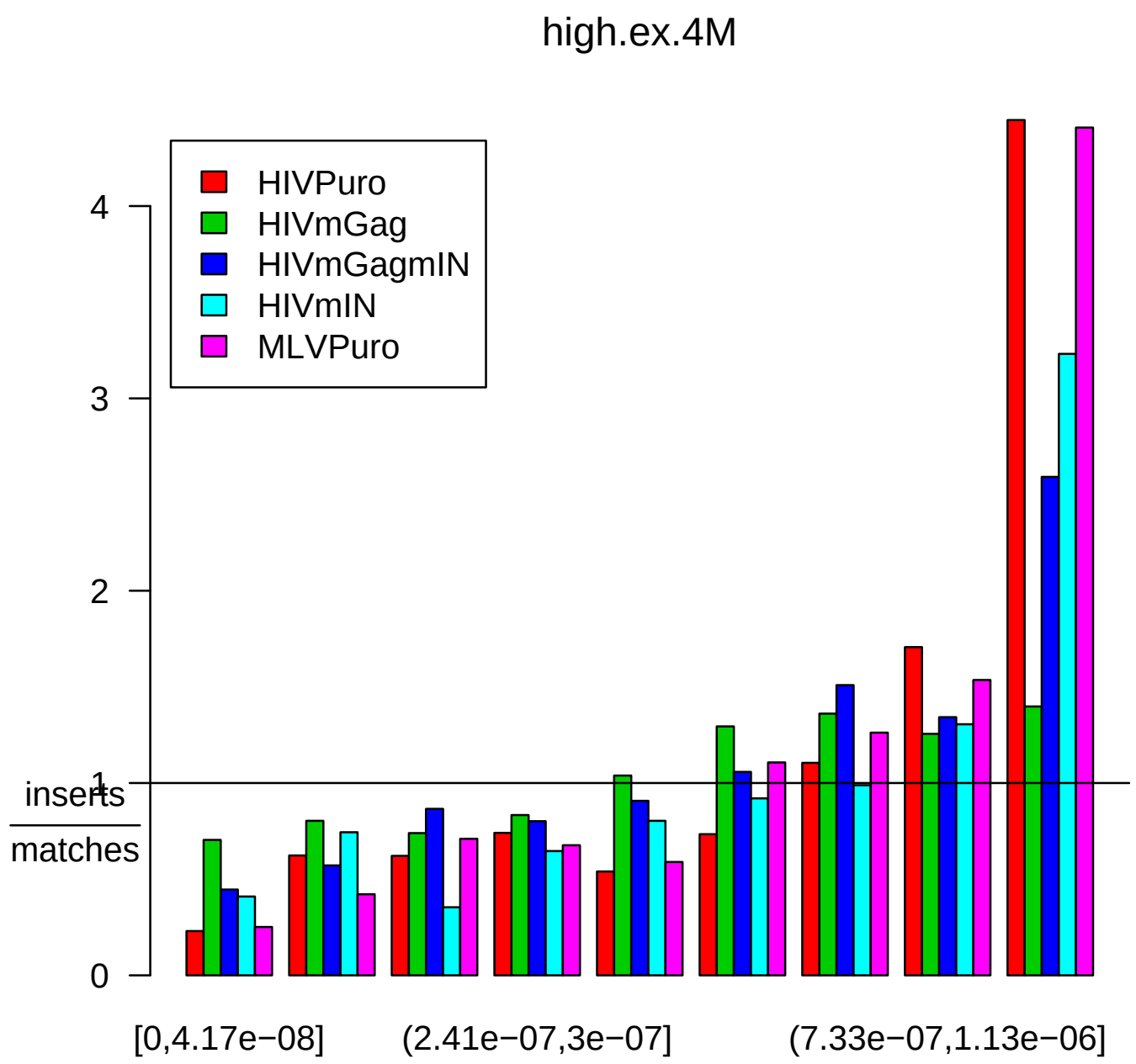

	coef	se	z	p
HIVPuro	1.170	0.1050	11.10	7.41e-29
HIVmGag	0.509	0.0963	5.28	1.28e-07
HIVmGagmIN	0.878	0.0979	8.97	3.07e-19
HIVmIN	1.020	0.1260	8.03	9.49e-16
MLVPuro	1.320	0.1060	12.50	1.34e-35

Here the effect of density of CpG islands is studied:

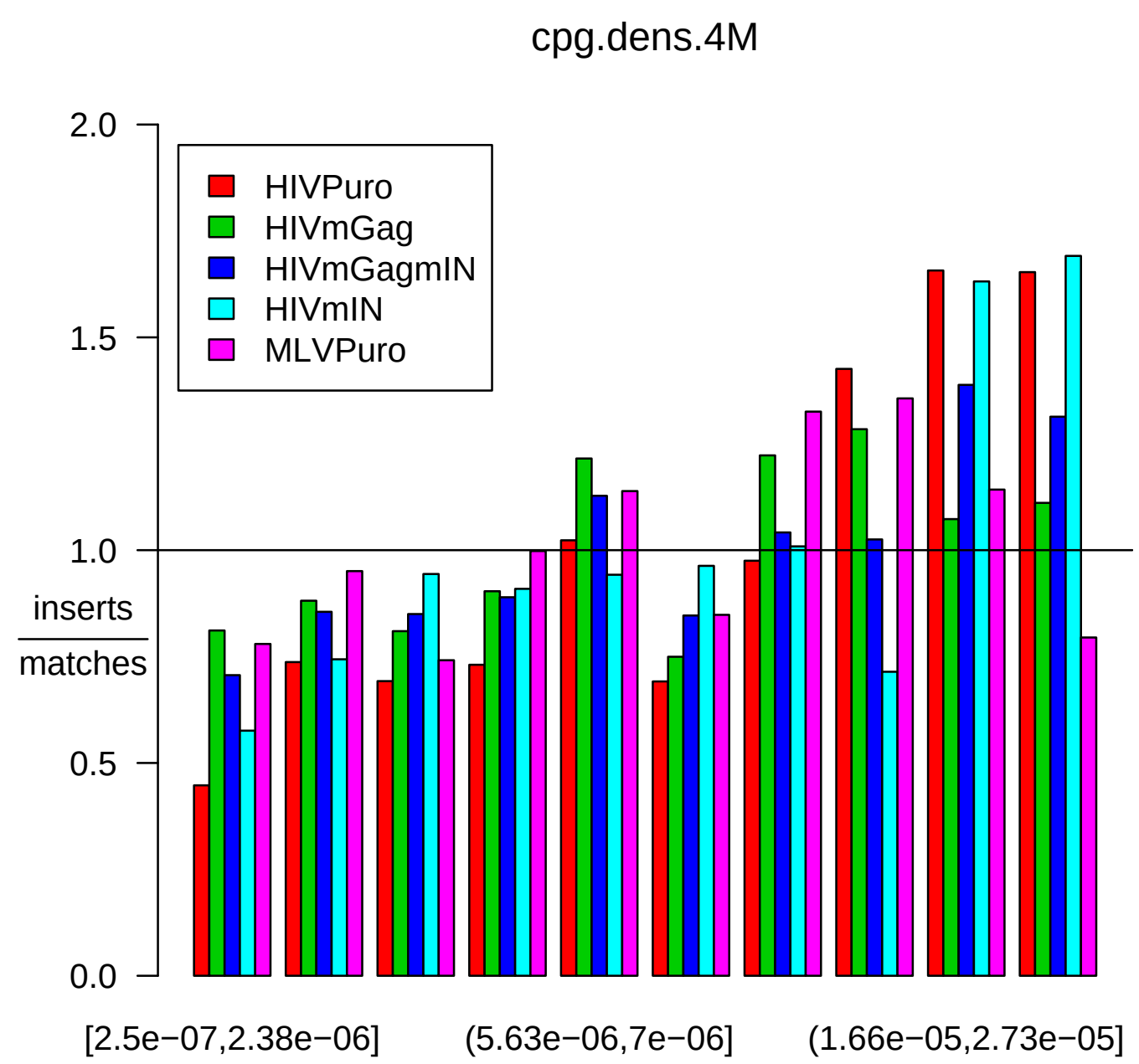

	coef	se	z	p
HIVPuro	0.553	0.0942	5.87	4.31e-09
HIVmGag	0.163	0.0944	1.73	8.40e-02
HIVmGagmIN	0.238	0.0919	2.59	9.52e-03
HIVmIN	0.381	0.1130	3.36	7.79e-04
MLVPuro	0.165	0.0900	1.84	6.63e-02

4.9 8 megabase Window

In the barplot that follows we examine the association of insertion sites with expression density in a 8 megabase window surrounding each locus. First, we count just the number of genes represented on the chip.

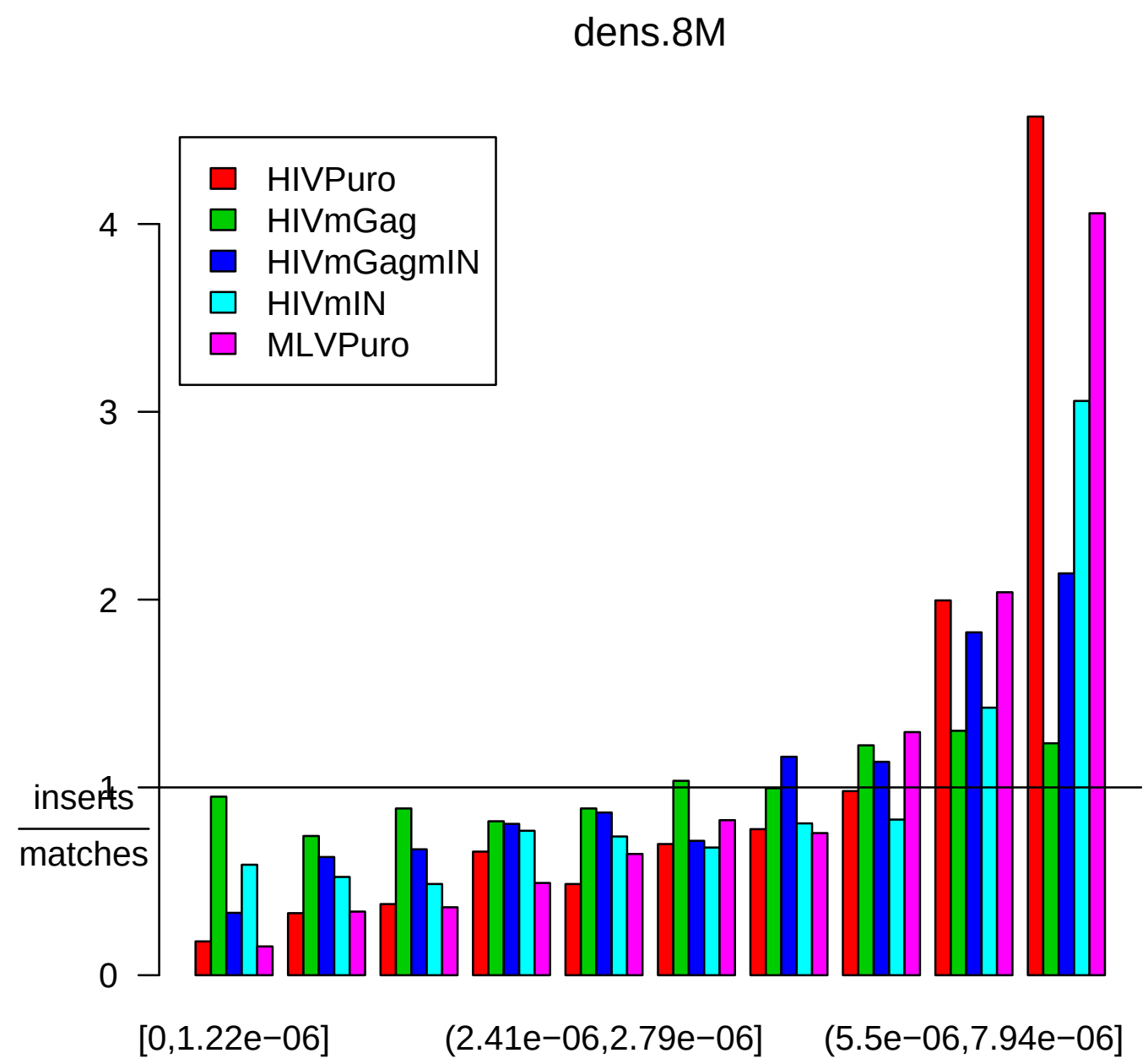

	coef	se	z	p
HIVPuro	1.420	0.1110	12.80	1.24e-37
HIVmGag	0.294	0.0951	3.09	2.00e-03
HIVmGagmIN	0.735	0.0964	7.62	2.50e-14
HIVmIN	0.806	0.1220	6.63	3.47e-11
MLVPuro	1.450	0.1090	13.30	4.44e-40

Here are the results for expression density. First, we count just genes that are in the upper half.

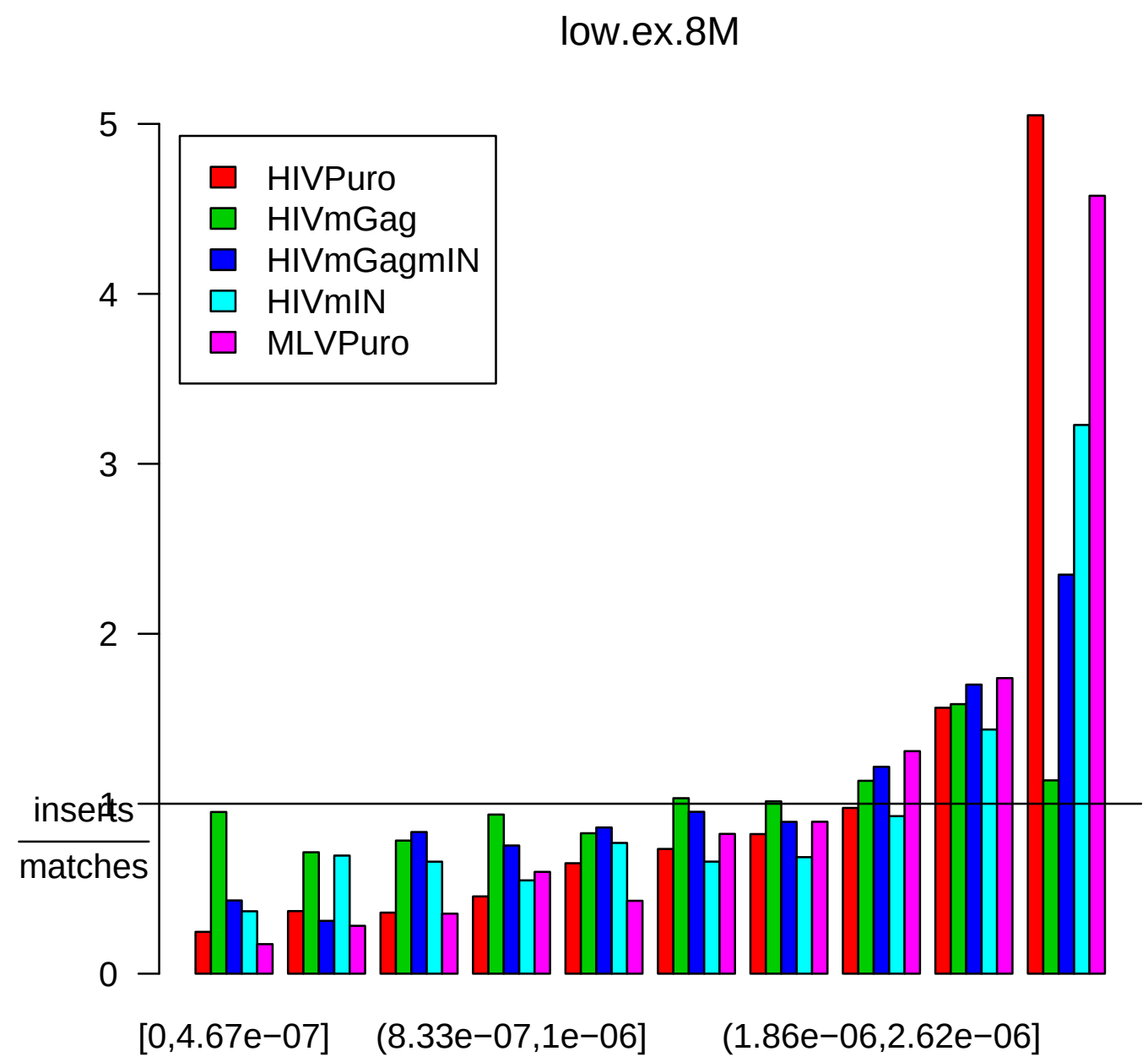

	coef	se	z	p
HIVPuro	1.370	0.1100	12.50	5.86e-36
HIVmGag	0.335	0.0952	3.52	4.32e-04
HIVmGagmIN	0.791	0.0968	8.18	2.95e-16
HIVmIN	0.836	0.1230	6.81	9.95e-12
MLVPuro	1.550	0.1120	13.90	8.97e-44

Now we count genes in the upper $1/8^{th}$:

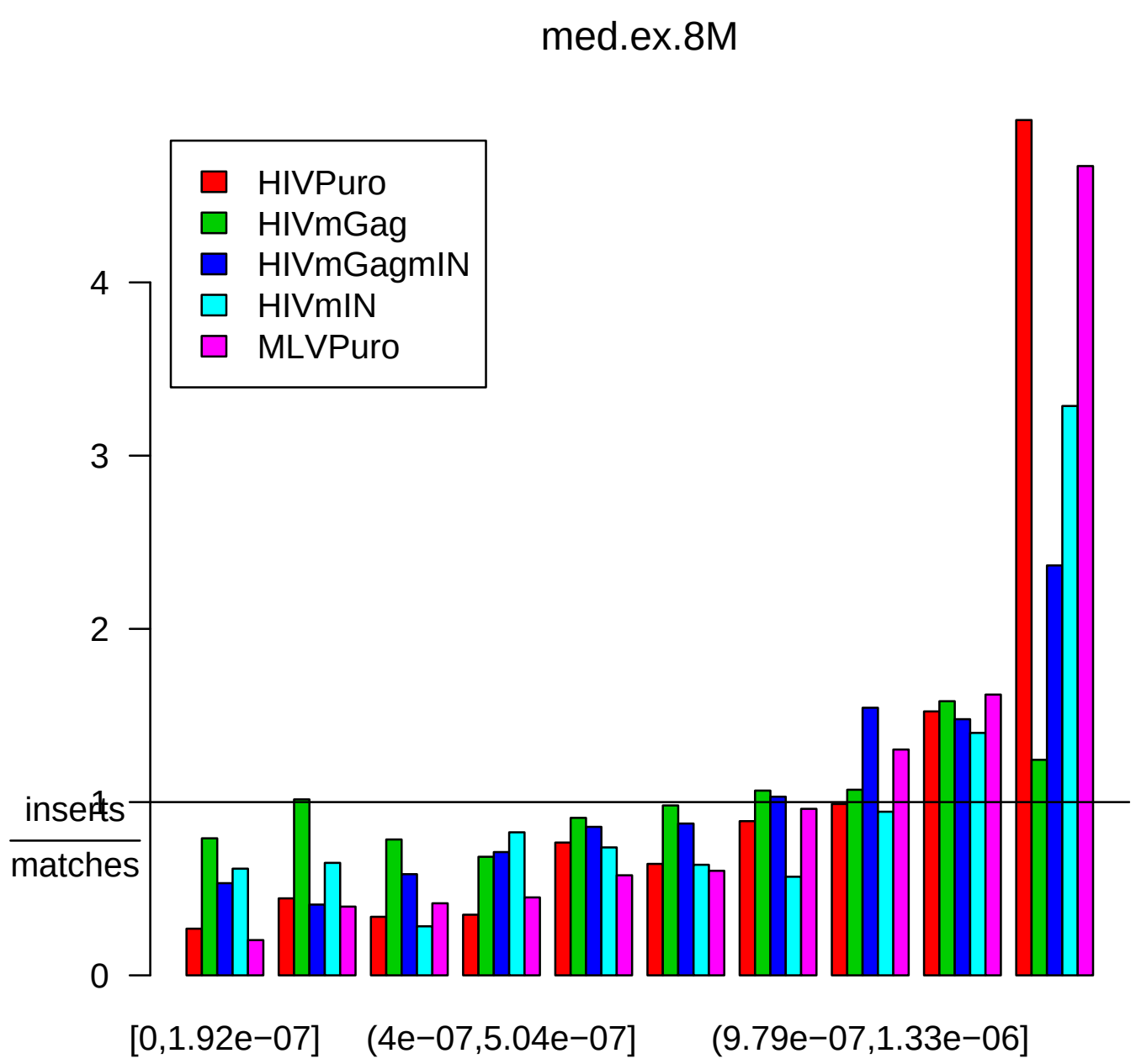

	coef	se	z	p
HIVPuro	1.310	0.1080	12.10	7.09e-34
HIVmGag	0.348	0.0950	3.66	2.53e-04
HIVmGagmIN	0.858	0.0978	8.78	1.67e-18
HIVmIN	0.789	0.1230	6.43	1.31e-10
MLVPuro	1.410	0.1080	13.00	1.68e-38

And here we count genes in the upper $1/16^{th}$:

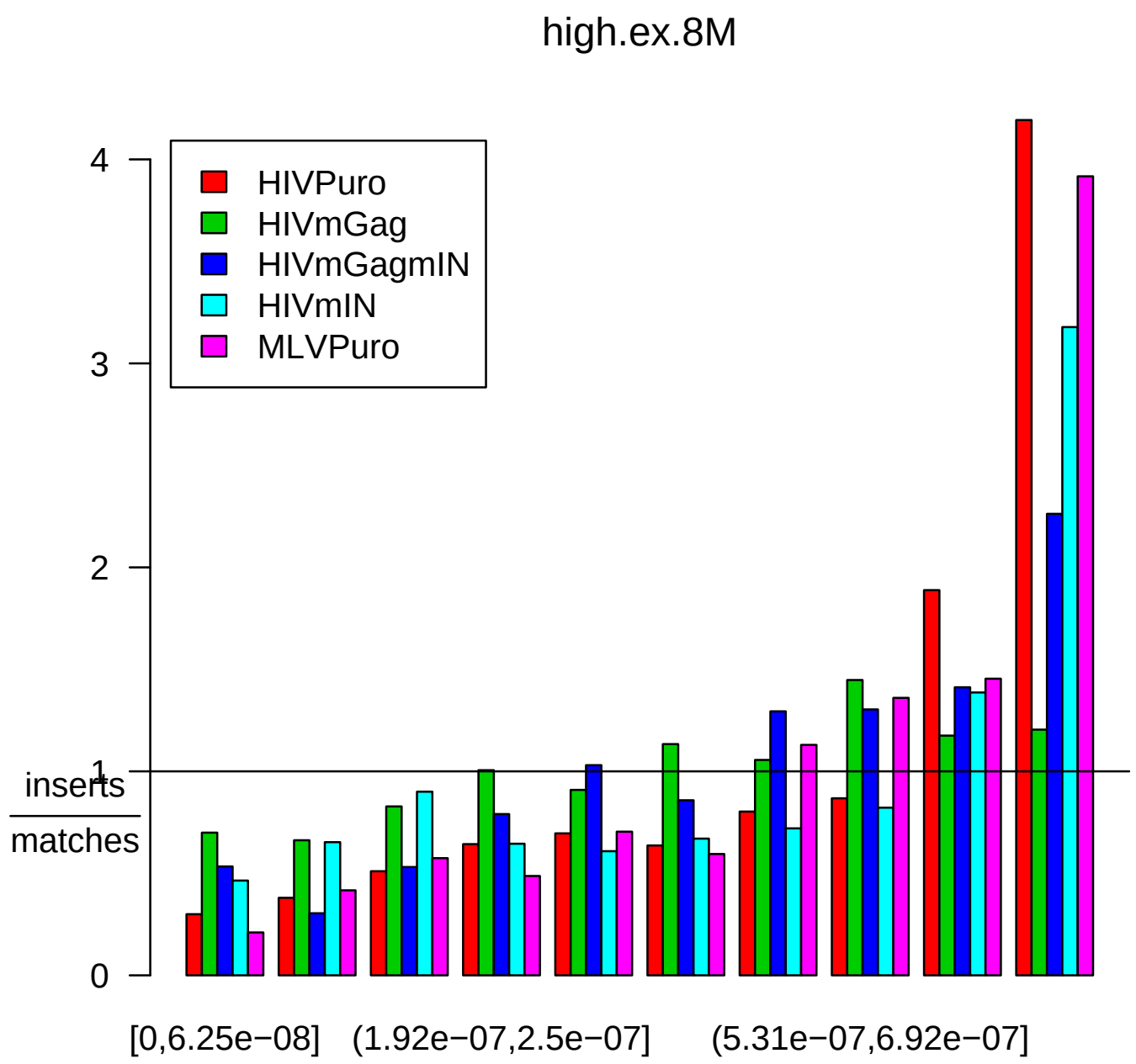

	coef	se	z	p
HIVPuro	1.130	0.1040	10.90	1.56e-27
HIVmGag	0.383	0.0956	4.00	6.28e-05
HIVmGagmIN	0.800	0.0973	8.22	2.10e-16
HIVmIN	0.746	0.1220	6.14	8.49e-10
MLVPuro	1.230	0.1030	11.90	7.96e-33

Here the effect of density of CpG islands is studied:

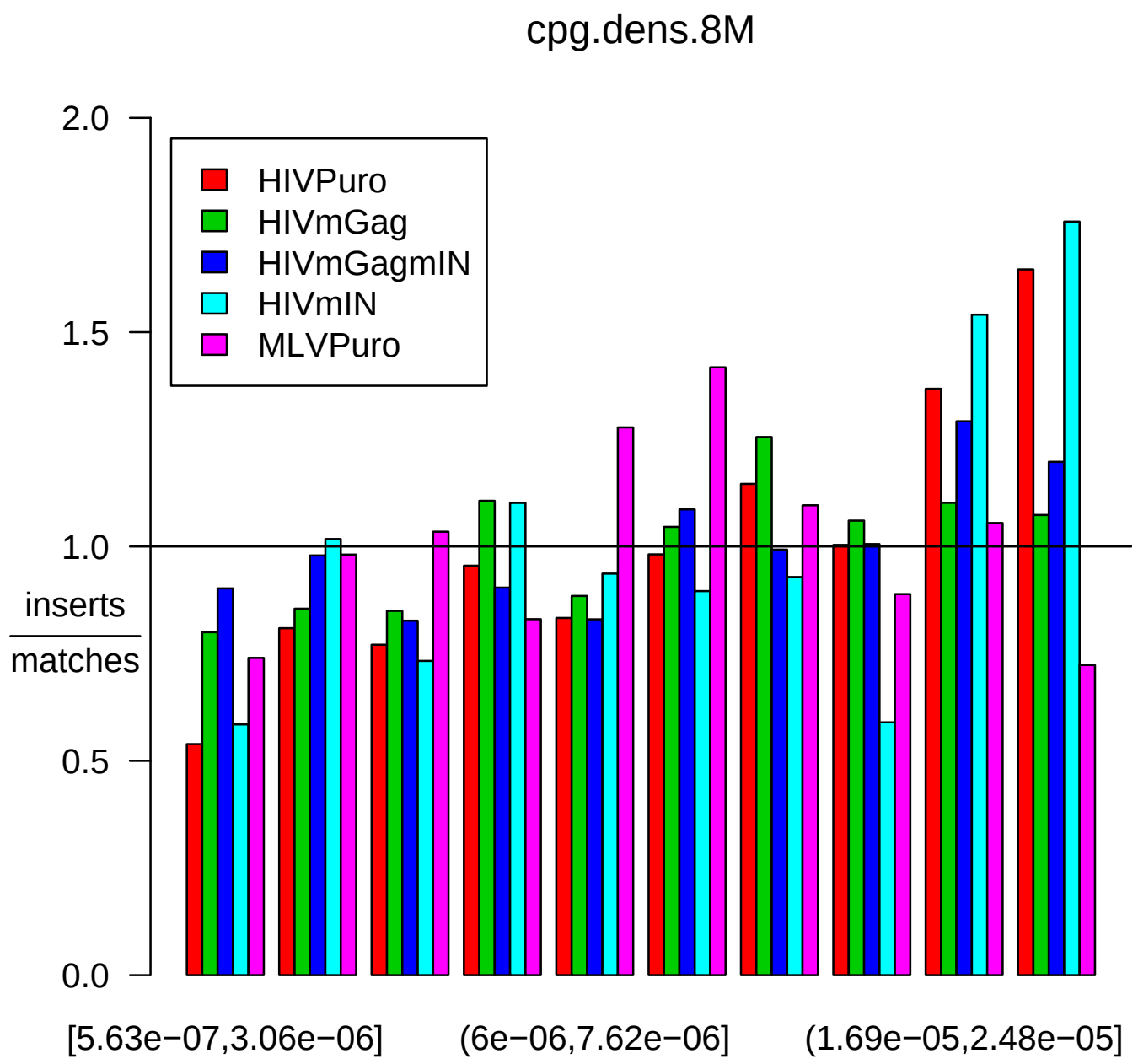

	coef	se	z	p
HIVPuro	0.446	0.0932	4.780	1.74e-06
HIVmGag	0.209	0.0947	2.210	2.71e-02
HIVmGagmIN	0.224	0.0921	2.430	1.50e-02
HIVmIN	0.253	0.1120	2.260	2.41e-02
MLVPuro	0.068	0.0902	0.754	4.51e-01

4.10 16 megabase Window

In the barplot that follows we examine the association of insertion sites with expression density in a 16 megabase window surrounding each locus. First, we count just the number of genes represented on the chip.

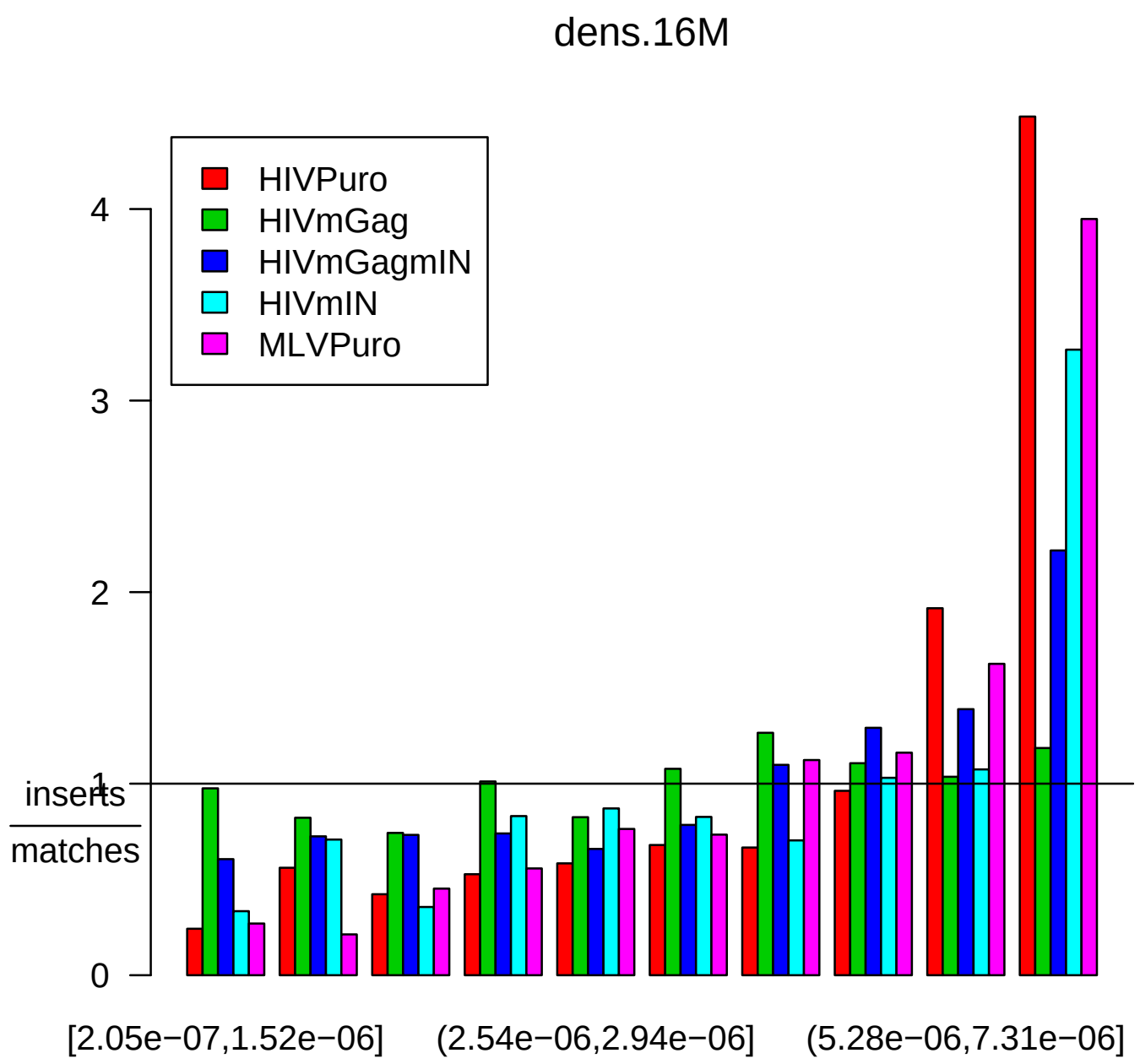

	coef	se	z	p
HIVPuro	1.220	0.1060	11.50	8.85e-31
HIVmGag	0.256	0.0948	2.70	6.92e-03
HIVmGagmIN	0.654	0.0952	6.88	6.20e-12
HIVmIN	0.803	0.1220	6.60	3.99e-11
MLVPuro	1.280	0.1050	12.20	2.07e-34

Here are the results for expression density. First, we count just genes that are in the upper half.

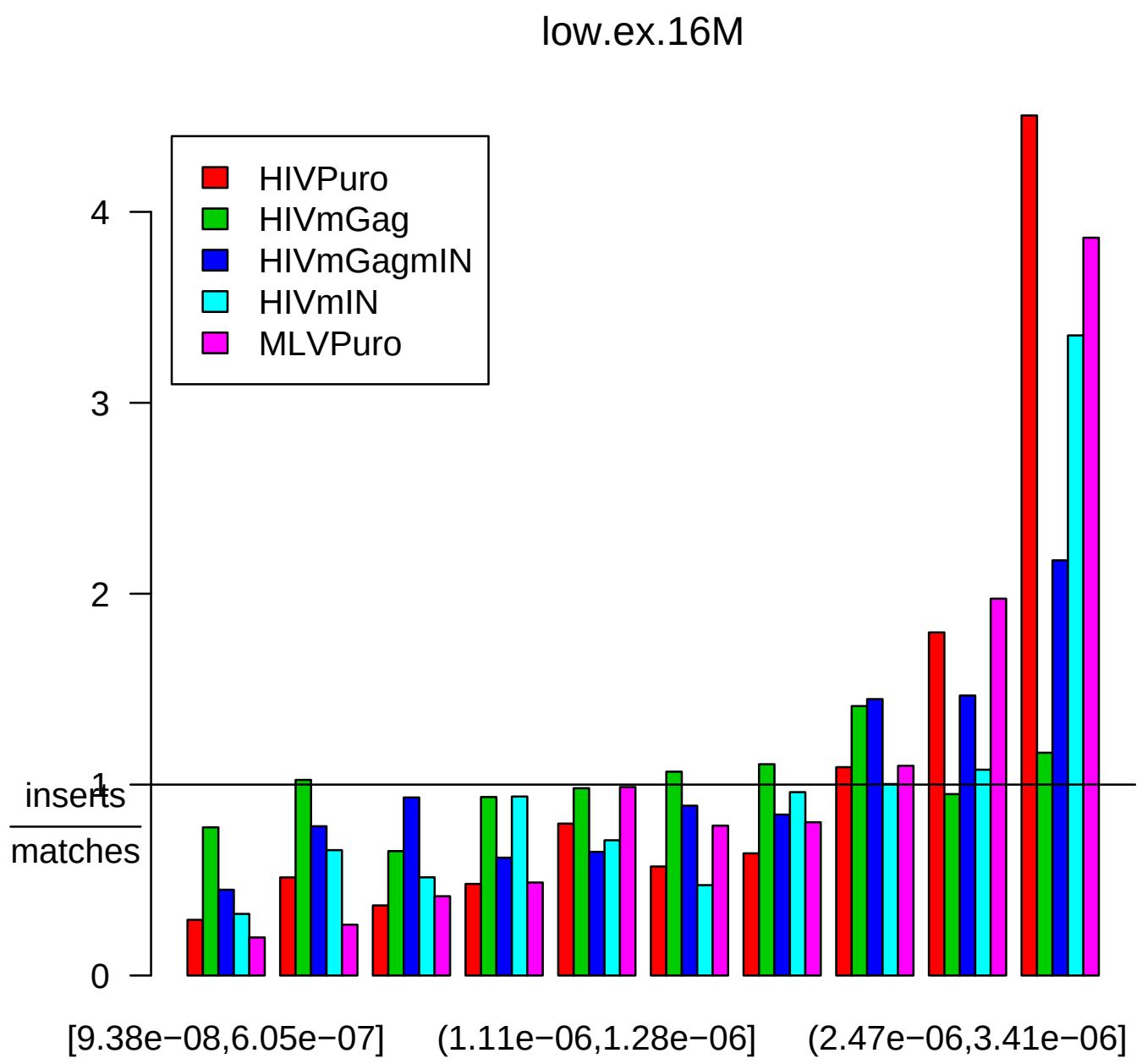

	coef	se	z	p
HIVPuro	1.140	0.1040	11.00	2.61e-28
HIVmGag	0.277	0.0951	2.92	3.55e-03
HIVmGagmIN	0.683	0.0957	7.13	1.01e-12
HIVmIN	0.788	0.1210	6.50	7.78e-11
MLVPuro	1.240	0.1040	12.00	4.78e-33

Now we count genes in the upper $1/8^{th}$:

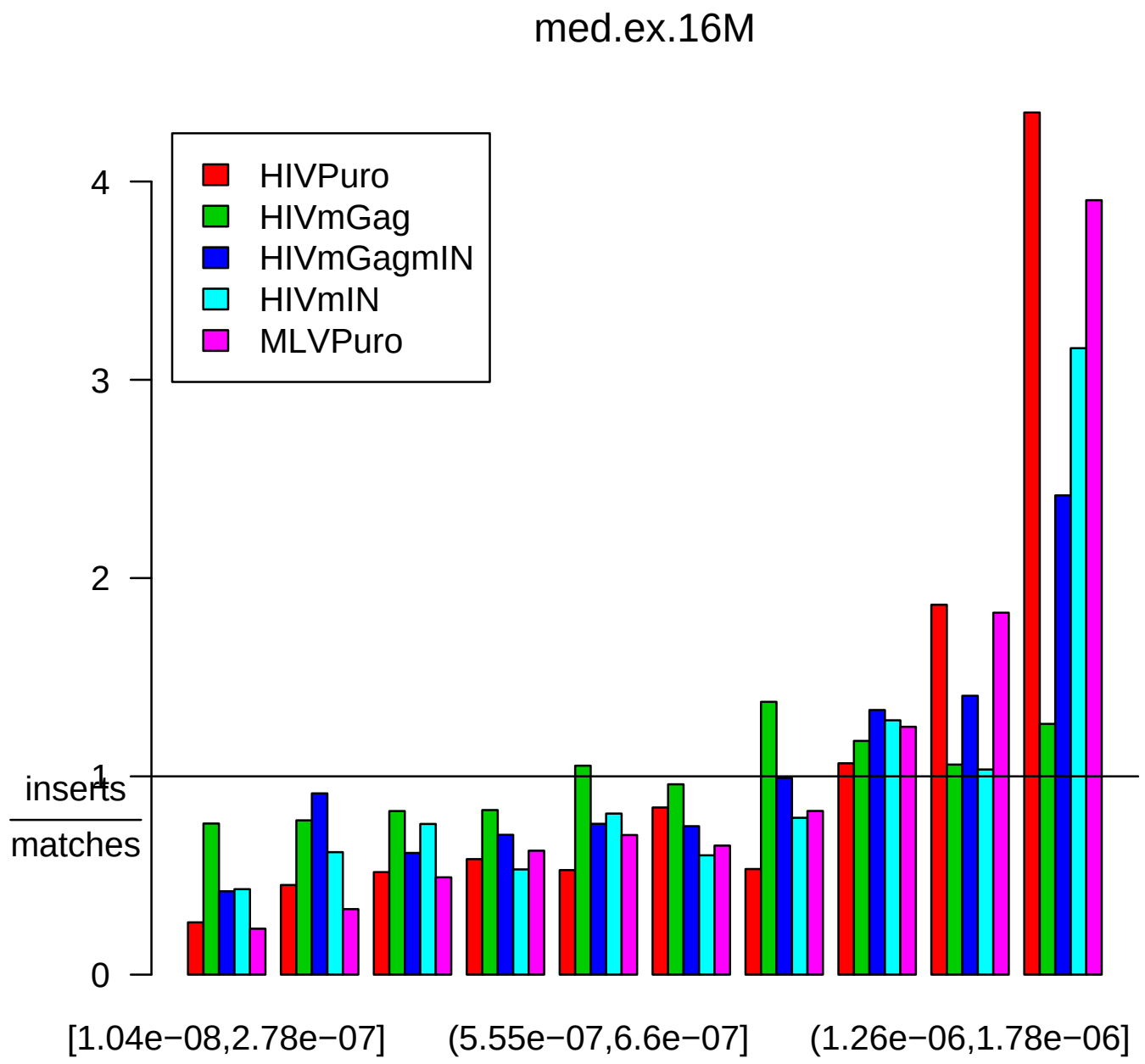

	coef	se	z	p
HIVPuro	1.210	0.1050	11.50	1.10e-30
HIVmGag	0.321	0.0954	3.37	7.60e-04
HIVmGagmIN	0.687	0.0953	7.21	5.74e-13
HIVmIN	0.789	0.1220	6.48	9.21e-11
MLVPuro	1.210	0.1030	11.70	1.85e-31

And here we count genes in the upper $1/16^{th}$:

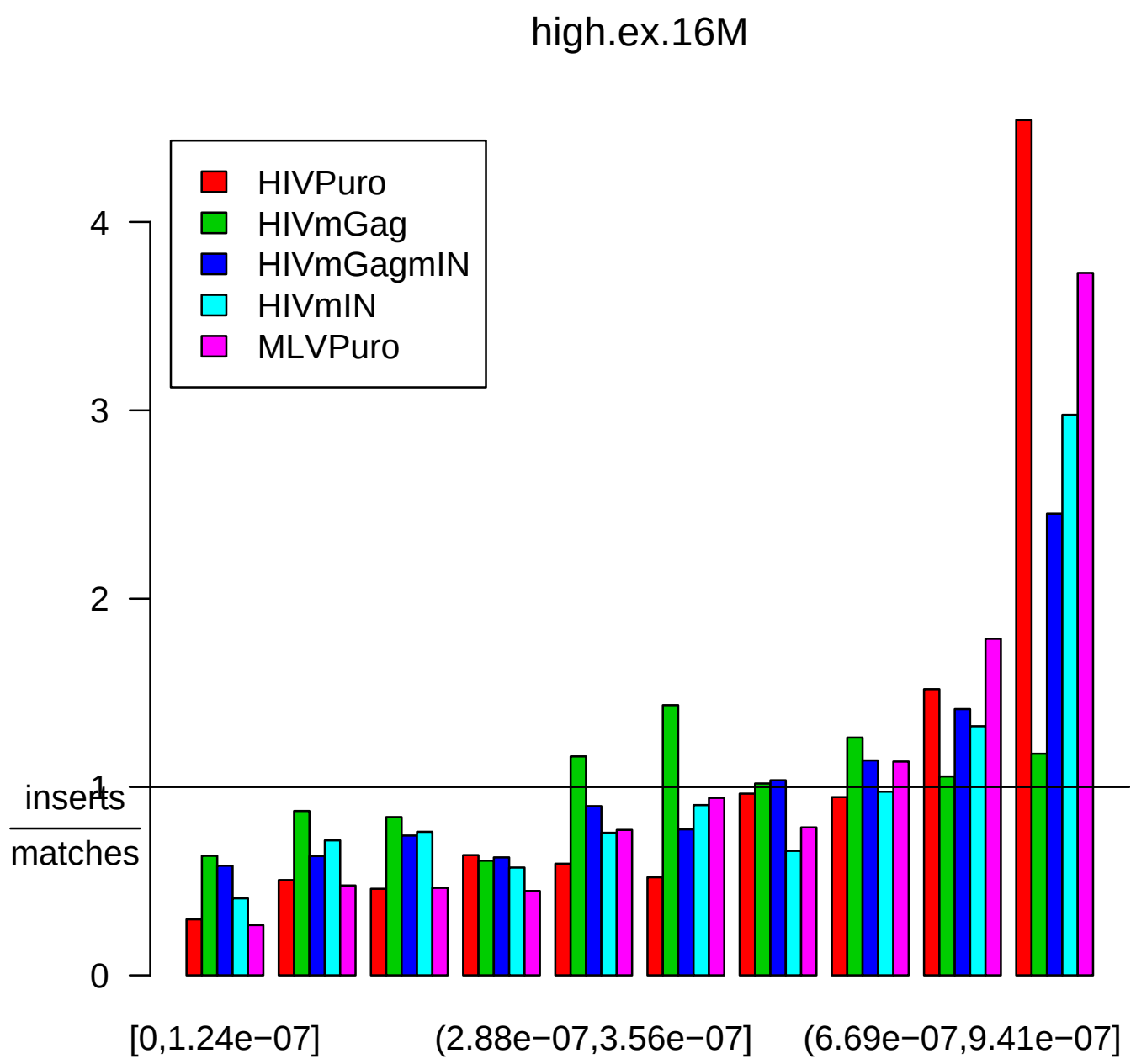

	coef	se	z	p
HIVPuro	1.130	0.1030	10.90	9.70e-28
HIVmGag	0.367	0.0955	3.85	1.19e-04
HIVmGagmIN	0.655	0.0952	6.88	5.99e-12
HIVmIN	0.772	0.1210	6.37	1.94e-10
MLVPuro	1.180	0.1020	11.50	1.14e-30

Here the effect of density of CpG islands is studied:

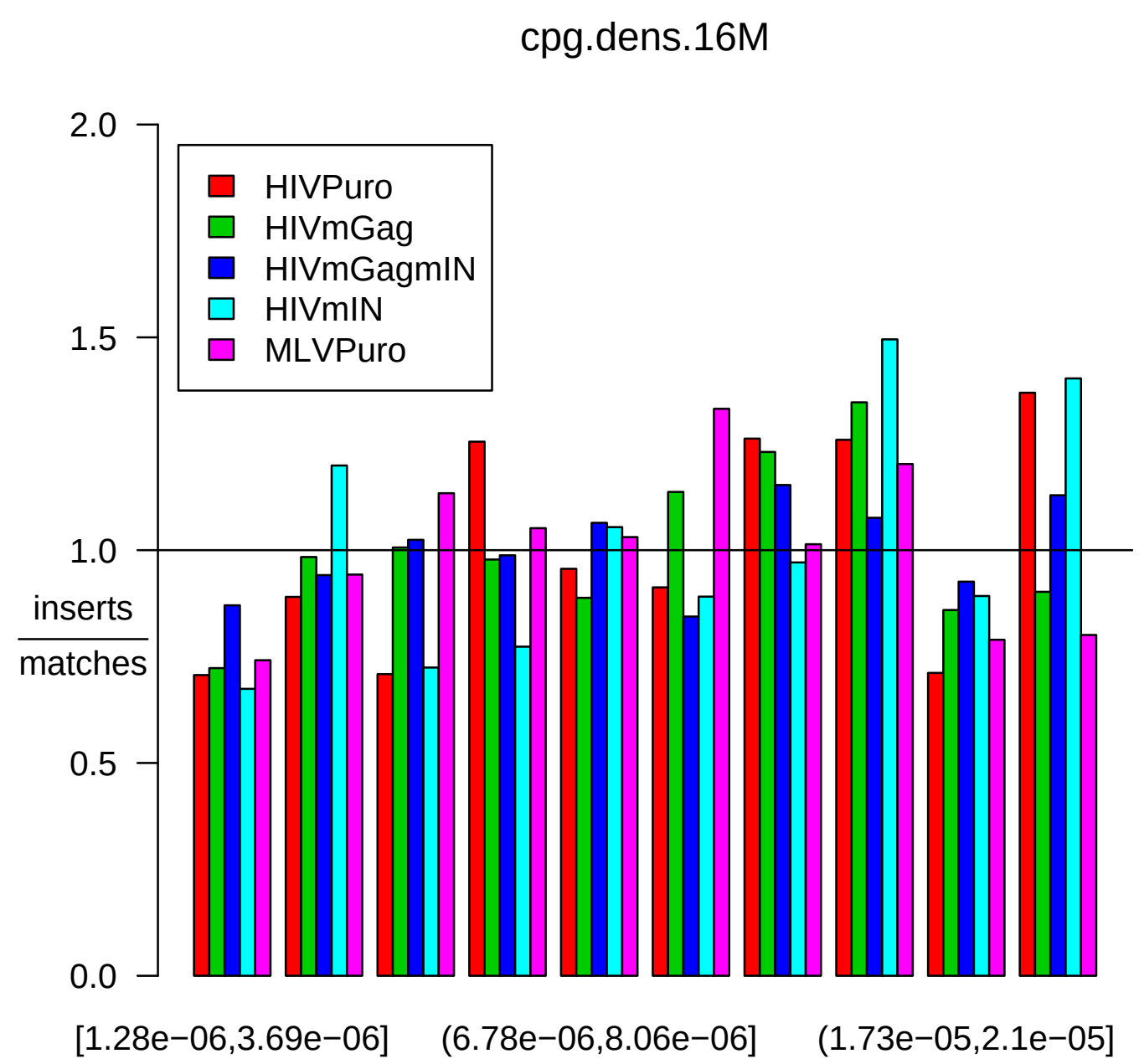

	coef	se	z	p
HIVPuro	0.1990	0.0914	2.180	0.0293
HIVmGag	0.1710	0.0945	1.810	0.0697
HIVmGagmIN	0.0464	0.0915	0.507	0.6120
HIVmIN	0.2440	0.1130	2.160	0.0304
MLVPuro	0.0464	0.0900	0.515	0.6060

4.11 32 megabase Window

In the barplot that follows we examine the association of insertion sites with expression density in a 32 megabase window surrounding each locus. First, we count just the number of genes represented on the chip.

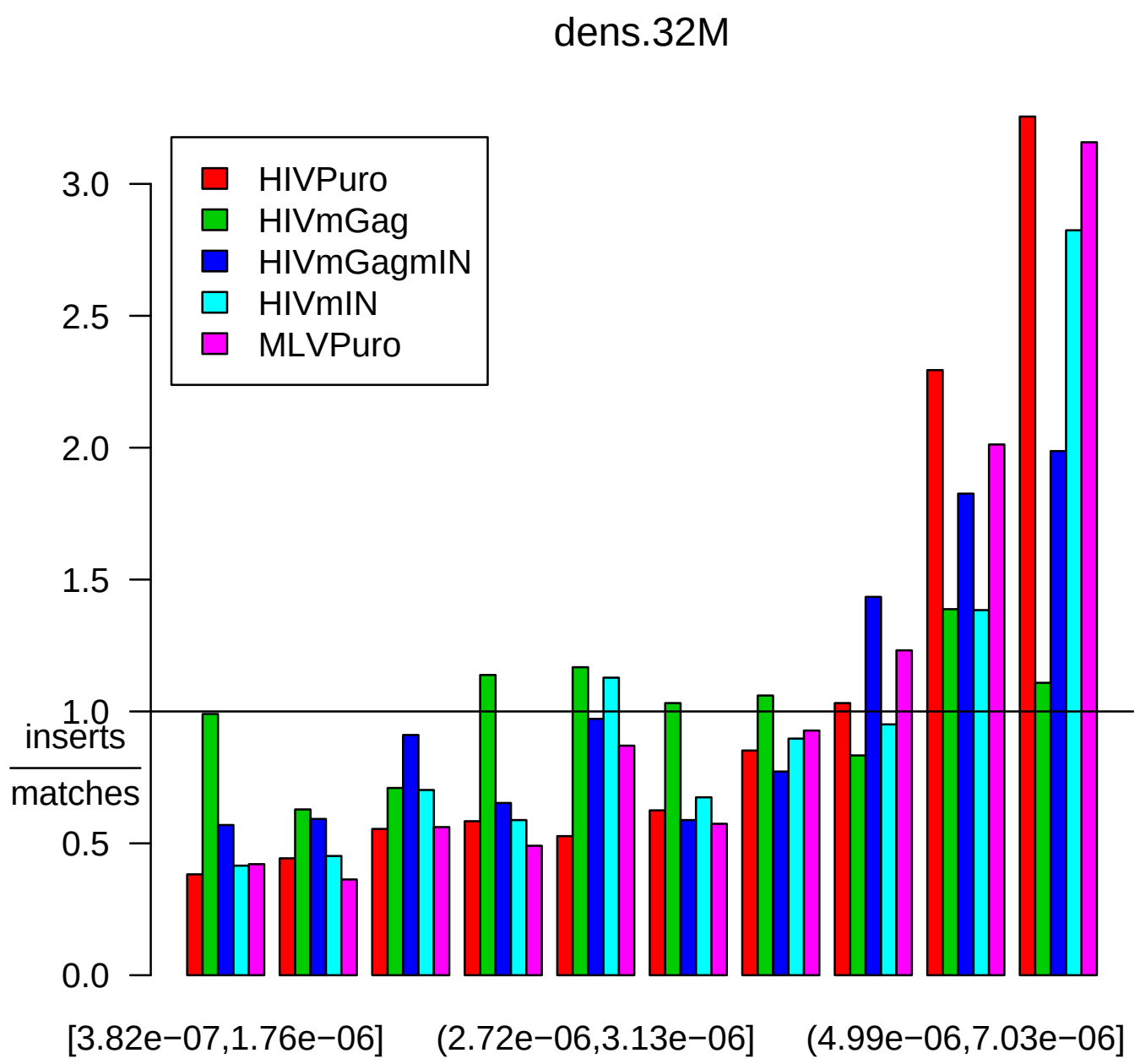

	coef	se	z	p
HIVPuro	1.130	0.1030	10.90	1.05e-27
HIVmGag	0.159	0.0945	1.68	9.31e-02
HIVmGagmIN	0.559	0.0943	5.93	3.08e-09
HIVmIN	0.724	0.1200	6.05	1.43e-09
MLVPuro	1.010	0.0984	10.30	7.18e-25

Here are the results for expression density. First, we count just genes that are in the upper half.

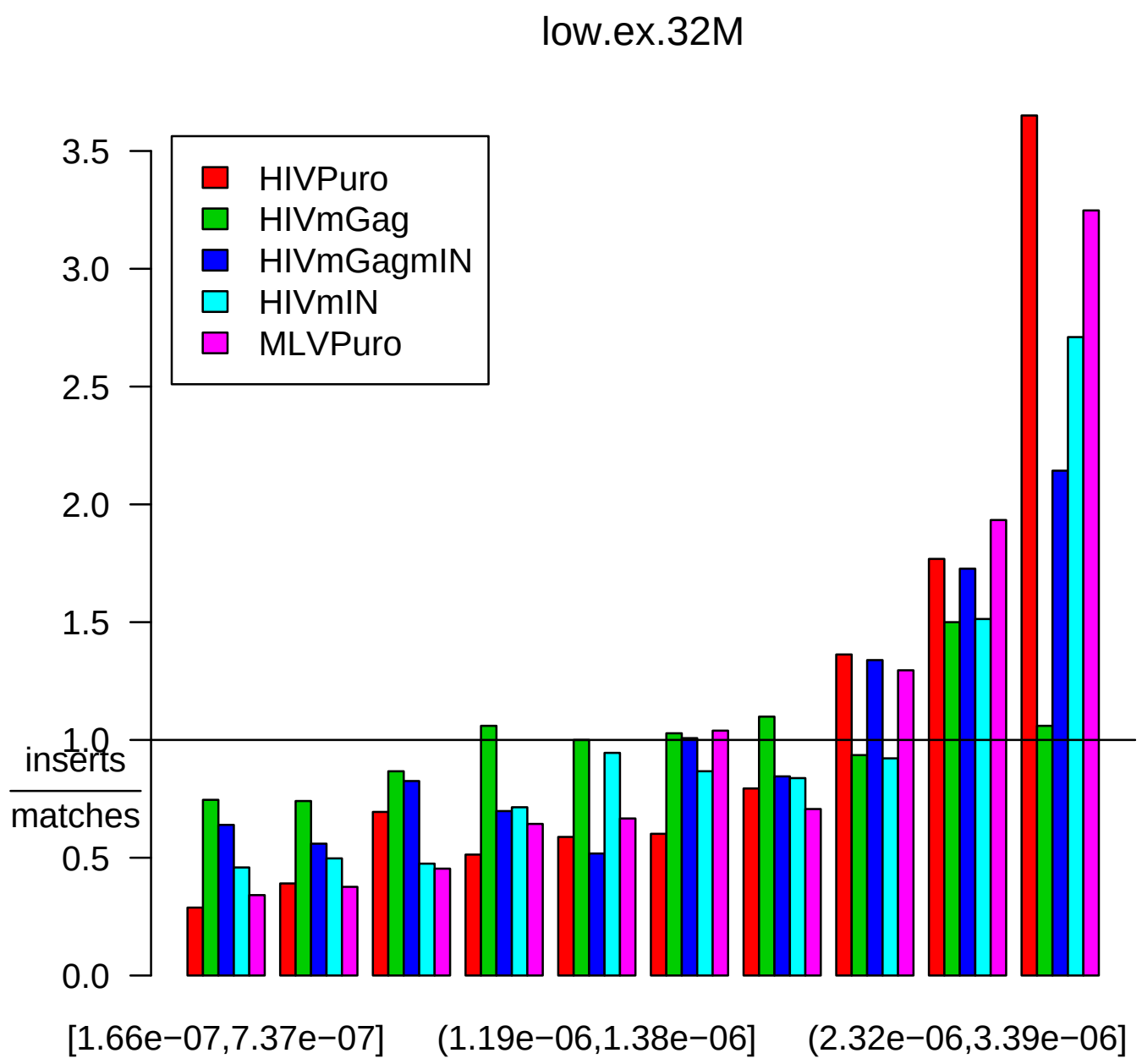

	coef	se	z	p
HIVPuro	1.140	0.1030	11.10	1.75e-28
HIVmGag	0.246	0.0950	2.59	9.59e-03
HIVmGagmIN	0.764	0.0965	7.92	2.37e-15
HIVmIN	0.816	0.1220	6.72	1.88e-11
MLVPuro	1.140	0.1020	11.20	3.49e-29

Now we count genes in the upper $1/8^{th}$:

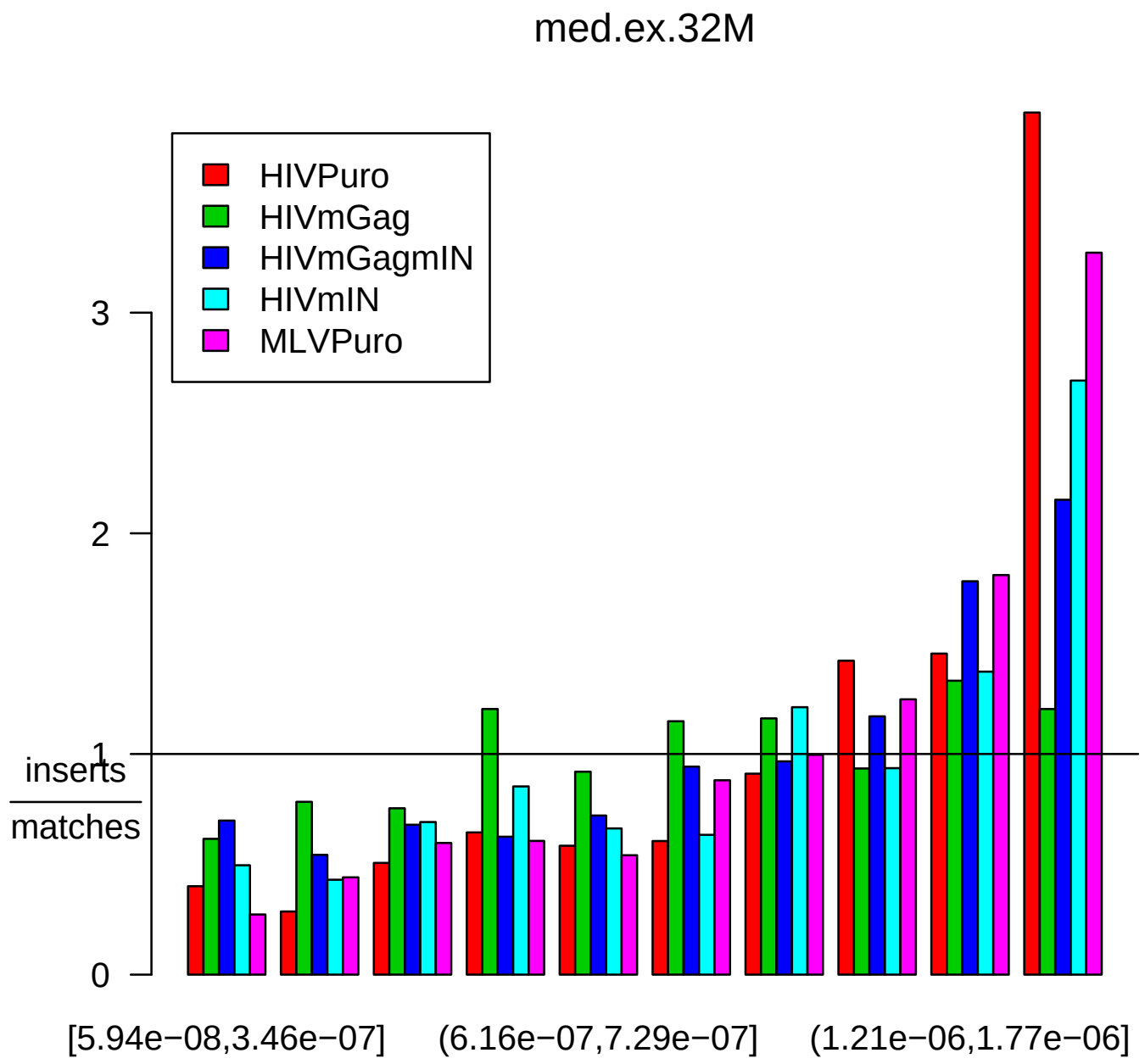

	coef	se	z	p
HIVPuro	1.170	0.1040	11.30	1.03e-29
HIVmGag	0.299	0.0950	3.15	1.63e-03
HIVmGagmIN	0.746	0.0962	7.75	9.25e-15
HIVmIN	0.801	0.1220	6.58	4.63e-11
MLVPuro	1.170	0.1030	11.40	5.40e-30

And here we count genes in the upper $1/16^{th}$:

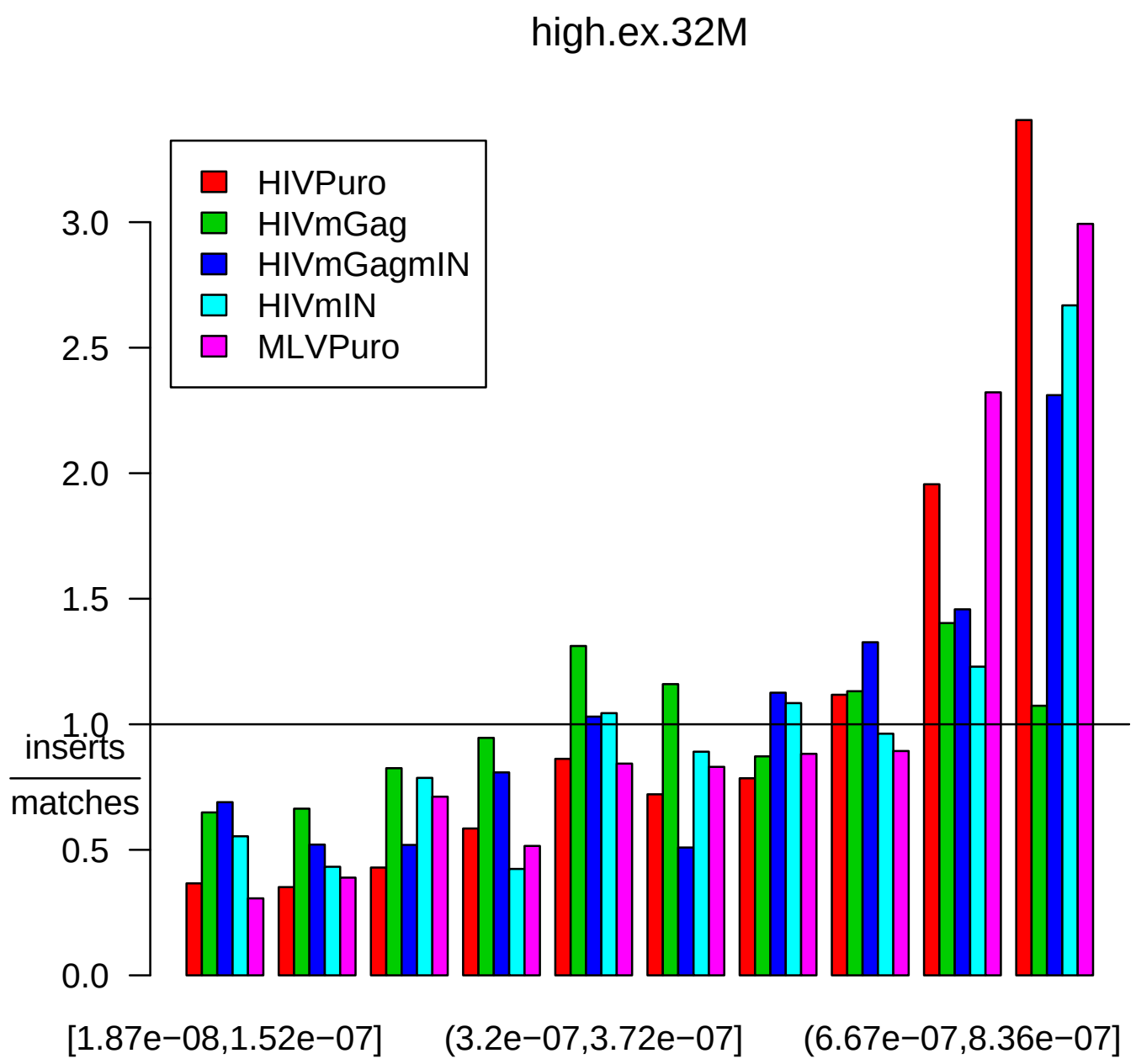

	coef	se	z	p
HIVPuro	1.090	0.1030	10.70	1.66e-26
HIVmGag	0.259	0.0950	2.72	6.47e-03
HIVmGagmIN	0.615	0.0946	6.50	8.16e-11
HIVmIN	0.762	0.1200	6.33	2.51e-10
MLVPuro	1.010	0.0986	10.20	1.28e-24

Here the effect of density of CpG islands is studied:

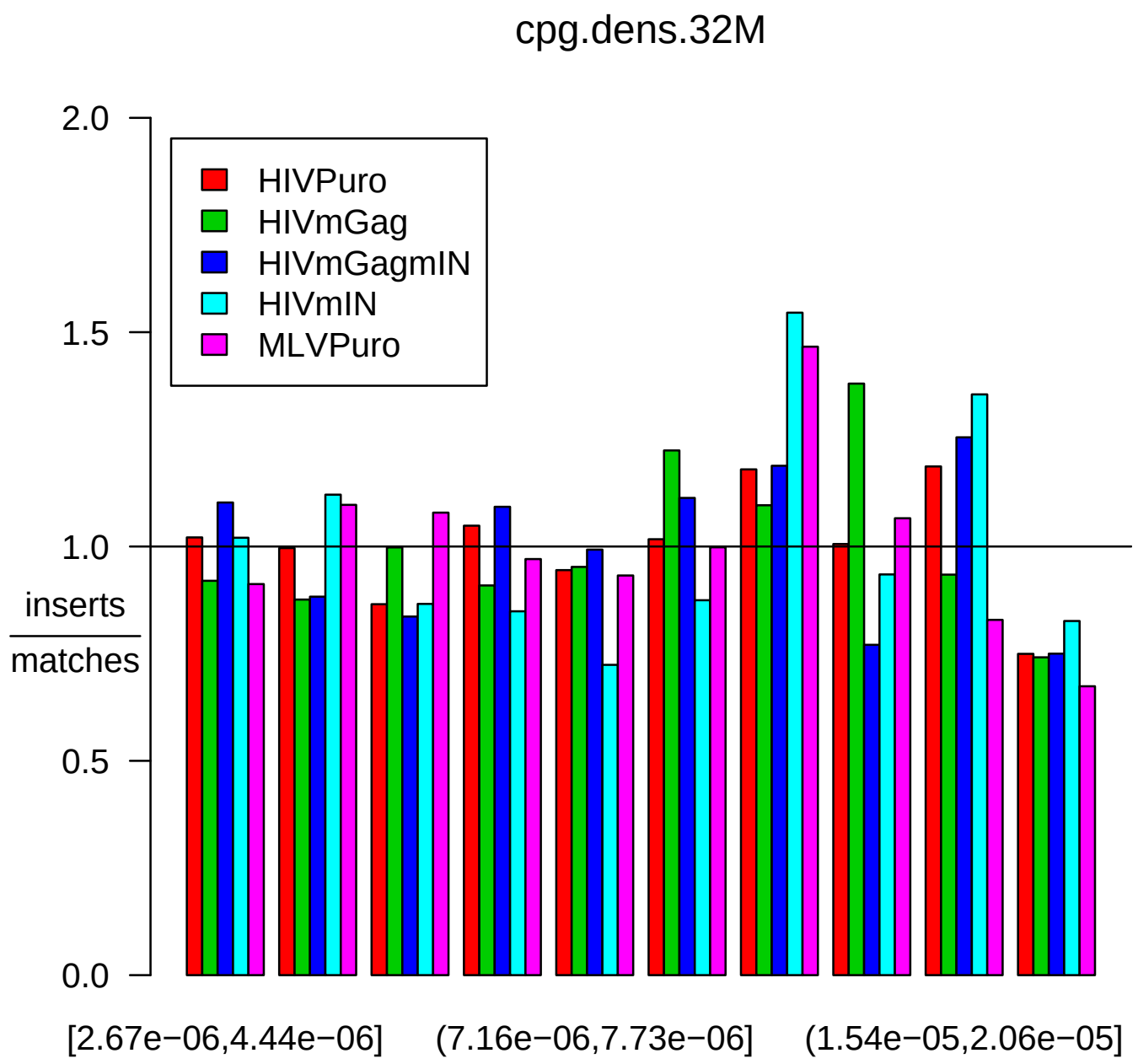

	coef	se	z	p
HIVPuro	0.04850	0.0913	0.5310	0.595
HIVmGag	0.13900	0.0944	1.4700	0.141
HIVmGagmIN	0.03110	0.0914	0.3410	0.733
HIVmIN	0.18500	0.1120	1.6500	0.098
MLVPuro	0.00514	0.0899	0.0572	0.954

5 Juxtaposition with Gene Start and End Positions

5.1 Acembly Annotations

In this section we study the effect of juxtaposition in terms of gene start and end positions. The first barplot shows the effect of gene width for those insertions that are located within an Acembly gene. The table following the barplot shows the p-values for a test of the hypothesis that the proportions in each of the categories that define the bars are equal in the insertions and their matches. This p-value is obtained from the $5 \times 2 \times k$ table of counts defined by gene width category, insertion/match status, and stratum (consisting of an insertion and its matched sites) using a likelihood ratio test for the hypothesis of no association between gene width category and insertion/match status. The test used compared the log-linear model [1] with all two-way configurations to that with no gene width category and insertion/match status configuration.

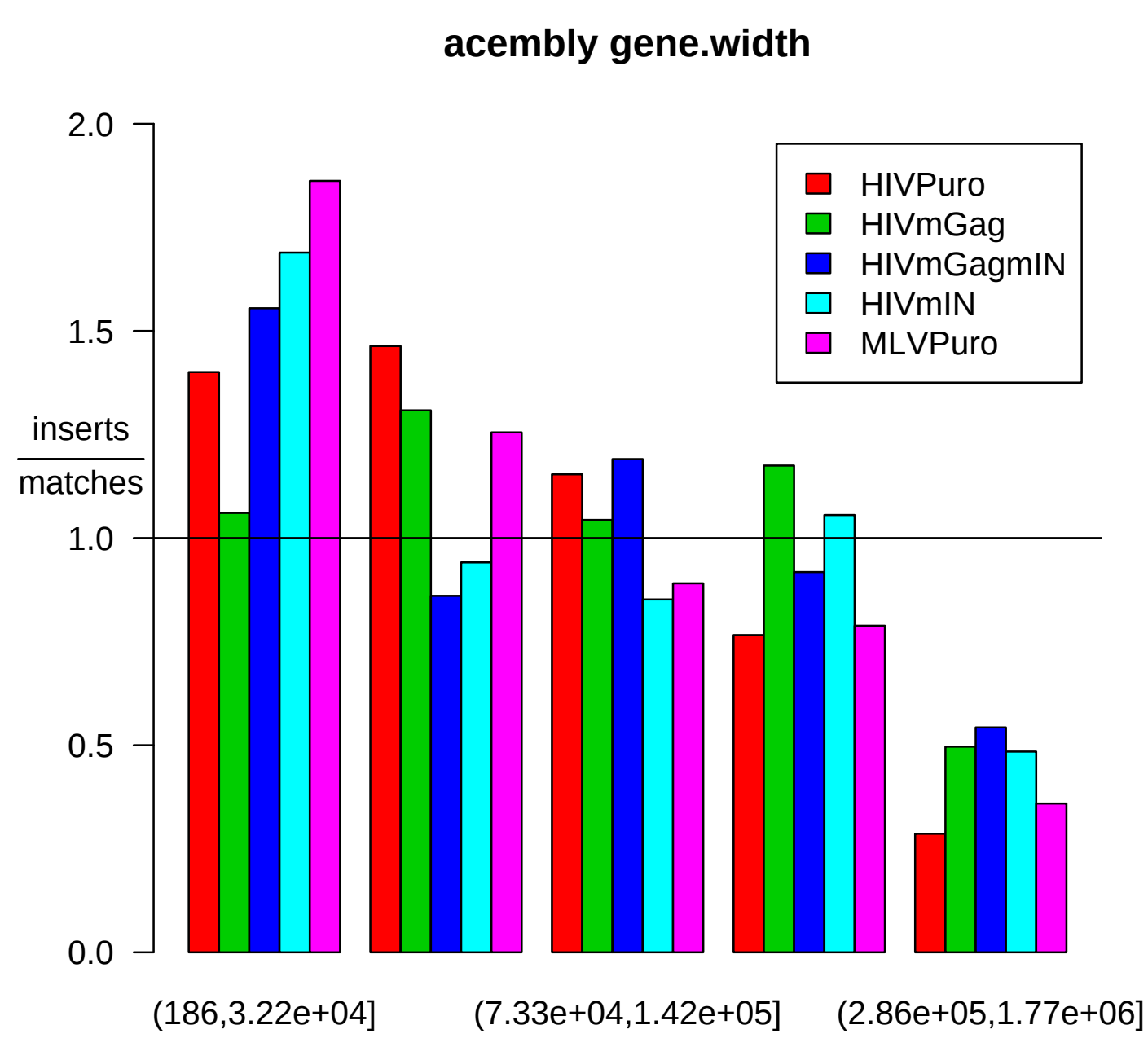

HIVPuro	HIVmGag	HIVmGagmIN	HIVmIN	MLVPuro
6.39e-22	7.70e-07	2.86e-08	2.51e-07	1.72e-19

The next plot uses the width of a non-gene region for insertions that fall into such regions.

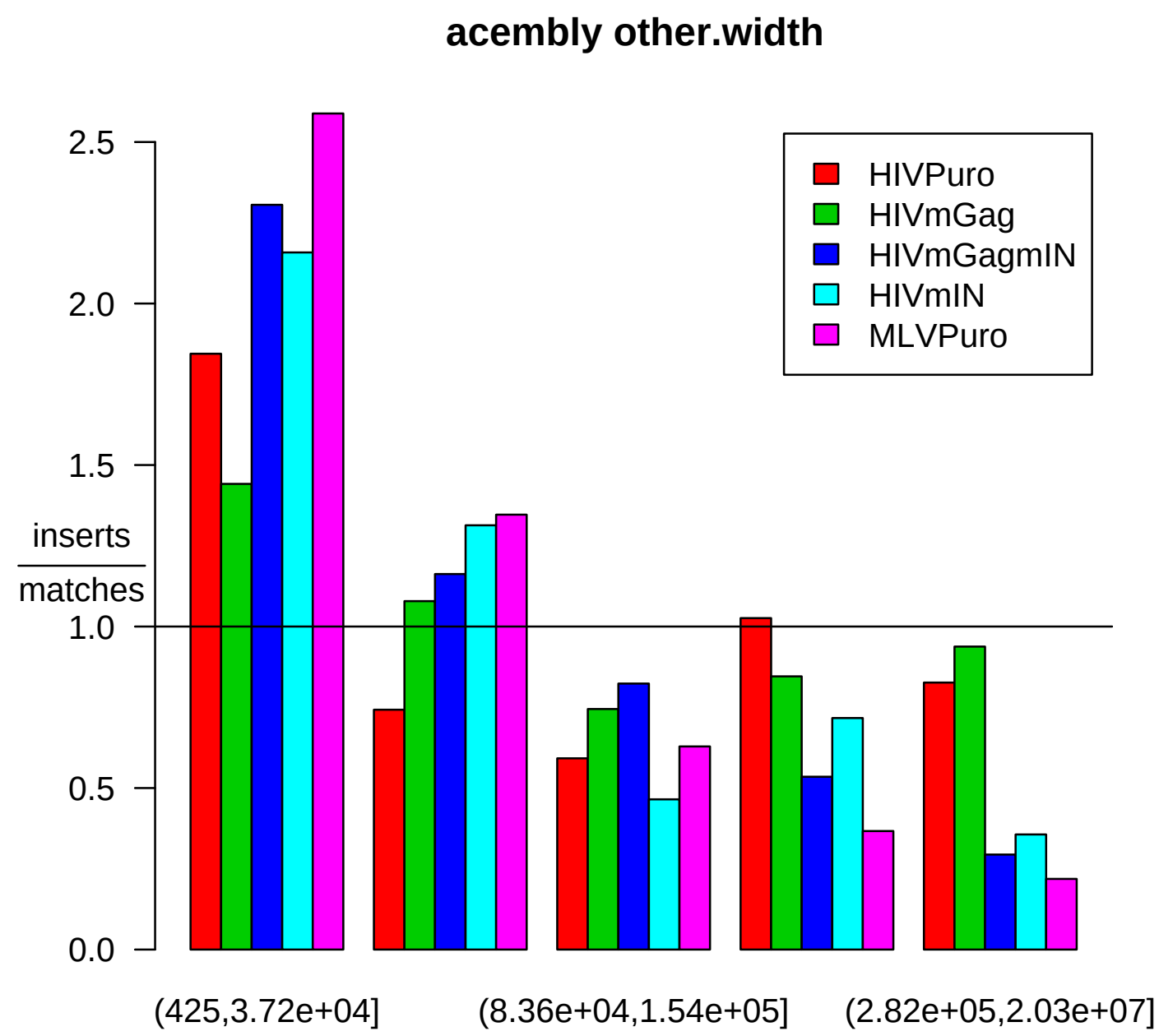


```
  HIVPuro    HIVmGag HIVmGagmIN     HIVmIN    MLVPuro
 9.93e-03   9.06e-02   6.31e-14   3.33e-11   8.01e-24
```

The next plot studies the distance to the nearest boundary between a gene and a non-gene region. The distance is expressed as a fraction of the length of the region. Thus, '0.25' refers to one quarter of the distance from the site to nearest boundary divided by the total width of the region.

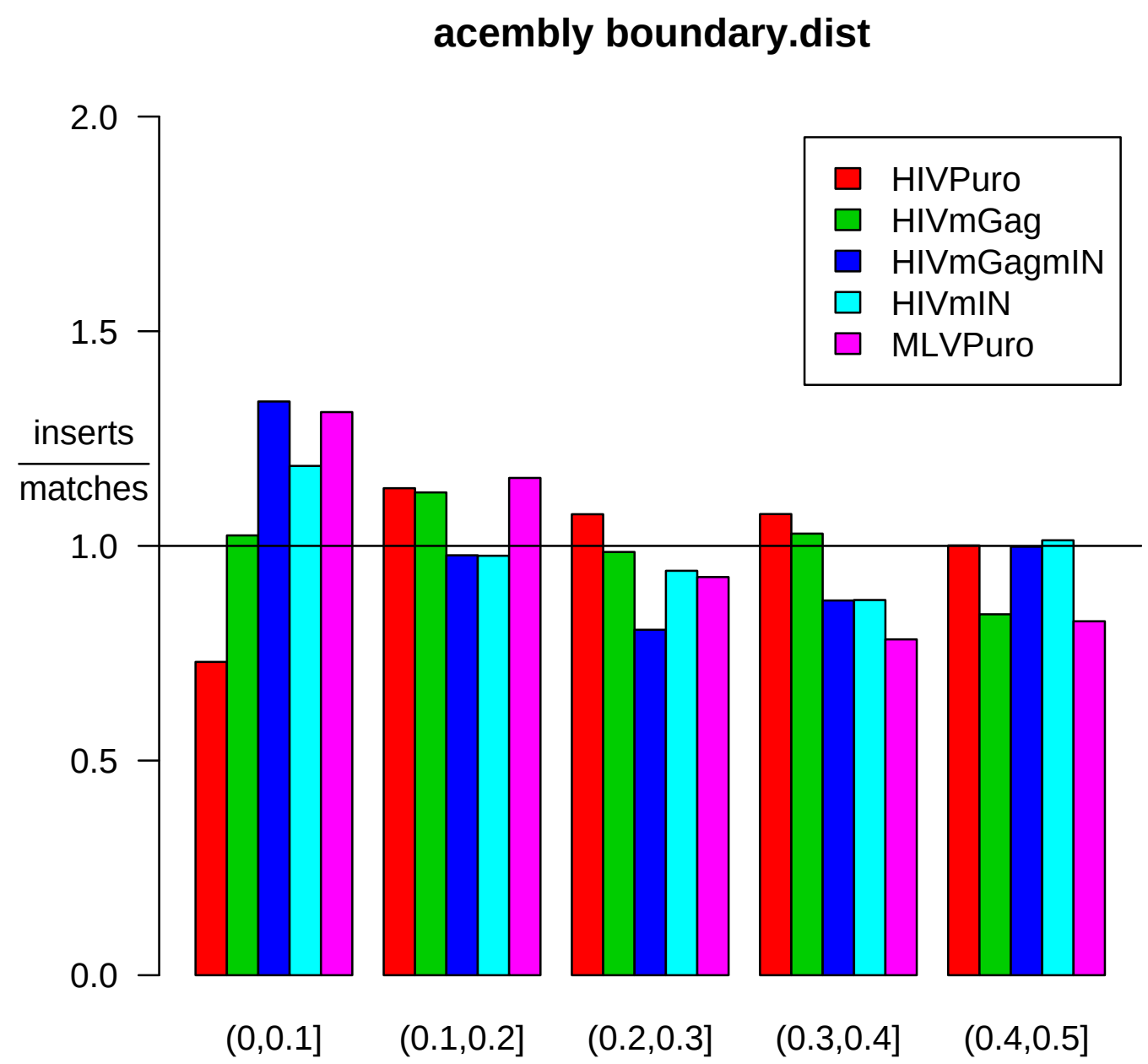

HIVPuro	HIVmGag	HIVmGagmIN	HIVmIN	MLVPuro
0.021900	0.377000	0.001910	0.428000	0.000201

This plot studies the effect of nearness to the beginning of a transcript. For sites in genes, it is the distance to the start of the gene divided by the width of the gene. For other sites it is the distance from the site to the nearer gene if that gene boundary is also a transcription starting point. Locations near '0' are relatively near the beginning of transcription, while those near '1' are near the termination of the transcript.

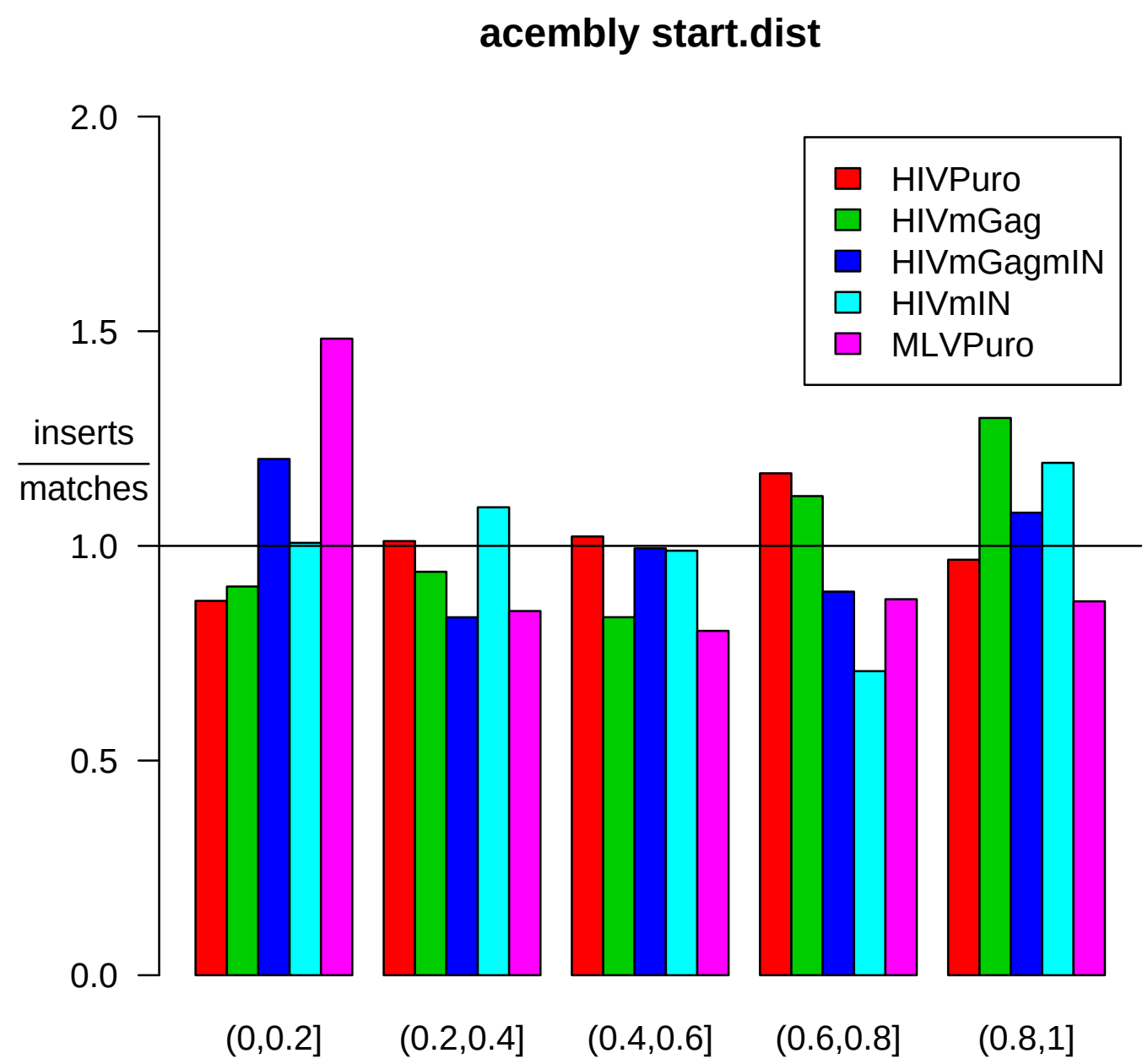

HIVPuro	HIVmGag	HIVmGagmIN	HIVmIN	MLVPuro
3.31e-01	1.94e-02	4.21e-02	5.61e-02	5.26e-07

5.2 RefSeq Annotations

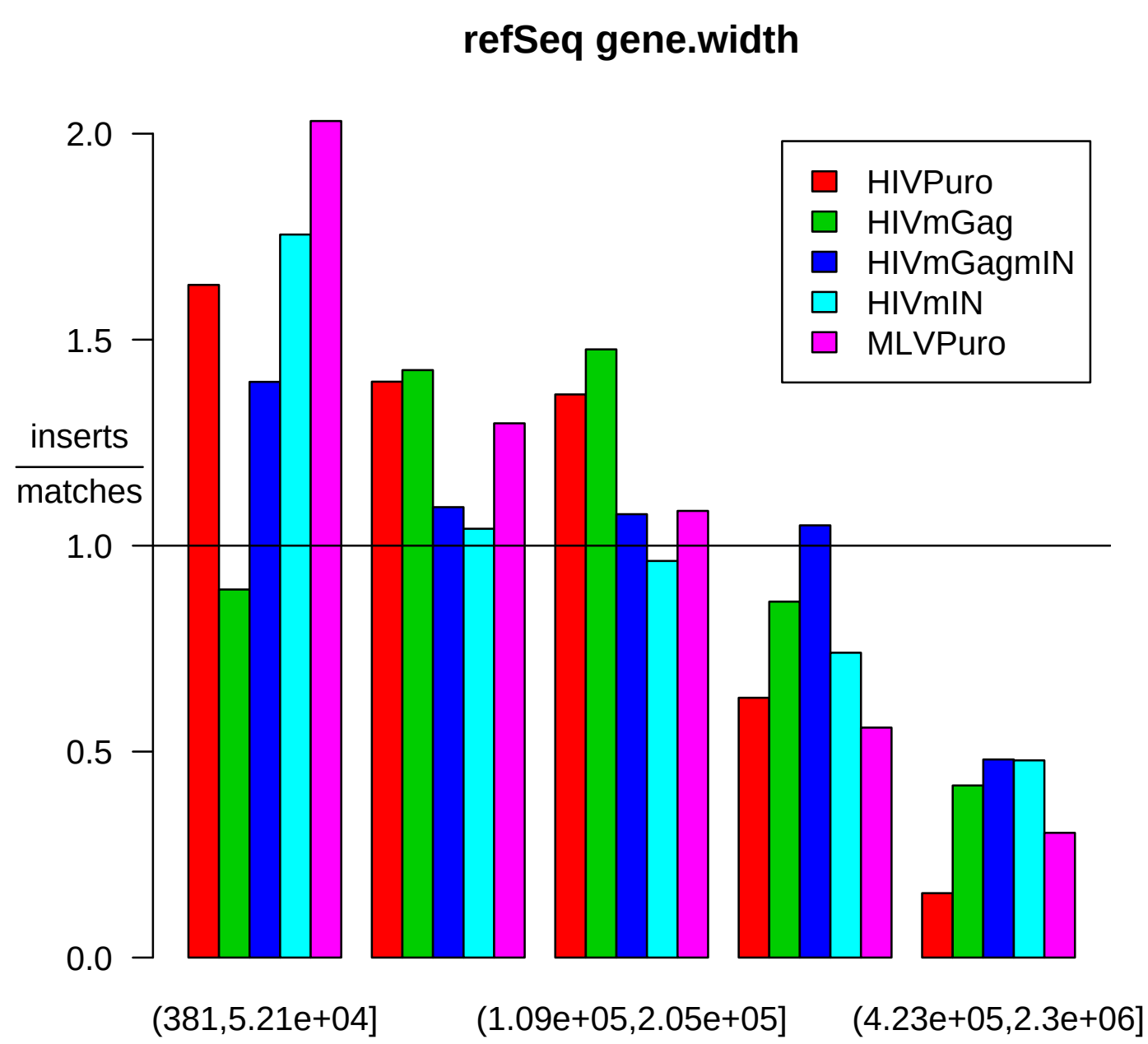


```
  HIVPuro     HIVmGag HIVmGagmIN      HIVmIN     MLVPuro
8.21e-35    1.21e-11    2.02e-05    3.96e-05    4.26e-16
```

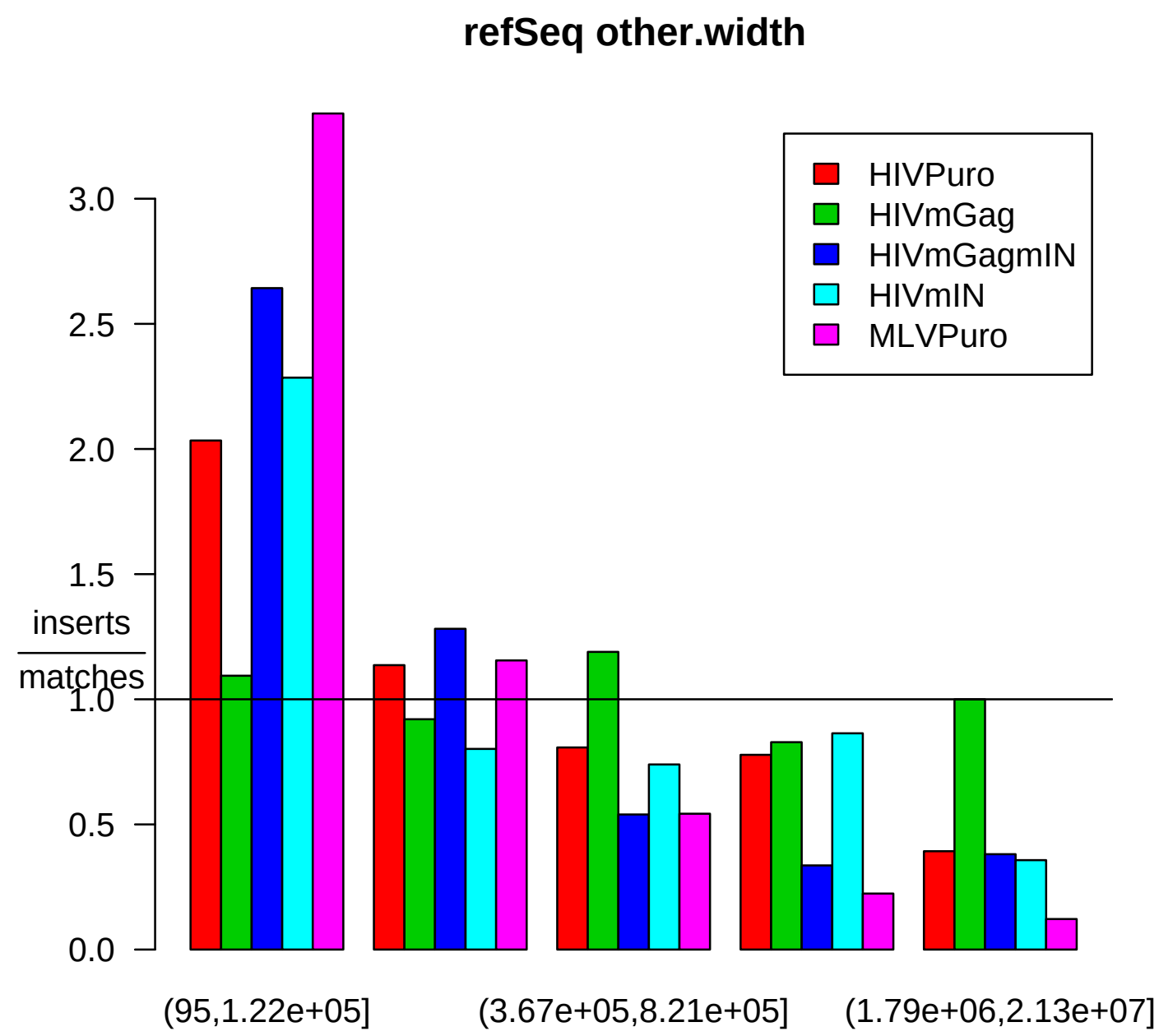


```
  HIVPuro     HIVmGag HIVmGagmIN      HIVmIN     MLVPuro
2.96e-04    6.04e-01    3.93e-37    5.50e-16    1.50e-74
```

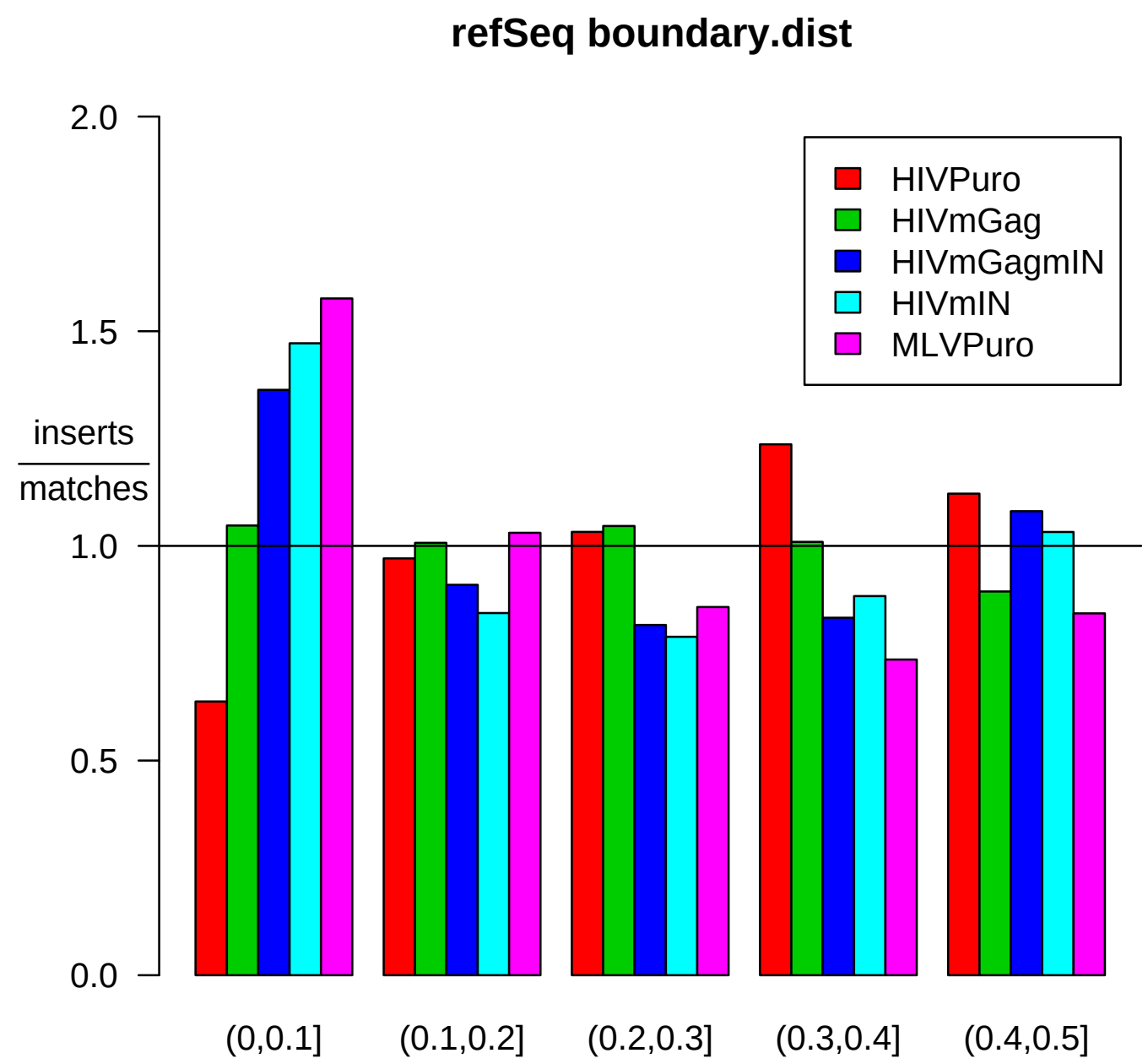

HIVPuro	HIVmGag	HIVmGagmIN	HIVmIN	MLVPuro
1.75e-04	8.14e-01	3.35e-04	6.19e-04	7.85e-09

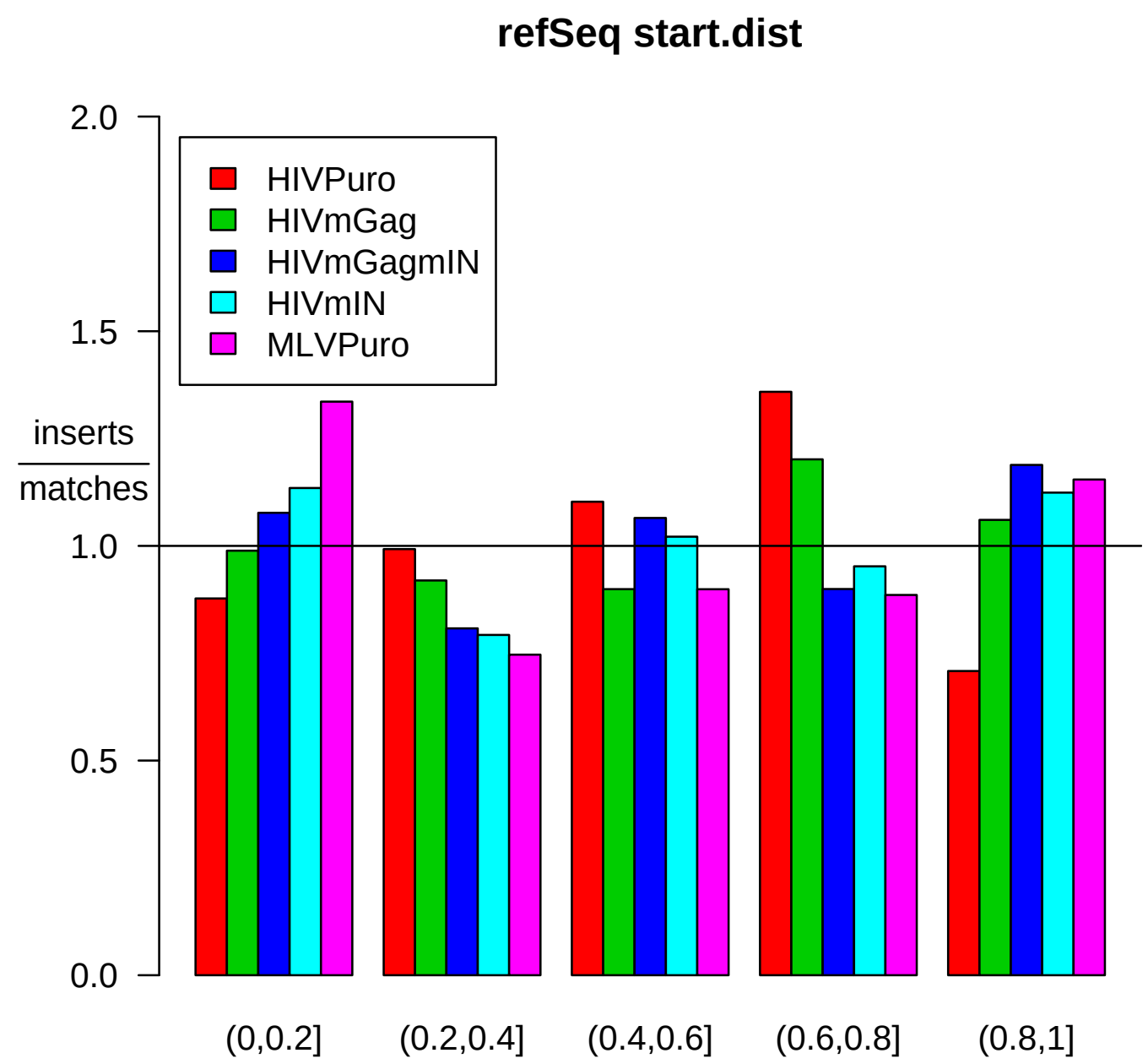

HIVPuro	HIVmGag	HIVmGagmIN	HIVmIN	MLVPuro
0.000393	0.413000	0.160000	0.248000	0.000952

5.3 genScan Annotations

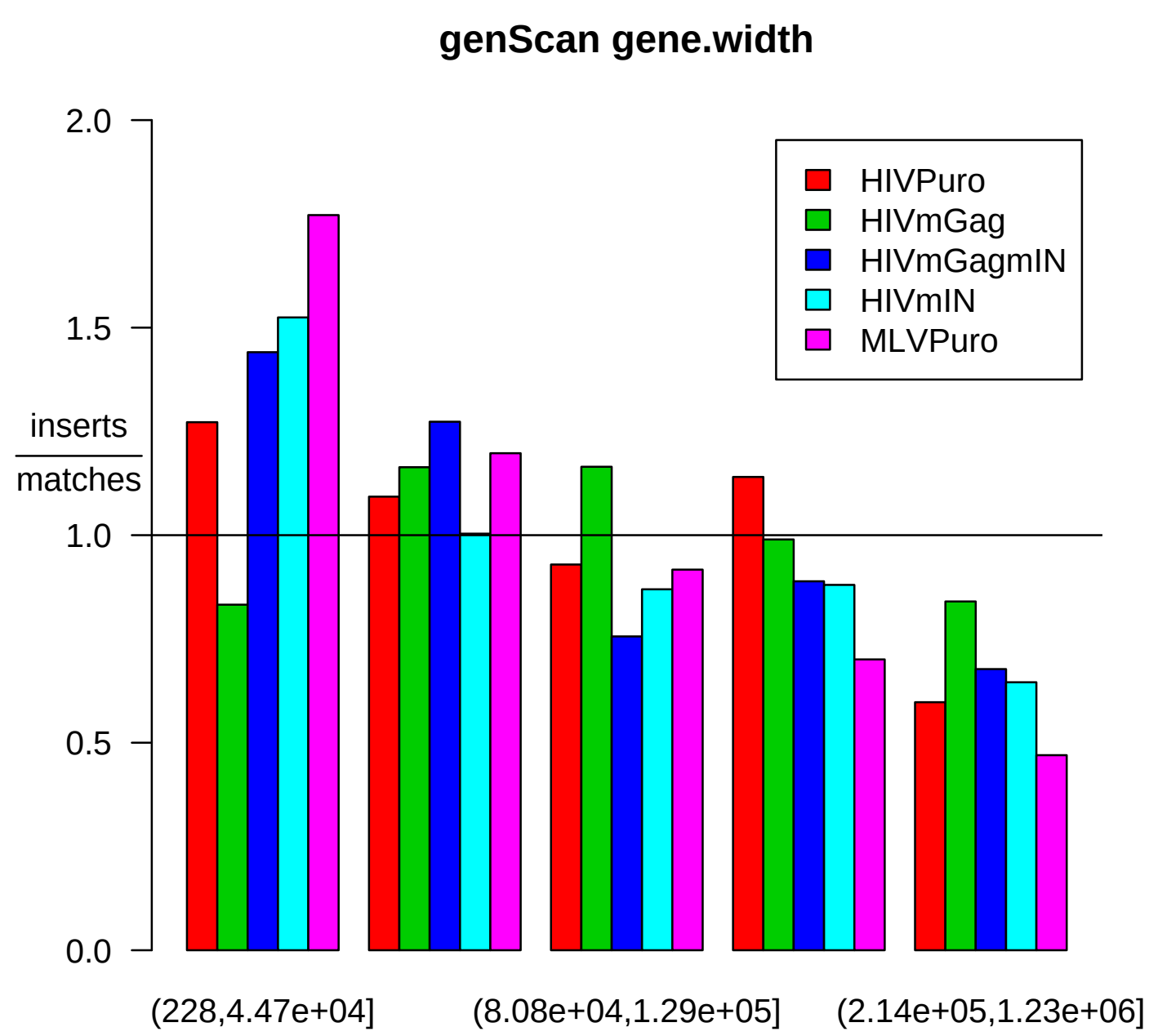


```
   HIVPuro     HIVmGag HIVmGagmIN      HIVmIN     MLVPuro
  1.24e-04    9.03e-02    3.97e-06    3.05e-04    2.48e-13
```

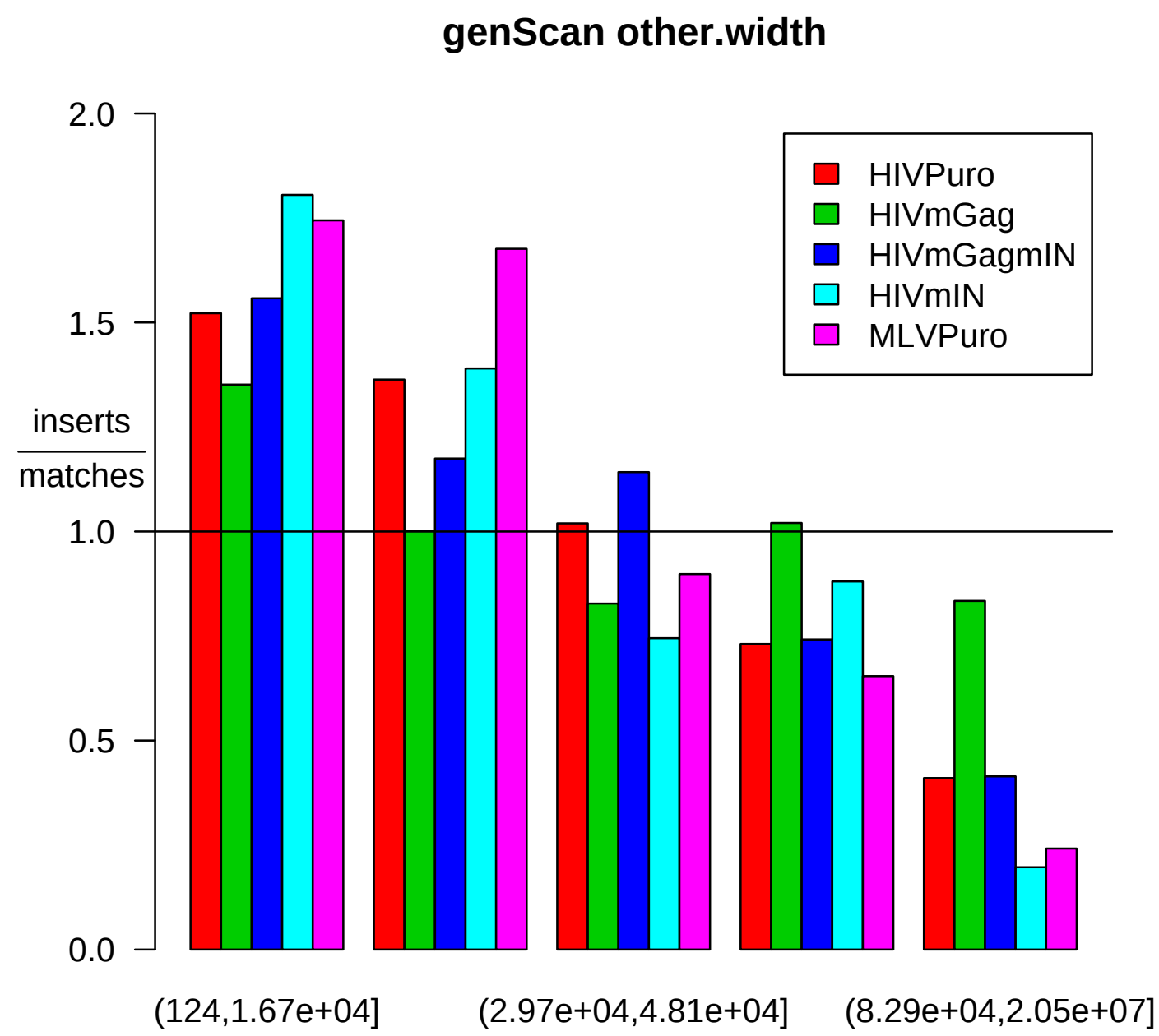

HIVPuro	HIVmGag	HIVmGagmIN	HIVmIN	MLVPuro
4.65e-02	4.61e-01	1.43e-05	2.24e-11	5.23e-15

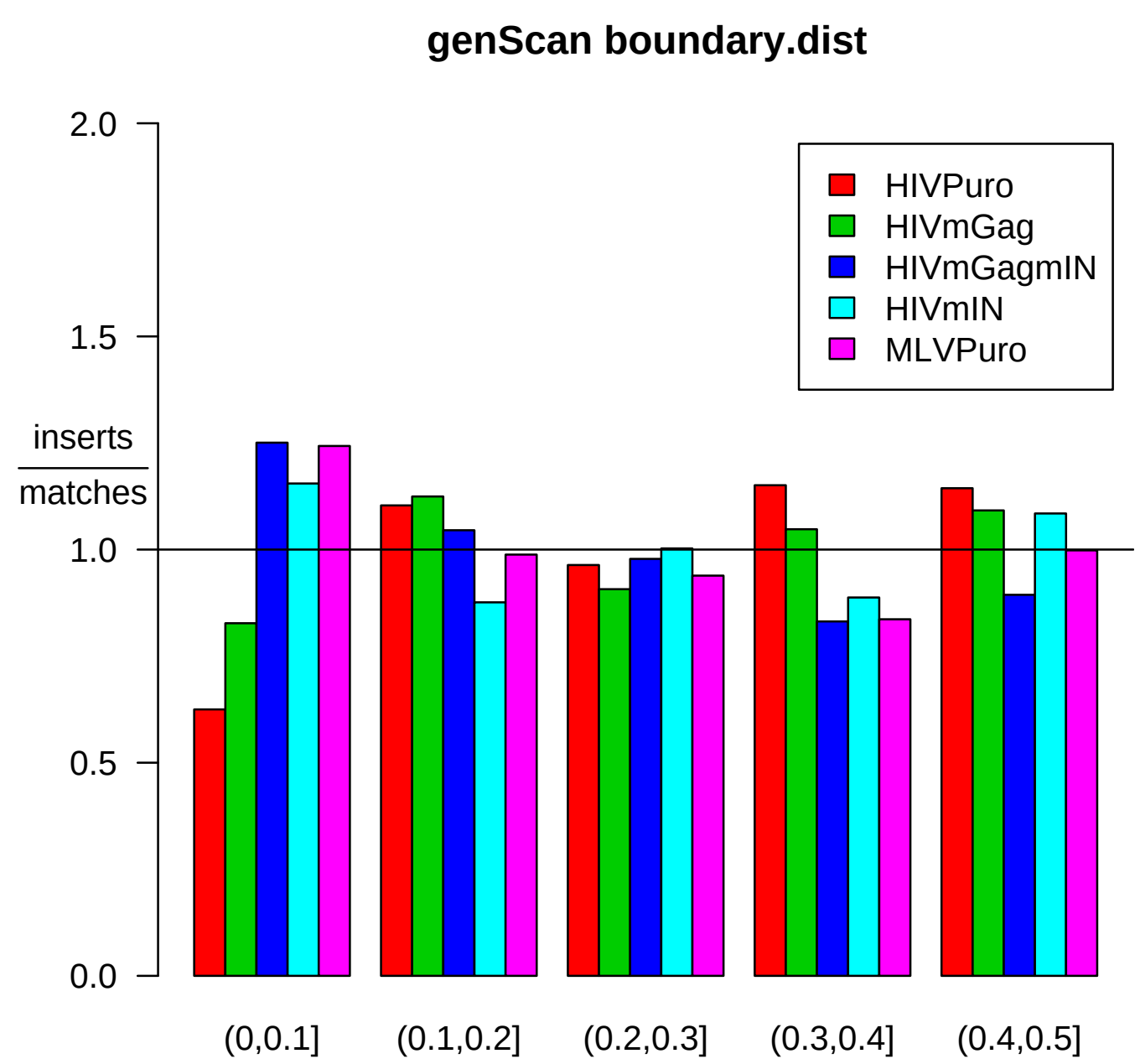

HIVPuro	HIVmGag	HIVmGagmIN	HIVmIN	MLVPuro
0.000154	0.167000	0.029900	0.396000	0.053300

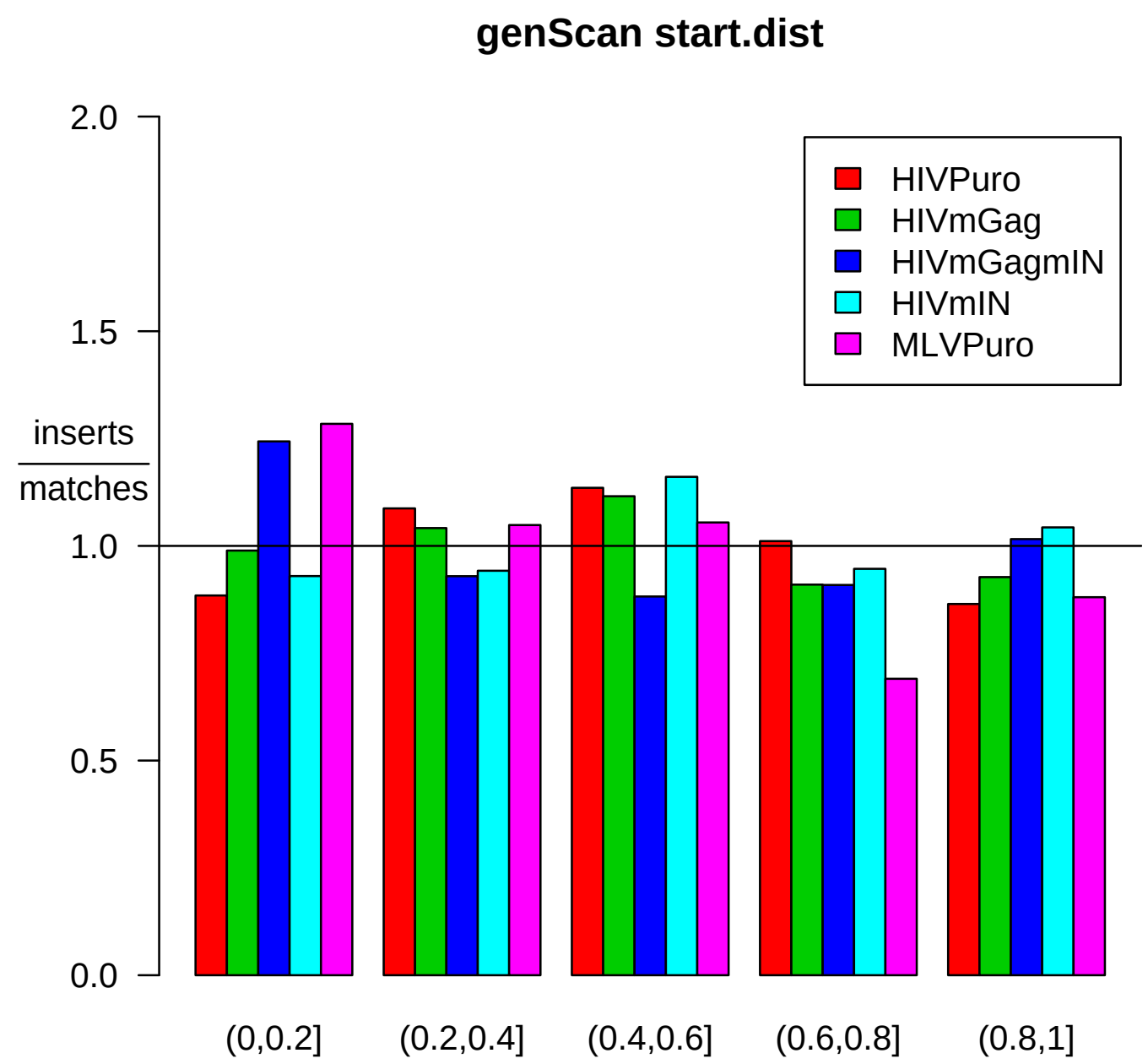

HIVPuro	HIVmGag	HIVmGagmIN	HIVmIN	MLVPuro
0.262000	0.636000	0.098200	0.656000	0.000701

5.4 uniGene Annotations

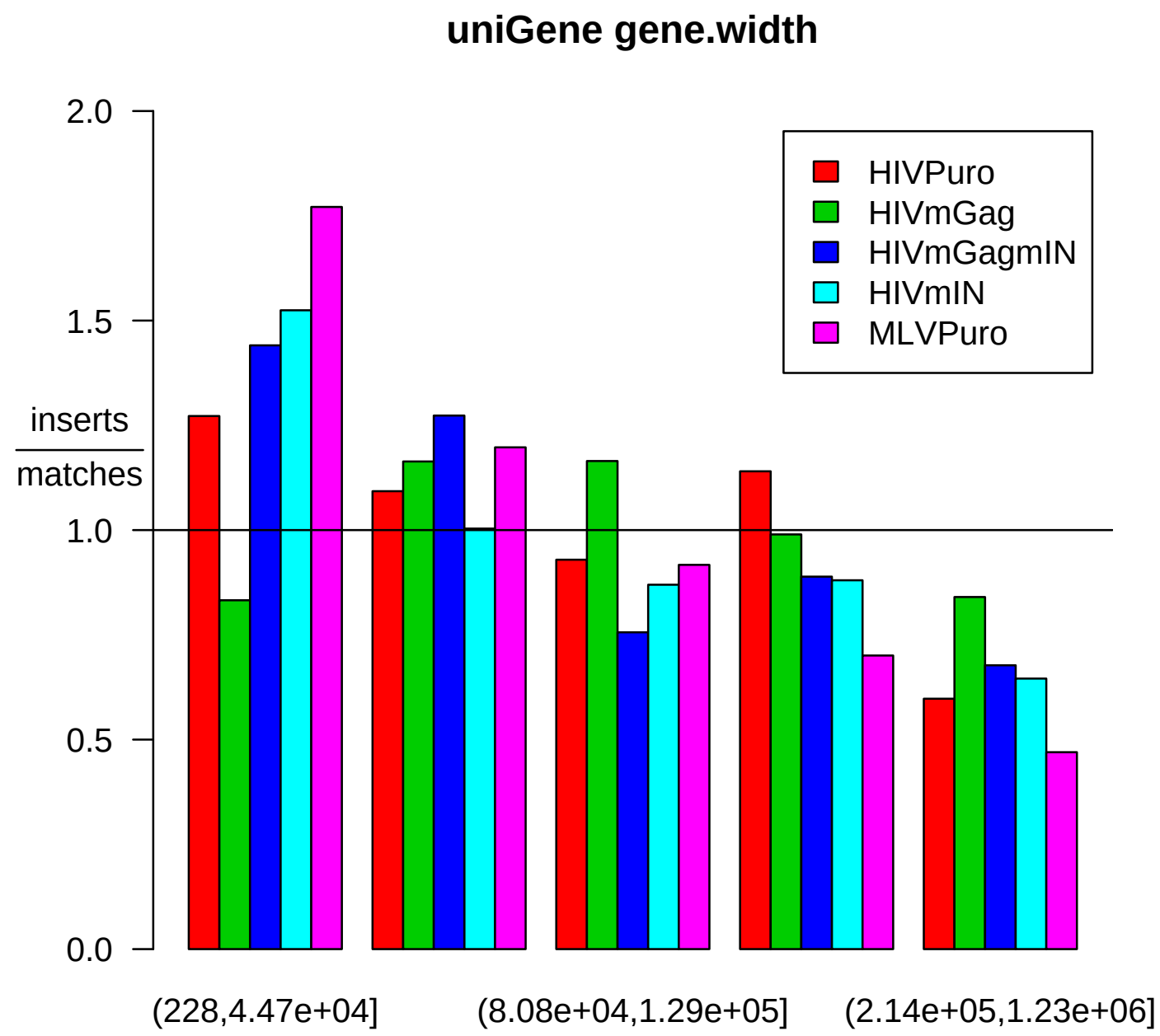

HIVPuro	HIVmGag	HIVmGagmIN	HIVmIN	MLVPuro
1.24e-04	9.03e-02	3.97e-06	3.05e-04	2.48e-13

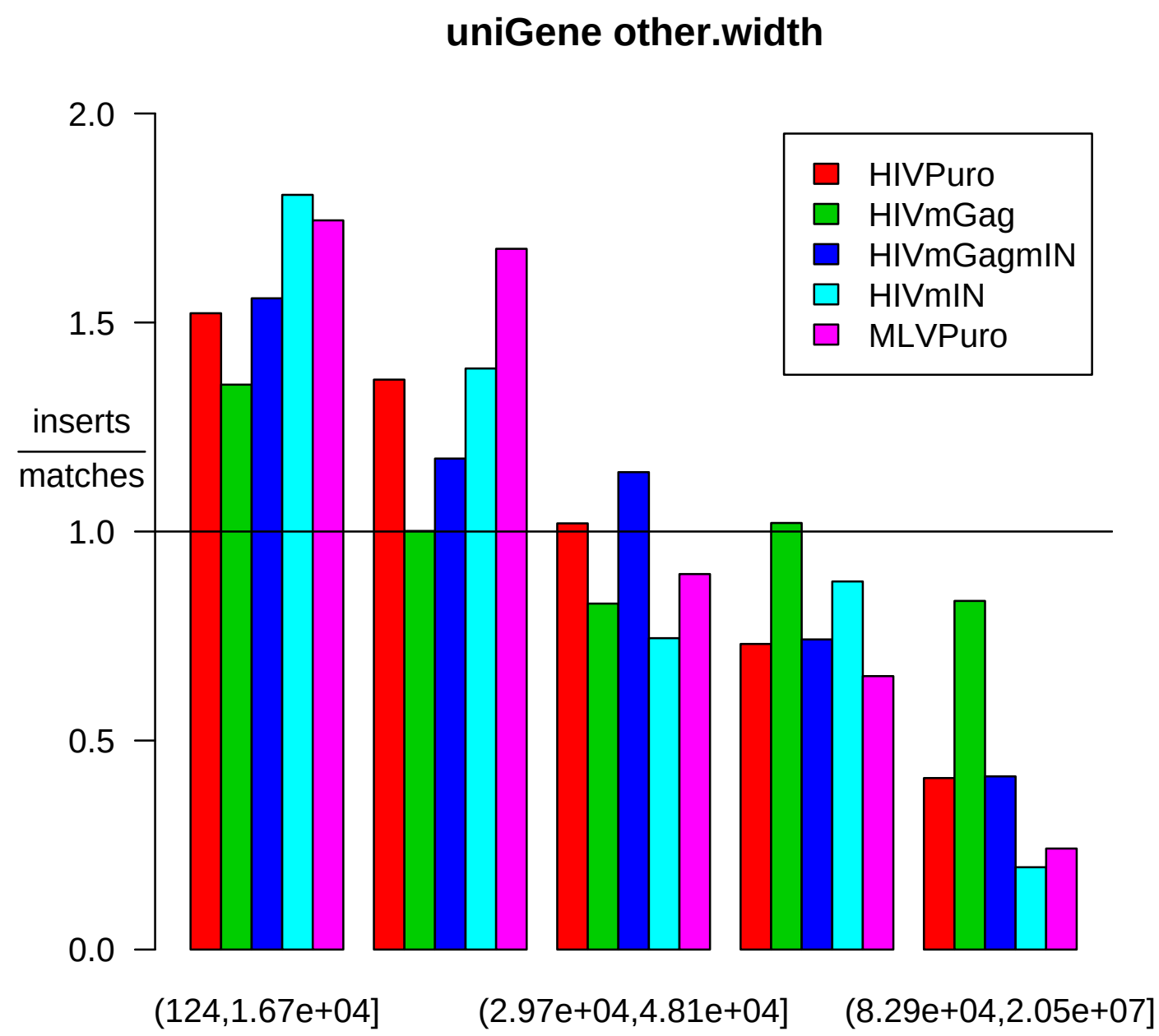

HIVPuro	HIVmGag	HIVmGagmIN	HIVmIN	MLVPuro
4.65e-02	4.61e-01	1.43e-05	2.24e-11	5.23e-15

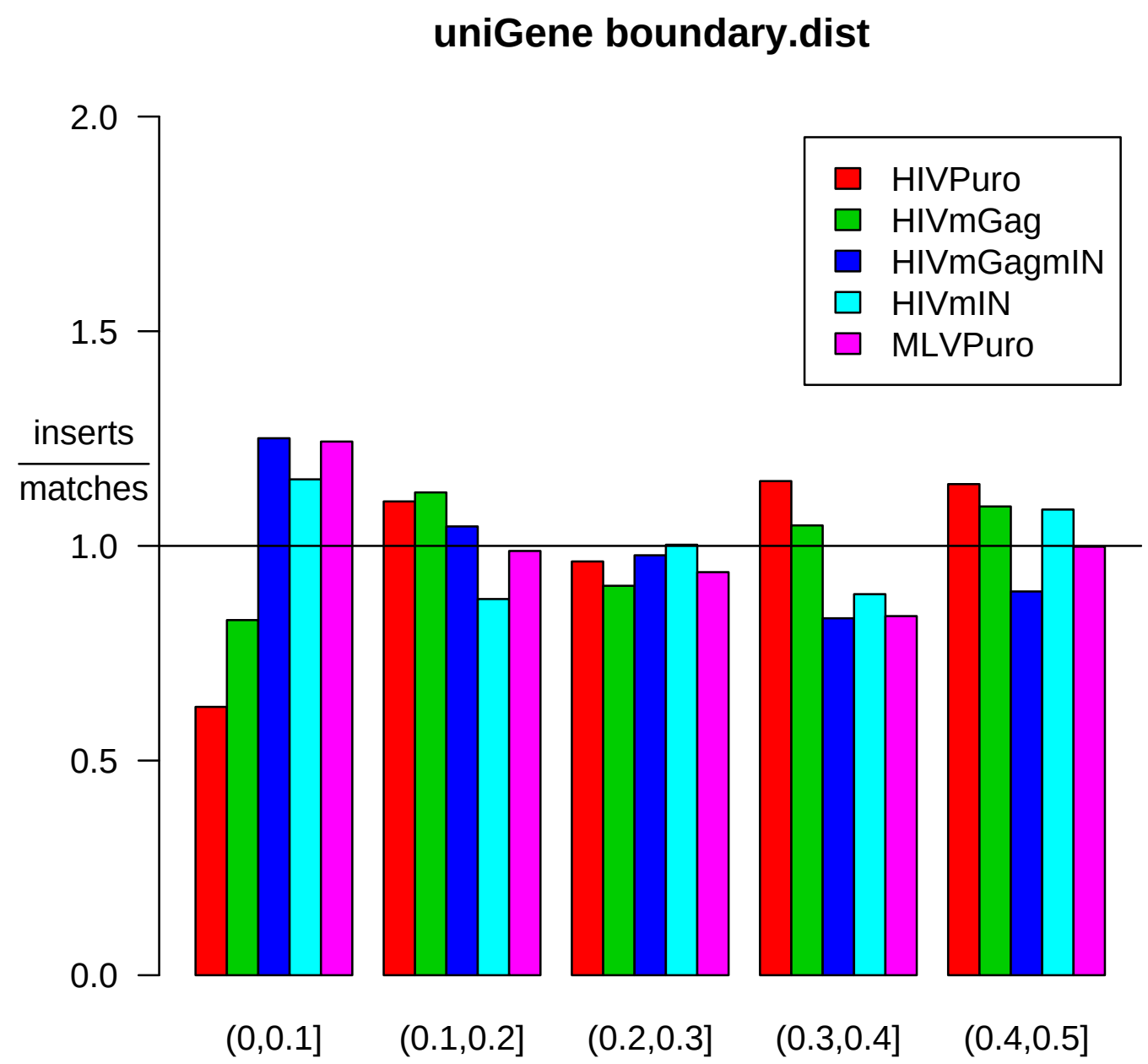

HIVPuro	HIVmGag	HIVmGagmIN	HIVmIN	MLVPuro
0.000154	0.167000	0.029900	0.396000	0.053300

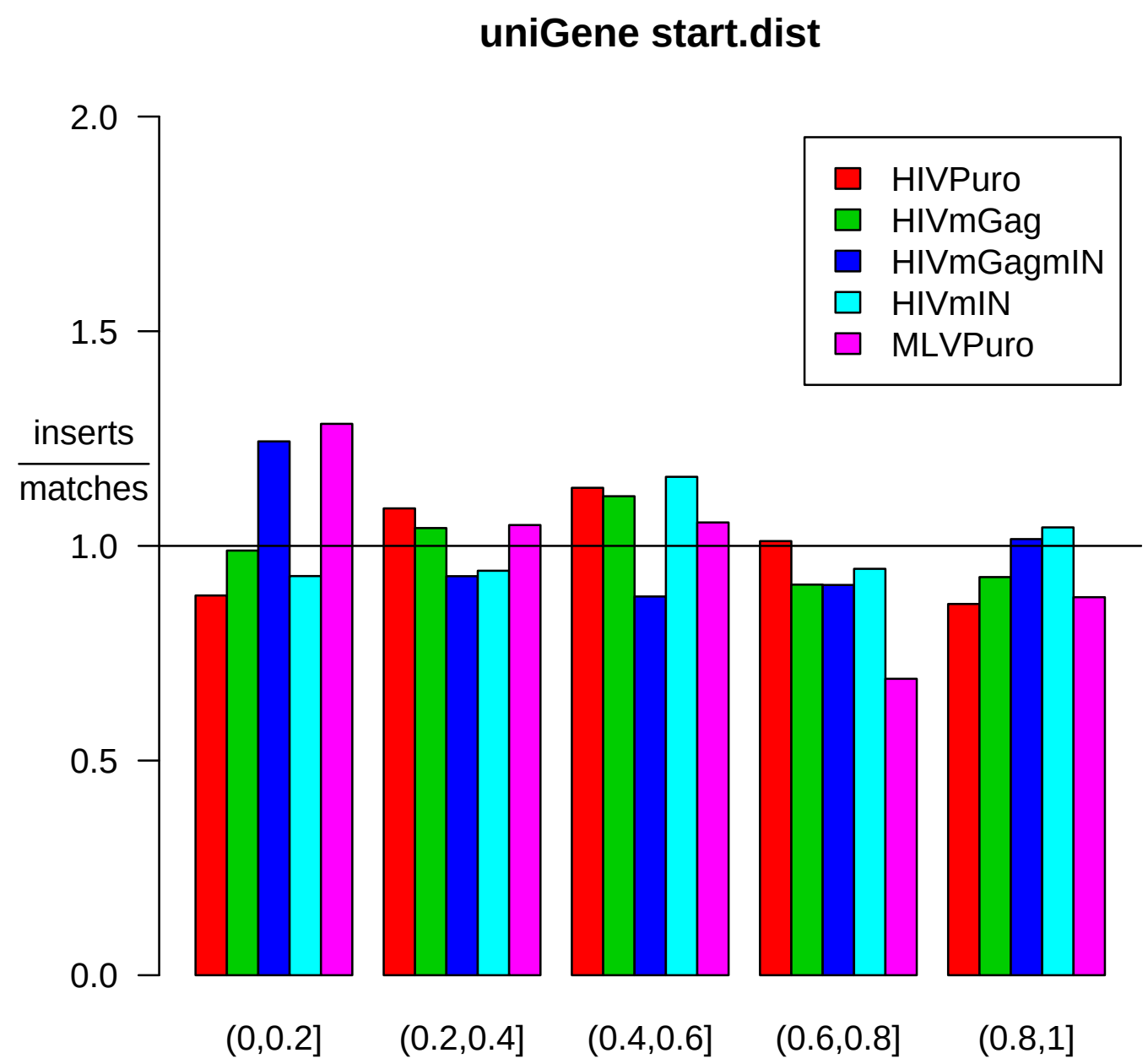

HIVPuro	HIVmGag	HIVmGagmIN	HIVmIN	MLVPuro
0.262000	0.636000	0.098200	0.656000	0.000701

6 GC content

Here we study the effect of GC content on insertion. The GC content is taken from the Human Genome Draft at GoldenPath from the table
http://genome.ucsc.edu/goldenPath/hg17/database/gc5Base.txt.gz.

Following the plot is a table of fitted coefficients based on splitting the GC percent data at the median.

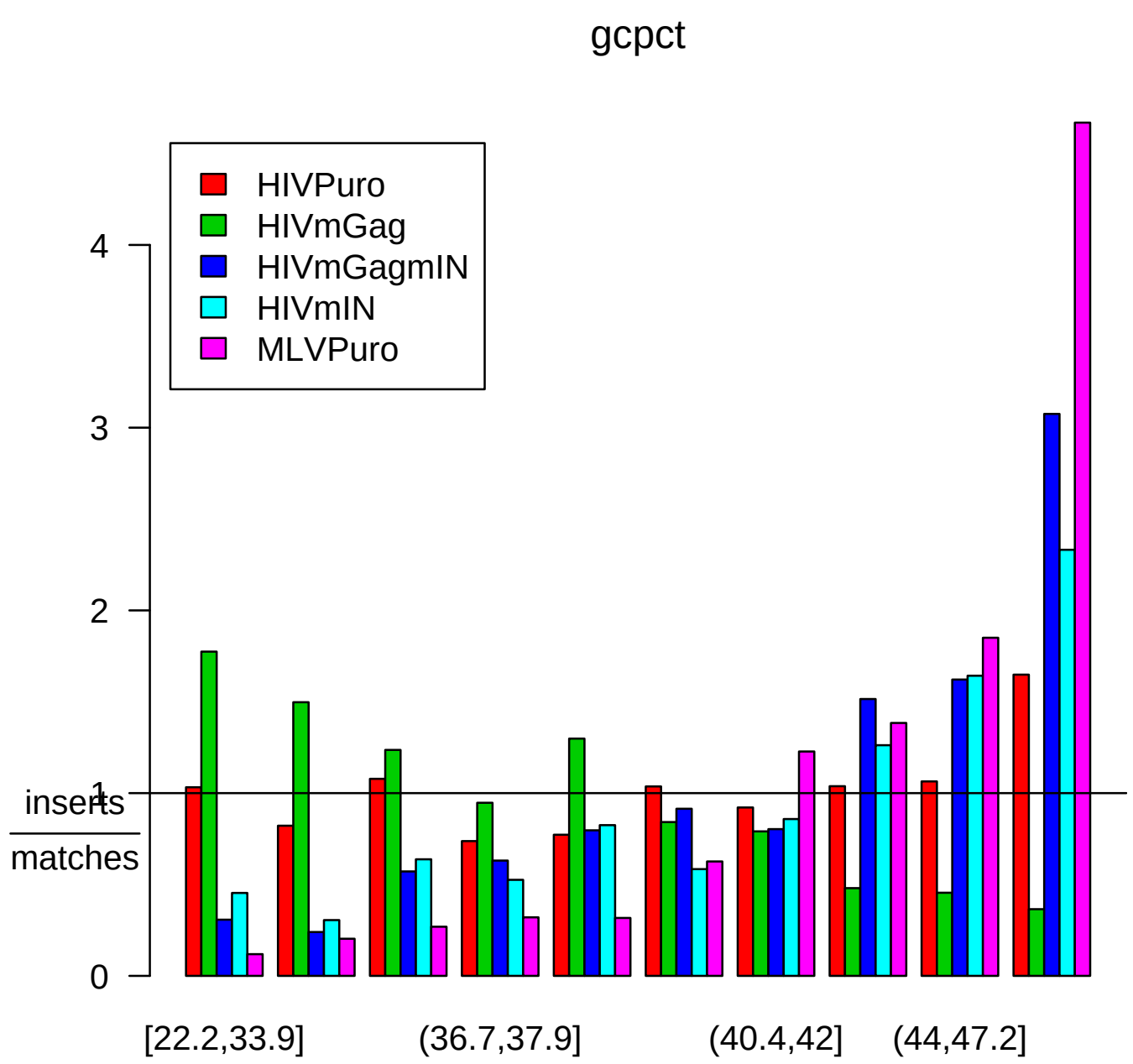

	coef	se	z	p
HIVPuro	0.234	0.092	2.54	1.10e-02
HIVmGag	-0.812	0.104	-7.82	5.49e-15
HIVmGagmIN	1.110	0.104	10.80	5.50e-27
HIVmIN	0.914	0.128	7.13	1.02e-12
MLVPuro	2.050	0.133	15.40	8.63e-54

7 Cytobands

Here we study the association of cytoband with insertion intensity. The data are obtained from
`http://genome.ucsc.edu/goldenPath/hg17/database/cytoBand.txt.gz`.

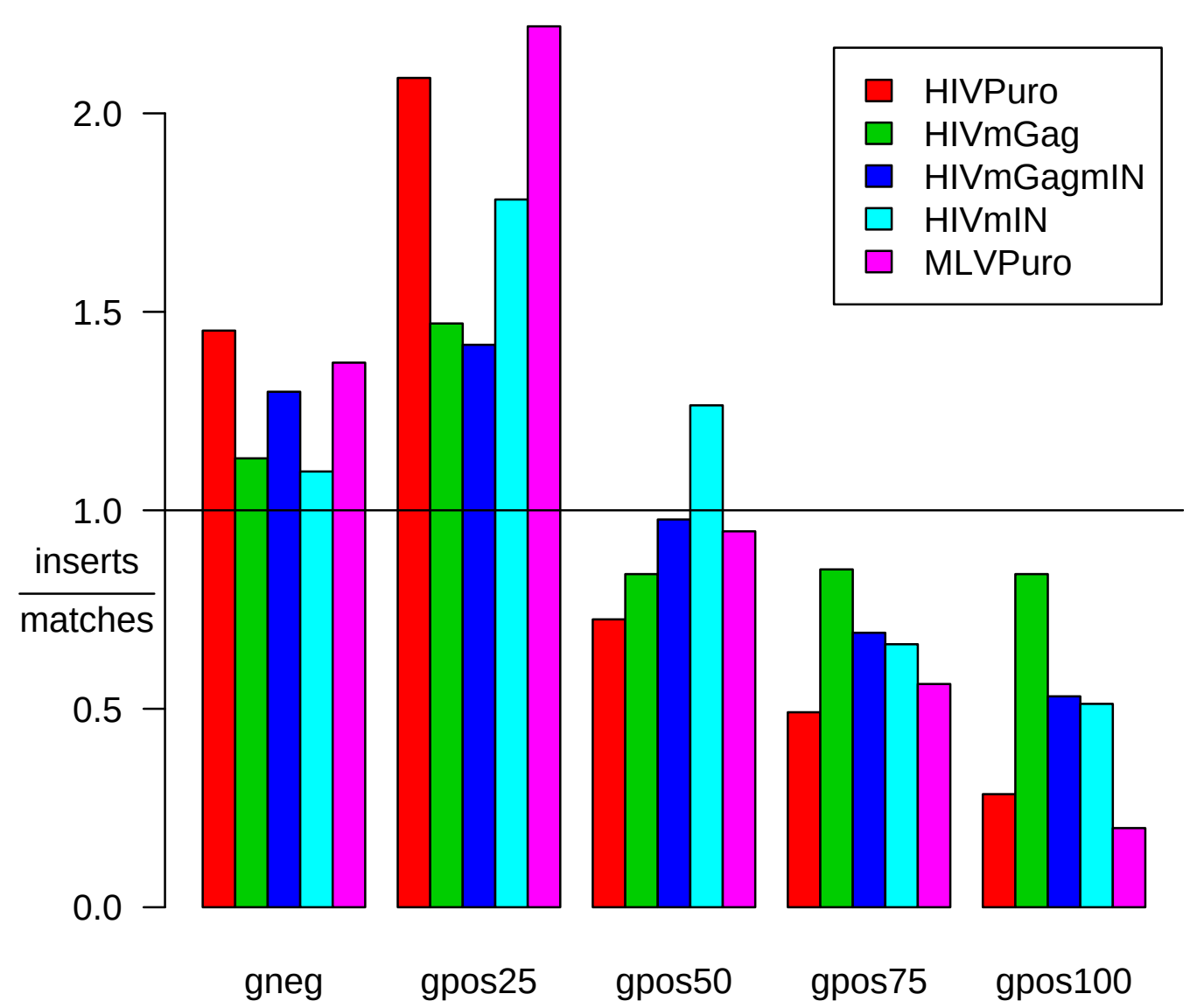

A formal test of significance attains a p-value of $< 2.22e-16$. Here is the table of coefficients of the log ratio of intensities for true insertion sites versus control insertion sites (comparing each category of Giemsa staining to 'gneg') along with their standard errors, z statistics, and p-values:

```
                   coef     se      z        p
cyto.typegpos100 -1.020 0.0750 -13.60 6.62e-42
cyto.typegpos25   0.342 0.0689   4.96 6.94e-07
cyto.typegpos50  -0.322 0.0643  -5.00 5.68e-07
cyto.typegpos75  -0.687 0.0735  -9.36 8.33e-21
```

References

[1] Yvonne M.M. Bishop, Stephen E. Fienberg, and Paul W. Holland. *Discrete multivariate analyses: Theory and practice* (MIT Press, 1975).

[2] P. McCullagh and John A. Nelder. *Generalized linear models.* (Chapman & Hall ltd, 1999).

[3] Xiaolin Wu, Yuan Li, Bruce Crise, Shawn M. Burgess "Transcription Start Regions in the Human Genome Are Favored Targets for MLV Integration," *Science,* **300**(5626), (June 2003): 1749-1751.
