# Supplementary material for: Retroviral DNA Integration: Viral and Cellular Determinants of Target-Site Selection
Source: PLoS Pathog. 2006 Jun 23;2(6):e60. doi: 10.1371/journal.ppat.0020060 (PMC1480600; doi:10.1371/journal.ppat.0020060)
Supplement: Supplementary file 2 [file ppat.0020060.sd002.pdf]

Screening Transcription Factors for Effects on Retroviral Integration

Charles C. Berry

Abstract

Using data from Rick Bushman's group — with special contributions from Mary Lewinski and Sridhar Hallenhalli — the associations of a collection of transcription factors with integration of various 'integration complexes' (including HIVPuro and MLVPuro vectors) are studied.

Of special interest is the existence of *interaction effects* with *local GC percentage*; that is, the joint effects of local GC percentage and particular transcription factors differs from their separate effects.

Contents

1 Association of Transcription Factors with Integration 1

2 Other Factors in Integration Targetting 4
2.1 GC Percent and Transcription Factors 6

3 Extending the Screen to All 'Other' Features 7
3.1 PCs for 'Other' Features 7
3.2 PCs for Positional Weight Matrices 11
3.3 Screening PCs for Confounding and Effect Modification 13
3.3.1 HIVPuro 14
3.3.2 HIVmGAGmIN 15
3.3.3 HIVmGAG 17
3.3.4 HIVmIN 18
3.3.5 MLVPuro 20

1 Association of Transcription Factors with Integration

A collection of 531 positional weight matrices (PWMs) (representing a collection of transcription factors) was used to anotate 26796 $\pm$1kilobase regions surrounding genomic sites. These sites are as follows:

```
             type
IntCplx       insertion match
  HIVPuro           524  5240
  HIVmGAGmIN        526  5260
  HIVmGAG           493  4930
  HIVmIN            350  3500
  MLVPuro           543  5430
```

The 'IntCplx's refer to the different integration complexes that were used in these experiments. The 'insertion' type represents actual integration events, while the 'match' type represents *in silico* events that are sampled from the genome (10 per 'insertion') to match an insertion with respect to its distance from the restriction sites used to isolate integration events.

For each combination of integration complex and PWM a 2 by 2 tables of counts is formed using the first in silico match for each insertion. Here is an example for MLVPuro and M00189:

```
           PWM("M00189")
type         NoTF   TF
  insertion  323  220
  match     4551  879
```

Evidently, the actual insertions have more integration events than their in silico matches. Formally, this may be assessed by the conditional logit test (as implemented in `survival` Package [Therneau and Lumley,]) for R that assigns each integration event and its matches to a single stratum and calculates the association between 'type' of integration event (insertion or match) and whether the PWM matched a sequence in the region ('TF') or not ('noTF'). This procedure aggregates results over all such strata thereby controlling for possible effects of the restriction enzyme used. The test statistic used is

$$z = \frac{\beta}{se_\beta}$$

where β is (in this special case of a binary regressor) the natural logarithm of the common odds ratio in the 2 by 2 table in each stratum and se_β estmiates its standard error. For M00189, a value of β =1.27 obtained, which corresponds to an odds ratio of 3.55.

The associated z-statistic is z =13.29. The figure below shows the histogram of z-values for the entire collection of PWMs for MLVPuro.

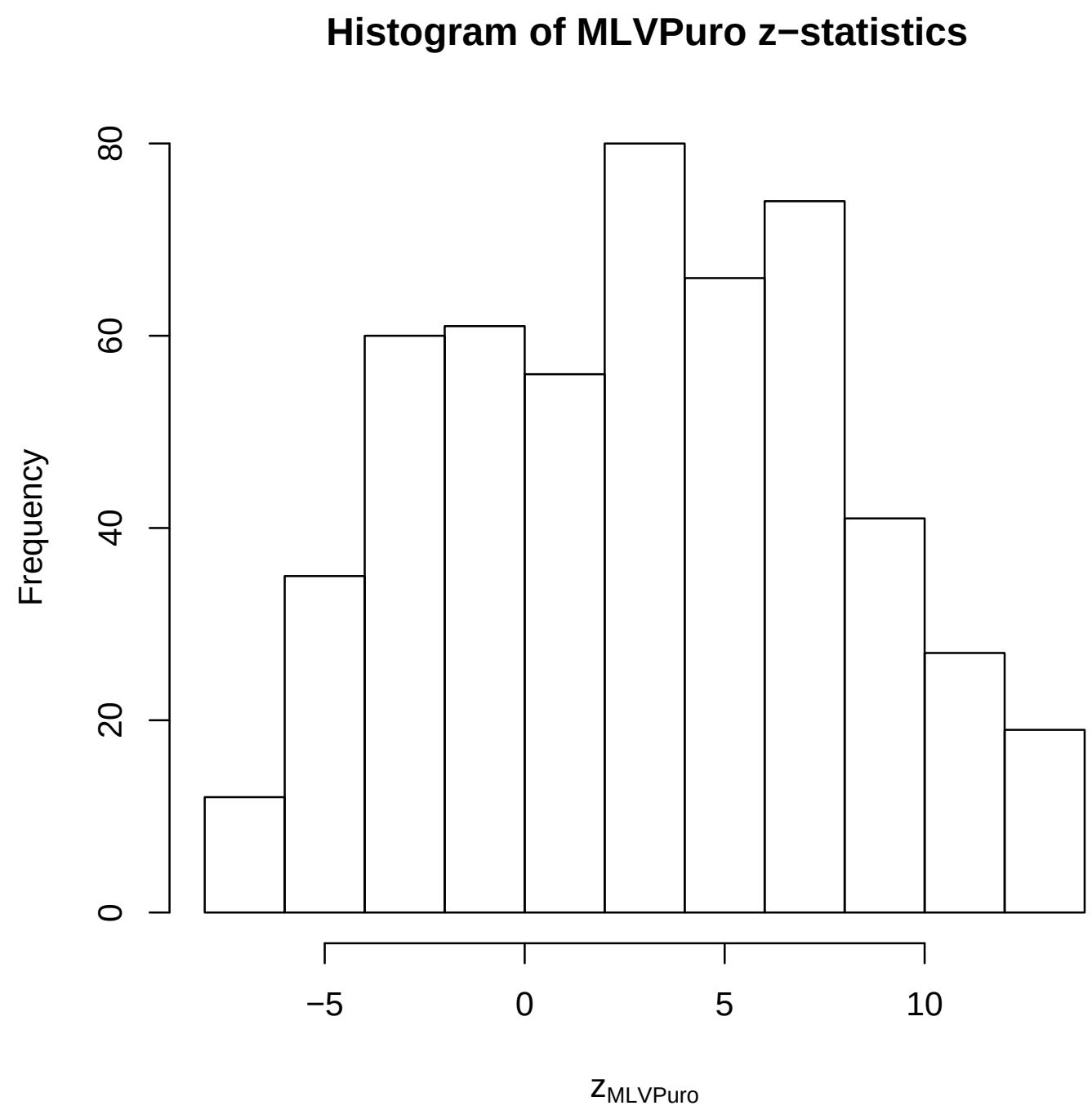

A two-sided test for the z-statistic attains $p < 0.05$ when it exceeds ± 1.96 and $p < 0.01$ when it exceeds ± 2.58. Evidently, many values in the histogram above exceed those values. In assessing the association of a large number of candidate predictors, it is useful to consider the *false discovery rates* (FDRs), that is the fraction of candidates that are selected as showing association that in reality have no association with integration events. Using the qvalue R package [Dabney et al.,], the cutoffs for a FDR of 5 percent can be found for the collection of z-statistics associated with each integration complex. Those cutoffs depend on the observed distribution of p-values and it may happen that the cutoff for a 5 percent FDR is higher or lower than the cutoff for $p = 0.05$.

The following matrix shows the scatterplots of each pair of integration complexes with pink lines to denote the 5 percent FDR cutoffs for each complex.

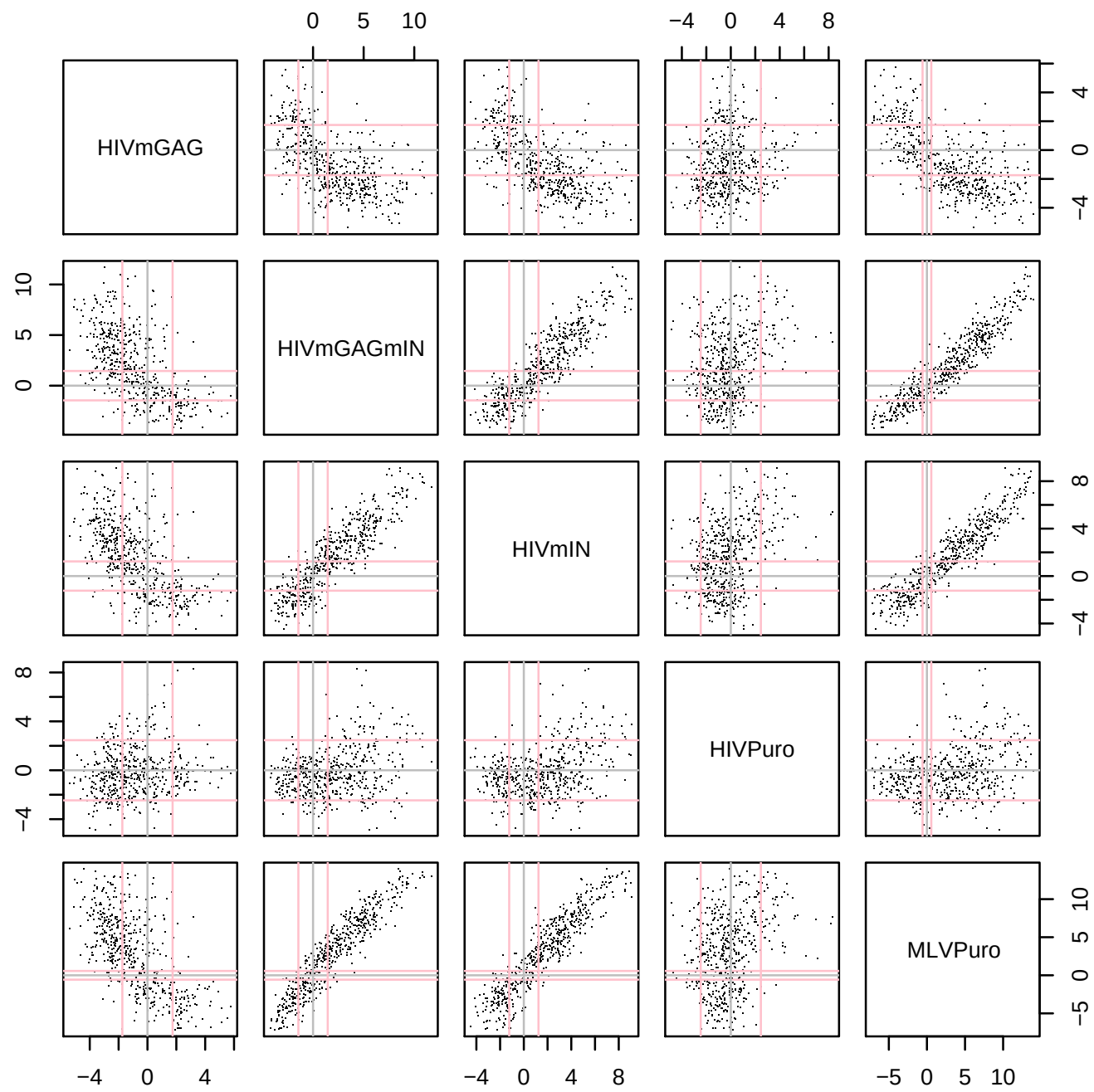

Evidently, many transcription factors are associated with integration siting. Perhaps, it is interesting to note that many of the PWMs whose associations with HIVmGAG sites are negative have positive associations with HIVmGAG-mIN sites.

2 Other Factors in Integration Targetting

Previously, we have studied the effect of various genomic fatures on integration targetting [Mitchell et al., 2004]. Plausibly, some of these could account for the association of transcription factors with integration siting. That is, if PWM M00189 tends to mark sites in GC rich regions more often than sites in GC poor regions, then the tendency of MLVPuro sites to be found in GC rich regions will induce a correlation of integration targetting with M00189 even if the transcription factor associated M00189 has no intrinsic effect on integration. By introducing a variable to represent GC richness, the effect of M00189 on targeting can be observed net of any effects of GC richness.

Here is the result of regressing MLVPuro integration site on GC richness as represented by the GC percent in 5 kilobase windows (`gcpct`) taken from the file

`hg17/database/gc5Base.txt.gz`

on the GoldenPath web site

(http://hgdownload.cse.ucsc.edu/goldenPath/).

The label `coef` is for β, `exp(coef)` gives the relative increase in the odds for a 1 percent increase in `gcpct` and `z` and `p` are obviously the z-statistic and its p-value (rounded off — so very small values appear as '0').

```
          coef exp(coef)    se(coef)        z p
gcpct 0.1569463  1.169933 0.007643321 20.53379 0
```

As can be seen the z-statistic has a larger value than the corresponding z-statistic for M00189 reported above, which superficially suggests that GC percent could be more important as a determinant of MLVPuro targetting than M00189 is. To assess this, we 'control' for the effects of GC richness by regressing MLVPuro siting on both `gcpct` and `M00189`

```
                   coef exp(coef) se(coef)       z     p
gcpct            0.1482    1.1597   0.0088 16.7623 0.000
PWM("M00189")TF 0.2314    1.2604   0.1184  1.9550 0.051
```

Comparing these coefficients to those obtained earlier, it turns out that `gcpct` is slightly reduced, while `PWM("M00189")TF` is greatly reduced, and its effect on integration targetting is no longer statistically significant. So, it appears that the transcription factors represented by M00189 play at most a limited role in MLVPuro siting. A variable whose inclusion in a regression model substantially reduces the effect of another variable is sometimes called a *confounder*, because failing to account for its effects may give a misleading impression of the other variable's importance in determining the dependent variable. Additional variables could be added to the regression in an attempt to see if further reductions in the association of M00189 with MLVPuro sites occurs. But before pursuing that possibility and expanding the analysis to accomodate other PWMs, it is worth looking at a slightly more complicated model than the one just fit. The model just fit supposes that the incremental effect of `gcpct` is the same regardless of the value of M00189; the model presented here relaxes that assumption:

```
                          coef exp(coef) se(coef)       z      p
gcpct                   0.1716    1.1871   0.0116 14.7826 0.0000
PWM("M00189")TF         2.6781   14.5578   0.7765  3.4488 0.0006
gcpct:PWM("M00189")TF -0.0535    0.9479   0.0169 -3.1732 0.0015
```

The term `gcpct` now reflects the effect of GC percent when `PWM("M00189")` has the value 'noTF' (i.e. the PWM did not match) and the term `gcpct:PWM("M00189")TF` reflects the increase in the effect beyond that when `PWM("M00189")` has the value 'TF'. (This term is often referred to as an *interaction effect* and sometimes one of the variables is said to be an *effect modifier* implying that is changes the efefct of the other variable.) So, if both these coefficients are positive, then GC percent increases the odds of MLVPuro siting when the PWM did not match

and increases it still more when the PWM did match. Likewise, if both are negative, then the effect of increasing `gcpct` to disfavor MLVPuro siting is intensified when the PWM found a match. As can be seen, both coefficient of `gcpct` is positive and that of `gcpct:PWM("M00189")TF` is negative so the effect of GC percent to increase MLVPuro targetting is reduced when M00189 finds a match. The coefficient of `PWM("M00189")TF` along with `PWM("M00189")TF` set the relative odds for 'TF' versus 'noTF' when a value of `gcpct` is given. For example, the median of `gcpct` in the data used here is 39.28. At this value, the odds of MLVPuro siting are 1.78 times as great when the PWM matches as when it does not. In other words, there is a modest difference between the intensity of MLVPuro targetting when M00189 matches as when it does not for values of `gcpct` at this level. For slightly higher of `gcpct`, the effect of a M00189 match to increase MLVPuro targetting is reduced and for a value of `gcpct` of 50 the odds increase by a factor of 1.003 with a M00189 match.

2.1 GC Percent and Transcription Factors

The matrix of scatterplots below shows the z-statistics for the interaction of `gcpct` and of the PWMs for each pair of integration complexes.

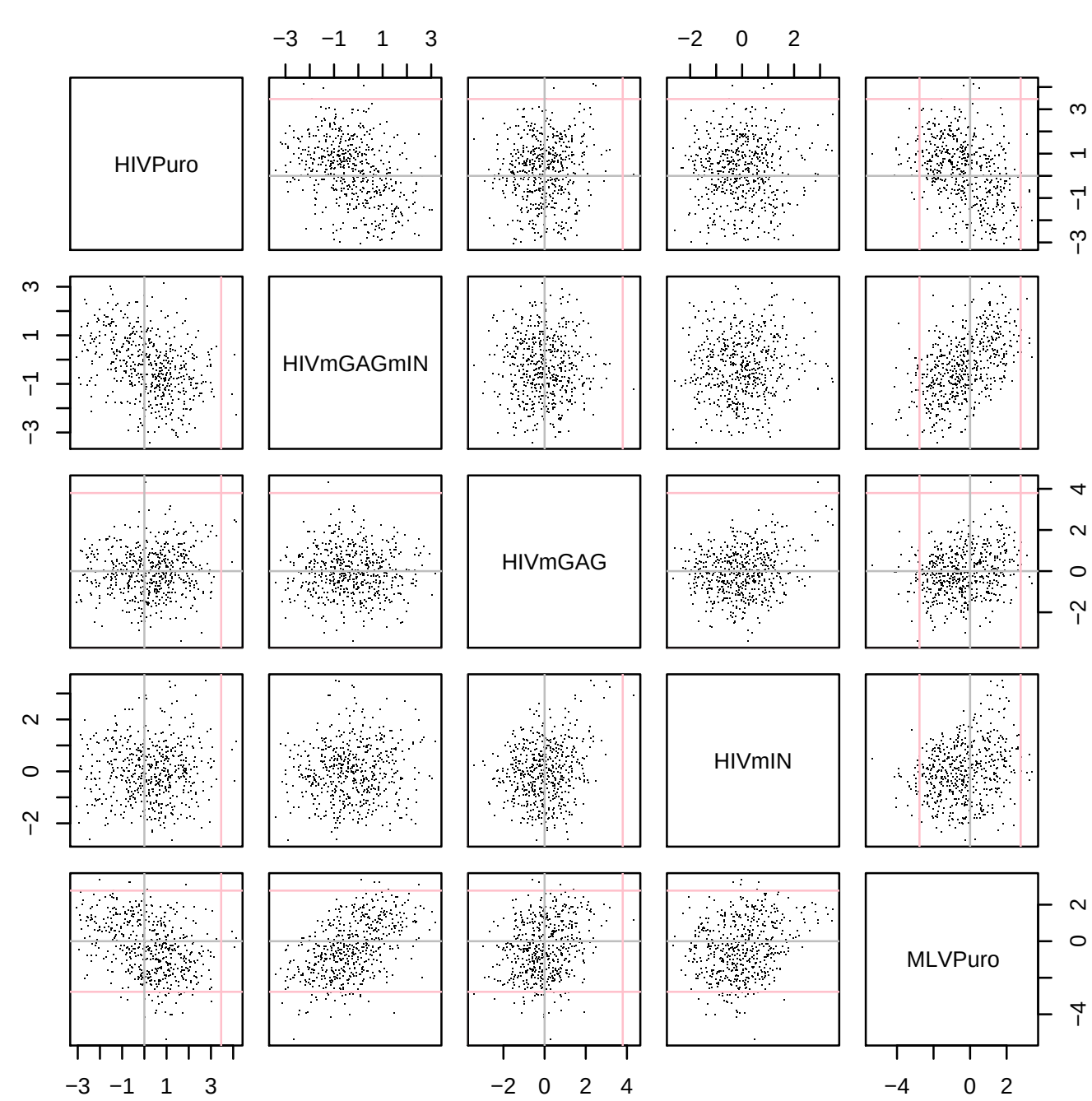

Evidently, there are a few interaction effects between `gcpct` and the `PWMs`.

3 Extending the Screen to All 'Other' Features

So far, the analysis of confounding and interaction effects involving PWMs has been restricted to `gcpct`. However, there are numerous genomic features other than `gcpct` that could be considered. Given that there are 531 PWMs, 5 Integration Complexes, over 100 genomic features available for study , and the number of combinations exceeds 10^{200}. Obviously, examining each combination is infeasible, and some data reduction strategy is needed.

One alternative finds low dimensional summaries of the PWMs and the other genomic features and uses these as the objects of study. For example, the first few principal components (i.e. those with the largest variances) of (the scaled values of) each set of features and their cross-products could be used in conditional logit regression analyses. This is very feasible, but begs the question "is the interesting variation in each set confined to the first few principal components?" A preliminary screening of all principal components in each set could be used to decide which to include in a more detailed analysis. This kind of selection biases assessments of the correlations of the features in a set in favor of finding larger correlations, however, inferences about confounding and possible effect modification seem less likely to be affected by this sort of preliminary screening.

One difficulty that would need to be addressed in using this approach lies in interpretng the end results. Principal components are linear combinations (weighted sums and differences) of a collection of variables. Usually, the variables have some well understood interpretation, but it can happen that an apparently important principal component does not. However, by inspecting the weights used to form a principal component, it may happen that an interpertation is forthcoming. As it will turn out, the largest principal component in the collection of genomic features (other than PWMs) gives large negative weights to measures of gene density and expression density. (The sign of a principal component is arbitrary.) Other important components likewise turn out to combine features that tap a common theme.

3.1 PCs for 'Other' Features

The percent of variance for the principal components for the other features is given in this plot:

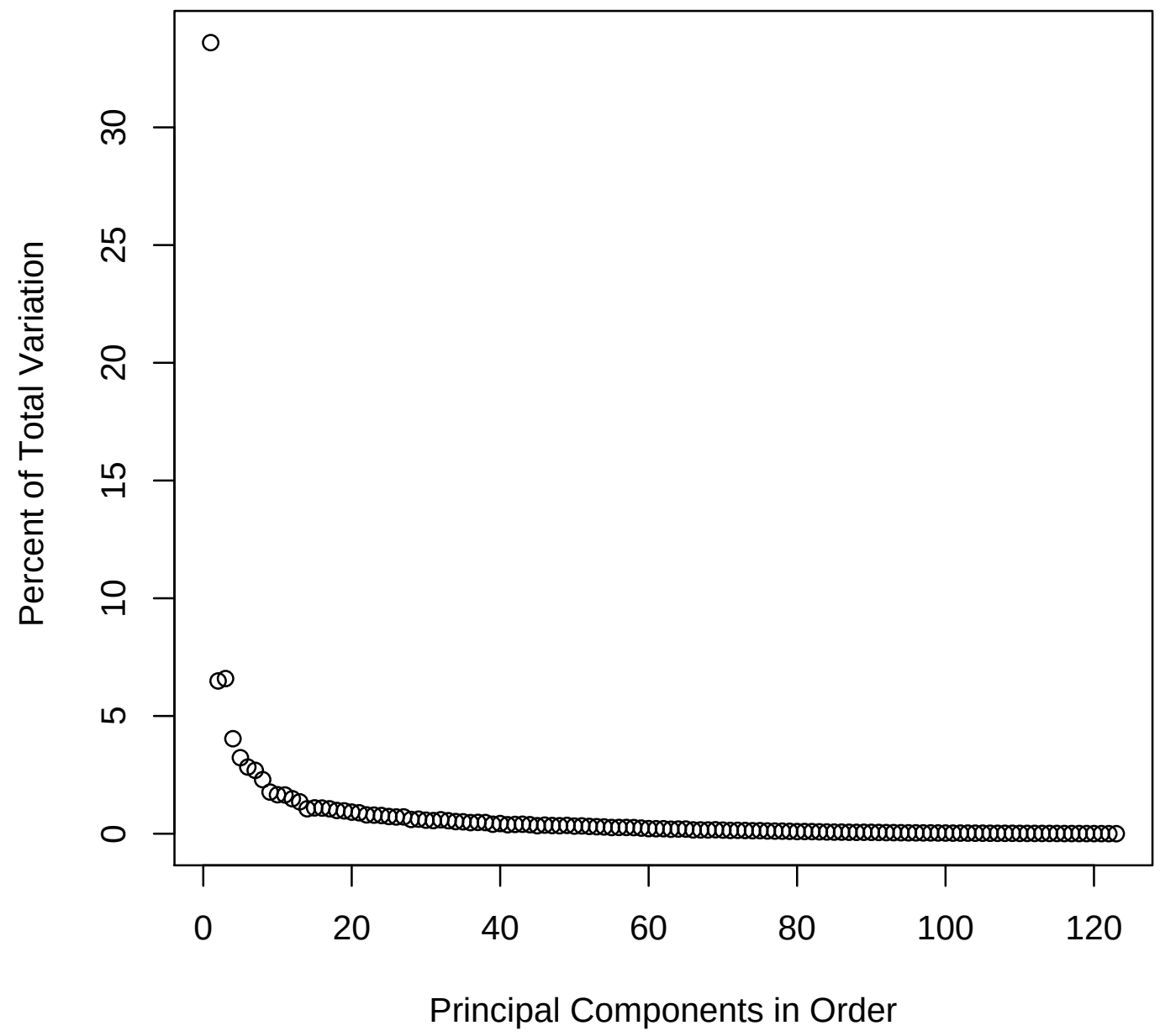

The largest principal component accounts for more than 30 percent of the total variation, which suggests much redundancy in the data. This is no surprise as many measures of gene density are taken using different annotation schemes and different window widths. Likewise other features based on different annotation schemes but attempting to measure the same phenomenon are used adding to the redundancy.

Selected correlations of the principal component scores with a zero-one indicator for 'insertion' vs 'match' appear in the next table. (Each row has at least one correlation greater than 0.10 in magnitude.) The "*" indicates correlations that surpass the FDR= 0.01 cutoff.

	HIVPuro	HIVmGAGmIN	HIVmGAG	HIVmIN	MLVPuro
PC1	-0.381*	-0.225*	-0.089*	-0.288*	-0.354*
PC5	-0.124*	0.094*	-0.065*	0.113*	0.157*
PC6	0.095*	-0.039	0.134*	-0.047*	-0.069*
PC8	0.056*	0.128*	0.038	0.095*	0.205*
PC29	-0.145*	0.004	-0.081*	-0.031	-0.017
PC32	-0.023	0.104*	-0.023	0.041	0.066*
PC46	0.103*	0.007	0.073*	0.015	0.029
PC50	0.101*	-0.032	0.067*	0.084*	0.011

It is no surprise that the largest principal component has substantial correlations with integration targetting for each integration complex, because the

features used were selected based on previous studies that showed their impact on integration and because of the redundancy noted above.

It is worth noting that several 'minor' principal components showed some correlation for one or more of the integration complexes. Perhaps it is also worth noting that many correlations exceeded the cutoff for FDR= 0.01. The table below shows the tallies:

	HIVPuro	HIVmGAGmIN	HIVmGAG	HIVmIN	MLVPuro
FDR > 0.01	82	105	104	102	89
FDR < 0.01	41	18	19	21	34

Most of the correlations that pass the FDR= 0.01 cutoff are very modest. In further analyses only the components given above will be used. The correlations of these components with the original features are given in this table.

	X.PC1	X.PC5	X.PC6	X.PC8	X.PC29	X.PC32	X.PC46	X.PC50
acembly.genes	0.28	0.19	-0.54	-0.17	0.05	-0.10	-0.03	-0.01
refGene.genes	0.25	0.22	-0.57	-0.16	0.12	0.02	-0.06	-0.09
uniGene.genes	0.19	0.22	-0.57	-0.16	0.06	-0.05	0.05	0.04
ace.100k	0.75	-0.07	-0.09	-0.05	0.17	-0.04	-0.05	-0.11
ace.200k	0.82	0.01	0.00	-0.07	0.15	0.02	-0.04	-0.07
ace.500k	0.88	0.09	0.10	-0.10	0.10	0.01	-0.08	-0.04
ace.1M	0.91	0.10	0.11	-0.10	0.08	-0.05	-0.07	-0.03
ace.2M	0.90	0.07	0.08	-0.09	0.06	-0.05	-0.03	-0.03
ref.100k	0.66	-0.04	-0.06	0.06	0.18	-0.07	0.02	-0.03
ref.200k	0.77	0.03	0.04	0.03	0.17	0.00	0.03	0.03
ref.500k	0.84	0.11	0.13	-0.02	0.12	0.00	-0.02	0.05
ref.1M	0.88	0.11	0.14	-0.04	0.09	-0.05	-0.02	0.04
ref.2M	0.88	0.09	0.10	-0.05	0.07	-0.06	0.00	0.02
uni.100k	0.63	-0.03	-0.16	-0.03	-0.03	-0.02	0.04	-0.07
uni.200k	0.69	0.00	-0.09	-0.03	-0.05	0.02	0.03	-0.03
uni.500k	0.77	0.04	-0.02	-0.03	-0.06	0.01	-0.03	0.03
uni.1M	0.80	0.05	0.00	-0.03	-0.05	-0.04	-0.04	0.04
uni.2M	0.82	0.03	-0.01	-0.02	-0.04	-0.04	-0.03	0.04
gen.100k	0.48	-0.22	0.12	0.36	0.11	0.00	0.02	-0.17
gen.200k	0.57	-0.18	0.16	0.37	0.09	0.03	0.03	-0.09
gen.500k	0.65	-0.11	0.18	0.32	0.06	0.02	-0.01	-0.01
gen.1M	0.70	-0.07	0.15	0.27	0.03	-0.02	-0.01	0.02
gen.2M	0.71	-0.08	0.10	0.21	0.02	-0.02	0.01	0.01
onco.1M	0.35	0.25	0.14	0.09	0.04	-0.01	0.14	-0.05
onco.2M	0.42	0.22	0.11	0.06	0.04	-0.03	-0.04	0.01
onco.4M	0.48	0.15	0.06	0.03	0.05	-0.01	-0.18	0.06
dens.25k	0.44	-0.15	-0.22	0.17	-0.13	0.08	0.00	-0.01
low.ex.25k	0.42	-0.20	-0.24	0.15	0.19	0.10	0.02	0.02
med.ex.25k	0.35	-0.22	-0.23	0.13	0.17	0.11	0.08	0.03
dens.50k	0.56	-0.14	-0.16	0.15	-0.17	-0.07	-0.07	-0.05
low.ex.50k	0.53	-0.18	-0.18	0.11	0.15	-0.11	-0.09	-0.02

med.ex.50k	0.44	-0.19	-0.17	0.10	0.13	-0.11	-0.03	0.00
high.ex.50k	0.35	-0.17	-0.15	0.10	-0.13	-0.06	-0.08	-0.08
dens.100k	0.67	-0.08	-0.07	0.11	-0.13	-0.07	0.02	-0.03
low.ex.100k	0.64	-0.10	-0.08	0.04	0.14	-0.12	0.01	-0.01
med.ex.100k	0.54	-0.09	-0.06	0.03	0.10	-0.15	0.05	0.04
high.ex.100k	0.43	-0.08	-0.04	0.02	-0.15	-0.11	-0.02	-0.02
dens.250k	0.80	0.06	0.09	0.03	-0.09	0.04	0.05	0.00
low.ex.250k	0.77	0.08	0.11	-0.08	0.11	0.04	0.04	-0.01
med.ex.250k	0.68	0.10	0.13	-0.11	0.08	0.03	0.06	0.04
high.ex.250k	0.55	0.11	0.14	-0.12	-0.15	0.05	-0.01	0.02
dens.500k	0.85	0.13	0.16	-0.02	-0.05	0.03	-0.01	-0.01
low.ex.500k	0.83	0.17	0.19	-0.14	0.11	0.03	-0.04	-0.06
med.ex.500k	0.76	0.21	0.22	-0.18	0.10	0.01	-0.05	-0.05
high.ex.500k	0.64	0.23	0.23	-0.19	-0.09	0.03	-0.11	-0.05
dens.1M	0.89	0.14	0.15	-0.05	-0.03	-0.05	-0.01	-0.03
low.ex.1M	0.88	0.19	0.20	-0.17	0.10	-0.07	-0.03	-0.08
med.ex.1M	0.82	0.23	0.23	-0.21	0.11	-0.09	-0.02	-0.08
high.ex.1M	0.72	0.25	0.24	-0.23	-0.02	-0.08	-0.06	-0.06
dens.2M	0.89	0.10	0.11	-0.06	-0.01	-0.06	0.01	-0.03
low.ex.2M	0.89	0.15	0.14	-0.16	0.09	-0.08	0.02	-0.07
med.ex.2M	0.85	0.19	0.17	-0.20	0.11	-0.10	0.05	-0.06
high.ex.2M	0.77	0.22	0.19	-0.21	0.02	-0.10	0.03	-0.02
dens.4M	0.87	0.02	0.04	-0.05	-0.01	0.02	-0.01	-0.05
low.ex.4M	0.87	0.06	0.06	-0.12	0.06	0.01	0.00	-0.07
med.ex.4M	0.85	0.09	0.08	-0.16	0.08	0.00	0.03	-0.05
high.ex.4M	0.77	0.13	0.11	-0.18	0.02	0.01	0.02	0.01
dens.8M	0.81	-0.08	-0.07	-0.01	-0.01	0.06	-0.05	-0.07
low.ex.8M	0.83	-0.06	-0.05	-0.06	0.05	0.06	-0.04	-0.07
med.ex.8M	0.80	-0.04	-0.04	-0.09	0.07	0.06	-0.02	-0.04
high.ex.8M	0.75	0.00	-0.01	-0.11	0.03	0.08	-0.03	0.03
dens.16M	0.74	-0.19	-0.17	0.05	0.00	0.00	-0.05	-0.06
low.ex.16M	0.75	-0.18	-0.17	0.01	0.05	0.01	-0.04	-0.05
med.ex.16M	0.73	-0.17	-0.16	-0.01	0.07	0.00	-0.03	-0.01
high.ex.16M	0.69	-0.14	-0.14	-0.03	0.06	0.01	-0.03	0.05
dens.32M	0.64	-0.26	-0.23	0.07	-0.01	-0.08	-0.01	-0.05
low.ex.32M	0.65	-0.26	-0.24	0.04	0.03	-0.08	0.01	-0.04
med.ex.32M	0.64	-0.24	-0.23	0.02	0.06	-0.08	0.02	0.00
high.ex.32M	0.61	-0.21	-0.21	0.00	0.05	-0.08	0.03	0.05
gcpct	0.58	-0.26	0.08	-0.05	-0.06	-0.03	0.21	0.16
cpg.1k	0.25	-0.40	0.09	-0.32	0.04	-0.21	-0.12	-0.01
cpg.5k	0.37	-0.47	0.12	-0.28	0.03	-0.24	0.00	-0.06
cpg.10k	0.44	-0.45	0.14	-0.23	0.02	-0.08	0.00	-0.10
cpg.25k	0.51	-0.34	0.13	-0.13	0.02	0.20	-0.05	0.01
cpg.50k	0.52	-0.23	0.10	-0.05	0.01	0.26	-0.04	0.10
dnaseI.1k	0.19	-0.31	0.06	-0.42	0.06	0.06	0.03	-0.10
dnaseI.2k	0.24	-0.37	0.07	-0.48	0.05	-0.04	-0.02	-0.03

dnaseI.10k	0.42	-0.44	0.05	-0.44	0.00	-0.19	-0.01	0.15
dnaseI.25k	0.54	-0.39	0.05	-0.36	0.01	-0.07	-0.01	0.03
dnaseI.50k	0.63	-0.33	0.04	-0.27	0.03	0.02	-0.02	-0.06
dnaseI.100k	0.71	-0.24	0.03	-0.21	0.04	0.04	-0.02	-0.11
dnaseI.1M	0.85	-0.02	0.07	-0.12	0.03	0.00	-0.05	-0.12
dnaseI.5M	0.81	-0.11	-0.05	-0.03	0.01	0.06	-0.06	-0.15
dnaseI.20M	0.66	-0.27	-0.21	0.07	0.00	0.00	-0.07	-0.14
start.dx.ace	0.03	0.24	-0.33	0.06	-0.11	0.06	0.02	0.00
signed.dx.ace	0.10	0.19	-0.43	-0.07	-0.06	-0.08	0.03	-0.03
general.wd.ace	-0.37	0.11	-0.14	-0.13	-0.10	0.10	0.00	0.13
start.dx.ref	0.03	0.28	-0.34	0.07	0.24	0.11	0.02	-0.04
general.wd.ref	-0.61	-0.05	0.11	-0.02	-0.05	0.01	0.00	-0.05
general.wd.gens	-0.29	0.20	-0.14	-0.34	-0.09	0.07	0.22	-0.09
start.dx.uni	0.02	0.24	-0.33	0.04	-0.12	0.06	-0.07	0.06
signed.dx.uni	0.13	0.16	-0.40	-0.03	0.00	0.04	0.06	0.05
general.wd.uni	-0.44	0.08	-0.08	-0.13	-0.05	0.14	-0.05	0.04

3.2 PCs for Positional Weight Matrices

The percent of variance for the principal components for the PWMs is given in this plot:

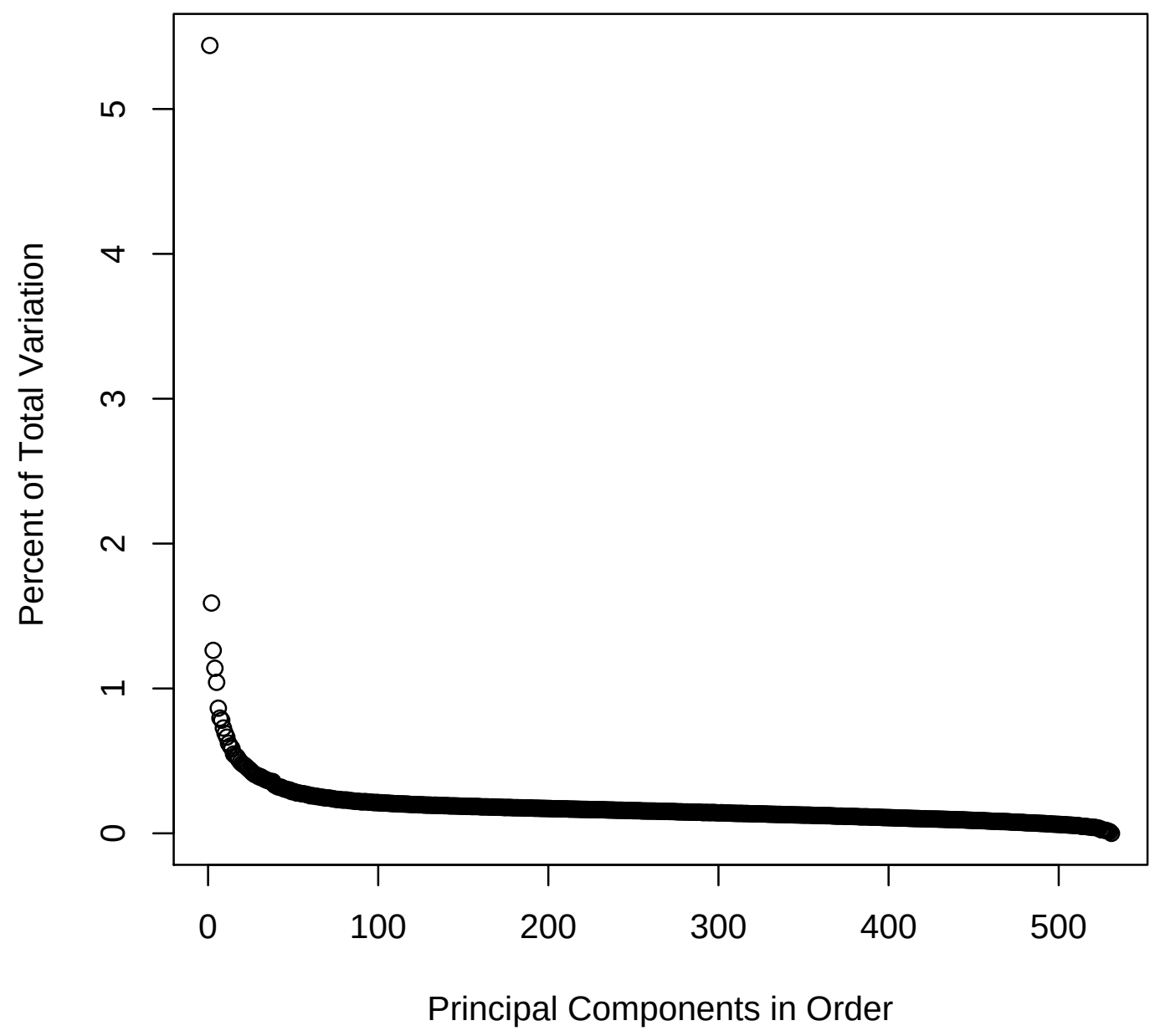

The largest principal component accounts for about 5 percent of the to-

tal variation. This may in part reflect the larger number of variables studied. The visual appearance of a few dominant components and many much smaller components is similar to that for the other genomic features.

Selected correlations of the principal component scores with a zero-one indicator for 'insertion' vs 'match' appear in the next table. (Each row has at least one correlation greater than 0.10 in magnitude.) The "*" indicates correlations that surpass the FDR= 0.01 cutoff.

	HIVPuro	HIVmGAGmIN	HIVmGAG	HIVmIN	MLVPuro
PC1	-0.025	-0.209*	0.108*	-0.205*	-0.303*
PC2	0.156*	0.033	0.102*	0.049	0.033

Here at least one of the largest two principal components have at least modest correlations with integration targetting for each integration complex.

Perhaps it is also worth noting how many correlations exceeded the cutoff for FDR= 0.01. The table below shows the tallies:

	HIVPuro	HIVmGAGmIN	HIVmGAG	HIVmIN	MLVPuro
FDR > 0.01	529	528	529	530	527
FDR < 0.01	2	3	2	1	4

Here only a few correlations pass the FDR= 0.01 cutoff. In further analyses all the components that passed that cutoff for a given integration complex will be used in analyses for that complex. The correlations of these components with the original features are given in this table. Correlations of less than 0.40 in magnitude are omiited to ease viewing.

	PWM.PC1	PWM.PC2	PWM.PC5	PWM.PC6	PWM.PC8
M00002	0.40				
M00008	0.43				
M00017					-0.46
M00041					-0.42
M00050			0.48		
M00143	0.44				
M00174				-0.58	
M00178					-0.43
M00188				-0.44	
M00189	0.49				
M00196	0.47				
M00199				-0.57	
M00255	0.46				
M00316		-0.61			
M00321	0.49				
M00323	0.48				
M00324	0.46				
M00330		-0.60			
M00333	0.42				

M00338					-0.44
M00373	0.45				
M00428	0.46				
M00431	0.44				
M00469	0.46				
M00470	0.44				
M00480		-0.45			
M00490				-0.41	
M00516	0.44				
M00517				-0.48	
M00695	0.41				
M00716	0.45				
M00738			0.45		
M00740			0.46		
M00797	0.40				
M00800	0.47				
M00801					-0.42
M00807	0.42				
M00915	0.49				
M00917					-0.46
M00918	0.49				
M00919	0.47				
M00920	0.47				
M00925				-0.56	
M00926				-0.57	
M00929	0.43				
M00931	0.46				
M00932	0.47				
M00933	0.43				
M00938	0.47				
M00939	0.49				
M00940	0.47				
M00976	0.41				
M00981					-0.55
M00982	0.45				
M00983		-0.55			
M00986	0.41				
M01001	0.43				

3.3 Screening PCs for Confounding and Effect Modification

The results for each integration complex are considered in the this section.

3.3.1 HIVPuro

The table of coefficients for the other features is:

	coef	exp(coef)	se(coef)	z	p
X.PC1	0.1207379	1.1283291	0.007071203	17.074591	0.0e+00
X.PC29	0.3177331	1.3740095	0.052754154	6.022902	1.7e-09
X.PC5	0.1673096	1.1821202	0.023340526	7.168204	7.6e-13
X.PC46	-0.3352490	0.7151600	0.064105789	-5.229621	1.7e-07
X.PC50	-0.2994394	0.7412336	0.071933376	-4.162733	3.1e-05
X.PC14	-0.1583334	0.8535652	0.046205661	-3.426709	6.1e-04
X.PC6	-0.2153389	0.8062682	0.025395281	-8.479483	0.0e+00
X.PC28	-0.1859700	0.8302985	0.063658102	-2.921388	3.5e-03
X.PC20	0.0951034	1.0997726	0.045898461	2.072039	3.8e-02
X.PC13	-0.1480670	0.8623733	0.038260236	-3.869998	1.1e-04

The analysis of deviance table here compares a model with just PWM PCs in it to the model with both PWM and 'other' feature PCs. If significant, it shows that 'other' features have an association with targetting independent of that due to PWMs.

```
Analysis of Deviance Table

Model 1: PWM PCs
Model 2: Both PWM PCs and 'other' PCs
  Resid. Df Resid. Dev   Df Deviance  P(>|Chi|)
1      5762    2354.15
2      5752    1640.35   10   713.81 6.824e-147
```

The table of coefficients for the PWMs is:

	coef	exp(coef)	se(coef)	z	p
PWM.PC2	-0.1738340	0.8404364	0.01502861	-11.566869	0.0e+00
PWM.PC6	0.1146841	1.1215191	0.02316062	4.951685	7.4e-07

The analysis of deviance table here compares a model with just 'other' PCs in it to the model with both PWM and 'other' feature PCs. If significant, it shows that PWM PCs have an association with targetting independent of that due to 'other' features.

```
Analysis of Deviance Table

Model 1: 'other' feature PCs
Model 2: Both PWM PCs and 'other' PCs
  Resid. Df Resid. Dev   Df Deviance P(>|Chi|)
1      5754     1652.1
2      5752     1640.3    2     11.7  0.002874
```

And here is the table of coefficients with both PWM PCs and 'other' PCs. Substantial reductions in the magnitude of these coefficients from the earlier tables show that the added variables in the model may 'confound' the association seen earlier.

	coef	exp(coef)	se(coef)	z	p
PWM.PC2	-0.04827033	0.9528762	0.017942577	-2.690267	7.1e-03
PWM.PC6	0.05607902	1.0576813	0.026630775	2.105798	3.5e-02
X.PC1	0.11404875	1.1208068	0.007307753	15.606542	0.0e+00
X.PC29	0.30404312	1.3553275	0.052728854	5.766162	8.1e-09
X.PC5	0.16369309	1.1778528	0.023378531	7.001855	2.5e-12
X.PC46	-0.33021678	0.7187679	0.064331560	-5.133045	2.9e-07
X.PC50	-0.29226496	0.7465707	0.071945287	-4.062323	4.9e-05
X.PC14	-0.15893295	0.8530536	0.046371820	-3.427361	6.1e-04
X.PC6	-0.21510978	0.8064529	0.025436130	-8.456860	0.0e+00
X.PC28	-0.18467530	0.8313742	0.063762629	-2.896294	3.8e-03
X.PC20	0.09684114	1.1016853	0.045986551	2.105858	3.5e-02
X.PC13	-0.13431859	0.8743115	0.038419781	-3.496079	4.7e-04

Tests for interactions between the PWM principal components and the 'other' features principal components yielded no results beyond the FDR= 0.05 cutoff.

3.3.2 HIVmGAGmIN

The table of coefficients for the other features is:

	coef	exp(coef)	se(coef)	z	p
X.PC1	0.07206298	1.0747230	0.005856132	12.305560	0.0e+00
X.PC8	-0.09797616	0.9066705	0.024269727	-4.036970	5.4e-05
X.PC32	-0.15476543	0.8566161	0.047179376	-3.280362	1.0e-03
X.PC5	-0.04504427	0.9559552	0.019062934	-2.362924	1.8e-02
X.PC40	0.07291283	1.0756368	0.052996588	1.375802	1.7e-01
X.PC44	0.23376434	1.2633467	0.060555284	3.860346	1.1e-04
X.PC9	0.13446876	1.1439289	0.035037172	3.837888	1.2e-04
X.PC33	0.15072497	1.1626768	0.049978152	3.015817	2.6e-03
X.PC89	-0.47493279	0.6219269	0.138441533	-3.430566	6.0e-04
X.PC7	0.03217988	1.0327032	0.019552752	1.645798	1.0e-01

The analysis of deviance table here compares a model with just PWM PCs in it to the model with both PWM and 'other' feature PCs. If significant, it shows that 'other' feature have an association with targetting independent of that due to PWMs.

```
Analysis of Deviance Table

Model 1: PWM PCs
Model 2: Both PWM PCs and 'other' PCs
```

```
  Resid. Df Resid. Dev   Df Deviance P(>|Chi|)
1      5783    2247.07
2      5773    2076.43   10   170.65 2.038e-31
```

The table of coefficients for the PWMs is:

```
               coef exp(coef)    se(coef)         z       p
PWM.PC1  0.11443454 1.1212392 0.007771449 14.724994 0.0e+00
PWM.PC5  0.06183618 1.0637881 0.018793243  3.290341 1.0e-03
PWM.PC6 -0.12405699 0.8833295 0.020524549 -6.044322 1.5e-09
```

The analysis of deviance table here compares a model with just 'other' PCs in it to the model with both PWM and 'other' feature PCs. If significant, it shows that PWM PCs have an association with targetting independent of that due to 'other' features.

```
Analysis of Deviance Table

Model 1: 'other' feature PCs
Model 2: Both PWM PCs and 'other' PCs
  Resid. Df Resid. Dev   Df Deviance P(>|Chi|)
1      5776    2154.08
2      5773    2076.43    3    77.65 9.798e-17
```

And here is the table of coefficients with both PWM PCs and 'other' PCs. Substantial reductions in the magnitude of these coefficients from the earlier tables show that the added variables in the model may 'confound' the association seen earlier.

```
                coef exp(coef)    se(coef)          z       p
PWM.PC1  0.051067773 1.0523942 0.010577462  4.8279797 1.4e-06
PWM.PC5  0.070250980 1.0727774 0.019940280  3.5230688 4.3e-04
PWM.PC6 -0.126977197 0.8807538 0.021384522 -5.9378085 2.9e-09
X.PC1    0.057702627 1.0593999 0.007012645  8.2283684 2.2e-16
X.PC8   -0.083724888 0.9196842 0.024341907 -3.4395370 5.8e-04
X.PC32  -0.136748551 0.8721895 0.047156246 -2.8999033 3.7e-03
X.PC5   -0.007687977 0.9923415 0.020117231 -0.3821588 7.0e-01
X.PC40   0.023467224 1.0237447 0.054517097  0.4304562 6.7e-01
X.PC44   0.204215136 1.2265620 0.061522485  3.3193577 9.0e-04
X.PC9    0.124635022 1.1327350 0.034882422  3.5730037 3.5e-04
X.PC33   0.146274536 1.1575139 0.050510455  2.8959259 3.8e-03
X.PC89  -0.496562419 0.6086192 0.140568313 -3.5325345 4.1e-04
X.PC7    0.021015863 1.0212383 0.019852088  1.0586223 2.9e-01
```

The following interaction terms exceed the FDR= 0.05 cutoff. The labelling scheme used is like this "PWM.PC1:X.PC10" refers to the first principal component for the PWMs and the tenth principal component for the 'other' features.

Note that by comparing one of these coefficients to the coefficients of the two main effects, a sense of whether the effect is synergystic or antagonistic can be obtained.

	coef	exp(coef)	se(coef)	z	p
PWM.PC1:X.PC1	-0.00466	0.995	0.000929	-5.02	5.2e-07

3.3.3 HIVmGAG

The table of coefficients for the other features is:

	coef	exp(coef)	se(coef)	z	p
X.PC6	-0.24113708	0.7857339	0.025176241	-9.577962	0.0e+00
X.PC37	0.41539528	1.5149695	0.060008086	6.922322	4.4e-12
X.PC1	0.02429313	1.0245906	0.007670503	3.167084	1.5e-03
X.PC29	0.27535321	1.3169958	0.052371631	5.257679	1.5e-07
X.PC2	-0.06979988	0.9325804	0.013441924	-5.192700	2.1e-07
X.PC11	0.21936166	1.2452816	0.033450515	6.557796	5.5e-11
X.PC19	0.25736755	1.2935205	0.049531145	5.196075	2.0e-07
X.PC46	-0.35224630	0.7031069	0.069567987	-5.063339	4.1e-07
X.PC40	-0.31100431	0.7327107	0.067584352	-4.601721	4.2e-06
X.PC3	0.05674996	1.0583911	0.015317328	3.704952	2.1e-04

The analysis of deviance table here compares a model with just PWM PCs in it to the model with both PWM and 'other' feature PCs. If significant, it shows that 'other' feature have an association with targetting independent of that due to PWMs.

```
Analysis of Deviance Table

Model 1: PWM PCs
Model 2: Both PWM PCs and 'other' PCs
  Resid. Df Resid. Dev   Df Deviance P(>|Chi|)
1      5421    2224.21
2      5411    1912.89   10   311.31 6.291e-61
```

The table of coefficients for the PWMs is:

	coef	exp(coef)	se(coef)	z	p
PWM.PC1	-0.1027376	0.9023637	0.01187279	-8.653203	0.0e+00
PWM.PC2	-0.1461638	0.8640162	0.01766963	-8.272032	1.1e-16

The analysis of deviance table here compares a model with just 'other' PCs in it to the model with both PWM and 'other' feature PCs. If significant, it shows that PWM PCs have an association with targetting independent of that due to 'other' features.

```
Analysis of Deviance Table

Model 1: ‘other’ feature PCs
Model 2: Both PWM PCs and ‘other’ PCs
  Resid. Df Resid. Dev   Df Deviance P(>|Chi|)
1      5413    2001.48
2      5411    1912.89    2    88.59 5.802e-20
```

And here is the table of coefficients with both PWM PCs and ‘other’ PCs. Substantial reductions in the magnitude of these coefficients from the earlier tables show that the added variables in the model may ’confound’ the association seen earlier.

	coef	exp(coef)	se(coef)	z	p
PWM.PC1	-0.12199117	0.8851562	0.015018554	-8.122697	4.4e-16
PWM.PC2	-0.09066381	0.9133247	0.019073148	-4.753479	2.0e-06
X.PC6	-0.22827352	0.7959065	0.025460908	-8.965647	0.0e+00
X.PC37	0.24083524	1.2723114	0.063979487	3.764257	1.7e-04
X.PC1	0.05907561	1.0608554	0.008989873	6.571351	5.0e-11
X.PC29	0.25502037	1.2904879	0.053171986	4.796142	1.6e-06
X.PC2	-0.06238308	0.9395229	0.013774043	-4.529032	5.9e-06
X.PC11	0.19931667	1.2205684	0.034201158	5.827775	5.6e-09
X.PC19	0.21703982	1.2423936	0.050530946	4.295186	1.7e-05
X.PC46	-0.22169903	0.8011565	0.072037666	-3.077543	2.1e-03
X.PC40	-0.24625877	0.7817199	0.069787570	-3.528691	4.2e-04
X.PC3	0.04397784	1.0449592	0.015397058	2.856250	4.3e-03

Tests for interactions between the PWM principal components and the ’other’ features principal components yielded no results beyond the FDR= 0.05 cutoff.

3.3.4 HIVmIN

The table of coefficients for the other features is:

	coef	exp(coef)	se(coef)	z	p
X.PC1	0.0906938361	1.0949337	0.007243716	12.52034702	0.00000
X.PC5	-0.0782493330	0.9247338	0.021193848	-3.69207774	0.00022
X.PC8	-0.0649838712	0.9370826	0.026212205	-2.47914552	0.01300
X.PC50	-0.1797638327	0.8354675	0.075245205	-2.38904039	0.01700
X.PC2	-0.0527715489	0.9485967	0.015406178	-3.42534992	0.00061
X.PC11	0.0568199898	1.0584653	0.029383959	1.93370777	0.05300
X.PC20	0.0643815829	1.0664993	0.048575366	1.32539574	0.19000
X.PC115	-0.9431543229	0.3893976	0.365269285	-2.58207947	0.00980
X.PC13	-0.0161645620	0.9839654	0.036339210	-0.44482426	0.66000
X.PC4	-0.0006980472	0.9993022	0.020905763	-0.03339018	0.97000

The analysis of deviance table here compares a model with just PWM PCs in it to the model with both PWM and ‘other’ feature PCs. If significant, it

shows that ‘other’ feature have an association with targetting independent of that due to PWMs.

```
Analysis of Deviance Table

Model 1: PWM PCs
Model 2: Both PWM PCs and ‘other’ PCs
  Resid. Df Resid. Dev   Df Deviance P(>|Chi|)
1      3849    1525.47
2      3839    1375.48   10   149.99 3.742e-27
```

The table of coefficients for the PWMs is:

	coef	exp(coef)	se(coef)	z	p
PWM.PC1	0.1132407	1.119901	0.009142378	12.38635	0

The analysis of deviance table here compares a model with just ‘other’ PCs in it to the model with both PWM and ‘other’ feature PCs. If significant, it shows that PWM PCs have an association with targetting independent of that due to ‘other’ features.

```
Analysis of Deviance Table

Model 1: ‘other’ feature PCs
Model 2: Both PWM PCs and ‘other’ PCs
  Resid. Df Resid. Dev   Df Deviance P(>|Chi|)
1      3840    1388.04
2      3839    1375.48    1    12.56 0.0003933
```

And here is the table of coefficients with both PWM PCs and ‘other’ PCs. Substantial reductions in the magnitude of these coefficients from the earlier tables show that the added variables in the model may ’confound’ the association seen earlier.

	coef	exp(coef)	se(coef)	z	p
PWM.PC1	0.04625266	1.0473390	0.012998071	3.5584247	3.7e-04
X.PC1	0.07145993	1.0740751	0.009044848	7.9006230	2.8e-15
X.PC5	-0.05304008	0.9483420	0.022423867	-2.3653405	1.8e-02
X.PC8	-0.06053021	0.9412653	0.026217074	-2.3088087	2.1e-02
X.PC50	-0.21854943	0.8036838	0.076662497	-2.8507998	4.4e-03
X.PC2	-0.05314464	0.9482429	0.015540236	-3.4198089	6.3e-04
X.PC11	0.06036726	1.0622266	0.029410250	2.0525925	4.0e-02
X.PC20	0.06571456	1.0679218	0.048770933	1.3474125	1.8e-01
X.PC115	-1.01096037	0.3638694	0.368076390	-2.7466048	6.0e-03
X.PC13	-0.01287512	0.9872074	0.036413456	-0.3535815	7.2e-01
X.PC4	0.02138073	1.0216109	0.022143458	0.9655553	3.3e-01

Tests for interactions between the PWM principal components and the ’other’ features principal components yielded no results beyond the FDR= 0.05 cutoff.

3.3.5 MLVPuro

The table of coefficients for the other features is:

	coef	exp(coef)	se(coef)	z	p
X.PC1	0.10745707	1.1134431	0.006270947	17.135702	0.0e+00
X.PC8	-0.15629130	0.8553100	0.024280543	-6.436895	1.2e-10
X.PC5	-0.09303074	0.9111655	0.019370253	-4.802763	1.6e-06
X.PC7	0.06294051	1.0649635	0.018973214	3.317335	9.1e-04
X.PC44	0.17222775	1.1879484	0.059470529	2.896018	3.8e-03
X.PC40	0.11199346	1.1185055	0.051474808	2.175694	3.0e-02
X.PC9	0.08825468	1.0922663	0.030089814	2.933042	3.4e-03
X.PC62	0.28162093	1.3252763	0.074586826	3.775746	1.6e-04
X.PC2	-0.05427592	0.9471707	0.015507321	-3.500019	4.7e-04
X.PC6	0.03743869	1.0381483	0.025194500	1.485986	1.4e-01

The analysis of deviance table here compares a model with just PWM PCs in it to the model with both PWM and 'other' feature PCs. If significant, it shows that 'other' feature have an association with targetting independent of that due to PWMs.

```
Analysis of Deviance Table

Model 1: PWM PCs
Model 2: Both PWM PCs and 'other' PCs
  Resid. Df Resid. Dev   Df Deviance P(>|Chi|)
1      5969    2096.43
2      5959    1804.32   10   292.11 7.214e-57
```

The table of coefficients for the PWMs is:

	coef	exp(coef)	se(coef)	z	p
PWM.PC1	0.15508369	1.1677557	0.00779116	19.905083	0.0e+00
PWM.PC5	0.05619431	1.0578032	0.01883252	2.983898	2.8e-03
PWM.PC6	-0.08363610	0.9197659	0.02124185	-3.937326	8.2e-05
PWM.PC8	-0.06646704	0.9356938	0.02175407	-3.055384	2.2e-03

The analysis of deviance table here compares a model with just 'other' PCs in it to the model with both PWM and 'other' feature PCs. If significant, it shows that PWM PCs have an association with targetting independent of that due to 'other' features.

```
Analysis of Deviance Table

Model 1: 'other' feature PCs
Model 2: Both PWM PCs and 'other' PCs
  Resid. Df Resid. Dev   Df Deviance P(>|Chi|)
1      5963    1882.30
2      5959    1804.32    4    77.98 4.666e-16
```

And here is the table of coefficients with both PWM PCs and 'other' PCs. Substantial reductions in the magnitude of these coefficients from the earlier tables show that the added variables in the model may 'confound' the association seen earlier.

	coef	exp(coef)	se(coef)	z	p
PWM.PC1	0.06989877	1.0723996	0.010704141	6.5300685	6.6e-11
PWM.PC5	0.07336511	1.0761234	0.021035899	3.4876147	4.9e-04
PWM.PC6	-0.09020705	0.9137420	0.023114362	-3.9026408	9.5e-05
PWM.PC8	-0.04695708	0.9541283	0.024560516	-1.9118930	5.6e-02
X.PC1	0.08514917	1.0888795	0.007262137	11.7250836	0.0e+00
X.PC8	-0.14369559	0.8661514	0.024617564	-5.8371164	5.3e-09
X.PC5	-0.03168751	0.9688093	0.020816885	-1.5222022	1.3e-01
X.PC7	0.04864058	1.0498429	0.019289715	2.5215810	1.2e-02
X.PC44	0.11023908	1.1165450	0.060345335	1.8268037	6.8e-02
X.PC40	0.05981605	1.0616412	0.052706748	1.1348841	2.6e-01
X.PC9	0.09888035	1.1039342	0.030448985	3.2474105	1.2e-03
X.PC62	0.26484046	1.3032230	0.074822400	3.5395878	4.0e-04
X.PC2	-0.05982555	0.9419288	0.015988734	-3.7417317	1.8e-04
X.PC6	0.01571003	1.0158341	0.025617105	0.6132633	5.4e-01

The following interaction terms exceed the FDR= 0.05 cutoff. The labelling scheme used is like this "PWM.PC1:X.PC10" refers to the first principal component for the PWMs and the tenth principal component for the 'other' features. Note that by comparing one of these coefficients to the coefficients of the two main effects, a sense of whether the effect is synergystic or antagonistic can be obtained.

	coef	exp(coef)	se(coef)	z	p
PWM.PC1:X.PC1	-0.00488	0.995	0.000876	-5.57	2.5e-08
PWM.PC1:X.PC8	0.00945	1.010	0.003350	2.82	4.8e-03
PWM.PC5:X.PC7	0.02290	1.020	0.008370	2.74	6.1e-03

References

[Dabney et al.,] Dabney, A., Storey, J. D., and with assistance from Gregory R. Warnes. *qvalue: Q-value estimation for false discovery rate control.* R package version 1.1.

[Mitchell et al., 2004] Mitchell, R. S., Beitzel, B. F., Schroder, A. R. W., Shinn, P., Chen, H., Berry, C. C., Ecker, J. R., and Bushman, F. D. (2004). Retroviral DNA integration: ASLV, HIV, and MLV show distinct target site preferences. *PLoS Biol*, 2(8):E234.

[Therneau and Lumley,] Therneau, T. and Lumley, T. *survival: Survival analysis, including penalised likelihood.* R package version 2.18, S original by Terry Therneau and ported by Thomas Lumley.
