# Supplementary material for: Retroviral DNA Integration: Viral and Cellular Determinants of Target-Site Selection
Source: PLoS Pathog. 2006 Jun 23;2(6):e60. doi: 10.1371/journal.ppat.0020060 (PMC1480600; doi:10.1371/journal.ppat.0020060)
Supplement: Supplementary file 3 [file ppat.0020060.sd003.pdf]

Similarity of Integration Sites of Different Integration Complexes

Charles C. Berry

Contents

1 Introduction **1**

2 Data Used **2**

3 Training the Predictive Algorithm **3**

4 Results **4**
4.1 Classification of the Training Data 4
4.2 Classification of Genomic Locations 10

A Appendix: Variable Names Described **12**

1 Introduction

This report studies data created by Mary Lewinski and others in and associated with the laboratory of Frederic Bushman at the University of Pennsylvania.

The aim of this report is to assess the tendency of different integration complexes — in this case retroviral vectors composed of HIV, MLV, or elements of both — to select particular genomic loci as favored integration targets. Previously, it has been shown that HIV and MLV favor different sites for integration. It is of particular interest to characterize the degree to which different integration complexes favor the same or different sites.

With a very large number of integration events (of the order of 10 per base by complex or 150,000,000,000 for this study), this could be done directly by counting the number of events at each genomic locus for each integration complex, then comparing the counts. Integration complexes that tend to share high counts at some loci and share low counts at other loci presumably share features that govern integration targetting. On the other hand, integration complexes whose counts do not correlate in this fashion presumably do not share features relevant to integration targetting.

Practically, it is not now possible to collect such large samples of integration events, so another strategy is needed. A number of genomic features (e.g. local GC percentage, exons, actively transcribing genes) have been identified that correlate with integration of HIV and/or MLV. By applying a machine learning algorithm to a sample of integration events, a function can be created that maps the local genomic features to a vector of probabilities of integration of different types.

The overall strategy used here is to characterize the integration intensity for different integration complexes at particular genomic positions according to a collection of *features* associated with each position. This will be done by using a supervised machine learning algorithm to form a classification rule. Once this rule is in hand it is of interest to see which complexes are easily distinguished based on their genomic features — and therefore have profiles of genetic features that are distinct — and which are not easily distinguished.

2 Data Used

The number of integration sites used for each integration complex used summarized here:

```
               count
HIVmIN           350
HIVPuro          524
HIVmGAGmIN       526
HIVmGAG          493
MLVPuro          543
matchedControl  2436
```

The 'matchedControl' sites are randomly sampled *in silico* from the genome (according to Chromosome, Position on the chromosome, and Strand), but at a similar distance from the restriction site used in these experiments as one of the actual insertion sites. A second set of randomly sampled sites is later used to compare the predicted targets of the different integration complexes.

The features used are are as follows:

In Gene The position is or is not in a gene according to each of these annotation schemes: Acembly, RefSeq, UniGene, and GenScan. (4 features)

In Exon The position is or is not in an exon according to each of these annotation schemes: Acembly, RefSeq, UniGene, and GenScan. (4 features)

Gene Density The density of genes according to each of the annotation schemes and within windows with widths of ±50,000 bases, ±100,000 bases, ±250,000 bases, ±500,000 bases, and ±1,000,000 bases. Each density is the number of genes counted divided by the number of bases.(20 features)

Density of Expressed Genes Using the genes on the Affymetrix Hu-133a GeneChip, the number of such genes, the numbers whose 'average difference score' were characterized as at least 'low' (above the median), at least 'medium' (above the 75th percentile), and at least 'high'(above teh 87.5^{th} percentile) were counted in windows of widths ±12,500 bases, ±25,000 bases,±50,000 bases,±125,000 bases,±250,000 bases,±500,000 bases,±1,000,000 bases,±2,000,000 bases,±4,000,000 bases,±8,000,000 bases and ±16,000,000 bases.(44 features)

GC percentage In running windows of width 5120 bases. Derived from the file `gc5Base.txt.gz` from the GoldenPath website (`http://hgdownload.cse.ucsc.edu/goldenPath/hg17/database/`). (1 feature)

In CpG Island In or not in a CpG island according to the `cpgIsland.txt.gz` from the GoldePath website. (1 feature)

CpG Island Neighborhoods Whether site is within ±500, ±2,500, ±5,000, ±12,500, or ±25,000 bases of a CpG island. (5 features)

CpG Island Density The density of CpG islands in windows of widths ±12,500,±25,000, ±50,000, ±125,000, , ±250,000, ±500,000, ±1,000,000, ±2,000,000, ±4,000,000, ±8,000,000, and ±16,000,000 bases. Each density is the number of island counted divided by the number of bases. (11 features)

DNAse I Site Density The number of DNAse I sites in windows of widths ±500, ±1000, and ±5000 bases. Each density is the number of sites counted divided by the number of bases. (3 features)

Juxtaposition of Transcription Start/Stop Sites Various measures are used: The width of the gene if the insertion site is in one or else the width of the intergeneic region, the fraction of that distance from/to the nearest gene boundary, the absolute distance to the nearest transcription start site, and the signed distance to the nearset start site (negative values preceed start sites). These are calculated for each of these annotation schemes: Acembly, RefSeq, UniGene, and GenScan. (16 features)

3 Training the Predictive Algorithm

The algorithm used in this report is the *randomForest* algorithm of Breiman [Breiman, 2001]. It was chosen for its proven ability to perform classification (including estimation of posterior probabilities) on data sets with modest numbers of observations but with many variables. In addition, accurate estimates of classification error and measures of the marginal importance of classifying variables are obtained as a by-product of the *bagging* algorithm used by the procedure.

Roughly speaking the algorithm grows a collection of binary trees — splitting the data recursively to create branches in which the one or a few classes

dominate. The use of resampling procedures for selecting the objects to be classified and the predictor variables for which candidate splits are allowed generates a collection of trees. These sampling procedures counter the tendency to overfit the training data and are responsible for the excellent performance of the randomForest algorithm. Each tree in the collection will produce a predicted class for a vector of predictor variables, and the 'votes' of the collection of trees is used to assign the ultimate prediction.

The implementation used is that of Liaw and Wiener [Liaw and Wiener, 2002] ('randomForest' version 4.5-12 — an R package [R Development Core Team, 2005]) and is based on the Fortran code of Leo Breiman and Adele Cutler.

The default values for options in the `randomForest` function that govern the approach to training the classifier were used with the exception of the `cutoff` values which were proportional to one for the integration complexes and to 5 for the match cotrol sequences. These values were chosen for the `cutoff` vector to balance the approximately fivefold larger number of matched control sequences. In subsequent runs, the number of variables screened for each candidate split was varied to half and twice the default number; there was little effect on the classification results and those results are not reported here.

4 Results

4.1 Classification of the Training Data

The classifications made on the training dataset are summarized in the following table:

	HIVmIN	HIVPuro	HIVmGAGmIN	HIVmGAG	MLVPuro	matchedControl
HIVmIN	52	48	58	43	96	53
HIVPuro	11	246	39	150	48	30
HIVmGAGmIN	25	61	149	81	161	49
HIVmGAG	10	110	44	223	51	55
MLVPuro	24	70	130	62	223	34
matchedControl	34	104	249	295	203	1551

As is evident from inspection of the table, matched control sites are usually not mistaken as *bona fide* integration sites (1551 of 2436 were correctly classified). Close inspection also shows that several rows have roughly similar patterns of counts. HIVMGAGmIN and MLVPuro are similar as are HIVPuro and HIVmGAG. This is more easily seen by displaying the table as a color map and clustering the rows and columns. This was performed by percentaging each row in the table above and applying the R `heatmap` function. (The clustering uses Euclidean distance between the resulting rows of percents and the 'complete' clustering method). The text in each cell shows the actual row percentage obtained from the table above. The color map is arranged so that green corresponds to values less than 16.7%, red to values more than 16.7%, and black to ≈ 16.7%. The results appear in the following graph:

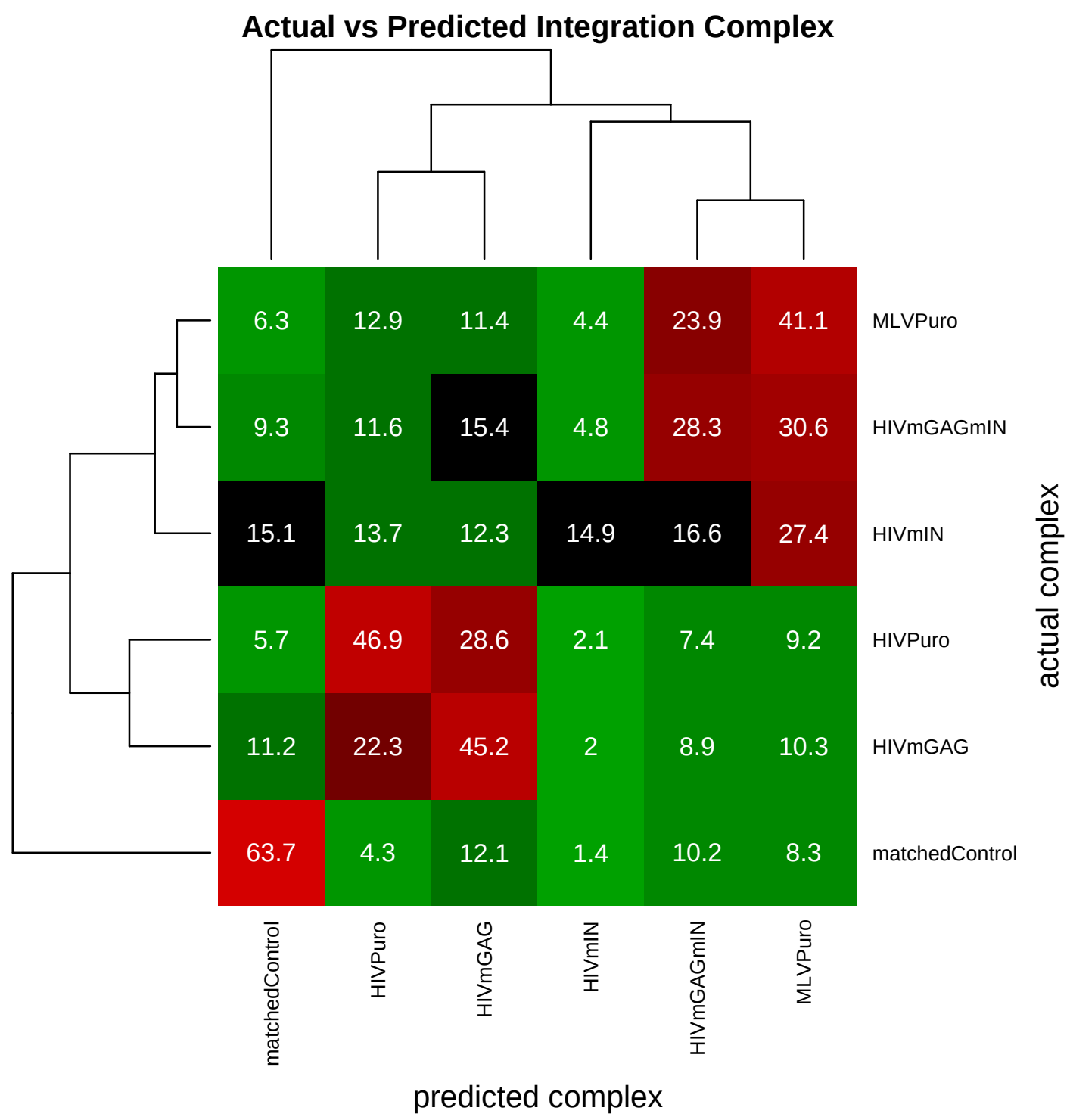

The merges that form clusters are the same for both the rows and the columns. As one might expect the MLVPuro - HIVmGAGmIN pair merge and joined with HIVmIN, the HIVmGAGmIN - HIVmIN pair merge, and the matched controls are last to merge.

Assessing Variable Importance The marginal importance of a variable can be judged by randomly permuting its values among those available for splitting a tree at a given point and computing the decrease in accuracy (i.e. the fraction correctly classified). When many variables are available as candidate predictors, it often happens that there is redundancy among them and that omitting one will not adversely affect the classifacation accuracy. The difference in accuracy between a classification based on just one variable and that based on its permutation is shown in the first column of the table below. The values in the second column reflect the decrease in accuracy for each variable when included in the full collection of variables The summary measure is the mean decrease in accuracy averaged over all 6 classes. Each class receives equal weight in this computation even though sample sizes may vary.

	Sole Predictor Variable	One of Many
acembly.genes	0.000	0.002
acembly.exon	0.000	0.001
refGene.genes	0.000	0.006

refGene.exon	0.001	0.000
genScan.genes	0.000	0.000
genScan.exon	0.000	0.000
uniGene.genes	0.000	0.002
uniGene.exon	0.000	0.000
ace.100k	0.042	0.013
ace.200k	0.056	0.015
ace.500k	0.068	0.018
ace.1M	0.073	0.011
ace.2M	0.076	0.009
ref.100k	0.019	0.002
ref.200k	0.020	0.002
ref.500k	0.039	0.005
ref.1M	0.045	0.003
ref.2M	0.053	0.004
uni.100k	0.026	0.008
uni.200k	0.038	0.011
uni.500k	0.043	0.012
uni.1M	0.053	0.009
uni.2M	0.068	0.007
gen.100k	0.004	0.000
gen.200k	0.011	0.001
gen.500k	0.013	0.001
gen.1M	0.022	0.002
gen.2M	0.034	0.002
onco.100k	0.000	0.000
onco.200k	0.001	0.000
onco.500k	0.005	0.000
onco.1M	0.002	0.000
onco.2M	0.008	0.000
onco.4M	0.020	0.000
plus.oncogenes	0.001	0.000
minus.oncogenes	0.000	0.000
dens.25k	0.019	0.001
low.ex.25k	0.026	0.000
med.ex.25k	0.019	0.000
high.ex.25k	0.012	0.000
dens.50k	0.029	0.001
low.ex.50k	0.034	0.001
med.ex.50k	0.026	0.000
high.ex.50k	0.021	0.000
dens.100k	0.039	0.001
low.ex.100k	0.042	0.004
med.ex.100k	0.029	0.001
high.ex.100k	0.022	0.000
dens.250k	0.064	0.003

low.ex.250k	0.065	0.007
med.ex.250k	0.046	0.002
high.ex.250k	0.021	0.001
dens.500k	0.075	0.004
low.ex.500k	0.079	0.009
med.ex.500k	0.060	0.005
high.ex.500k	0.034	0.002
dens.1M	0.087	0.003
low.ex.1M	0.086	0.006
med.ex.1M	0.080	0.003
high.ex.1M	0.053	0.001
dens.2M	0.091	0.005
low.ex.2M	0.087	0.005
med.ex.2M	0.081	0.004
high.ex.2M	0.066	0.003
dens.4M	0.081	0.003
low.ex.4M	0.083	0.004
med.ex.4M	0.086	0.004
high.ex.4M	0.071	0.003
dens.8M	0.069	0.003
low.ex.8M	0.071	0.003
med.ex.8M	0.081	0.003
high.ex.8M	0.075	0.003
dens.16M	0.080	0.002
low.ex.16M	0.072	0.002
med.ex.16M	0.076	0.003
high.ex.16M	0.081	0.002
dens.32M	0.061	0.002
low.ex.32M	0.065	0.002
med.ex.32M	0.081	0.001
high.ex.32M	0.072	0.002
gcpct	0.035	0.009
is.cpg	0.003	0.000
cpg.1k	0.031	0.001
cpg.5k	0.038	0.001
cpg.10k	0.001	0.001
cpg.25k	0.000	0.000
cpg.50k	0.000	0.001
cpg.dens.25k	0.002	0.001
cpg.dens.50k	0.004	0.001
cpg.dens.100k	0.003	0.002
cpg.dens.250k	0.005	0.003
cpg.dens.500k	0.010	0.002
cpg.dens.1M	0.006	0.002
cpg.dens.2M	0.021	0.001
cpg.dens.4M	0.022	0.001

cpg.dens.8M	0.026	0.001
cpg.dens.16M	0.021	0.001
cpg.dens.32M	0.023	0.001
dnaseI.1k	0.010	0.000
dnaseI.2k	0.020	0.000
dnaseI.10k	0.032	0.001
dnaseI.25k	0.037	0.002
dnaseI.50k	0.039	0.004
dnaseI.100k	0.047	0.008
dnaseI.1M	0.063	0.008
dnaseI.5M	0.063	0.005
dnaseI.20M	0.034	0.002
boundary.dx.ace	0.003	0.001
start.dx.ace	0.009	0.001
signed.dx.ace	0.039	0.008
general.wd.ace	0.026	0.003
boundary.dx.ref	0.007	0.001
start.dx.ref	0.013	0.001
signed.dx.ref	0.057	0.012
general.wd.ref	0.079	0.011
boundary.dx.gens	0.000	0.000
start.dx.gens	0.003	0.001
signed.dx.gens	0.010	0.001
general.wd.gens	0.037	0.001
boundary.dx.uni	0.005	0.000
start.dx.uni	0.009	0.001
signed.dx.uni	0.042	0.008
general.wd.uni	0.065	0.006

See appendix A for explanation of variable names

As can be seen many of the variables that have values of 0.05 or larger when considered as a sole predictor variable usually have values of less than 0.01 when considered along with the other predictor variables. No doubt this is due to the considerable redundancy between the variables in this collection.

The values in the table below reflect the decreases in accuracy for each class (i.e. the fraction correctly classified in each class) for selected members of a the full collection of variables. Each selected variable is among the top 5 for at least one of the classes of integration complex or matched control)

	HIVmIN	HIVPuro	HIVmGAGmIN	HIVmGAG	MLVPuro	matchedControl
general.wd.ref	0.016	0.036	0.016	0.019	0.024	0.000
signed.dx.ref	0.014	0.039	0.015	0.026	0.021	0.001
gcpct	0.011	0.008	0.008	0.023	0.024	0.002
signed.dx.uni	0.008	0.022	0.009	0.013	0.012	0.003
signed.dx.ace	0.007	0.024	0.008	0.016	0.012	0.001
refGene.genes	0.006	0.024	0.008	0.017	0.009	-0.002

general.wd.uni	0.006	0.009	0.007	0.006	0.012	0.004
ace.500k	0.004	0.012	0.001	0.000	0.005	0.031
ace.200k	0.003	0.014	0.000	0.003	0.004	0.025
ace.100k	0.002	0.013	0.000	0.004	0.000	0.023
uni.500k	0.004	0.005	0.001	-0.002	0.004	0.022
ace.1M	0.003	0.008	0.000	0.000	0.003	0.019

See appendix A for explanation of variable names

As is evident the more important variables for distinguishing between integration sites and matched control sites tend not to be so important for distinguishing among the different integration events (and vice versa). In particular, the juxtaposition of transcription start sites and being in a gene are at or near the top of each list for the integration sites, and `gcpct` is important for `MLVPuro`. However,this does not hold for the matched control sites. Measures of gene density are most important for classifying matched controls, but not for discriminating among integration complexes.

It is interesting to consider whether these 12 variables classify the integration complexes as well as the full collection of 123 variables used earlier. Here is the table of classification results.

	HIVmIN	HIVPuro	HIVmGAGmIN	HIVmGAG	MLVPuro	matchedControl
HIVmIN	46	50	74	40	98	42
HIVPuro	18	236	34	162	58	16
HIVmGAGmIN	36	66	156	67	166	35
HIVmGAG	16	128	57	208	33	51
MLVPuro	40	82	148	43	203	27
matchedControl	75	110	250	266	185	1550

These results differ only slightly from those seen above. It is probably worth taking another look at the variable importance measure now that many redundant variables have been eliminated. This table shows the revised variable importance measures:

	HIVmIN	HIVPuro	HIVmGAGmIN	HIVmGAG	MLVPuro	matchedControl
general.wd.ref	0.034	0.059	0.034	0.036	0.038	0.007
signed.dx.ref	0.041	0.093	0.043	0.069	0.059	0.008
gcpct	0.030	0.019	0.019	0.051	0.062	0.004
signed.dx.uni	0.018	0.024	0.010	0.017	0.011	0.019
signed.dx.ace	0.017	0.045	0.017	0.016	0.017	0.009
refGene.genes	0.027	0.088	0.037	0.068	0.039	-0.018
general.wd.uni	0.016	0.021	0.015	0.008	0.025	0.016
ace.500k	0.021	0.042	0.008	0.007	0.017	0.065
ace.200k	0.018	0.043	0.009	0.004	0.016	0.062
ace.100k	0.012	0.035	0.003	0.016	0.004	0.052
uni.500k	0.012	0.022	0.002	0.010	0.017	0.050
ace.1M	0.016	0.062	-0.003	0.009	0.024	0.043

See appendix A for explanation of variable names

Again, different variables tend to register as important for discriminating among integration comlpexes as opposed to discriminating between them and the match control sites.

4.2 Classification of Genomic Locations

To get a better sense of the relation between the attractiveness of a genomic location to different integration complexes the weighted votes for a set of random matched control loci are heuristically taken as predicted probabilities. These are computed applying the randomForest classification trees developed earlier to a new sample of genomic loci and tallying the votes for each integration complex or matched control.

The fractions of votes for each integration complex (or the matched control) are presented as scatterplots in the figure below. The numbers in the lower triangle are the correlations among the votes.

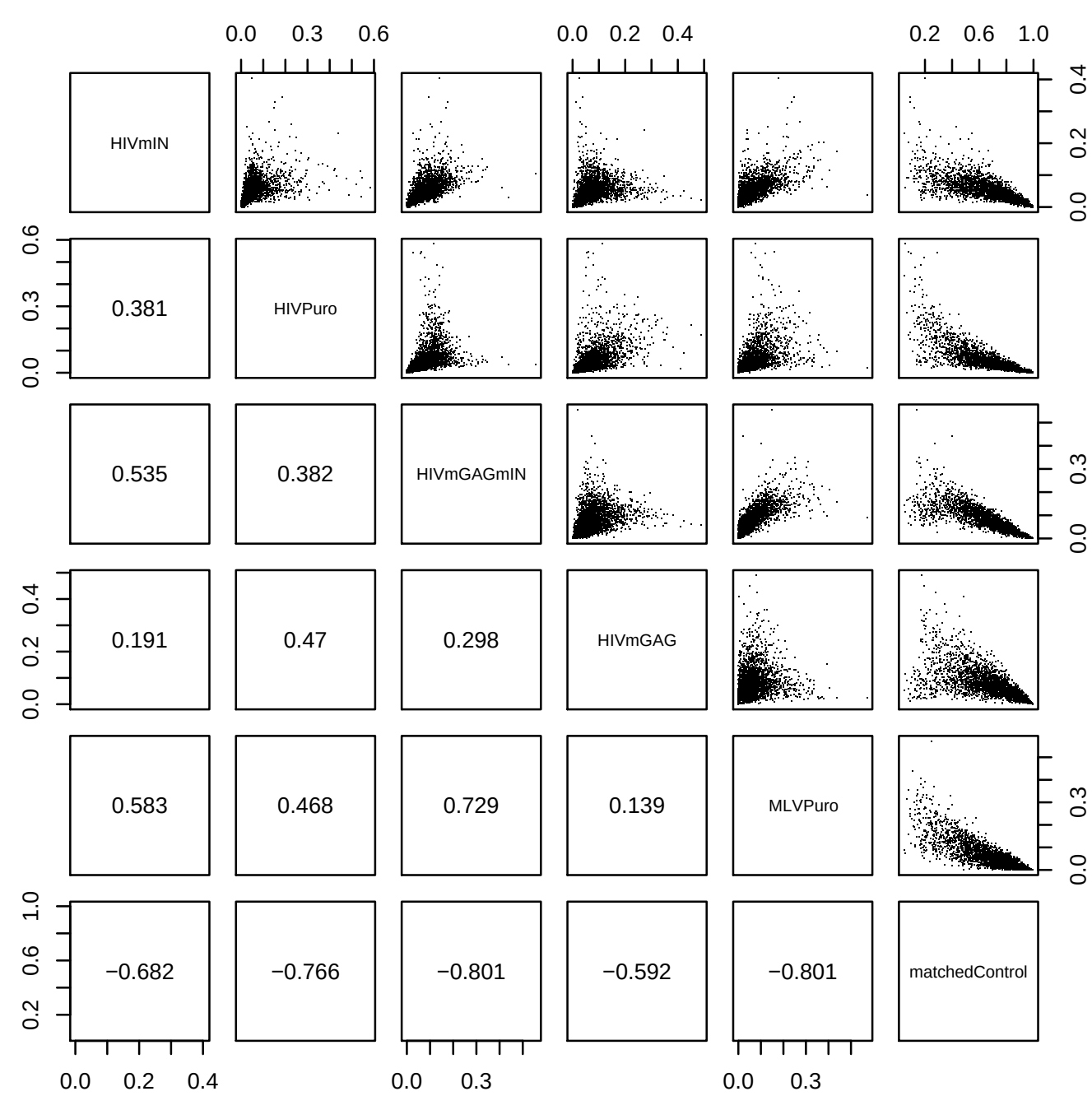

When viewed on-screen as a pdf document, it is helpful to *zoom in* on this plot to better reveal the locations of extreme data points. As might have been guessed from the earlier results, when there are many votes for the matched control, the number of votes for any of the integration complexes tends to be low. Among the integration complexes the number of votes for HIVmIN tends

to be highest when the number is also high for HIVPuro, and the number tends to be high for MLVPuro when the number for HIVmGAGmIN is also high.

It may also be helpful to restrict attention to only votes for integration complexes. In the following plots, the fraction of votes for each integration complex is divided by the fraction of votes for all integration complexes.

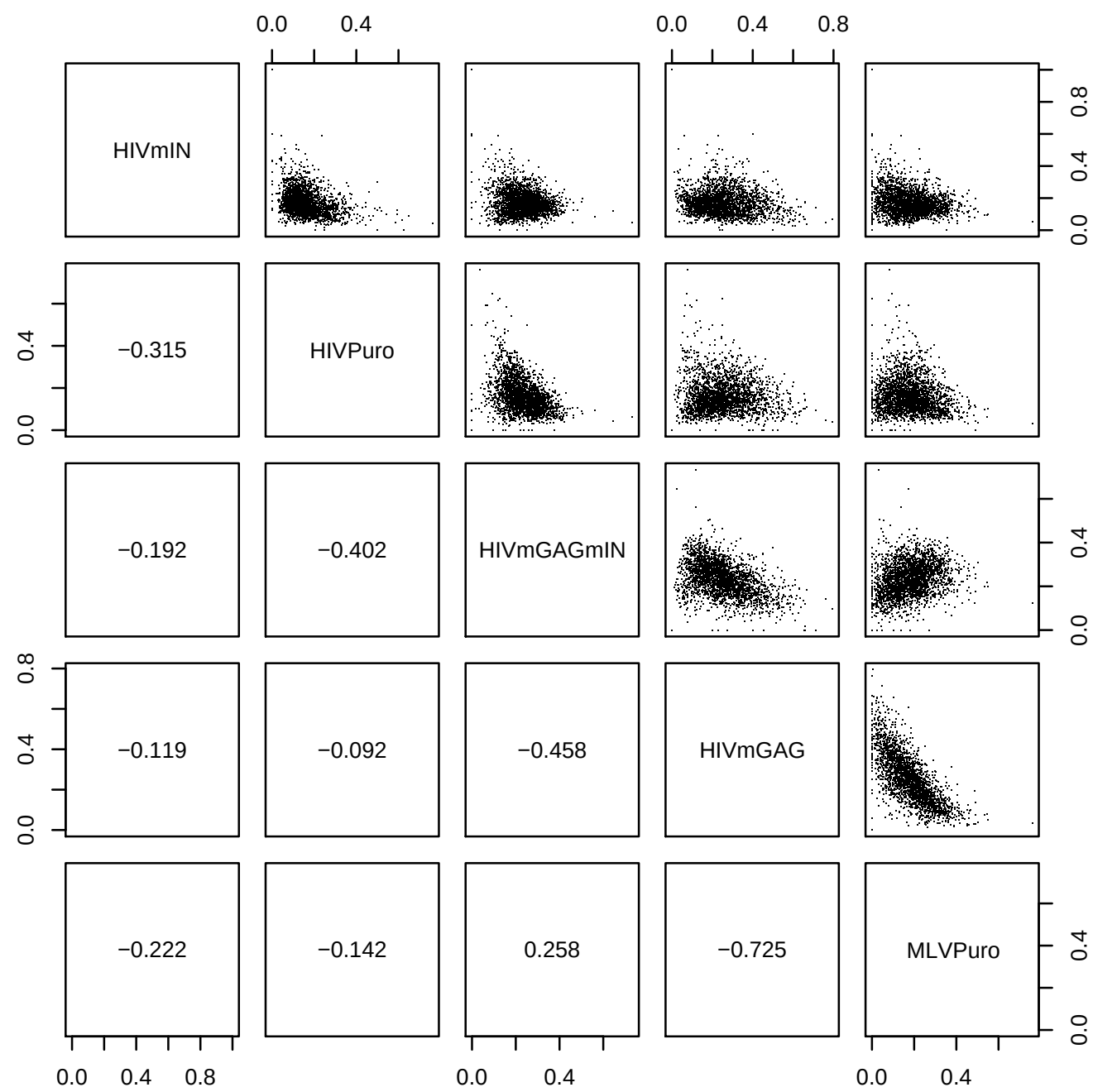

Only MLVPuro and HIVmGAGmIN have a positive correlation, which implies that similar genomic sites are favored by those two integration complexes. On the other hand the most negative correlation is between MLVPuro and HIVmGAG.

Similar observations can also be made from inspecting a dendrogram of suitably constructed distances between the vote counts for the different complexes. Taking p_{ij}, the fraction of votes for integration complex category j for genomic location i, a normalization is performed:

$$\tilde{p}_{ij} = p_{ij} / \sum_i p_{ij}$$

Then the symmetrized Kullback-Leibler distance is calculated as

$$KL(j,k) = \sum_i \tilde{p}_{ij} \log(\tilde{p}_{ij}/\tilde{p}_{ik}) + \tilde{p}_{ik} \log(\tilde{p}_{ik}/\tilde{p}_{ij})$$

Hierarchical clustering of these distances is presented in the following graph:

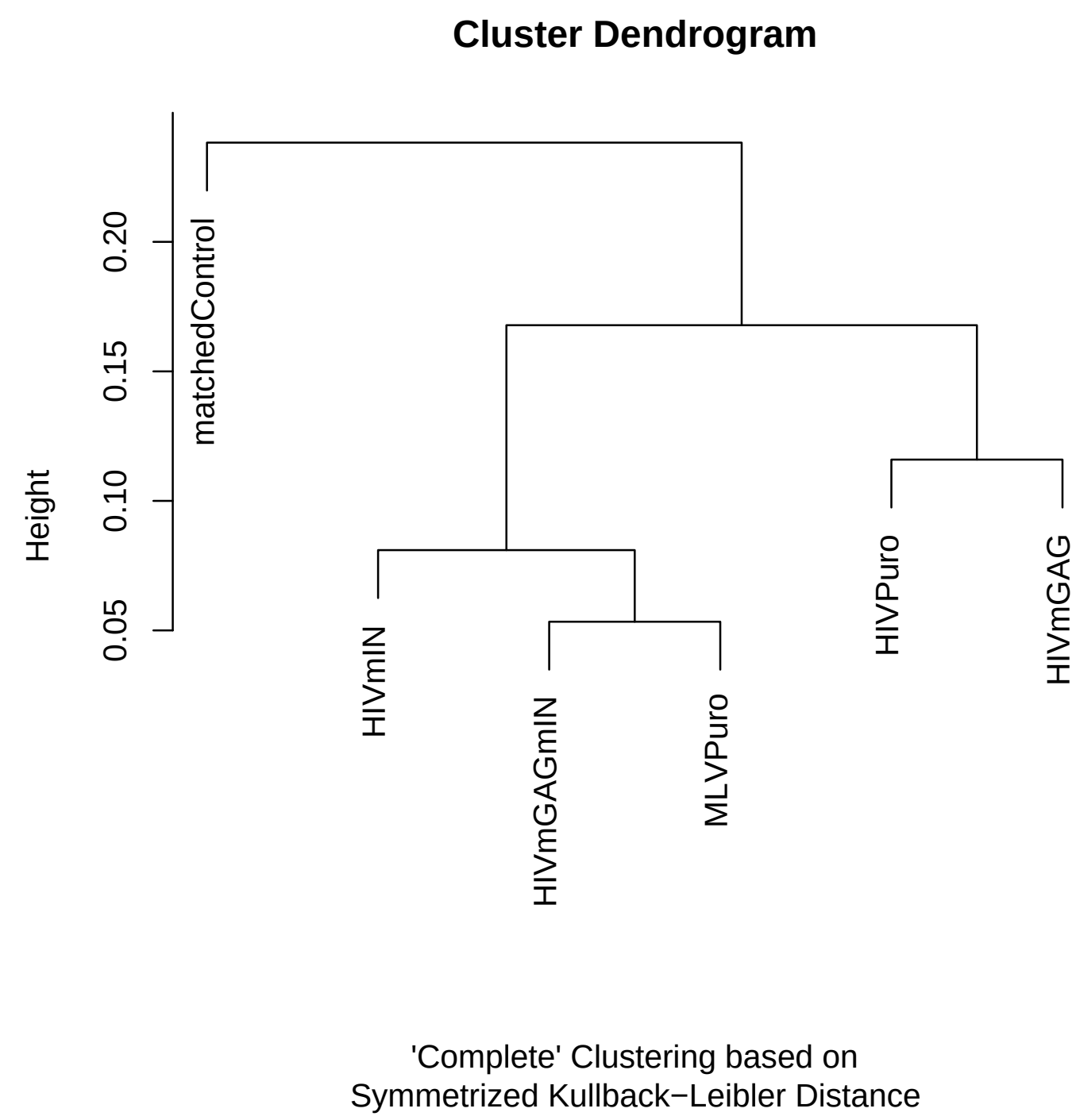

The same nodes are merged to form this tree as were merged earlier on.

References

[Breiman, 2001] Breiman, L. (2001). Random forests. *Machine Learning*, 45(1).

[Liaw and Wiener, 2002] Liaw, A. and Wiener, M. (2002). Classification and regression by randomforest. *R News*, 2(3):18–22.

[R Development Core Team, 2005] R Development Core Team (2005). *R: A language and environment for statistical computing*. R Foundation for Statistical Computing, Vienna, Austria. ISBN 3-900051-07-0.

A Appendix: Variable Names Described

The following table describes the variable names used in this document.

variable name	description
acembly.genes	In acembly gene
acembly.exon	In acembly exon
refGene.genes	In refGene gene
refGene.exon	In refGene exon
genScan.genes	In genScan gene

genScan.exon	In genScan exon
uniGene.genes	In uniGene gene
uniGene.exon	In uniGene exon
ace.100k	acembly Density in 100kBase window
ace.200k	acembly Density in 200kBase window
ace.500k	acembly Density in 500kBase window
ace.1M	acembly Density in 1MBase window
ace.2M	acembly Density in 2MBase window
ref.100k	refGene Density in 100kBase window
ref.200k	refGene Density in 200kBase window
ref.500k	refGene Density in 500kBase window
ref.1M	refGene Density in 1MBase window
ref.2M	refGene Density in 2MBase window
uni.100k	uniGene Density in 100kBase window
uni.200k	uniGene Density in 200kBase window
uni.500k	uniGene Density in 500kBase window
uni.1M	uniGene Density in 1MBase window
uni.2M	uniGene Density in 2MBase window
gen.100k	genScan Density in 100kBase window
gen.200k	genScan Density in 200kBase window
gen.500k	genScan Density in 500kBase window
gen.1M	genScan Density in 1MBase window
gen.2M	genScan Density in 2MBase window
onco.100k	onco.100k
onco.200k	onco.200k
onco.500k	onco.500k
onco.1M	onco.1M
onco.2M	onco.2M
onco.4M	onco.4M
plus.oncogenes	plus.oncogene
minus.oncogenes	minus.oncogene
dens.25k	Affymetrix Gene Density in 25kBase window
low.ex.25k	Density of Affymetrix Expr > 50%ile in 25kBase window
med.ex.25k	Density of Affymetrix Expr > 75%ile in 25kBase window
high.ex.25k	Density of Affymetrix Expr > 87.5%ile in 25kBase window
dens.50k	Affymetrix Gene Density in 50kBase window
low.ex.50k	Density of Affymetrix Expr > 50%ile in 50kBase window
med.ex.50k	Density of Affymetrix Expr > 75%ile in 50kBase window
high.ex.50k	Density of Affymetrix Expr > 87.5%ile in 50kBase window
dens.100k	Affymetrix Gene Density in 100kBase window
low.ex.100k	Density of Affymetrix Expr > 50%ile in 100kBase window
med.ex.100k	Density of Affymetrix Expr > 75%ile in 100kBase window
high.ex.100k	Density of Affymetrix Expr > 87.5%ile in 100kBase window
dens.250k	Affymetrix Gene Density in 250kBase window
low.ex.250k	Density of Affymetrix Expr > 50%ile in 250kBase window
med.ex.250k	Density of Affymetrix Expr > 75%ile in 250kBase window
high.ex.250k	Density of Affymetrix Expr > 87.5%ile in 250kBase window
dens.500k	Affymetrix Gene Density in 500kBase window
low.ex.500k	Density of Affymetrix Expr > 50%ile in 500kBase window
med.ex.500k	Density of Affymetrix Expr > 75%ile in 500kBase window

high.ex.500k	Density of Affymetrix Expr > 87.5%ile in 500kBase window
dens.1M	Affymetrix Gene Density in 1MBase window
low.ex.1M	Density of Affymetrix Expr > 50%ile in 1MBase window
med.ex.1M	Density of Affymetrix Expr > 75%ile in 1MBase window
high.ex.1M	Density of Affymetrix Expr > 87.5%ile in 1MBase window
dens.2M	Affymetrix Gene Density in 2MBase window
low.ex.2M	Density of Affymetrix Expr > 50%ile in 2MBase window
med.ex.2M	Density of Affymetrix Expr > 75%ile in 2MBase window
high.ex.2M	Density of Affymetrix Expr > 87.5%ile in 2MBase window
dens.4M	Affymetrix Gene Density in 4MBase window
low.ex.4M	Density of Affymetrix Expr > 50%ile in 4MBase window
med.ex.4M	Density of Affymetrix Expr > 75%ile in 4MBase window
high.ex.4M	Density of Affymetrix Expr > 87.5%ile in 4MBase window
dens.8M	Affymetrix Gene Density in 8MBase window
low.ex.8M	Density of Affymetrix Expr > 50%ile in 8MBase window
med.ex.8M	Density of Affymetrix Expr > 75%ile in 8MBase window
high.ex.8M	Density of Affymetrix Expr > 87.5%ile in 8MBase window
dens.16M	Affymetrix Gene Density in 16MBase window
low.ex.16M	Density of Affymetrix Expr > 50%ile in 16MBase window
med.ex.16M	Density of Affymetrix Expr > 75%ile in 16MBase window
high.ex.16M	Density of Affymetrix Expr > 87.5%ile in 16MBase window
dens.32M	Affymetrix Gene Density in 32MBase window
low.ex.32M	Density of Affymetrix Expr > 50%ile in 32MBase window
med.ex.32M	Density of Affymetrix Expr > 75%ile in 32MBase window
high.ex.32M	Density of Affymetrix Expr > 87.5%ile in 32MBase window
gcpct	GC percent in 5120 Base window
is.cpg	In CpG Island
cpg.1k	CpG Island in 1kBase window
cpg.5k	CpG Island in 5kBase window
cpg.10k	CpG Island in 10kBase window
cpg.25k	CpG Island in 25kBase window
cpg.50k	CpG Island in 50kBase window
cpg.dens.25k	CpG Island Density in 25kBase window
cpg.dens.50k	CpG Island Density in 50kBase window
cpg.dens.100k	CpG Island Density in 100kBase window
cpg.dens.250k	CpG Island Density in 250kBase window
cpg.dens.500k	CpG Island Density in 500kBase window
cpg.dens.1M	CpG Island Density in 1MBase window
cpg.dens.2M	CpG Island Density in 2MBase window
cpg.dens.4M	CpG Island Density in 4MBase window
cpg.dens.8M	CpG Island Density in 8MBase window
cpg.dens.16M	CpG Island Density in 16MBase window
cpg.dens.32M	CpG Island Density in 32MBase window
dnaseI.1k	Density of DNase Sites in 1kBase window
dnaseI.2k	Density of DNase Sites in 2kBase window
dnaseI.10k	Density of DNase Sites in 10kBase window
dnaseI.25k	Density of DNase Sites in 25kBase window
dnaseI.50k	Density of DNase Sites in 50kBase window
dnaseI.100k	Density of DNase Sites in 100kBase window
dnaseI.1M	Density of DNase Sites in 1MBase window

dnaseI.5M	Density of DNase Sites in 5MBase window
dnaseI.20M	Density of DNase Sites in 20MBase window
boundary.dx.ace	Distance to Nearest acembly Gene Boundary
start.dx.ace	Distance to Nearest acembly Gene Start
signed.dx.ace	Distance from(+)/to(-) Nearest acembly Gene Start
general.wd.ace	Width of acembly (Inter-)Gene (Region)
boundary.dx.ref	Distance to Nearest refGene Gene Boundary
start.dx.ref	Distance to Nearest refGene Gene Start
signed.dx.ref	Distance from(+)/to(-) Nearest refGene Gene Start
general.wd.ref	Width of refGene (Inter-)Gene (Region)
boundary.dx.gens	Distance to Nearest genScans Gene Boundary
start.dx.gens	Distance to Nearest genScans Gene Start
signed.dx.gens	Distance from(+)/to(-) Nearest genScans Gene Start
general.wd.gens	Width of genScans (Inter-)Gene (Region)
boundary.dx.uni	Distance to Nearest uniGene Gene Boundary
start.dx.uni	Distance to Nearest uniGene Gene Start
signed.dx.uni	Distance from(+)/to(-) Nearest uniGene Gene Start
general.wd.uni	Width of uniGene (Inter-)Gene (Region)
