# Supplementary material for: Retroviral DNA Integration: Viral and Cellular Determinants of Target-Site Selection
Source: PLoS Pathog. 2006 Jun 23;2(6):e60. doi: 10.1371/journal.ppat.0020060 (PMC1480600; doi:10.1371/journal.ppat.0020060)
Supplement: Supplementary file 4 [file ppat.0020060.sd004.pdf]

Association of Genomic Features with Integration: Unselected vs. Puromycin-Selected HIV

April 21, 2006

Contents

1 Introduction **3**

2 Preference for Genes **4**
2.1 Acembly Genes 4
2.2 refGenes 6
2.3 ensGenes 8
2.4 genScan Genes 10
2.5 uniGenes 12

3 CpG Island Neighborhoods **14**
3.1 1 kilobase neighborhoods 14
3.2 5 kilobase neighborhoods 15
3.3 10 kilobase neighborhoods 16
3.4 25 kilobase neighborhoods 17
3.5 50 kilobase neighborhoods 18

4 Gene Density, Expression 'Density', and CpG Island Density **19**
4.1 25 kiloBase Window 20
4.2 50 kiloBase Window 26
4.3 100 kiloBase Window 31
4.4 250 kiloBase Window 36
4.5 500 kiloBase Window 41
4.6 1 megaBase Window 46
4.7 2 megaBase Window 51
4.8 4 megaBase Window 56
4.9 8 megaBase Window 61
4.10 16 megaBase Window 66
4.11 32 megaBase Window 71

5 Juxtaposition with Gene Start and End Positions **76**
5.1 Acembly Annotations 76
5.2 RefSeq Annotations 80
5.3 genScan Annotations 84
5.4 uniGene Annotations 88

6 GC content **92**

7 Cytobands **93**

1 Introduction

In this document, I examine the association of integration siting with various genomic features.

The numbers are shown below:

```
Origin.of.data.set
HIV-Burgess      HIVPuro
290              525
```

The distribution of relative frequency of insertions across the chromosomes is given in this barplot:

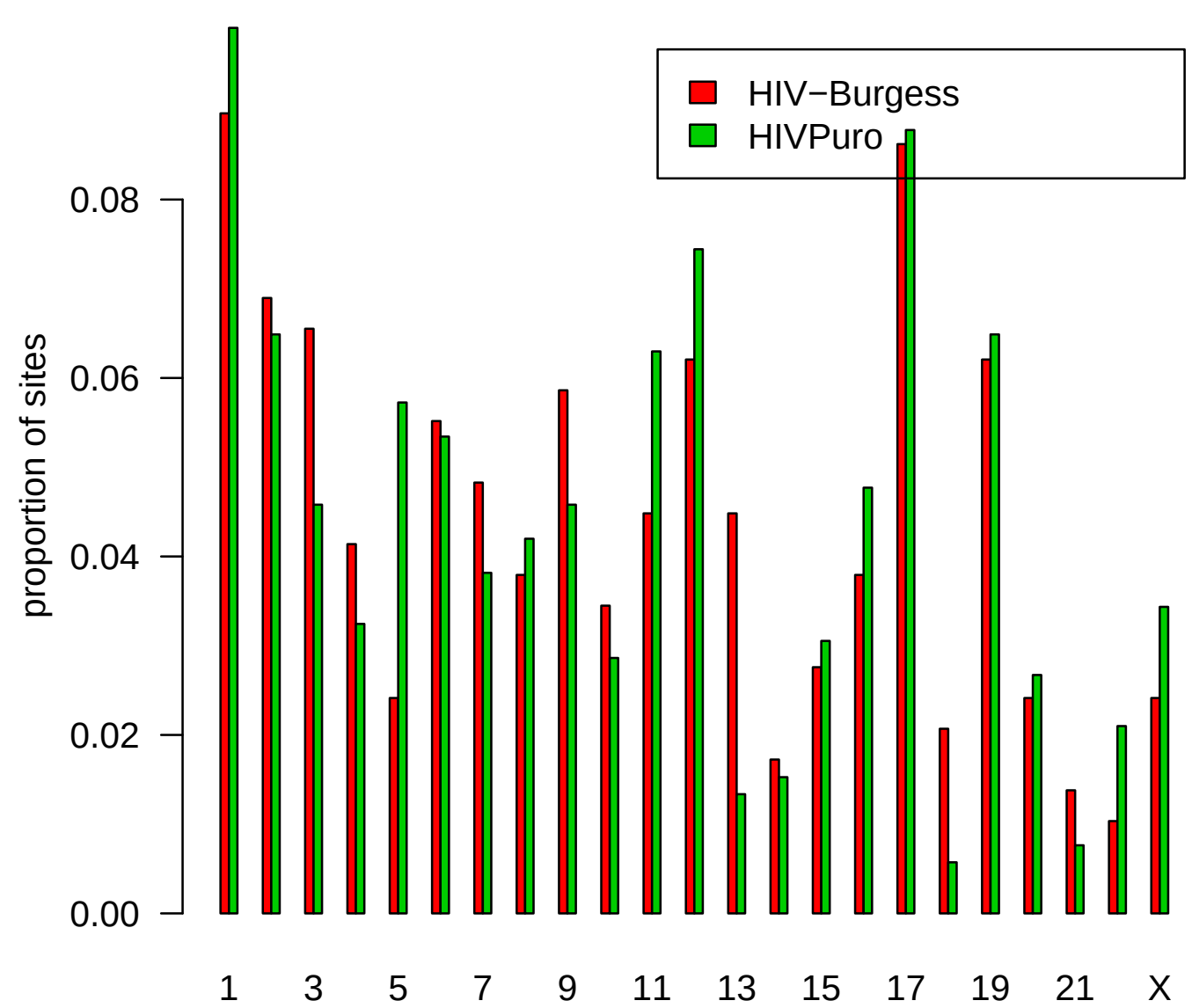

Are there chromosomes that are particularly favored for integration by one group over the other? This was tested for statistical significance. The test performed used the likelihood ratio statistic for the logistic regression model (reviewed in [1]) as implemented by the `glm` function of R using the `binomial` family. The null hypothesis tested is the ratio of true integration events in the two groups is constant across all chromosomes. This test attains a p-value of 0.35886.

2 Preference for Genes

2.1 Acembly Genes

Here we examine the relative preference that integration events in the two groups have for genes. In the following plot we show the relative frequency of integrations in genes according to the 'Acembly' annotation. The bars grouped over the label "In Gene" give the relative frequency of integration events (compared to control sites) between bases located within Acembly gene annotations, while the label "Not in Gene" give the relative frequency of integration events (compared to control sites) between bases not located within Acembly gene annotations.

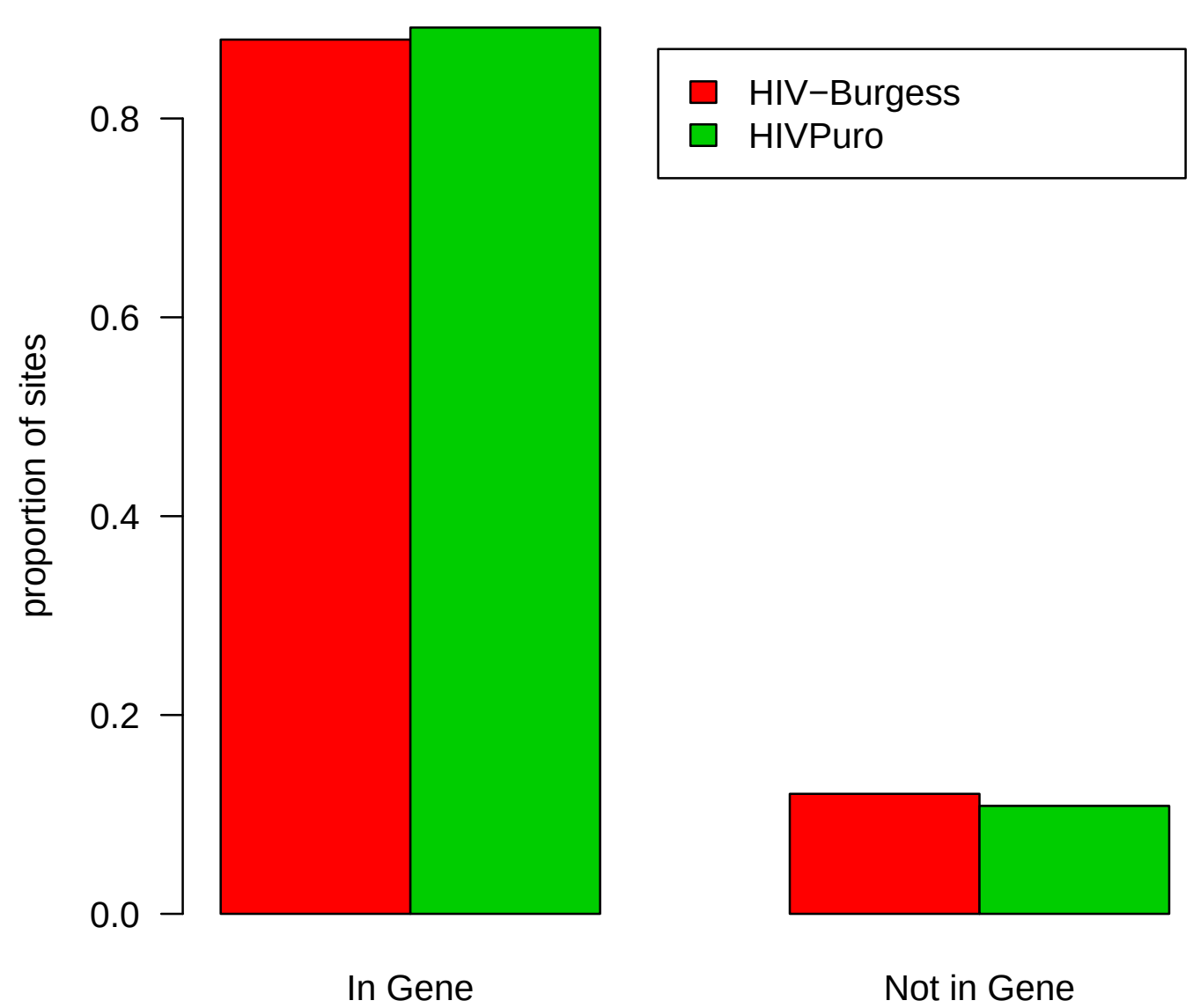

Is there is a difference in the tendency for insertions to occur in genes? A formal test of significance yields a p-value of 0.60225. In the following plot we show the relative frequency of insertions in exons according to the 'Acembly' annotation The bars grouped over the label "In Exon" give the relative frequency of integration events (compared to control sites) between bases located in exons according to the Acembly annotation, while the label "Not in Exon" give the relative frequency of integration events (compared to control sites) between bases not located in exons according to the Acembly gene annotation.

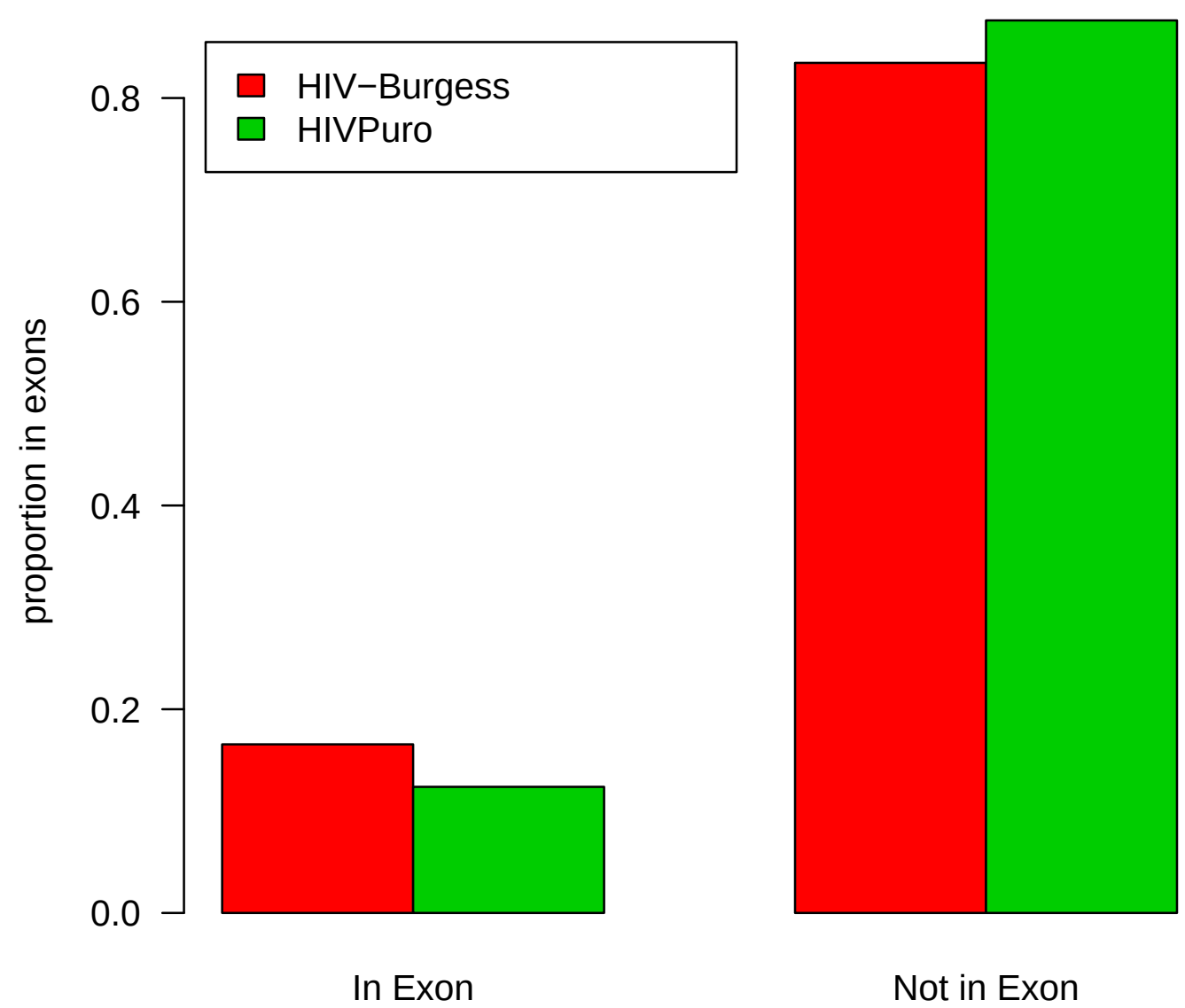

Here is the table of coefficients of the log ratio of intensities along with their standard errors, z statistics, and p-values:

```
              coef    se      z      p
(Intercept)  0.488 0.215  2.270 0.0231
in.gene      0.179 0.231  0.772 0.4400
in.exon     -0.363 0.209 -1.740 0.0819
```

The model on which these coefficients are based include terms for whether the site is in a gene or not. Thus, the effect shown as 'in.exon' is net of that due to being in a gene. Note that in the barplot above the 'Not in Exon' bars include the both introns and intergenic regions, so the impression given by the table may differ from that for the barplot.

2.2 refGenes

Here we examine the relative preference that insertions of the two types have for genes. In the following plot we show the relative frequency of insertions in genes according to the 'refGene' annotation.

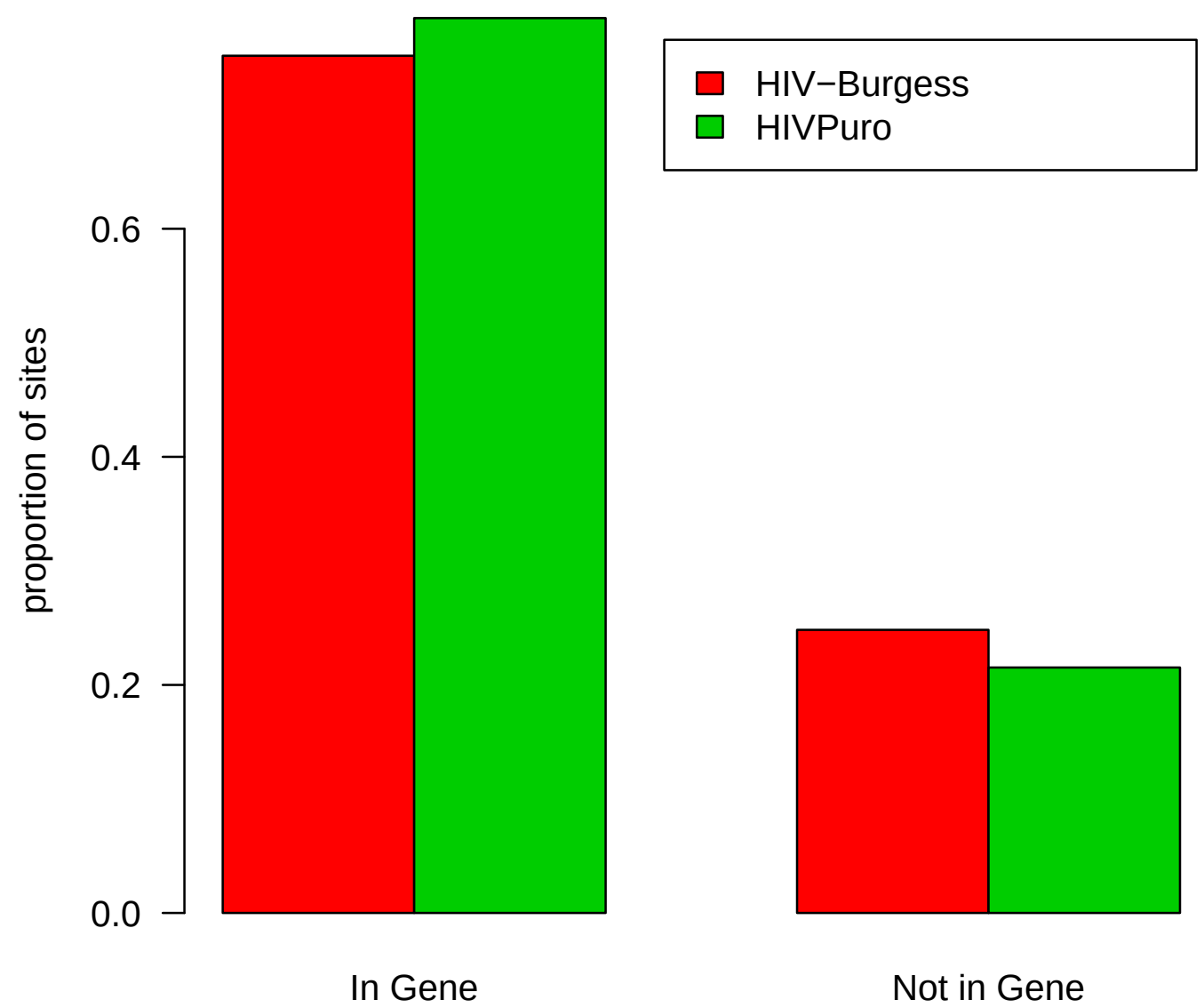

Is there is a tendency for insertions to occur in genes? A formal test of significance yields a p-value of 0.28323.

In the following plot we show the relative frequency of insertions in exons according to the 'refGene' annotation.

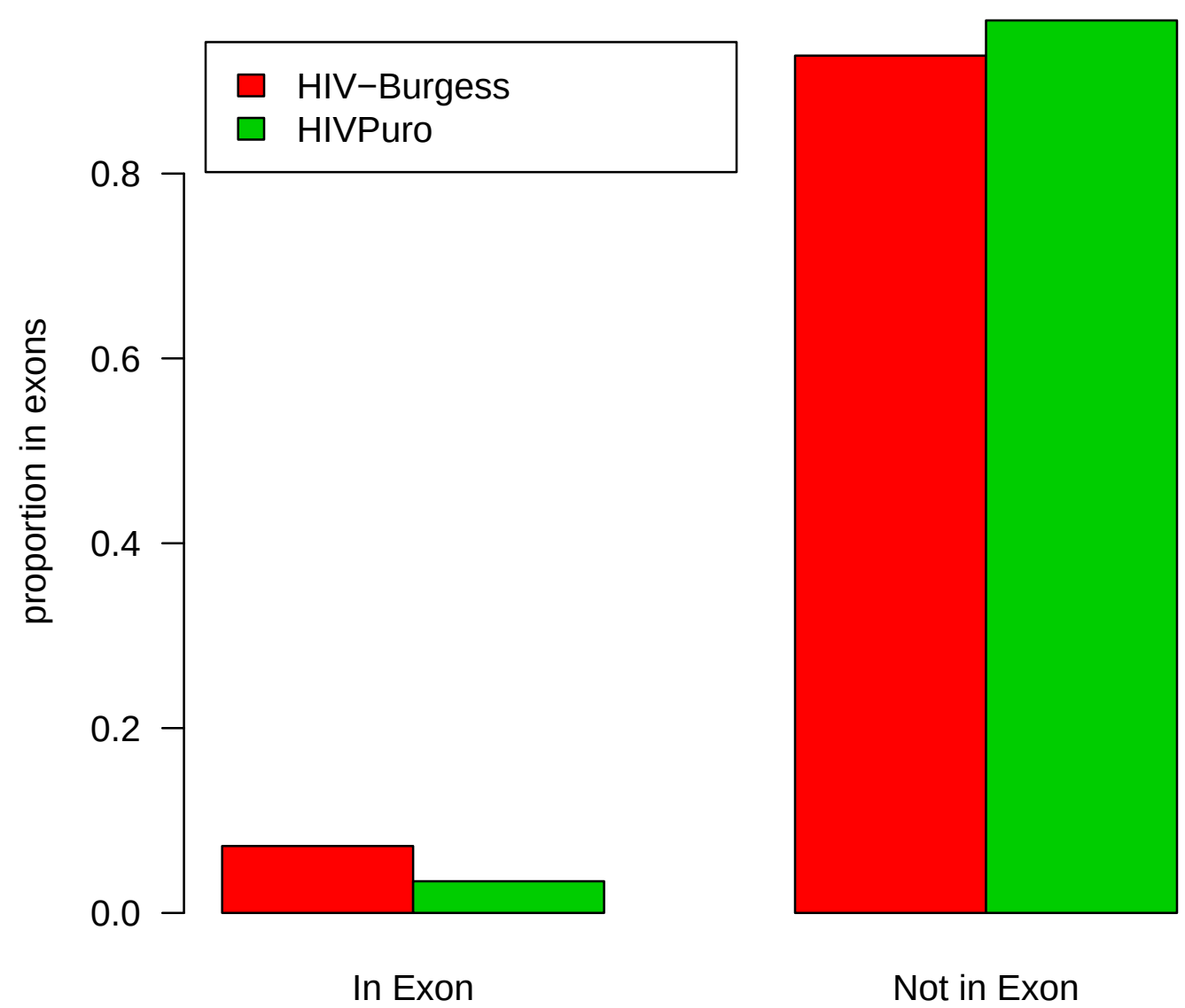

Here is the table of coefficients of the log ratio of intensities for along with their standard errors, z statistics, and p-values:

```
              coef    se     z      p
(Intercept)  0.451 0.151  2.99 0.0028
in.gene      0.242 0.174  1.39 0.1640
in.exon     -0.847 0.333 -2.55 0.0109
```

The model on which these coefficients are based include terms for whether the site is in a gene or not. Thus, the effect shown as 'in.exon' is net of that due to being in a gene. Note that in the barplot above the 'Not in Exon' bars include the both introns and intergenic regions, so the impression given by the table may differ from that for the barplot.

2.3 ensGenes

Here we examine the relative preference that insertions of the two types have for genes. In the following plot we show the relative frequency of insertions in genes according to the 'ensGene' annotation.

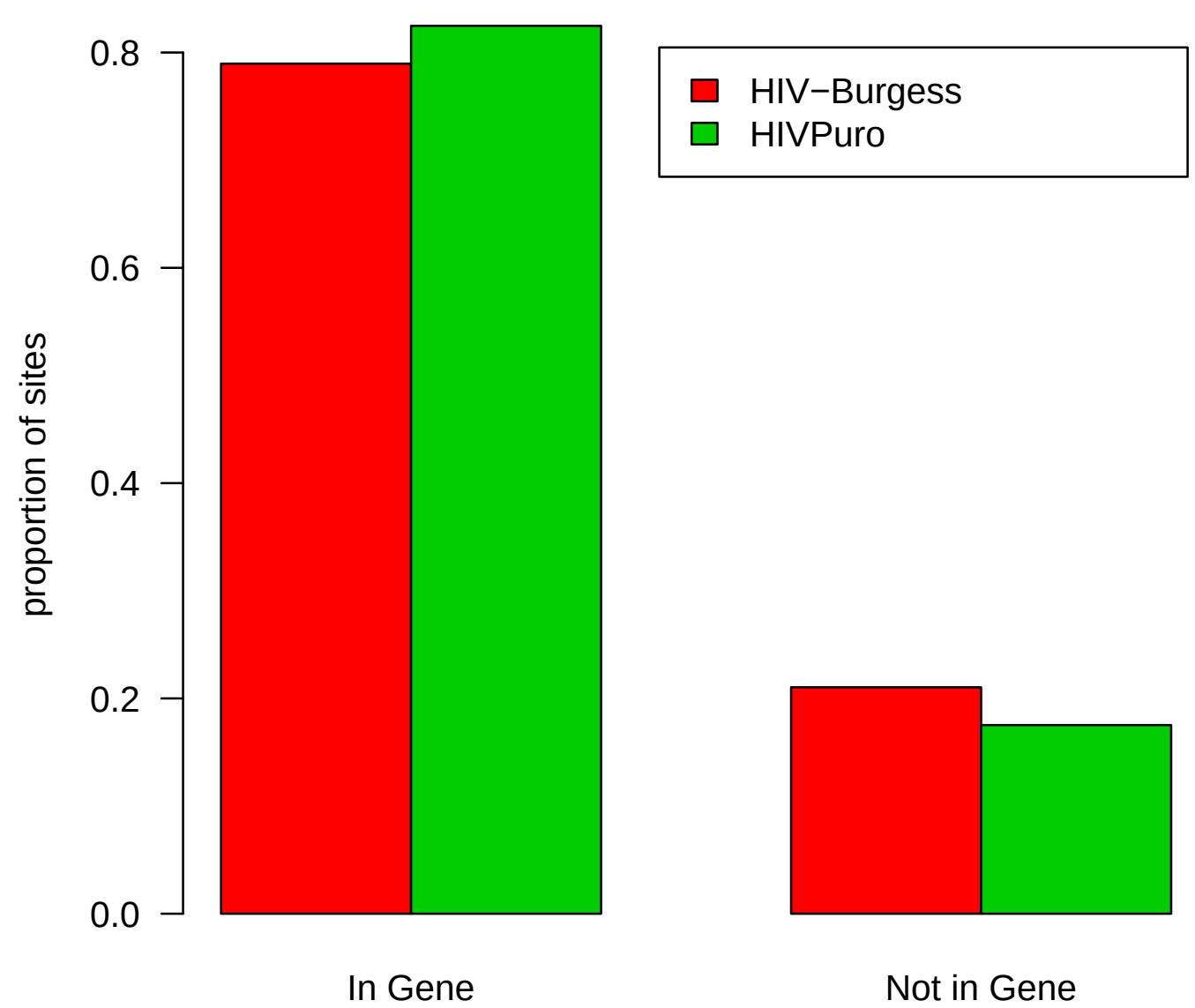

Is there is a tendency for insertions to occur in genes? A formal test of significance yields a p-value of 0.22203.

In the following plot we show the relative frequency of insertions in exons according to the 'ensGene' annotation.

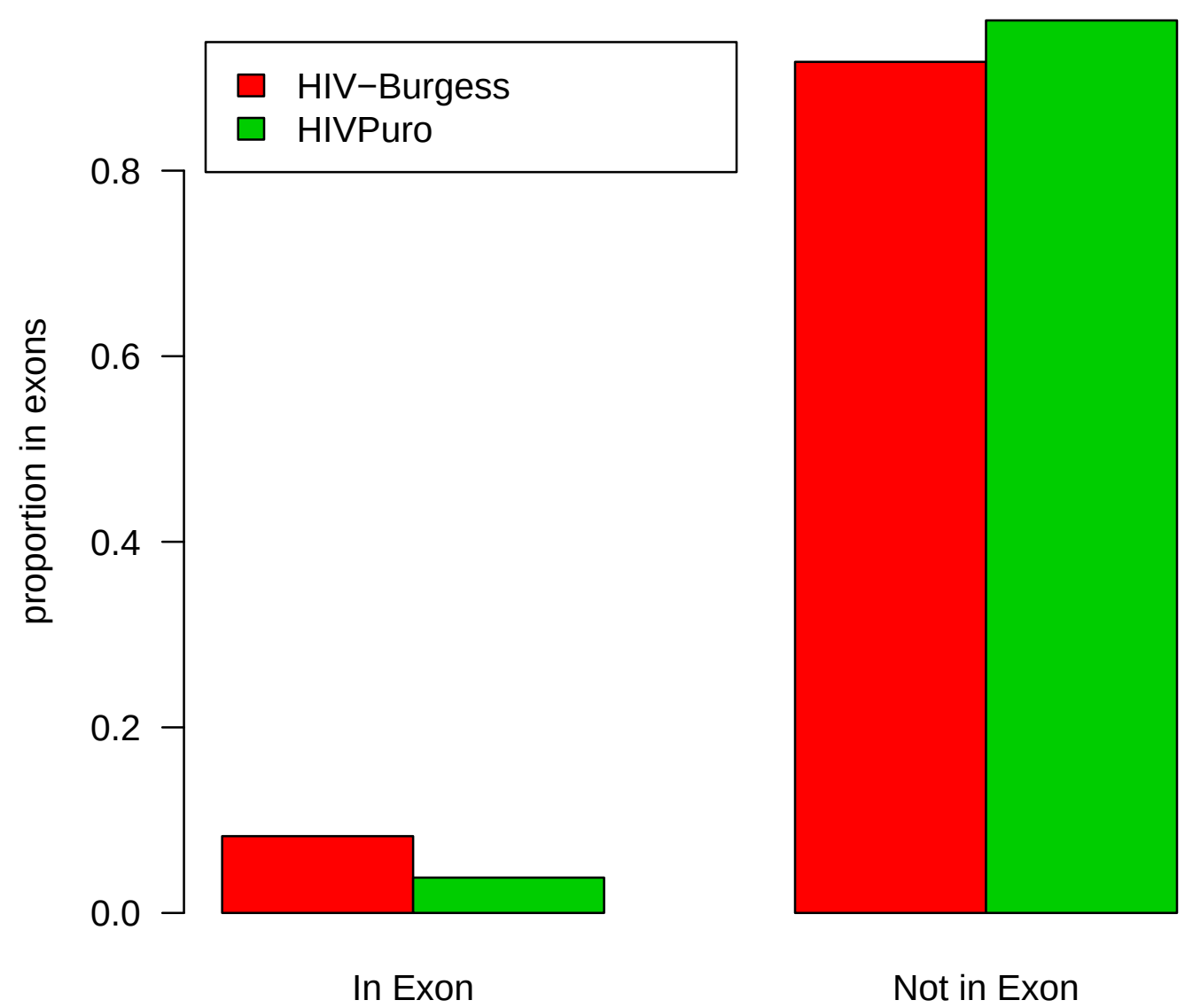

Here is the table of coefficients of the log ratio of intensities for along with their standard errors, z statistics, and p-values:

	coef	se	z	p
(Intercept)	0.411	0.165	2.49	0.01280
in.gene	0.290	0.186	1.56	0.11900
in.exon	-0.883	0.315	-2.81	0.00501

The model on which these coefficients are based include terms for whether the site is in a gene or not. Thus, the effect shown as 'in.exon' is net of that due to being in a gene. Note that in the barplot above the 'Not in Exon' bars include the both introns and intergenic regions, so the impression given by the table may differ from that for the barplot.

2.4 genScan Genes

Here we examine the p erence that insertions have for genes. In the following plot we show the relative frequency of insertions in genes according to the 'genScan' annotation.

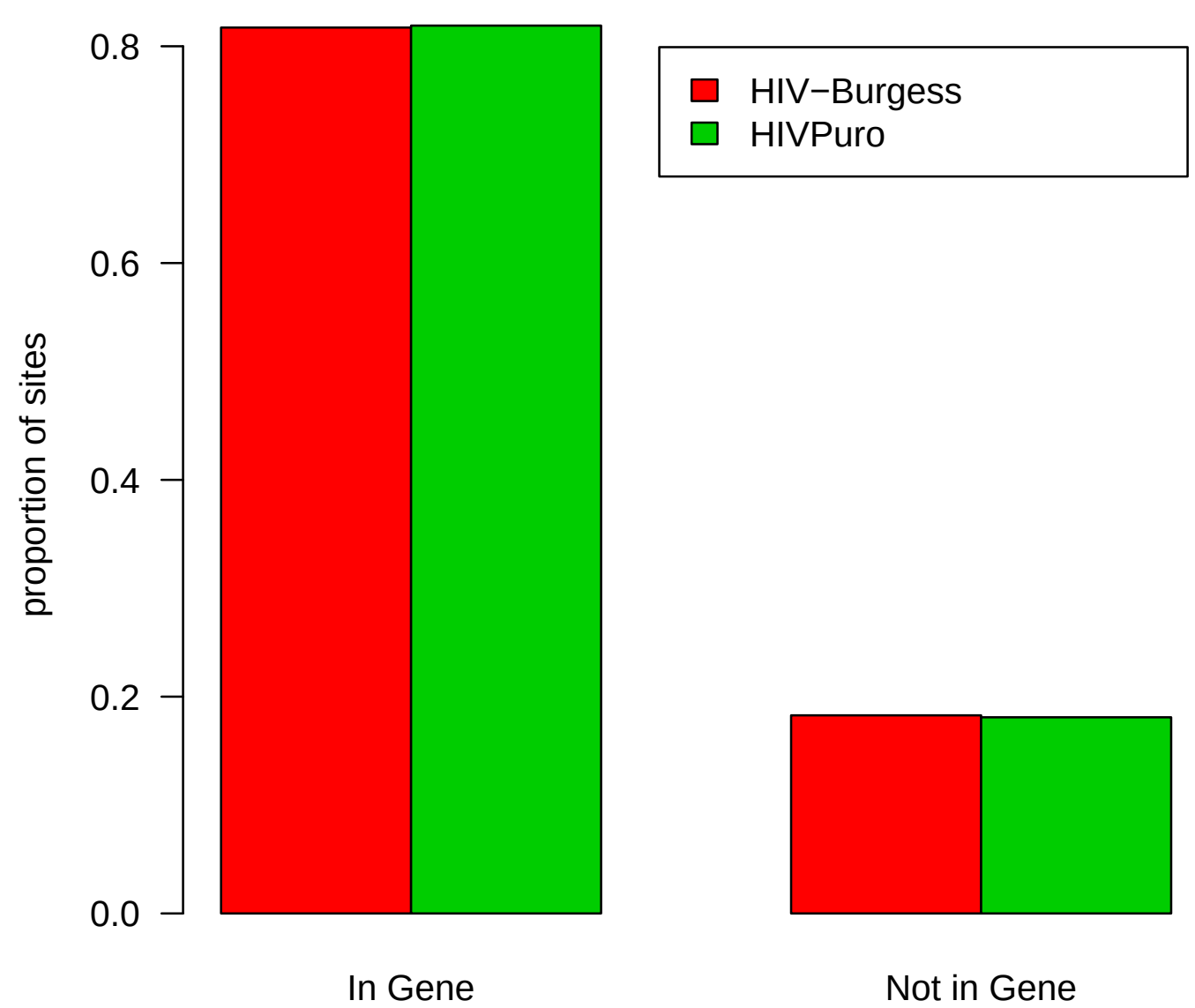

Is there is a tendency for insertions to occur in genes? A formal test of significance yields a p-value of 0.94896.

In the following plot we show the relative frequency of insertions in exons according to the 'genScan' annotation.

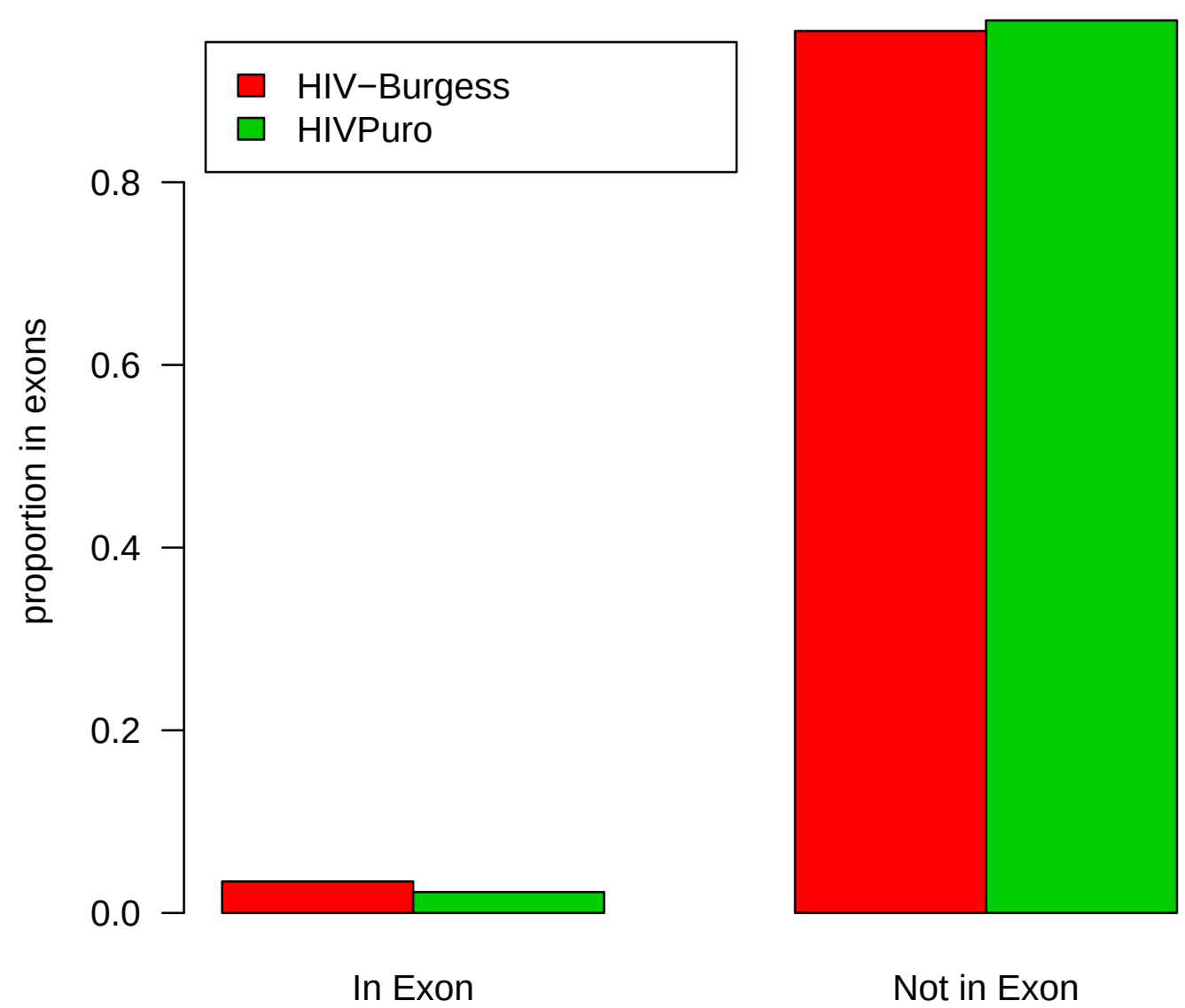

Here is the table of coefficients of the log ratio of intensities along with their standard errors, z statistics, and p-values:

	coef	se	z	p
(Intercept)	0.5840	0.171	3.400	0.000664
in.gene	0.0269	0.190	0.142	0.887000
in.exon	-0.4280	0.436	-0.982	0.326000

The model on which these coefficients are based include terms for whether the site is in a gene or not. Thus, the effect shown as 'in.exon' is net of that due to being in a gene. Note that in the barplot above the 'Not in Exon' bars include the both introns and intergenic regions, so the impression given by the table may differ from that for the barplot.

2.5 uniGenes

Here we examine the preference that insertions have for genes. In the following plot we show the relative frequency of insertions in genes according to the 'uniGene' annotation.

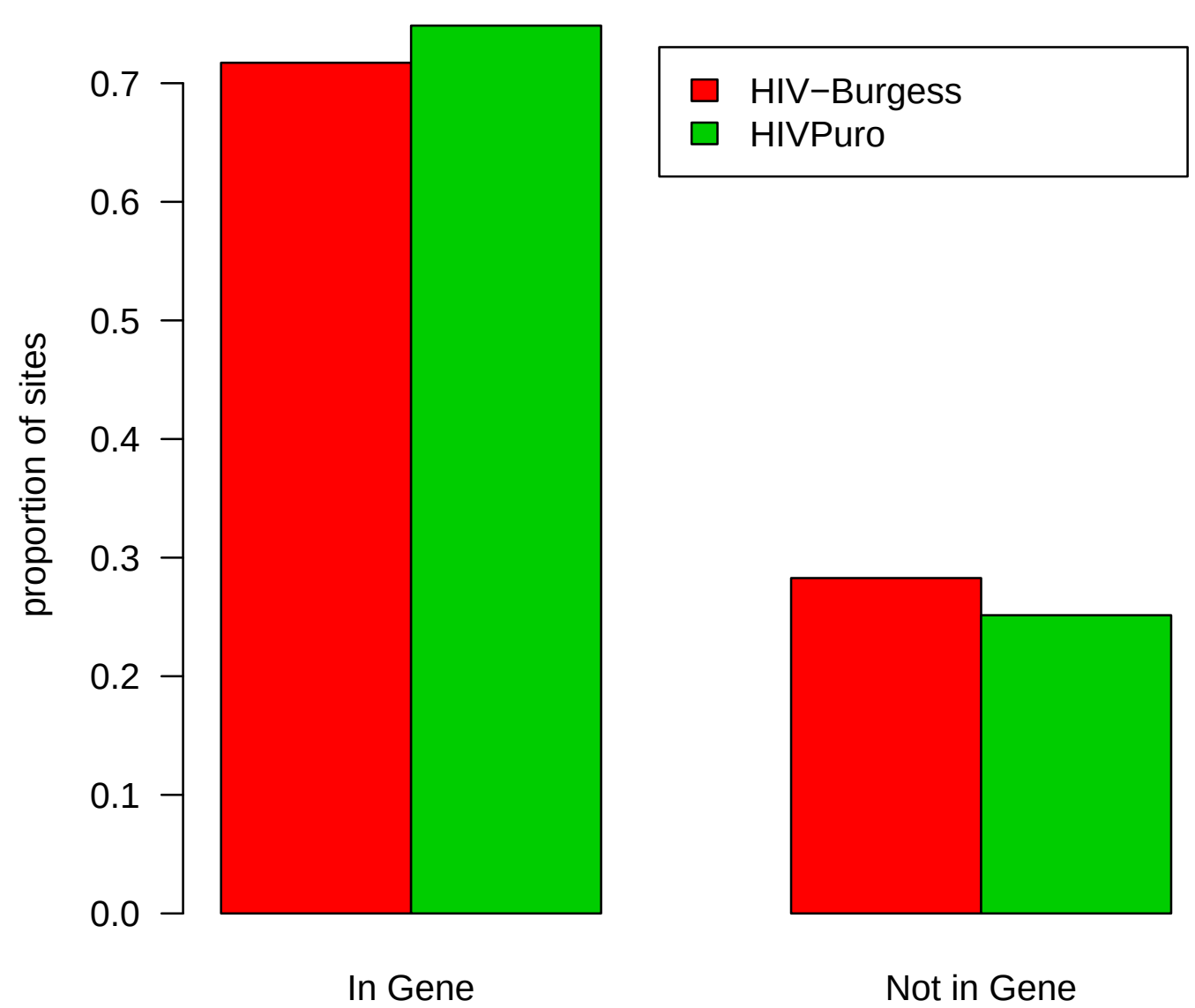

Is there is a tendency for insertions to occur in genes? A formal test of significance yields a p-value of 0.33214.

In the following plot we show the relative frequency of insertions in exons according to the 'uniGene' annotation.

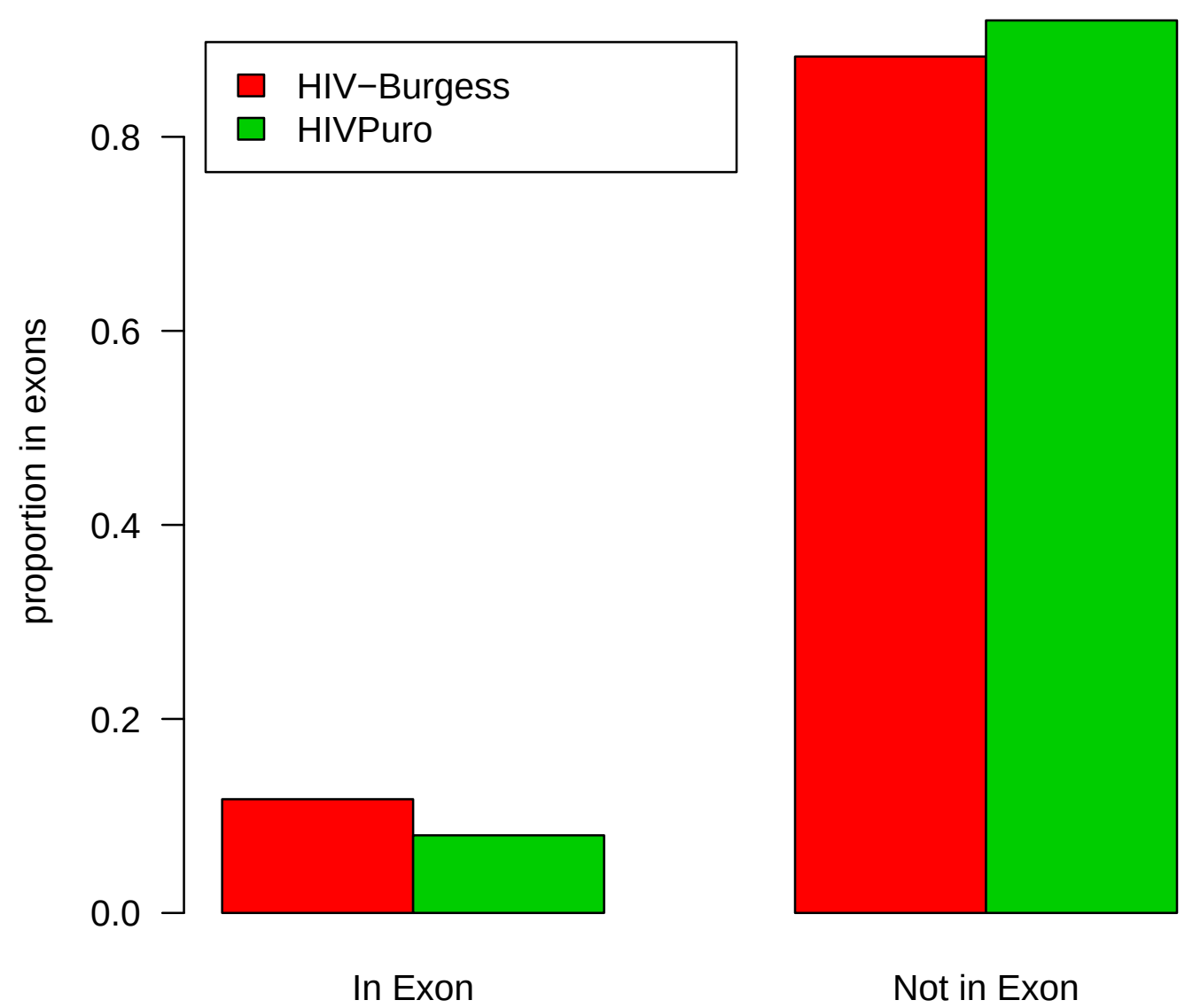

Here is the table of coefficients of the log ratio of intensities along with their standard errors, z statistics, and p-values:

	coef	se	z	p
(Intercept)	0.476	0.141	3.39	0.00071
in.gene	0.226	0.168	1.34	0.18000
in.exon	-0.490	0.249	-1.97	0.04860

The model on which these coefficients are based include terms for whether the site is in a gene or not. Thus, the effect shown as 'in.exon' is net of that due to being in a gene. Note that in the barplot above the 'Not in Exon' bars include the both introns and intergenic regions, so the impression given by the table may differ from that for the barplot.

3 CpG Island Neighborhoods

Here we study the effect of being in the neighborhood of CpG Islands. Following Wu et al [2], who found that the neighborhoods within ±1kb of CpG islands are enriched for MLV insertions, we study such neighborhoods.

3.1 1 kilobase neighborhoods

The following plot shows the effect of being in or within ±1kb of a CpG island:

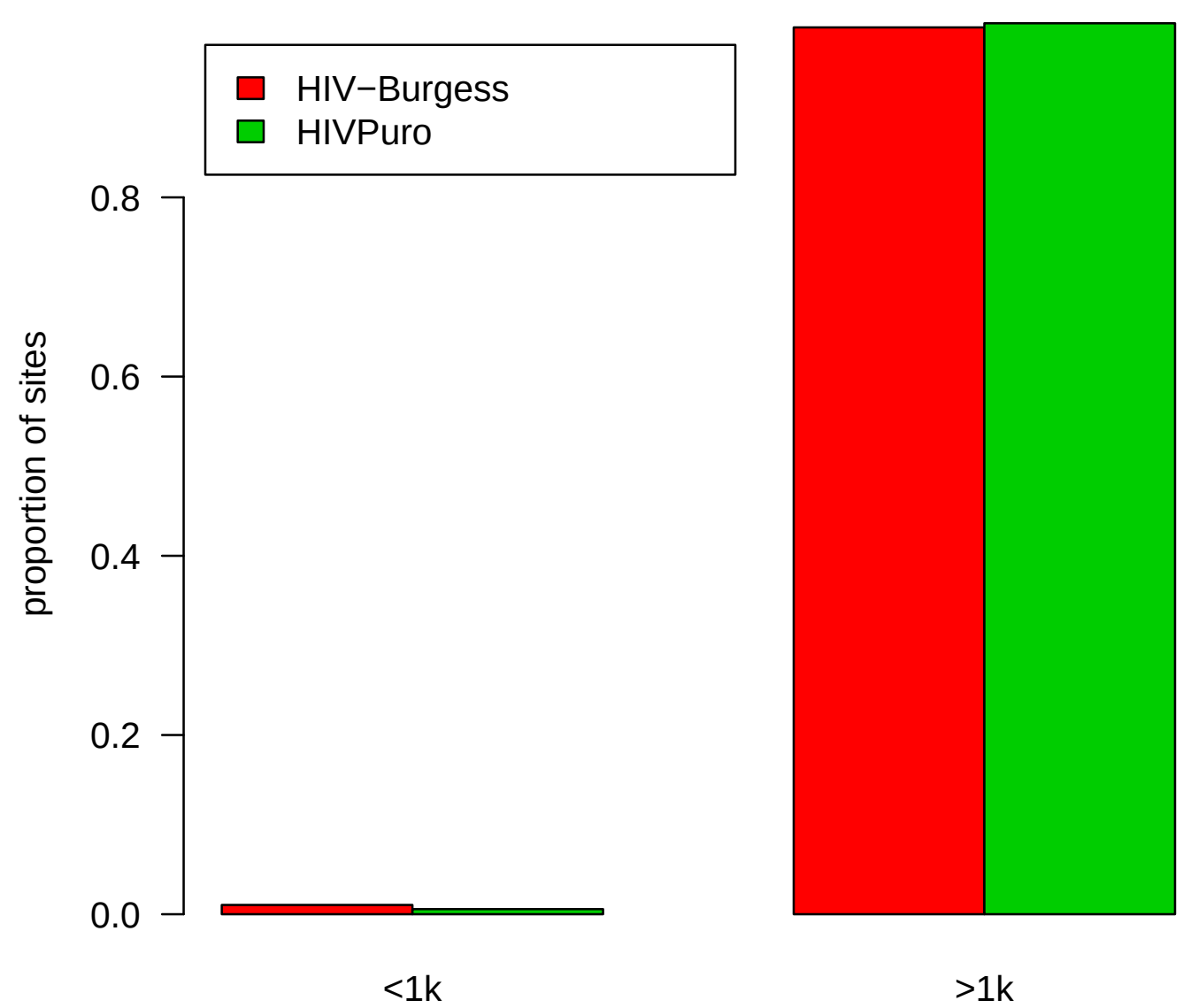

A formal test of significance comparing the difference attains a p-value of 0.46878.

3.2 5 kilobase neighborhoods

The following plot shows the effect of being in or within ±5kb of a CpG island:

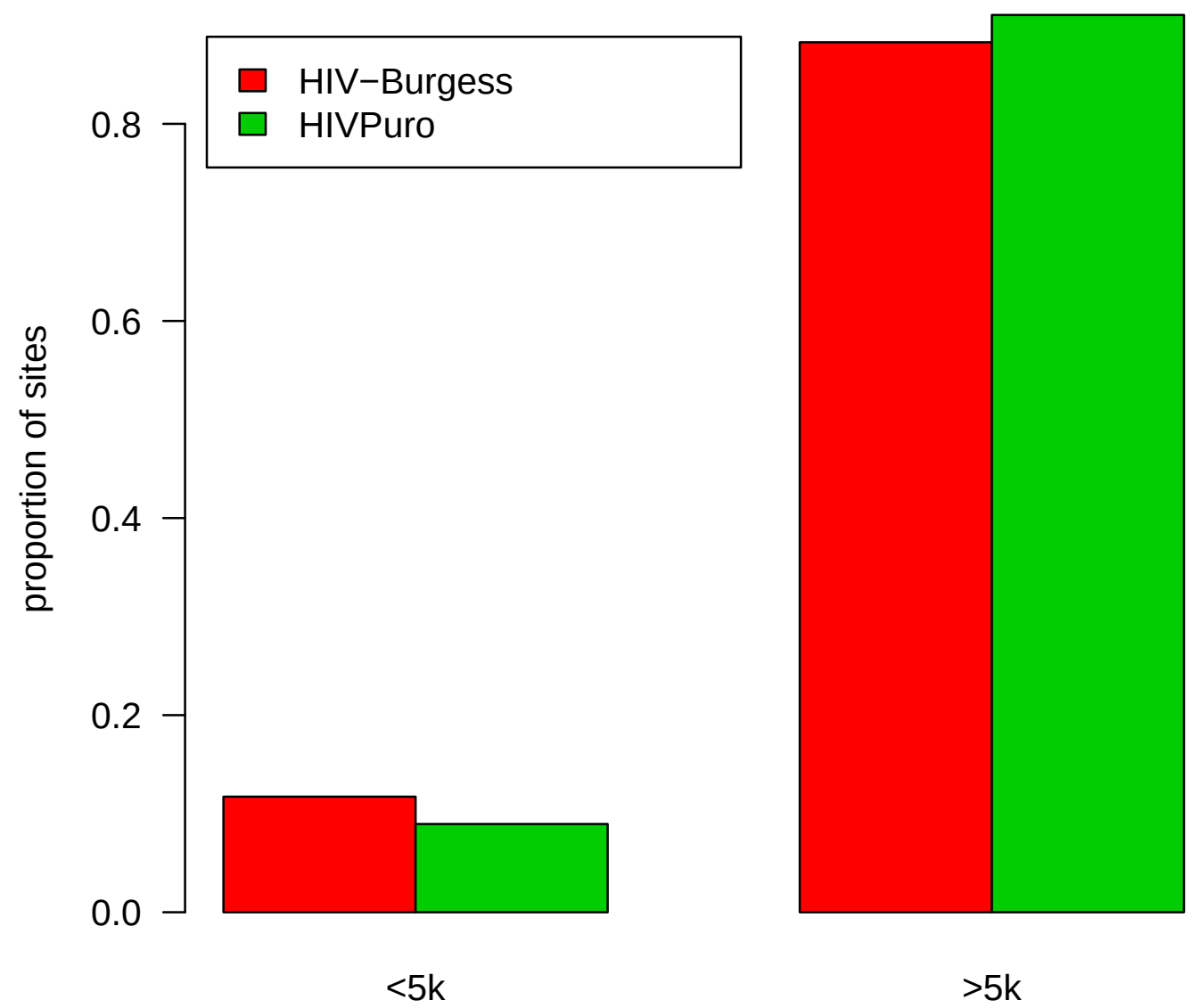

A formal test of significance comparing the difference attains a p-value of 0.21012.

3.3 10 kilobase neighborhoods

The following plot shows the effect of being in or within ±10kb of a CpG island:

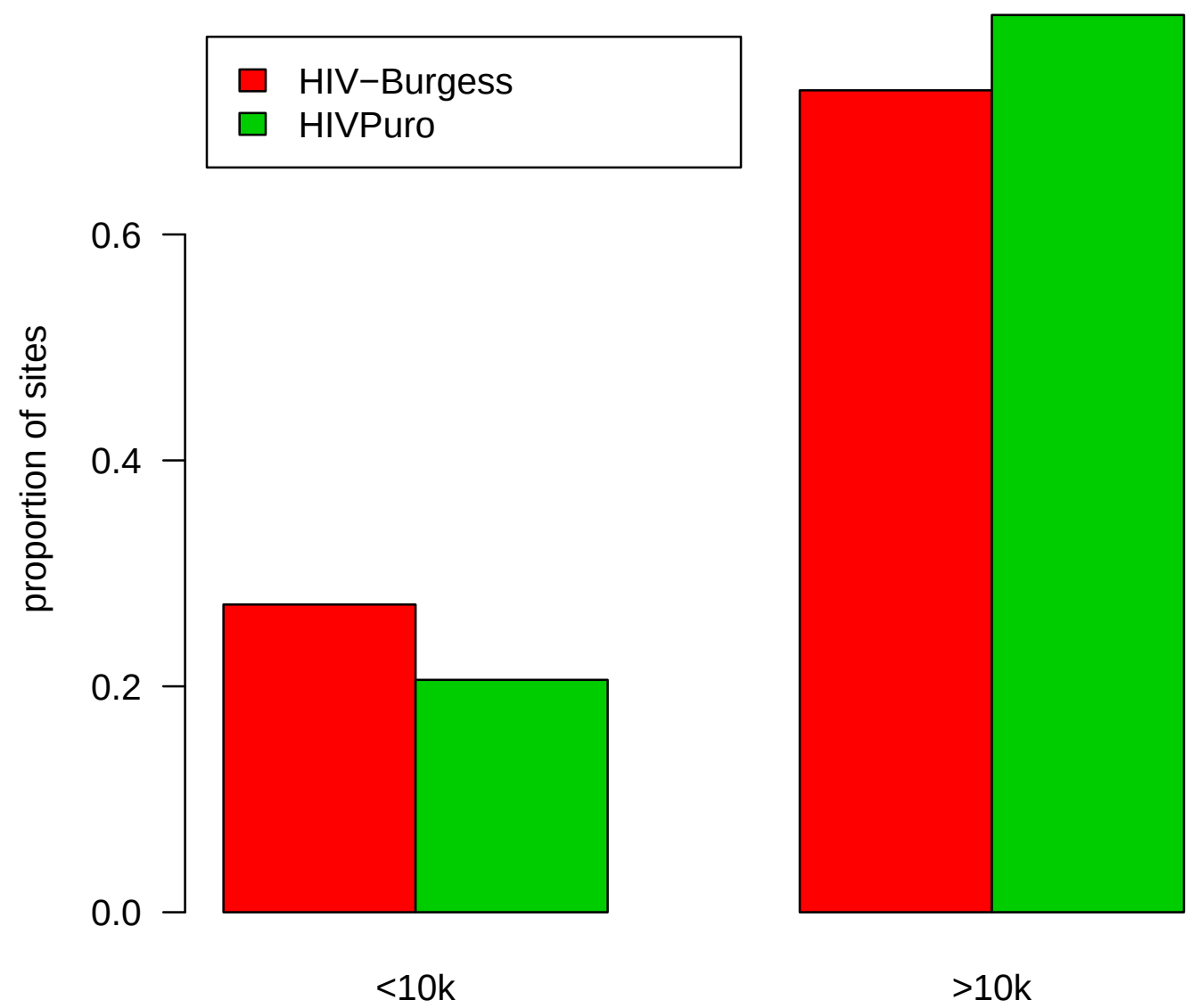

A formal test of significance comparing the difference attains a p-value of 0.031535.

3.4 25 kilobase neighborhoods

The following plot shows the effect of being in or within ±25kb of a CpG island:

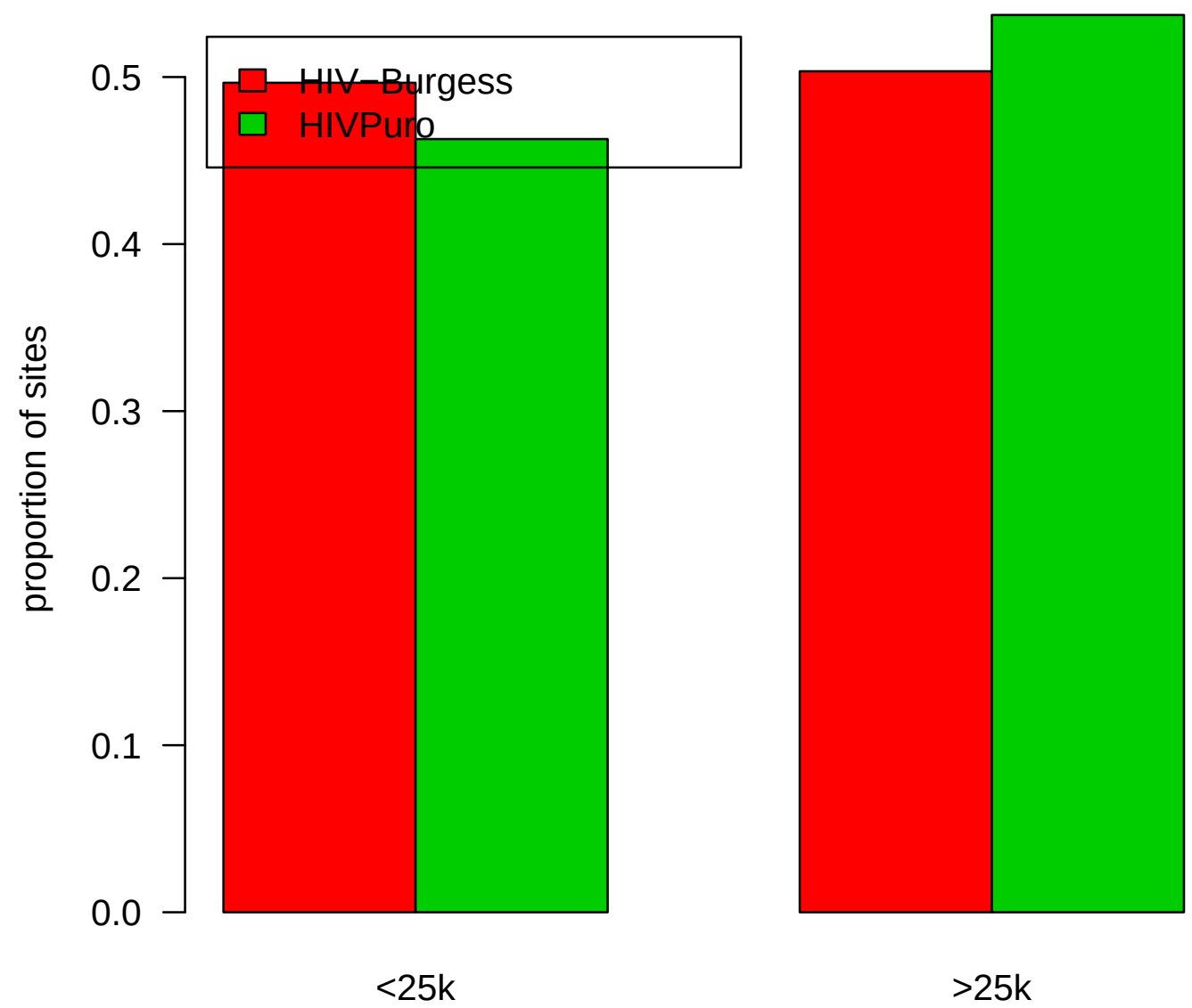

A formal test of significance comparing the difference attains a p-value of 0.35651.

3.5 50 kilobase neighborhoods

The following plot shows the effect of being in or within ±50kb of a CpG island:

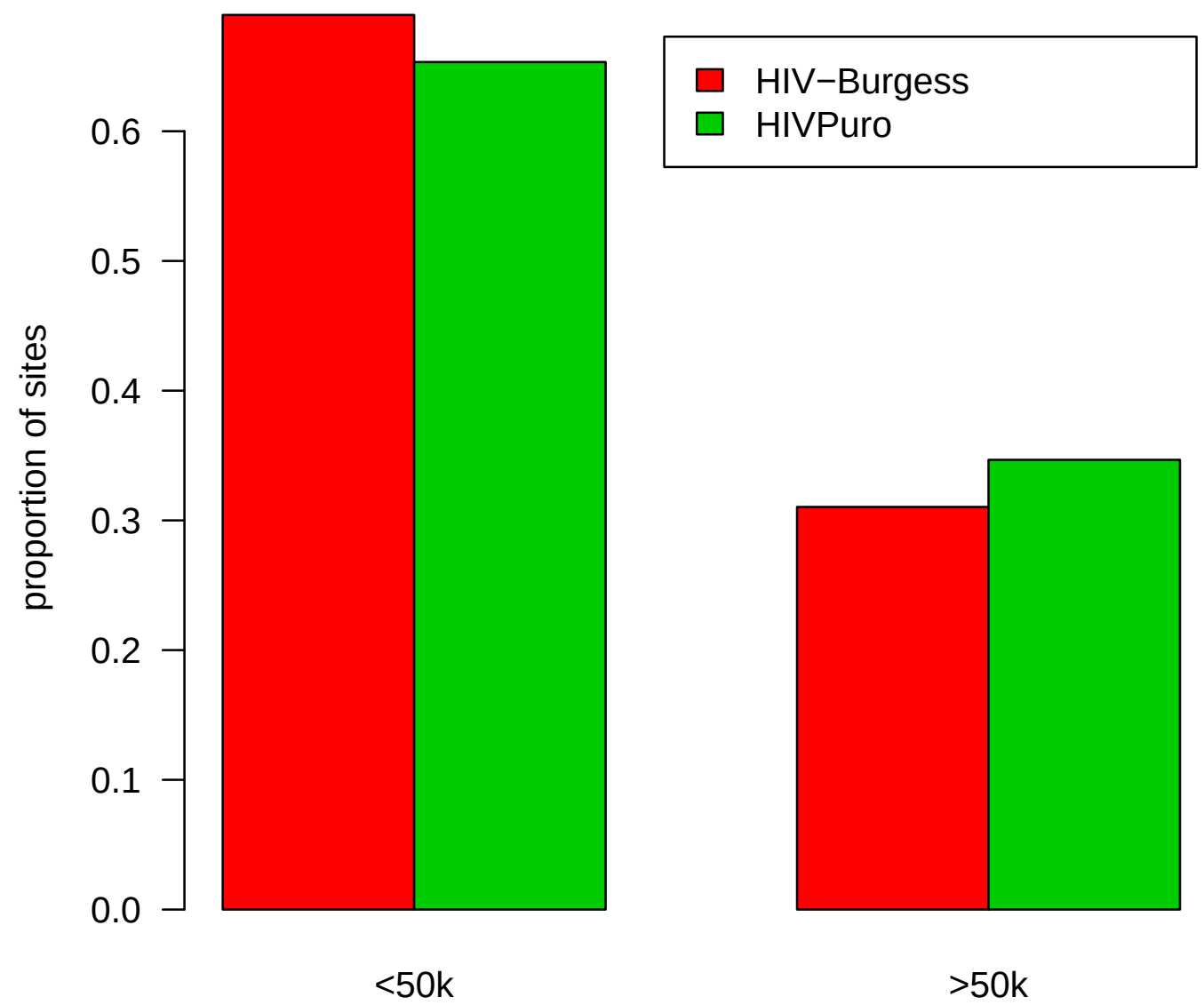

A formal test of significance comparing the difference attains a p-value of 0.29107.

4 Gene Density, Expression 'Density', and CpG Island Density

In this section the association with gene density is examined. The 'genes' that are counted are the genes represented on the microarray. In addition, we the number of such genes expressed at various levels. The levels are

low.ex Count genes whose expression is in the upper half and divide by number of bases

med.ex Count genes whose expression is in the upper $1/8^{th}$ and divide by number of bases

high.ex Count genes whose expression is in the upper $1/16^{th}$ and divide by number of bases

The bolded terms are used as abbreviations in what follows. The abbreviation **dens** is used to indicate gene density as number of genes per base.

4.1 25 kiloBase Window

In the barplot that follows we examine the association of insertion sites with gene density in a 25 kilobase window surrounding each locus. More such plots will follow and the method of their construction is always to try to divide the data according to the deciles of density. However, it often happens that there is a very skewed distribution of density and often even the 90th percentile is zero. In that case, the barplots simply show the sites for which the density is zero and those for which it is non-zero. If there are fewer than ten groups of bars, then the groupings contain ten percent of the sites each except for the leftmost grouping which will contain all of the remaining sites.

Also note that the title of the plot contains clues as to its content; the prefix indicates the type of variable studied while the suffix indicates the window width in the number of bases. The p-value given is the result of fitting a cubic polynomial to the gene density values.

The following expression data and probe set were used for this report:

```
[1] "HeLa_exp_data-HU133a"

[1] "HG-U133"

 Category limits

  lower category   upper
1 0e+00  group.1 0.00001
2 1e-05  group.2 0.00002
3 2e-05  group.3 0.00004
4 4e-05  group.4 0.00012
```

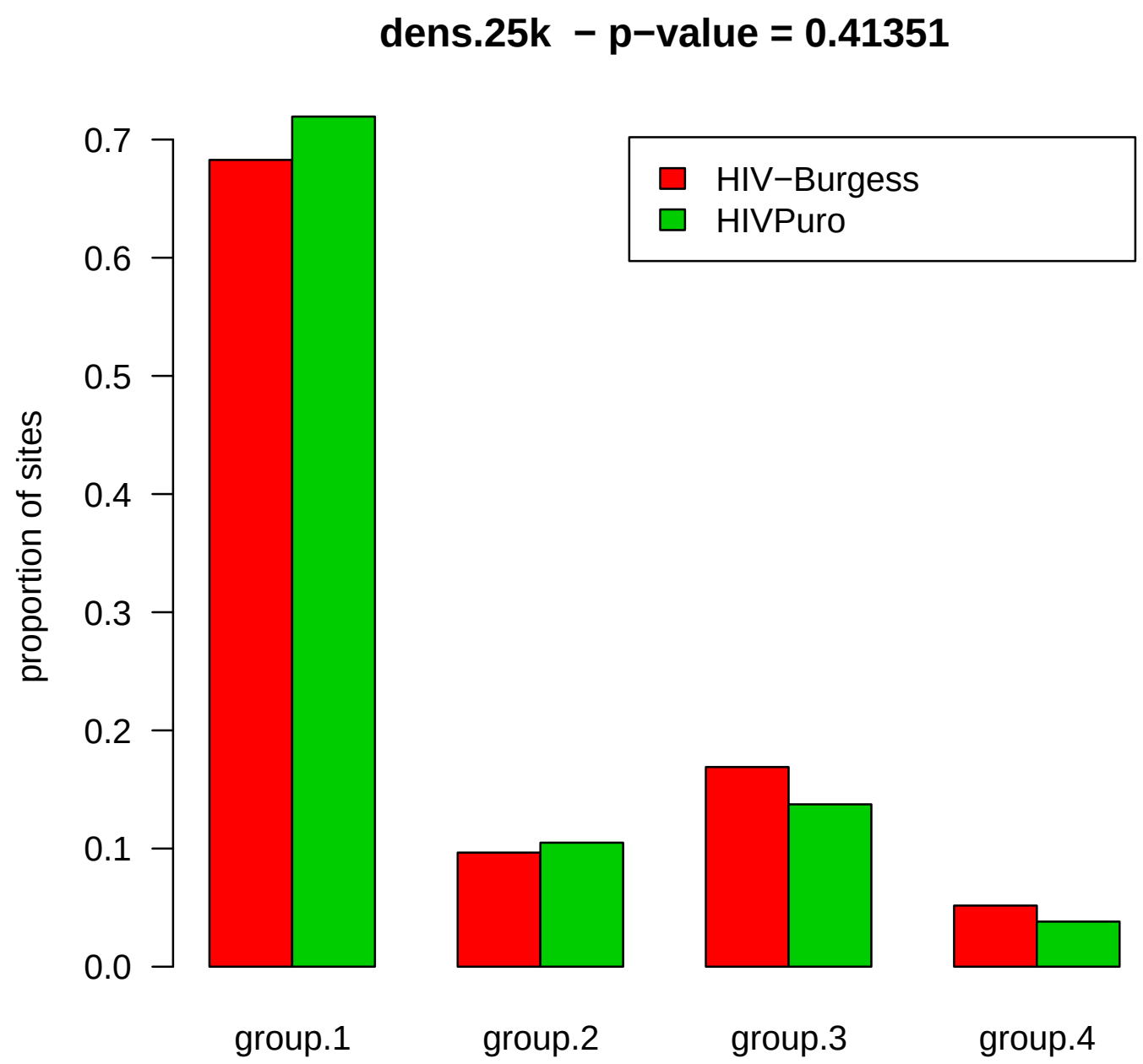
dens.25k − p−value = 0.41351
HIV−Burgess
HIVPuro
proportion of sites
0.0
0.1
0.2
0.3
0.4
0.5
0.6
0.7
group.1
group.2
group.3
group.4

Here are the results for expression density. First, we count just genes that are in the upper half.

```
 Category limits

          lower  category        upper
1 0.000000e+00  group.1 1.333333e-05
2 1.333333e-05  group.2 3.880000e-05
3 3.880000e-05  group.3 8.000000e-05
```

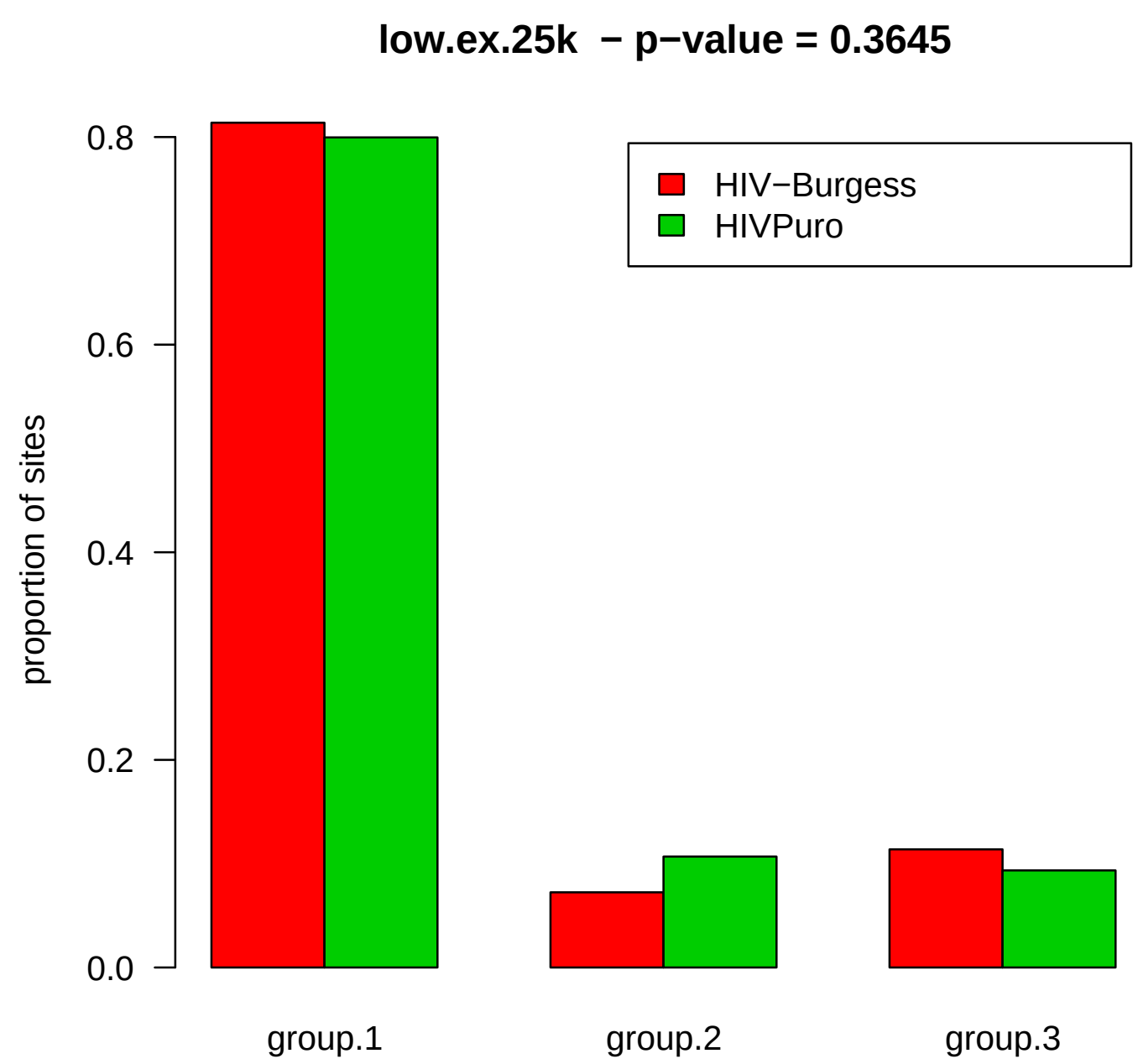

Now we count genes in the upper $1/8^{th}$:

```
Category limits

  lower category        upper
1 0e+00  group.1 2.000000e-05
2 2e-05  group.2 6.666667e-05
```

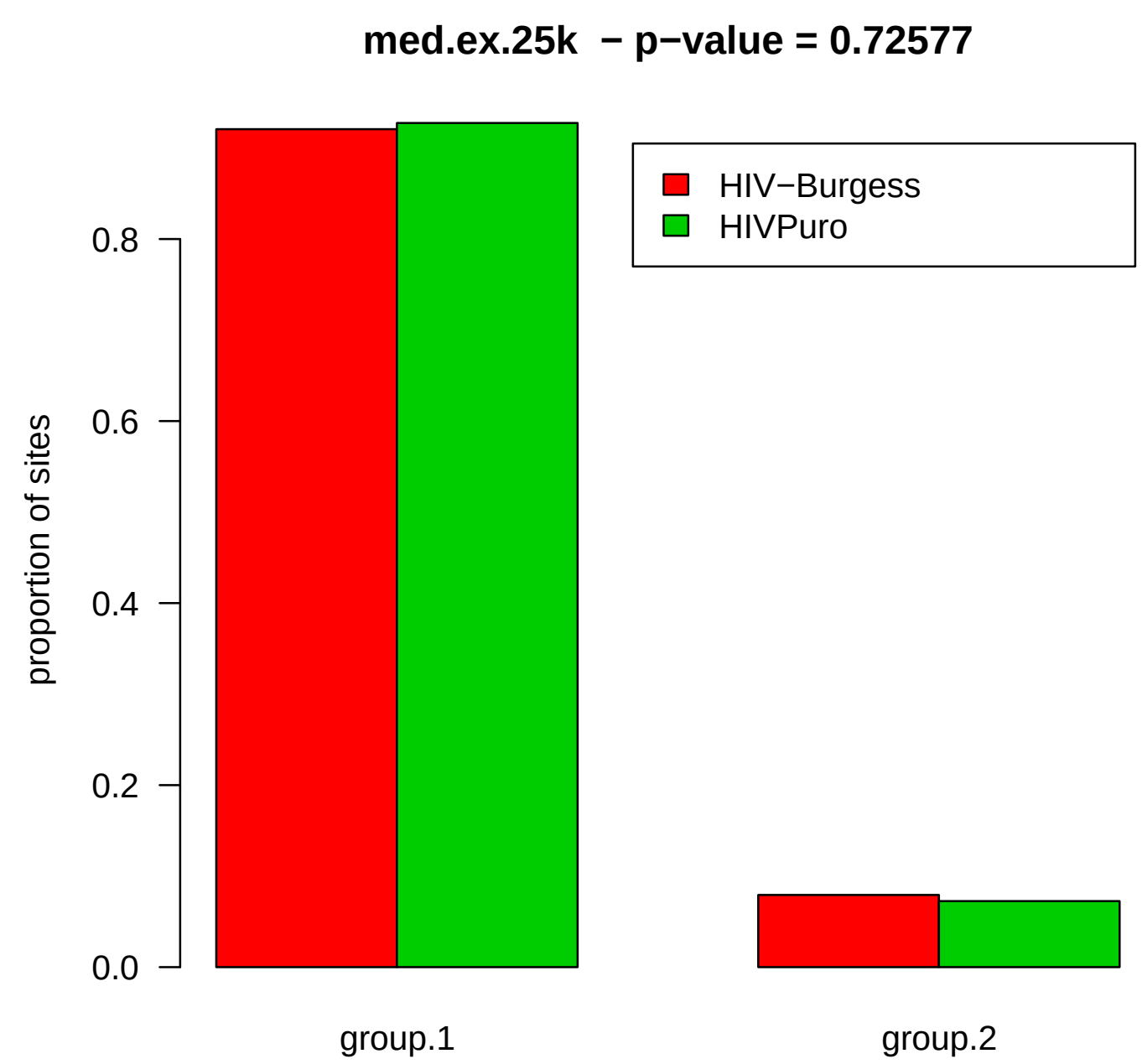

And here we count genes in the upper $1/16^{th}$:

```
Category limits

            lower category        upper
0%   0.000000e+00  group.1 3.333333e-06
100% 3.333333e-06  group.2 6.000000e-05
```

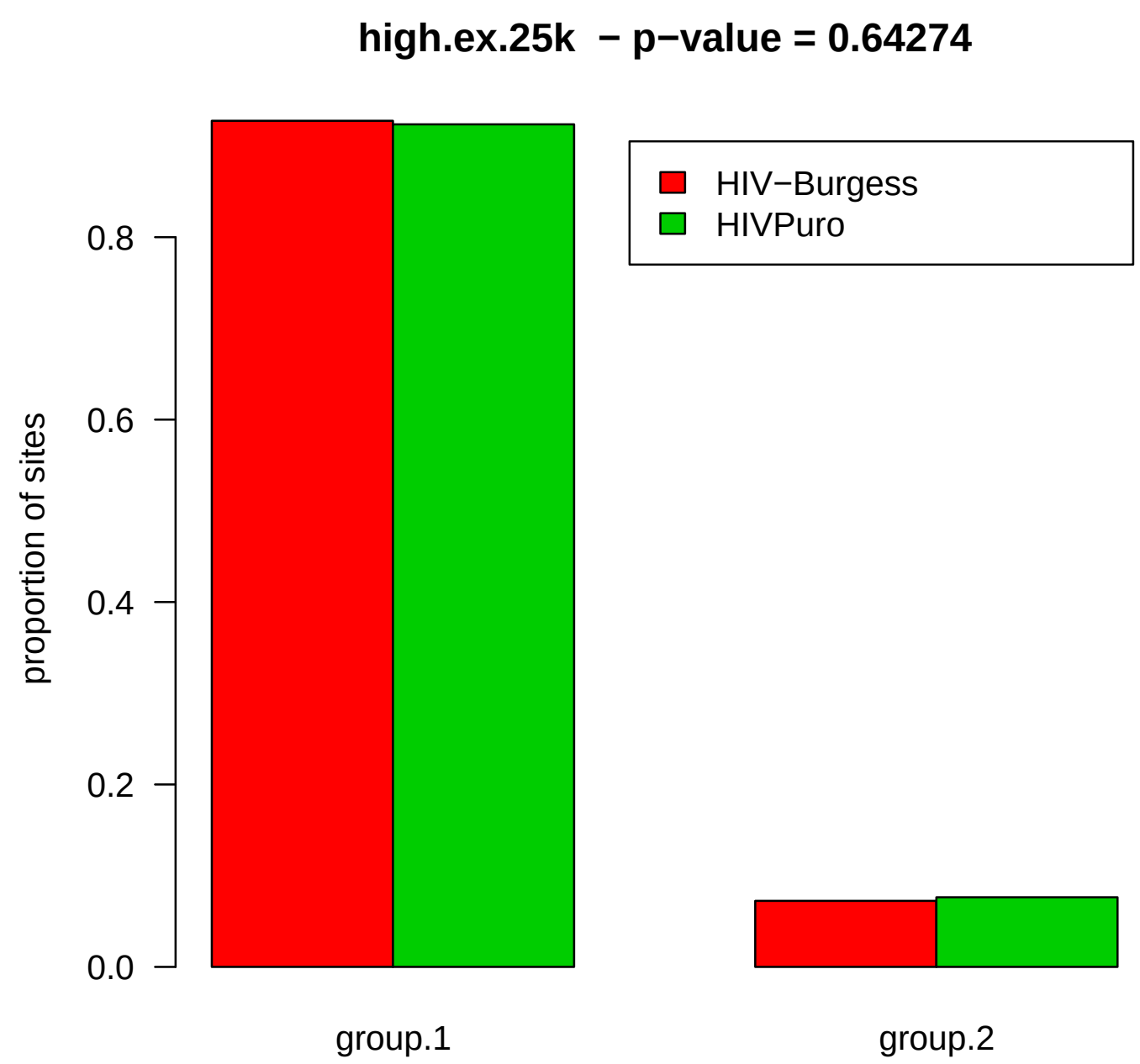

Here the effect of density of CpG islands is studied:

```
Category limits

  lower category   upper
1 0e+00  group.1 0.00002
2 2e-05  group.2 0.00004
3 4e-05  group.3 0.00024
```

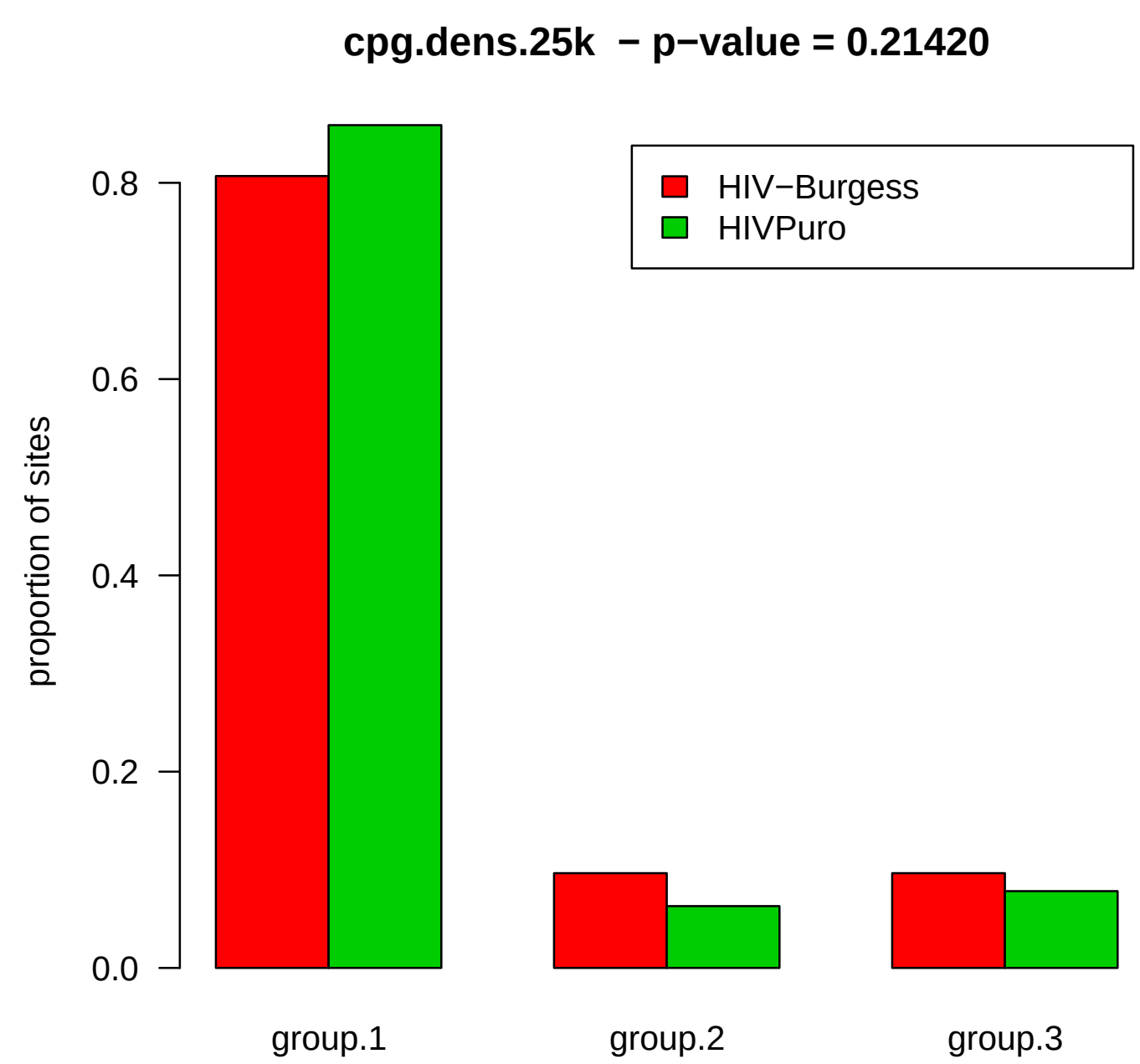

4.2 50 kiloBase Window

In the barplot that follows we examine the association of insertion sites with expression density in a 50 kilobase window surrounding each locus. First, we count just the number of genes on the represented on the chip.

```
 Category limits

         lower category        upper
1 0.000000e+00  group.1 4.000000e-06
2 4.000000e-06  group.2 1.000000e-05
3 1.000000e-05  group.3 1.333333e-05
4 1.333333e-05  group.4 2.000000e-05
5 2.000000e-05  group.5 3.000000e-05
6 3.000000e-05  group.6 9.666667e-05
```

dens.50k − p−value = 0.12079

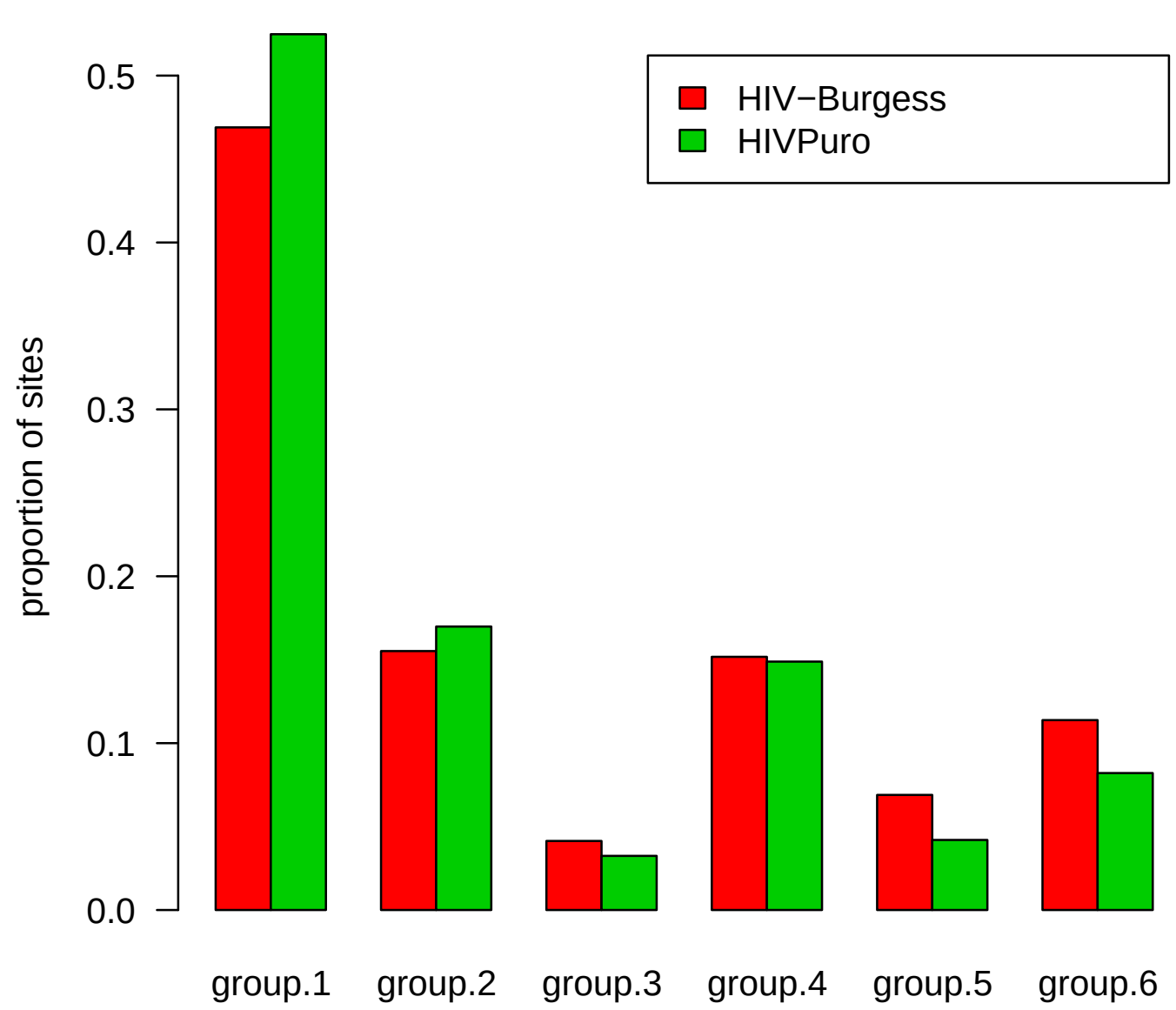

Here are the results for expression density. First, we count just genes that are in the upper half.

```
Category limits

         lower category        upper
1 0.000000e+00  group.1 3.575758e-06
2 3.575758e-06  group.2 8.000000e-06
3 8.000000e-06  group.3 1.333333e-05
4 1.333333e-05  group.4 2.000000e-05
5 2.000000e-05  group.5 7.000000e-05
```

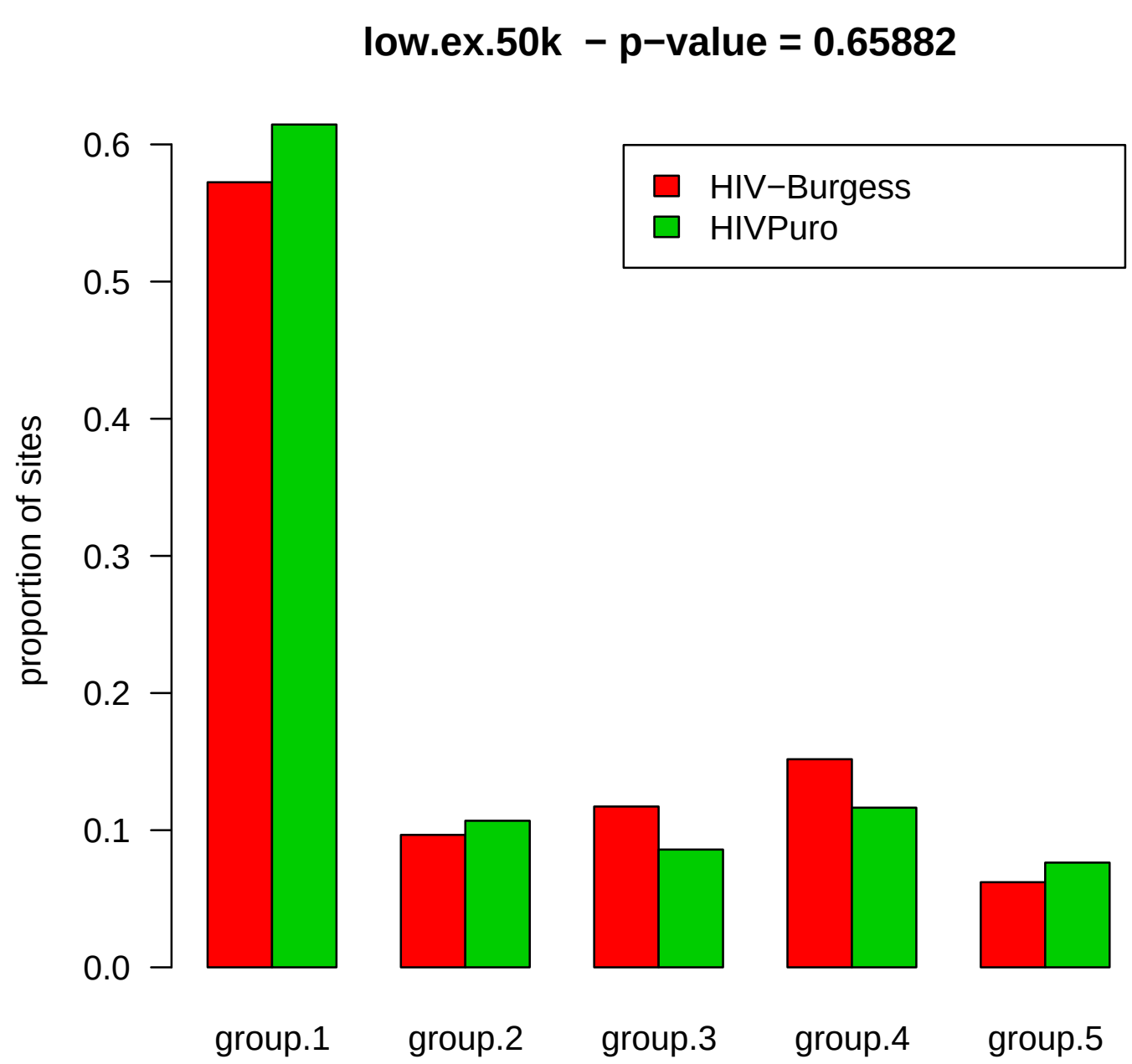

Now we count genes in the upper $1/8^{th}$:

```
Category limits

         lower category        upper
1 0.000000e+00  group.1 6.666667e-06
2 6.666667e-06  group.2 1.666667e-05
3 1.666667e-05  group.3 7.000000e-05
```

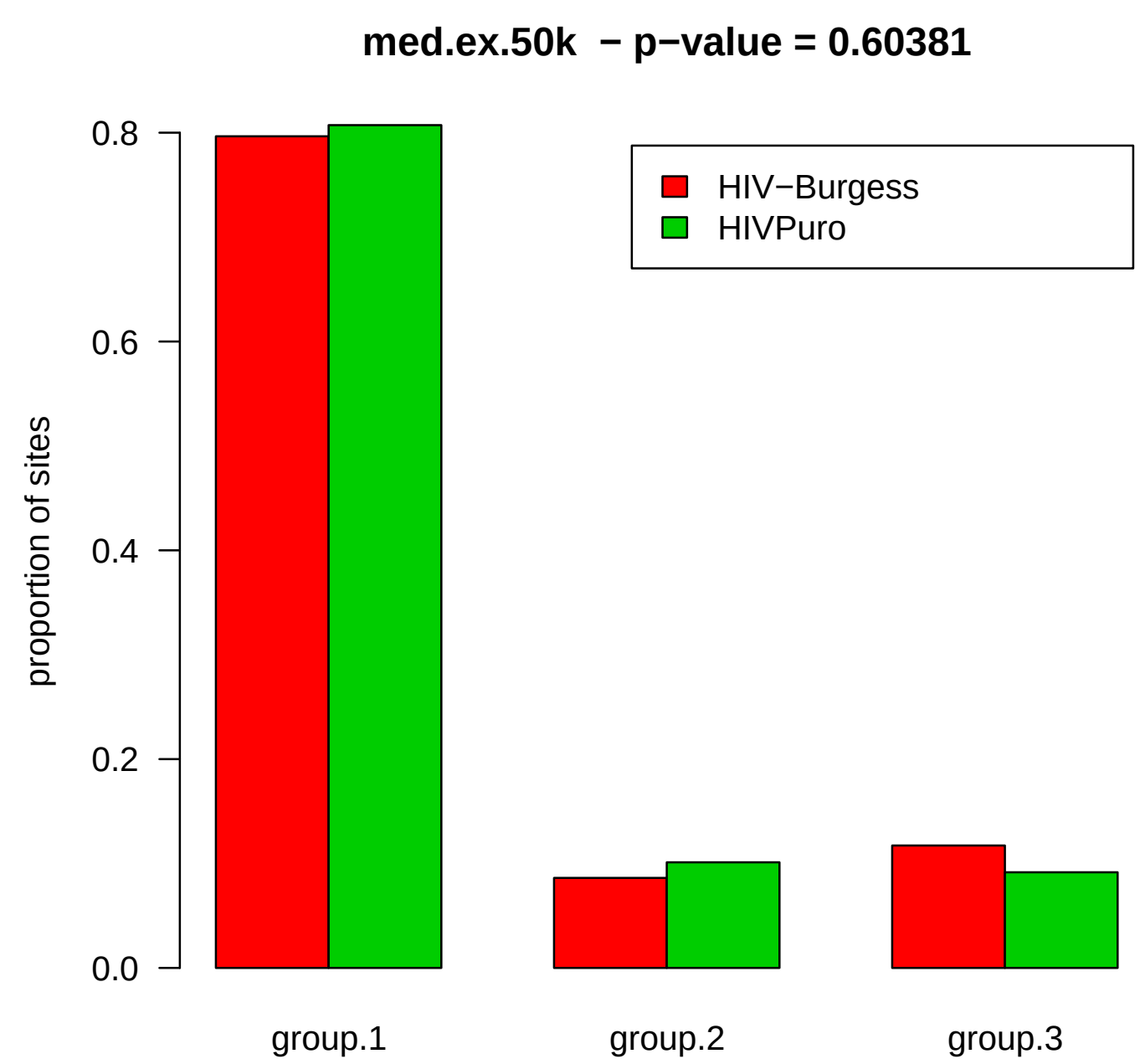

And here we count genes in the upper $1/16^{th}$:

```
Category limits

         lower category        upper
1 0.000000e+00  group.1 6.666667e-06
2 6.666667e-06  group.2 7.000000e-05
```

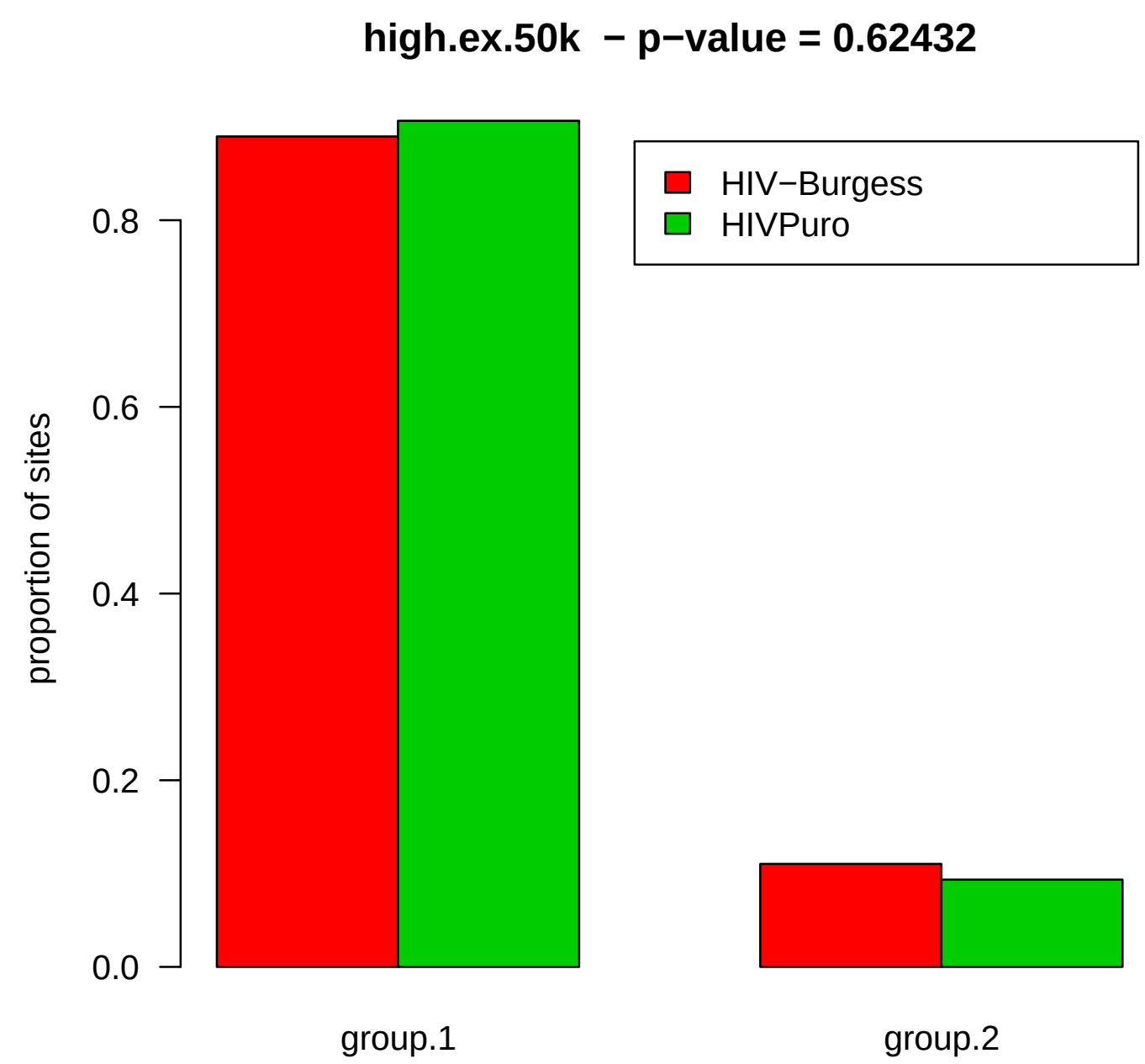

Here the effect of density of CpG islands is studied:

```
Category limits

  lower category   upper
1 0e+00  group.1 0.00001
2 1e-05  group.2 0.00002
3 2e-05  group.3 0.00003
4 3e-05  group.4 0.00005
5 5e-05  group.5 0.00026
```

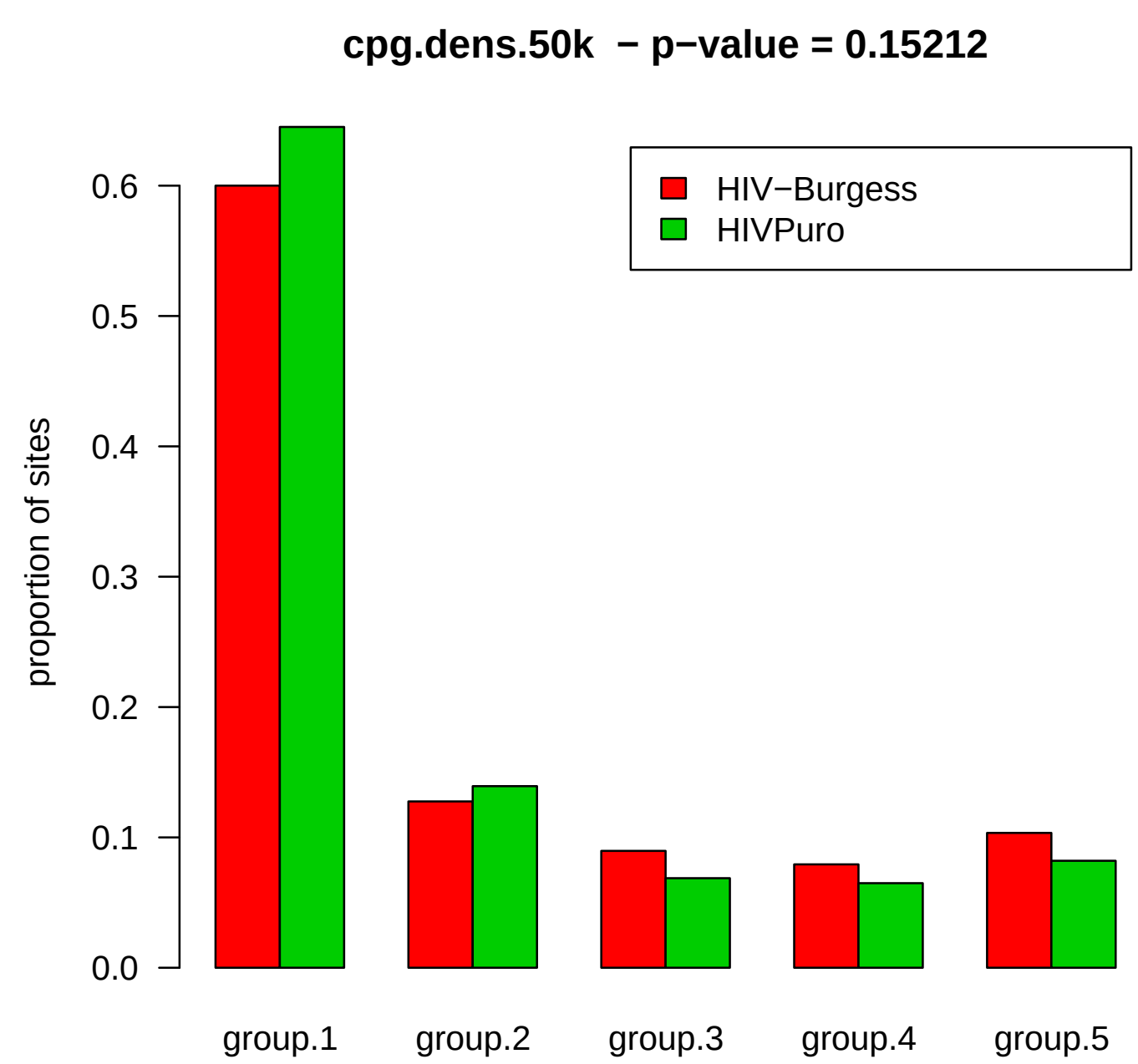

4.3 100 kiloBase Window

In the barplot that follows we examine the association of insertion sites with expression density in a 100 kilobase window surrounding each locus. First, we count just the number of genes on the represented on the chip.

```
 Category limits

         lower category        upper
1 0.000000e+00  group.1 4.000000e-06
2 4.000000e-06  group.2 6.666667e-06
3 6.666667e-06  group.3 1.000000e-05
4 1.000000e-05  group.4 1.100000e-05
5 1.100000e-05  group.5 1.666667e-05
6 1.666667e-05  group.6 2.500000e-05
7 2.500000e-05  group.7 8.833333e-05
```

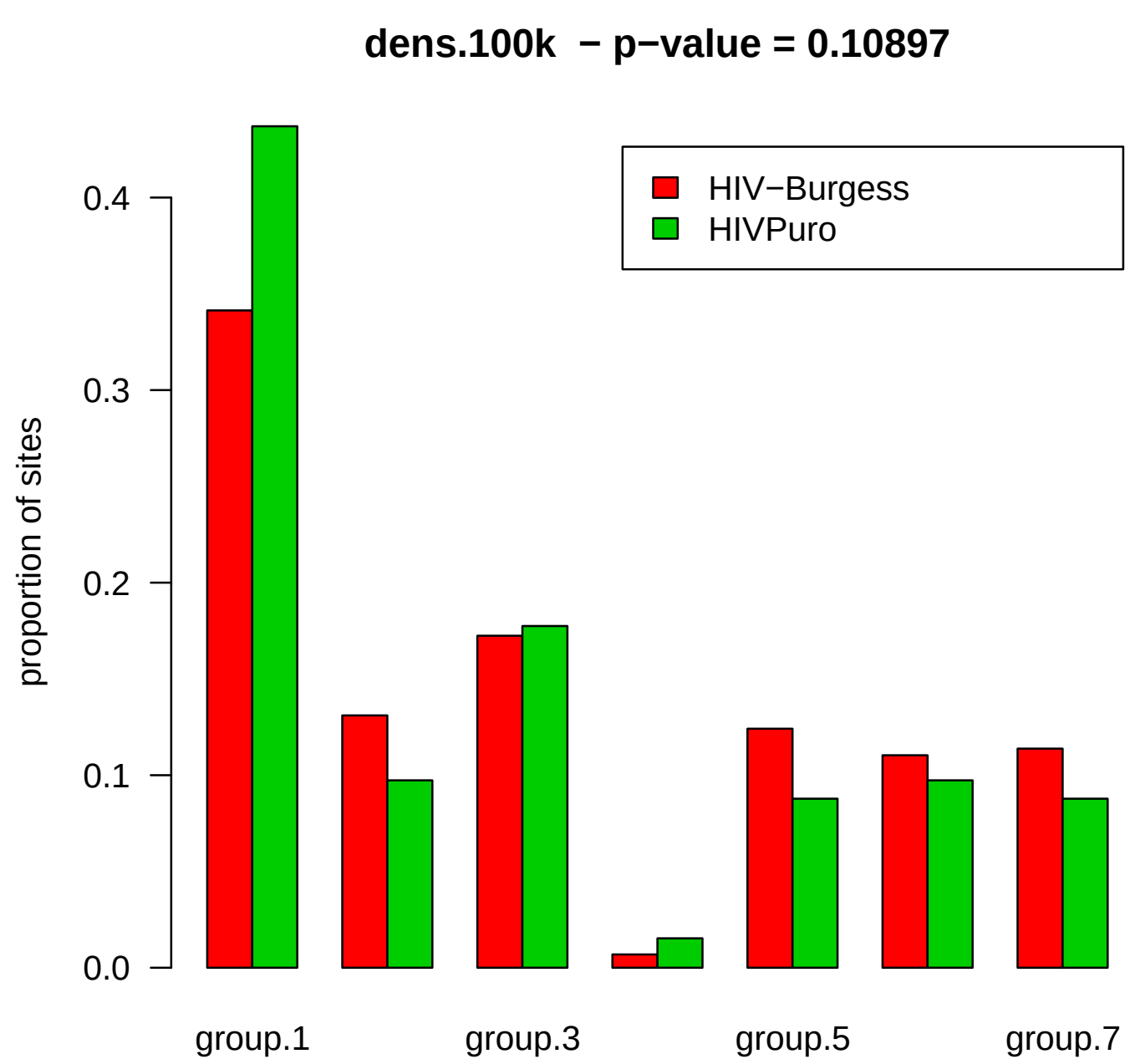

Here are the results for expression density. First, we count just genes that are in the upper half.

```
 Category limits

         lower category        upper
1 0.000000e+00  group.1 2.678571e-06
2 2.678571e-06  group.2 5.000000e-06
3 5.000000e-06  group.3 7.850000e-06
4 7.850000e-06  group.4 1.000000e-05
5 1.000000e-05  group.5 1.666667e-05
6 1.666667e-05  group.6 6.000000e-05
```

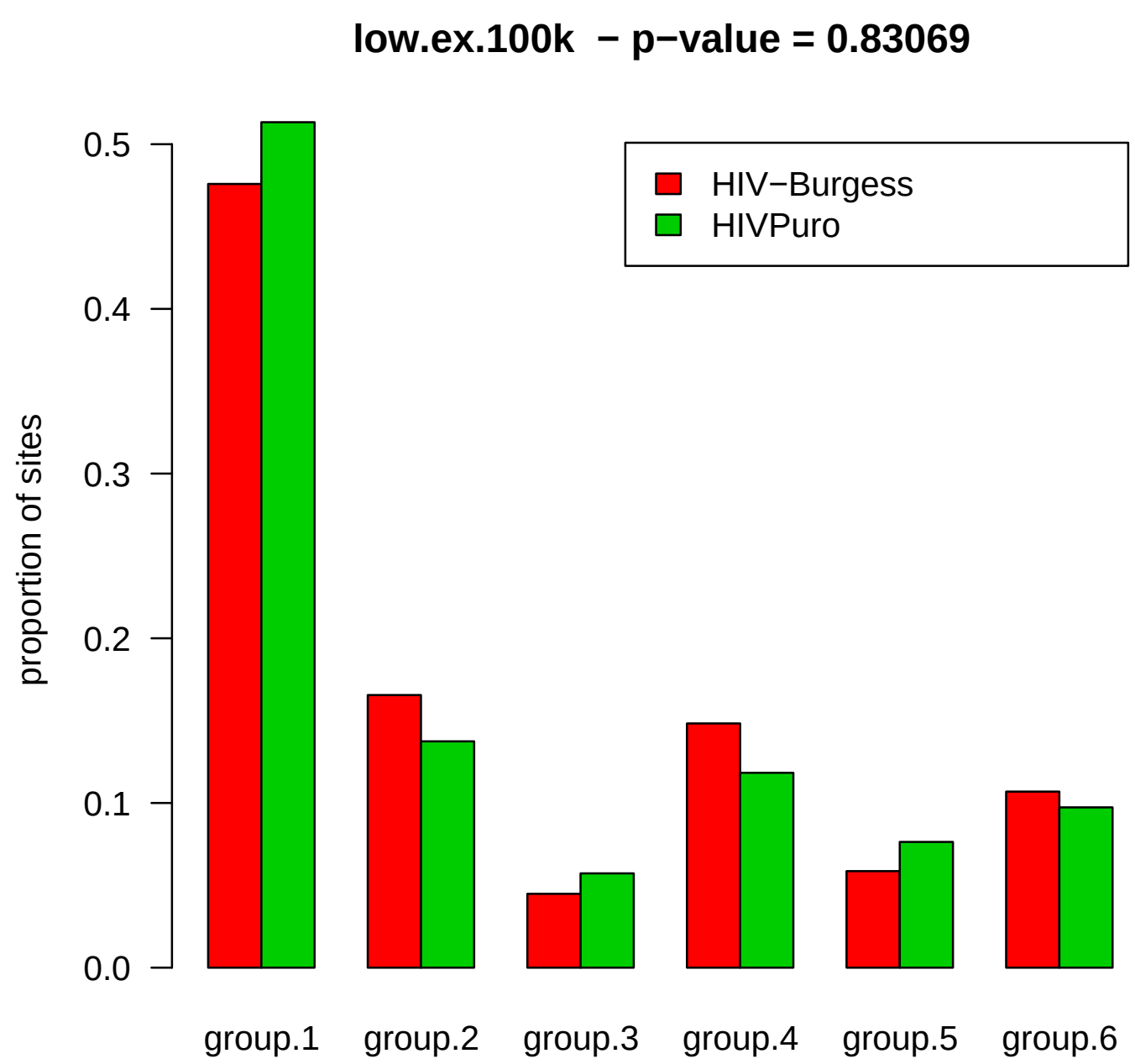

Now we count genes in the upper $1/8^{th}$:

```
Category limits

         lower category        upper
1 0.000000e+00  group.1 3.333333e-06
2 3.333333e-06  group.2 6.666667e-06
3 6.666667e-06  group.3 1.000000e-05
4 1.000000e-05  group.4 5.000000e-05
```

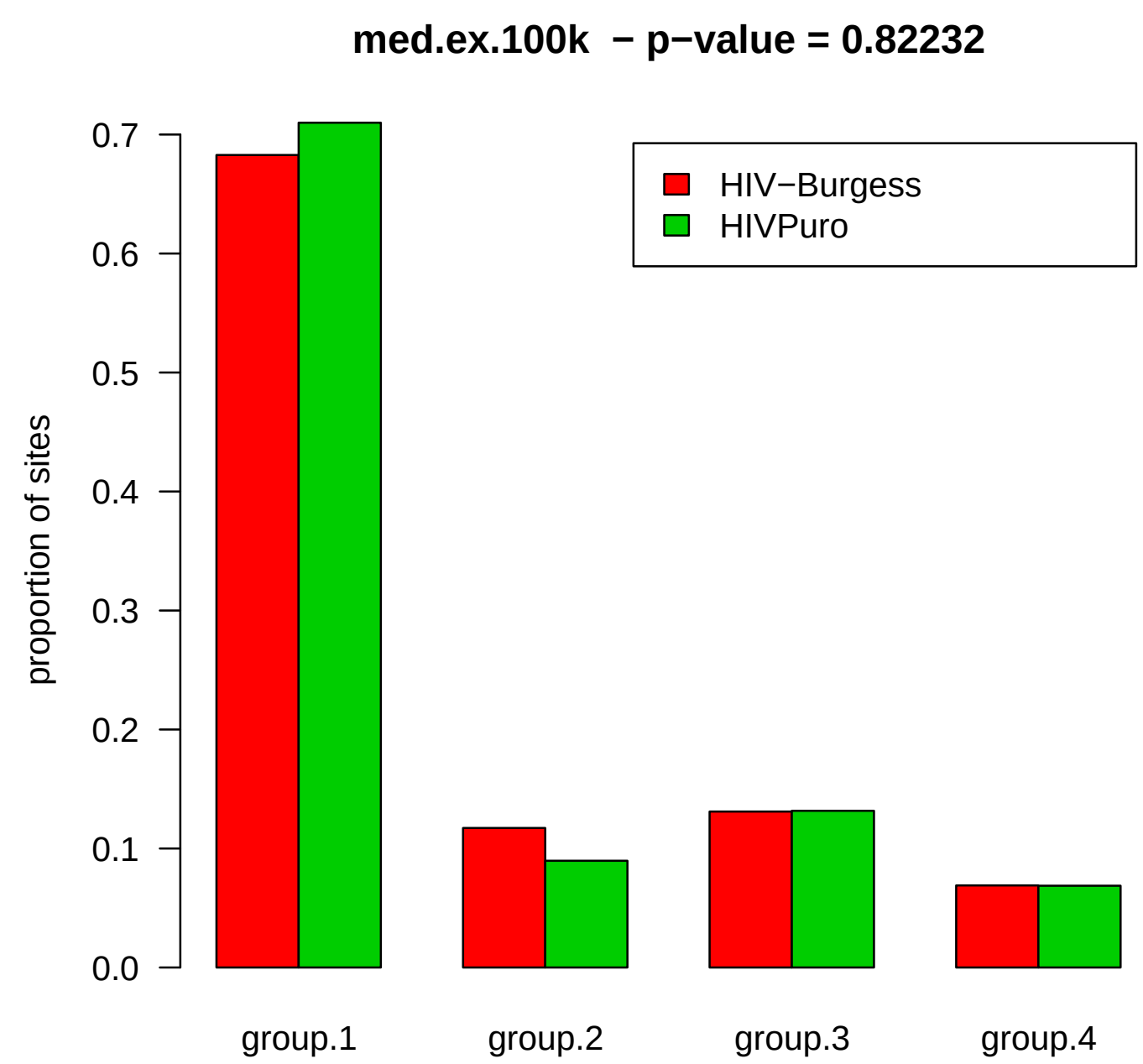

And here we count genes in the upper $1/16^{th}$:

```
Category limits

         lower category        upper
1 0.000000e+00  group.1 3.333333e-07
2 3.333333e-07  group.2 5.000000e-06
3 5.000000e-06  group.3 3.500000e-05
```

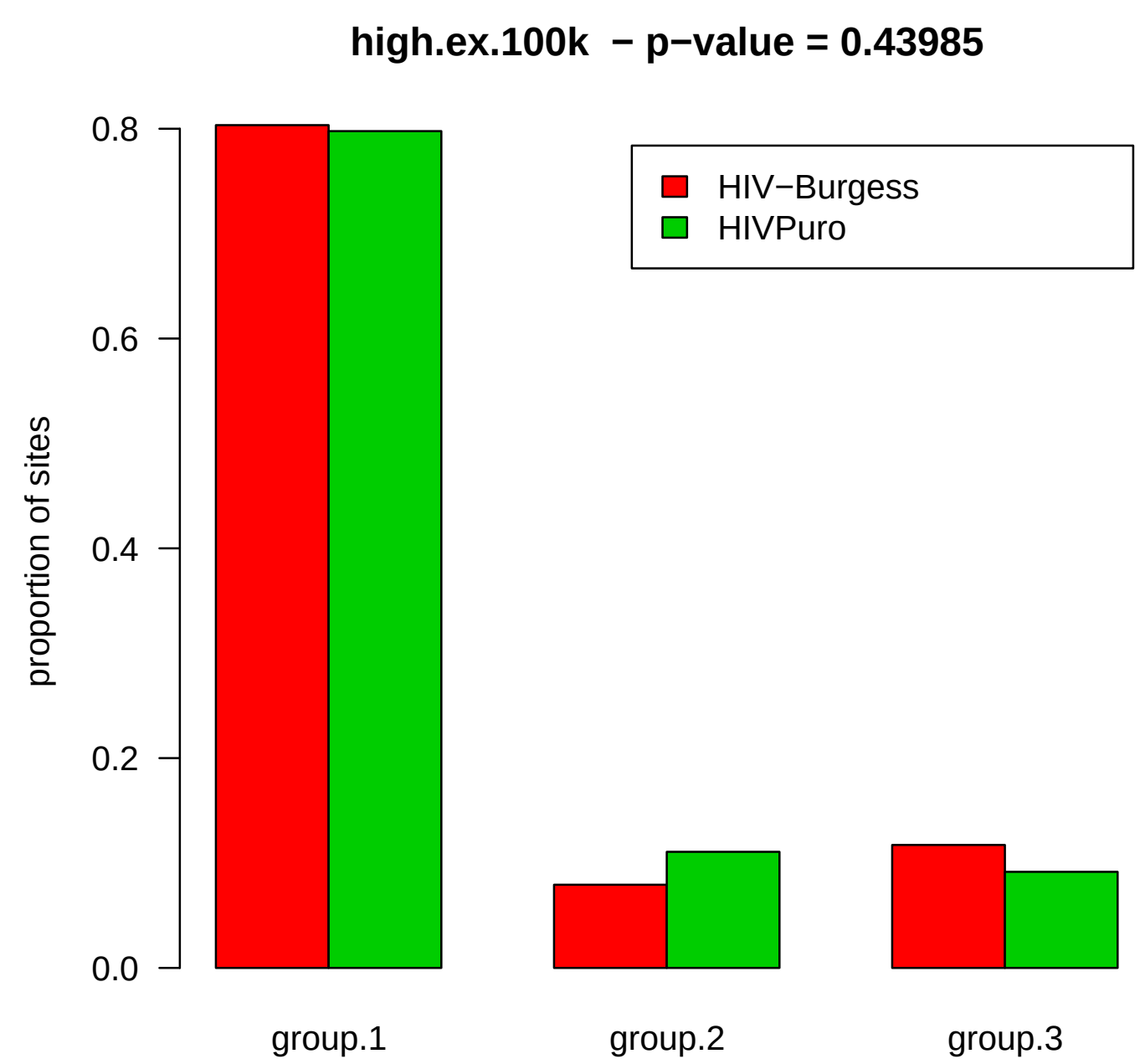

Here the effect of density of CpG islands is studied:

```
Category limits

     lower category    upper
1 0.00e+00  group.1 5.00e-06
2 5.00e-06  group.2 1.00e-05
3 1.00e-05  group.3 1.50e-05
4 1.50e-05  group.4 2.05e-05
5 2.05e-05  group.5 3.20e-05
6 3.20e-05  group.6 5.50e-05
7 5.50e-05  group.7 1.95e-04
```

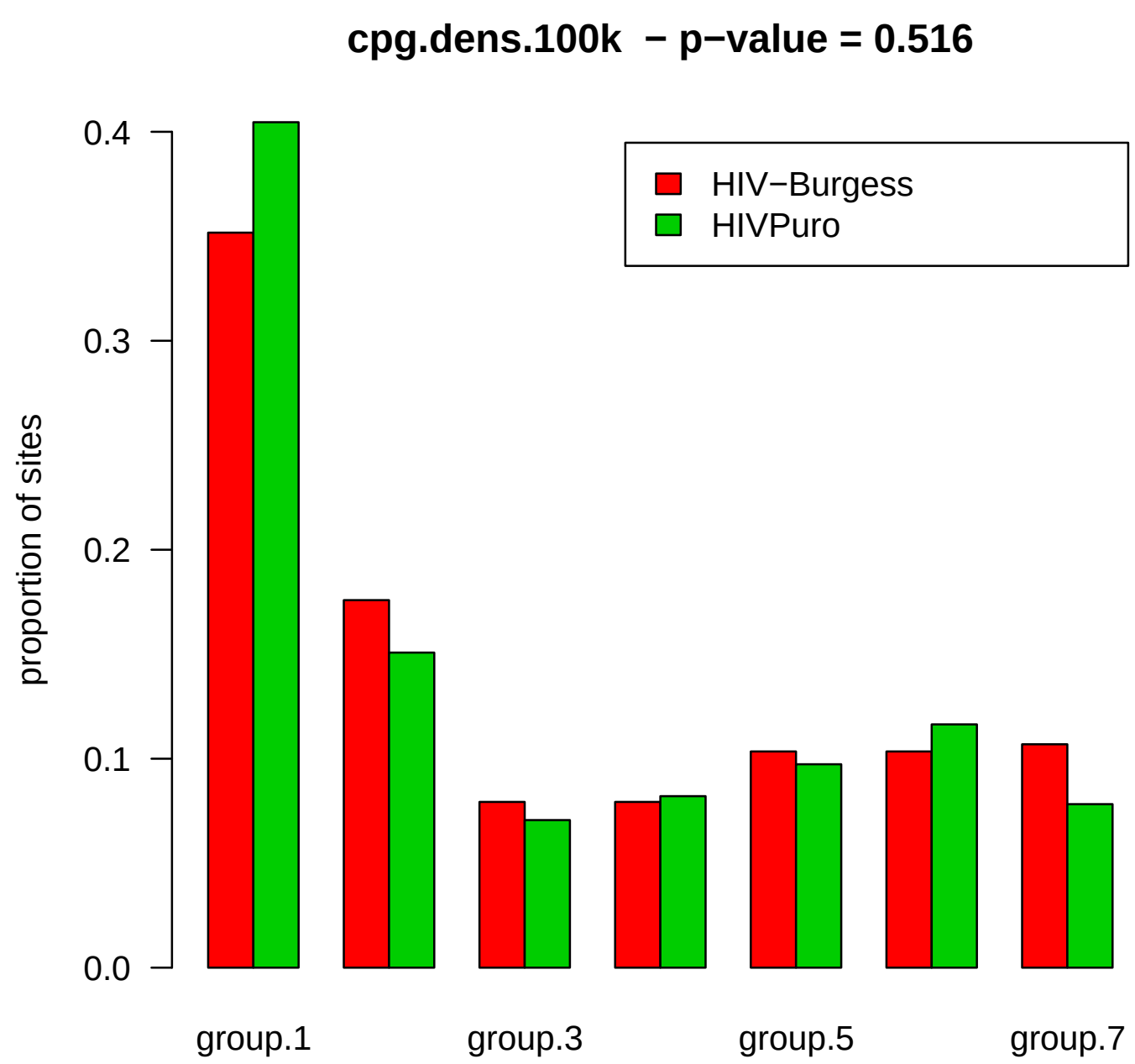

4.4 250 kiloBase Window

In the barplot that follows we examine the association of insertion sites with expression density in a 250 kilobase window surrounding each locus. First, we count just the number of genes on the represented on the chip.

```
 Category limits

          lower category        upper
1 0.000000e+00  group.1 2.000000e-06
2 2.000000e-06  group.2 4.000000e-06
3 4.000000e-06  group.3 5.333333e-06
4 5.333333e-06  group.4 6.954545e-06
5 6.954545e-06  group.5 9.000000e-06
6 9.000000e-06  group.6 1.200000e-05
7 1.200000e-05  group.7 1.600000e-05
8 1.600000e-05  group.8 2.400000e-05
9 2.400000e-05  group.9 7.200000e-05
```

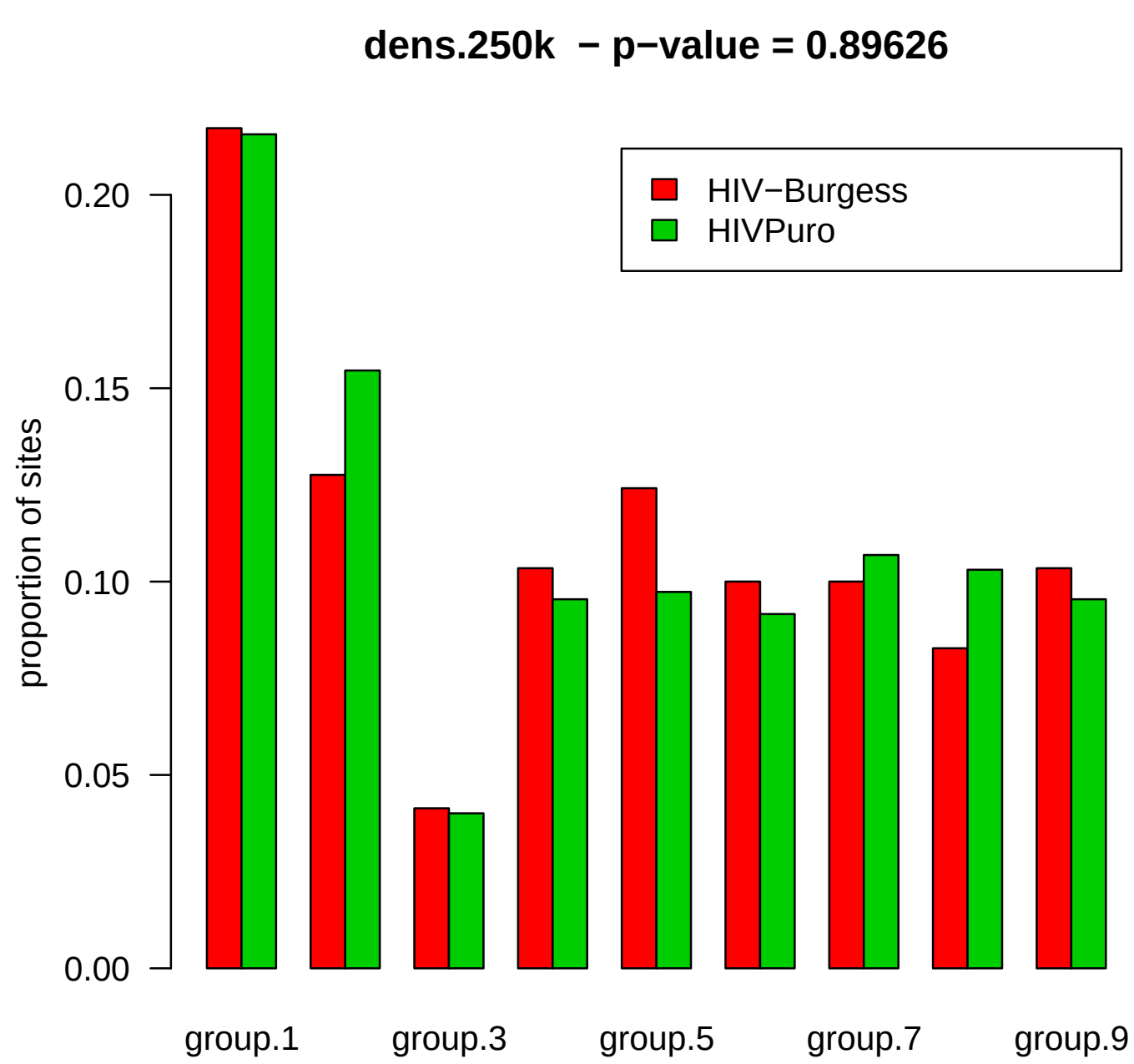

Here are the results for expression density. First, we count just genes that are in the upper half.

```
 Category limits

           lower  category       upper
1 0.000000e+00  group.1 1.333333e-06
2 1.333333e-06  group.2 2.135238e-06
3 2.135238e-06  group.3 4.000000e-06
4 4.000000e-06  group.4 4.647619e-06
5 4.647619e-06  group.5 6.671429e-06
6 6.671429e-06  group.6 9.066667e-06
7 9.066667e-06  group.7 1.400000e-05
8 1.400000e-05  group.8 4.280000e-05
```

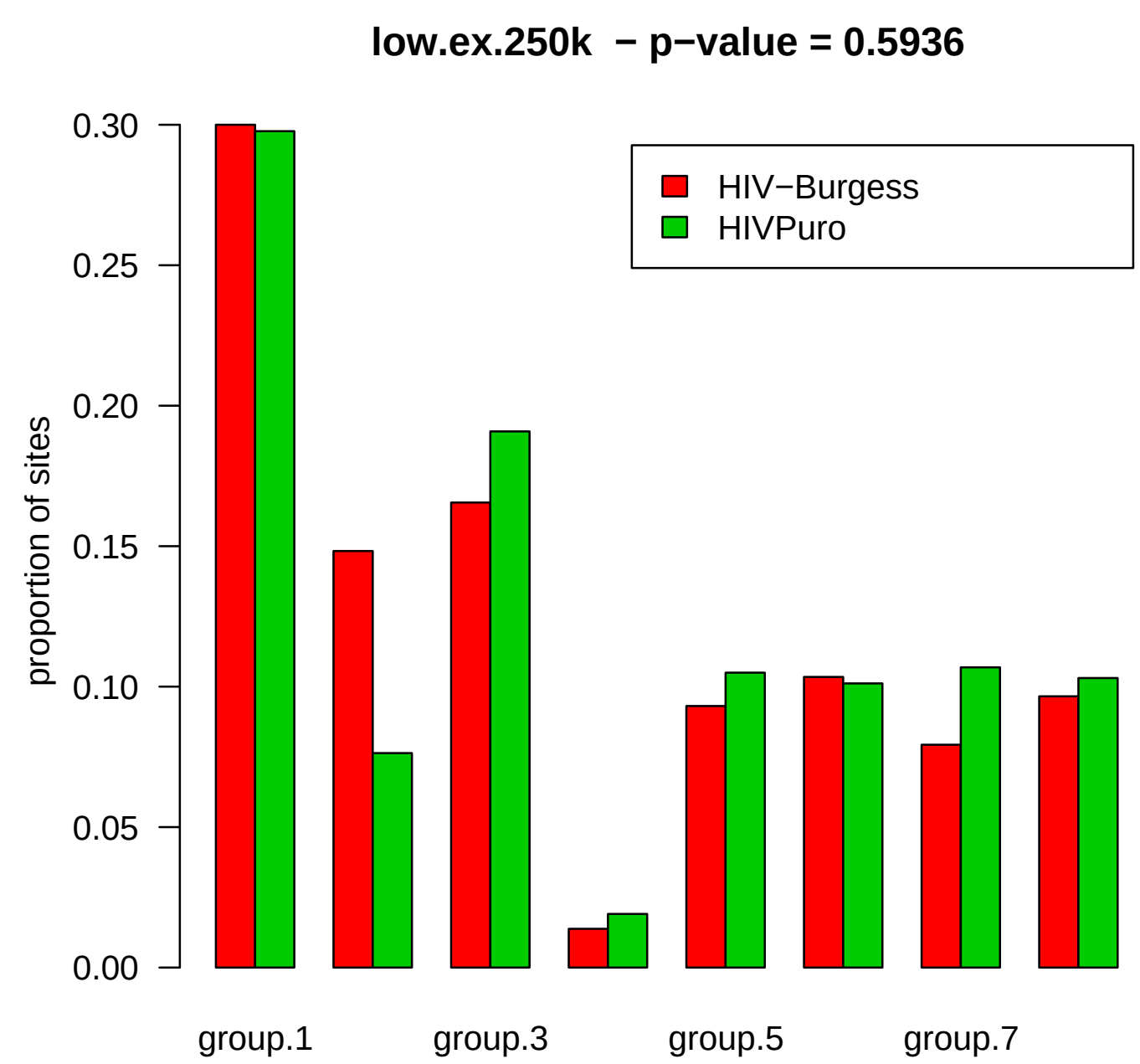

Now we count genes in the upper $1/8^{th}$:

```
Category limits

         lower category        upper
1 0.000000e+00  group.1 1.333333e-06
2 1.333333e-06  group.2 2.106667e-06
3 2.106667e-06  group.3 4.000000e-06
4 4.000000e-06  group.4 5.333333e-06
5 5.333333e-06  group.5 8.000000e-06
6 8.000000e-06  group.6 2.666667e-05
```

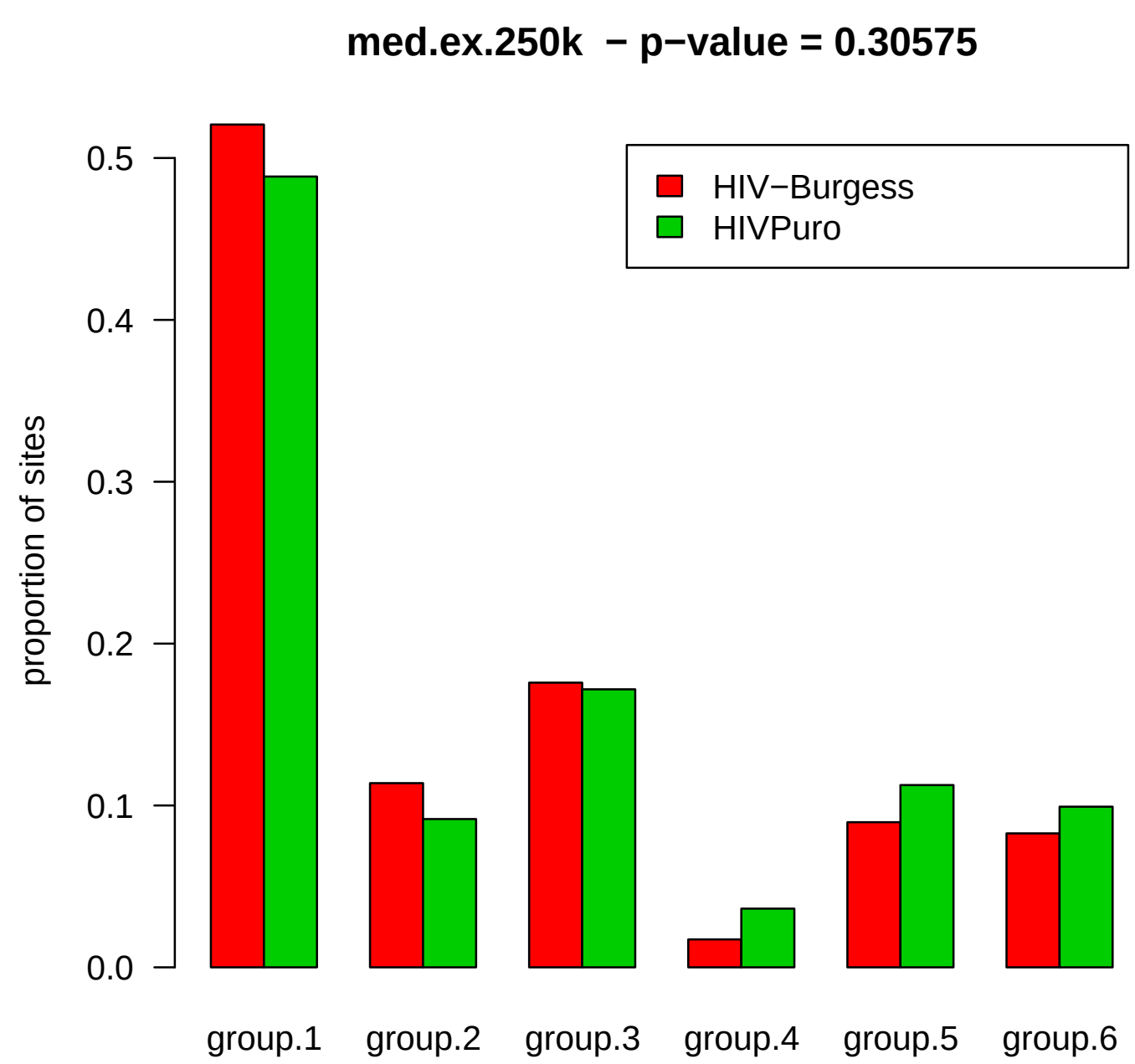

And here we count genes in the upper $1/16^{th}$:

```
 Category limits

     lower category    upper
1 0.00e+00  group.1 1.36e-06
2 1.36e-06  group.2 3.20e-06
3 3.20e-06  group.3 4.00e-06
4 4.00e-06  group.4 1.80e-05
```

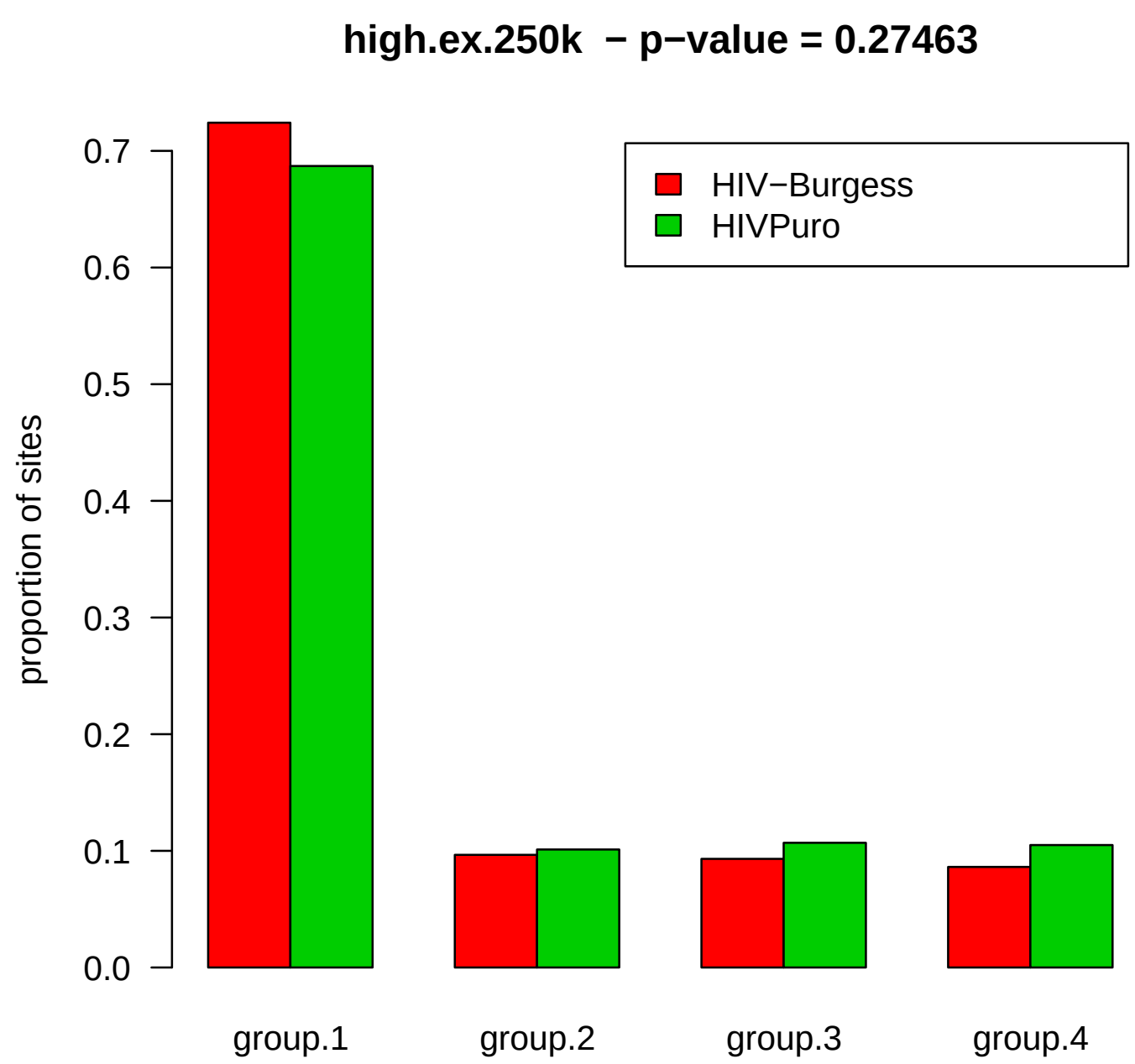

Here the effect of density of CpG islands is studied:

```
Category limits

      lower category    upper
1  0.00e+00  group.1 2.00e-06
2  2.00e-06  group.2 4.00e-06
3  4.00e-06  group.3 8.00e-06
4  8.00e-06  group.4 1.00e-05
5  1.00e-05  group.5 1.20e-05
6  1.20e-05  group.6 1.80e-05
7  1.80e-05  group.7 2.40e-05
8  2.40e-05  group.8 3.40e-05
9  3.40e-05  group.9 5.94e-05
10 5.94e-05 group.10 1.98e-04
```

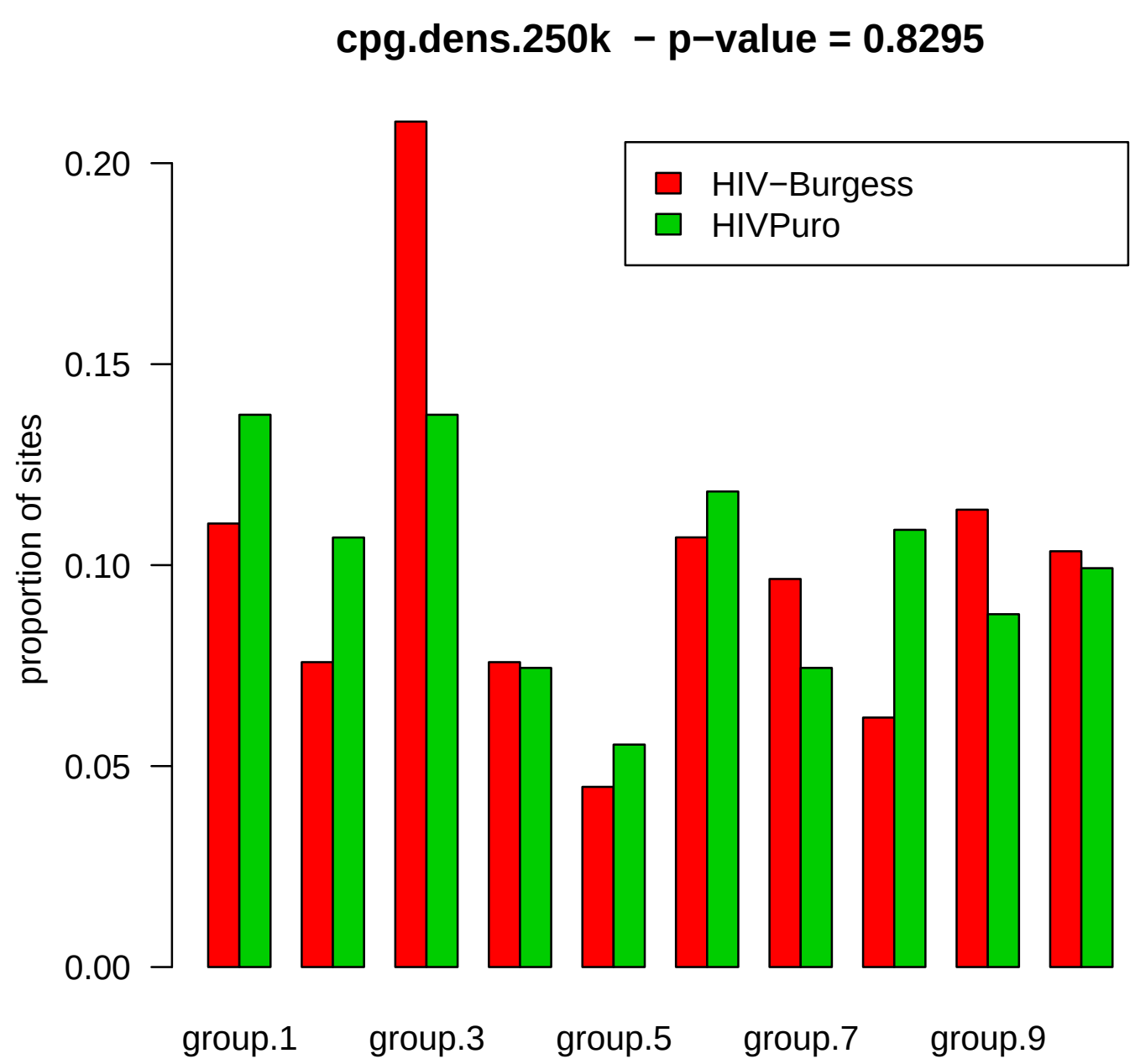

4.5 500 kiloBase Window

In the barplot that follows we examine the association of insertion sites with expression density in a 500 kilobase window surrounding each locus. First, we count just the number of genes on the represented on the chip.

```
 Category limits

          lower category        upper
1  0.000000e+00  group.1 1.333333e-06
2  1.333333e-06  group.2 2.333333e-06
3  2.333333e-06  group.3 3.833333e-06
4  3.833333e-06  group.4 5.147619e-06
5  5.147619e-06  group.5 6.878571e-06
6  6.878571e-06  group.6 8.666667e-06
7  8.666667e-06  group.7 1.167000e-05
8  1.167000e-05  group.8 1.580000e-05
9  1.580000e-05  group.9 2.433333e-05
10 2.433333e-05 group.10 7.433333e-05
```

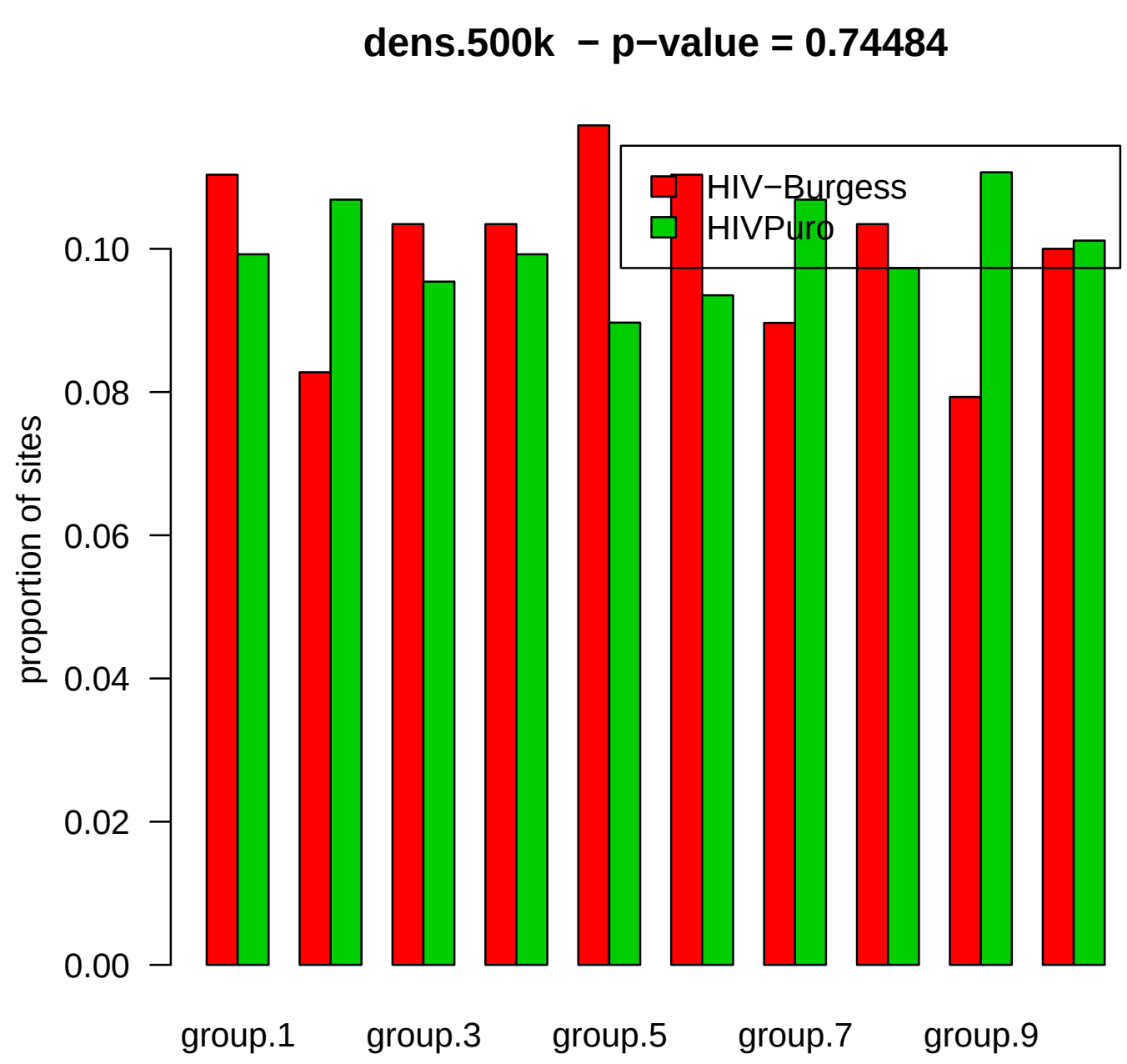

Here are the results for expression density. First, we count just genes that are in the upper half.

```
 Category limits

         lower category        upper
1 0.000000e+00  group.1 1.000000e-06
2 1.000000e-06  group.2 2.000000e-06
3 2.000000e-06  group.3 2.500000e-06
4 2.500000e-06  group.4 3.500000e-06
5 3.500000e-06  group.5 4.666667e-06
6 4.666667e-06  group.6 6.273333e-06
7 6.273333e-06  group.7 8.802222e-06
8 8.802222e-06  group.8 1.400000e-05
9 1.400000e-05  group.9 3.633333e-05
```

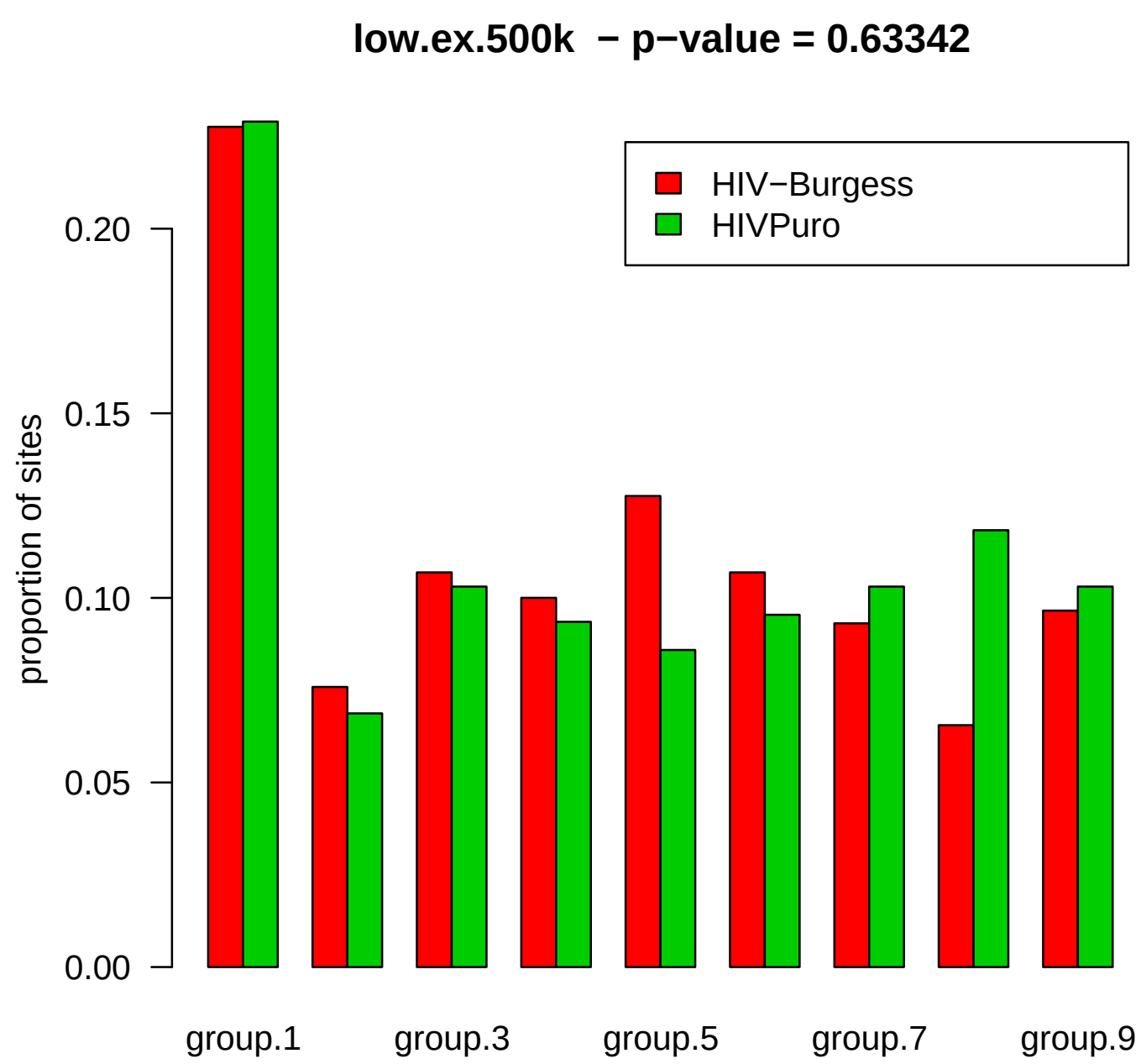

Now we count genes in the upper $1/8^{th}$:

```
Category limits

         lower category        upper
1 0.000000e+00  group.1 4.000000e-07
2 4.000000e-07  group.2 1.000000e-06
3 1.000000e-06  group.3 2.000000e-06
4 2.000000e-06  group.4 2.333333e-06
5 2.333333e-06  group.5 3.333333e-06
6 3.333333e-06  group.6 4.666667e-06
7 4.666667e-06  group.7 7.950000e-06
8 7.950000e-06  group.8 2.000000e-05
```

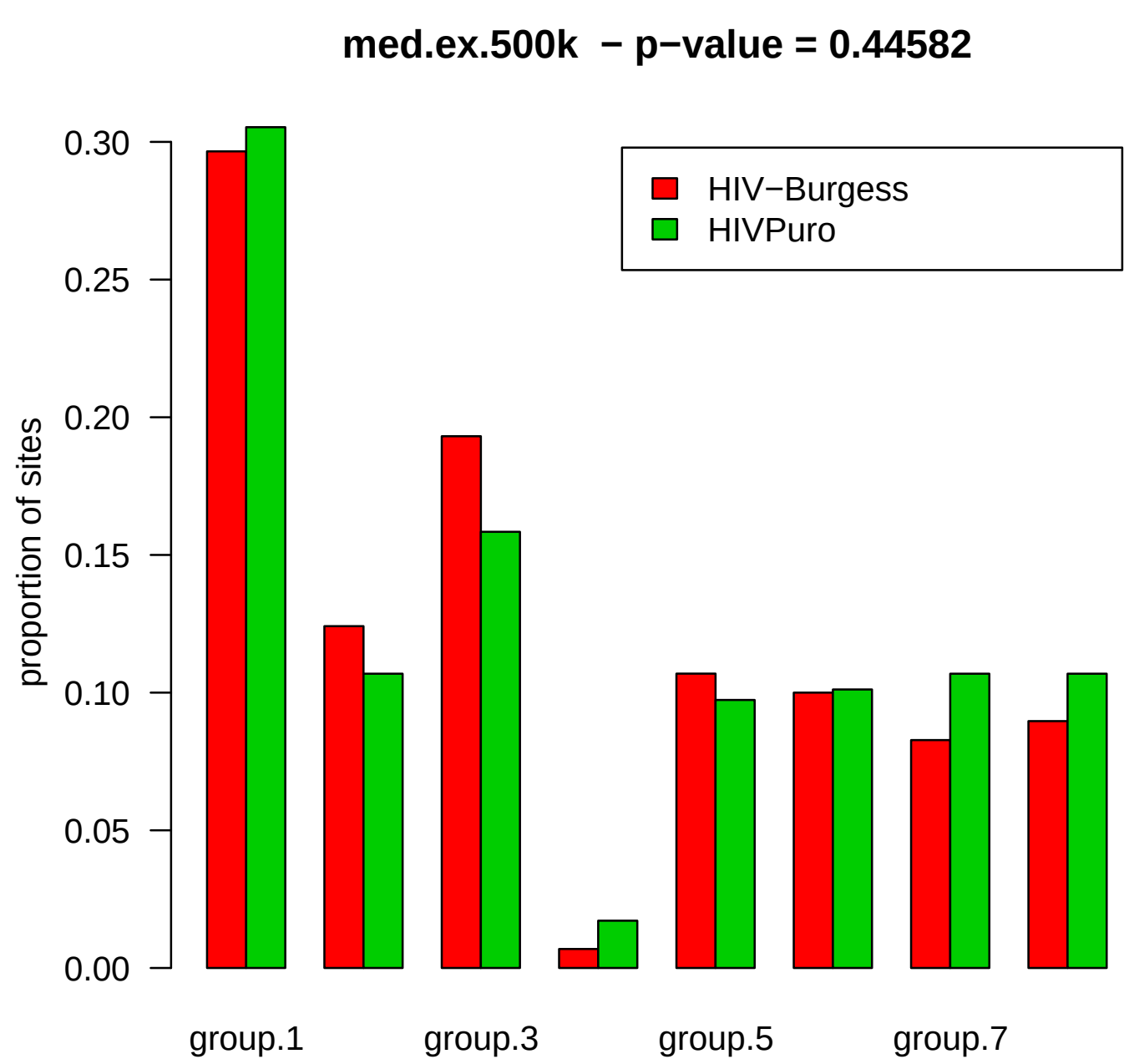

And here we count genes in the upper $1/16^{th}$:

```
Category limits

         lower category        upper
1 0.000000e+00  group.1 5.000000e-07
2 5.000000e-07  group.2 1.000000e-06
3 1.000000e-06  group.3 2.000000e-06
4 2.000000e-06  group.4 2.333333e-06
5 2.333333e-06  group.5 4.000000e-06
6 4.000000e-06  group.6 1.400000e-05
```

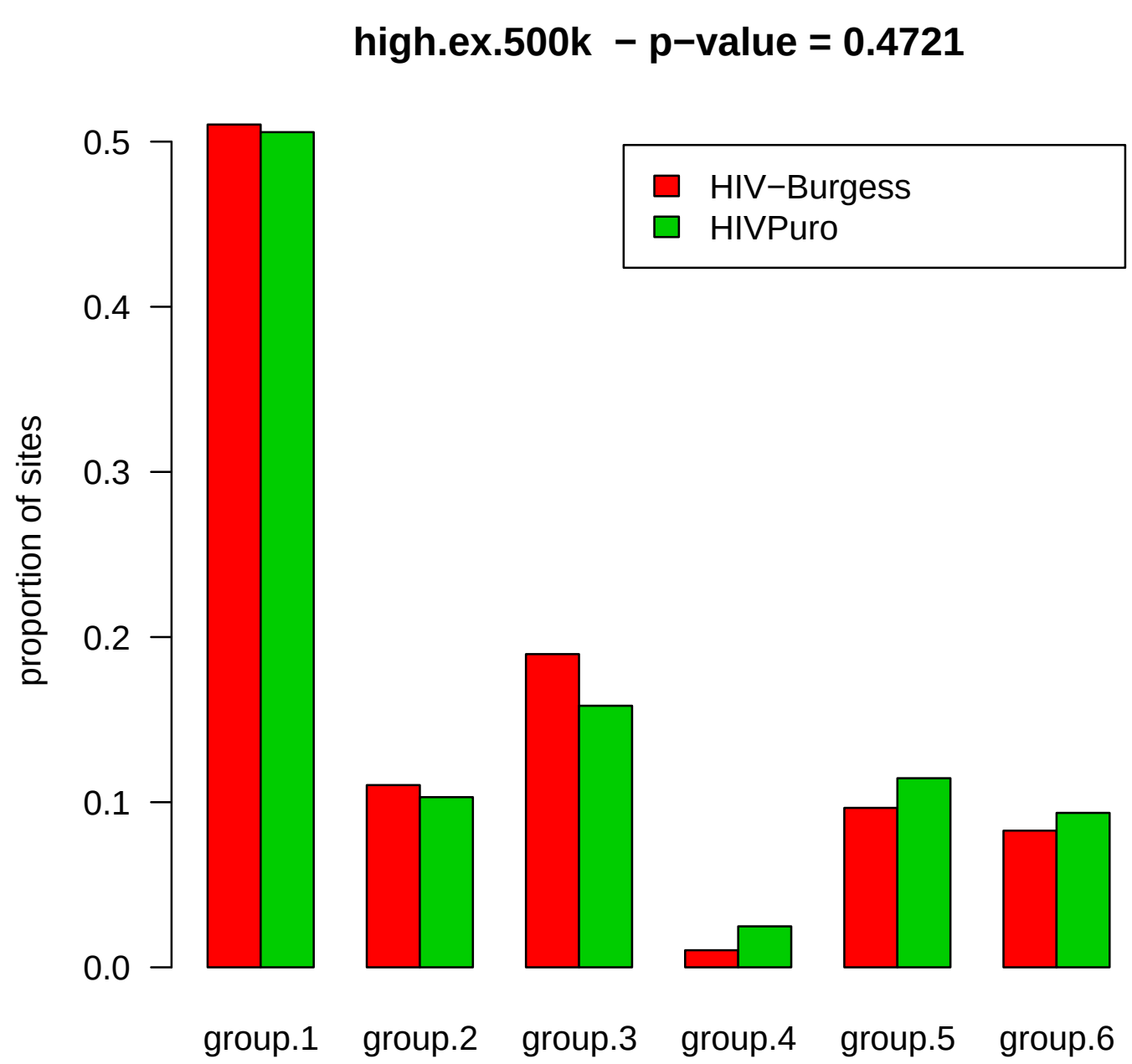

Here the effect of density of CpG islands is studied:

```
Category limits

      lower  category    upper
1  0.00e+00   group.1 2.00e-06
2  2.00e-06   group.2 4.00e-06
3  4.00e-06   group.3 7.00e-06
4  7.00e-06   group.4 9.00e-06
5  9.00e-06   group.5 1.20e-05
6  1.20e-05   group.6 1.70e-05
7  1.70e-05   group.7 2.30e-05
8  2.30e-05   group.8 3.40e-05
9  3.40e-05   group.9 5.57e-05
10 5.57e-05  group.10 1.83e-04
```

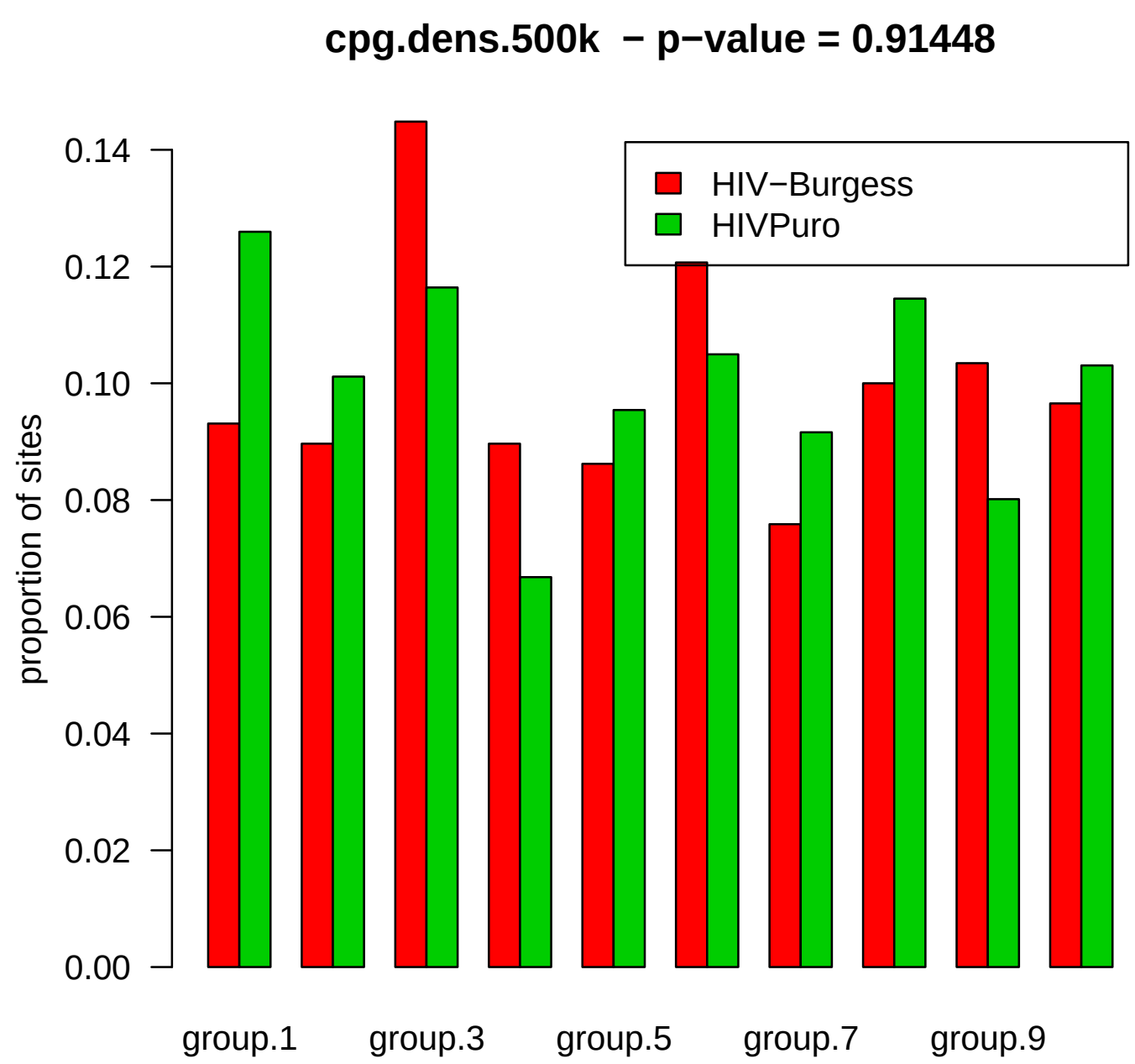

4.6 1 megaBase Window

In the barplot that follows we examine the association of insertion sites with expression density in a 1 megabase window surrounding each locus. First, we count just the number of genes on the represented on the chip.

```
 Category limits

          lower category        upper
1  0.000000e+00  group.1 1.345833e-06
2  1.345833e-06  group.2 2.500000e-06
3  2.500000e-06  group.3 3.795000e-06
4  3.795000e-06  group.4 5.083333e-06
5  5.083333e-06  group.5 6.500000e-06
6  6.500000e-06  group.6 8.093333e-06
7  8.093333e-06  group.7 1.133385e-05
8  1.133385e-05  group.8 1.565429e-05
9  1.565429e-05  group.9 2.133333e-05
10 2.133333e-05 group.10 5.516667e-05
```

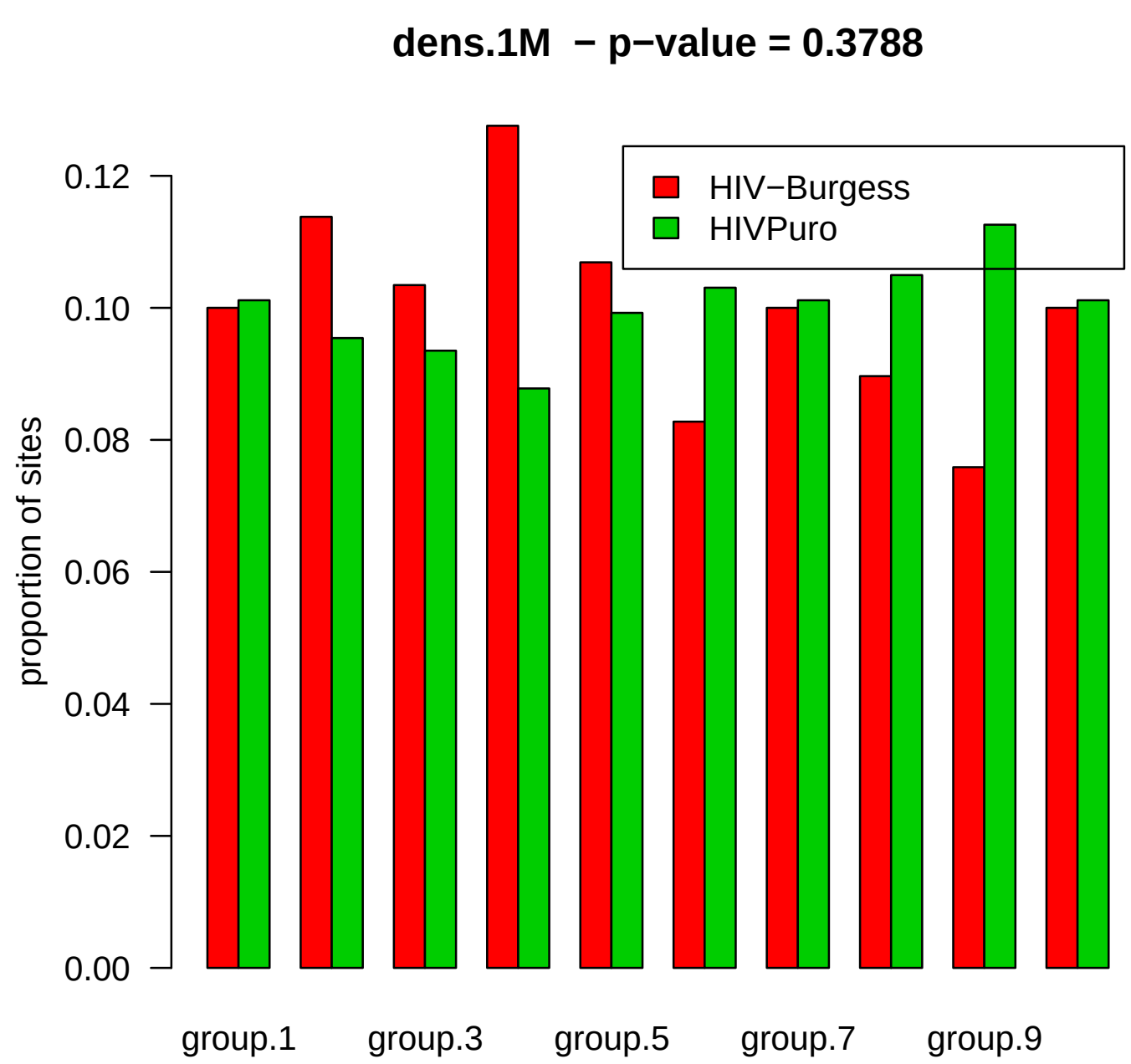

Here are the results for expression density. First, we count just genes that are in the upper half.

```
 Category limits

          lower category        upper
1  0.000000e+00  group.1 3.333333e-07
2  3.333333e-07  group.2 1.000000e-06
3  1.000000e-06  group.3 1.730000e-06
4  1.730000e-06  group.4 2.500000e-06
5  2.500000e-06  group.5 3.308333e-06
6  3.308333e-06  group.6 4.333333e-06
7  4.333333e-06  group.7 5.750000e-06
8  5.750000e-06  group.8 8.000000e-06
9  8.000000e-06  group.9 1.248500e-05
10 1.248500e-05 group.10 2.691667e-05
```

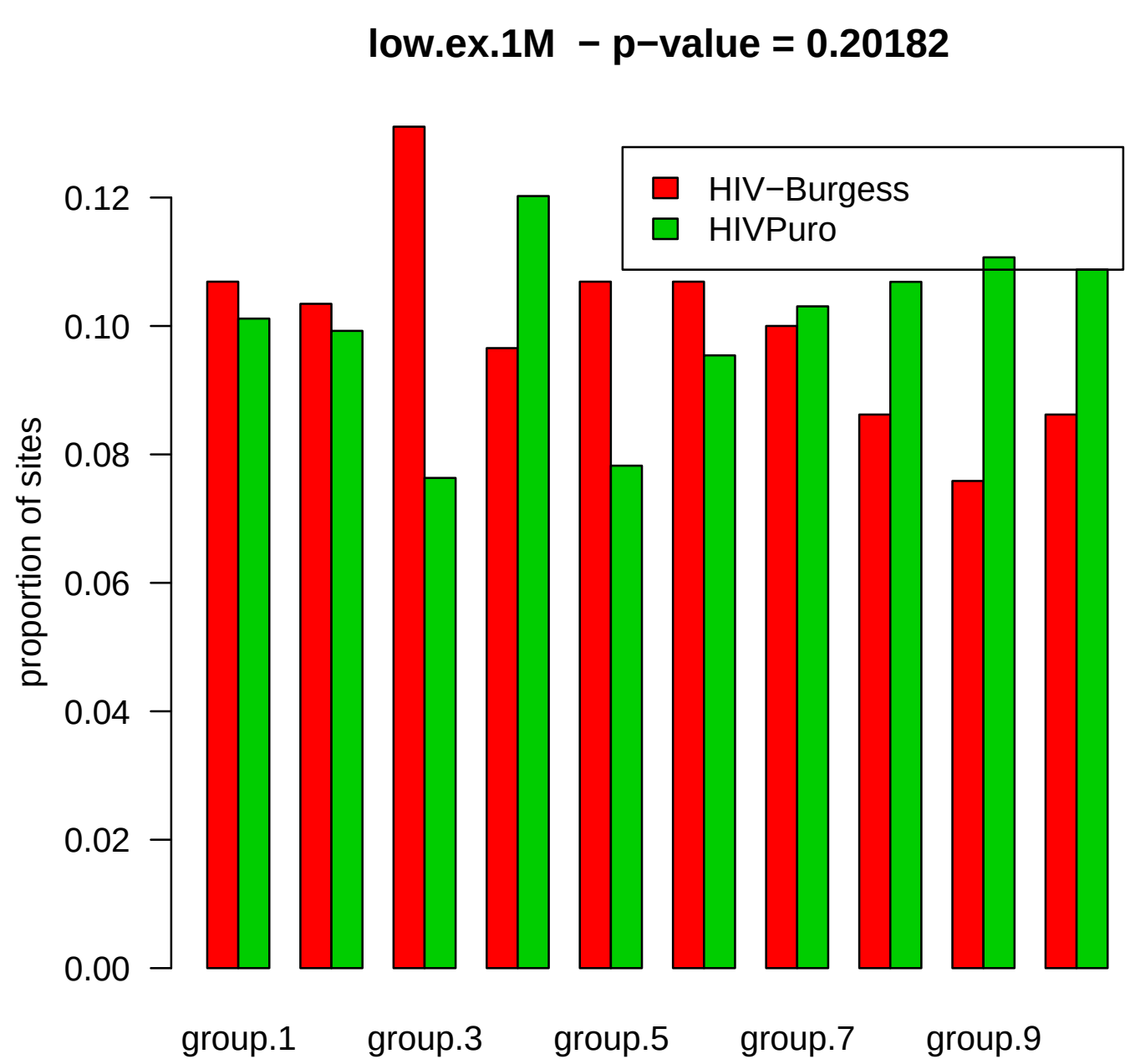

Now we count genes in the upper $1/8^{th}$:

```
Category limits

         lower category        upper
1 0.000000e+00  group.1 2.500000e-07
2 2.500000e-07  group.2 6.666667e-07
3 6.666667e-07  group.3 1.083333e-06
4 1.083333e-06  group.4 1.666667e-06
5 1.666667e-06  group.5 2.166072e-06
6 2.166072e-06  group.6 3.000000e-06
7 3.000000e-06  group.7 4.000000e-06
8 4.000000e-06  group.8 6.803333e-06
9 6.803333e-06  group.9 1.400000e-05
```

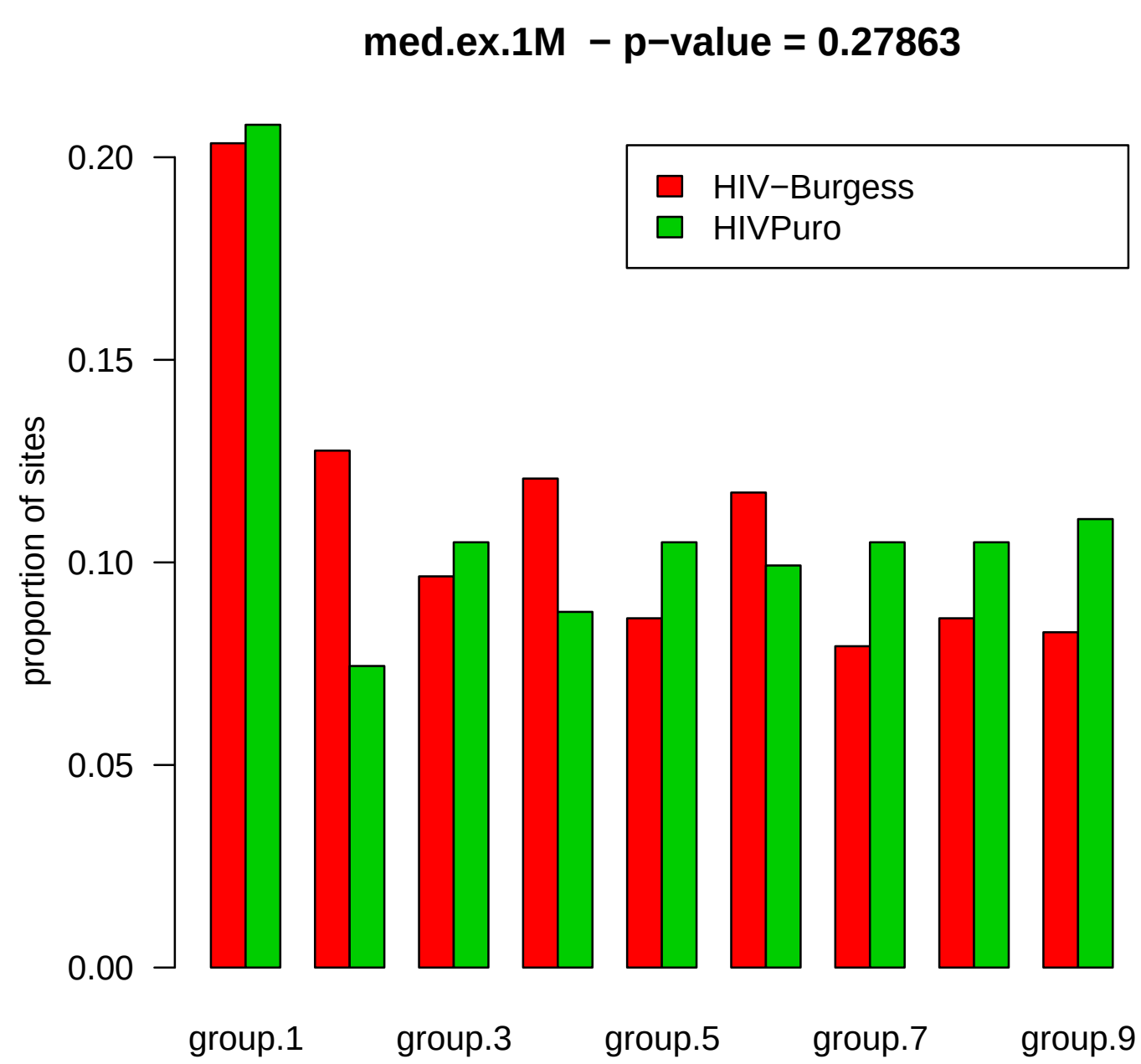

And here we count genes in the upper $1/16^{th}$:

```
Category limits

         lower category        upper
1 0.000000e+00  group.1 4.000000e-07
2 4.000000e-07  group.2 7.500000e-07
3 7.500000e-07  group.3 1.000000e-06
4 1.000000e-06  group.4 1.500000e-06
5 1.500000e-06  group.5 2.166667e-06
6 2.166667e-06  group.6 3.500000e-06
7 3.500000e-06  group.7 8.500000e-06
```

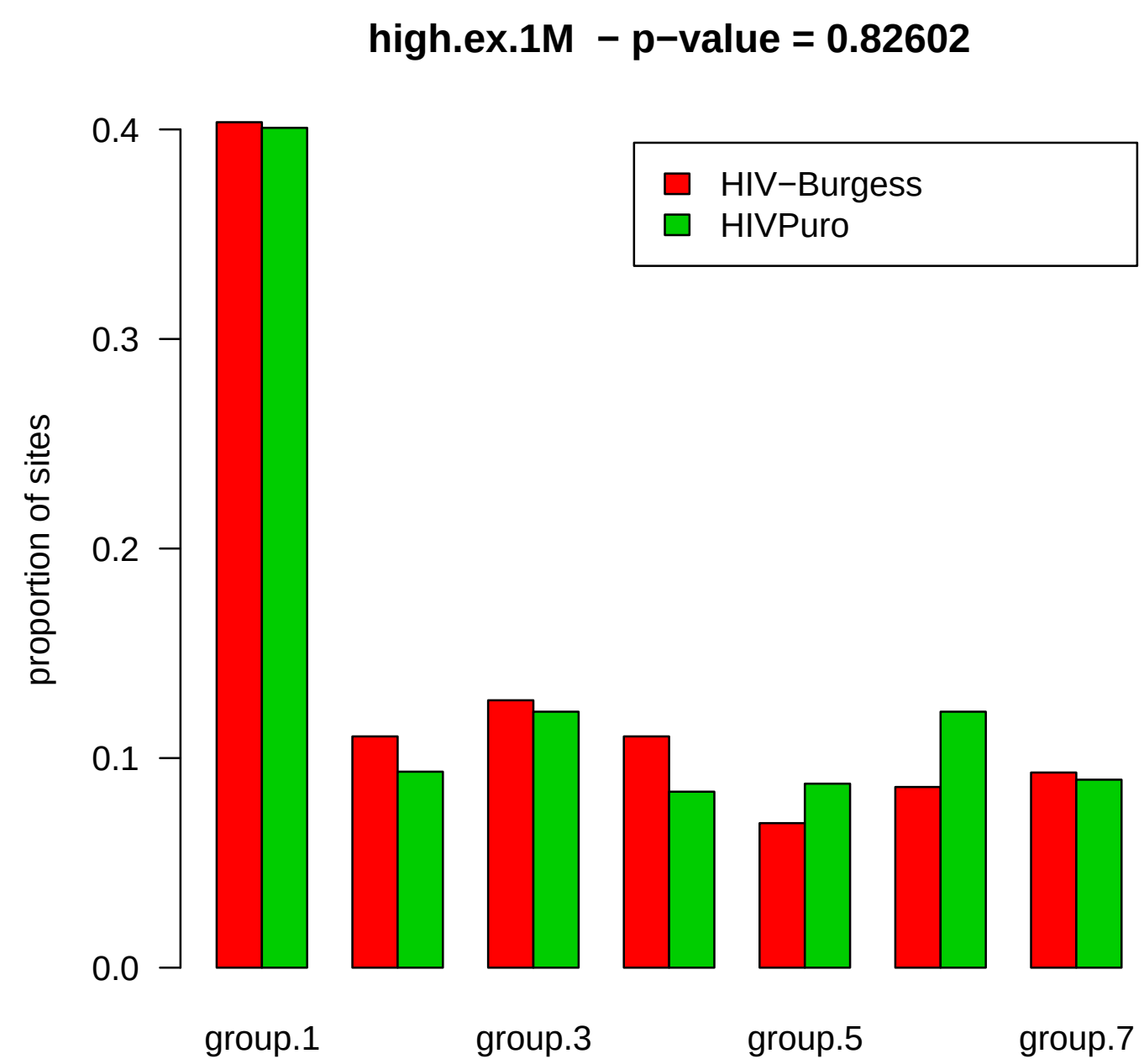

Here the effect of density of CpG islands is studied:

```
Category limits

        lower  category     upper
1  0.000e+00   group.1 3.000e-06
2  3.000e-06   group.2 4.300e-06
3  4.300e-06   group.3 6.500e-06
4  6.500e-06   group.4 9.000e-06
5  9.000e-06   group.5 1.250e-05
6  1.250e-05   group.6 1.800e-05
7  1.800e-05   group.7 2.250e-05
8  2.250e-05   group.8 3.170e-05
9  3.170e-05   group.9 5.235e-05
10 5.235e-05  group.10 1.685e-04
```

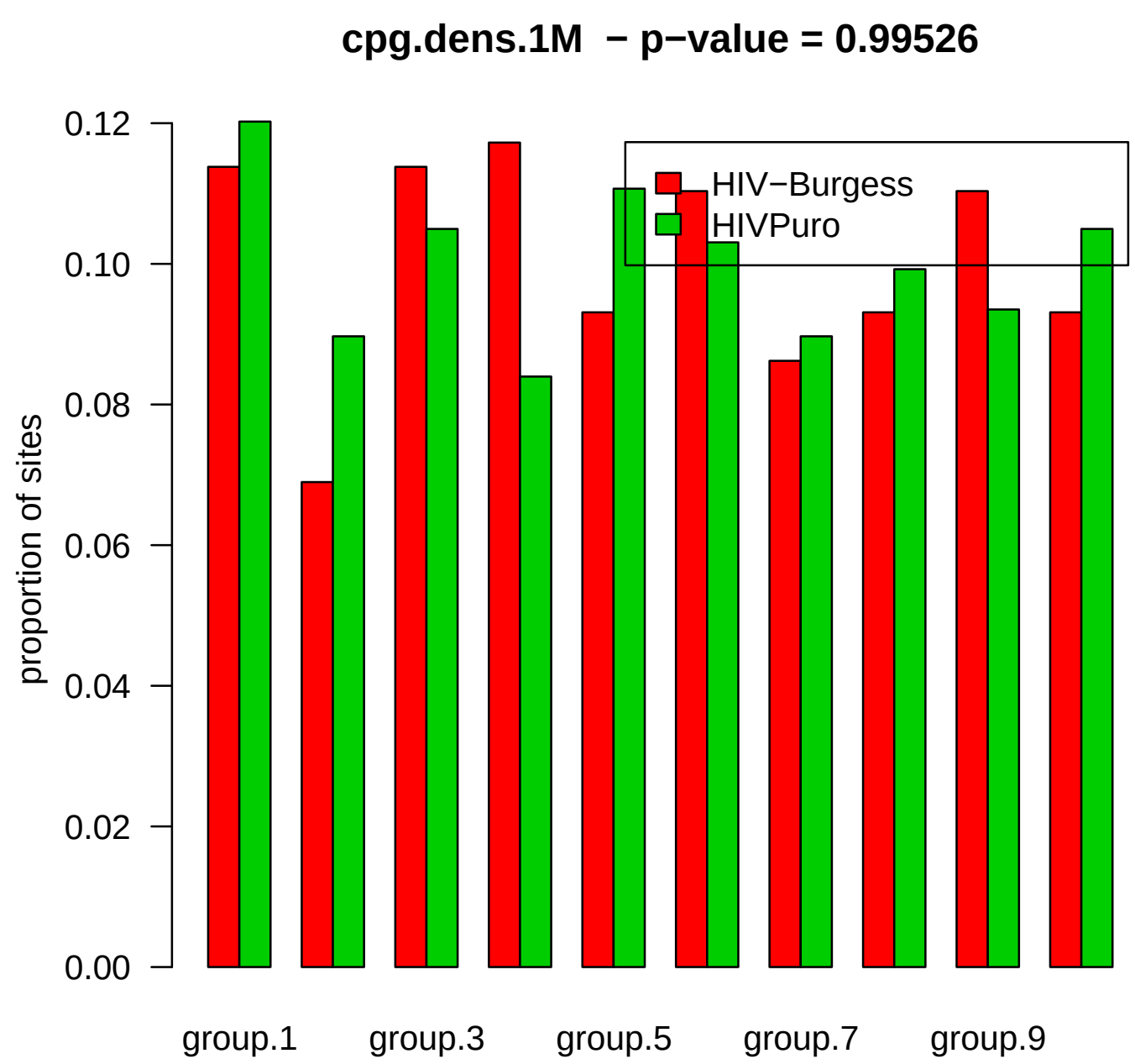

4.7 2 megaBase Window

In the barplot that follows we examine the association of insertion sites with expression density in a 2 megabase window surrounding each locus. First, we count just the number of genes on the represented on the chip.

```
 Category limits

          lower category        upper
1  0.000000e+00  group.1 1.566667e-06
2  1.566667e-06  group.2 2.500000e-06
3  2.500000e-06  group.3 3.541667e-06
4  3.541667e-06  group.4 4.626667e-06
5  4.626667e-06  group.5 6.021429e-06
6  6.021429e-06  group.6 8.185000e-06
7  8.185000e-06  group.7 1.120083e-05
8  1.120083e-05  group.8 1.518833e-05
9  1.518833e-05  group.9 1.941667e-05
10 1.941667e-05 group.10 3.912500e-05
```

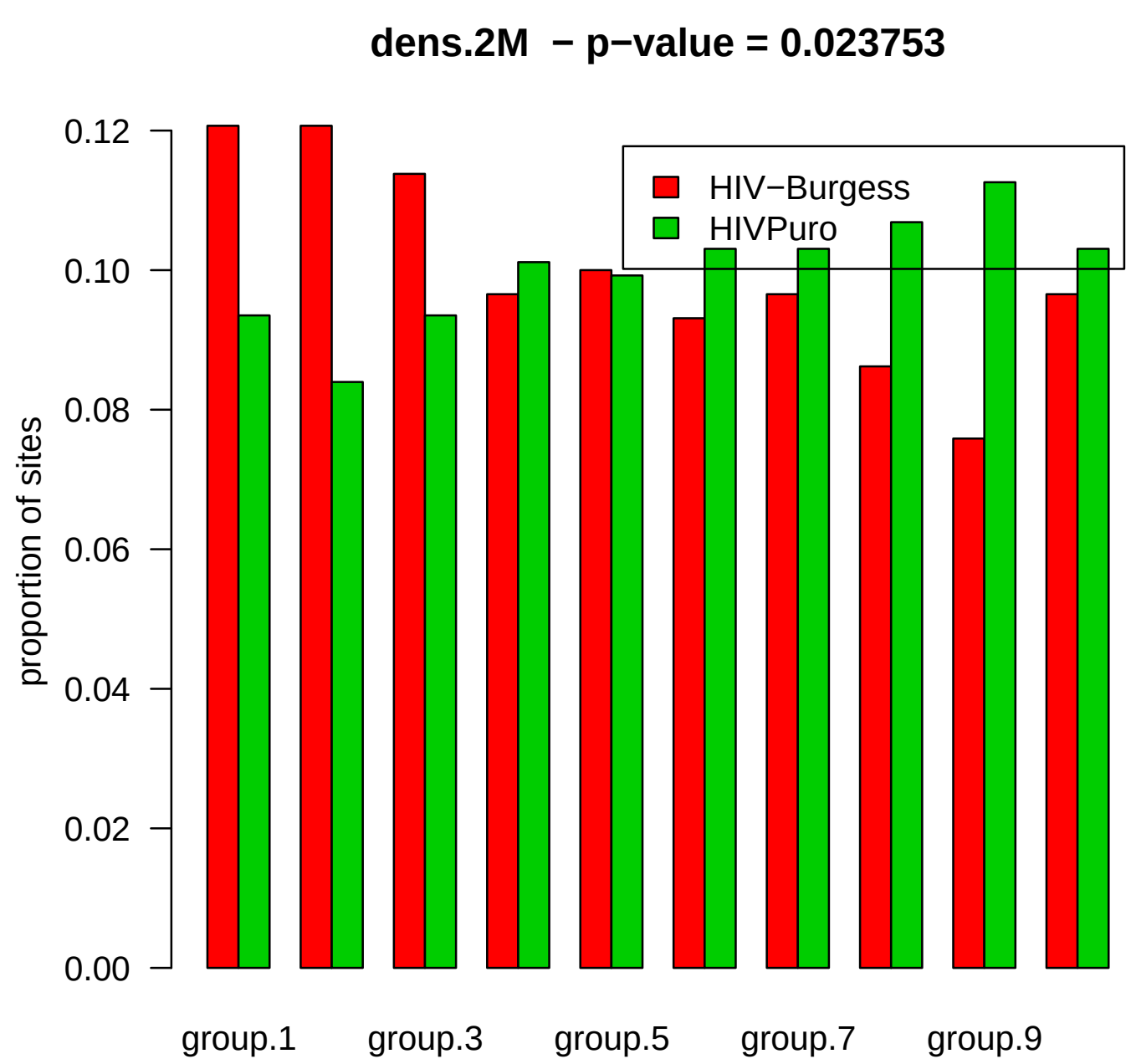

Here are the results for expression density. First, we count just genes that are in the upper half.

```
 Category limits

          lower category        upper
1  0.000000e+00  group.1 5.000000e-07
2  5.000000e-07  group.2 1.082857e-06
3  1.082857e-06  group.3 1.591905e-06
4  1.591905e-06  group.4 2.102619e-06
5  2.102619e-06  group.5 2.766667e-06
6  2.766667e-06  group.6 3.915000e-06
7  3.915000e-06  group.7 5.941667e-06
8  5.941667e-06  group.8 7.567857e-06
9  7.567857e-06  group.9 9.969167e-06
10 9.969167e-06 group.10 2.093333e-05
```

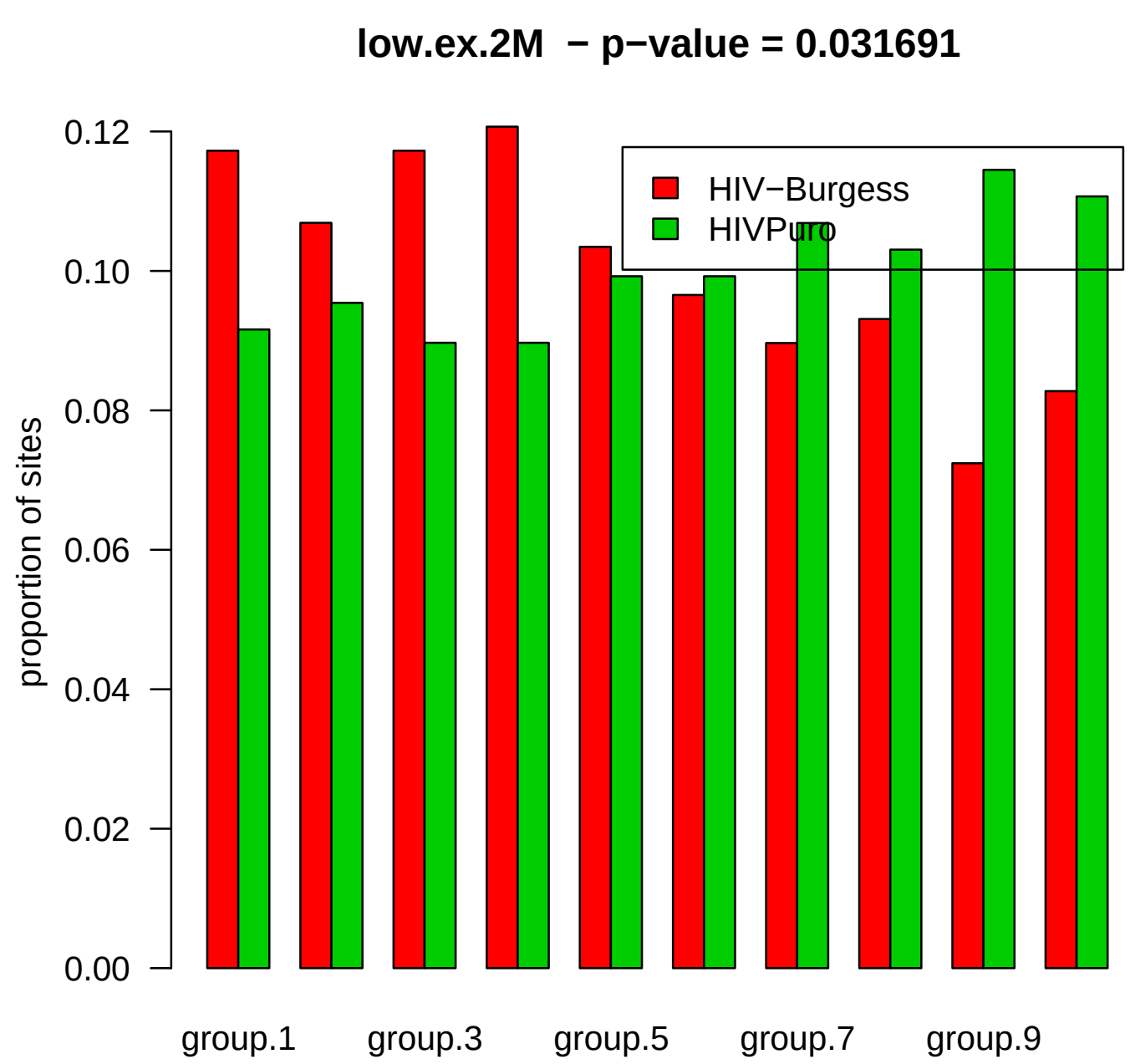

Now we count genes in the upper $1/8^{th}$:

```
Category limits

          lower  category        upper
1  0.000000e+00   group.1 1.461538e-07
2  1.461538e-07   group.2 4.707143e-07
3  4.707143e-07   group.3 7.500000e-07
4  7.500000e-07   group.4 1.041667e-06
5  1.041667e-06   group.5 1.416667e-06
6  1.416667e-06   group.6 1.958333e-06
7  1.958333e-06   group.7 2.810811e-06
8  2.810811e-06   group.8 3.995000e-06
9  3.995000e-06   group.9 5.570833e-06
10 5.570833e-06 group.10 1.126667e-05
```

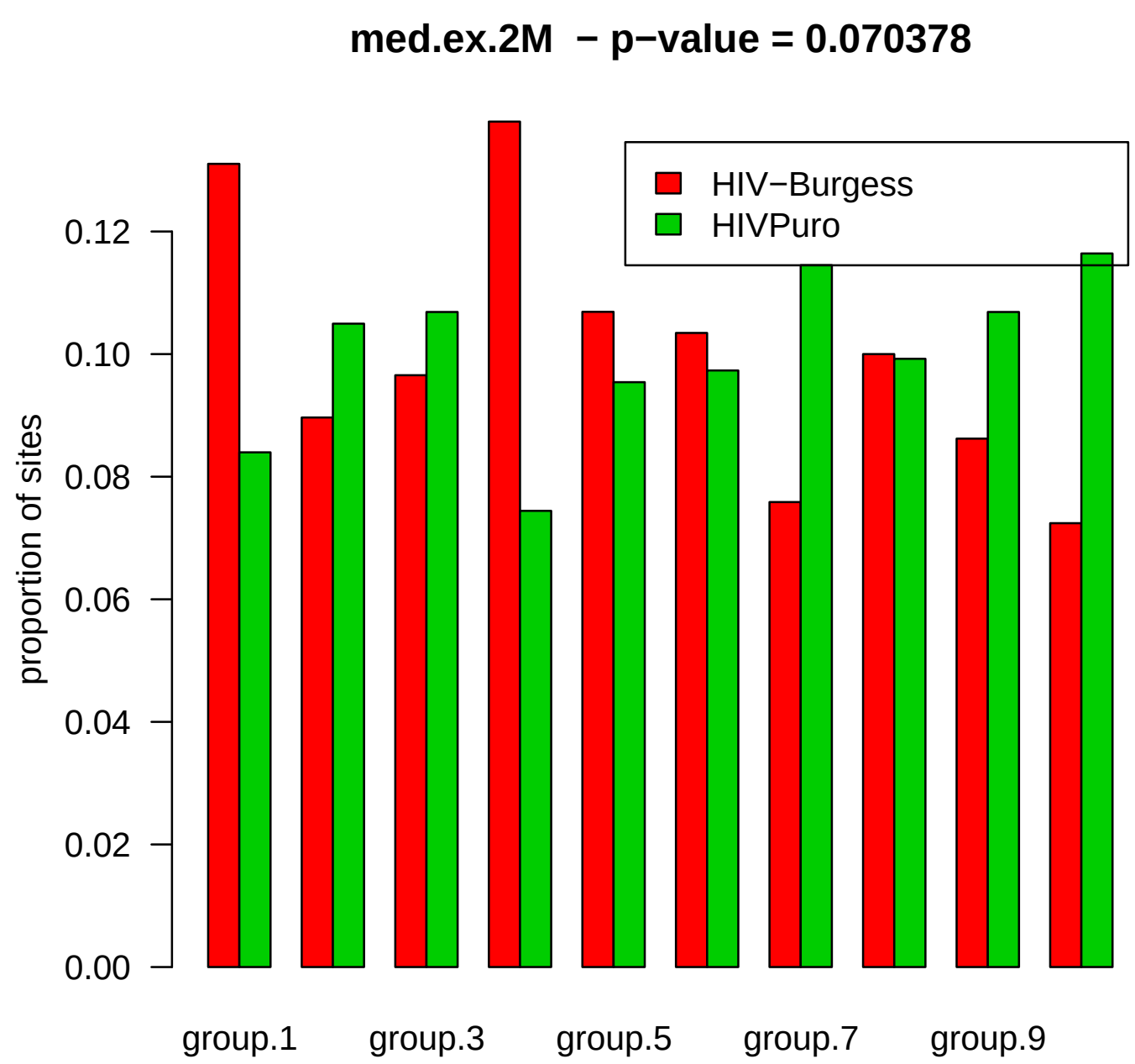

And here we count genes in the upper $1/16^{th}$:

```
Category limits

         lower category        upper
1 0.000000e+00  group.1 1.000000e-07
2 1.000000e-07  group.2 2.500000e-07
3 2.500000e-07  group.3 5.000000e-07
4 5.000000e-07  group.4 6.898810e-07
5 6.898810e-07  group.5 1.000000e-06
6 1.000000e-06  group.6 1.375000e-06
7 1.375000e-06  group.7 1.923333e-06
8 1.923333e-06  group.8 2.737500e-06
9 2.737500e-06  group.9 7.433333e-06
```

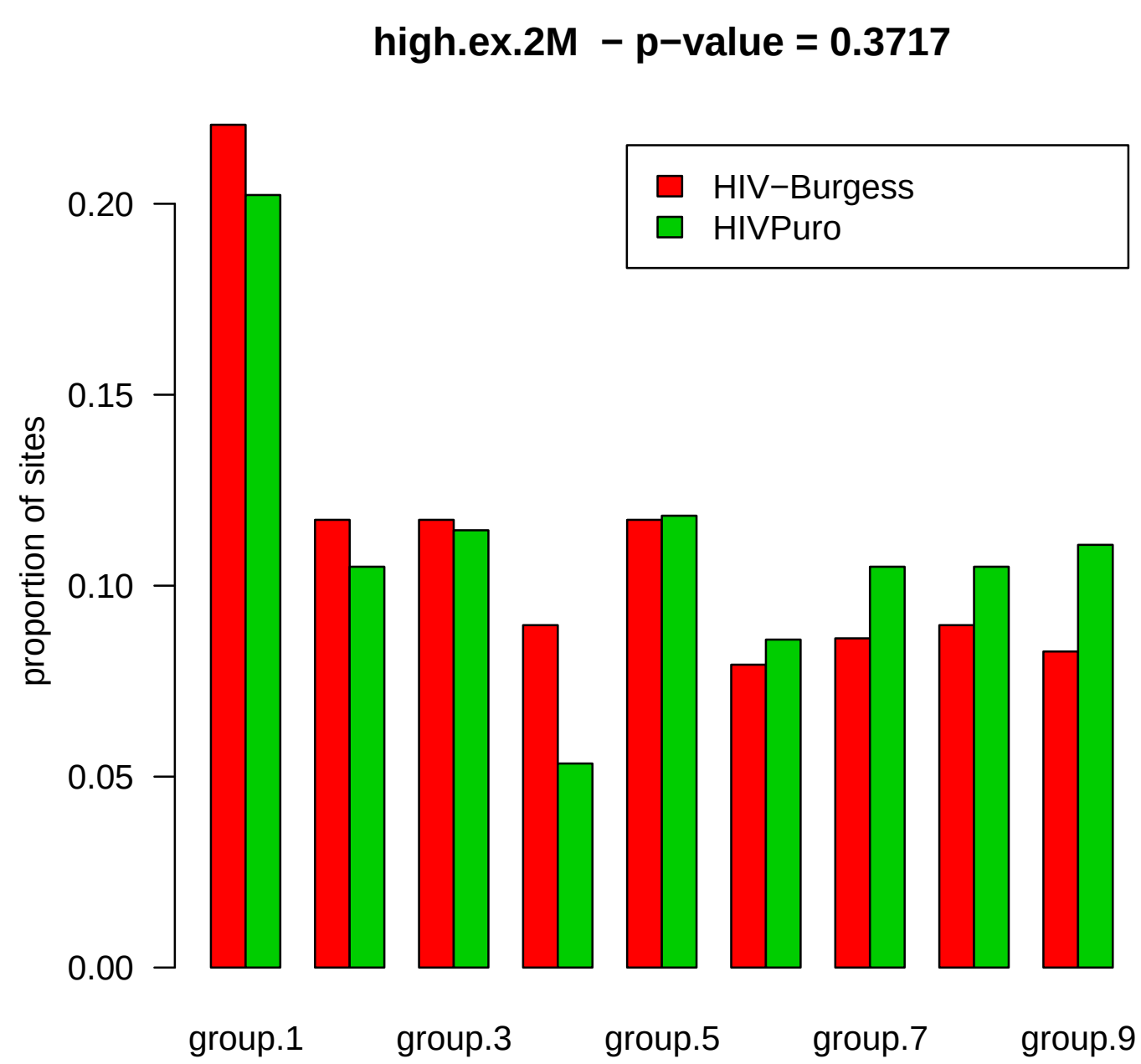

Here the effect of density of CpG islands is studied:

```
Category limits

          lower  category        upper
1  0.000000e+00   group.1 2.825000e-06
2  2.825000e-06   group.2 4.500000e-06
3  4.500000e-06   group.3 5.750000e-06
4  5.750000e-06   group.4 8.253564e-06
5  8.253564e-06   group.5 1.225000e-05
6  1.225000e-05   group.6 1.770000e-05
7  1.770000e-05   group.7 2.218569e-05
8  2.218569e-05   group.8 2.835000e-05
9  2.835000e-05   group.9 4.785000e-05
10 4.785000e-05  group.10 1.476696e-04
```

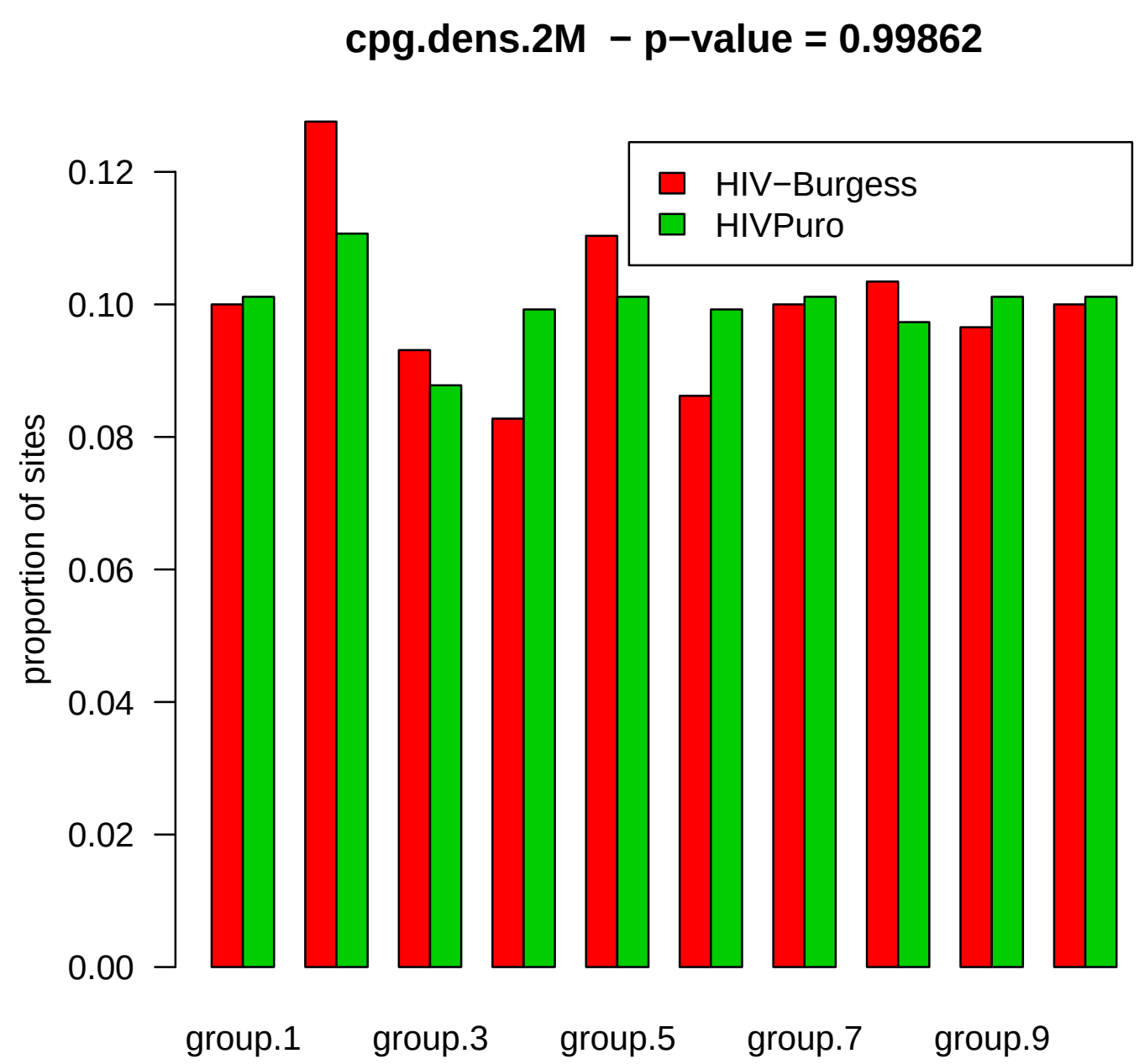

4.8 4 megaBase Window

In the barplot that follows we examine the association of insertion sites with expression density in a 4 megabase window surrounding each locus. First, we count just the number of genes on the represented on the chip.

```
 Category limits

          lower category        upper
1  3.571429e-08  group.1 1.822798e-06
2  1.822798e-06  group.2 2.557500e-06
3  2.557500e-06  group.3 3.279167e-06
4  3.279167e-06  group.4 4.209524e-06
5  4.209524e-06  group.5 5.728125e-06
6  5.728125e-06  group.6 7.947841e-06
7  7.947841e-06  group.7 1.042583e-05
8  1.042583e-05  group.8 1.331500e-05
9  1.331500e-05  group.9 1.725521e-05
10 1.725521e-05 group.10 3.462917e-05
```

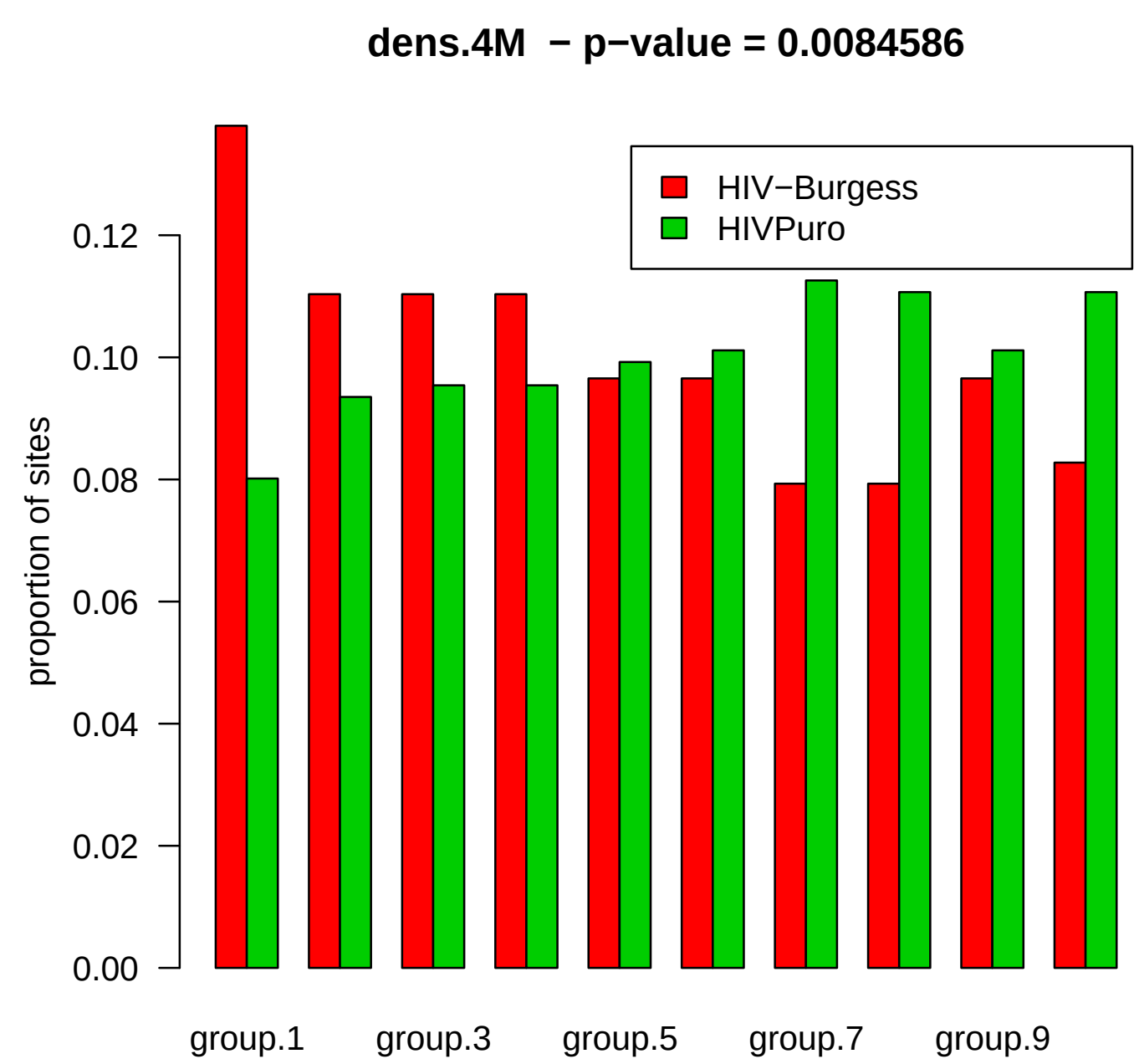

Here are the results for expression density. First, we count just genes that are in the upper half.

```
 Category limits

          lower category        upper
1  0.000000e+00  group.1 7.720833e-07
2  7.720833e-07  group.2 1.097262e-06
3  1.097262e-06  group.3 1.474226e-06
4  1.474226e-06  group.4 1.910256e-06
5  1.910256e-06  group.5 2.598810e-06
6  2.598810e-06  group.6 3.825833e-06
7  3.825833e-06  group.7 5.262500e-06
8  5.262500e-06  group.8 6.631667e-06
9  6.631667e-06  group.9 8.235681e-06
10 8.235681e-06 group.10 1.792917e-05
```

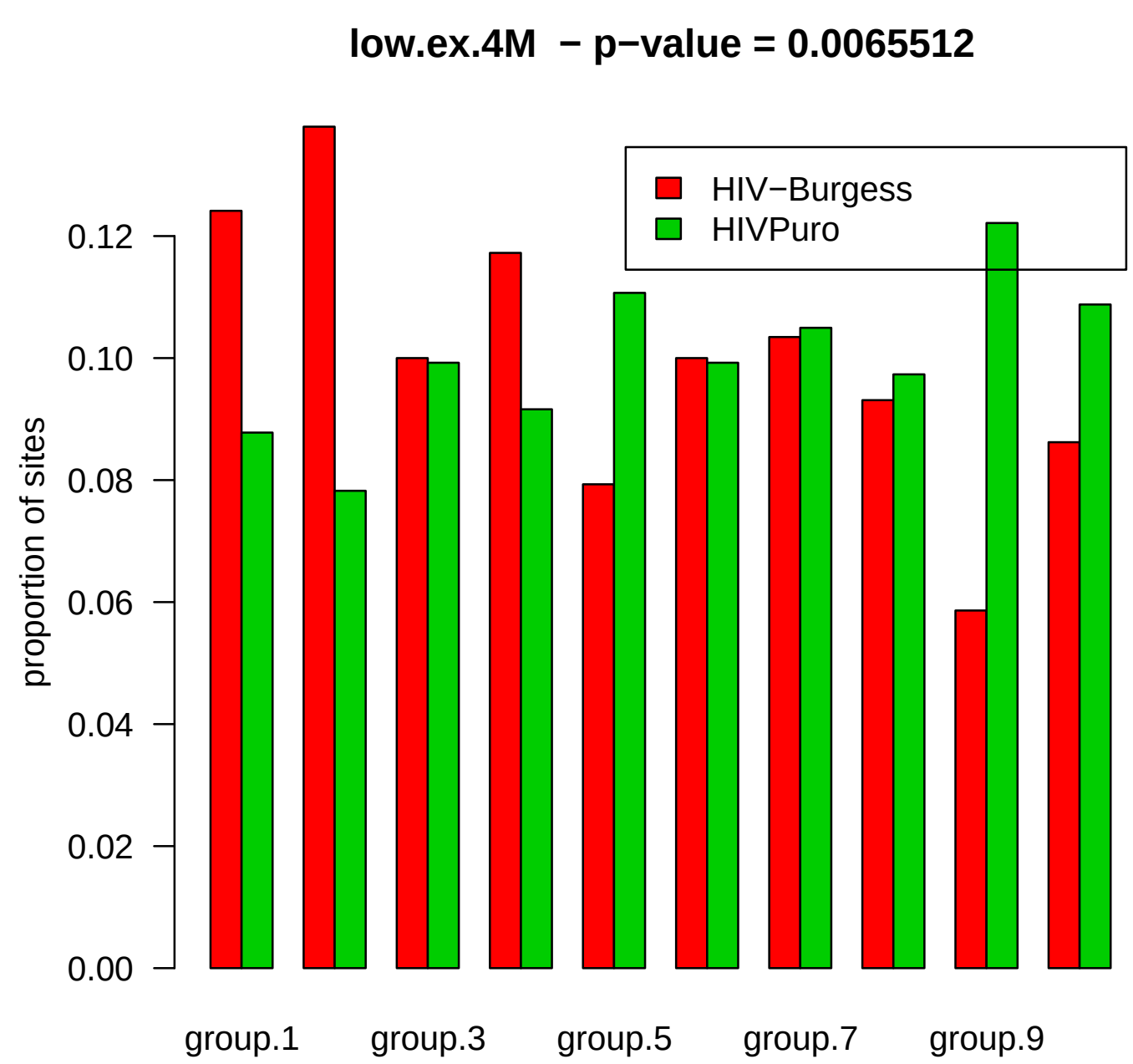

Now we count genes in the upper $1/8^{th}$:

```
Category limits

          lower  category         upper
1  0.000000e+00   group.1 2.562500e-07
2  2.562500e-07   group.2 5.416667e-07
3  5.416667e-07   group.3 7.291667e-07
4  7.291667e-07   group.4 9.782143e-07
5  9.782143e-07   group.5 1.330684e-06
6  1.330684e-06   group.6 1.851667e-06
7  1.851667e-06   group.7 2.725417e-06
8  2.725417e-06   group.8 3.380682e-06
9  3.380682e-06   group.9 4.661907e-06
10 4.661907e-06  group.10 9.337500e-06
```

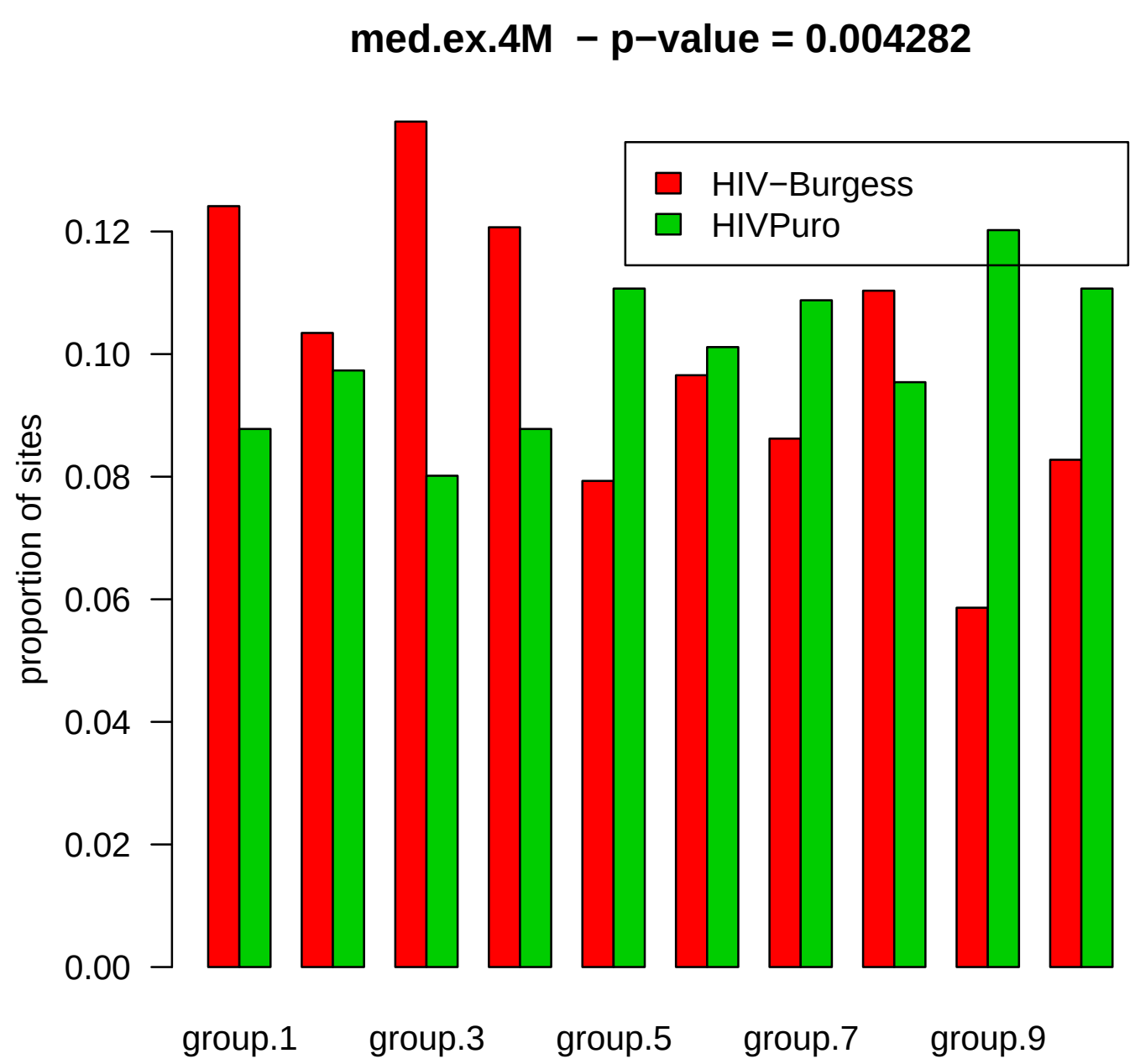

And here we count genes in the upper $1/16^{th}$:

```
Category limits

          lower category        upper
1  0.000000e+00  group.1 8.333333e-08
2  8.333333e-08  group.2 2.500000e-07
3  2.500000e-07  group.3 3.333333e-07
4  3.333333e-07  group.4 5.208333e-07
5  5.208333e-07  group.5 6.666667e-07
6  6.666667e-07  group.6 9.350000e-07
7  9.350000e-07  group.7 1.209722e-06
8  1.209722e-06  group.8 1.666667e-06
9  1.666667e-06  group.9 2.276667e-06
10 2.276667e-06 group.10 5.766667e-06
```

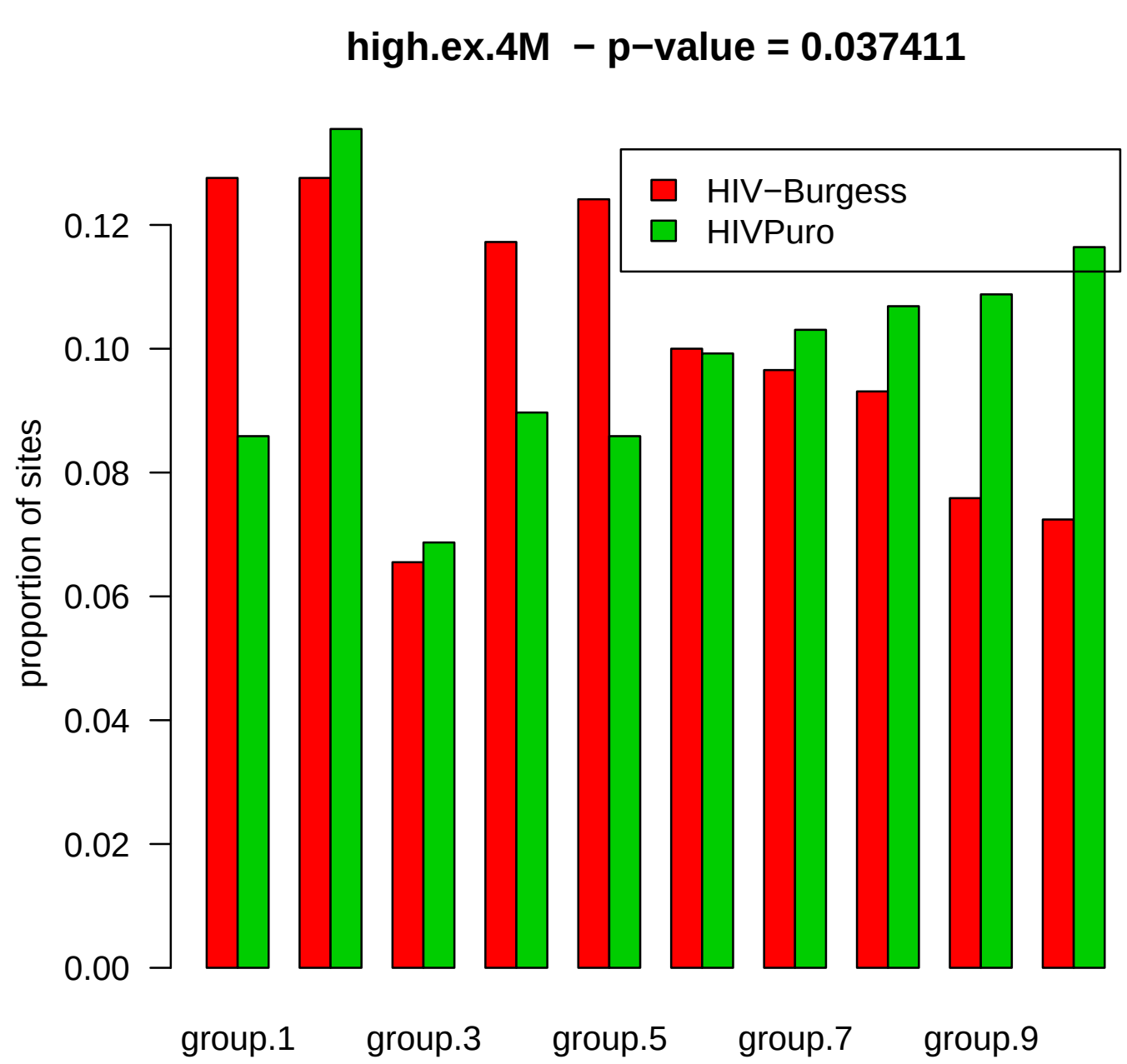

Here the effect of density of CpG islands is studied:

```
Category limits

           lower category        upper
1  5.000000e-07  group.1 3.250000e-06
2  3.250000e-06  group.2 4.250000e-06
3  4.250000e-06  group.3 5.862500e-06
4  5.862500e-06  group.4 7.650000e-06
5  7.650000e-06  group.5 1.156250e-05
6  1.156250e-05  group.6 1.600000e-05
7  1.600000e-05  group.7 2.100611e-05
8  2.100611e-05  group.8 2.517500e-05
9  2.517500e-05  group.9 4.175000e-05
10 4.175000e-05 group.10 1.235785e-04
```

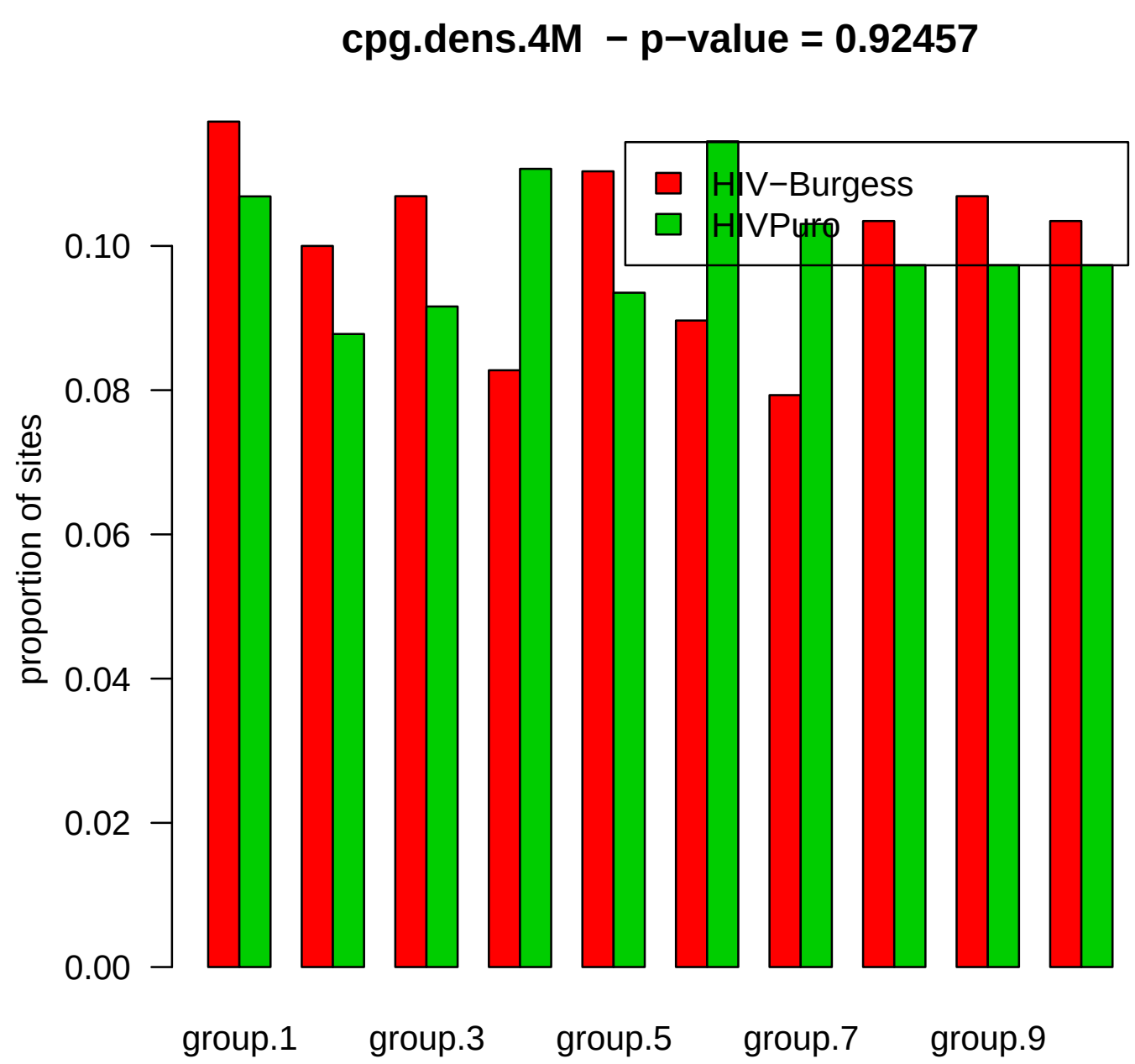

4.9 8 megaBase Window

In the barplot that follows we examine the association of insertion sites with expression density in a 8 megabase window surrounding each locus. First, we count just the number of genes on the represented on the chip.

```
 Category limits

          lower category        upper
1  2.083333e-07  group.1 1.906598e-06
2  1.906598e-06  group.2 2.470119e-06
3  2.470119e-06  group.3 3.193542e-06
4  3.193542e-06  group.4 4.052008e-06
5  4.052008e-06  group.5 5.521181e-06
6  5.521181e-06  group.6 7.063116e-06
7  7.063116e-06  group.7 8.620625e-06
8  8.620625e-06  group.8 1.139873e-05
9  1.139873e-05  group.9 1.546811e-05
10 1.546811e-05 group.10 2.956875e-05
```

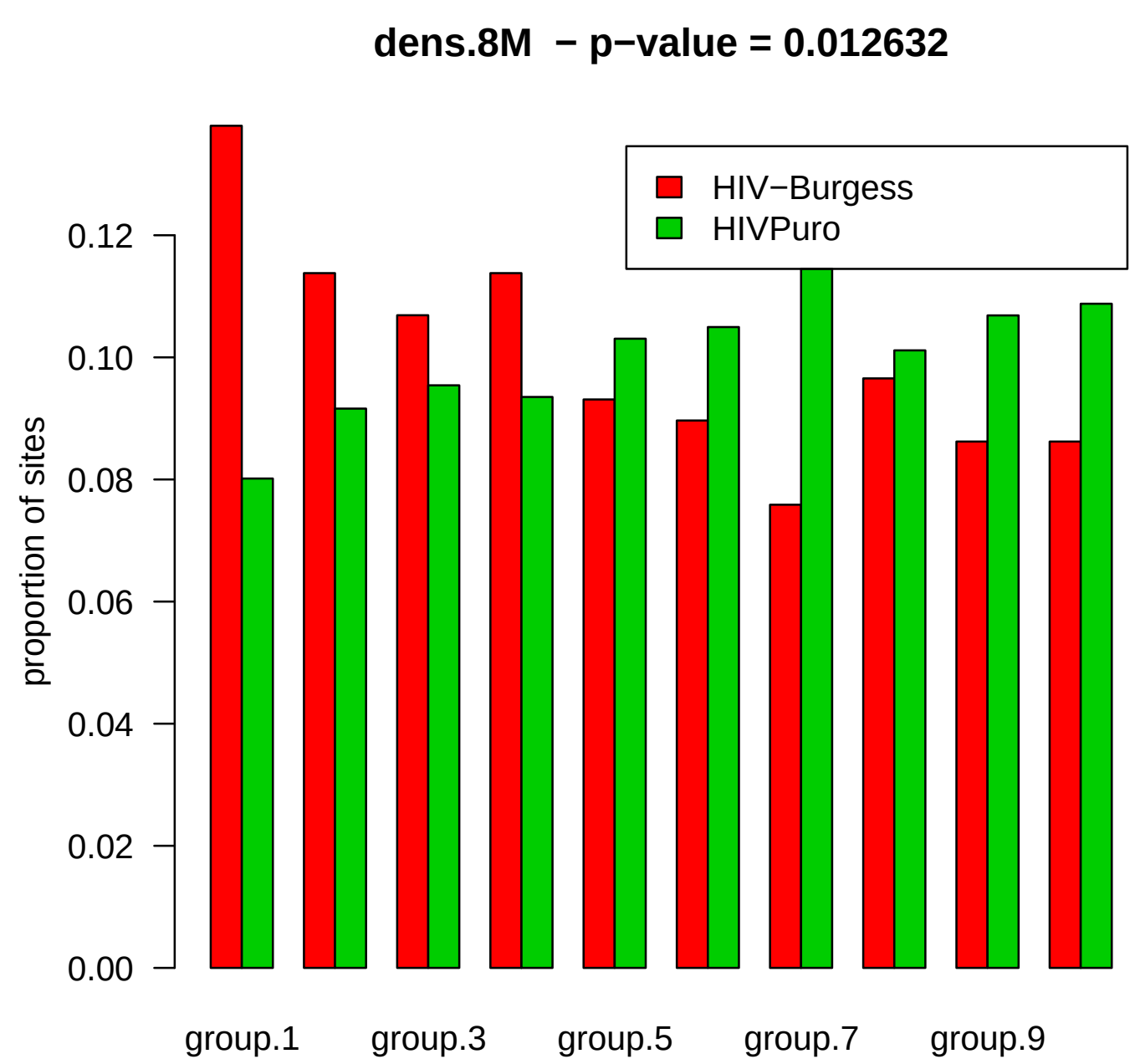

Here are the results for expression density. First, we count just genes that are in the upper half.

```
Category limits

          lower category        upper
1  0.000000e+00  group.1 7.374420e-07
2  7.374420e-07  group.2 1.060417e-06
3  1.060417e-06  group.3 1.385578e-06
4  1.385578e-06  group.4 1.766667e-06
5  1.766667e-06  group.5 2.516667e-06
6  2.516667e-06  group.6 3.314523e-06
7  3.314523e-06  group.7 4.458681e-06
8  4.458681e-06  group.8 5.663284e-06
9  5.663284e-06  group.9 7.561637e-06
10 7.561637e-06 group.10 1.221042e-05
```

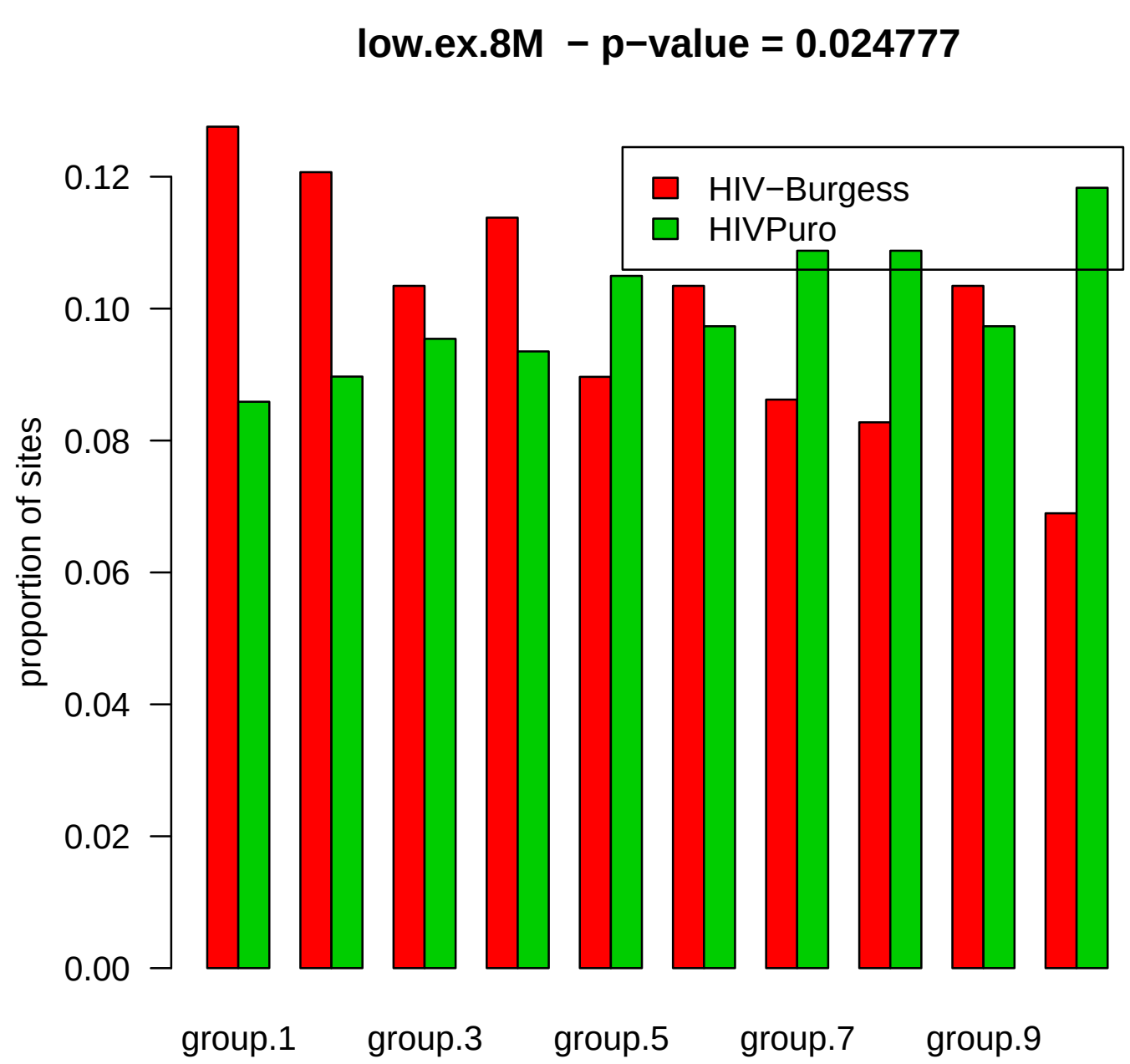

Now we count genes in the upper $1/8^{th}$:

```
Category limits

          lower  category        upper
1  0.000000e+00   group.1 3.378125e-07
2  3.378125e-07   group.2 5.256250e-07
3  5.256250e-07   group.3 6.916667e-07
4  6.916667e-07   group.4 8.945734e-07
5  8.945734e-07   group.5 1.267708e-06
6  1.267708e-06   group.6 1.709524e-06
7  1.709524e-06   group.7 2.210625e-06
8  2.210625e-06   group.8 3.099444e-06
9  3.099444e-06   group.9 3.880208e-06
10 3.880208e-06  group.10 6.927083e-06
```

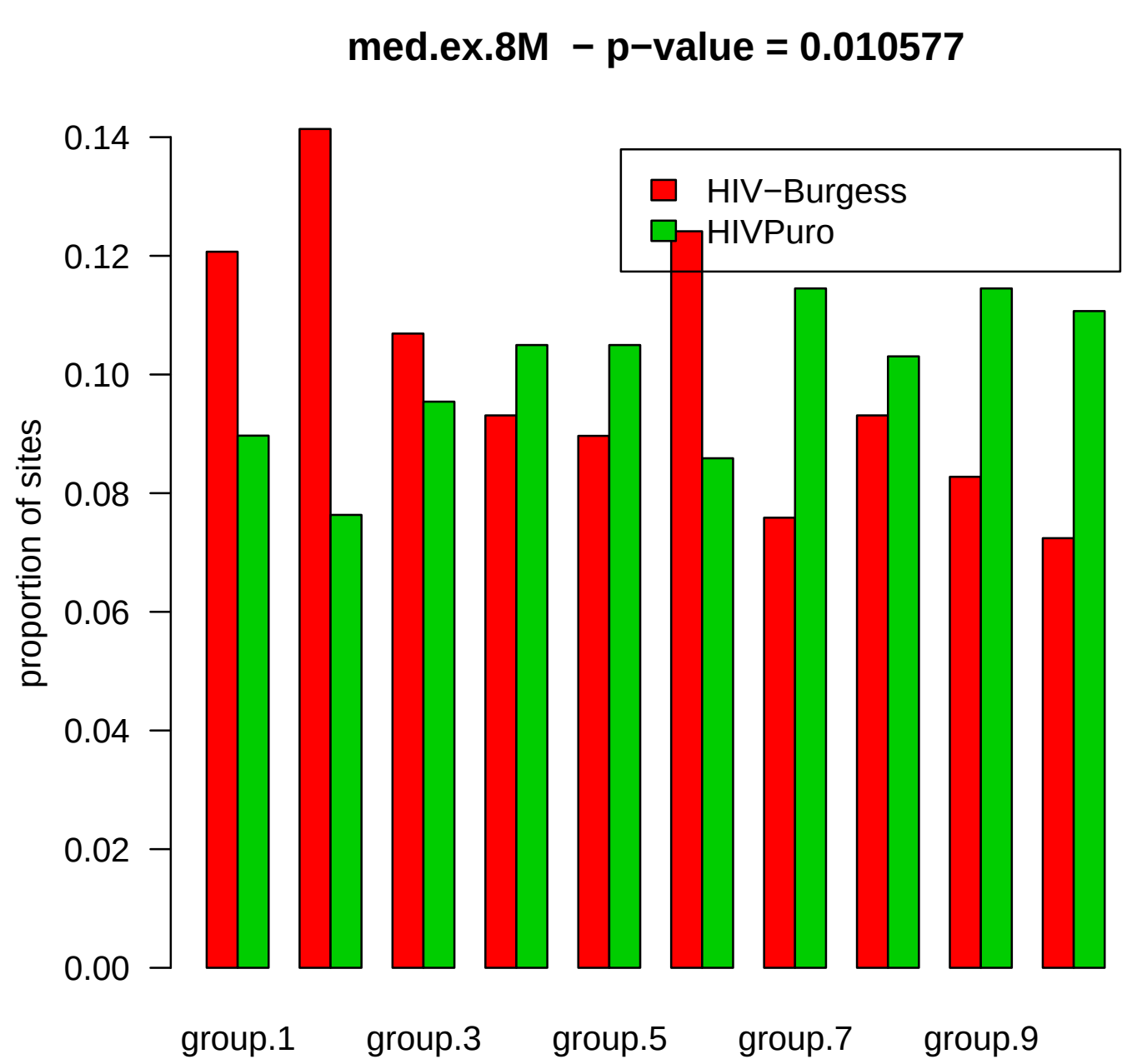

And here we count genes in the upper $1/16^{th}$:

```
Category limits

          lower category        upper
1  0.000000e+00  group.1 1.562500e-07
2  1.562500e-07  group.2 2.500000e-07
3  2.500000e-07  group.3 3.330476e-07
4  3.330476e-07  group.4 4.583333e-07
5  4.583333e-07  group.5 6.568452e-07
6  6.568452e-07  group.6 8.729167e-07
7  8.729167e-07  group.7 1.062500e-06
8  1.062500e-06  group.8 1.428467e-06
9  1.428467e-06  group.9 1.935417e-06
10 1.935417e-06 group.10 4.458333e-06
```

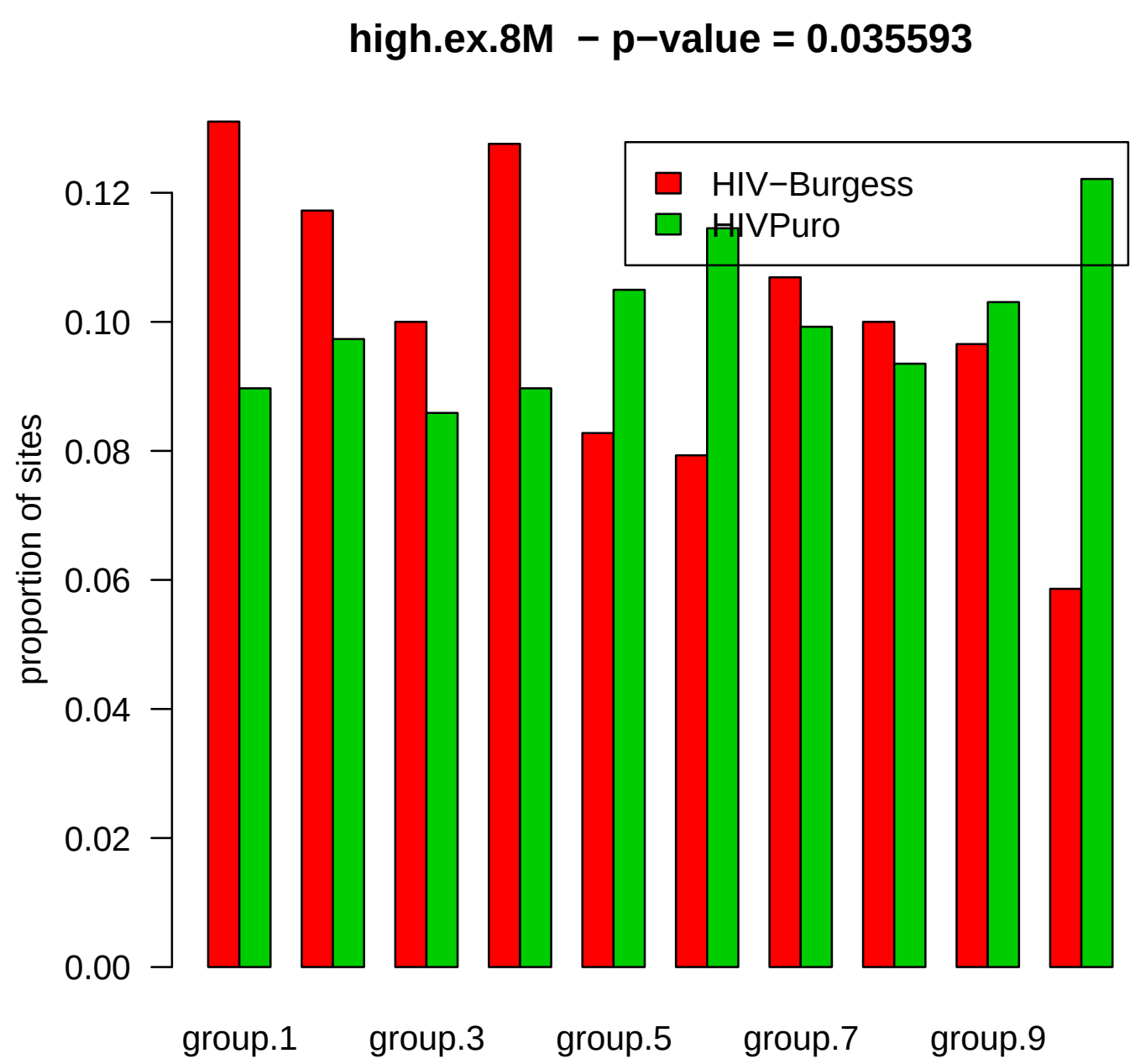

Here the effect of density of CpG islands is studied:

```
Category limits

        lower  category       upper
1  7.500000e-07   group.1 3.562500e-06
2  3.562500e-06   group.2 4.487279e-06
3  4.487279e-06   group.3 5.750000e-06
4  5.750000e-06   group.4 7.839015e-06
5  7.839015e-06   group.5 1.037500e-05
6  1.037500e-05   group.6 1.344793e-05
7  1.344793e-05   group.7 1.787500e-05
8  1.787500e-05   group.8 2.311342e-05
9  2.311342e-05   group.9 3.458812e-05
10 3.458812e-05  group.10 9.242333e-05
```

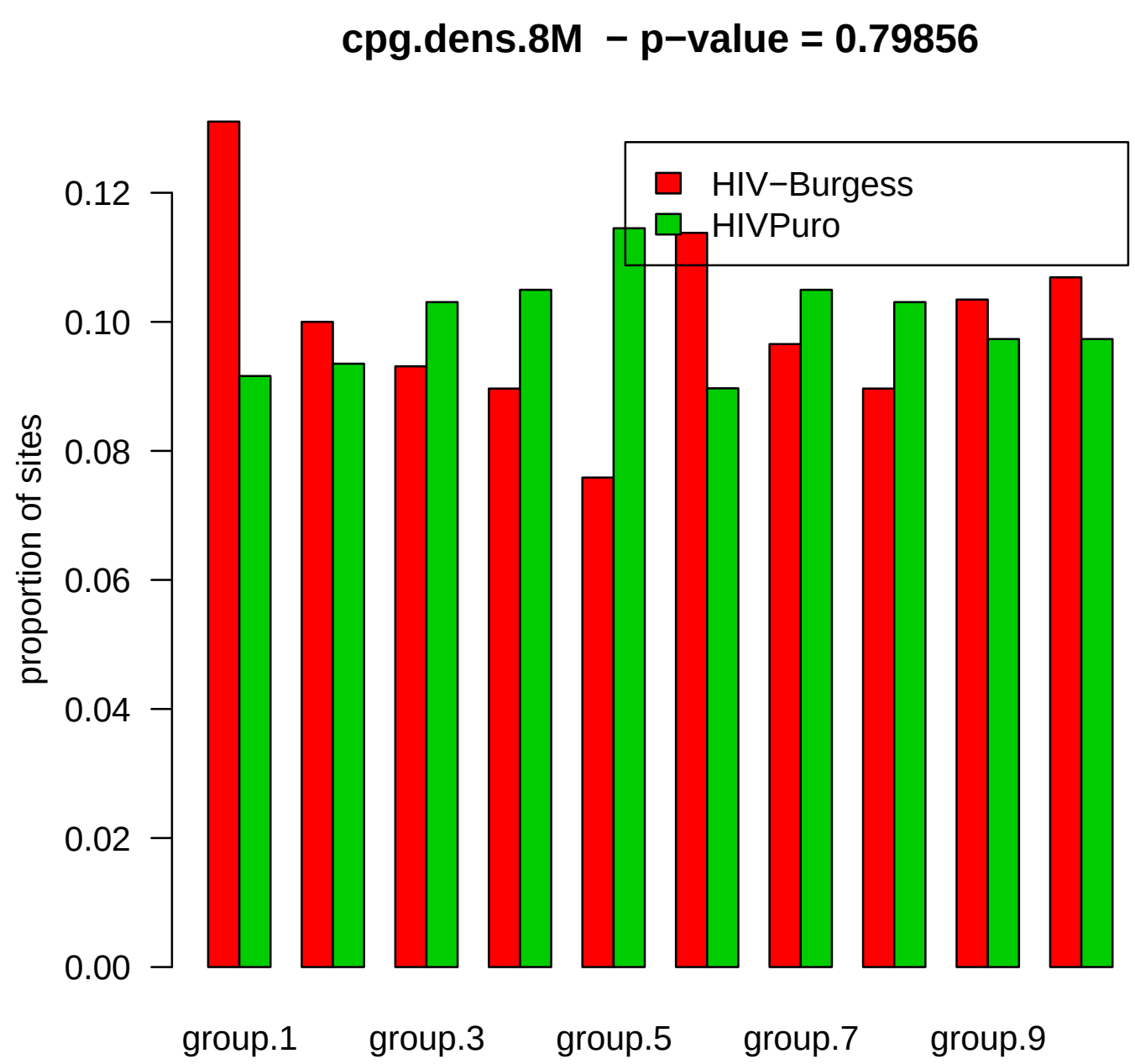

4.10 16 megaBase Window

In the barplot that follows we examine the association of insertion sites with expression density in a 16 megabase window surrounding each locus. First, we count just the number of genes on the represented on the chip.

```
 Category limits

          lower category        upper
1  6.166667e-07  group.1 1.858780e-06
2  1.858780e-06  group.2 2.474033e-06
3  2.474033e-06  group.3 3.130491e-06
4  3.130491e-06  group.4 3.900208e-06
5  3.900208e-06  group.5 5.193123e-06
6  5.193123e-06  group.6 6.079664e-06
7  6.079664e-06  group.7 7.680484e-06
8  7.680484e-06  group.8 9.670616e-06
9  9.670616e-06  group.9 1.312768e-05
10 1.312768e-05 group.10 1.928512e-05
```

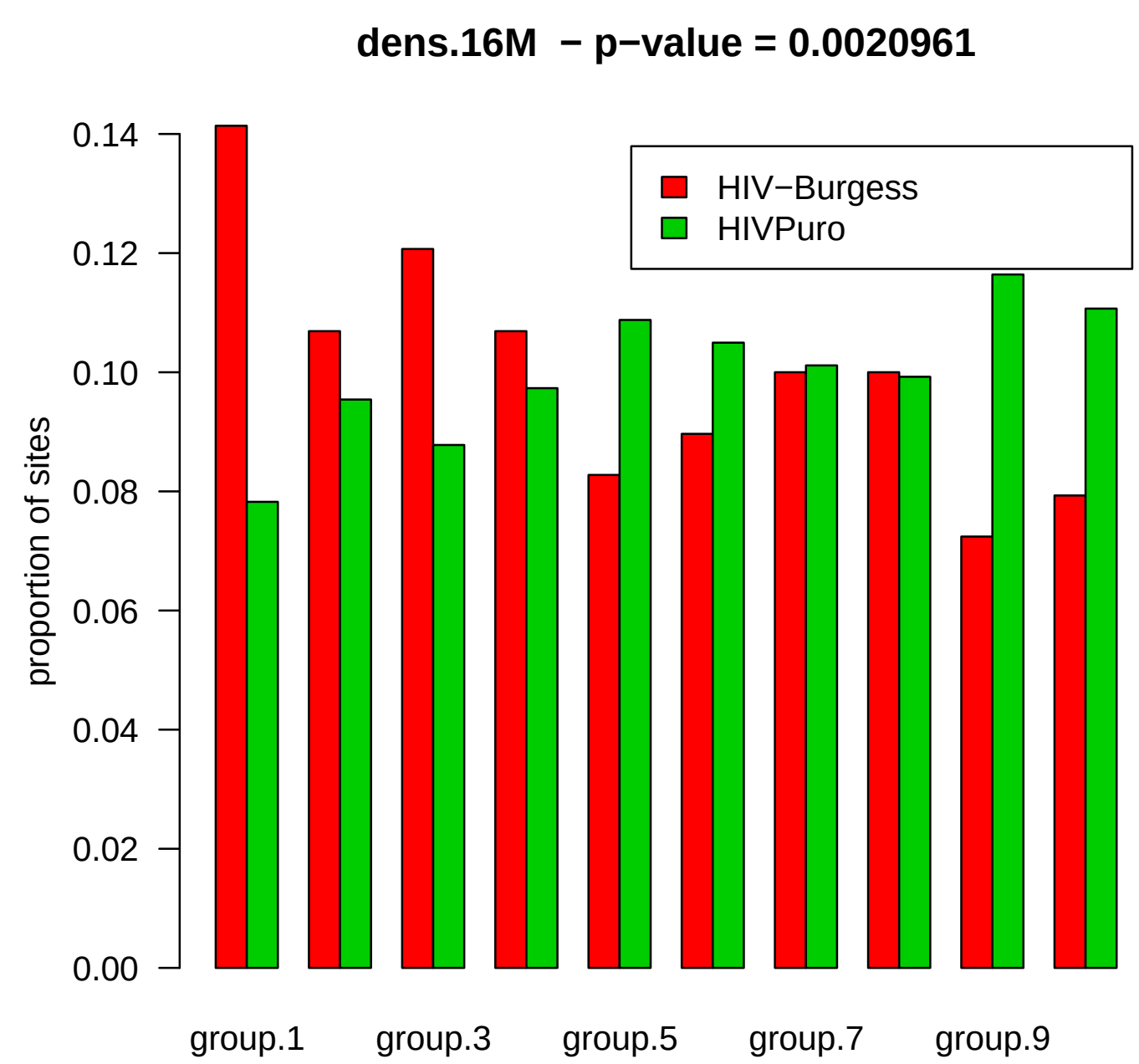

Here are the results for expression density. First, we count just genes that are in the upper half.

Category limits

	lower	category	upper
1	1.919643e-07	group.1	7.821875e-07
2	7.821875e-07	group.2	1.094980e-06
3	1.094980e-06	group.3	1.303125e-06
4	1.303125e-06	group.4	1.748333e-06
5	1.748333e-06	group.5	2.397900e-06
6	2.397900e-06	group.6	2.838750e-06
7	2.838750e-06	group.7	3.606250e-06
8	3.606250e-06	group.8	5.100166e-06
9	5.100166e-06	group.9	6.354021e-06
10	6.354021e-06	group.10	9.098263e-06

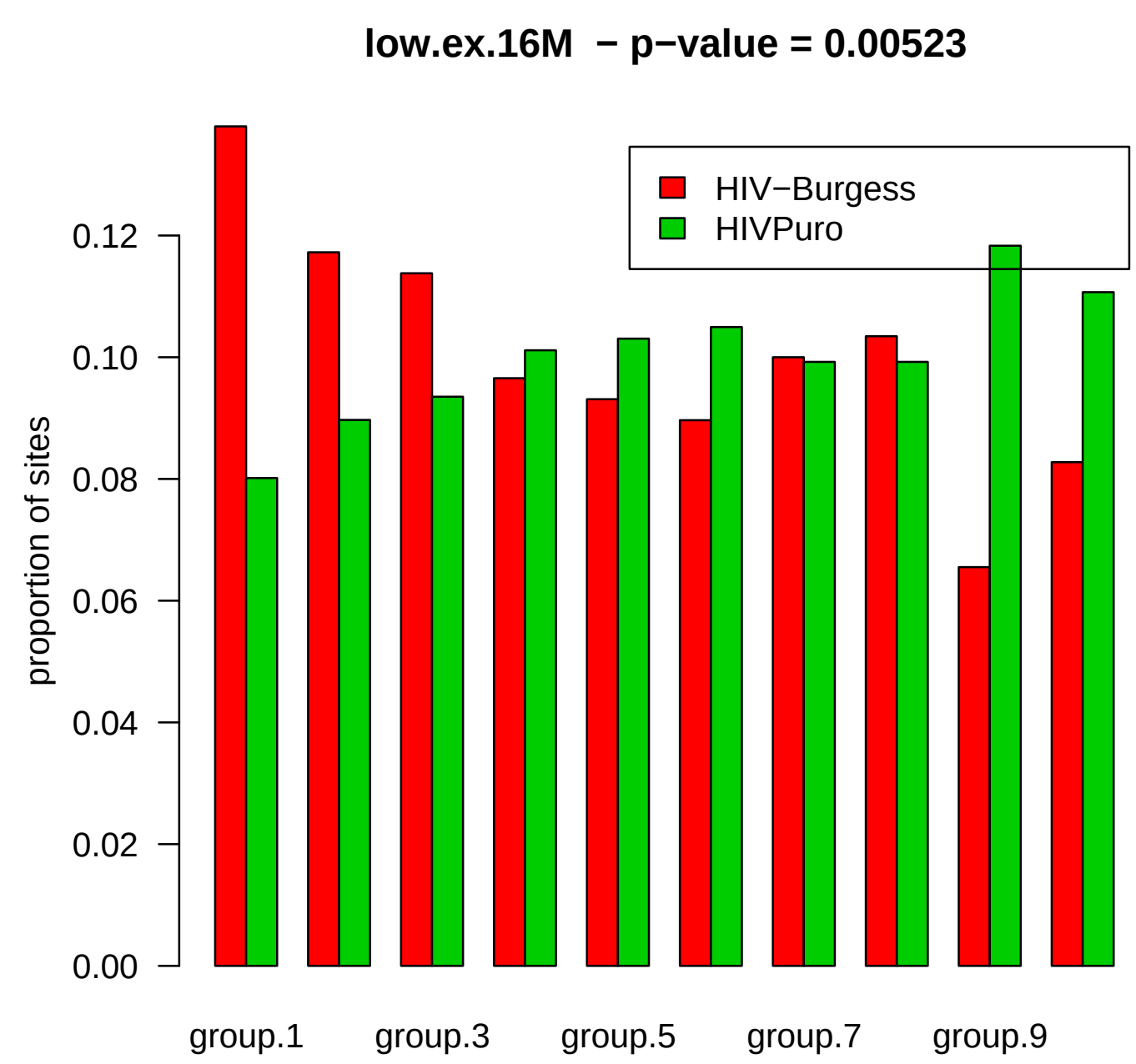

Now we count genes in the upper $1/8^{th}$:

```
Category limits

          lower  category        upper
1  6.250000e-08   group.1 3.794792e-07
2  3.794792e-07   group.2 5.270833e-07
3  5.270833e-07   group.3 6.779167e-07
4  6.779167e-07   group.4 9.320536e-07
5  9.320536e-07   group.5 1.184896e-06
6  1.184896e-06   group.6 1.496875e-06
7  1.496875e-06   group.7 1.897917e-06
8  1.897917e-06   group.8 2.681056e-06
9  2.681056e-06   group.9 3.113679e-06
10 3.113679e-06  group.10 4.852258e-06
```

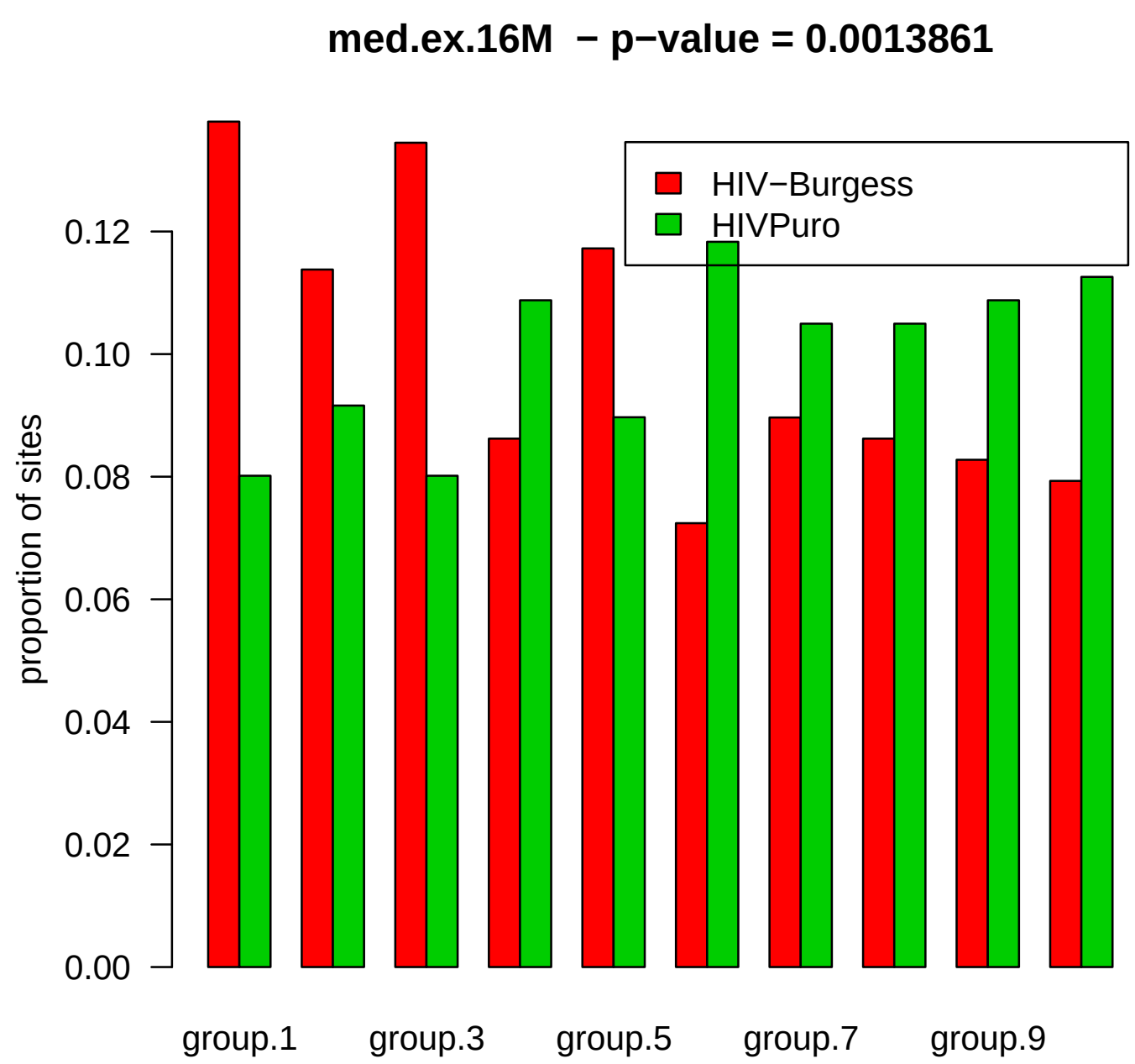

And here we count genes in the upper $1/16^{th}$:

```
Category limits

          lower category        upper
1  0.000000e+00  group.1 1.875000e-07
2  1.875000e-07  group.2 2.604167e-07
3  2.604167e-07  group.3 3.505878e-07
4  3.505878e-07  group.4 4.843750e-07
5  4.843750e-07  group.5 6.093750e-07
6  6.093750e-07  group.6 7.706250e-07
7  7.706250e-07  group.7 9.528362e-07
8  9.528362e-07  group.8 1.108333e-06
9  1.108333e-06  group.9 1.630218e-06
10 1.630218e-06 group.10 2.718750e-06
```

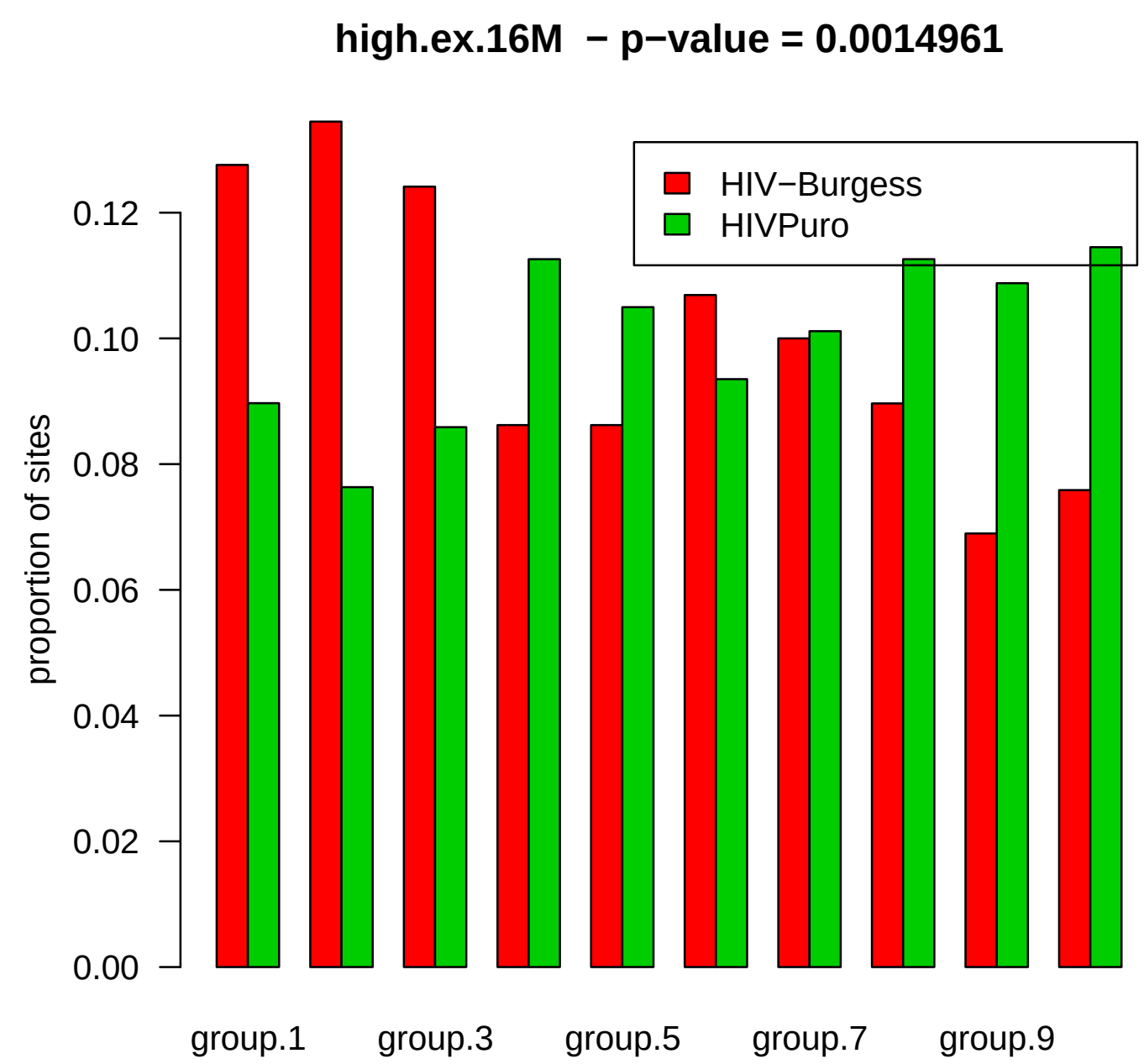

Here the effect of density of CpG islands is studied:

```
Category limits

          lower category        upper
1  1.343750e-06  group.1 3.884375e-06
2  3.884375e-06  group.2 4.656250e-06
3  4.656250e-06  group.3 6.031250e-06
4  6.031250e-06  group.4 7.805925e-06
5  7.805925e-06  group.5 9.625000e-06
6  9.625000e-06  group.6 1.121250e-05
7  1.121250e-05  group.7 1.606562e-05
8  1.606562e-05  group.8 1.916041e-05
9  1.916041e-05  group.9 2.473723e-05
10 2.473723e-05 group.10 6.694779e-05
```

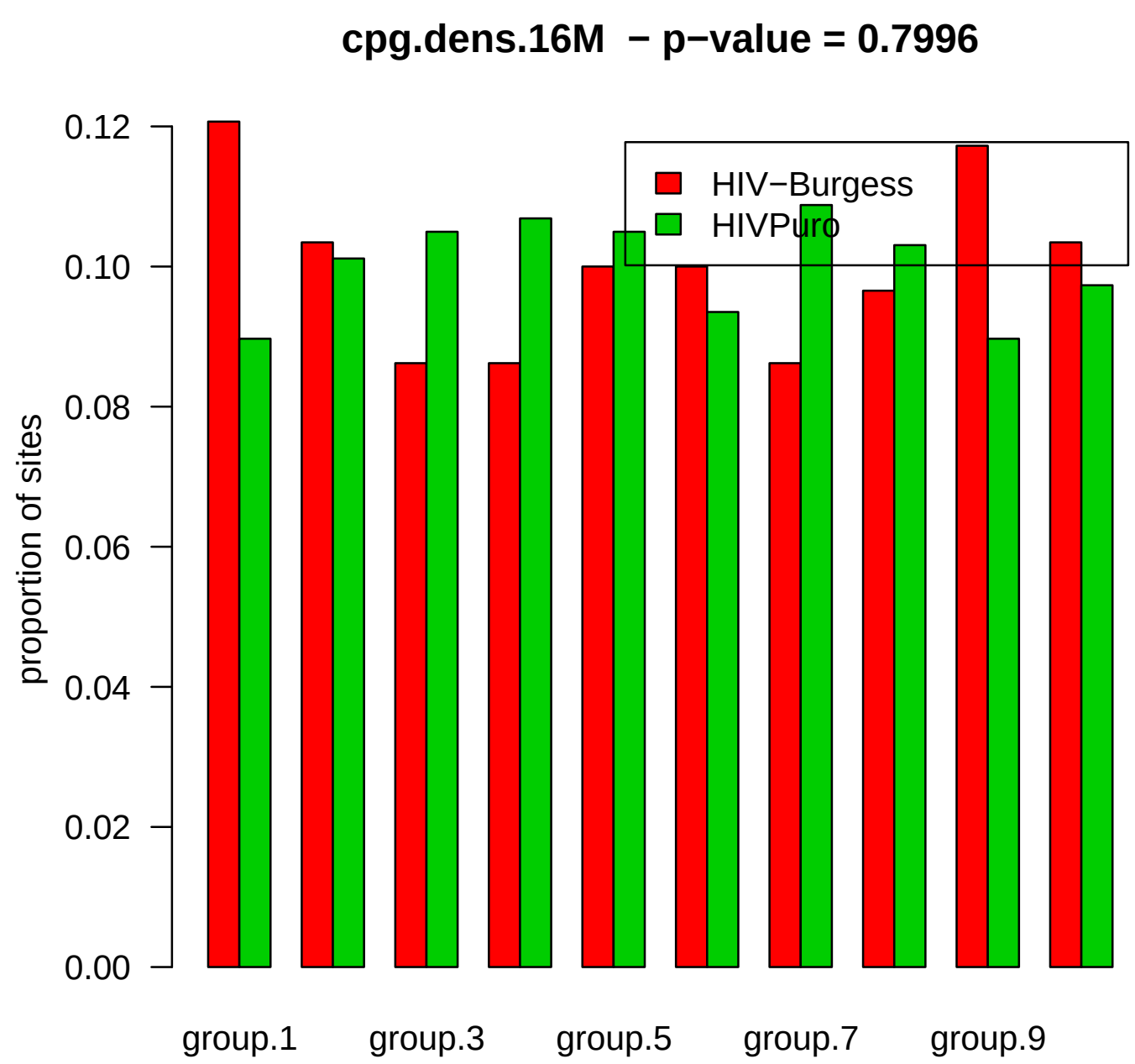

4.11 32 megaBase Window

In the barplot that follows we examine the association of insertion sites with expression density in a 32 megabase window surrounding each locus. First, we count just the number of genes on the represented on the chip.

```
 Category limits

          lower category        upper
1  5.753393e-07  group.1 2.036203e-06
2  2.036203e-06  group.2 2.534345e-06
3  2.534345e-06  group.3 3.160329e-06
4  3.160329e-06  group.4 3.977480e-06
5  3.977480e-06  group.5 4.703423e-06
6  4.703423e-06  group.6 5.361487e-06
7  5.361487e-06  group.7 6.739752e-06
8  6.739752e-06  group.8 8.247105e-06
9  8.247105e-06  group.9 9.252270e-06
10 9.252270e-06 group.10 1.839120e-05
```

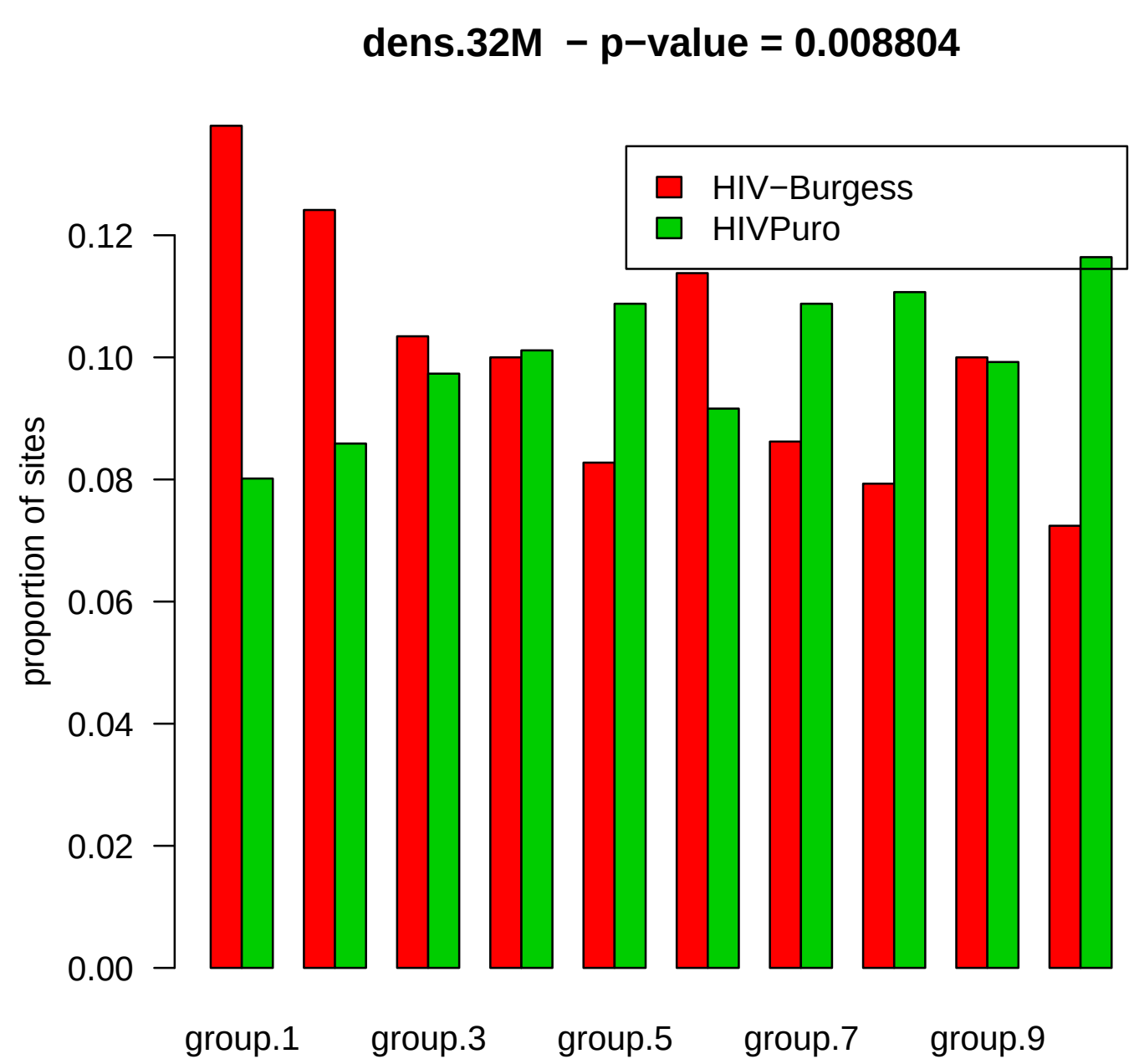

Here are the results for expression density. First, we count just genes that are in the upper half.

```
 Category limits

          lower category        upper
1  2.840978e-07  group.1 8.776843e-07
2  8.776843e-07  group.2 1.091334e-06
3  1.091334e-06  group.3 1.396384e-06
4  1.396384e-06  group.4 1.752302e-06
5  1.752302e-06  group.5 2.099380e-06
6  2.099380e-06  group.6 2.551565e-06
7  2.551565e-06  group.7 3.279120e-06
8  3.279120e-06  group.8 4.097512e-06
9  4.097512e-06  group.9 4.536546e-06
10 4.536546e-06 group.10 8.754914e-06
```

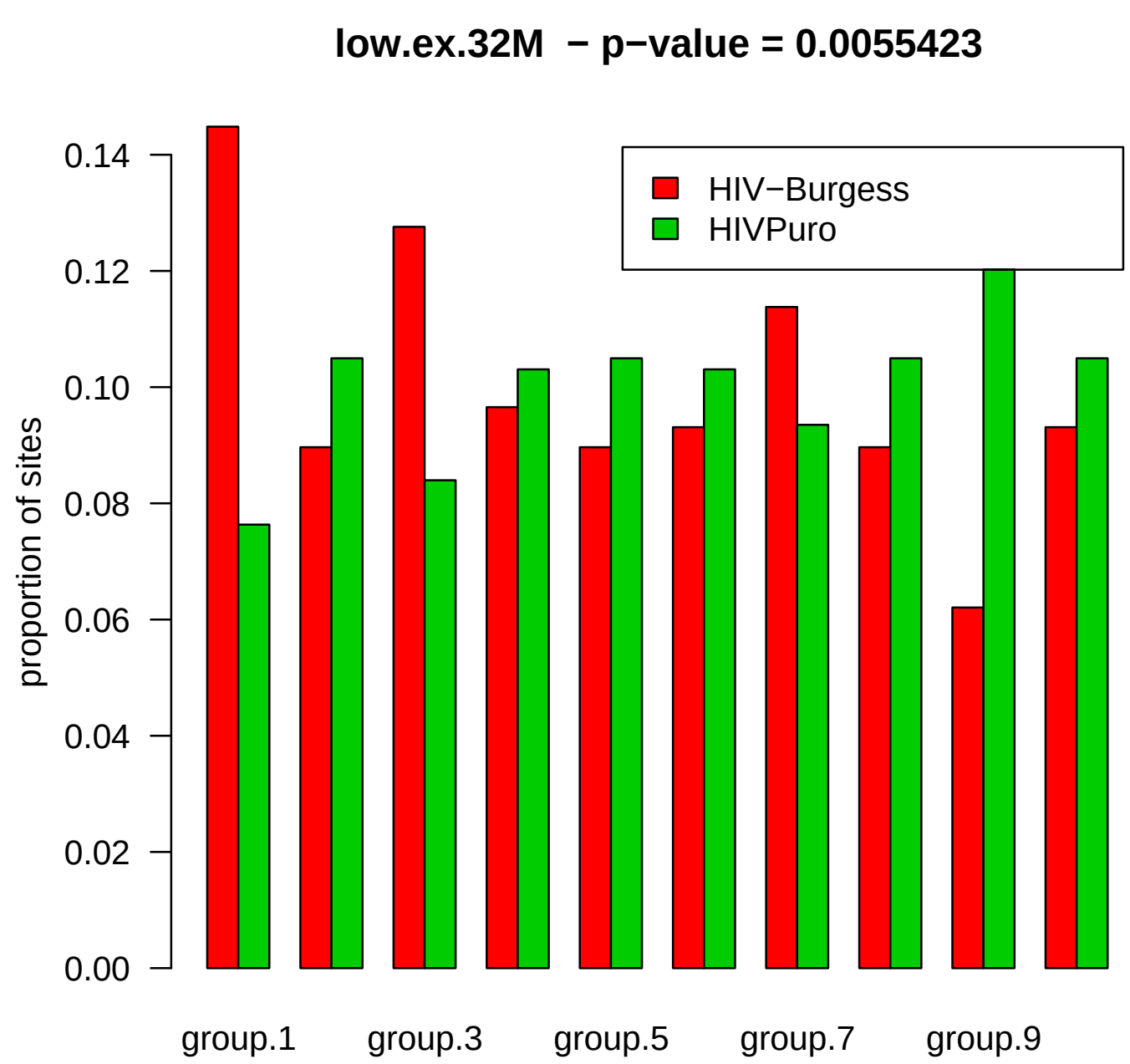

Now we count genes in the upper $1/8^{th}$:

```
Category limits

         lower  category        upper
1  9.050701e-08  group.1 4.401184e-07
2  4.401184e-07  group.2 5.687500e-07
3  5.687500e-07  group.3 7.312846e-07
4  7.312846e-07  group.4 9.068750e-07
5  9.068750e-07  group.5 1.088542e-06
6  1.088542e-06  group.6 1.276027e-06
7  1.276027e-06  group.7 1.764750e-06
8  1.764750e-06  group.8 2.014504e-06
9  2.014504e-06  group.9 2.438892e-06
10 2.438892e-06 group.10 4.505173e-06
```

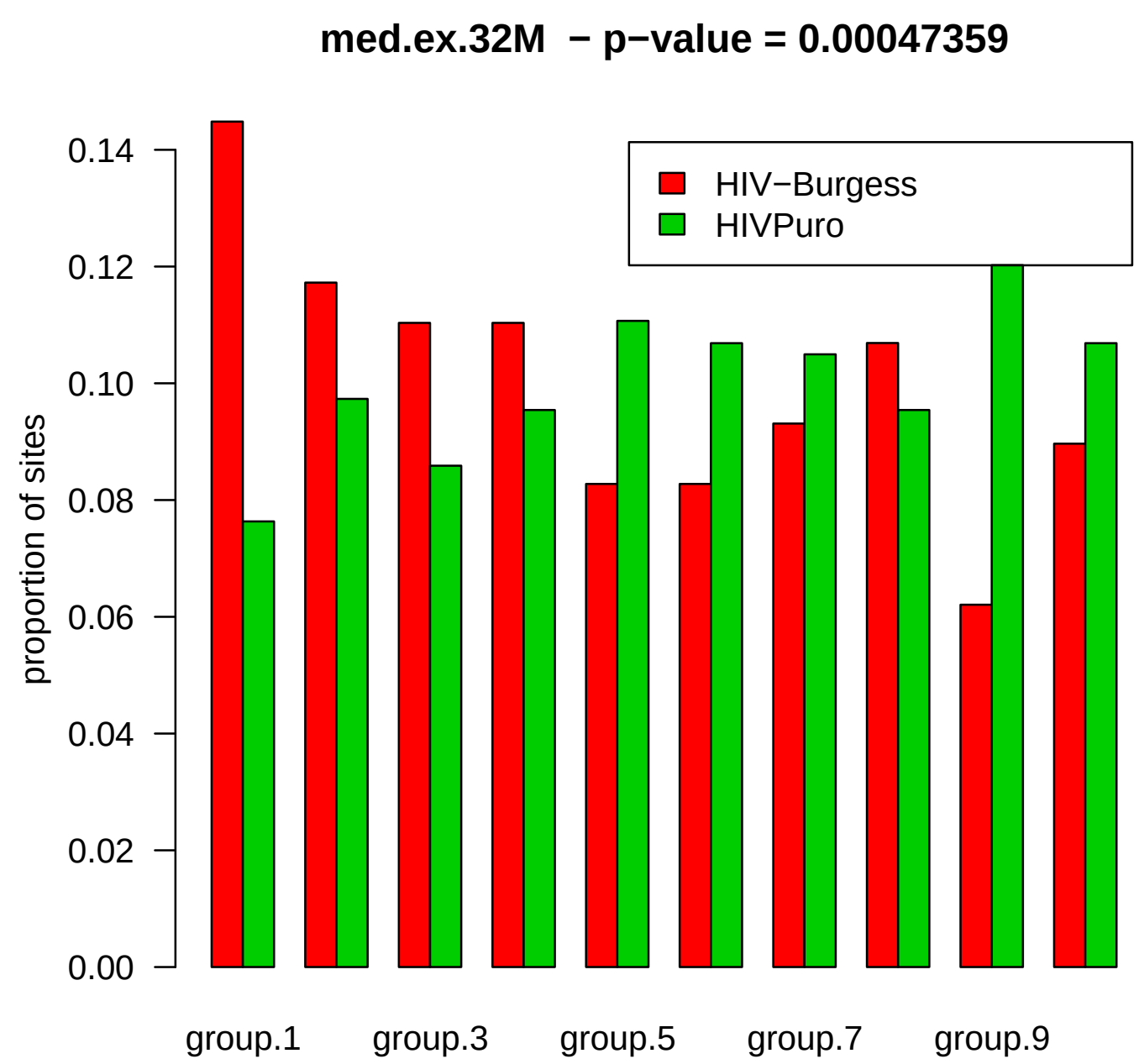

And here we count genes in the upper $1/16^{th}$:

```
Category limits

          lower  category        upper
1   3.016900e-08  group.1 2.124740e-07
2   2.124740e-07  group.2 2.992708e-07
3   2.992708e-07  group.3 3.632552e-07
4   3.632552e-07  group.4 4.630208e-07
5   4.630208e-07  group.5 5.781250e-07
6   5.781250e-07  group.6 7.018929e-07
7   7.018929e-07  group.7 8.080357e-07
8   8.080357e-07  group.8 9.372024e-07
9   9.372024e-07  group.9 1.207031e-06
10  1.207031e-06 group.10 2.171921e-06
```

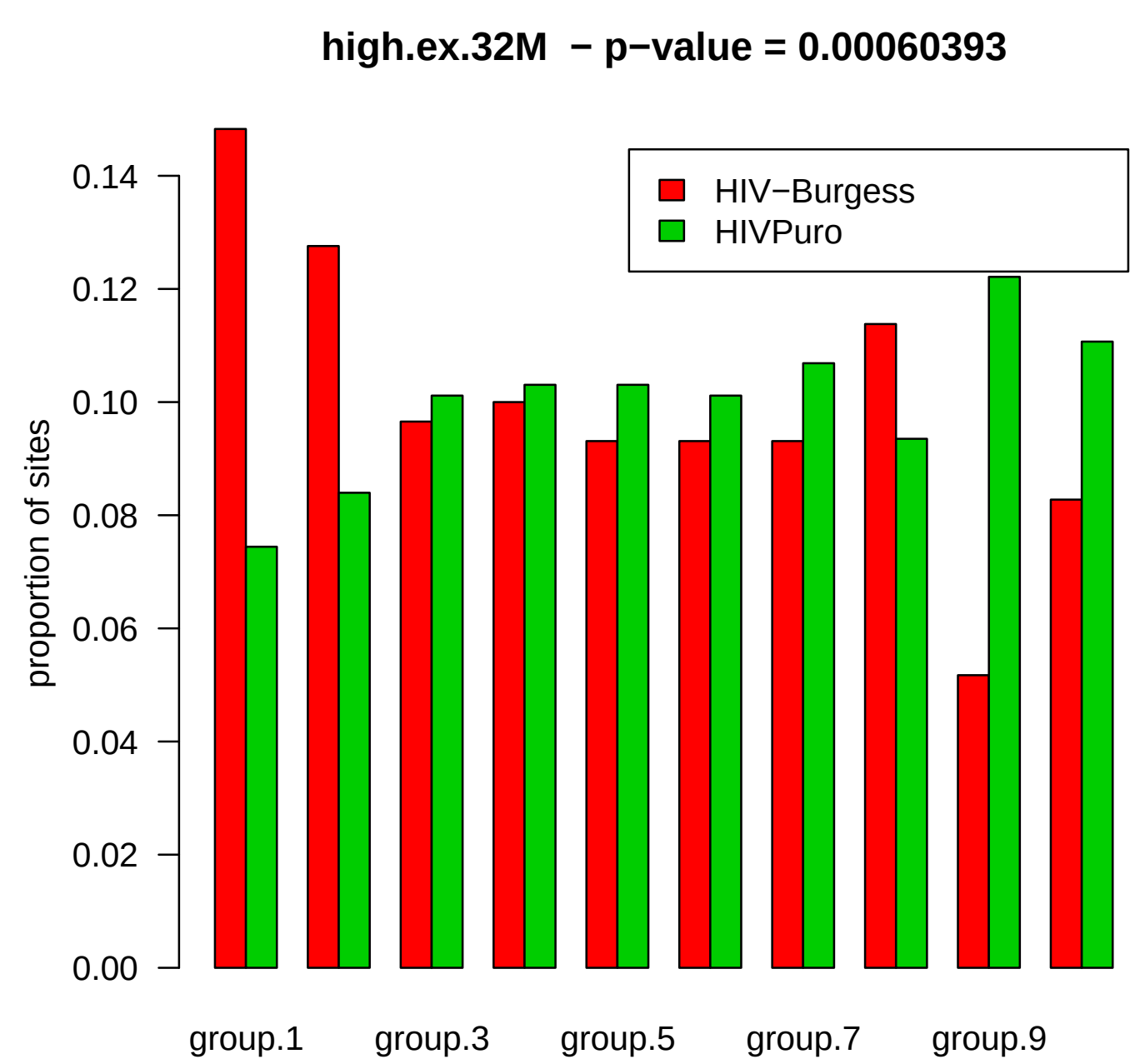

Here the effect of density of CpG islands is studied:

```
Category limits

          lower category        upper
1  2.734375e-06  group.1 4.433832e-06
2  4.433832e-06  group.2 5.643750e-06
3  5.643750e-06  group.3 6.611517e-06
4  6.611517e-06  group.4 7.296875e-06
5  7.296875e-06  group.5 8.469715e-06
6  8.469715e-06  group.6 1.043897e-05
7  1.043897e-05  group.7 1.246406e-05
8  1.246406e-05  group.8 1.665107e-05
9  1.665107e-05  group.9 2.010270e-05
10 2.010270e-05 group.10 4.036208e-05
```

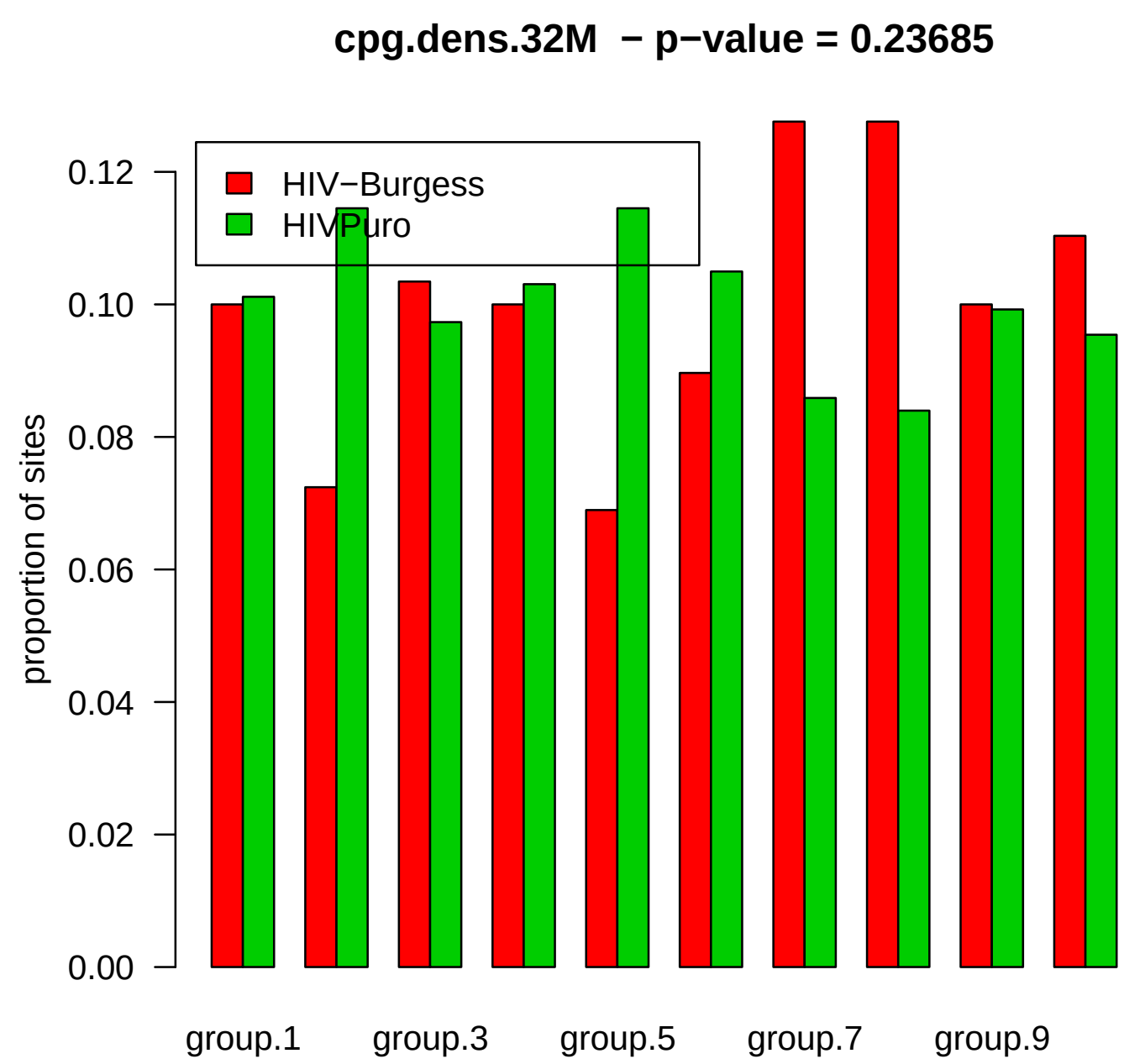

5 Juxtaposition with Gene Start and End Positions

5.1 Acembly Annotations

In this section we study the effect of juxtaposition in terms of gene start and end positions. The first barplot shows the effect of gene width for those insertions that are located within an Acembly gene.

```
 Category limits

      lower category    upper
1     319.0  group.1  14085.2
2   14085.2  group.2  23312.6
3   23312.6  group.3  34922.3
4   34922.3  group.4  46396.8
5   46396.8  group.5  61817.0
6   61817.0  group.6  77882.0
7   77882.0  group.7 105342.3
8  105342.3  group.8 142274.6
9  142274.6  group.9 204679.4
10 204679.4 group.10 777203.0
```

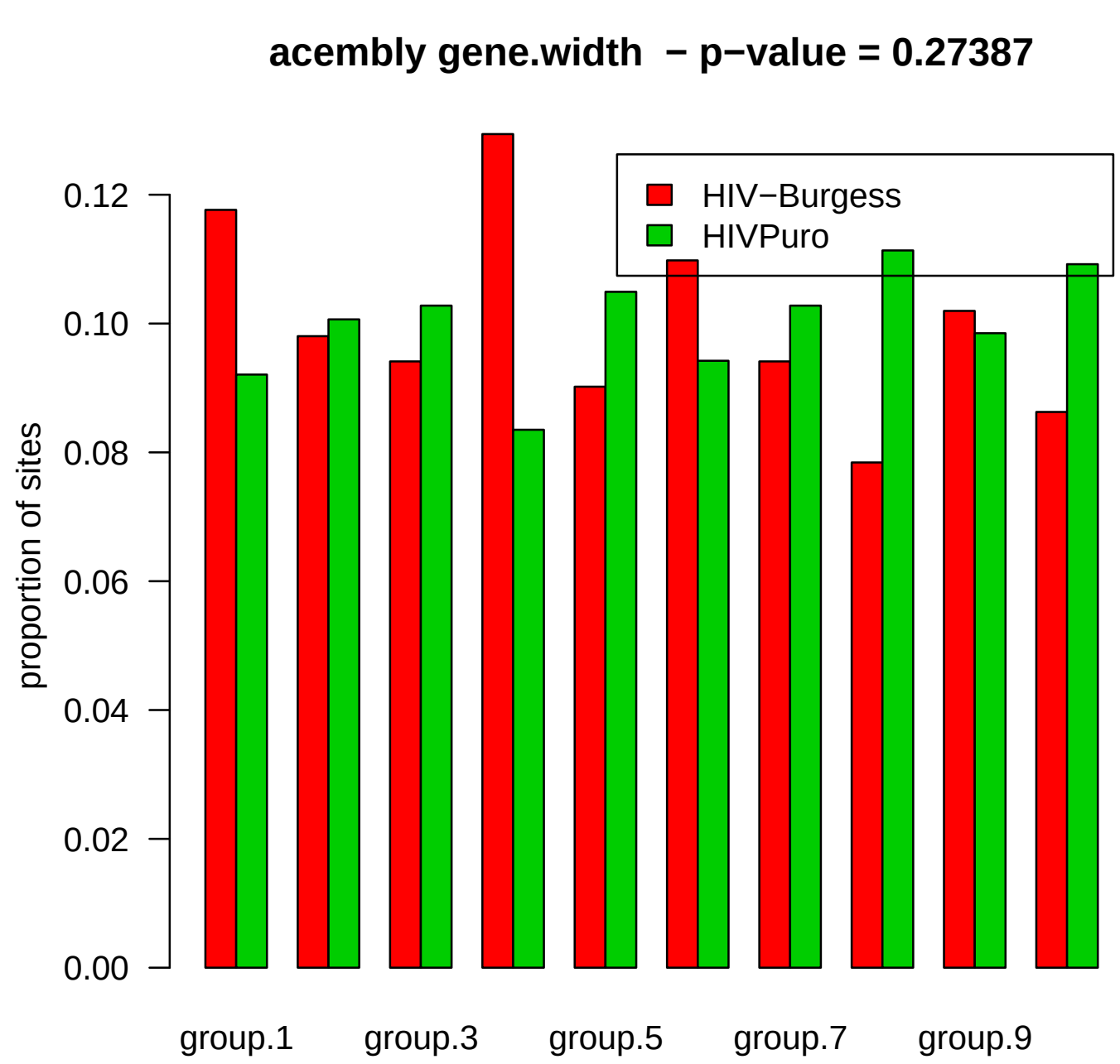

The next plot uses the width of a non-gene region for insertions that fall into such regions.

```
 Category limits

      lower category    upper
1    1831.0  group.1  10067.8
2   10067.8  group.2  18439.8
3   18439.8  group.3  28426.9
4   28426.9  group.4  55108.4
5   55108.4  group.5  69209.5
6   69209.5  group.6 100116.4
7  100116.4  group.7 161656.4
8  161656.4  group.8 204695.0
9  204695.0  group.9 304640.0
10 304640.0 group.10 765281.0
```

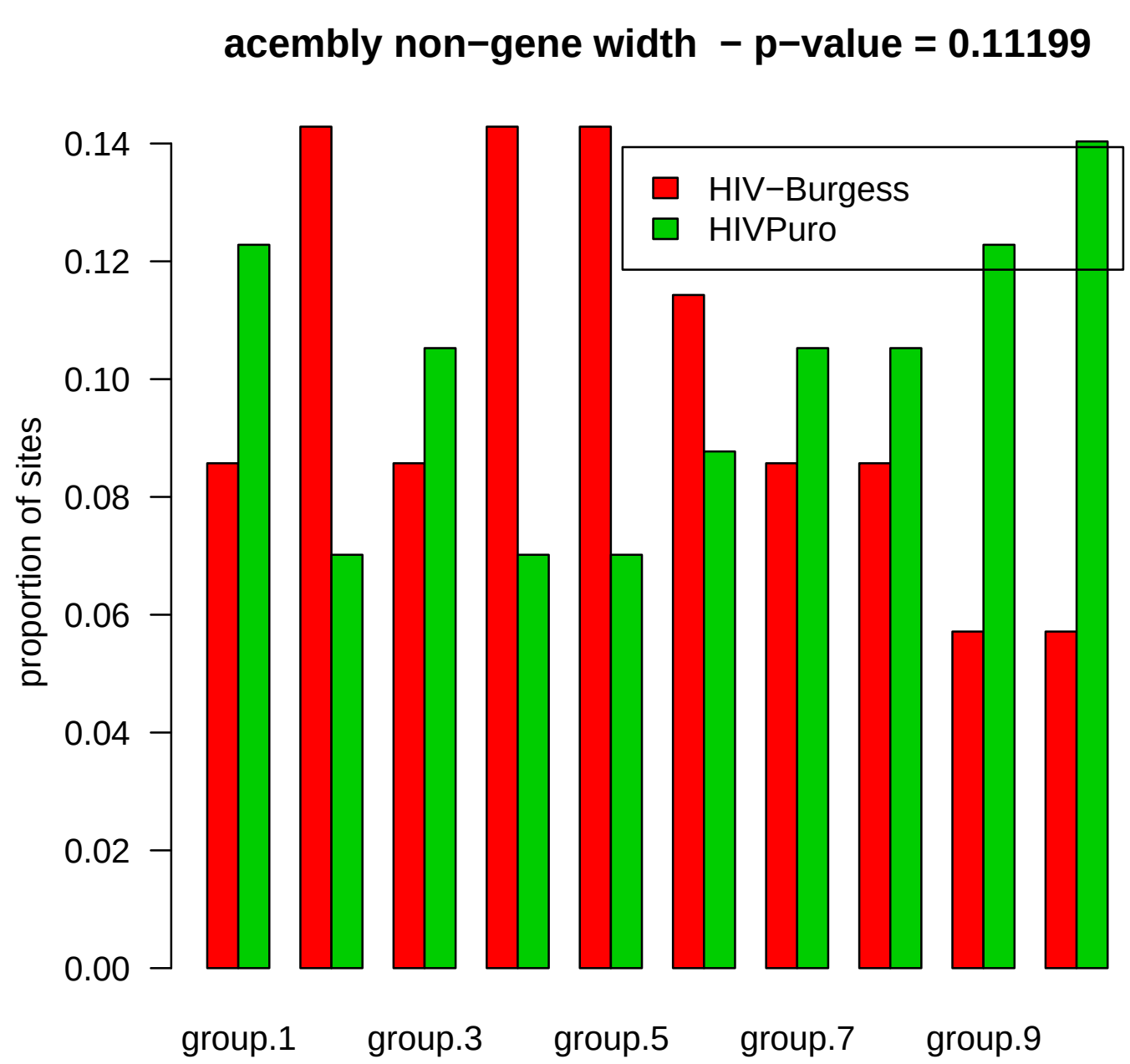

The next plot studies the distance to the nearest boundary between a gene and a non-gene region. The distance is expressed as a fraction of the length of the region. Thus, '0.25' refers to one quarter of the distance from the site to nearest boundary divided by the total width of the region.

```
 Category limits

          lower category      upper
1  0.0001694987  group.1 0.05353284
2  0.0535328379  group.2 0.10995390
3  0.1099538989  group.3 0.15953682
4  0.1595368165  group.4 0.21857310
5  0.2185731007  group.5 0.25746174
6  0.2574617365  group.6 0.31155298
7  0.3115529771  group.7 0.35748191
8  0.3574819092  group.8 0.40318291
9  0.4031829149  group.9 0.44503803
10 0.4450380305 group.10 0.49994218
```

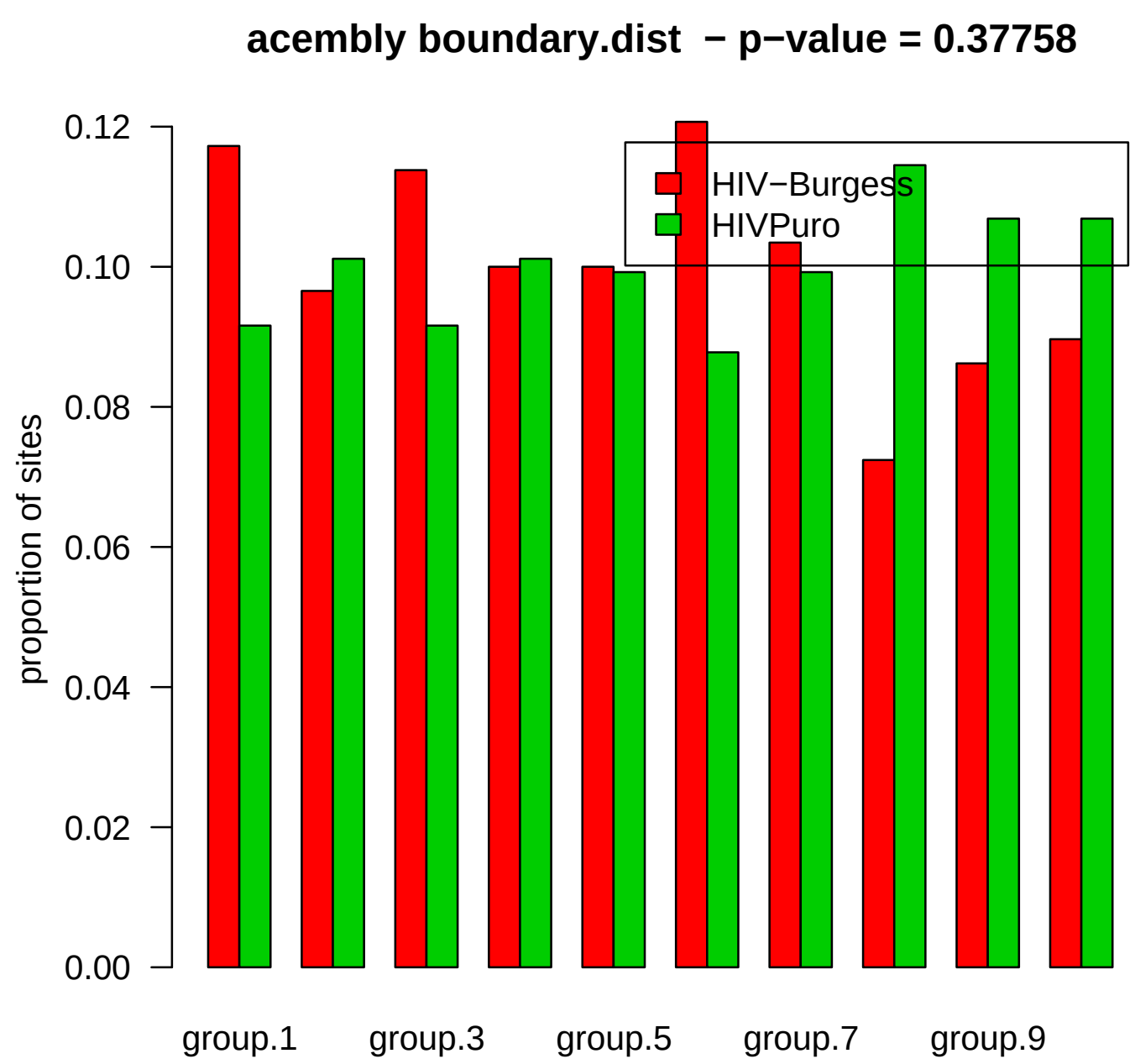

This plot studies the effect of nearness to the beginning of a transcript. For sites in genes, it is the distance to the start of the gene divided by the width of the gene. For other sites it is the distance from the site to the nearer gene if that gene boundary is also a transcription starting point. Locations near '0' are relatively near the beginning of transcription, while those near '1' are near the termination of the transcript.

```
 Category limits

          lower category      upper
1  0.0009294105  group.1 0.09256817
2  0.0925681712  group.2 0.18756371
3  0.1875637105  group.3 0.26255787
4  0.2625578735  group.4 0.34918394
5  0.3491839386  group.5 0.43963176
6  0.4396317624  group.6 0.54339454
7  0.5433945379  group.7 0.63546206
8  0.6354620571  group.8 0.74275959
9  0.7427595947  group.9 0.86813987
10 0.8681398661 group.10 0.99983050
```

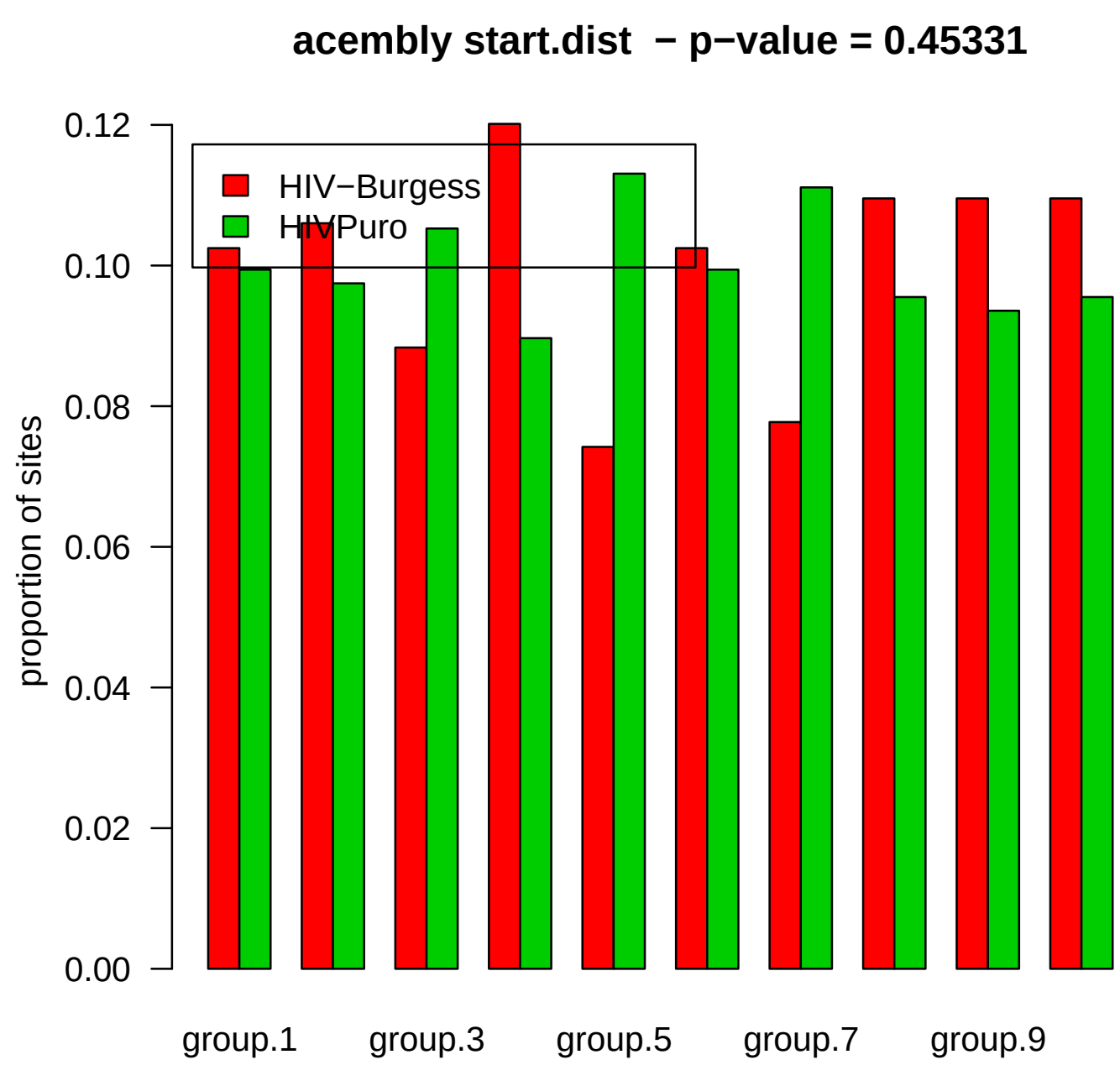

5.2 RefSeq Annotations

Category limits

	lower	category	upper
1	4339.0	group.1	23732.9
2	23732.9	group.2	35378.4
3	35378.4	group.3	46472.1
4	46472.1	group.4	63481.8
5	63481.8	group.5	80910.0
6	80910.0	group.6	102225.0
7	102225.0	group.7	131988.4
8	131988.4	group.8	166140.0
9	166140.0	group.9	247961.7
10	247961.7	group.10	992672.0

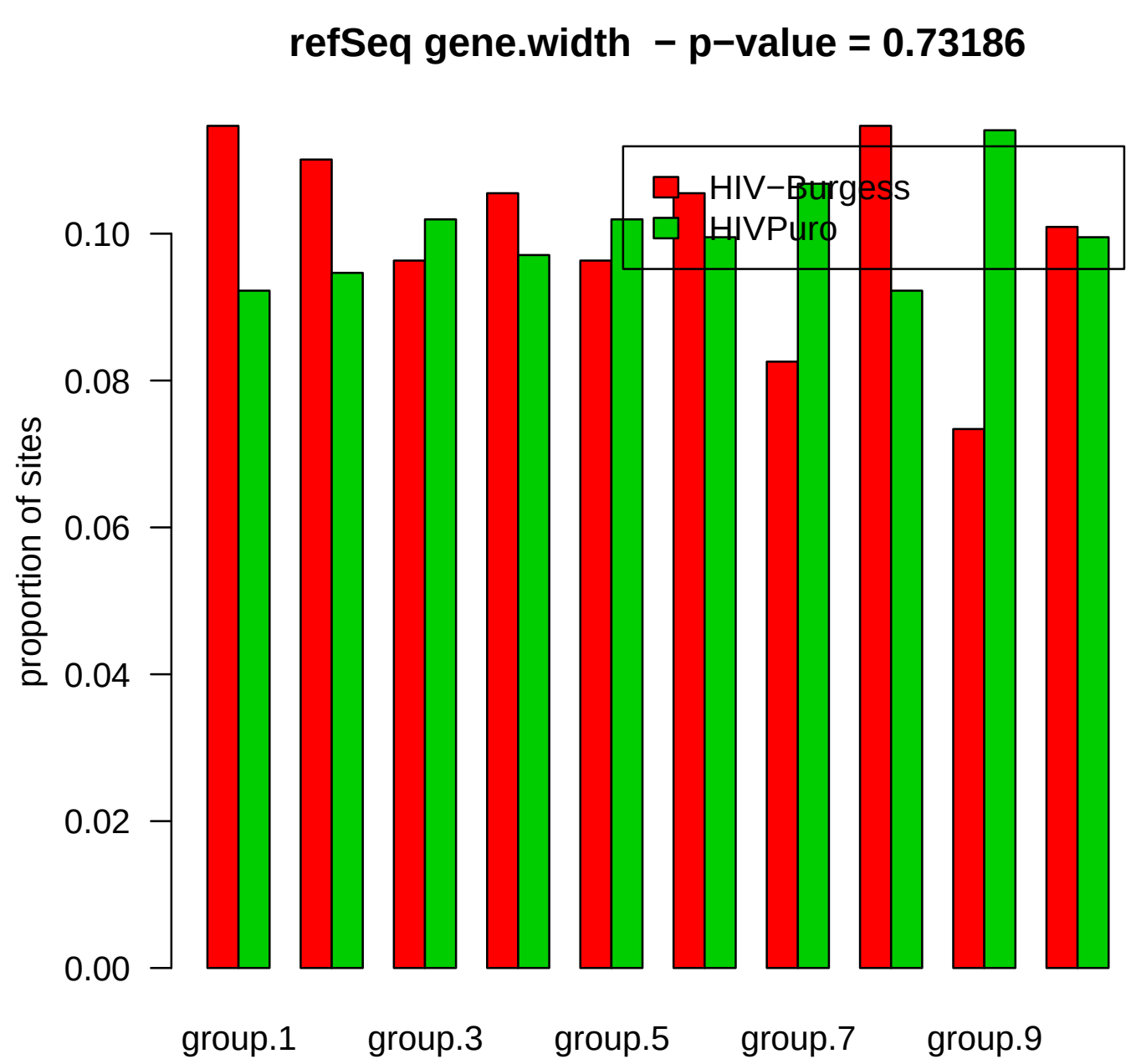

Category limits

	lower	category	upper
1	3478.0	group.1	24812.3
2	24812.3	group.2	48950.8
3	48950.8	group.3	76135.6
4	76135.6	group.4	119032.0

5	119032.0	group.5	185385.5
6	185385.5	group.6	337935.4
7	337935.4	group.7	540105.2
8	540105.2	group.8	923251.2
9	923251.2	group.9	1682141.6
10	1682141.6	group.10	21293005.0

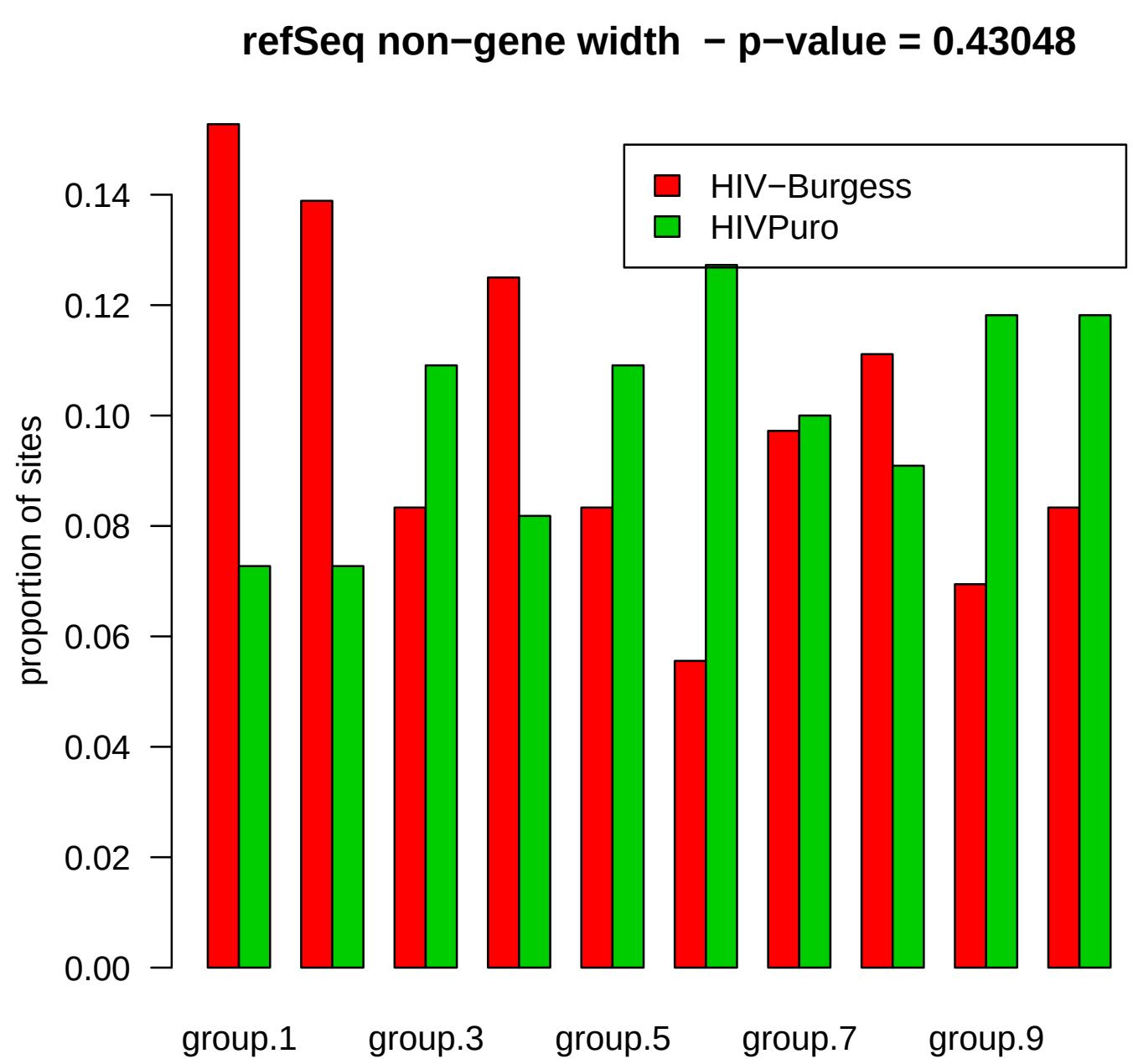

Category limits

	lower	category	upper
1	0.001127961	group.1	0.06017484
2	0.060174840	group.2	0.11592495
3	0.115924954	group.3	0.16239860
4	0.162398602	group.4	0.21729528
5	0.217295283	group.5	0.26540012
6	0.265400123	group.6	0.31453438
7	0.314534378	group.7	0.35773016
8	0.357730160	group.8	0.40532204
9	0.405322039	group.9	0.45348723
10	0.453487226	group.10	0.49990109

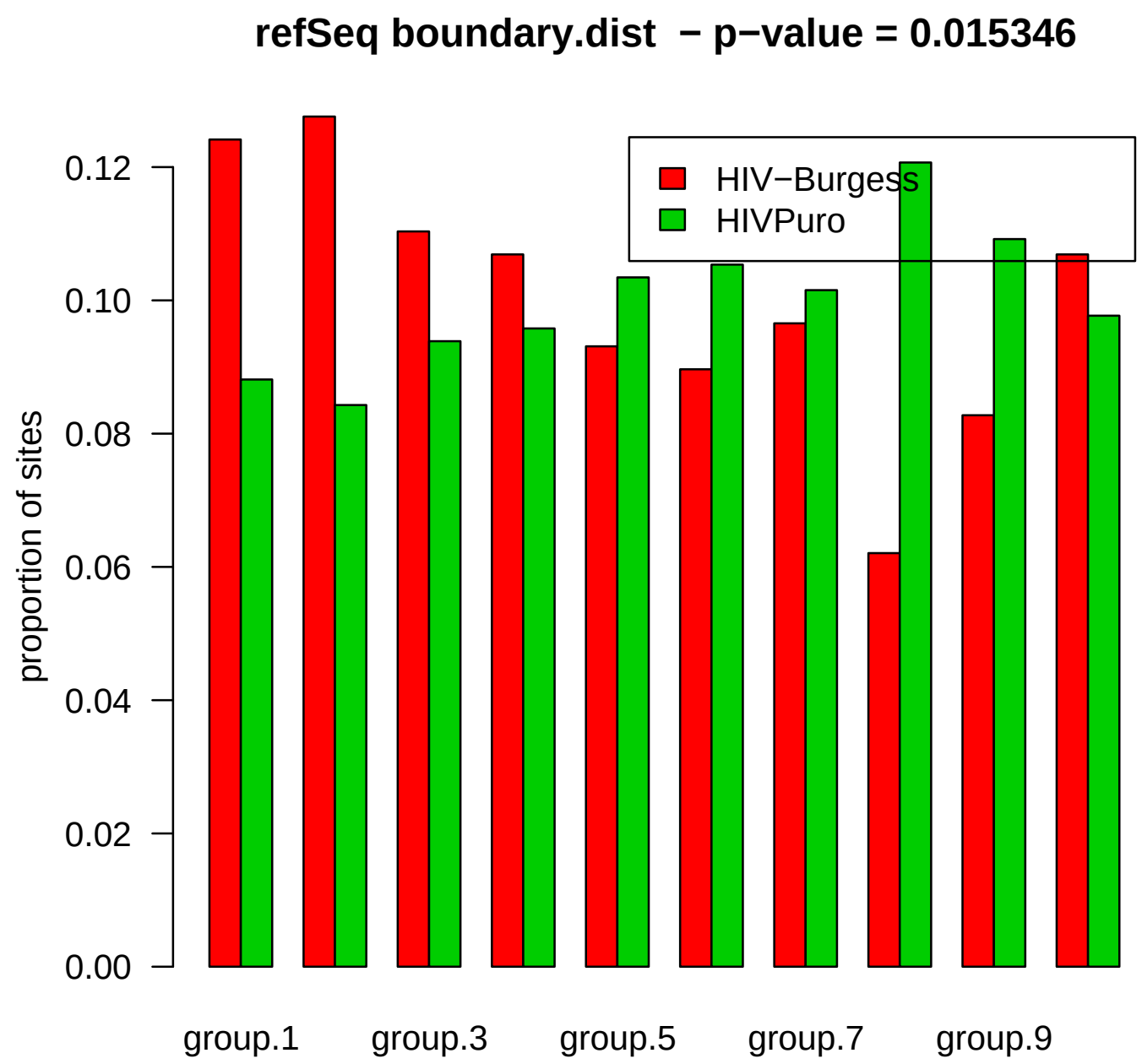

Category limits

	lower	category	upper
1	0.002402913	group.1	0.09925882
2	0.099258817	group.2	0.18405402
3	0.184054021	group.3	0.27356617
4	0.273566167	group.4	0.35470220
5	0.354702204	group.5	0.45746632
6	0.457466318	group.6	0.54922751
7	0.549227514	group.7	0.64173437
8	0.641734368	group.8	0.73845920
9	0.738459204	group.9	0.86435298
10	0.864352976	group.10	0.99887204

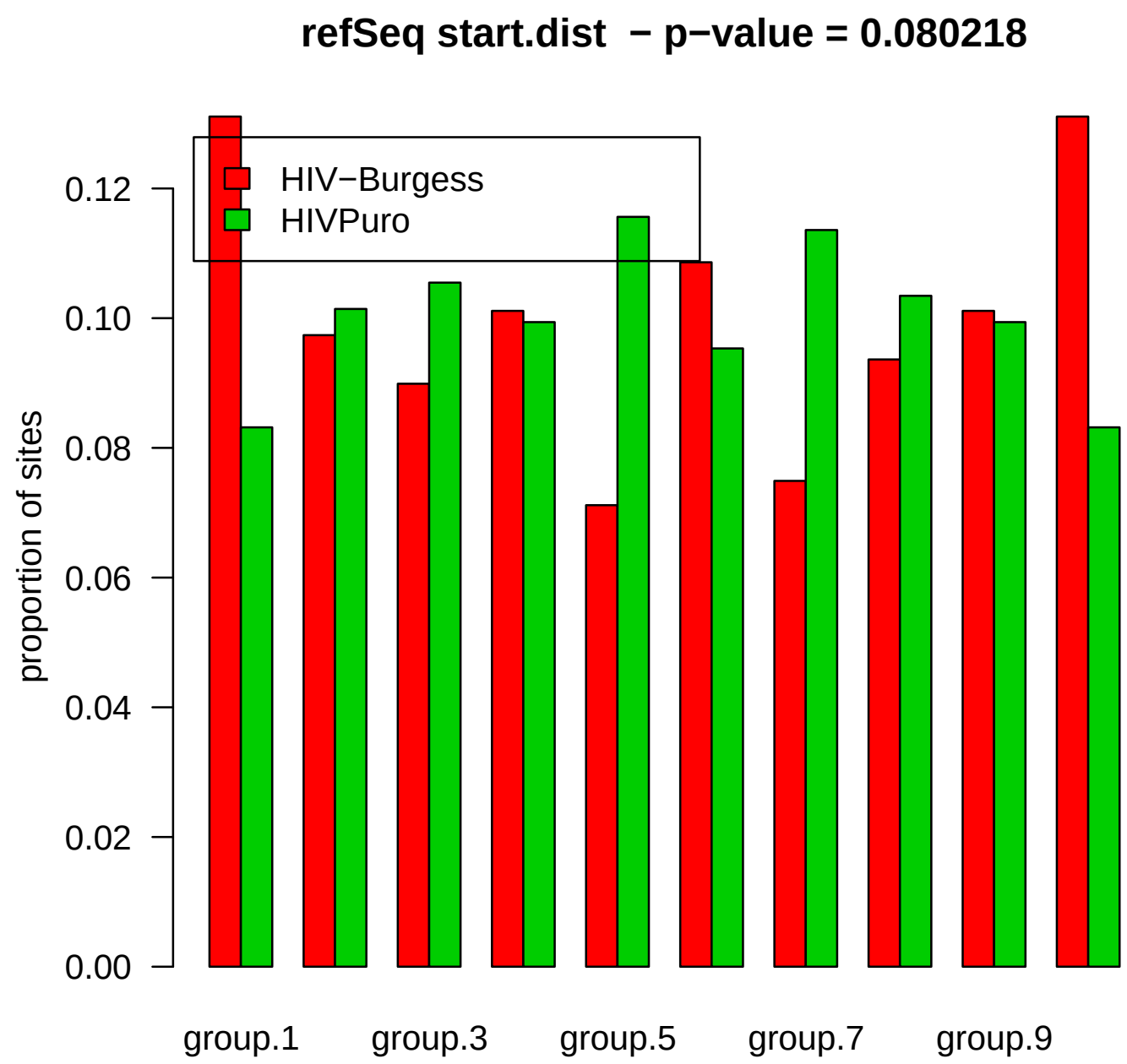

refSeq start.dist − p−value = 0.080218
HIV−Burgess
HIVPuro
proportion of sites
0.00
0.02
0.04
0.06
0.08
0.10
0.12
group.1
group.3
group.5
group.7
group.9

5.3 genScan Annotations

Category limits

	lower	category	upper
1	4684.0	group.1	24682.5
2	24682.5	group.2	39421.0
3	39421.0	group.3	53523.5
4	53523.5	group.4	72479.0
5	72479.0	group.5	88359.0
6	88359.0	group.6	113194.0
7	113194.0	group.7	139572.5
8	139572.5	group.8	184425.0
9	184425.0	group.9	230325.5
10	230325.5	group.10	644368.0

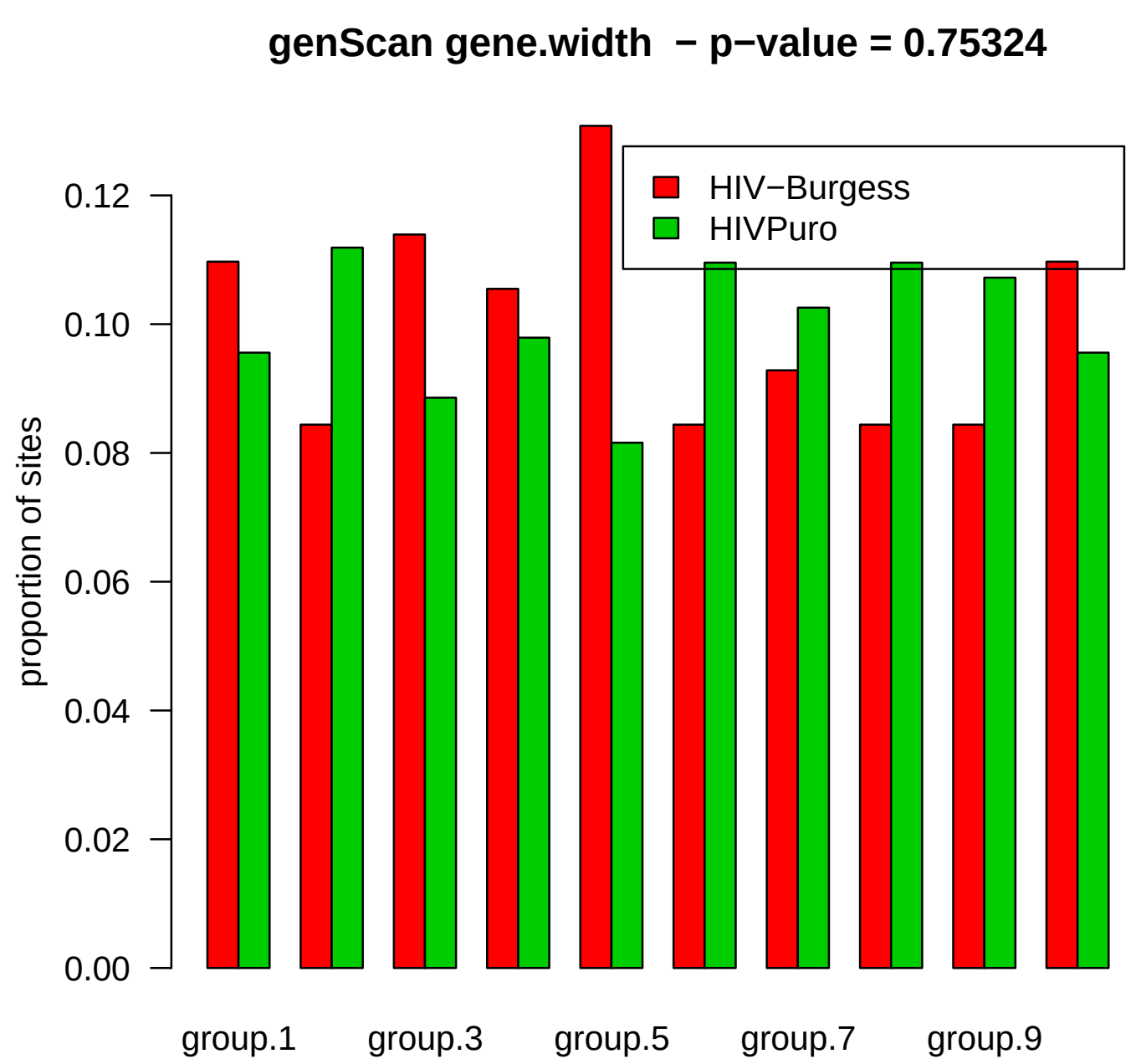

Category limits

	lower	category	upper
1	690.0	group.1	7004.7
2	7004.7	group.2	10520.4
3	10520.4	group.3	17265.9
4	17265.9	group.4	22417.6

5	22417.6	group.5	29233.0
6	29233.0	group.6	34344.4
7	34344.4	group.7	43923.5
8	43923.5	group.8	56102.0
9	56102.0	group.9	79819.1
10	79819.1	group.10	20489465.0

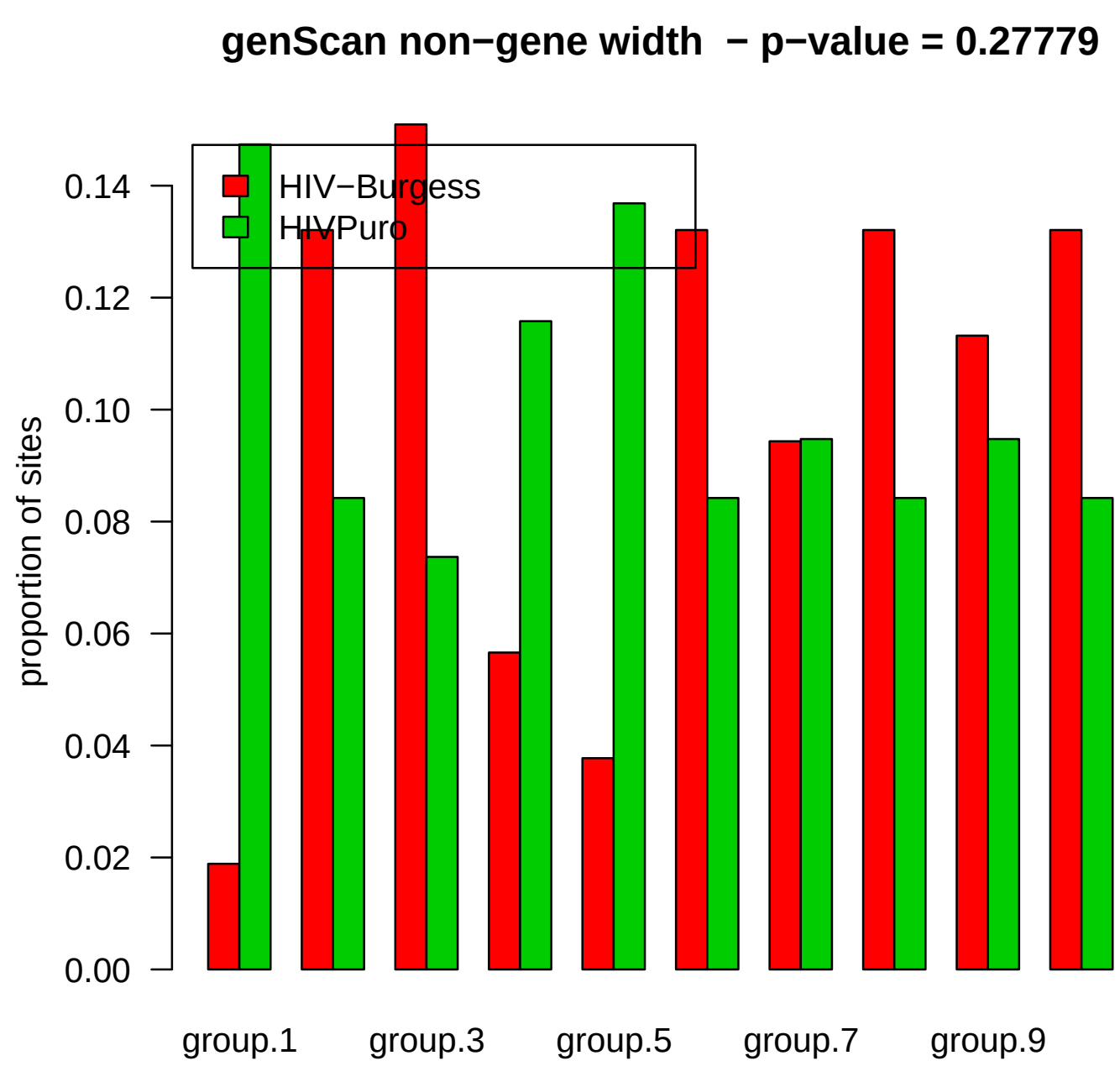

Category limits

	lower	category	upper
1	0.0005360666	group.1	0.0739102
2	0.0739101980	group.2	0.1333521
3	0.1333520781	group.3	0.1750946
4	0.1750945709	group.4	0.2191114
5	0.2191114320	group.5	0.2702058
6	0.2702058292	group.6	0.3190237
7	0.3190236584	group.7	0.3703712
8	0.3703711563	group.8	0.4080653
9	0.4080653451	group.9	0.4529110
10	0.4529109601	group.10	0.4993297

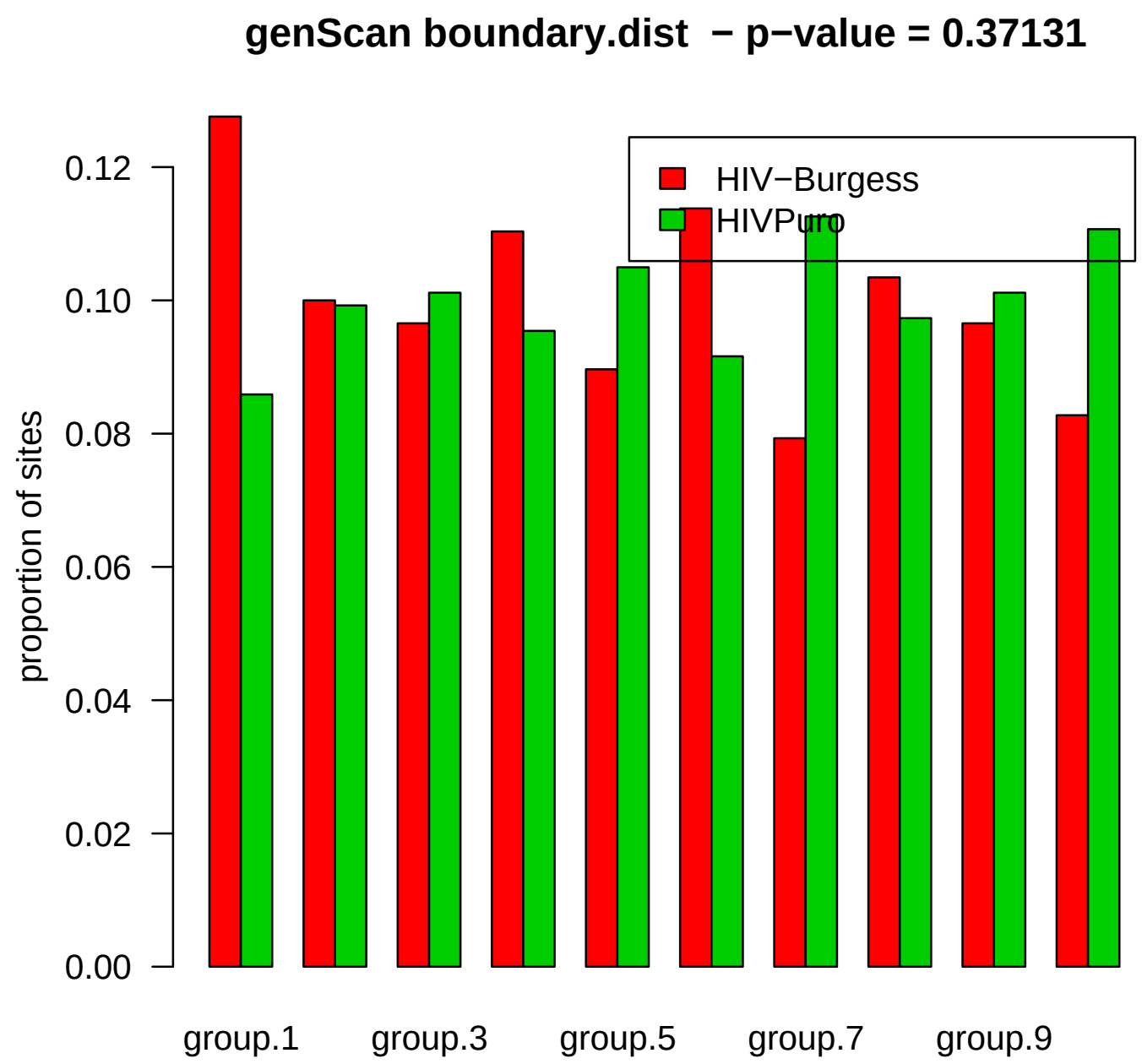

Category limits

	lower	category	upper
1	0.001081510	group.1	0.1227651
2	0.122765116	group.2	0.2001852
3	0.200185174	group.3	0.2909930
4	0.290992951	group.4	0.3781252
5	0.378125208	group.5	0.4806164
6	0.480616353	group.6	0.5672257
7	0.567225686	group.7	0.6356691
8	0.635669075	group.8	0.7596218
9	0.759621794	group.9	0.8533575
10	0.853357467	group.10	0.9994639

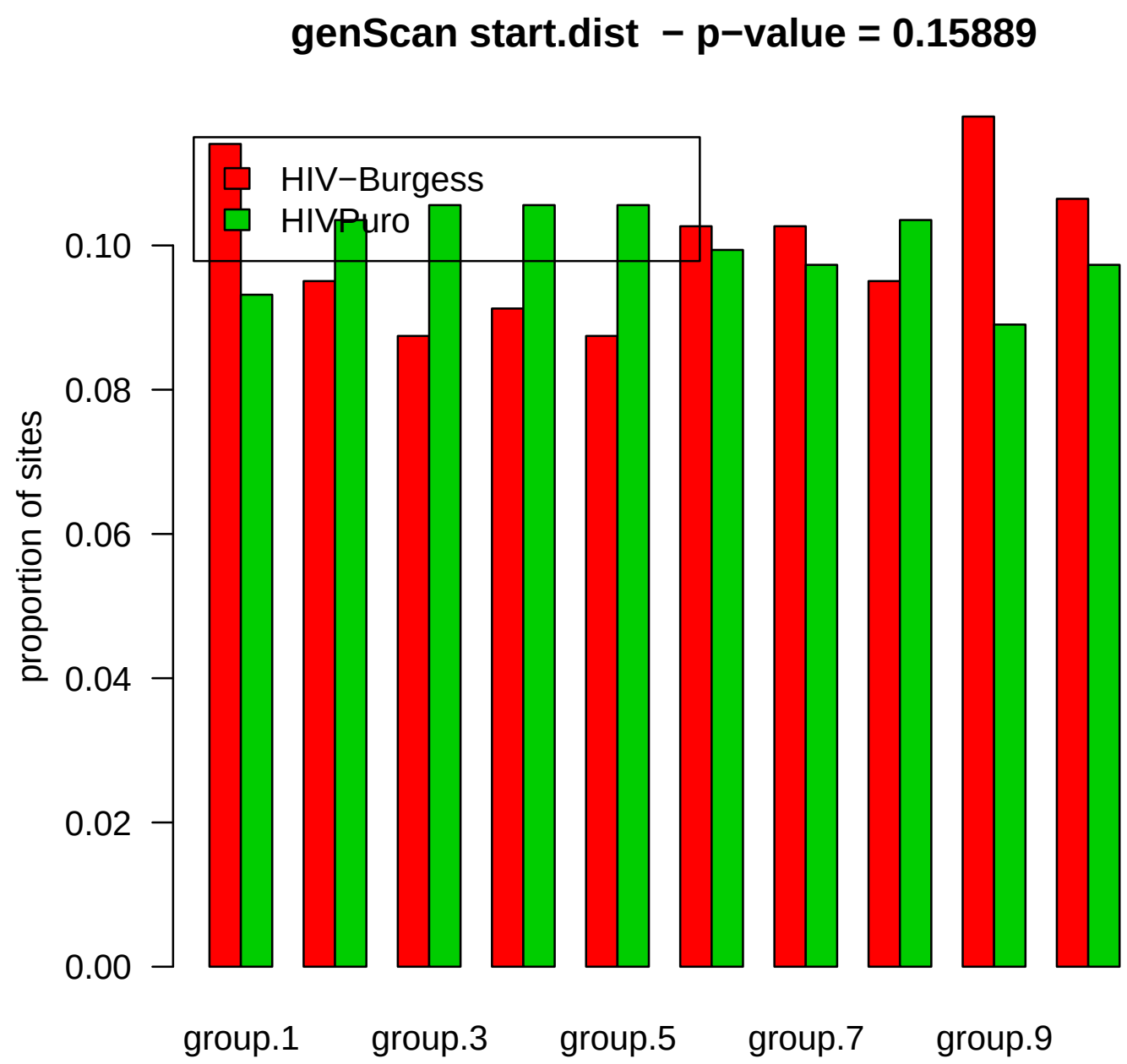
genScan start.dist − p−value = 0.15889
HIV−Burgess
HIVPuro
proportion of sites
0.00
0.02
0.04
0.06
0.08
0.10
group.1
group.3
group.5
group.7
group.9

5.4 uniGene Annotations

```
 Category limits

      lower category    upper
1    4684.0  group.1  24682.5
2   24682.5  group.2  39421.0
3   39421.0  group.3  53523.5
4   53523.5  group.4  72479.0
5   72479.0  group.5  88359.0
6   88359.0  group.6 113194.0
7  113194.0  group.7 139572.5
8  139572.5  group.8 184425.0
9  184425.0  group.9 230325.5
10 230325.5 group.10 644368.0
```

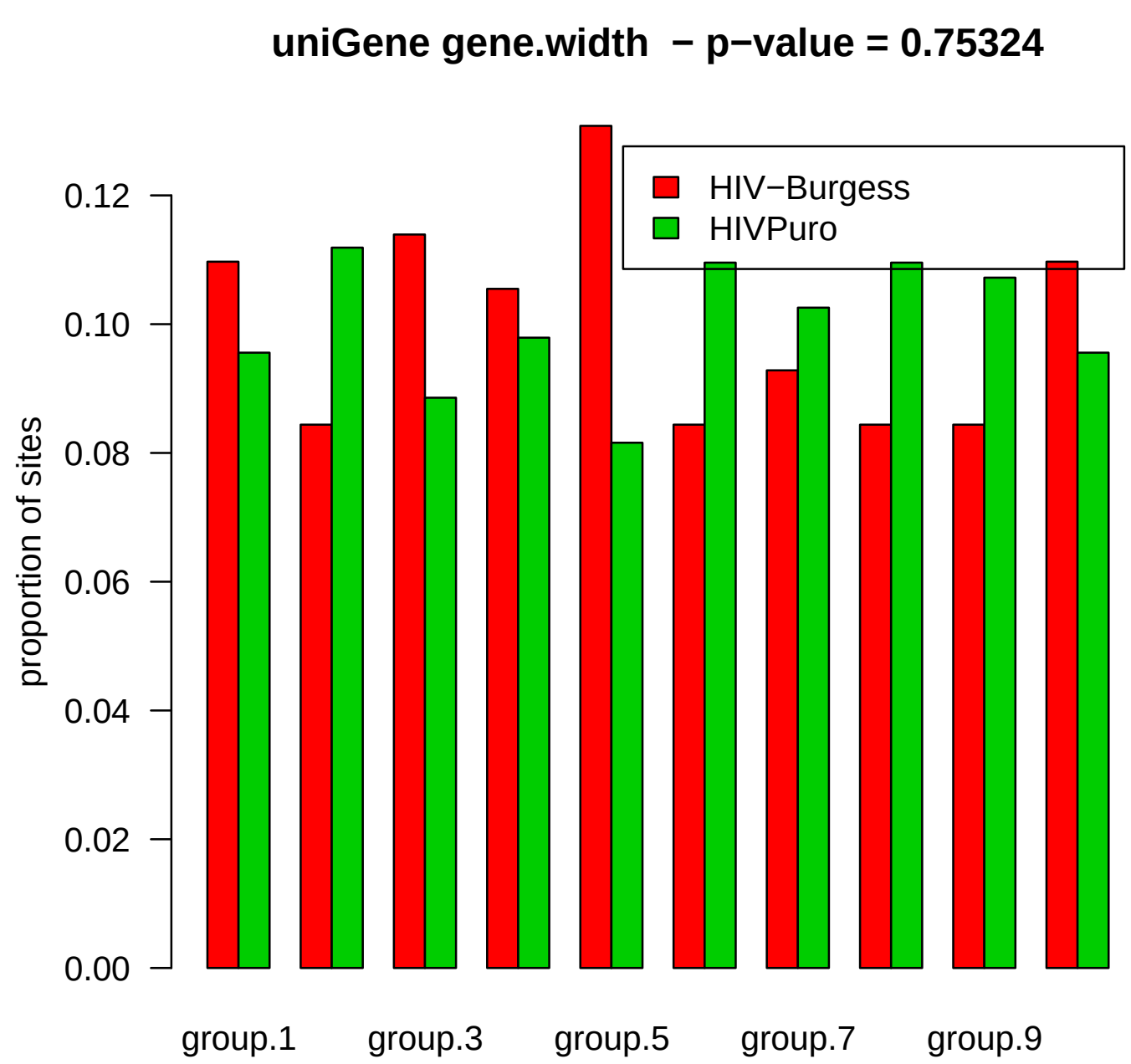


```
 Category limits

     lower category      upper
1    690.0  group.1     7004.7
2   7004.7  group.2    10520.4
3  10520.4  group.3    17265.9
4  17265.9  group.4    22417.6
```

5	22417.6	group.5	29233.0
6	29233.0	group.6	34344.4
7	34344.4	group.7	43923.5
8	43923.5	group.8	56102.0
9	56102.0	group.9	79819.1
10	79819.1	group.10	20489465.0

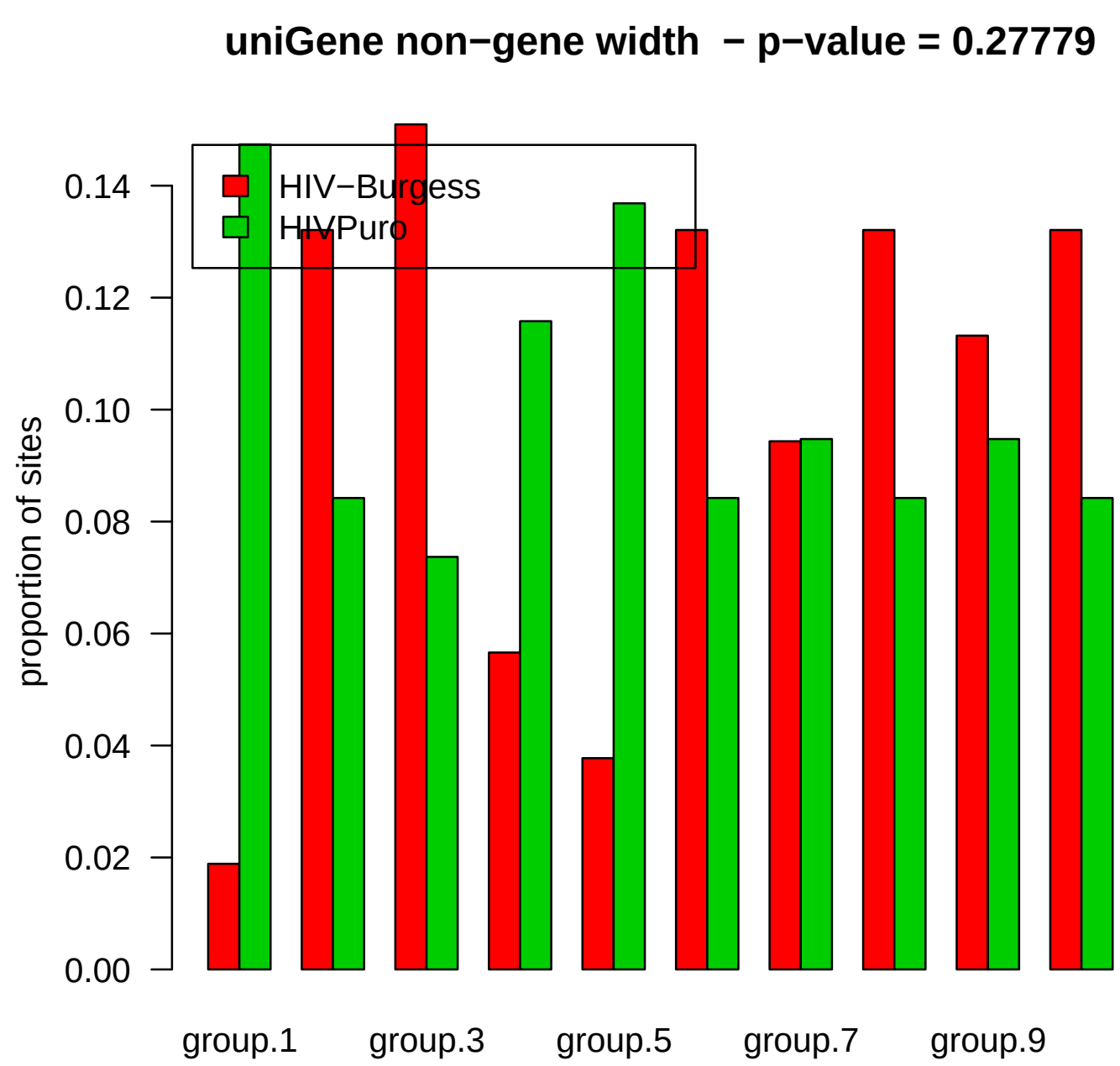

Category limits

	lower	category	upper
1	0.0005360666	group.1	0.0739102
2	0.0739101980	group.2	0.1333521
3	0.1333520781	group.3	0.1750946
4	0.1750945709	group.4	0.2191114
5	0.2191114320	group.5	0.2702058
6	0.2702058292	group.6	0.3190237
7	0.3190236584	group.7	0.3703712
8	0.3703711563	group.8	0.4080653
9	0.4080653451	group.9	0.4529110
10	0.4529109601	group.10	0.4993297

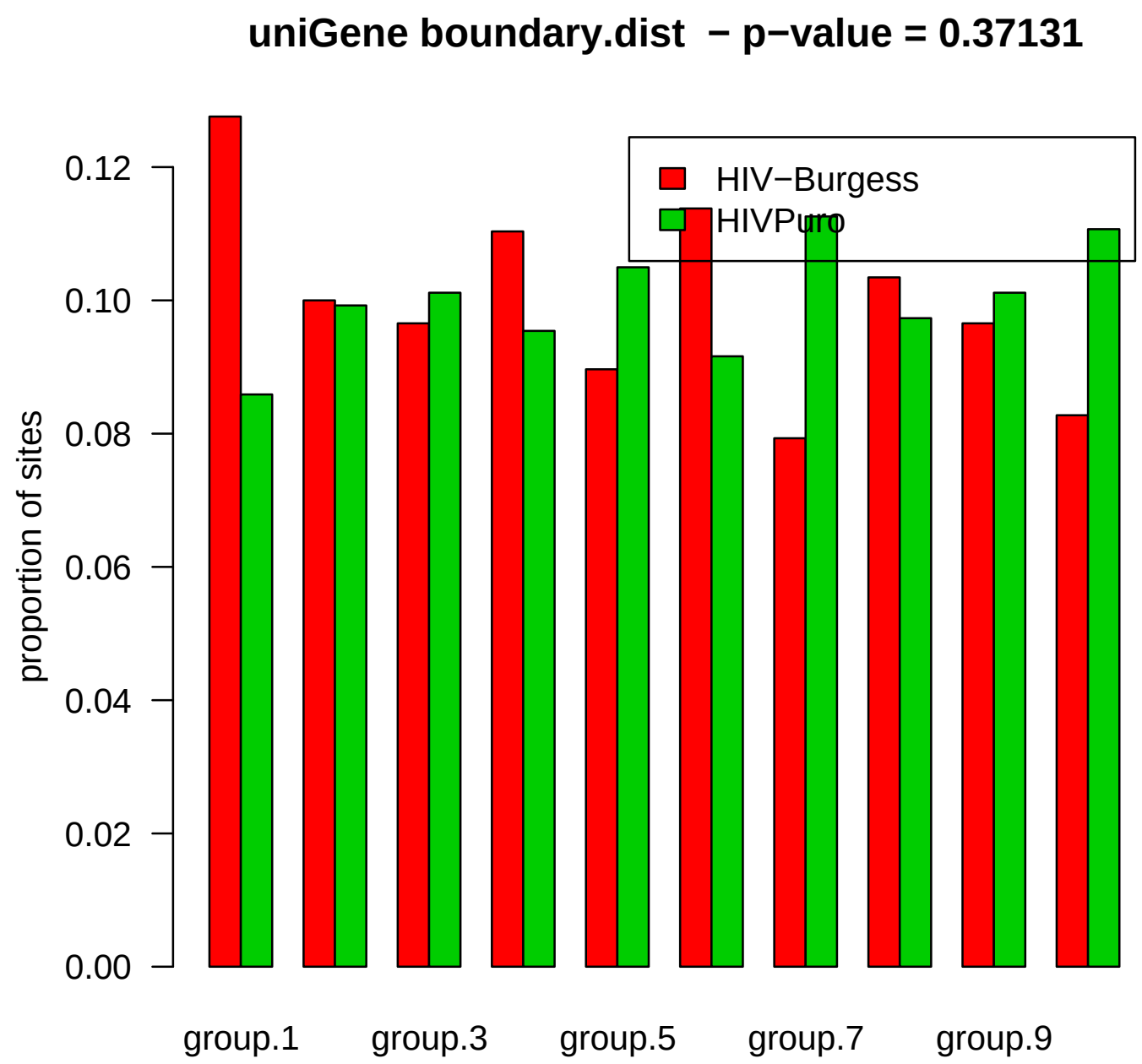

Category limits

	lower	category	upper
1	0.001081510	group.1	0.1227651
2	0.122765116	group.2	0.2001852
3	0.200185174	group.3	0.2909930
4	0.290992951	group.4	0.3781252
5	0.378125208	group.5	0.4806164
6	0.480616353	group.6	0.5672257
7	0.567225686	group.7	0.6356691
8	0.635669075	group.8	0.7596218
9	0.759621794	group.9	0.8533575
10	0.853357467	group.10	0.9994639

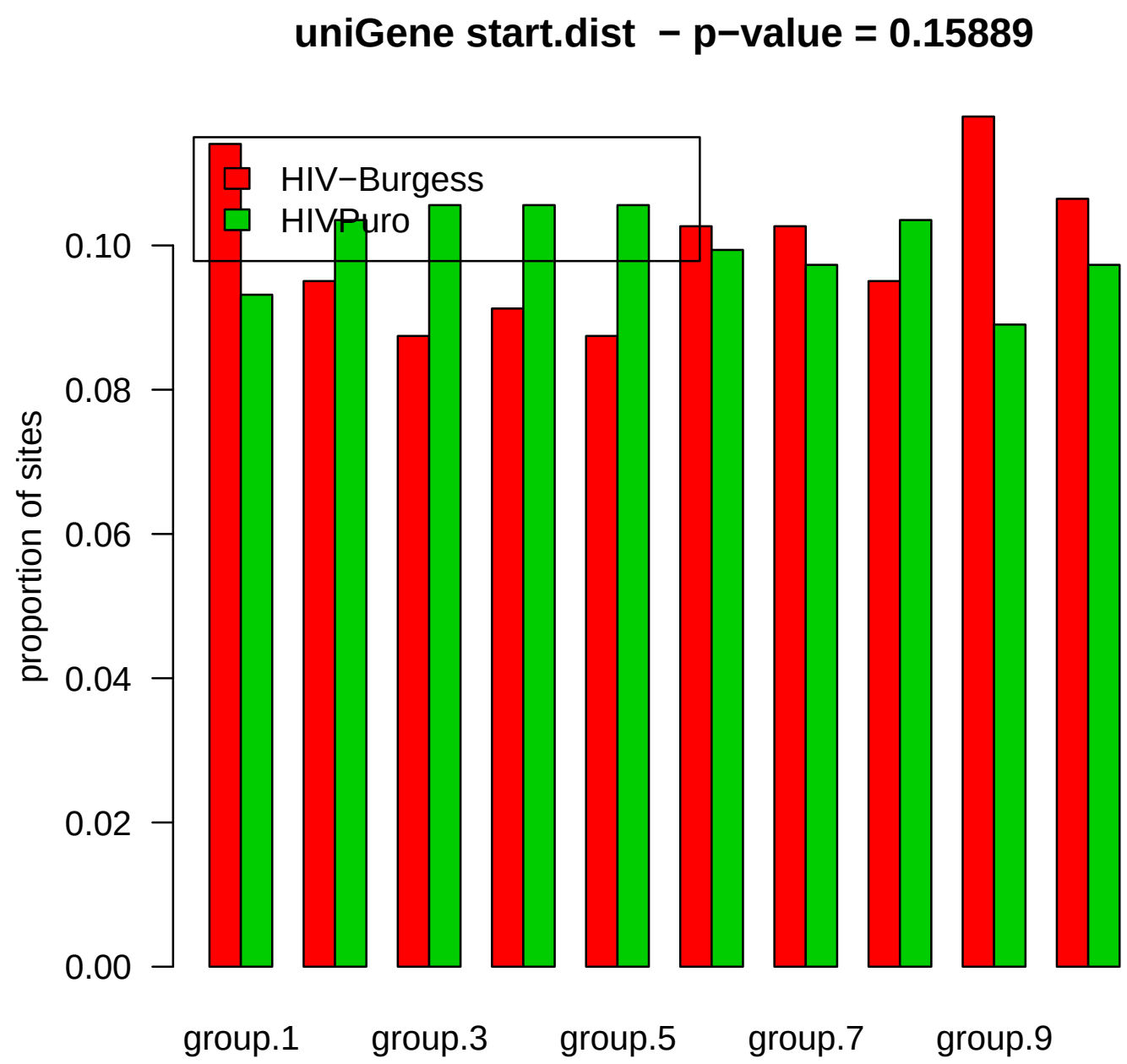

uniGene start.dist − p−value = 0.15889
HIV−Burgess
HIVPuro
proportion of sites
0.00
0.02
0.04
0.06
0.08
0.10
group.1
group.3
group.5
group.7
group.9

6 GC content

Here we study the effect of GC content on insertion. The GC content is taken from the Human Genome Draft at GoldenPath from the table `http://genome.ucsc.edu/goldenPath/hg17/database/gc5Base.txt.gz`.

Following the plot is a table of fitted coefficients based on splitting the GC percent data at the median.

```
 Category limits

      lower category    upper
1  28.49609  group.1 33.75000
2  33.75000  group.2 35.34766
3  35.34766  group.3 36.48828
4  36.48828  group.4 38.07812
5  38.07812  group.5 39.43359
6  39.43359  group.6 40.94531
7  40.94531  group.7 42.75781
8  42.75781  group.8 44.94531
9  44.94531  group.9 49.17188
10 49.17188 group.10 64.10156
```

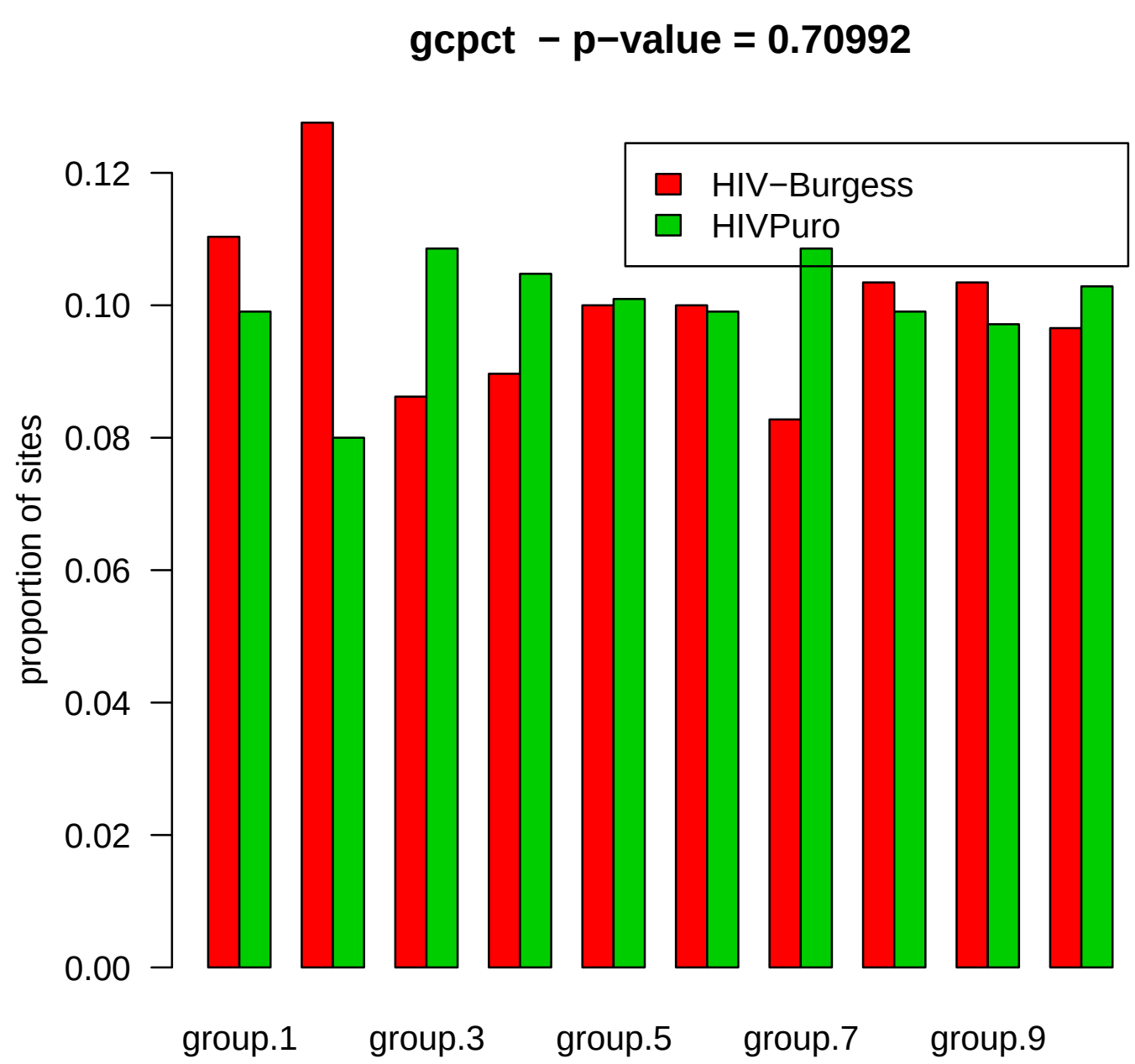

7 Cytobands

Here we study the association of cytoband with insertion intensity. The data are obtained from
`http://genome.ucsc.edu/goldenPath/hg17/database/cytoBand.txt.gz`.

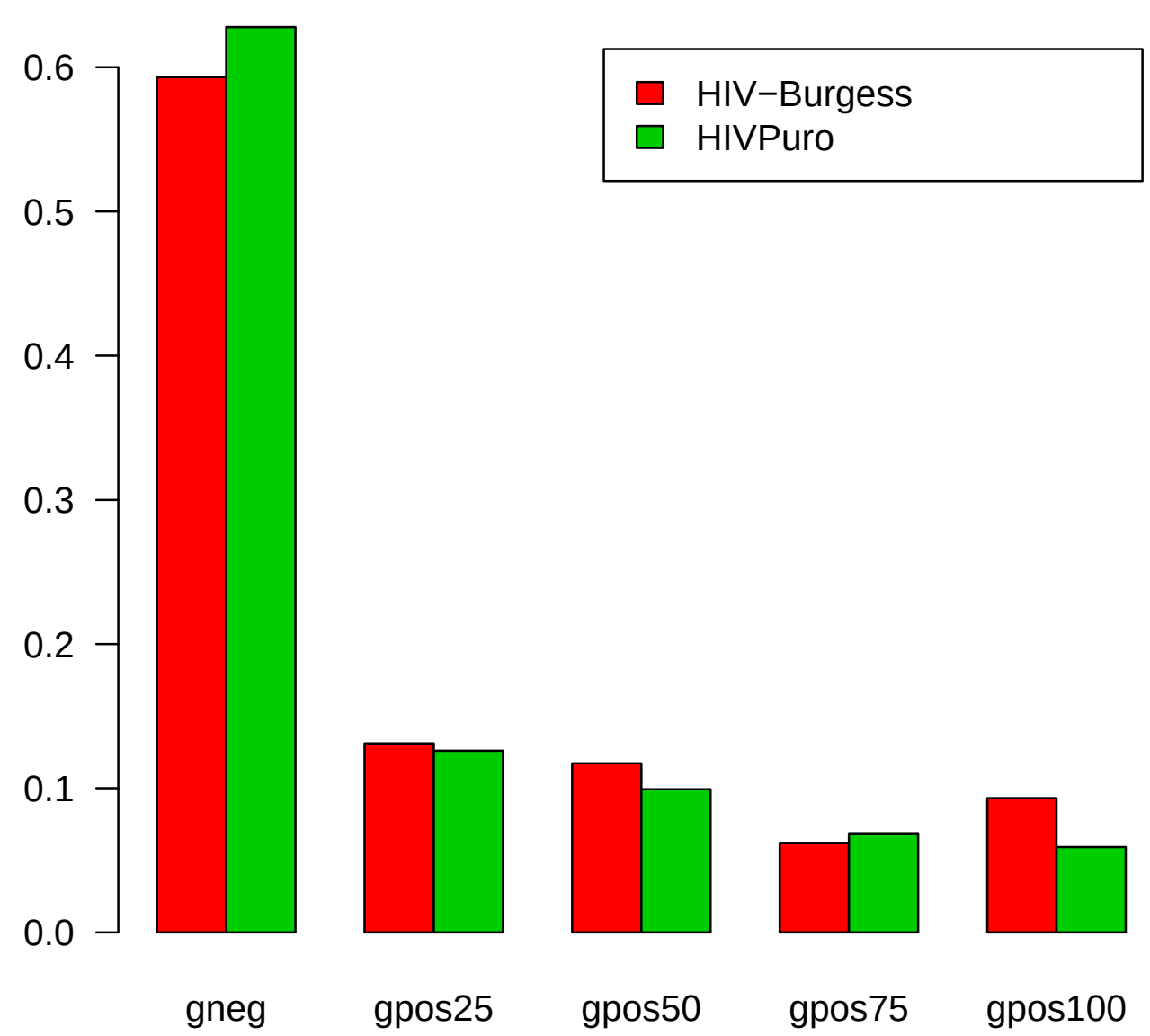

A formal test of significance attains a p-value of 0.41123.

References

[1] P. McCullagh and John A. Nelder. *Generalized linear models.* (Chapman & Hall ltd, 1999).

[2] Xiaolin Wu, Yuan Li, Bruce Crise, Shawn M. Burgess "Transcription Start Regions in the Human Genome Are Favored Targets for MLV Integration," *Science,* **300**(5626), (June 2003): 1749-1751.
