# Supplementary material for: Retroviral DNA Integration: Viral and Cellular Determinants of Target-Site Selection
Source: PLoS Pathog. 2006 Jun 23;2(6):e60. doi: 10.1371/journal.ppat.0020060 (PMC1480600; doi:10.1371/journal.ppat.0020060)
Supplement: Supplementary file 5 [file ppat.0020060.sd005.pdf]

Association of Genomic Features with Integration: Unselected vs. Puromycin-Selected MLV

April 21, 2006

Contents

1 Introduction **3**

2 Preference for Genes **4**
- 2.1 Acembly Genes 4
- 2.2 refGenes 6
- 2.3 ensGenes 8
- 2.4 genScan Genes 10
- 2.5 uniGenes 12

3 CpG Island Neighborhoods **14**
- 3.1 1 kilobase neighborhoods 14
- 3.2 5 kilobase neighborhoods 15
- 3.3 10 kilobase neighborhoods 16
- 3.4 25 kilobase neighborhoods 17
- 3.5 50 kilobase neighborhoods 18

4 Gene Density, Expression 'Density', and CpG Island Density **19**
- 4.1 25 kiloBase Window 20
- 4.2 50 kiloBase Window 26
- 4.3 100 kiloBase Window 31
- 4.4 250 kiloBase Window 36
- 4.5 500 kiloBase Window 41
- 4.6 1 megaBase Window 46
- 4.7 2 megaBase Window 51
- 4.8 4 megaBase Window 56
- 4.9 8 megaBase Window 61
- 4.10 16 megaBase Window 66
- 4.11 32 megaBase Window 71

5 Juxtaposition with Gene Start and End Positions **76**
5.1 Acembly Annotations 76
5.2 RefSeq Annotations 80
5.3 genScan Annotations 84
5.4 uniGene Annotations 88

6 GC content **92**

7 Cytobands **93**

1 Introduction

In this document, I examine the association of integration siting with various genomic features.

The numbers are shown below:

```
Origin.of.data.set
MLV-Burgess      MLVPuro
        917          544
```

The distribution of relative frequency of insertions across the chromosomes is given in this barplot:

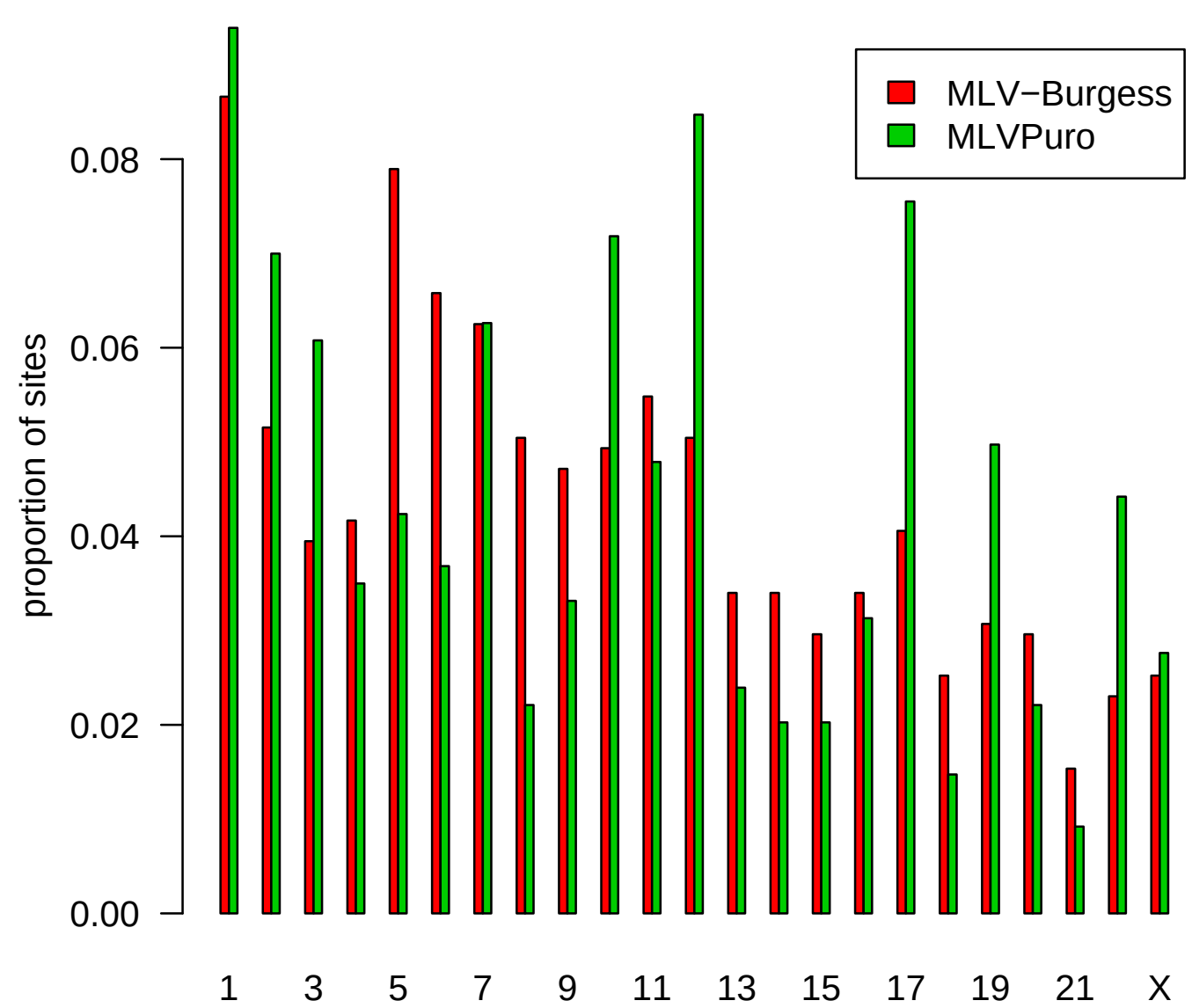

Are there chromosomes that are particularly favored for integration by one group over the other? This was tested for statistical significance. The test performed used the likelihood ratio statistic for the logistic regression model (reviewed in [1]) as implemented by the `glm` function of R using the `binomial` family. The null hypothesis tested is the ratio of true integration events in the two groups is constant across all chromosomes. This test attains a p-value of $1.6095e-05$.

2 Preference for Genes

2.1 Acembly Genes

Here we examine the relative preference that integration events in the two groups have for genes. In the following plot we show the relative frequency of integrations in genes according to the 'Acembly' annotation. The bars grouped over the label "In Gene" give the relative frequency of integration events (compared to control sites) between bases located within Acembly gene annotations, while the label "Not in Gene" give the relative frequency of integration events (compared to control sites) between bases not located within Acembly gene annotations.

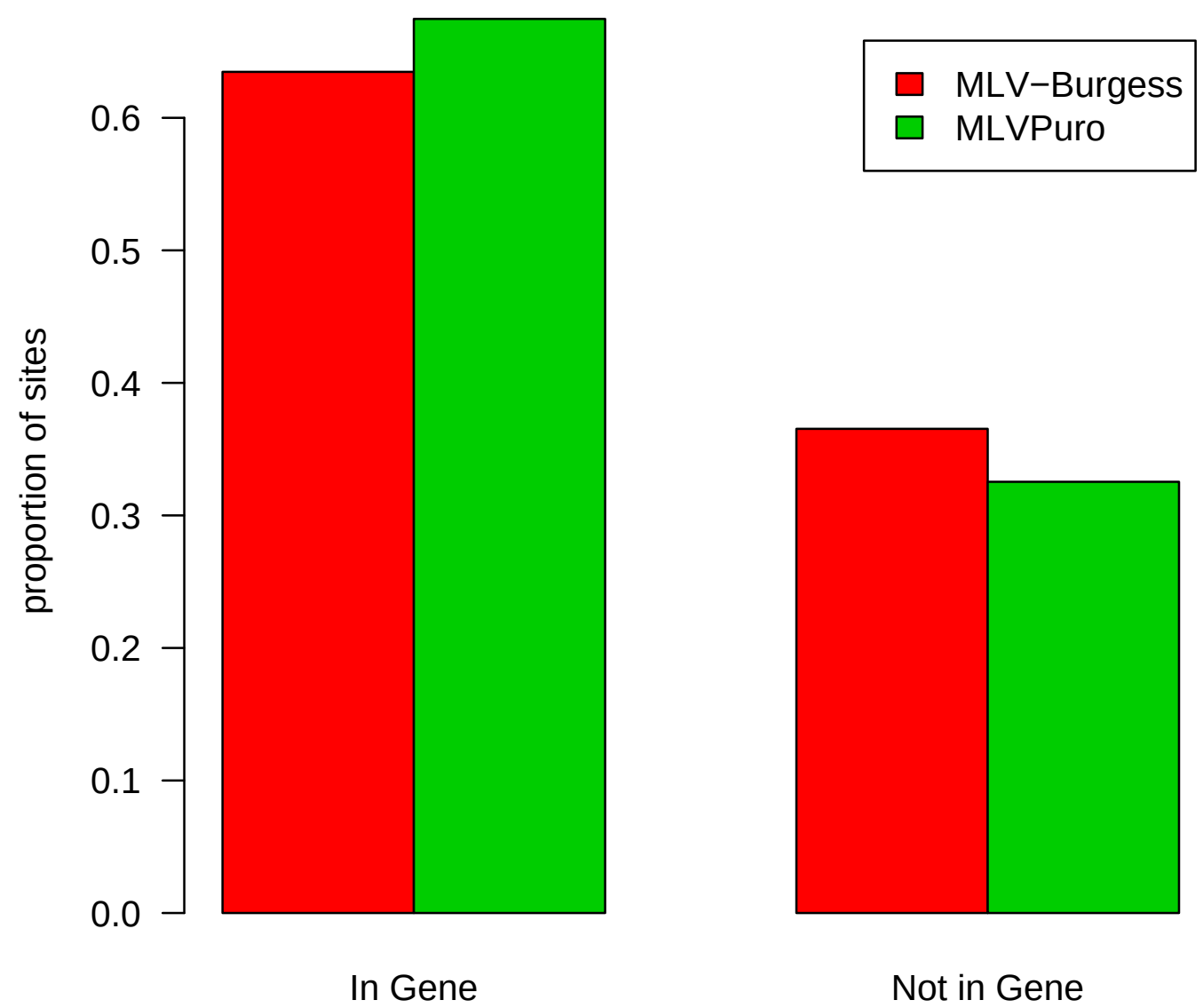

Is there is a difference in the tendency for insertions to occur in genes? A formal test of significance yields a p-value of 0.12085. In the following plot we show the relative frequency of insertions in exons according to the 'Acembly' annotation The bars grouped over the label "In Exon" give the relative frequency of integration events (compared to control sites) between bases located in exons according to the Acembly annotation, while the label "Not in Exon" give the relative frequency of integration events (compared to control sites) between bases not located in exons according to the Acembly gene annotation.

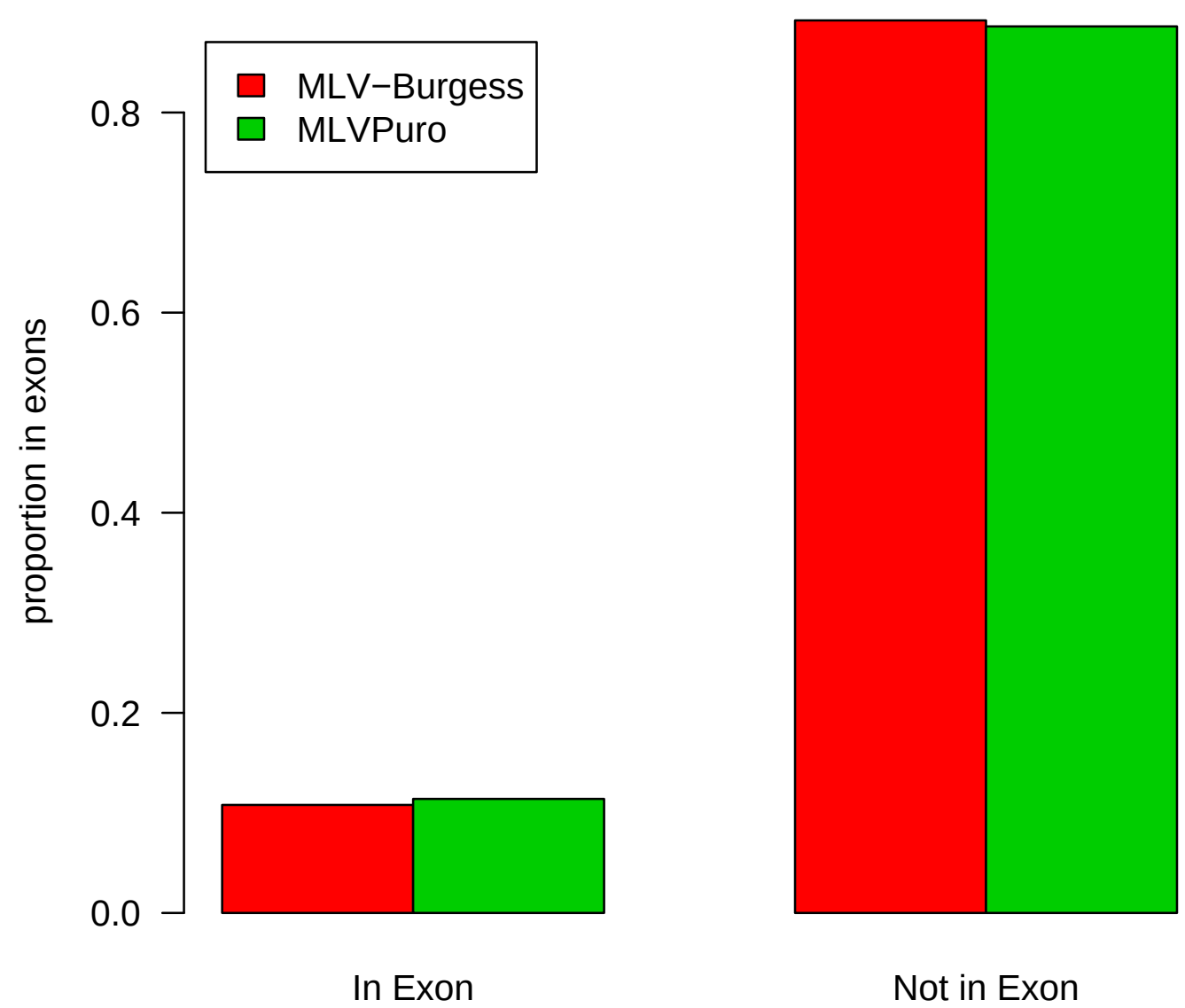

Here is the table of coefficients of the log ratio of intensities along with their standard errors, z statistics, and p-values:

	coef	se	z	p
(Intercept)	-0.63800	0.0929	-6.8700	6.62e-12
in.gene	0.17800	0.1180	1.5100	1.32e-01
in.exon	-0.00828	0.1780	-0.0466	9.63e-01

The model on which these coefficients are based include terms for whether the site is in a gene or not. Thus, the effect shown as 'in.exon' is net of that due to being in a gene. Note that in the barplot above the 'Not in Exon' bars include the both introns and intergenic regions, so the impression given by the table may differ from that for the barplot.

2.2 refGenes

Here we examine the relative preference that insertions of the two types have for genes. In the following plot we show the relative frequency of insertions in genes according to the 'refGene' annotation.

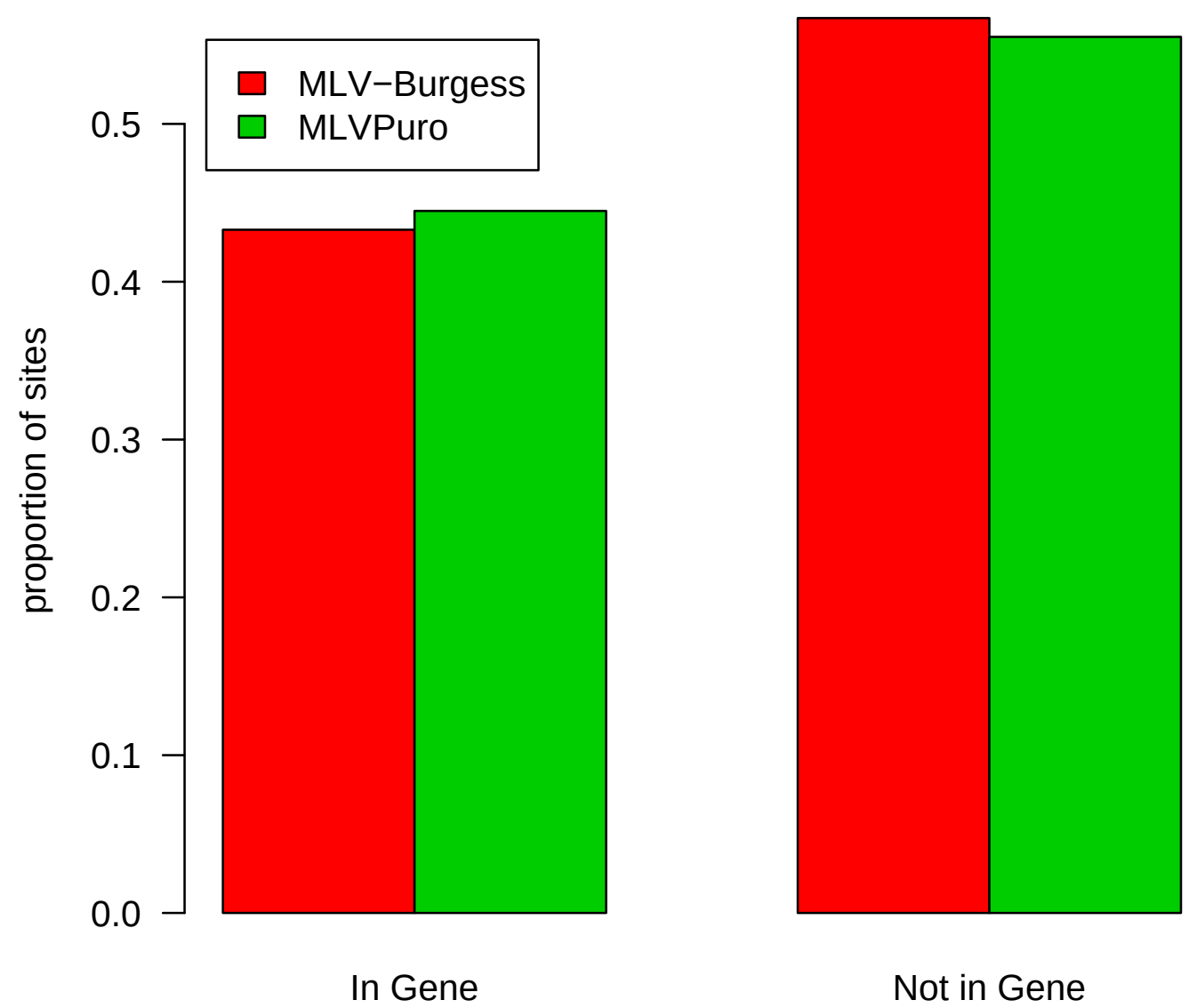

Is there is a tendency for insertions to occur in genes? A formal test of significance yields a p-value of 0.65712.

In the following plot we show the relative frequency of insertions in exons according to the 'refGene' annotation.

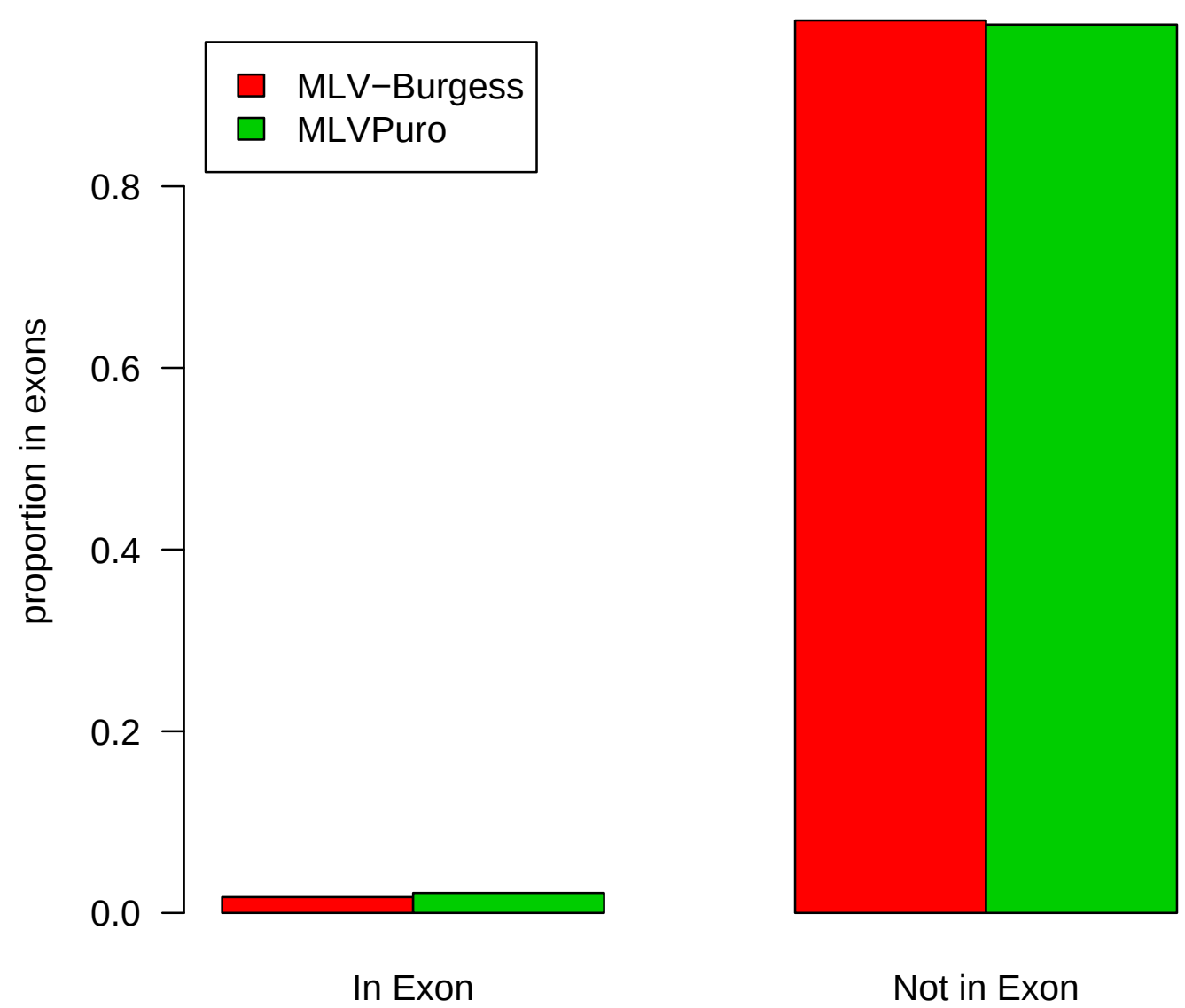

Here is the table of coefficients of the log ratio of intensities for along with their standard errors, z statistics, and p-values:

	coef	se	z	p
(Intercept)	-0.5430	0.0723	-7.510	5.87e-14
in.gene	0.0387	0.1100	0.350	7.26e-01
in.exon	0.2170	0.3910	0.555	5.79e-01

The model on which these coefficients are based include terms for whether the site is in a gene or not. Thus, the effect shown as 'in.exon' is net of that due to being in a gene. Note that in the barplot above the 'Not in Exon' bars include the both introns and intergenic regions, so the impression given by the table may differ from that for the barplot.

2.3 ensGenes

Here we examine the relative preference that insertions of the two types have for genes. In the following plot we show the relative frequency of insertions in genes according to the 'ensGene' annotation.

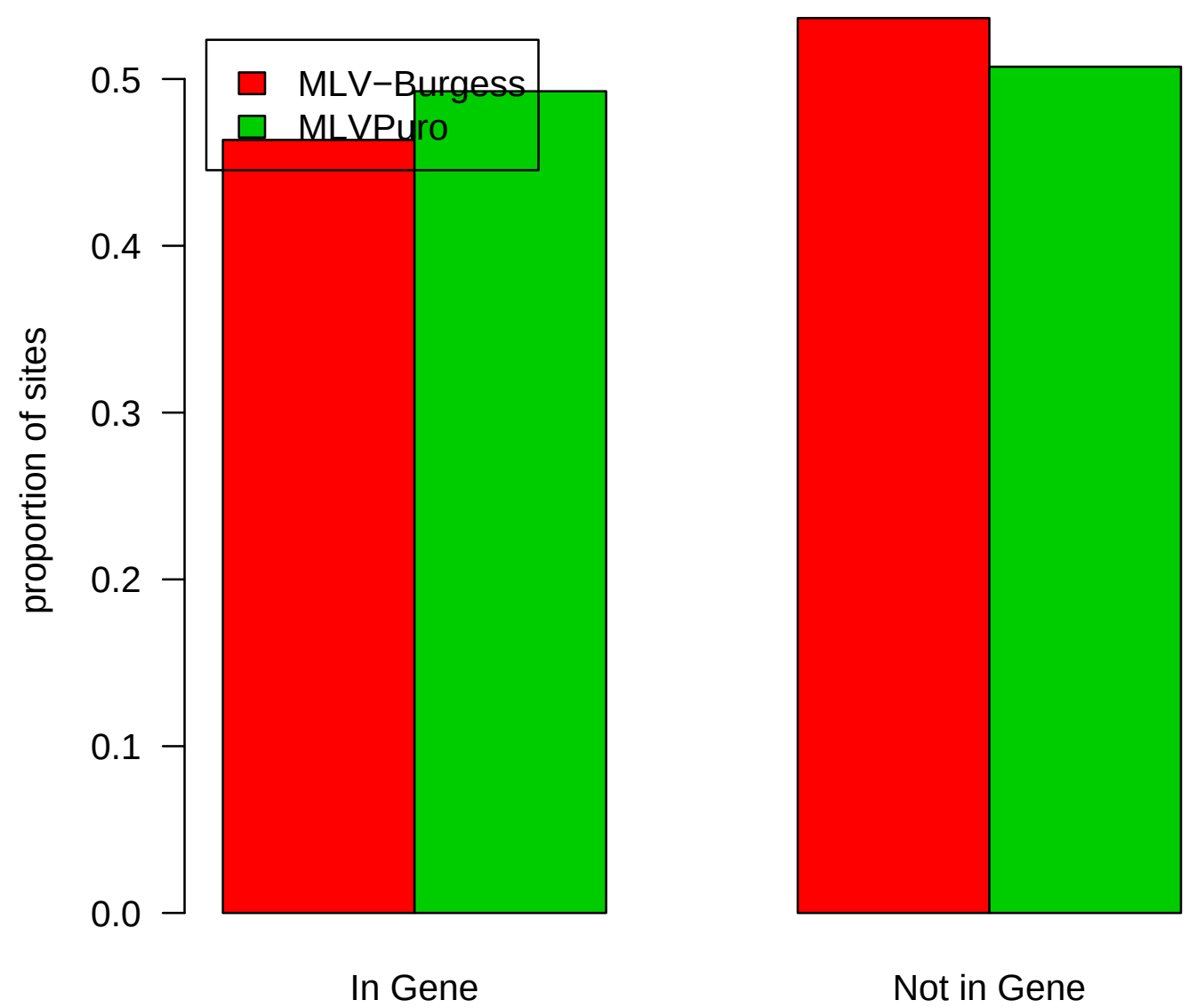

Is there is a tendency for insertions to occur in genes? A formal test of significance yields a p-value of 0.28032.

In the following plot we show the relative frequency of insertions in exons according to the 'ensGene' annotation.

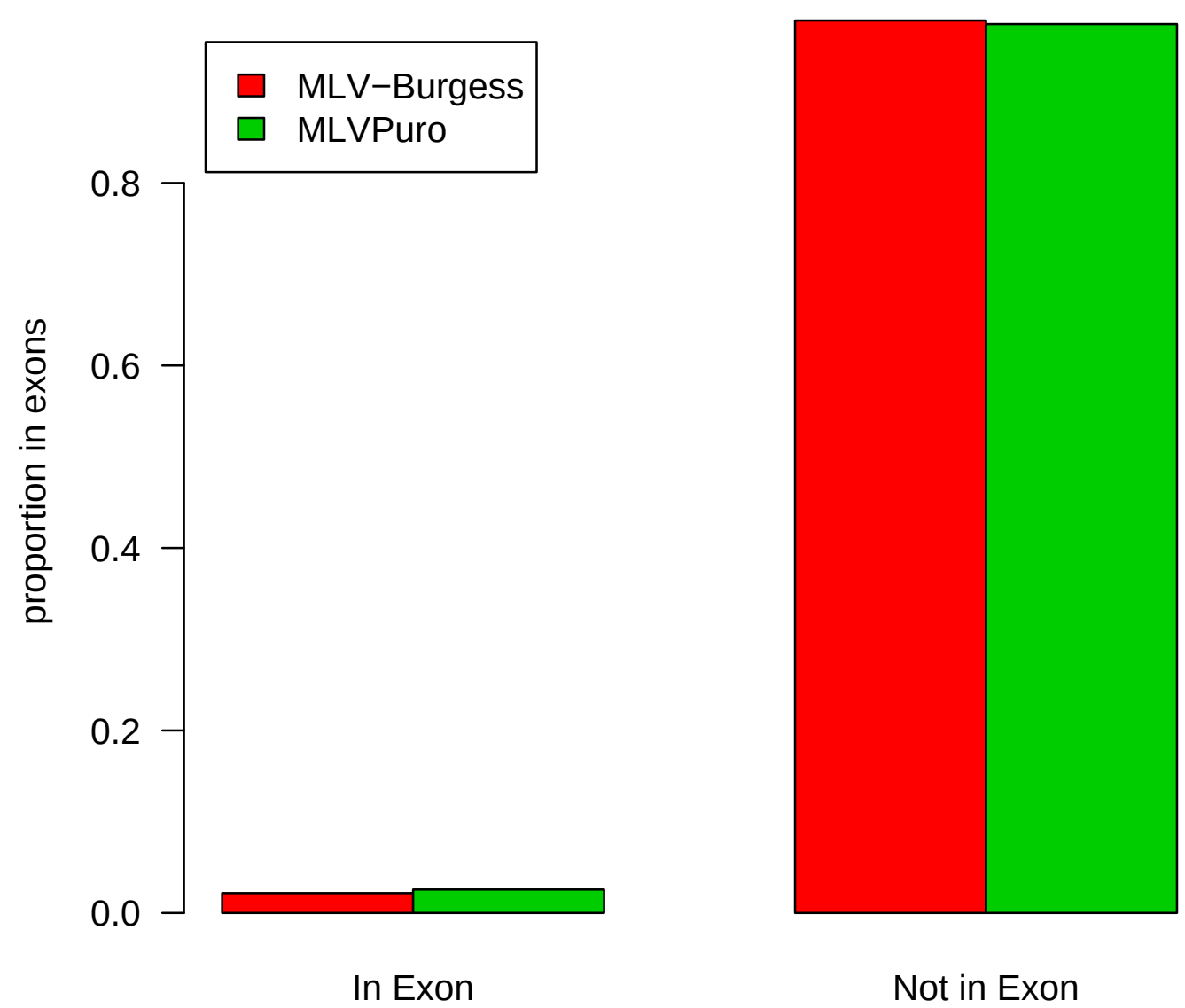

Here is the table of coefficients of the log ratio of intensities for along with their standard errors, z statistics, and p-values:

	coef	se	z	p
(Intercept)	-0.578	0.0752	-7.690	1.51e-14
in.gene	0.112	0.1100	1.020	3.10e-01
in.exon	0.110	0.3580	0.307	7.59e-01

The model on which these coefficients are based include terms for whether the site is in a gene or not. Thus, the effect shown as 'in.exon' is net of that due to being in a gene. Note that in the barplot above the 'Not in Exon' bars include the both introns and intergenic regions, so the impression given by the table may differ from that for the barplot.

2.4 genScan Genes

Here we examine the p erence that insertions have for genes. In the following plot we show the relative frequency of insertions in genes according to the 'genScan' annotation.

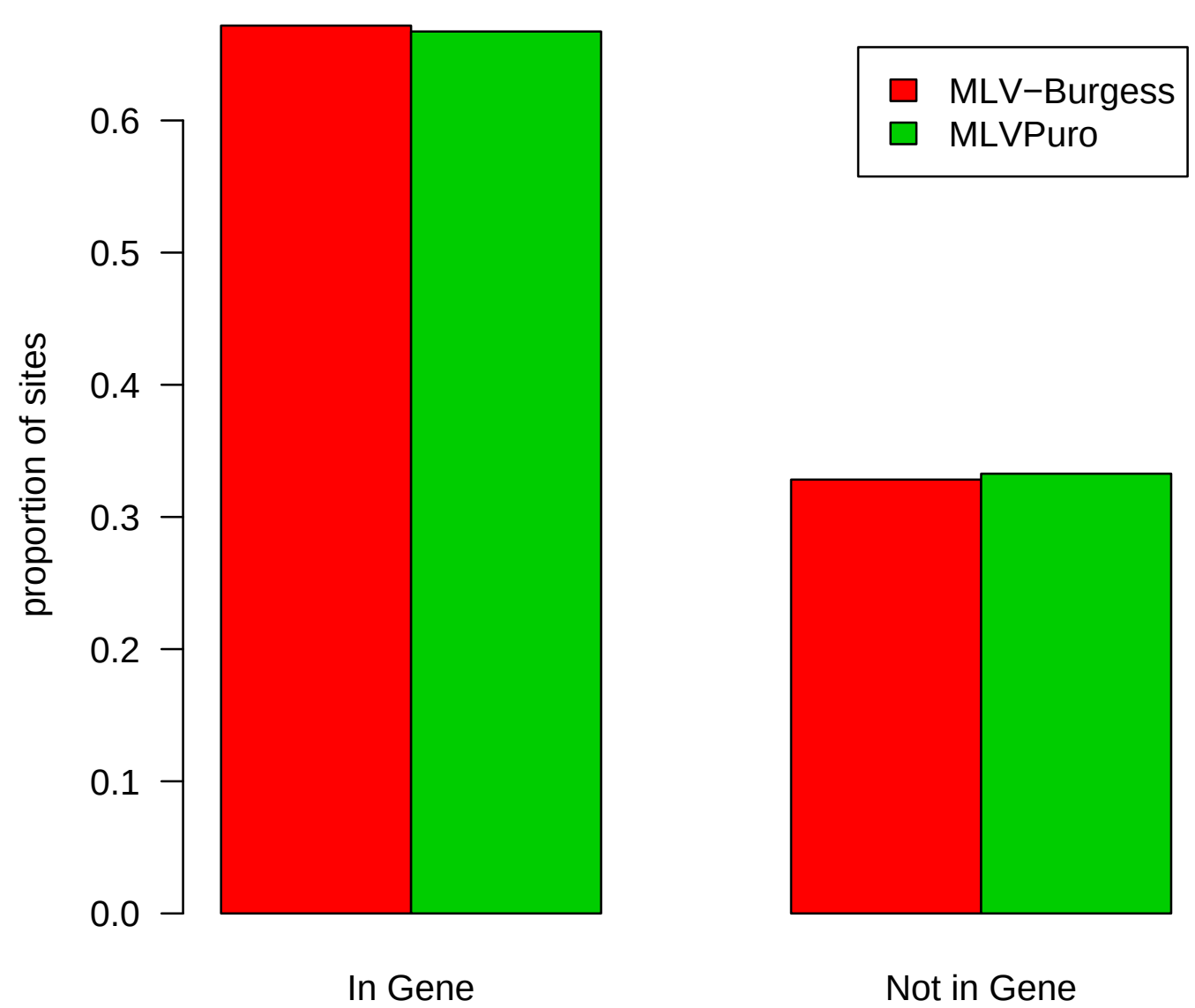

Is there is a tendency for insertions to occur in genes? A formal test of significance yields a p-value of 0.8604.

In the following plot we show the relative frequency of insertions in exons according to the 'genScan' annotation.

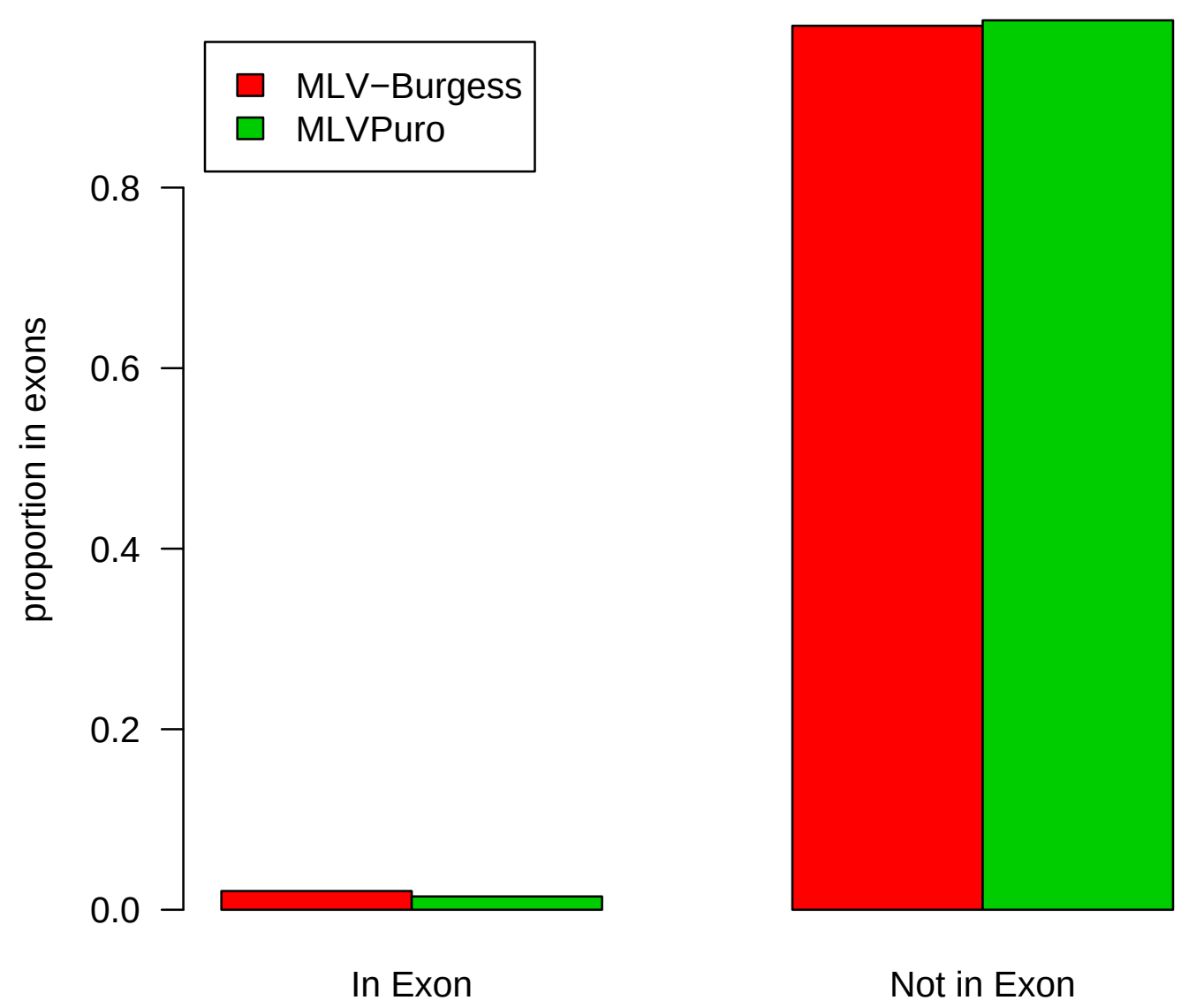

Here is the table of coefficients of the log ratio of intensities along with their standard errors, z statistics, and p-values:

	coef	se	z	p
(Intercept)	-0.5090	0.0941	-5.4100	6.40e-08
in.gene	-0.0112	0.1150	-0.0969	9.23e-01
in.exon	-0.3450	0.4270	-0.8090	4.19e-01

The model on which these coefficients are based include terms for whether the site is in a gene or not. Thus, the effect shown as 'in.exon' is net of that due to being in a gene. Note that in the barplot above the 'Not in Exon' bars include the both introns and intergenic regions, so the impression given by the table may differ from that for the barplot.

2.5 uniGenes

Here we examine the preference that insertions have for genes. In the following plot we show the relative frequency of insertions in genes according to the 'uniGene' annotation.

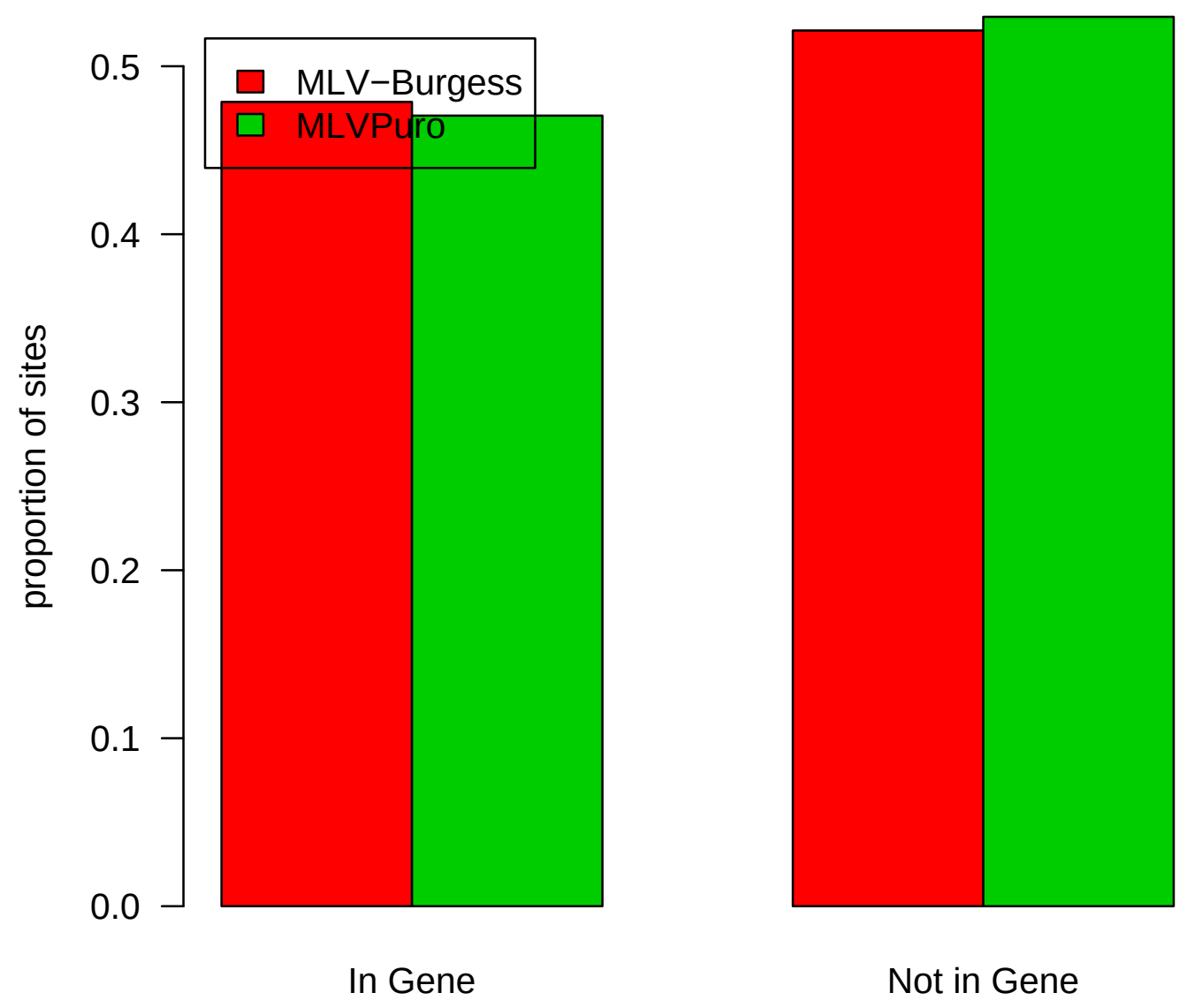

Is there is a tendency for insertions to occur in genes? A formal test of significance yields a p-value of 0.76307.

In the following plot we show the relative frequency of insertions in exons according to the 'uniGene' annotation.

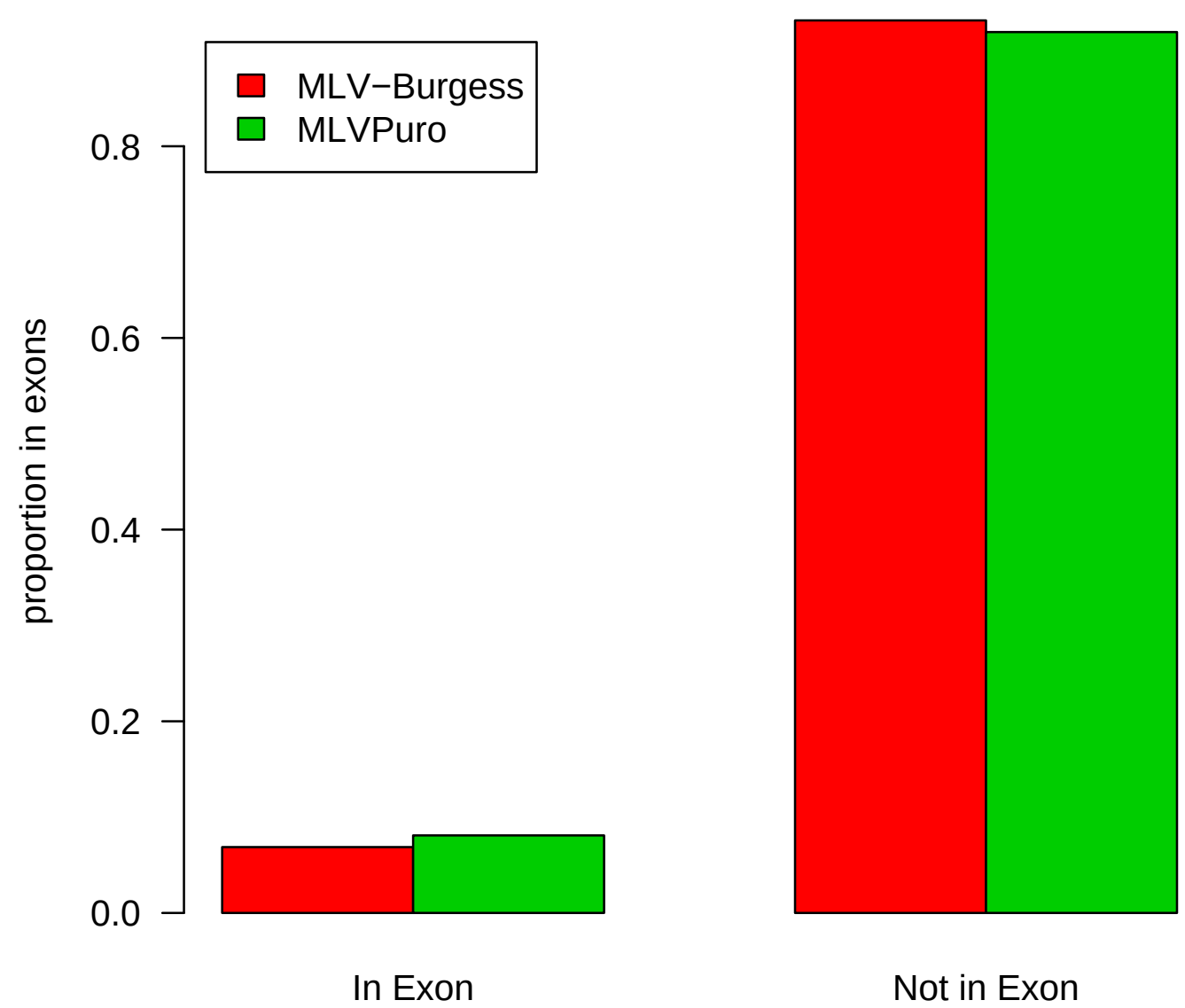

Here is the table of coefficients of the log ratio of intensities along with their standard errors, z statistics, and p-values:

	coef	se	z	p
(Intercept)	-0.5070	0.0746	-6.790	1.11e-11
in.gene	-0.0664	0.1140	-0.583	5.60e-01
in.exon	0.2140	0.2140	0.998	3.18e-01

The model on which these coefficients are based include terms for whether the site is in a gene or not. Thus, the effect shown as 'in.exon' is net of that due to being in a gene. Note that in the barplot above the 'Not in Exon' bars include the both introns and intergenic regions, so the impression given by the table may differ from that for the barplot.

3 CpG Island Neighborhoods

Here we study the effect of being in the neighborhood of CpG Islands. Following Wu et al [2], who found that the neighborhoods within ±1kb of CpG islands are enriched for MLV insertions, we study such neighborhoods.

3.1 1 kilobase neighborhoods

The following plot shows the effect of being in or within ±1kb of a CpG island:

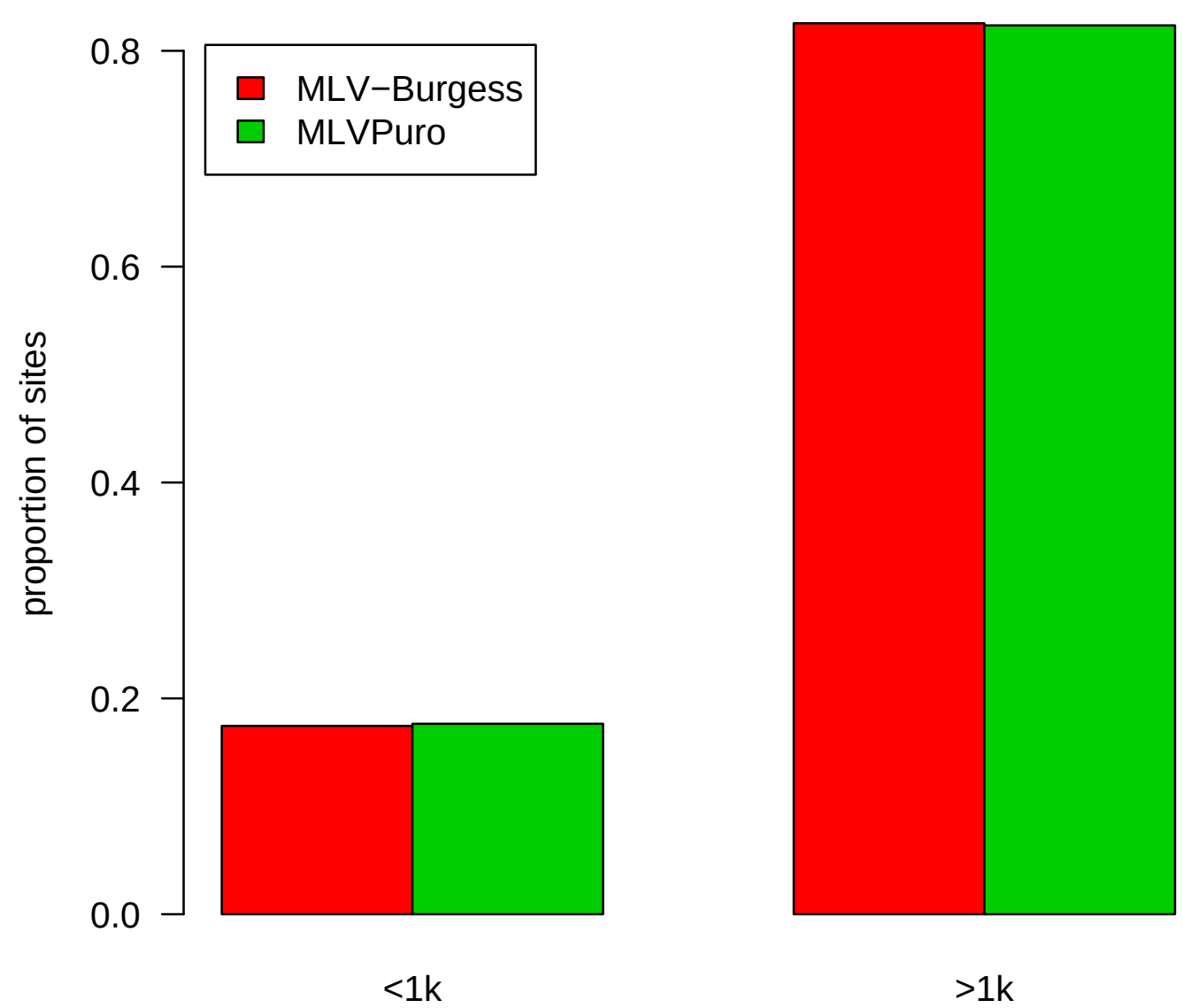

A formal test of significance comparing the difference attains a p-value of 0.92303.

3.2 5 kilobase neighborhoods

The following plot shows the effect of being in or within ±5kb of a CpG island:

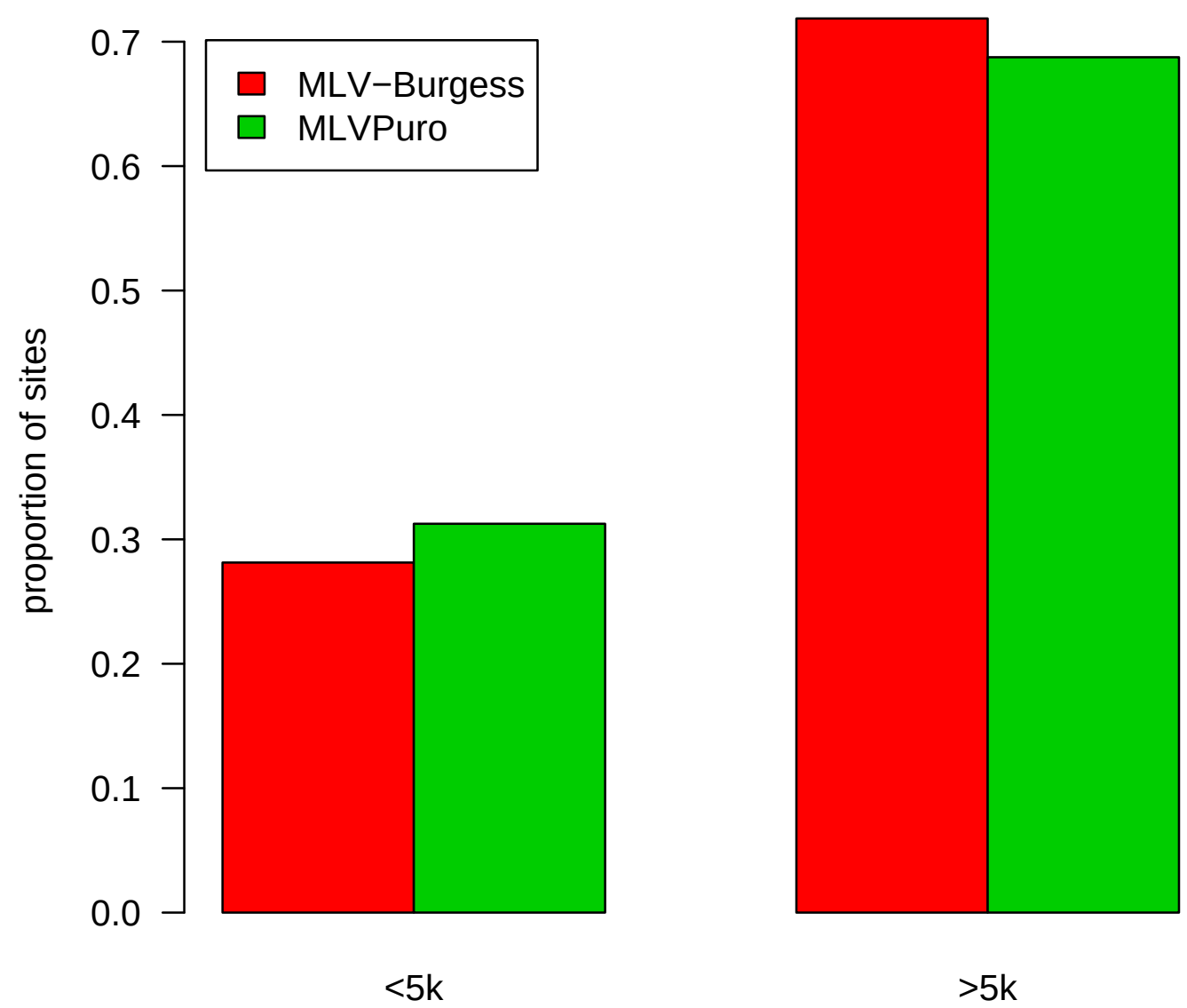

A formal test of significance comparing the difference attains a p-value of 0.20711.

3.3 10 kilobase neighborhoods

The following plot shows the effect of being in or within ±10kb of a CpG island:

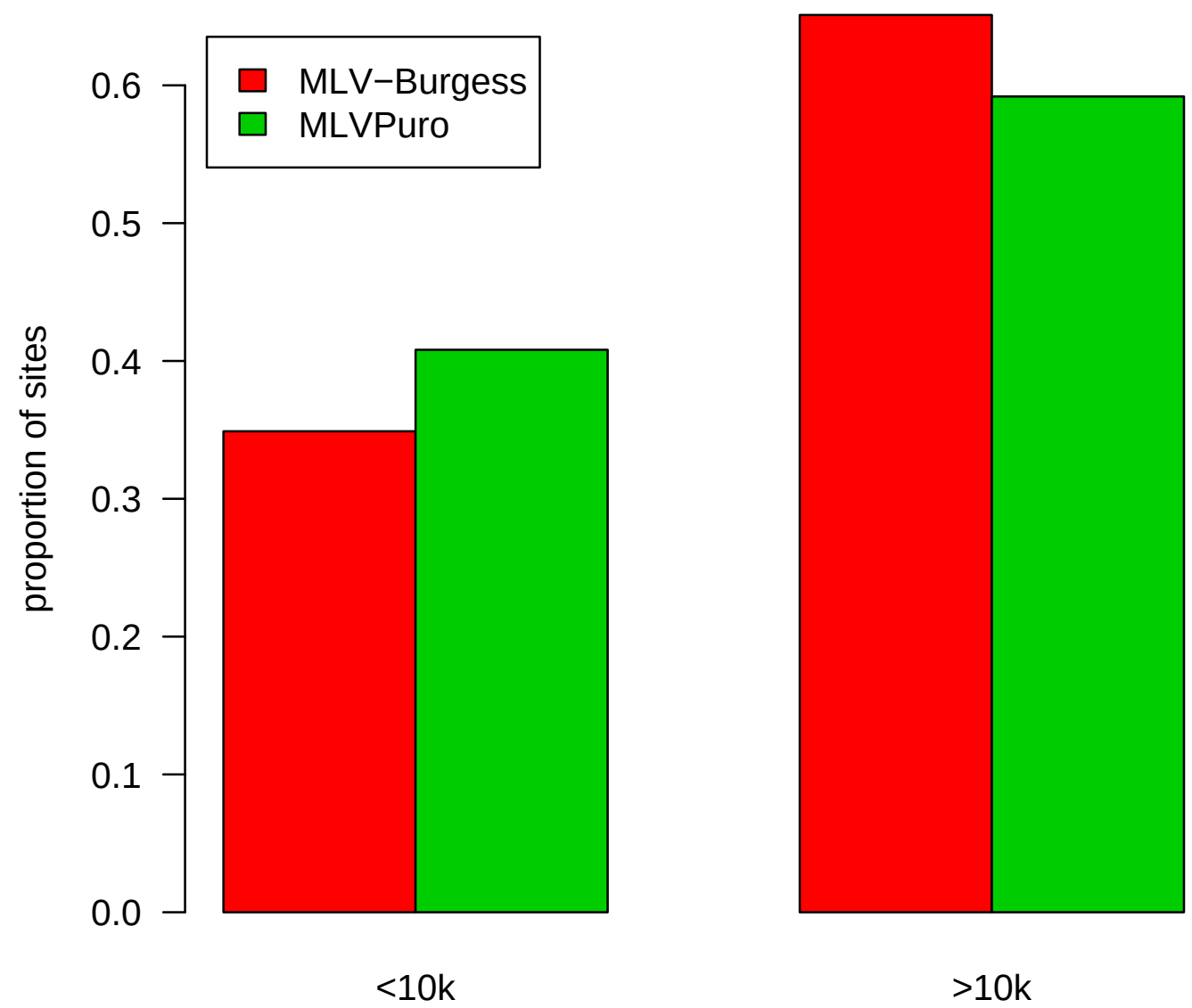

A formal test of significance comparing the difference attains a p-value of 0.024048.

3.4 25 kilobase neighborhoods

The following plot shows the effect of being in or within ±25kb of a CpG island:

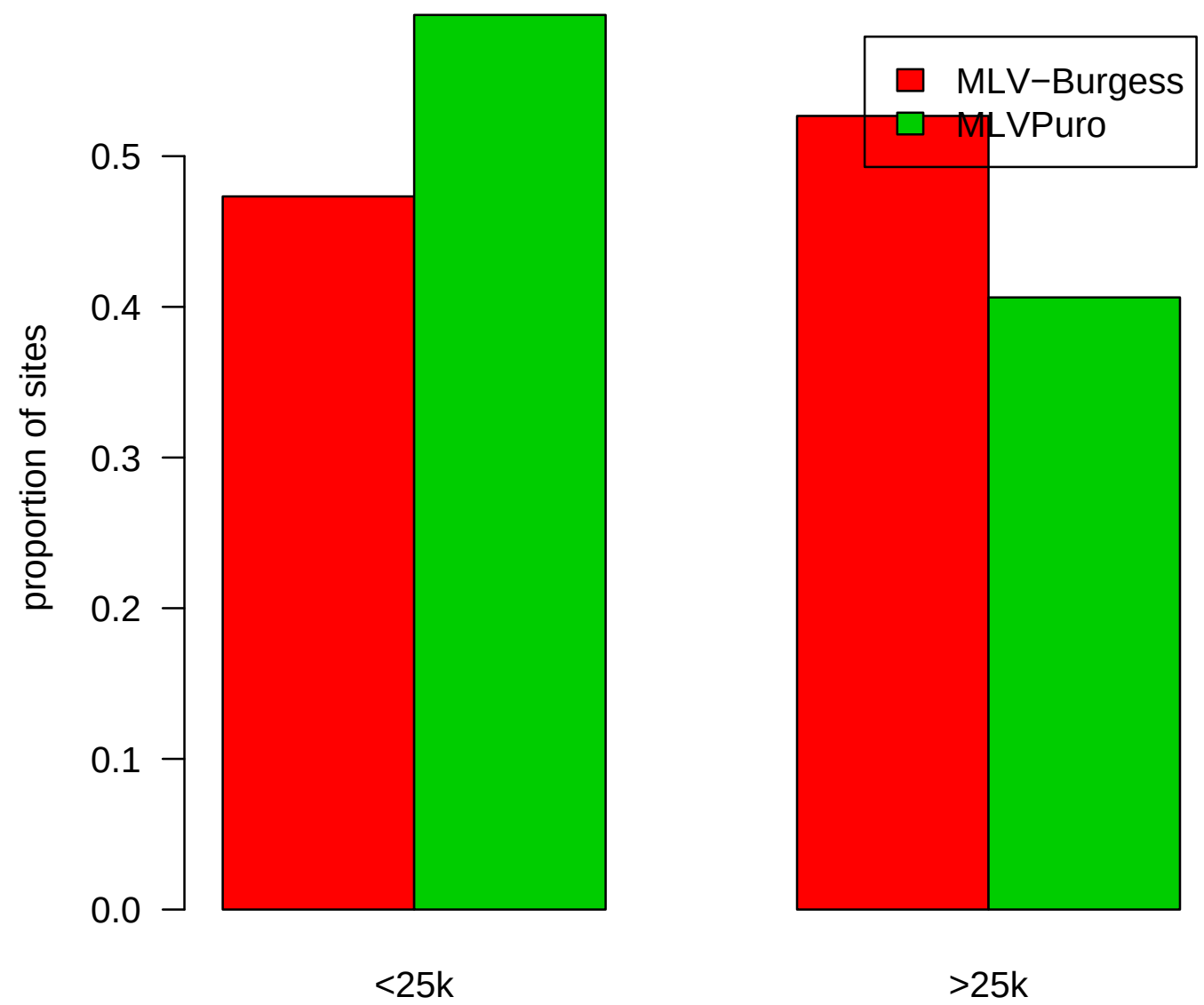

A formal test of significance comparing the difference attains a p-value of $8.0113e-06$.

3.5 50 kilobase neighborhoods

The following plot shows the effect of being in or within ±50kb of a CpG island:

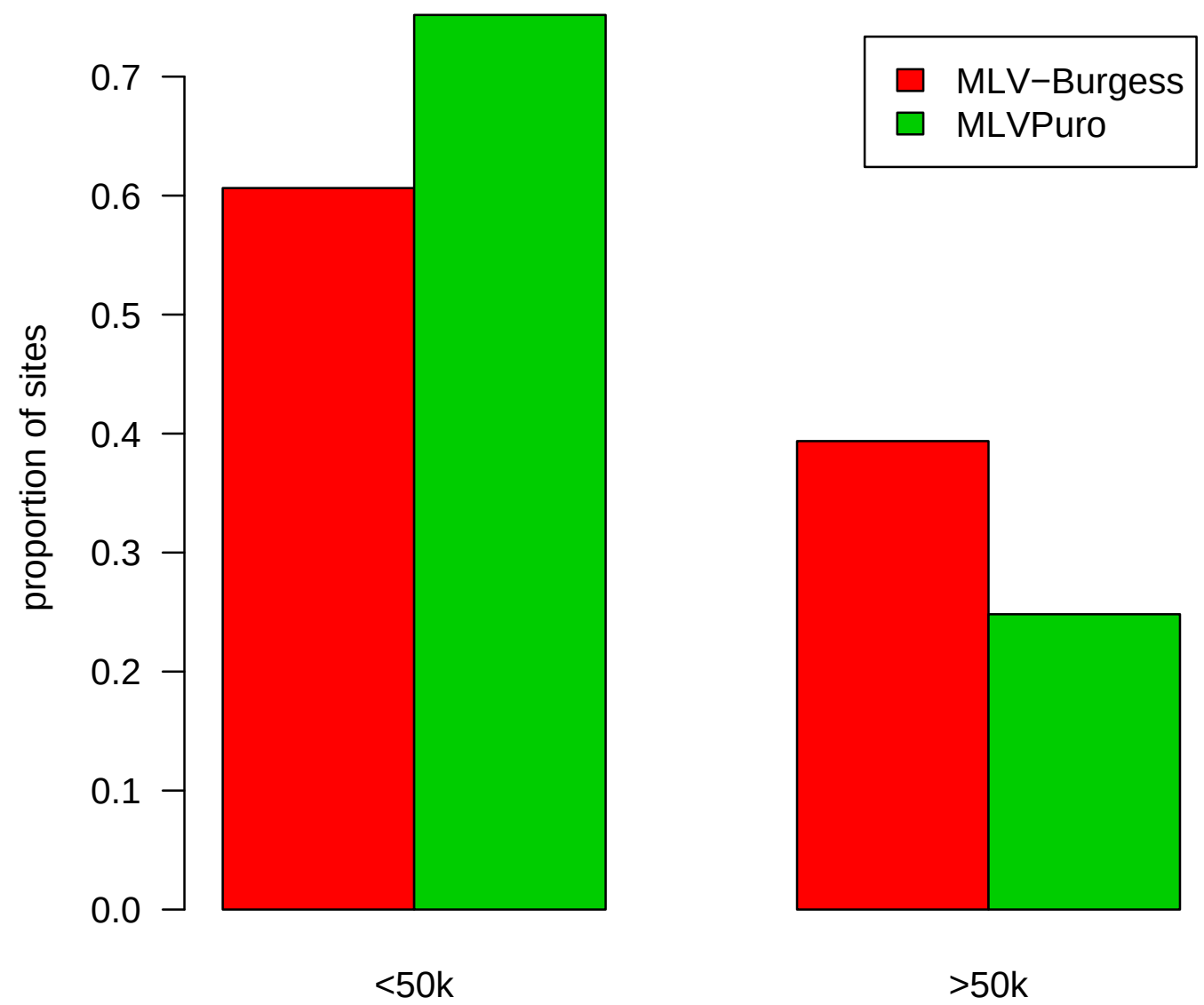

A formal test of significance comparing the difference attains a p-value of $8.9273e-09$.

4 Gene Density, Expression 'Density', and CpG Island Density

In this section the association with gene density is examined. The 'genes' that are counted are the genes represented on the microarray. In addition, we the number of such genes expressed at various levels. The levels are

low.ex Count genes whose expression is in the upper half and divide by number of bases

med.ex Count genes whose expression is in the upper $1/8^{th}$ and divide by number of bases

high.ex Count genes whose expression is in the upper $1/16^{th}$ and divide by number of bases

The bolded terms are used as abbreviations in what follows. The abbreviation **dens** is used to indicate gene density as number of genes per base.

4.1 25 kiloBase Window

In the barplot that follows we examine the association of insertion sites with gene density in a 25 kilobase window surrounding each locus. More such plots will follow and the method of their construction is always to try to divide the data according to the deciles of density. However, it often happens that there is a very skewed distribution of density and often even the 90th percentile is zero. In that case, the barplots simply show the sites for which the density is zero and those for which it is non-zero. If there are fewer than ten groups of bars, then the groupings contain ten percent of the sites each except for the leftmost grouping which will contain all of the remaining sites.

Also note that the title of the plot contains clues as to its content; the prefix indicates the type of variable studied while the suffix indicates the window width in the number of bases. The p-value given is the result of fitting a cubic polynomial to the gene density values.

The following expression data and probe set were used for this report:

```
[1] "HeLa_exp_data-HU133a"
```

```
[1] "HG-U133"
```

```
 Category limits

  lower category   upper
1 0e+00  group.1 8.0e-06
2 8e-06  group.2 4.0e-05
3 4e-05  group.3 2.4e-04
```

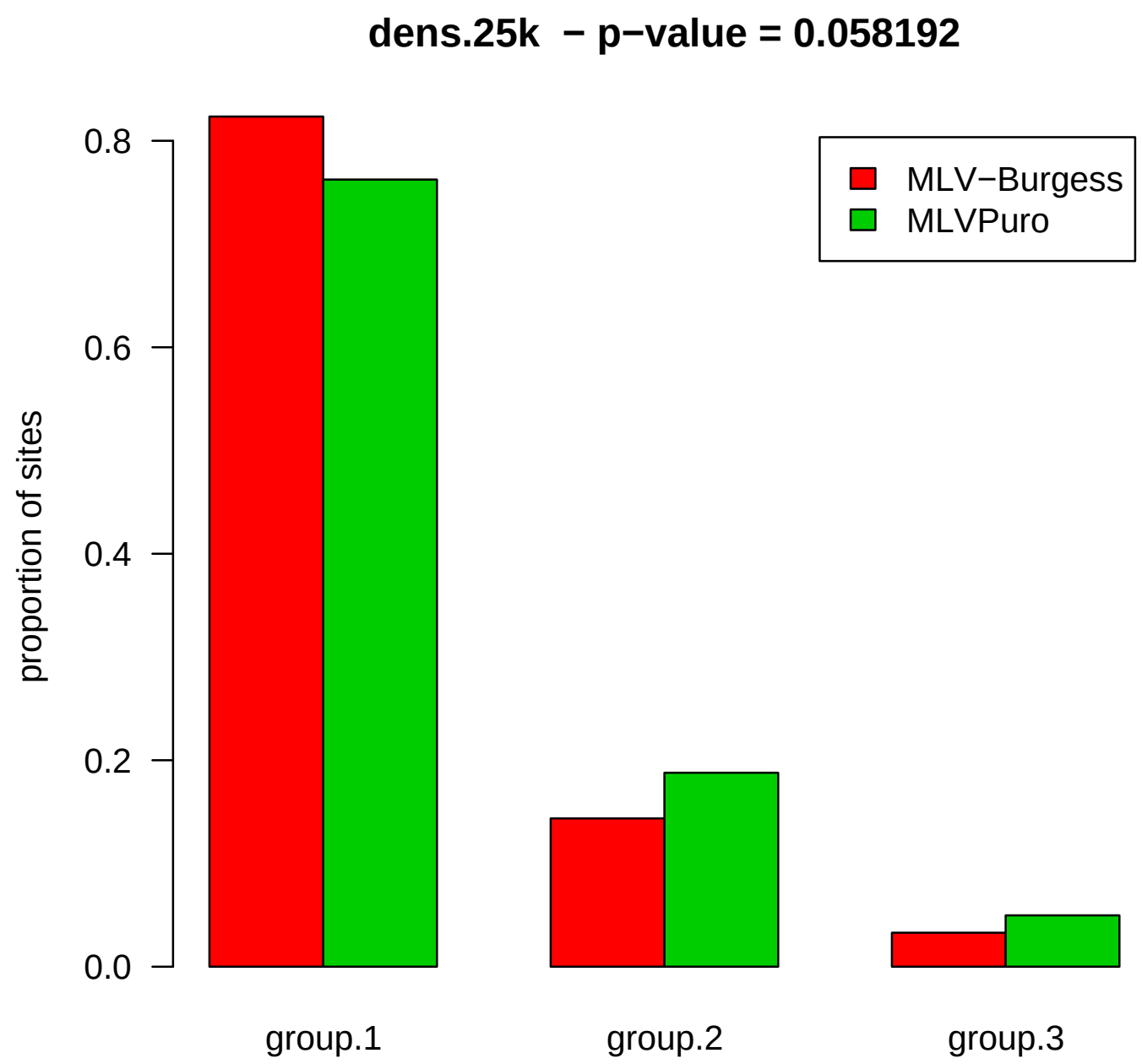

dens.25k − p−value = 0.058192
MLV−Burgess
MLVPuro
proportion of sites
0.0
0.2
0.4
0.6
0.8
group.1
group.2
group.3

Here are the results for expression density. First, we count just genes that are in the upper half.

```
 Category limits

  lower category   upper
1 0e+00   group.1 0.00002
2 2e-05   group.2 0.00014
```

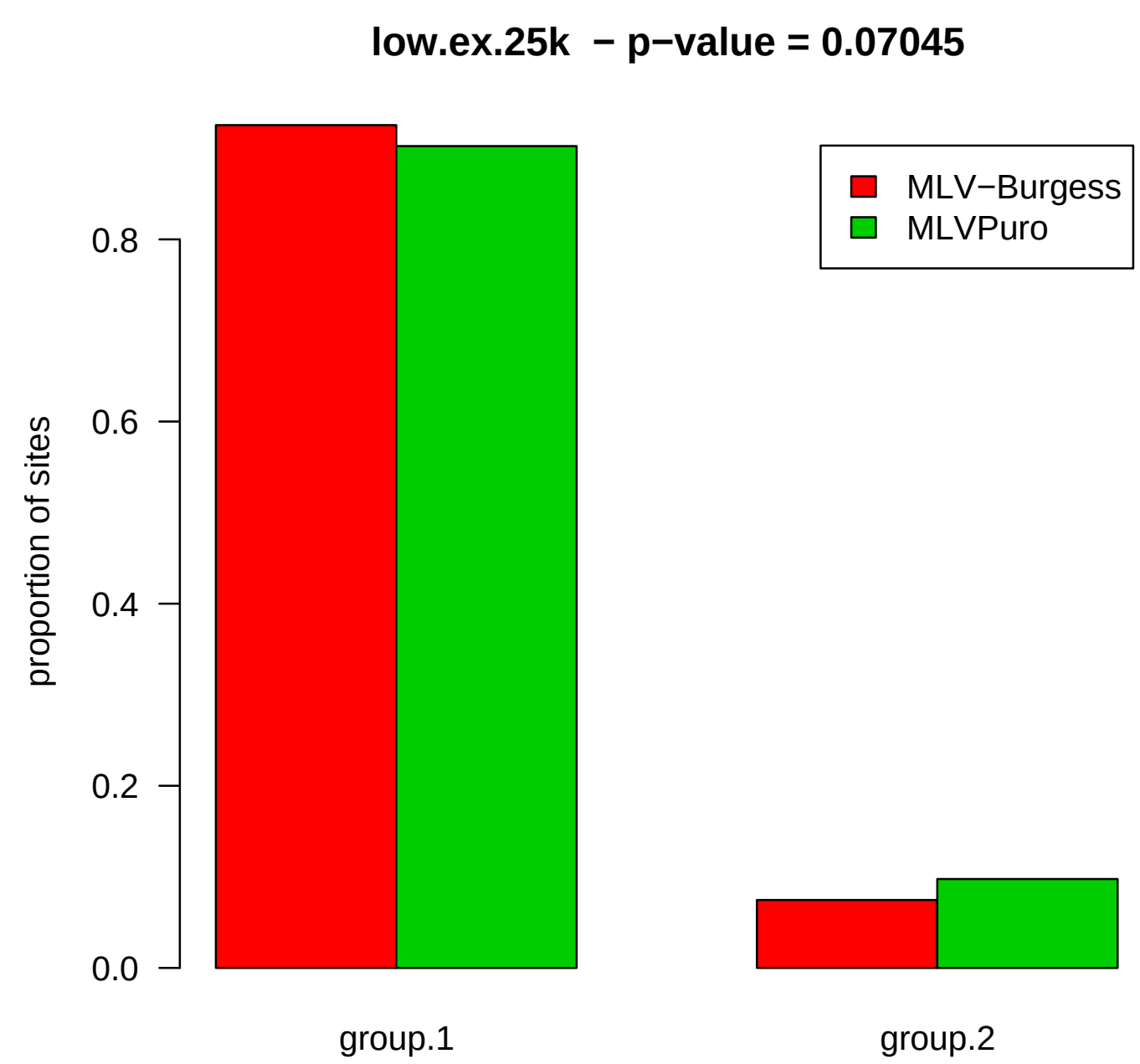

Now we count genes in the upper $1/8^{th}$:

```
Category limits

            lower category        upper
0%   0.000000e+00  group.1 6.666667e-06
100% 6.666667e-06  group.2 1.400000e-04
```

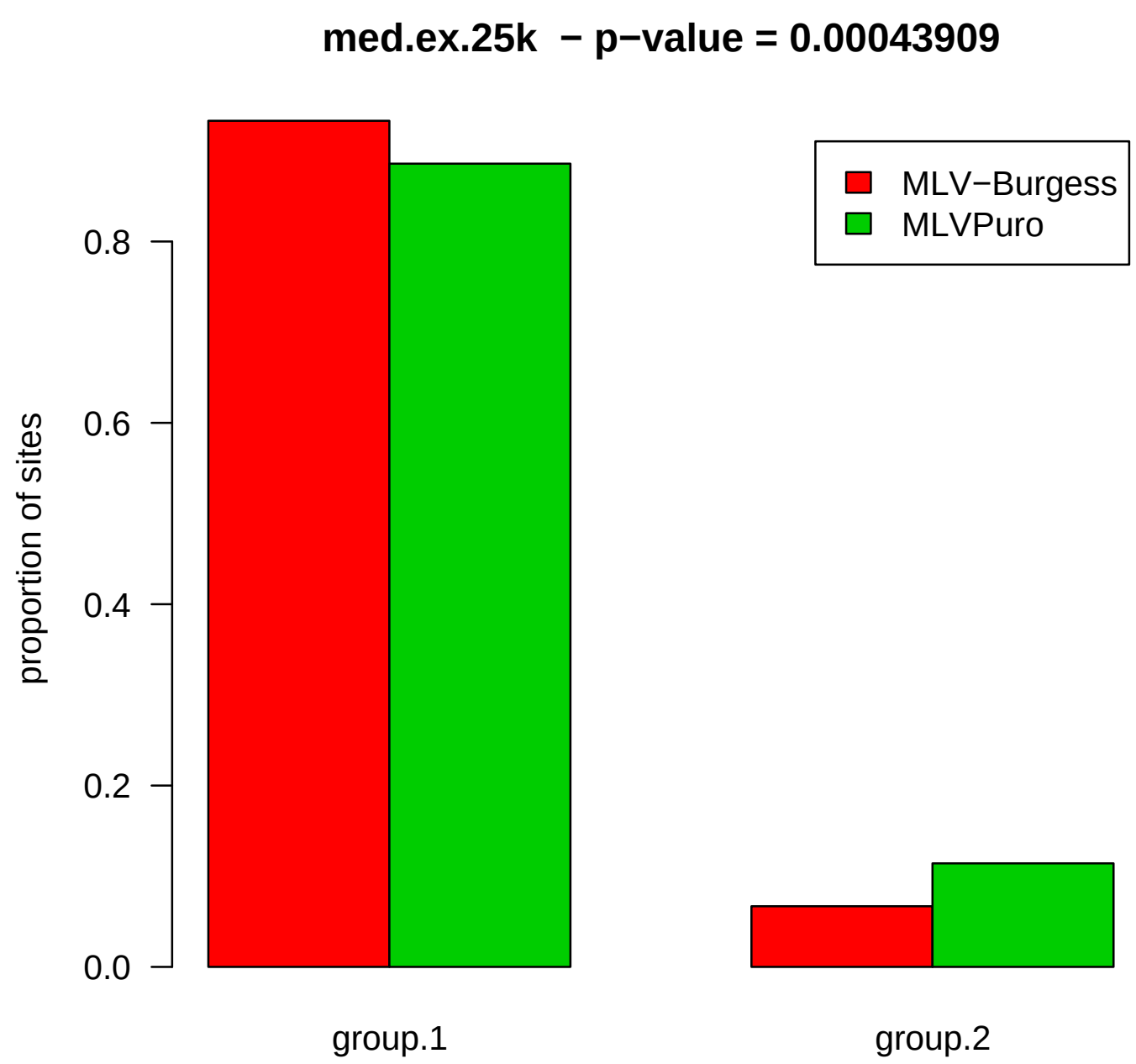

And here we count genes in the upper $1/16^{th}$:

```
Category limits

             lower category        upper
0%    0.000000e+00  group.1 6.666667e-06
100% 6.666667e-06  group.2 1.000000e-04
```

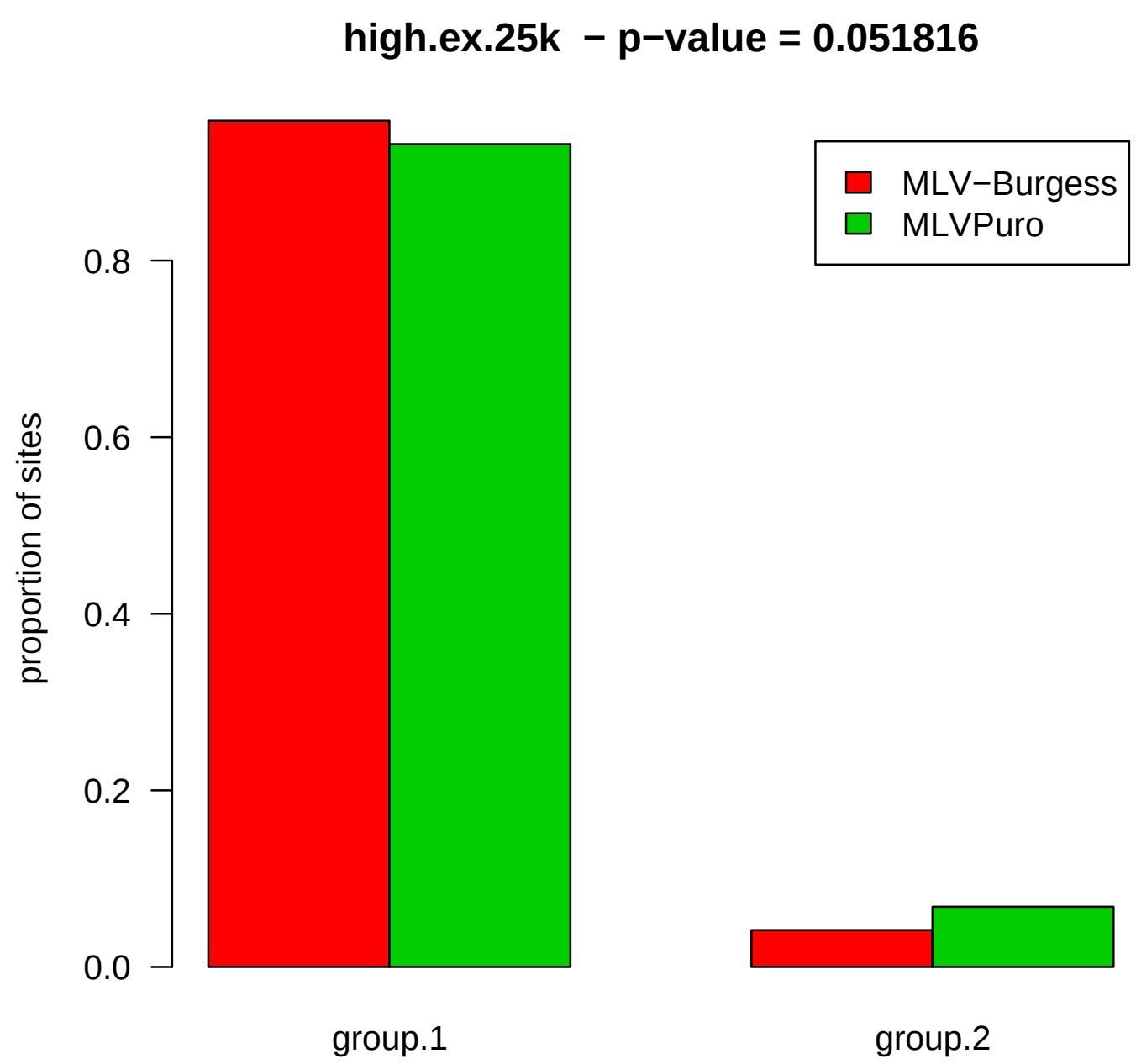

Here the effect of density of CpG islands is studied:

```
Category limits

  lower category   upper
1 0e+00  group.1 0.00002
2 2e-05  group.2 0.00004
3 4e-05  group.3 0.00006
4 6e-05  group.4 0.00026
```

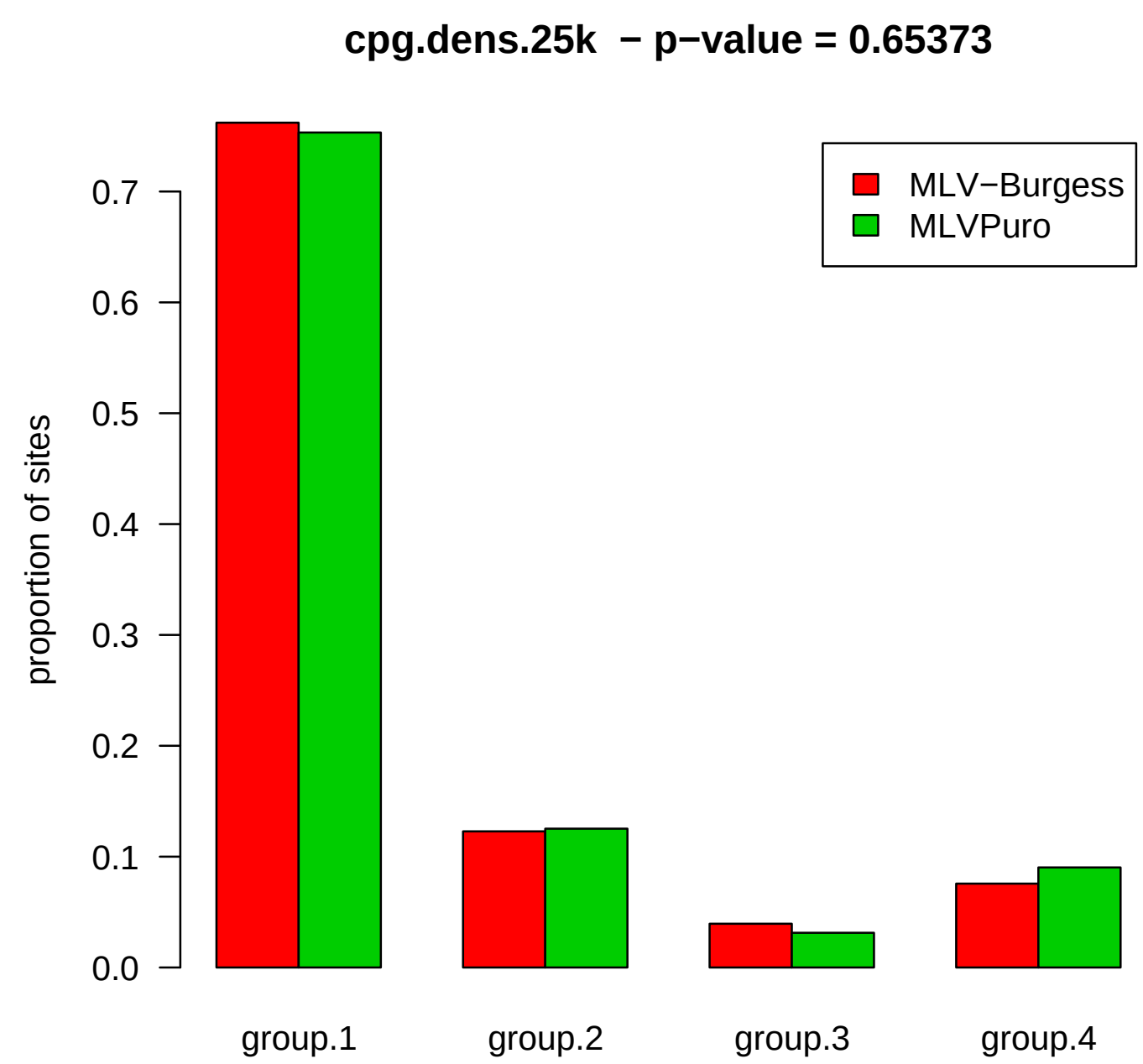

4.2 50 kiloBase Window

In the barplot that follows we examine the association of insertion sites with expression density in a 50 kilobase window surrounding each locus. First, we count just the number of genes on the represented on the chip.

```
 Category limits

  lower category   upper
1 0e+00  group.1 0.00001
2 1e-05  group.2 0.00002
3 2e-05  group.3 0.00002
4 2e-05  group.4 0.00016
```

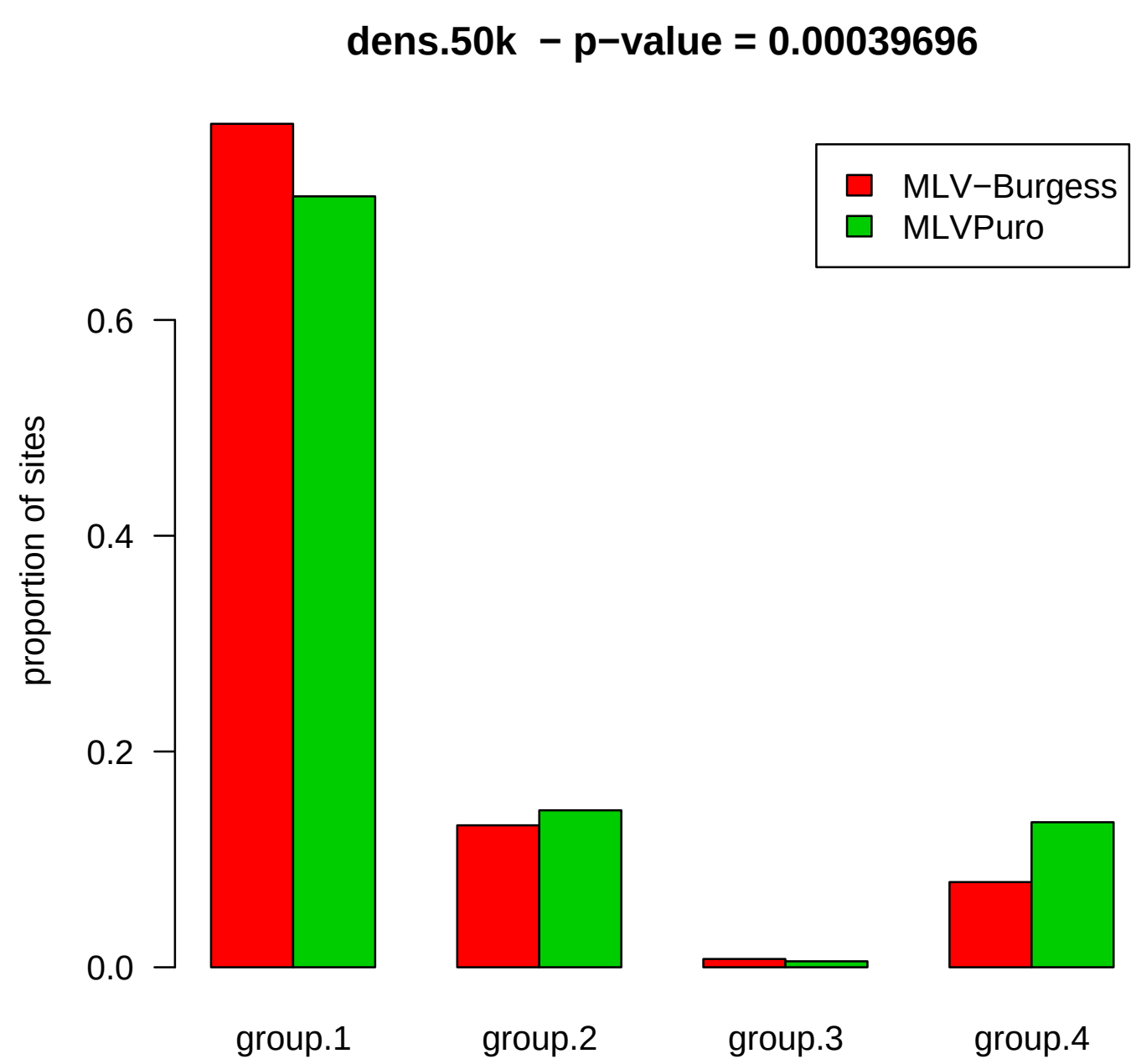

Here are the results for expression density. First, we count just genes that are in the upper half.

```
 Category limits

  lower category upper
1 0e+00  group.1 1e-05
2 1e-05  group.2 2e-05
3 2e-05  group.3 9e-05
```

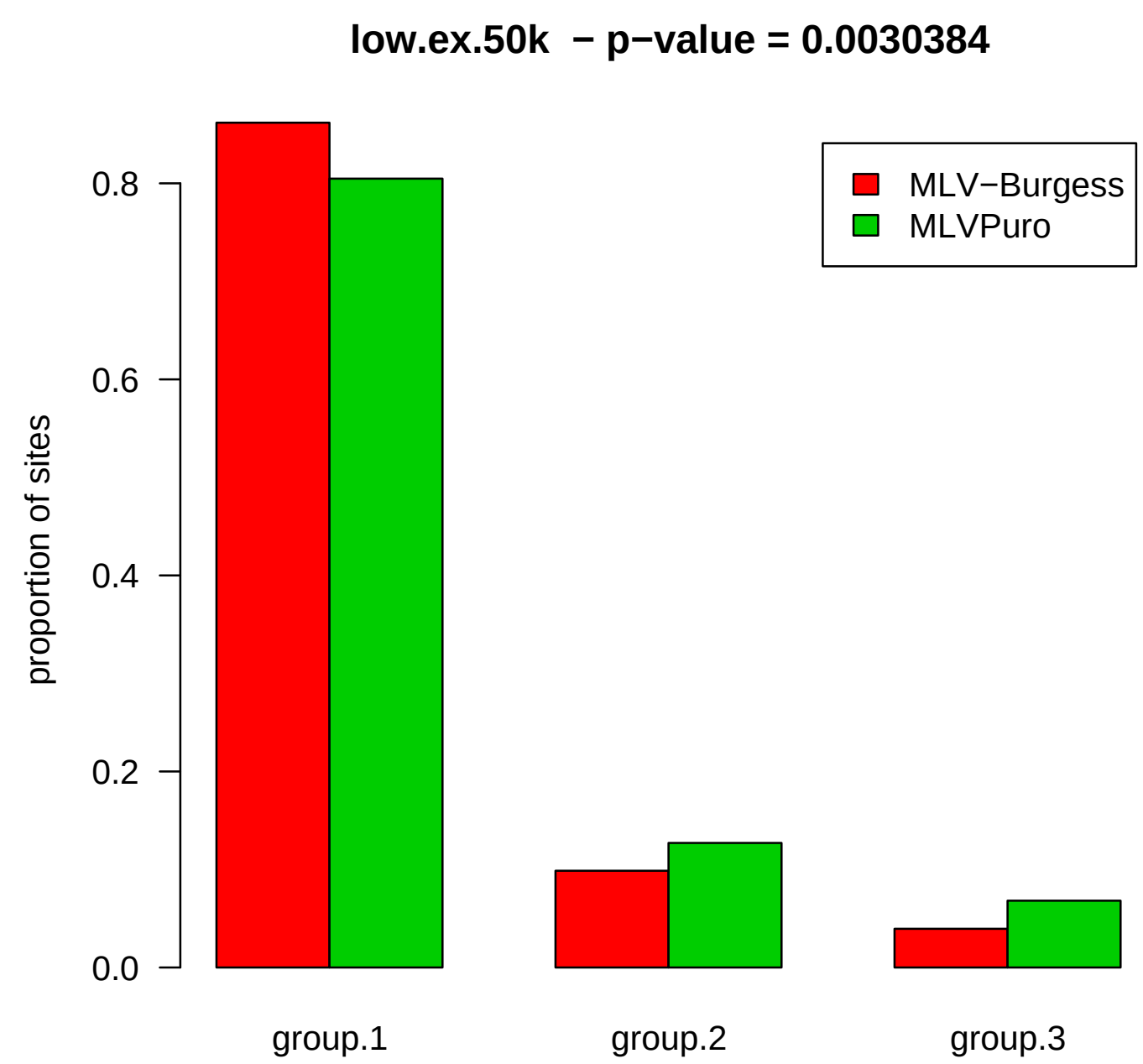

Now we count genes in the upper $1/8^{th}$:

```
Category limits

  lower category upper
1 0e+00  group.1 1e-05
2 1e-05  group.2 7e-05
```

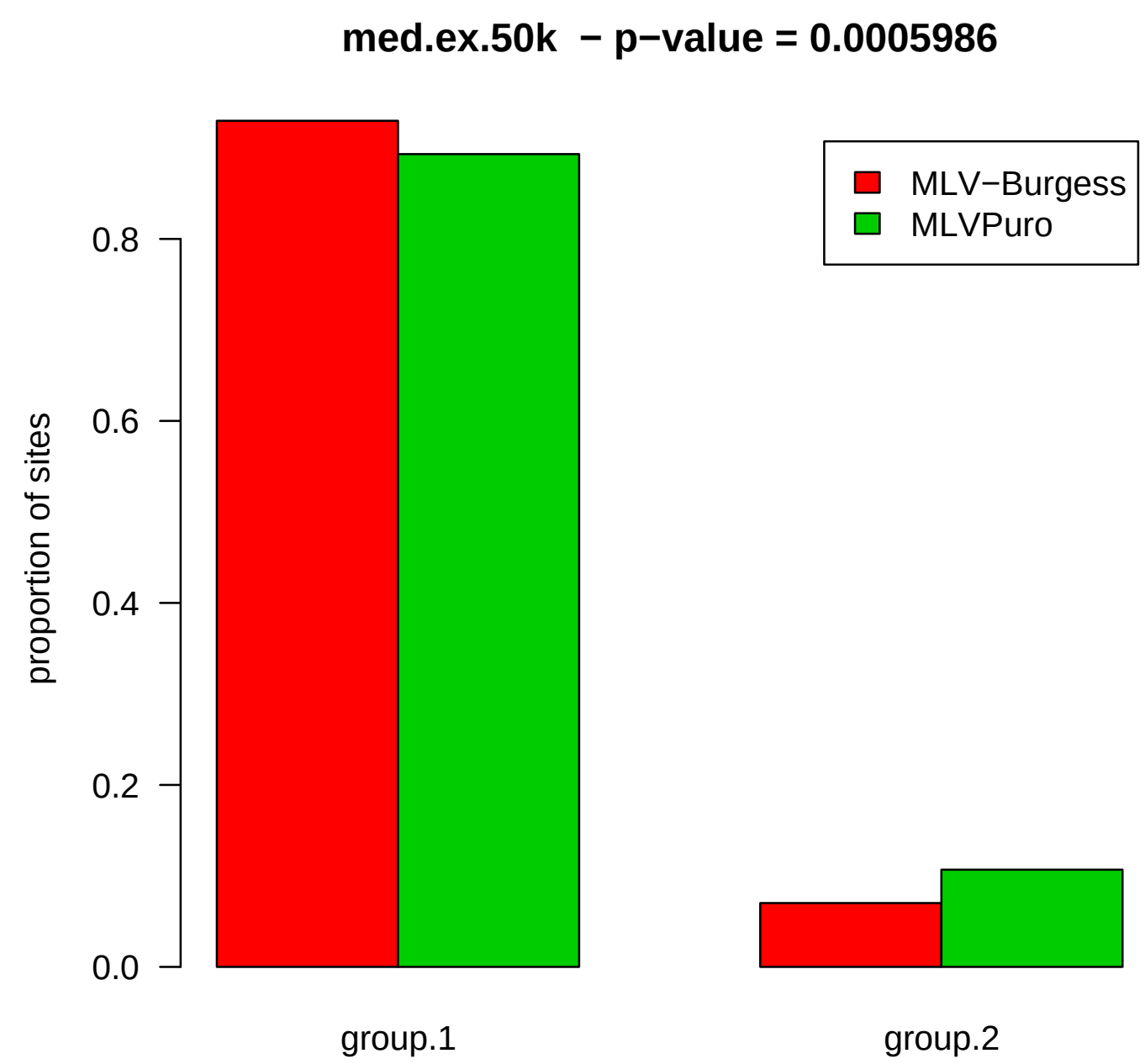

And here we count genes in the upper $1/16^{th}$:

```
Category limits

      lower category   upper
0%   0.0e+00  group.1 2.5e-06
100% 2.5e-06  group.2 5.0e-05
```

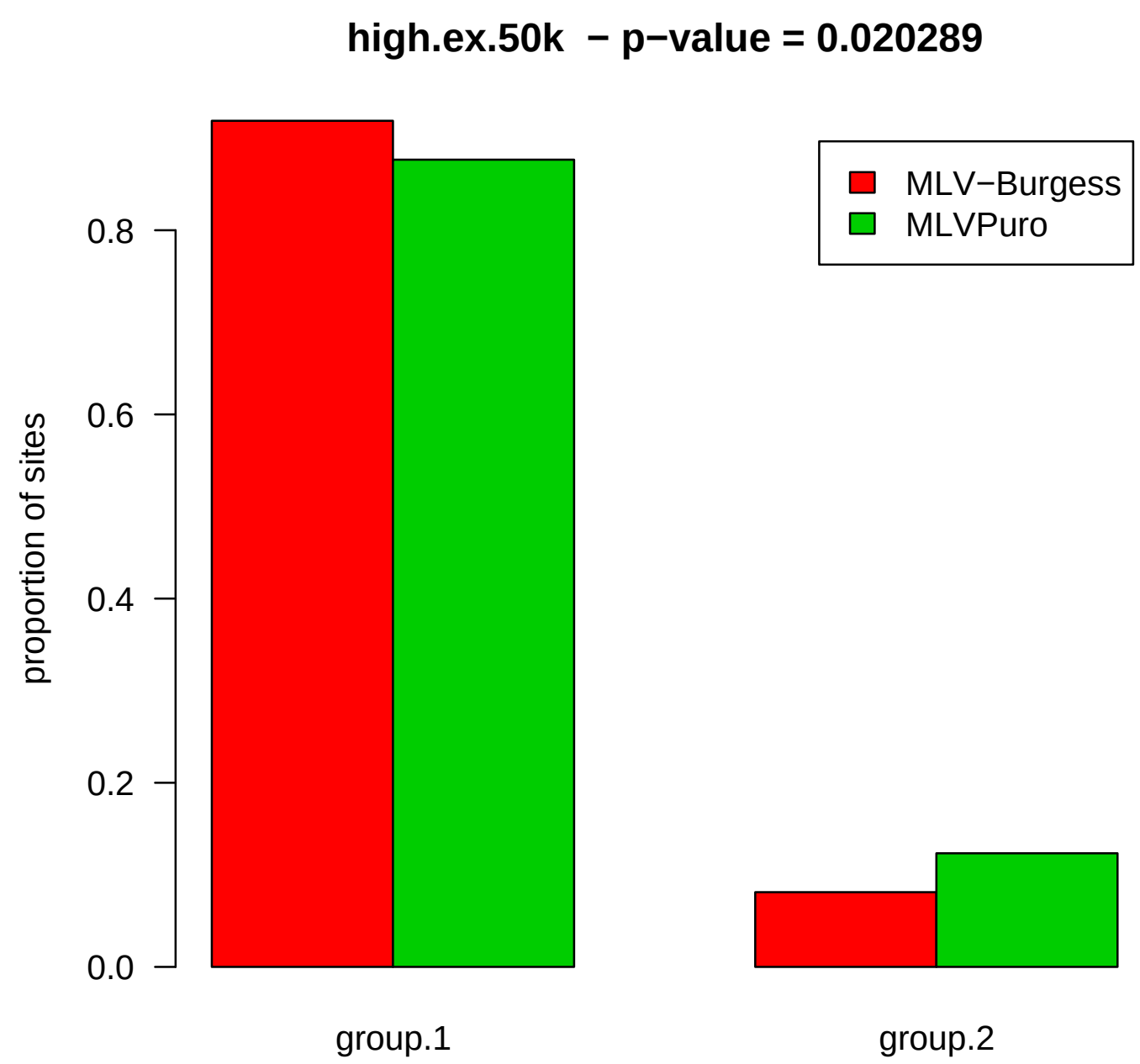

Here the effect of density of CpG islands is studied:

```
Category limits

  lower category   upper
1 0e+00  group.1 0.00001
2 1e-05  group.2 0.00002
3 2e-05  group.3 0.00003
4 3e-05  group.4 0.00005
5 5e-05  group.5 0.00022
```

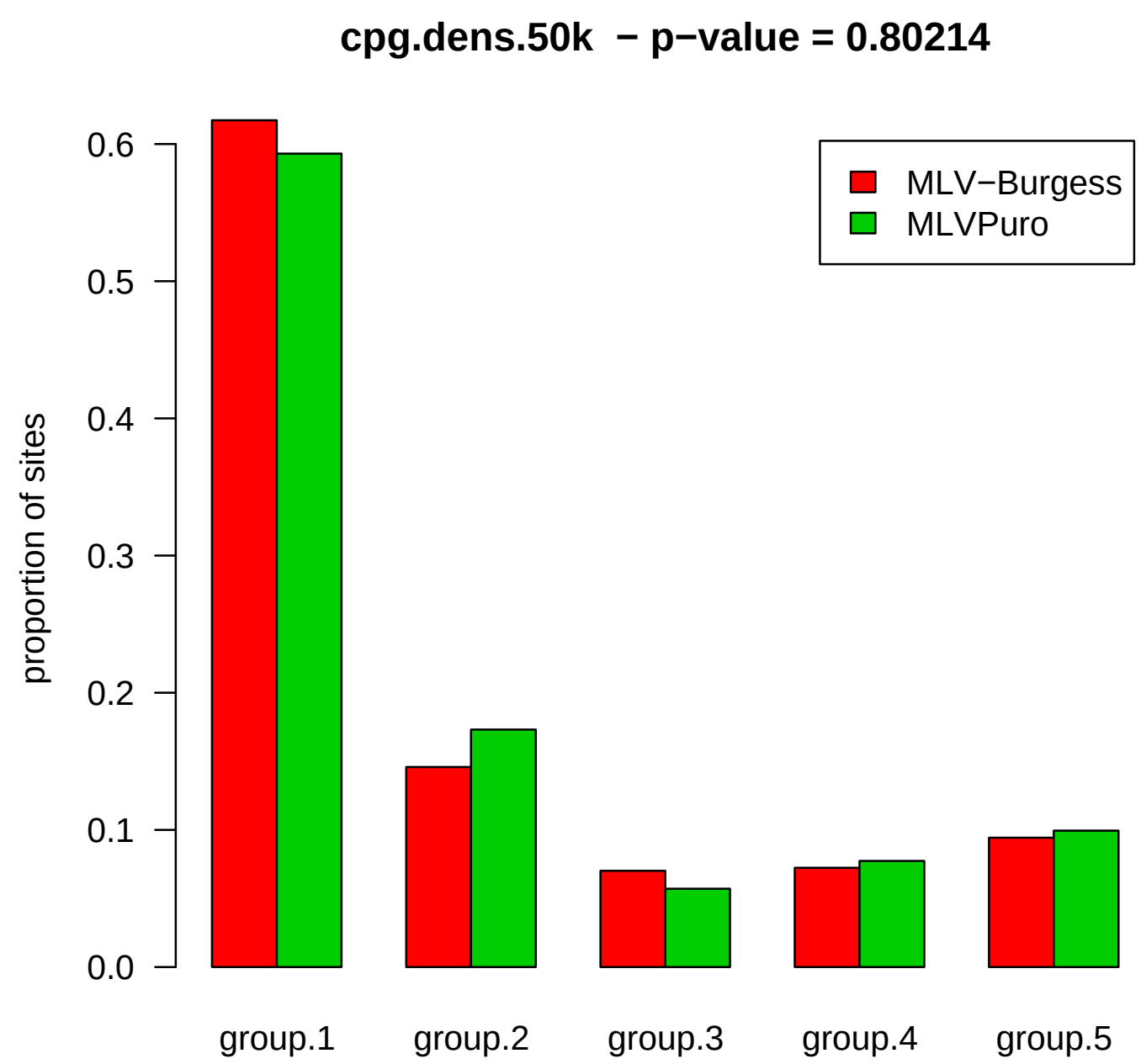

4.3 100 kiloBase Window

In the barplot that follows we examine the association of insertion sites with expression density in a 100 kilobase window surrounding each locus. First, we count just the number of genes on the represented on the chip.

```
 Category limits

         lower category        upper
1 0.000000e+00  group.1 3.333333e-06
2 3.333333e-06  group.2 6.666667e-06
3 6.666667e-06  group.3 1.000000e-05
4 1.000000e-05  group.4 1.346667e-05
5 1.346667e-05  group.5 2.200000e-05
6 2.200000e-05  group.6 1.050000e-04
```

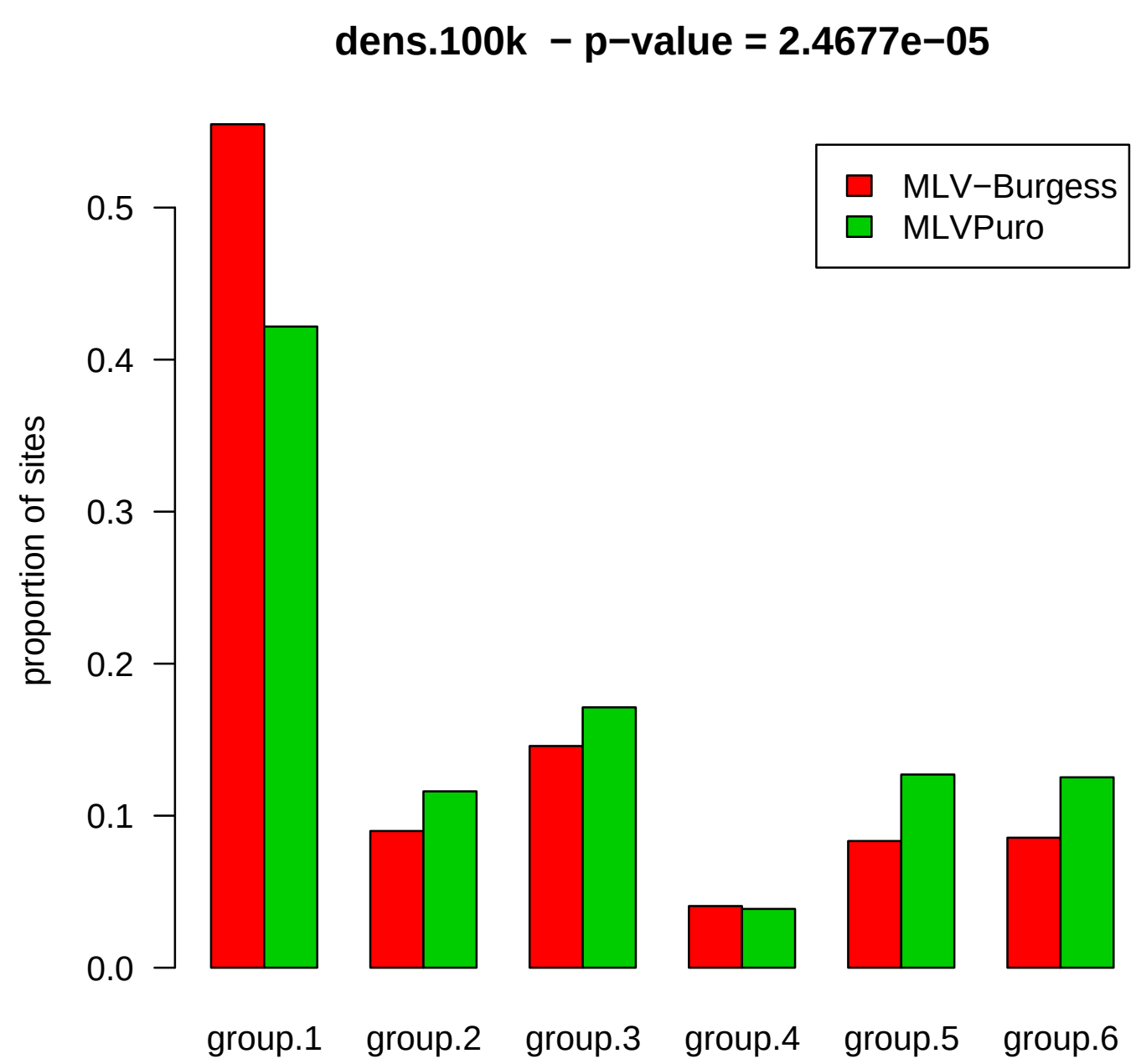

Here are the results for expression density. First, we count just genes that are in the upper half.

```
 Category limits

    lower category   upper
1 0.0e+00  group.1 2.5e-06
2 2.5e-06  group.2 5.0e-06
3 5.0e-06  group.3 1.0e-05
4 1.0e-05  group.4 1.4e-05
5 1.4e-05  group.5 5.5e-05
```

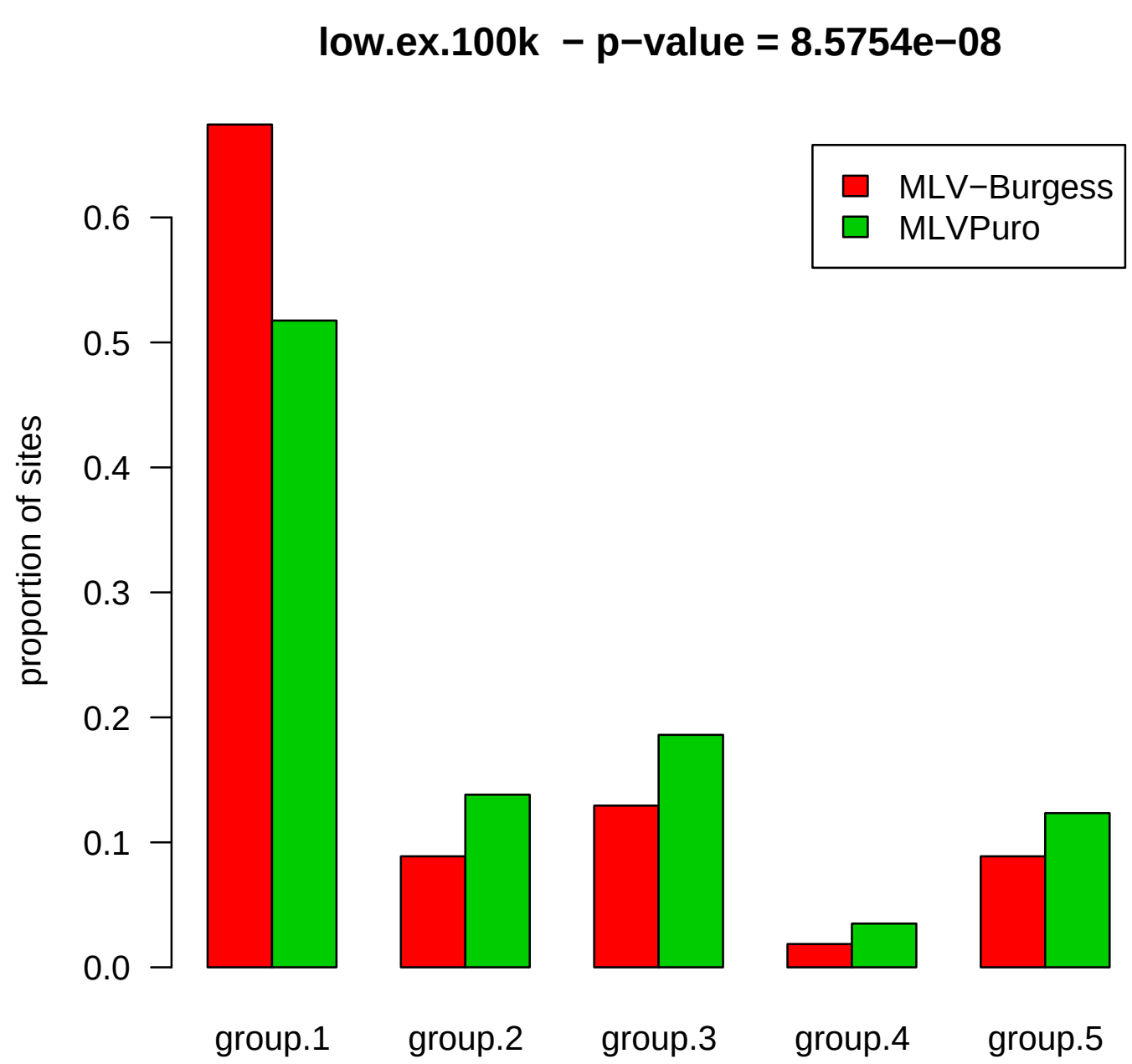

Now we count genes in the upper $1/8^{th}$:

```
Category limits

  lower category upper
1 0e+00  group.1 5e-06
2 5e-06  group.2 1e-05
3 1e-05  group.3 5e-05
```

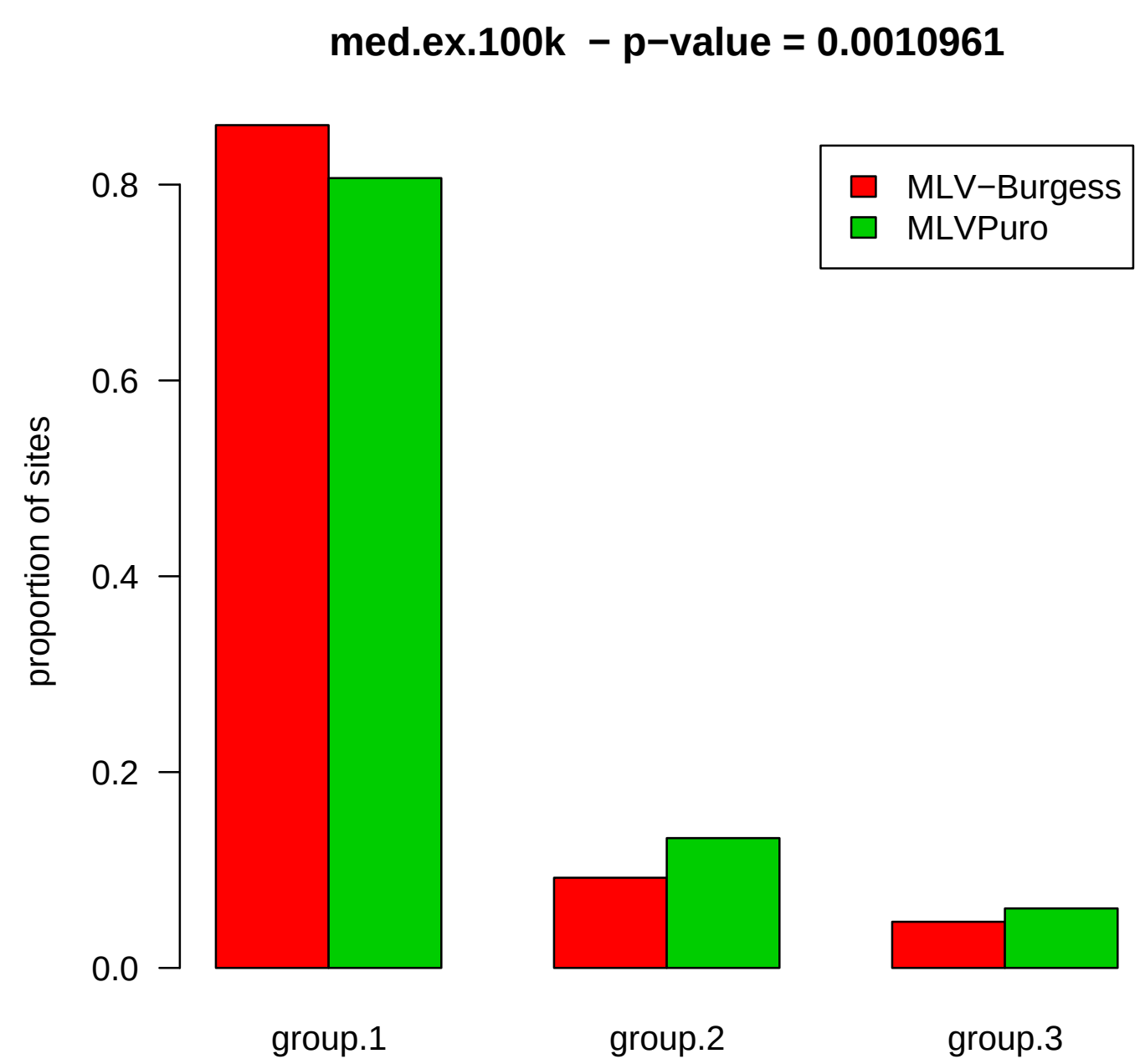

And here we count genes in the upper $1/16^{th}$:

```
Category limits

  lower category    upper
1 0e+00  group.1 5.00e-06
2 5e-06  group.2 3.25e-05
```

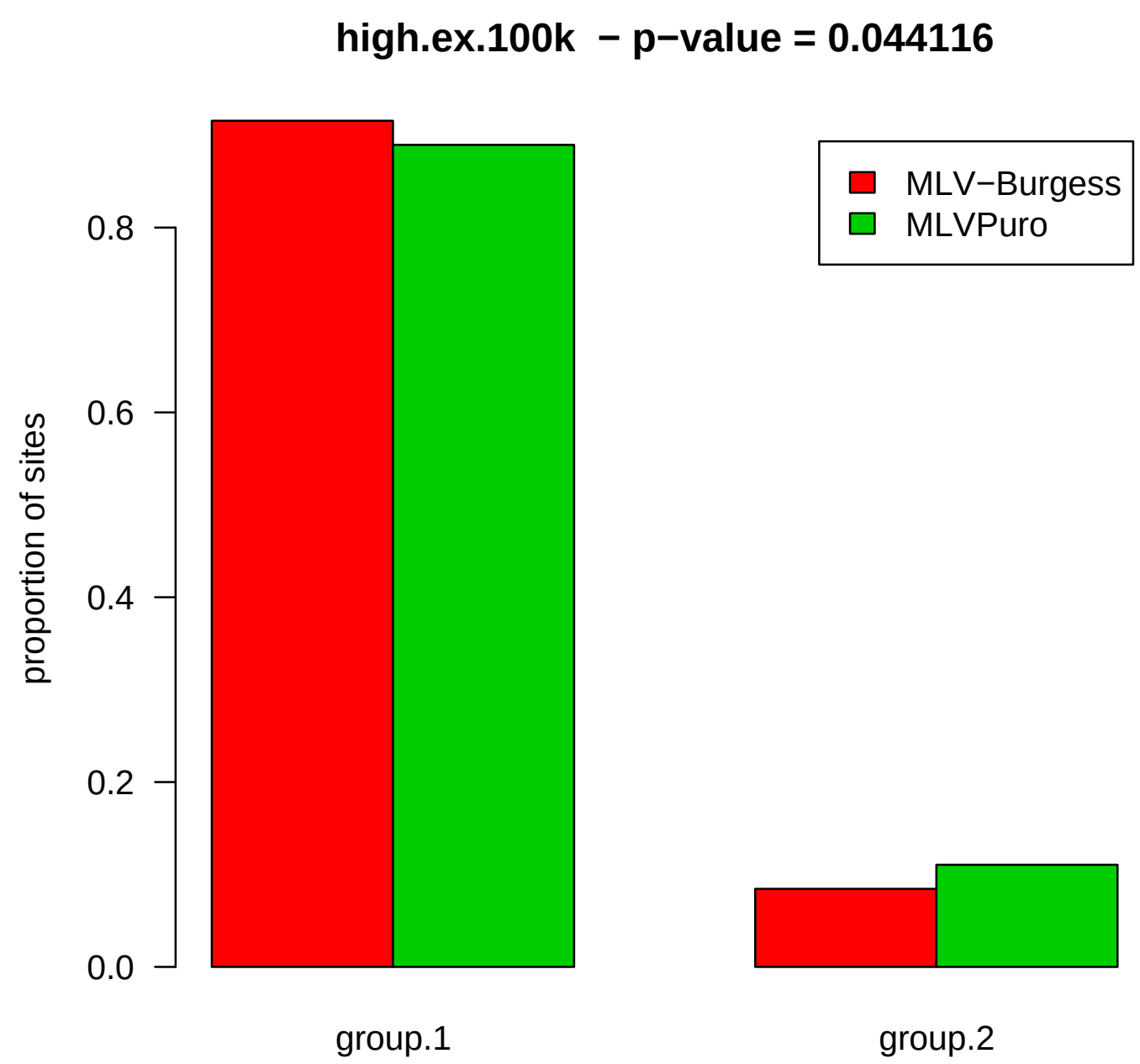

Here the effect of density of CpG islands is studied:

```
Category limits

    lower category    upper
1 0.0e+00  group.1 0.000005
2 5.0e-06  group.2 0.000010
3 1.0e-05  group.3 0.000015
4 1.5e-05  group.4 0.000020
5 2.0e-05  group.5 0.000025
6 2.5e-05  group.6 0.000045
7 4.5e-05  group.7 0.000175
```

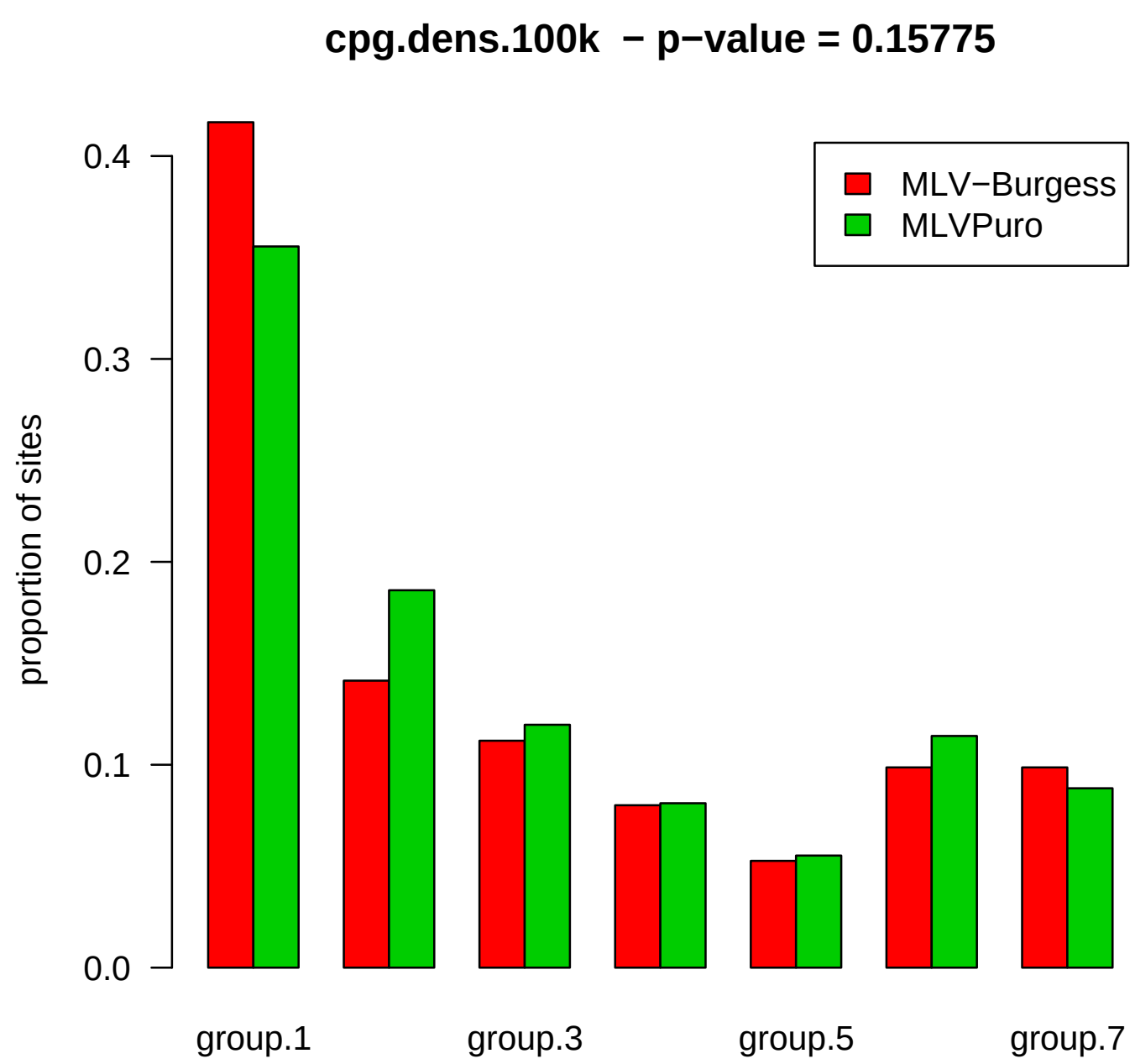

4.4 250 kiloBase Window

In the barplot that follows we examine the association of insertion sites with expression density in a 250 kilobase window surrounding each locus. First, we count just the number of genes on the represented on the chip.

```
 Category limits

         lower category        upper
1 0.000000e+00  group.1 2.000000e-06
2 2.000000e-06  group.2 4.000000e-06
3 4.000000e-06  group.3 4.571429e-06
4 4.571429e-06  group.4 6.666667e-06
5 6.666667e-06  group.5 9.333333e-06
6 9.333333e-06  group.6 1.200000e-05
7 1.200000e-05  group.7 1.866667e-05
8 1.866667e-05  group.8 8.466667e-05
```

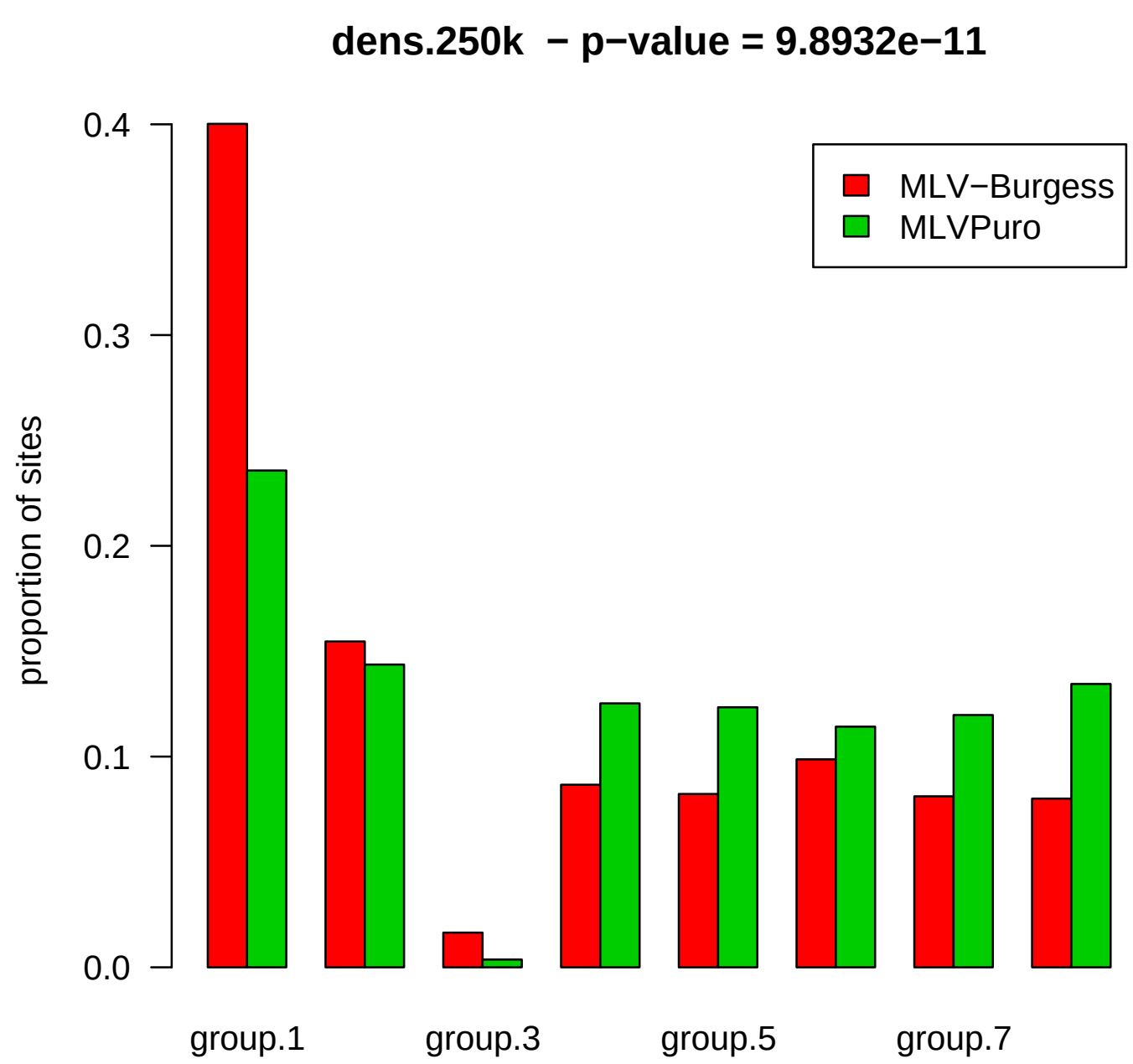

Here are the results for expression density. First, we count just genes that are in the upper half.

```
 Category limits

         lower category        upper
1 0.000000e+00  group.1 1.333333e-06
2 1.333333e-06  group.2 2.000000e-06
3 2.000000e-06  group.3 4.000000e-06
4 4.000000e-06  group.4 5.000000e-06
5 5.000000e-06  group.5 7.000000e-06
6 7.000000e-06  group.6 1.000000e-05
7 1.000000e-05  group.7 4.600000e-05
```

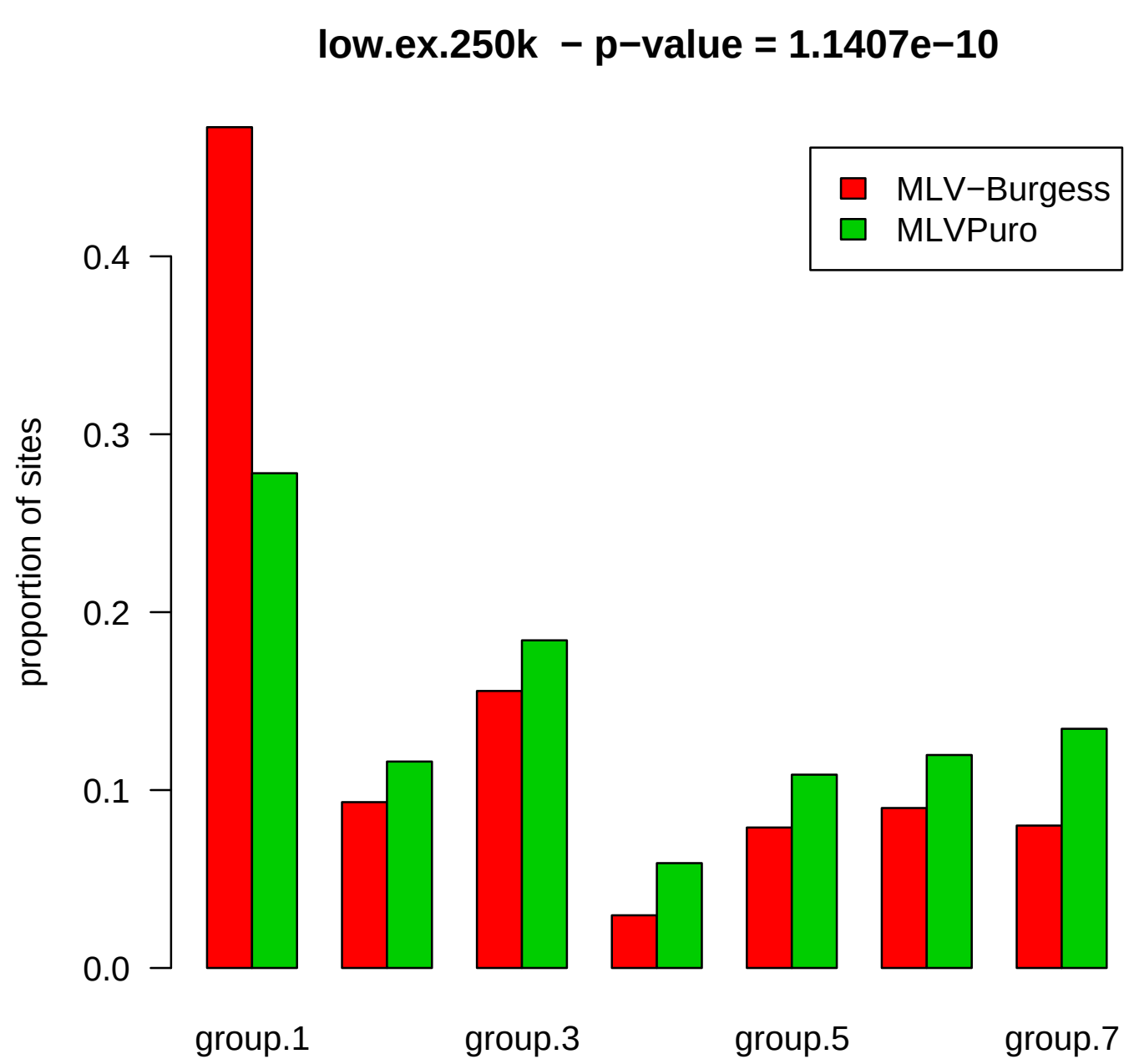

Now we count genes in the upper $1/8^{th}$:

```
Category limits

         lower category        upper
1 0.000000e+00  group.1 1.333333e-06
2 1.333333e-06  group.2 2.666667e-06
3 2.666667e-06  group.3 4.000000e-06
4 4.000000e-06  group.4 6.000000e-06
5 6.000000e-06  group.5 2.733333e-05
```

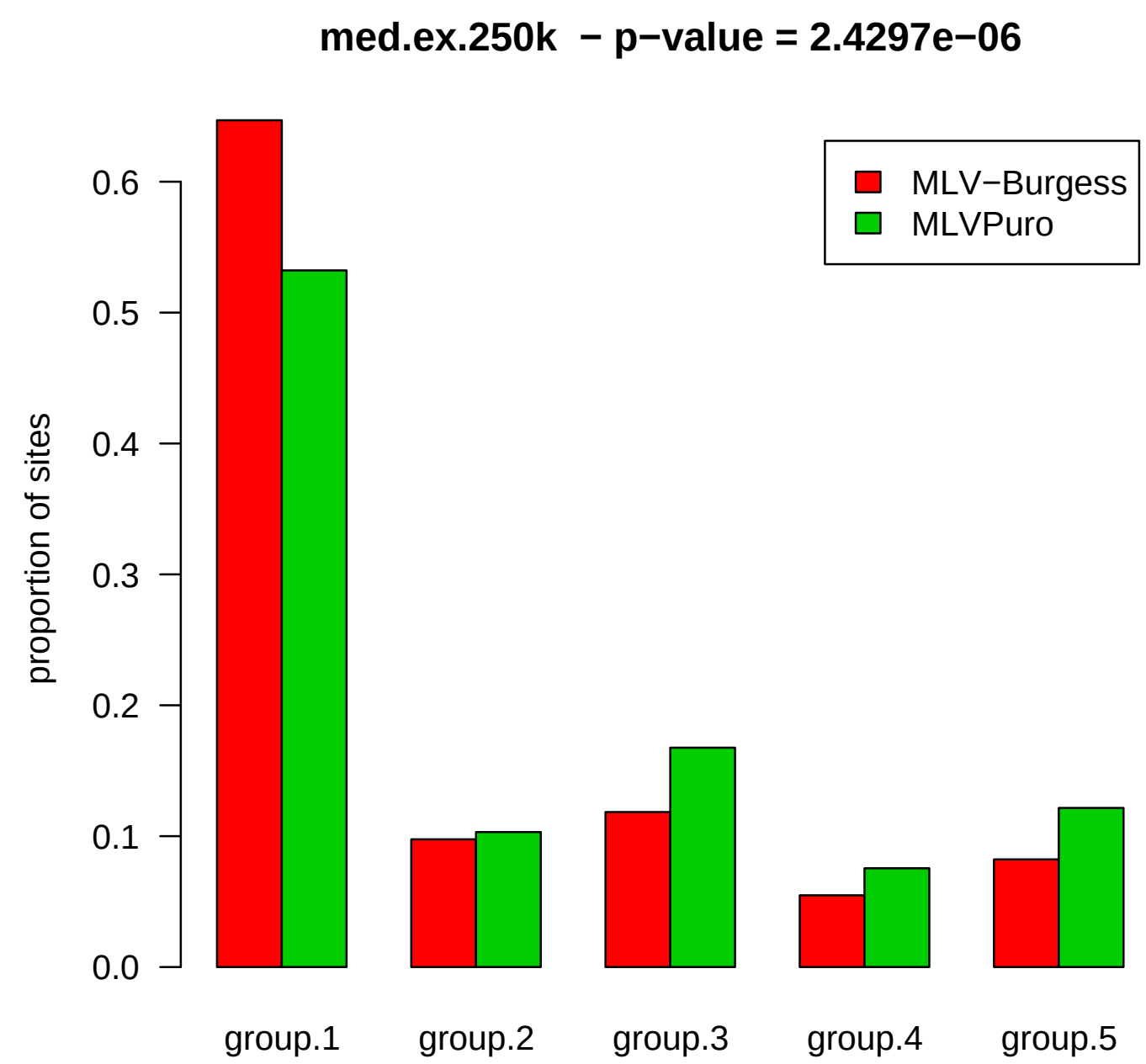

And here we count genes in the upper $1/16^{th}$:

```
Category limits

  lower category upper
1 0e+00  group.1 1e-06
2 1e-06  group.2 2e-06
3 2e-06  group.3 4e-06
4 4e-06  group.4 2e-05
```

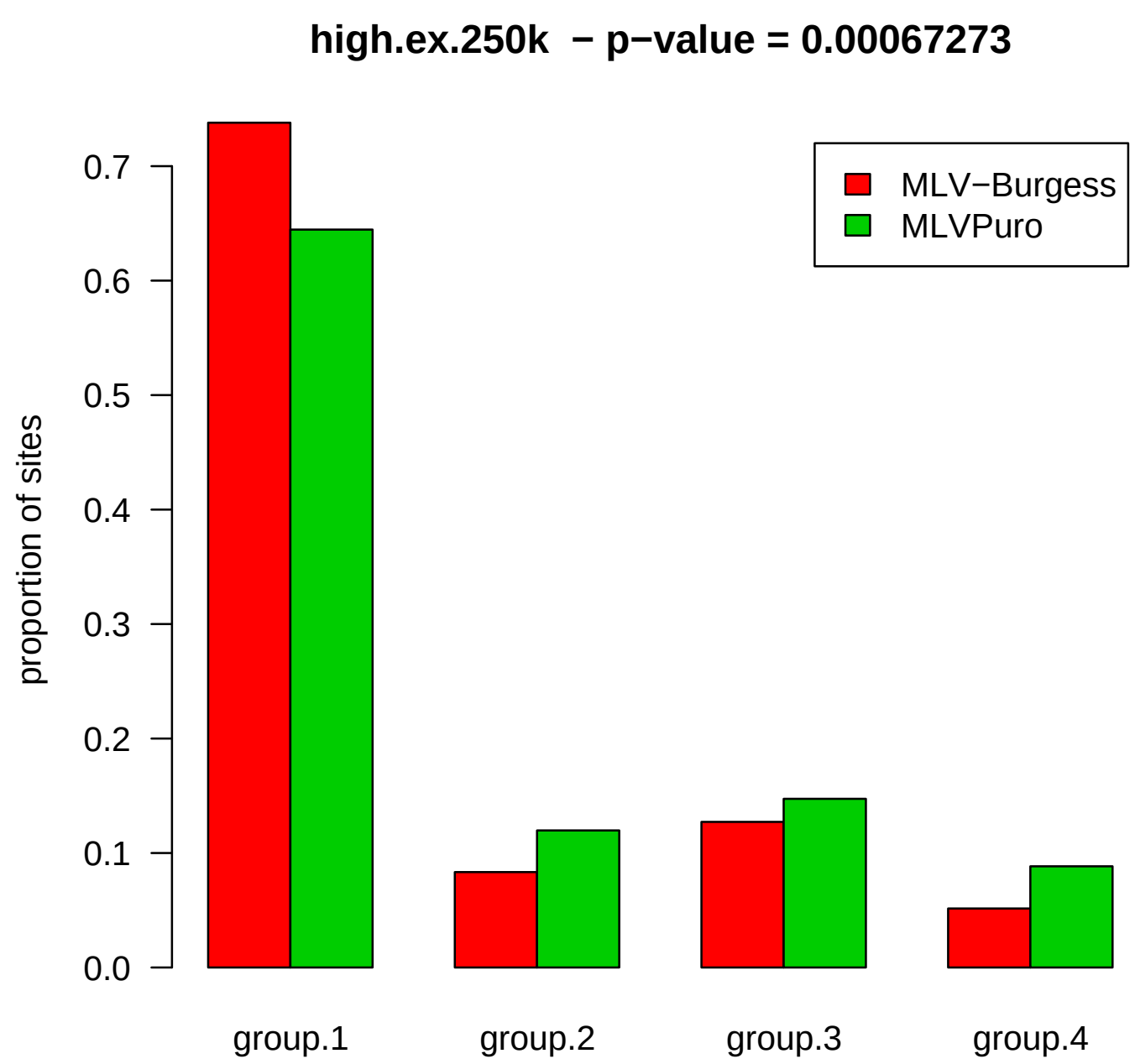

Here the effect of density of CpG islands is studied:

```
Category limits

     lower category    upper
1  0.0e+00  group.1 0.000002
2  2.0e-06  group.2 0.000004
3  4.0e-06  group.3 0.000006
4  6.0e-06  group.4 0.000008
5  8.0e-06  group.5 0.000010
6  1.0e-05  group.6 0.000014
7  1.4e-05  group.7 0.000018
8  1.8e-05  group.8 0.000026
9  2.6e-05  group.9 0.000042
10 4.2e-05 group.10 0.000164
```

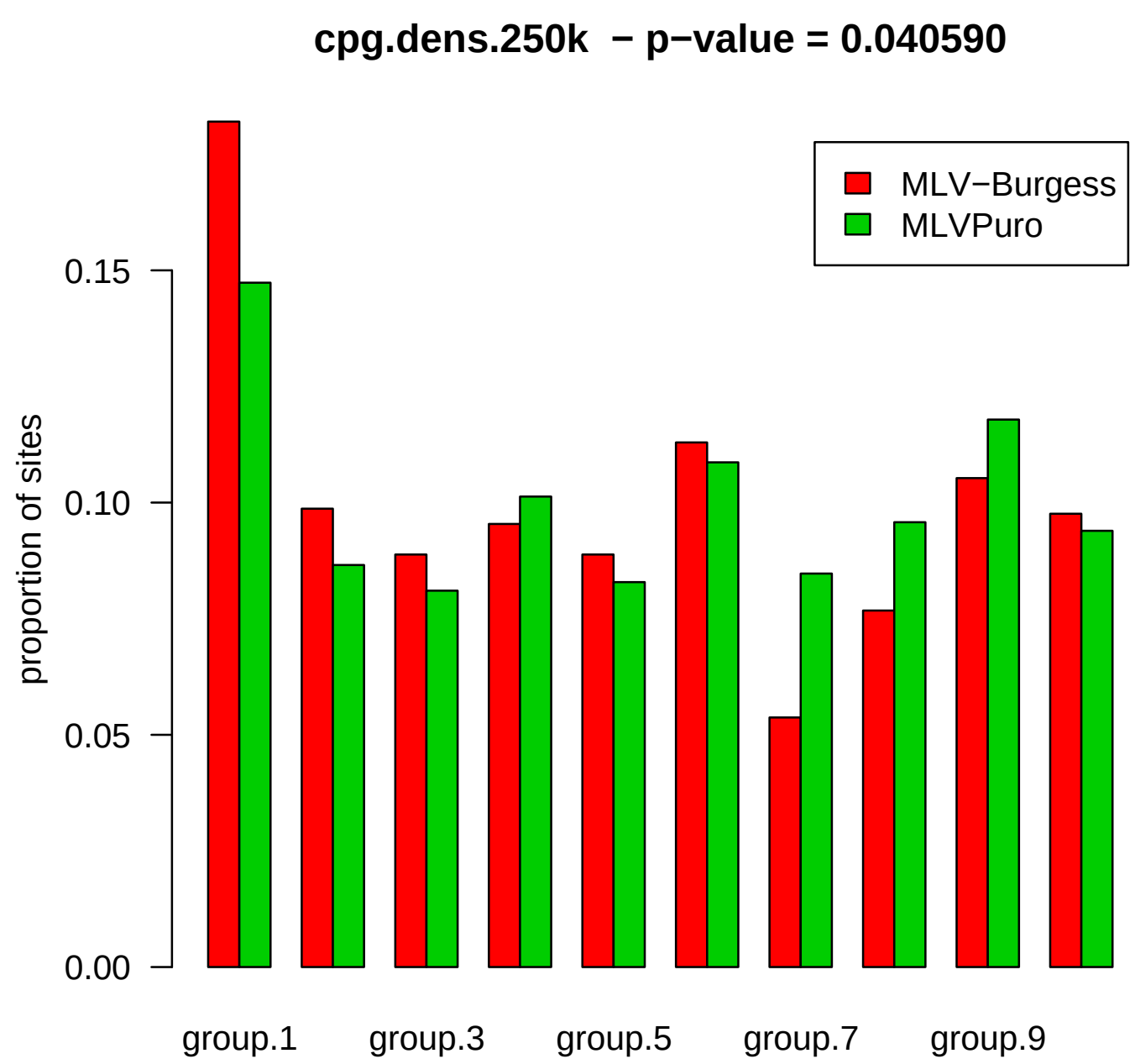

4.5 500 kiloBase Window

In the barplot that follows we examine the association of insertion sites with expression density in a 500 kilobase window surrounding each locus. First, we count just the number of genes on the represented on the chip.

```
 Category limits

         lower category        upper
1 0.000000e+00  group.1 1.480000e-06
2 1.480000e-06  group.2 2.500000e-06
3 2.500000e-06  group.3 3.666667e-06
4 3.666667e-06  group.4 5.000000e-06
5 5.000000e-06  group.5 6.666667e-06
6 6.666667e-06  group.6 8.926667e-06
7 8.926667e-06  group.7 1.150000e-05
8 1.150000e-05  group.8 1.666667e-05
9 1.666667e-05  group.9 7.566667e-05
```

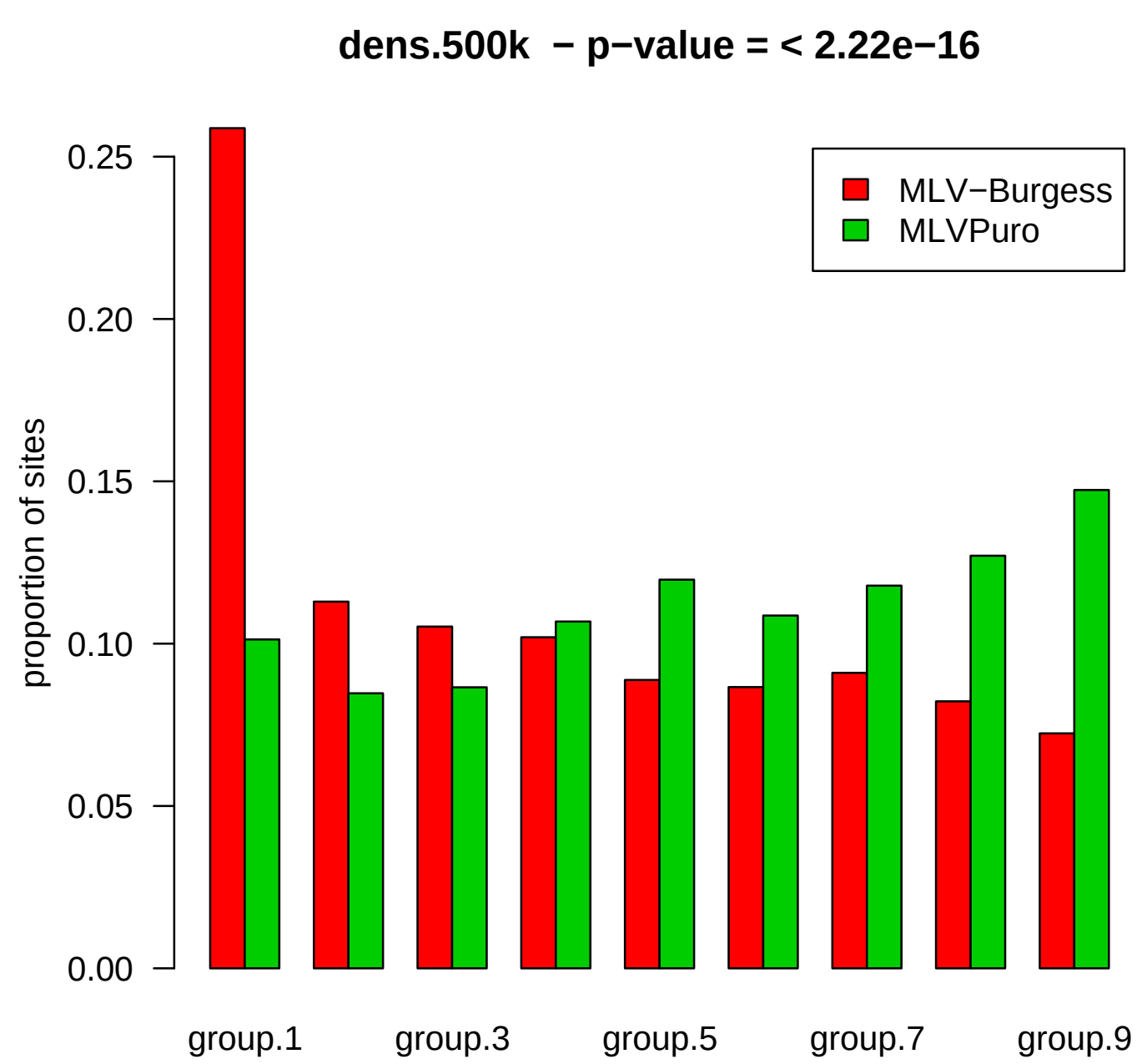

Here are the results for expression density. First, we count just genes that are in the upper half.

```
Category limits

          lower category        upper
1 0.000000e+00  group.1 1.777778e-07
2 1.777778e-07  group.2 1.000000e-06
3 1.000000e-06  group.3 2.000000e-06
4 2.000000e-06  group.4 2.500000e-06
5 2.500000e-06  group.5 3.333333e-06
6 3.333333e-06  group.6 4.500000e-06
7 4.500000e-06  group.7 6.295238e-06
8 6.295238e-06  group.8 9.000000e-06
9 9.000000e-06  group.9 3.633333e-05
```

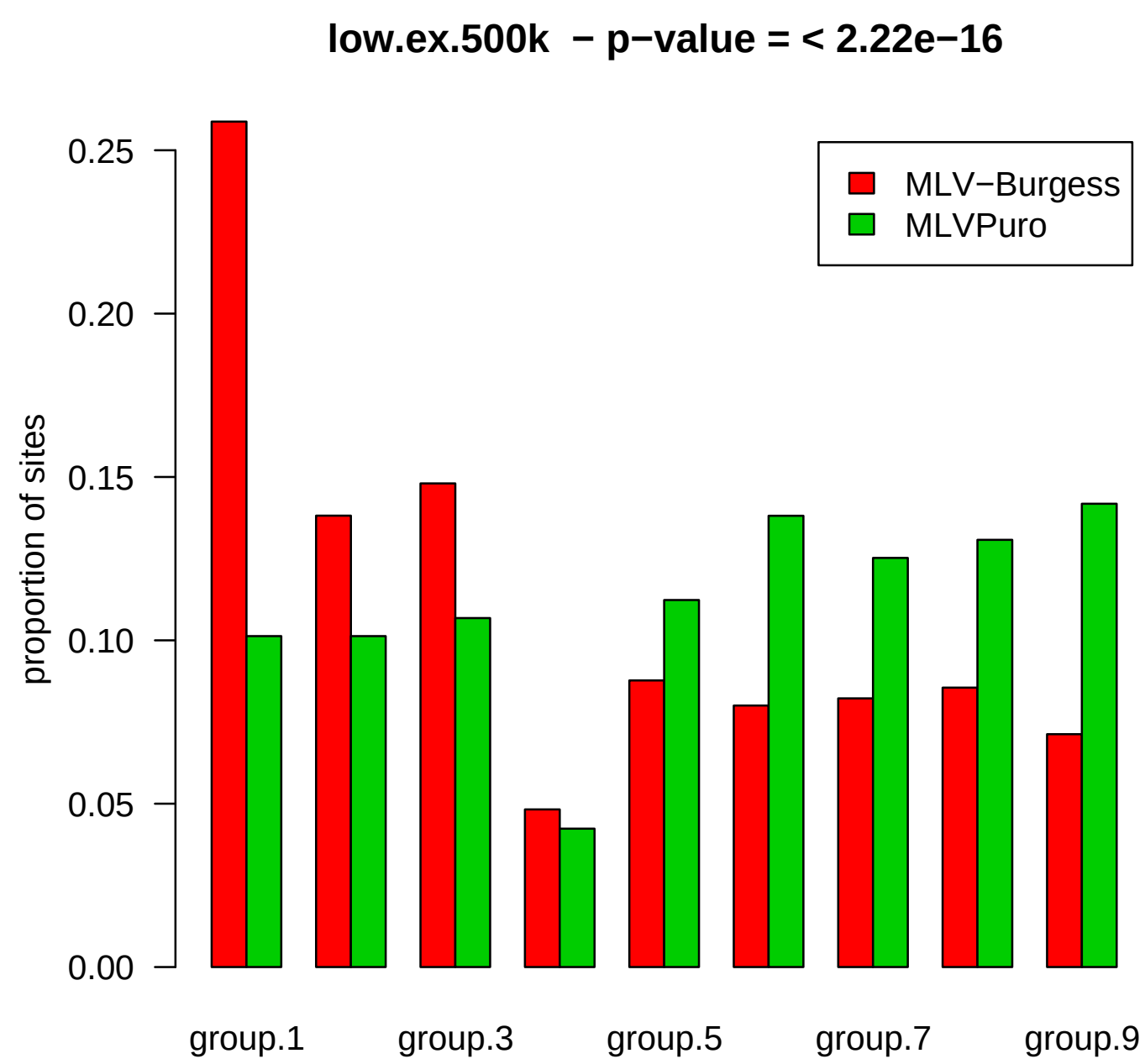

Now we count genes in the upper $1/8^{th}$:

```
Category limits

         lower category        upper
1 0.000000e+00  group.1 6.666667e-07
2 6.666667e-07  group.2 1.000000e-06
3 1.000000e-06  group.3 2.000000e-06
4 2.000000e-06  group.4 2.400000e-06
5 2.400000e-06  group.5 3.666667e-06
6 3.666667e-06  group.6 5.333333e-06
7 5.333333e-06  group.7 2.066667e-05
```

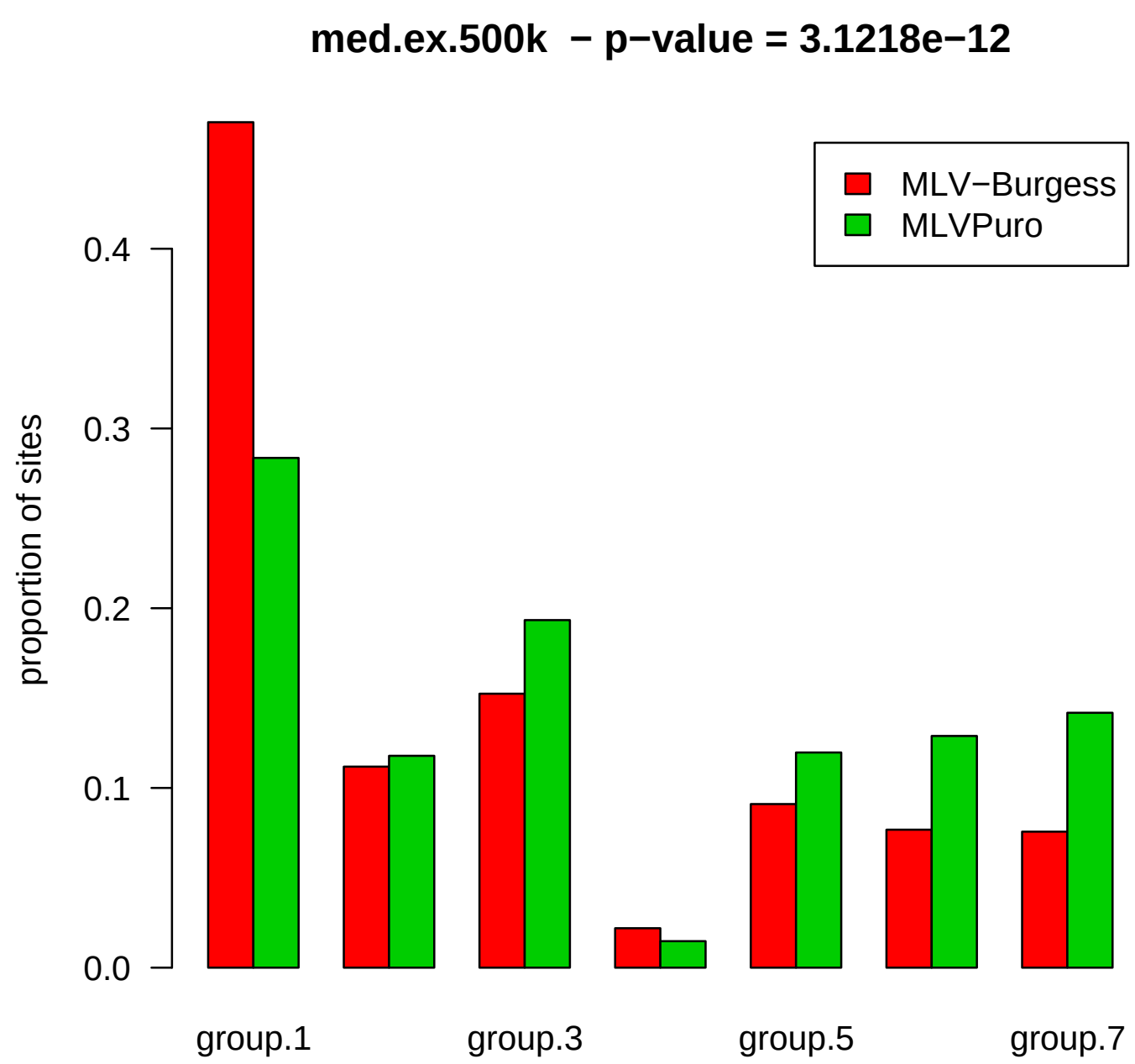

And here we count genes in the upper $1/16^{th}$:

```
Category limits

         lower category        upper
1 0.000000e+00  group.1 6.666667e-07
2 6.666667e-07  group.2 1.400000e-06
3 1.400000e-06  group.3 2.000000e-06
4 2.000000e-06  group.4 3.000000e-06
5 3.000000e-06  group.5 1.400000e-05
```

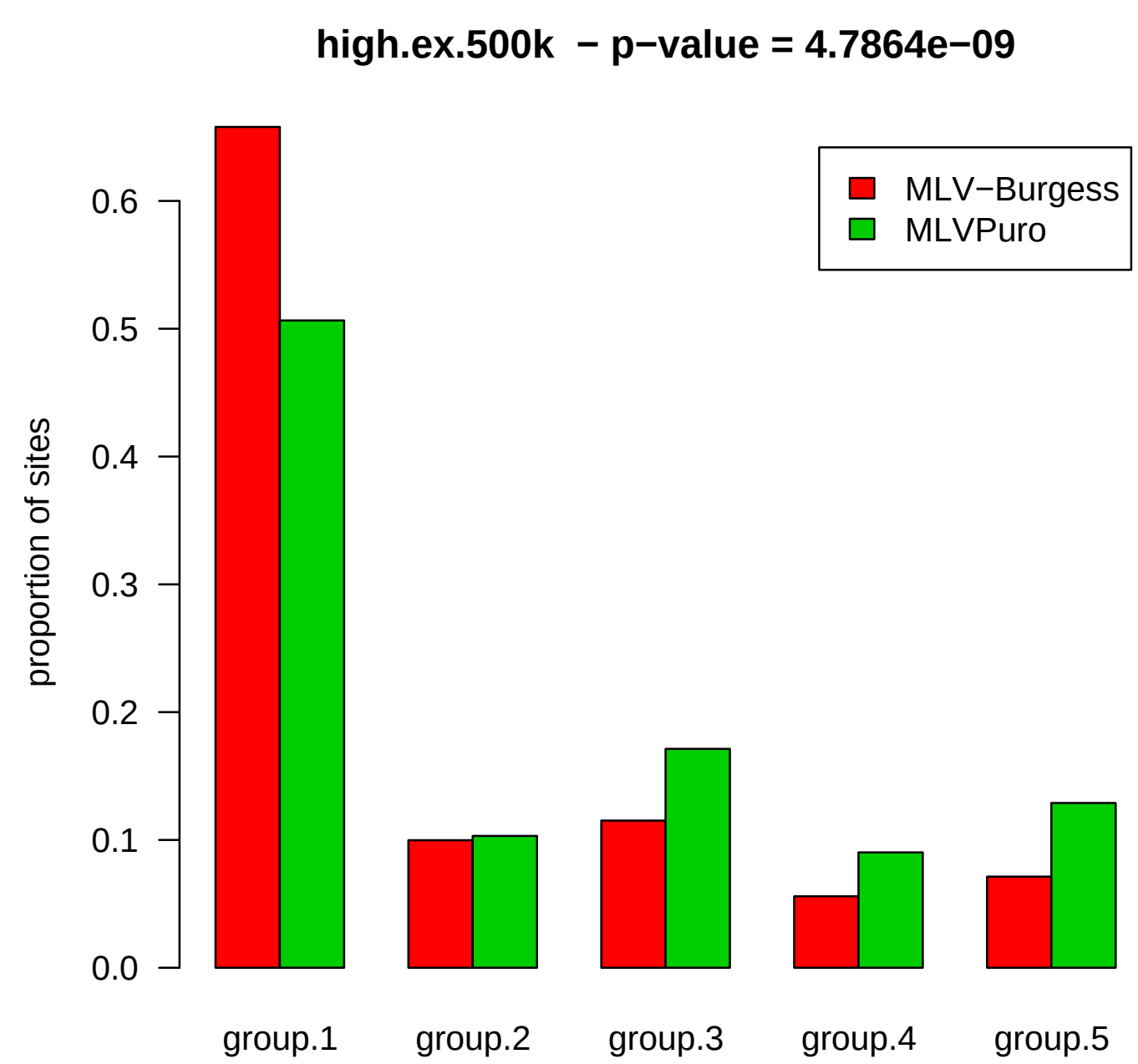

Here the effect of density of CpG islands is studied:

```
Category limits

     lower  category    upper
1  0.0e+00   group.1 0.000002
2  2.0e-06   group.2 0.000004
3  4.0e-06   group.3 0.000006
4  6.0e-06   group.4 0.000008
5  8.0e-06   group.5 0.000010
6  1.0e-05   group.6 0.000013
7  1.3e-05   group.7 0.000017
8  1.7e-05   group.8 0.000025
9  2.5e-05   group.9 0.000037
10 3.7e-05  group.10 0.000151
```

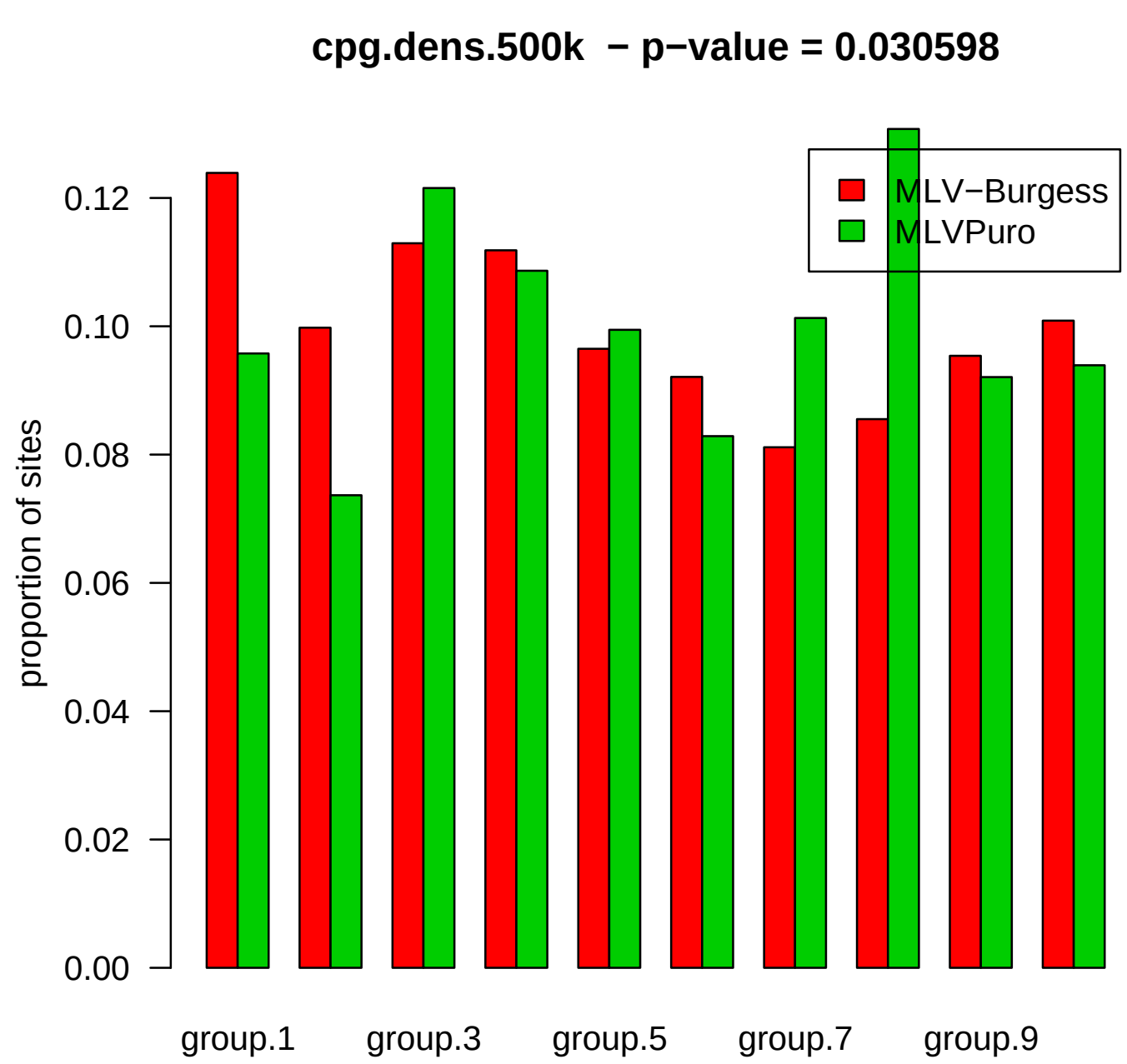

4.6 1 megaBase Window

In the barplot that follows we examine the association of insertion sites with expression density in a 1 megabase window surrounding each locus. First, we count just the number of genes on the represented on the chip.

```
 Category limits

          lower category        upper
1  0.000000e+00  group.1 8.190476e-07
2  8.190476e-07  group.2 1.750000e-06
3  1.750000e-06  group.3 2.666667e-06
4  2.666667e-06  group.4 3.700000e-06
5  3.700000e-06  group.5 4.844444e-06
6  4.844444e-06  group.6 6.250000e-06
7  6.250000e-06  group.7 8.161905e-06
8  8.161905e-06  group.8 1.101576e-05
9  1.101576e-05  group.9 1.656333e-05
10 1.656333e-05 group.10 5.716667e-05
```

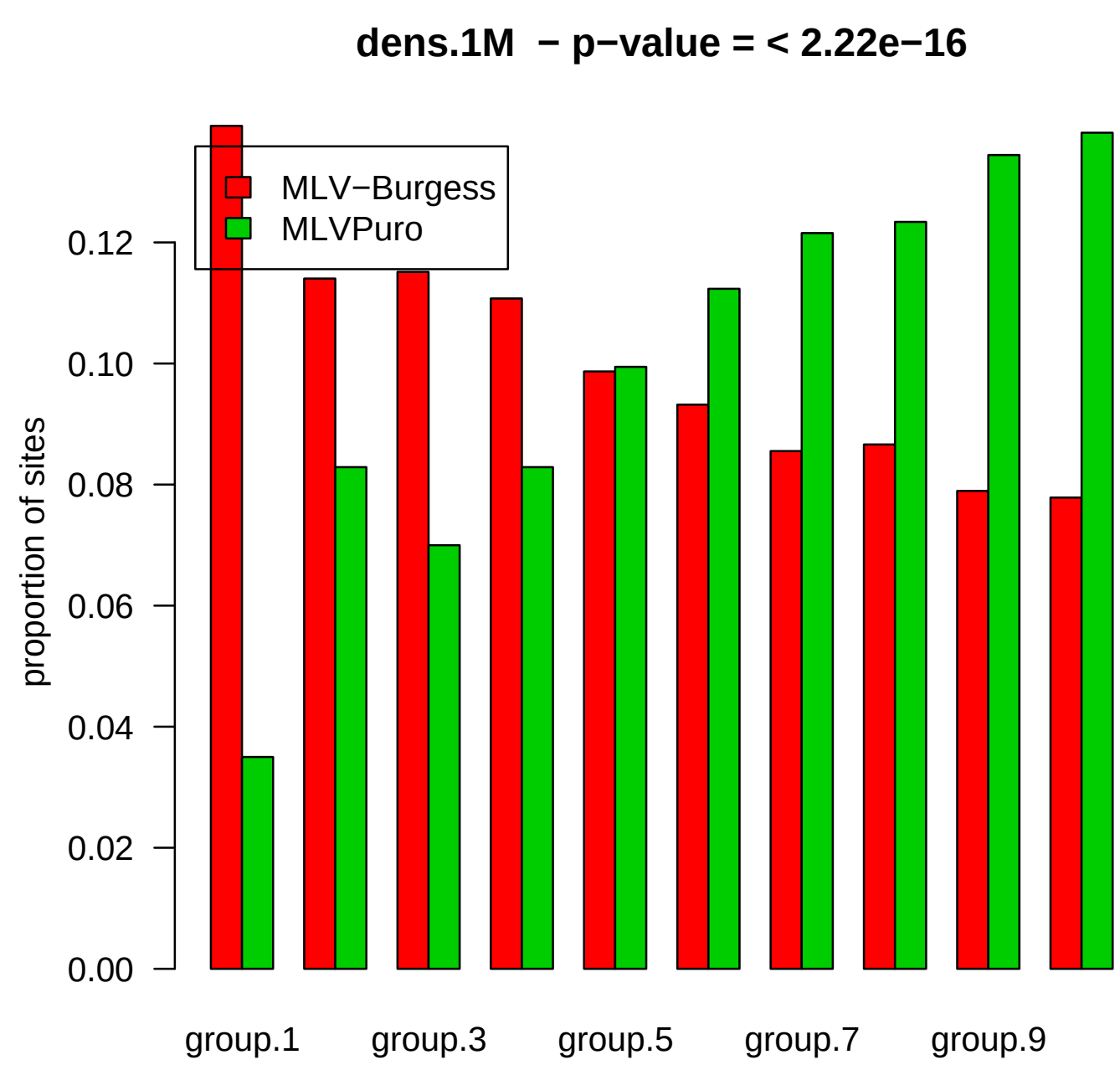

Here are the results for expression density. First, we count just genes that are in the upper half.

```
Category limits

         lower category        upper
1 0.000000e+00  group.1 7.000000e-07
2 7.000000e-07  group.2 1.250000e-06
3 1.250000e-06  group.3 1.750000e-06
4 1.750000e-06  group.4 2.333333e-06
5 2.333333e-06  group.5 3.083333e-06
6 3.083333e-06  group.6 4.166667e-06
7 4.166667e-06  group.7 5.416667e-06
8 5.416667e-06  group.8 8.353333e-06
9 8.353333e-06  group.9 2.891667e-05
```

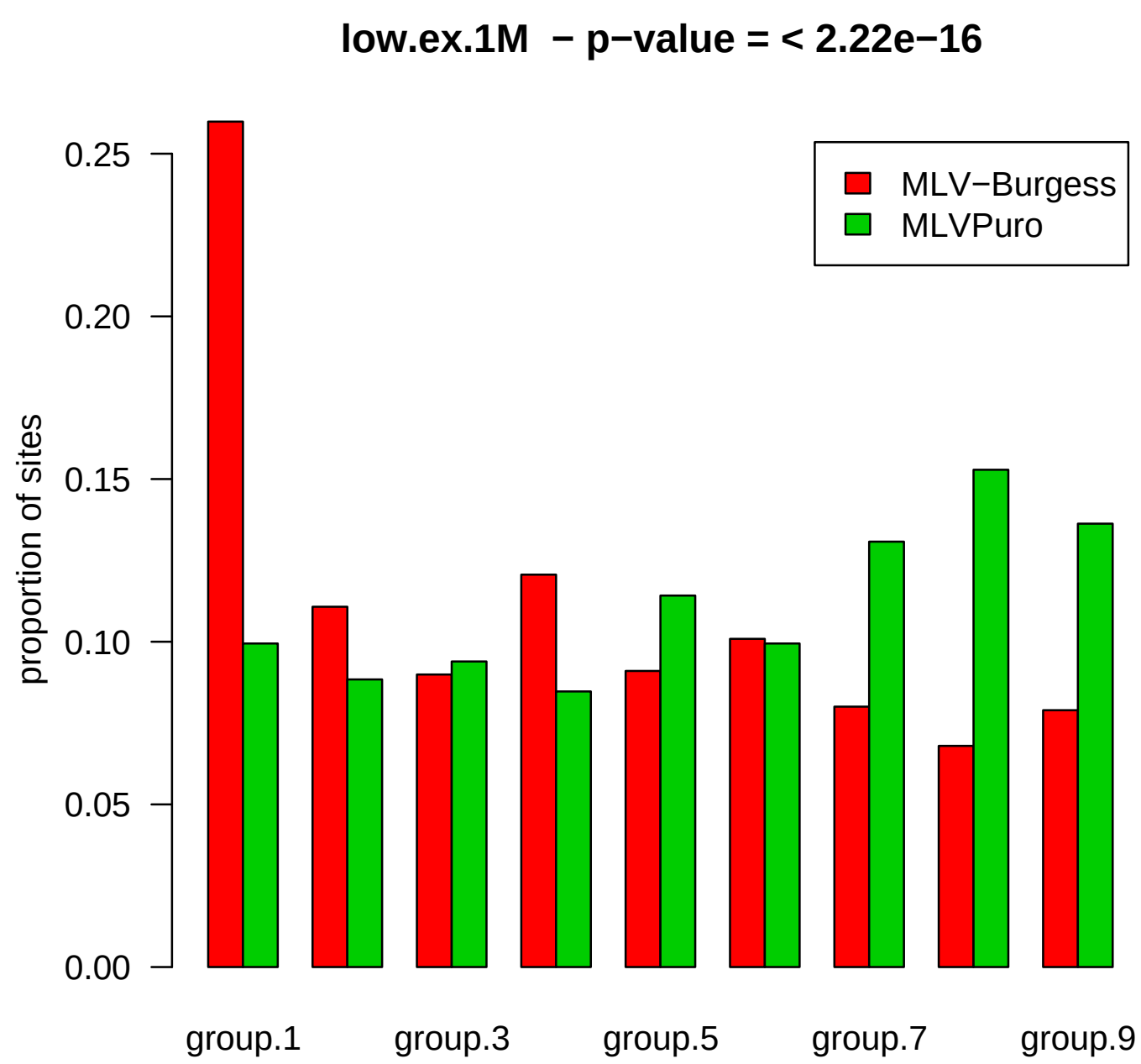

Now we count genes in the upper $1/8^{th}$:

```
Category limits

         lower category        upper
1 0.000000e+00  group.1 1.111111e-07
2 1.111111e-07  group.2 5.000000e-07
3 5.000000e-07  group.3 9.000000e-07
4 9.000000e-07  group.4 1.200000e-06
5 1.200000e-06  group.5 1.666667e-06
6 1.666667e-06  group.6 2.200000e-06
7 2.200000e-06  group.7 3.000000e-06
8 3.000000e-06  group.8 4.500000e-06
9 4.500000e-06  group.9 1.600000e-05
```

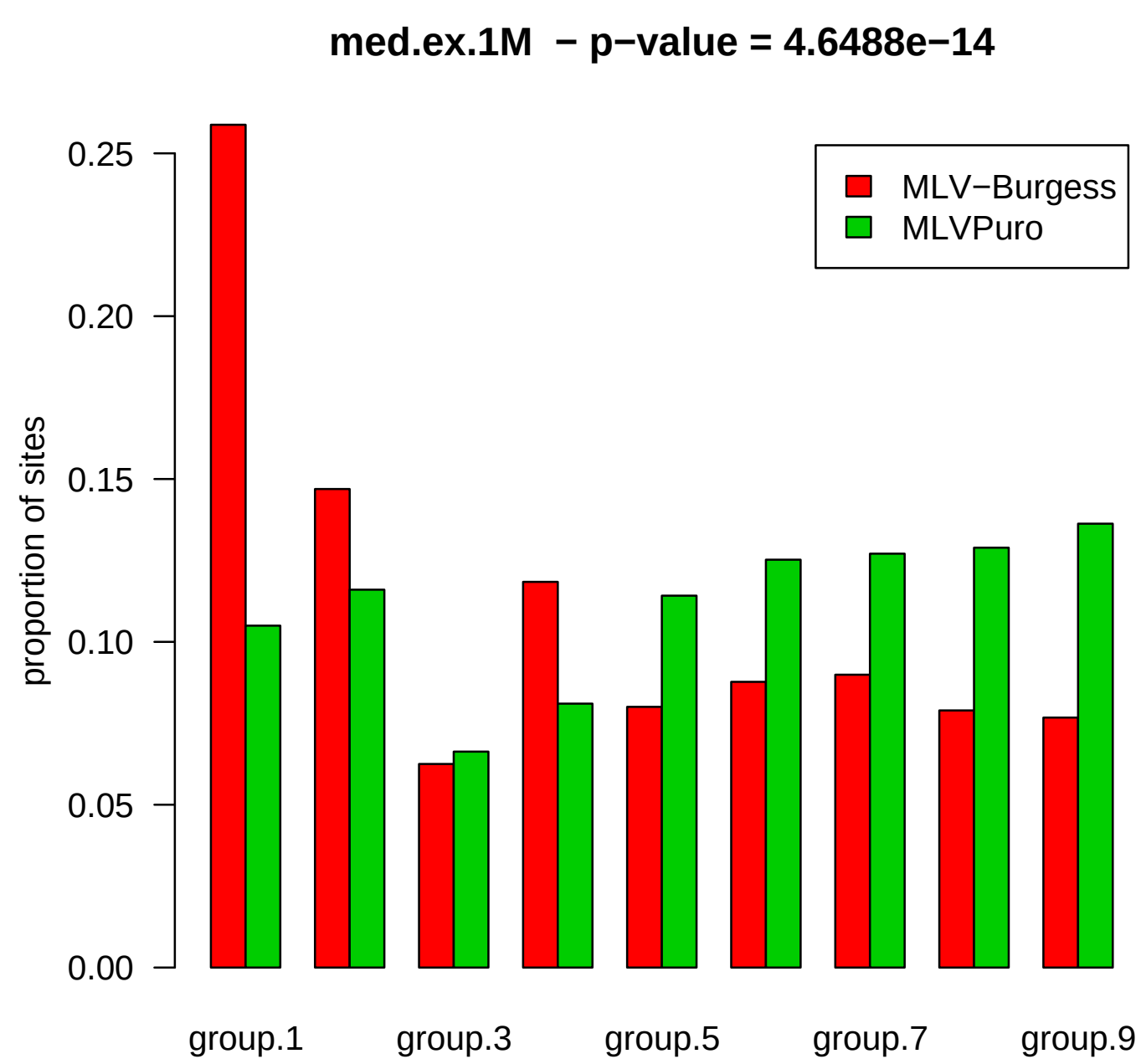

And here we count genes in the upper $1/16^{th}$:

```
Category limits

        lower  category        upper
1 0.000000e+00  group.1 2.500000e-07
2 2.500000e-07  group.2 5.000000e-07
3 5.000000e-07  group.3 1.000000e-06
4 1.000000e-06  group.4 1.122222e-06
5 1.122222e-06  group.5 1.666667e-06
6 1.666667e-06  group.6 2.500000e-06
7 2.500000e-06  group.7 1.050000e-05
```

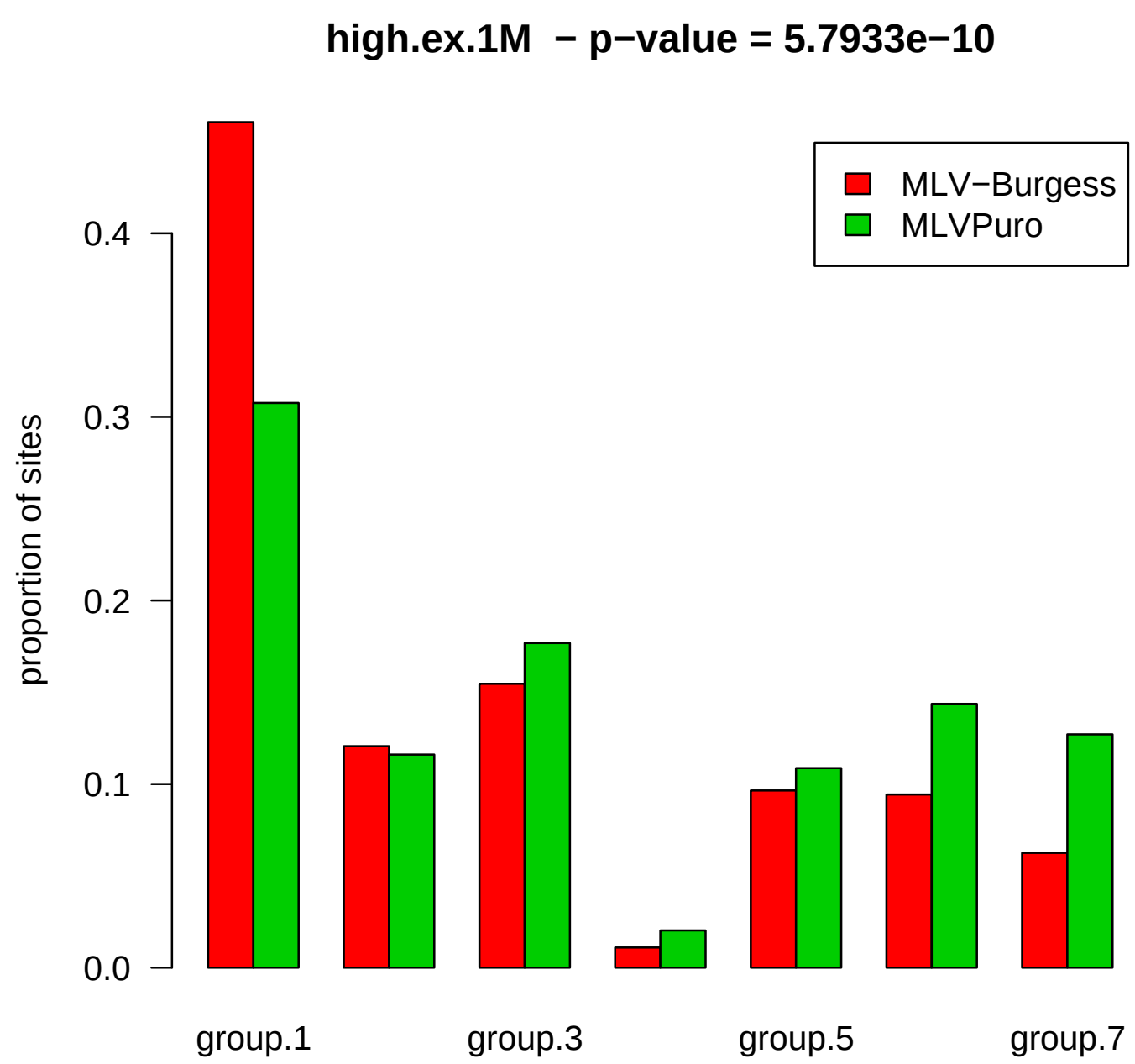

Here the effect of density of CpG islands is studied:

```
Category limits

      lower category    upper
1  0.00e+00  group.1 3.00e-06
2  3.00e-06  group.2 4.50e-06
3  4.50e-06  group.3 6.00e-06
4  6.00e-06  group.4 7.50e-06
5  7.50e-06  group.5 9.50e-06
6  9.50e-06  group.6 1.25e-05
7  1.25e-05  group.7 1.65e-05
8  1.65e-05  group.8 2.25e-05
9  2.25e-05  group.9 3.33e-05
10 3.33e-05 group.10 1.48e-04
```

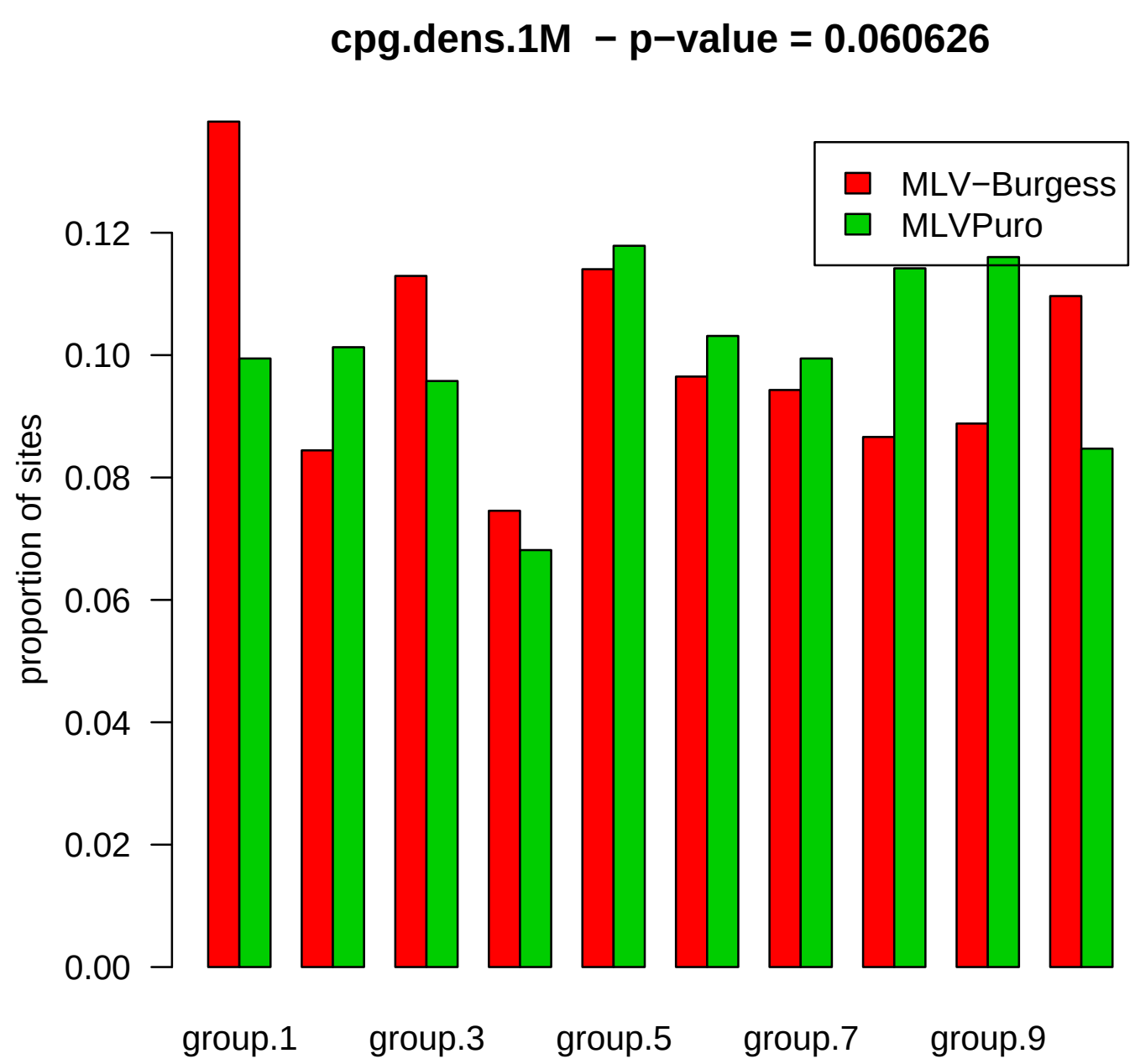

4.7 2 megaBase Window

In the barplot that follows we examine the association of insertion sites with expression density in a 2 megabase window surrounding each locus. First, we count just the number of genes on the represented on the chip.

```
 Category limits

          lower category        upper
1  0.000000e+00  group.1 1.250000e-06
2  1.250000e-06  group.2 1.916667e-06
3  1.916667e-06  group.3 2.736508e-06
4  2.736508e-06  group.4 3.619167e-06
5  3.619167e-06  group.5 4.648810e-06
6  4.648810e-06  group.6 5.837143e-06
7  5.837143e-06  group.7 7.583333e-06
8  7.583333e-06  group.8 1.008750e-05
9  1.008750e-05  group.9 1.575000e-05
10 1.575000e-05 group.10 3.950000e-05
```

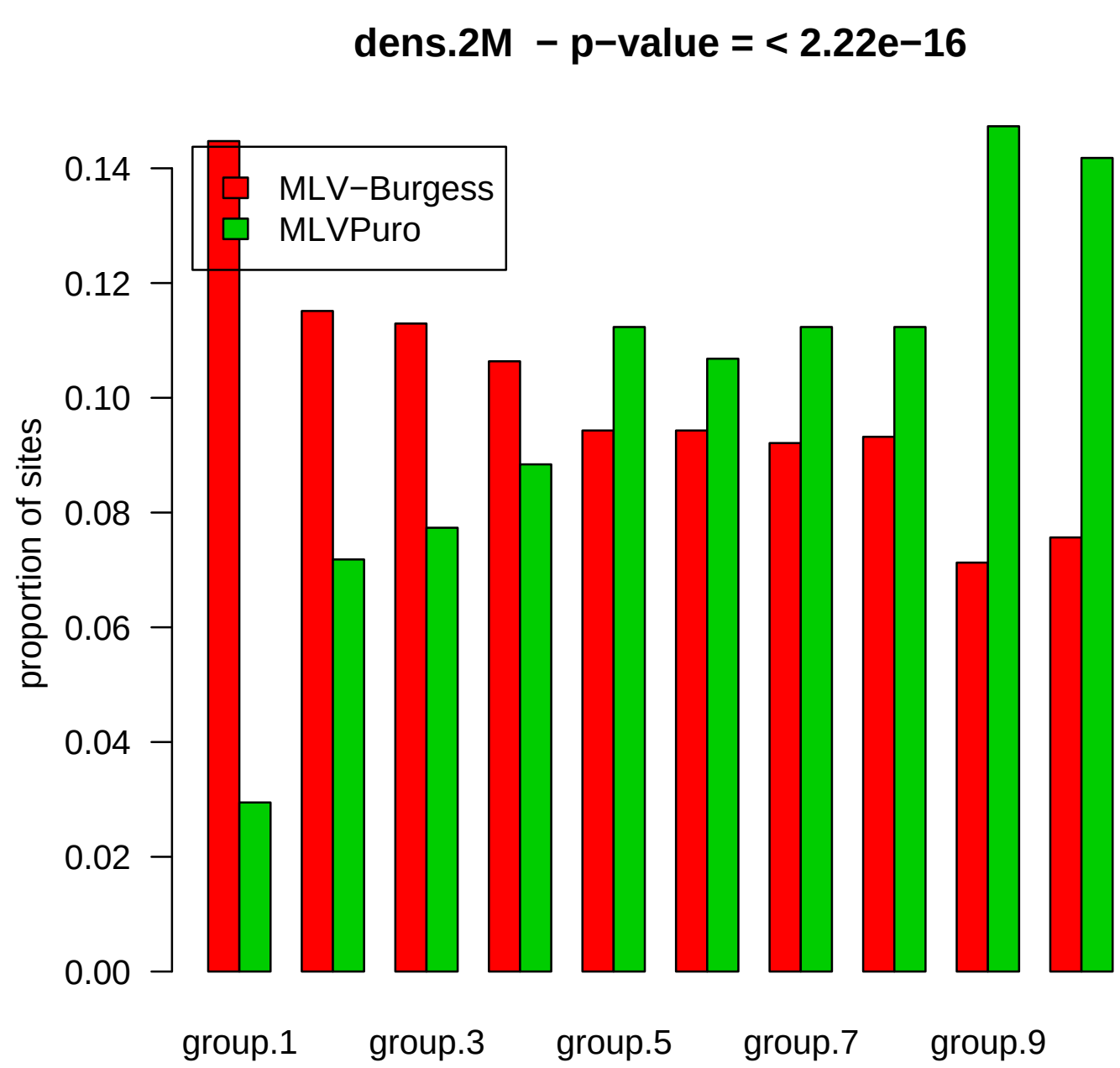

Here are the results for expression density. First, we count just genes that are in the upper half.

```
 Category limits

          lower category        upper
1  0.000000e+00  group.1 4.185714e-07
2  4.185714e-07  group.2 8.750000e-07
3  8.750000e-07  group.3 1.250000e-06
4  1.250000e-06  group.4 1.713333e-06
5  1.713333e-06  group.5 2.216667e-06
6  2.216667e-06  group.6 2.750000e-06
7  2.750000e-06  group.7 3.655000e-06
8  3.655000e-06  group.8 5.250000e-06
9  5.250000e-06  group.9 7.683333e-06
10 7.683333e-06 group.10 1.933333e-05
```

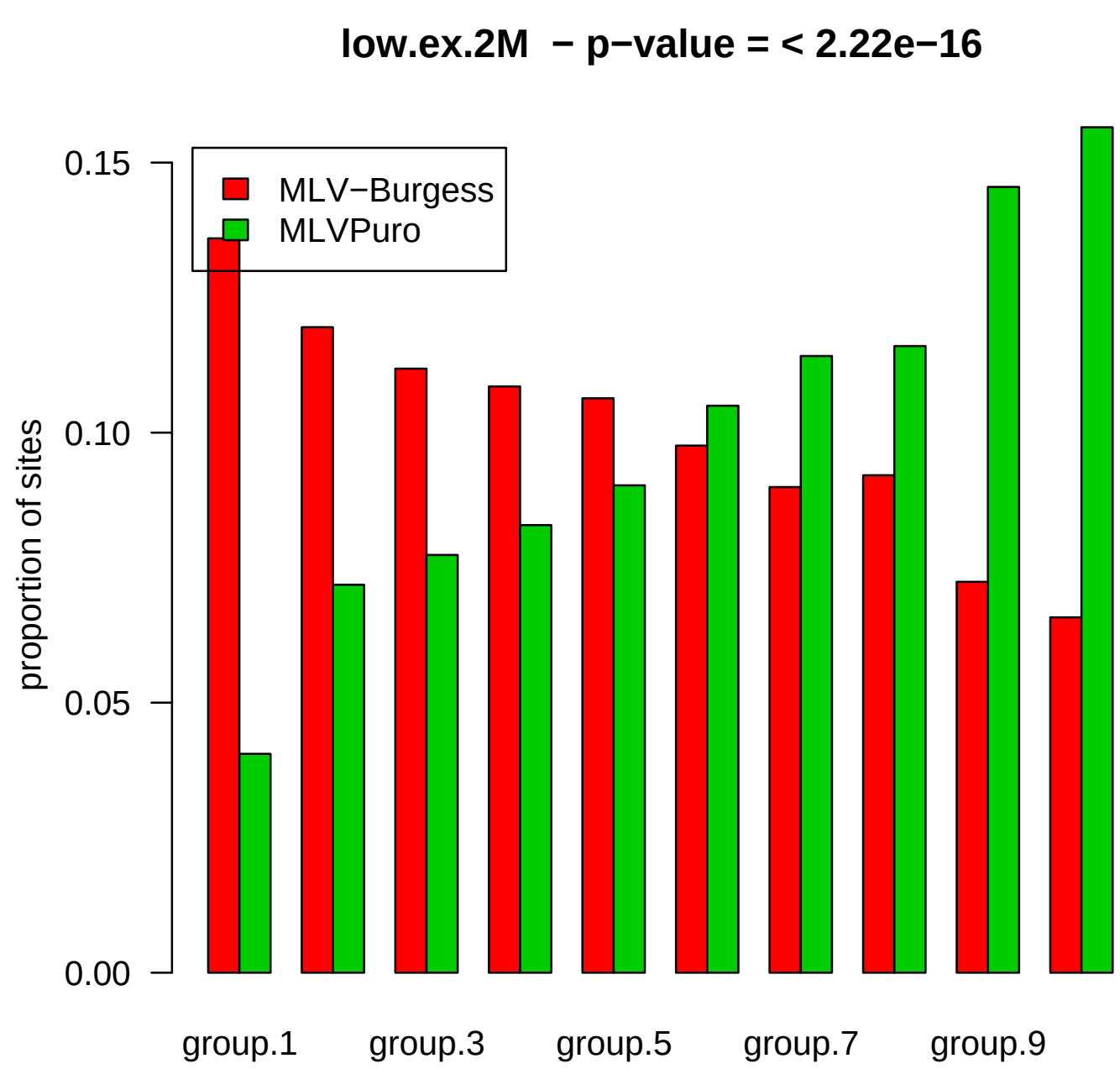

Now we count genes in the upper $1/8^{th}$:

```
Category limits

          lower  category        upper
1  0.000000e+00   group.1 7.619048e-08
2  7.619048e-08   group.2 3.333333e-07
3  3.333333e-07   group.3 5.833333e-07
4  5.833333e-07   group.4 8.316667e-07
5  8.316667e-07   group.5 1.125000e-06
6  1.125000e-06   group.6 1.481667e-06
7  1.481667e-06   group.7 1.995000e-06
8  1.995000e-06   group.8 2.602778e-06
9  2.602778e-06   group.9 4.083333e-06
10 4.083333e-06  group.10 1.108333e-05
```

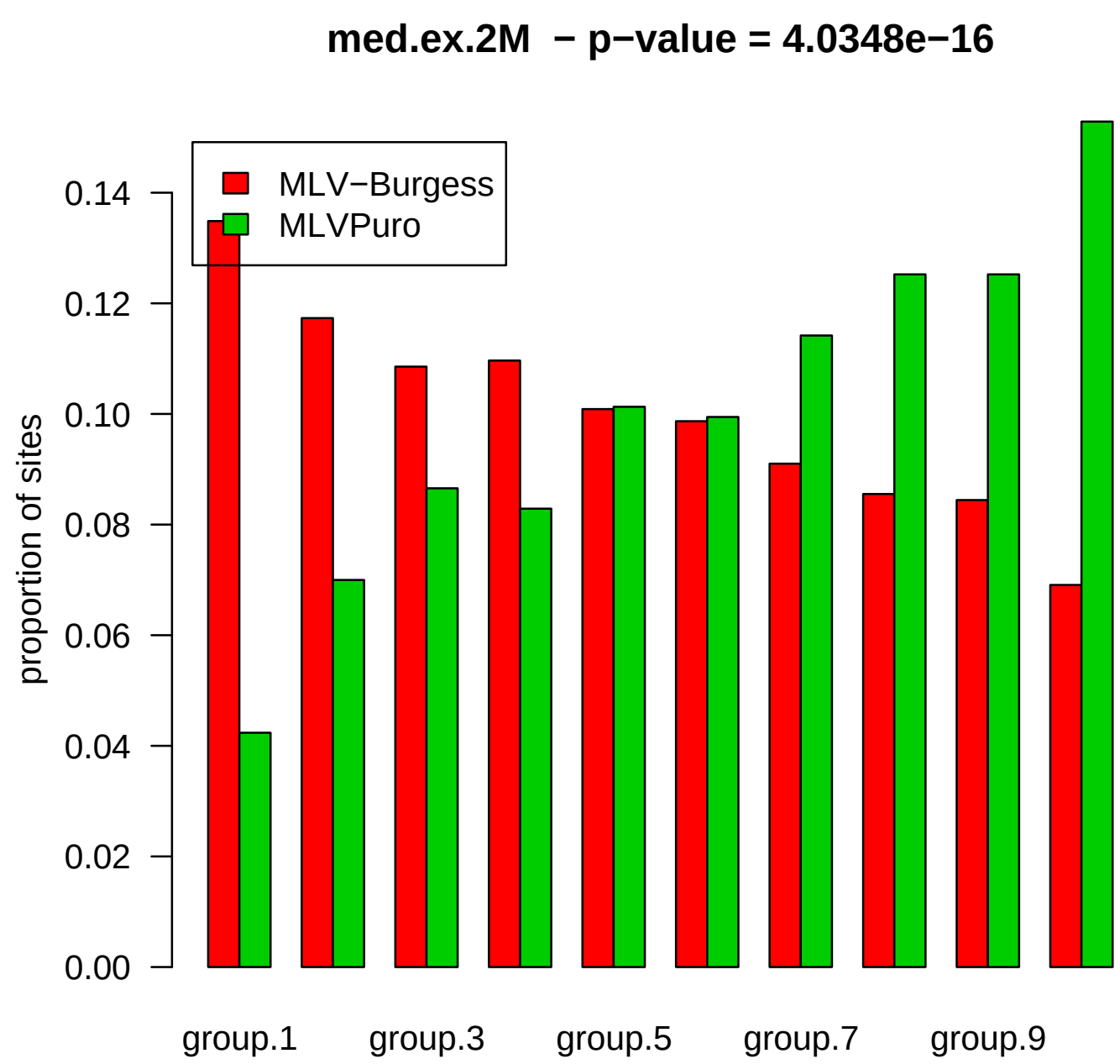

And here we count genes in the upper $1/16^{th}$:

```
Category limits

         lower category        upper
1 0.000000e+00  group.1 2.500000e-07
2 2.500000e-07  group.2 4.166667e-07
3 4.166667e-07  group.3 5.416667e-07
4 5.416667e-07  group.4 7.500000e-07
5 7.500000e-07  group.5 1.000000e-06
6 1.000000e-06  group.6 1.408333e-06
7 1.408333e-06  group.7 2.075000e-06
8 2.075000e-06  group.8 6.875000e-06
```

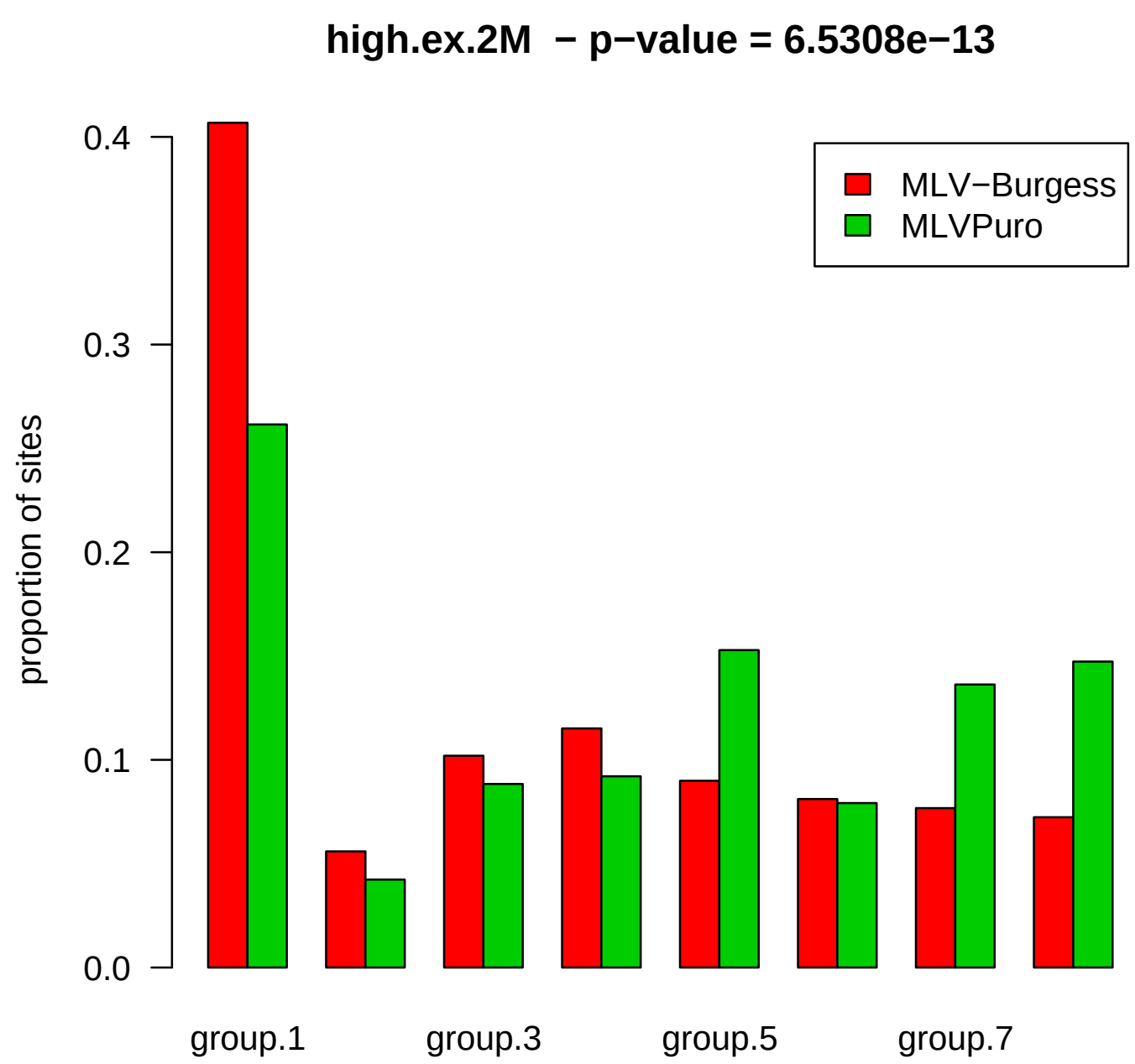

Here the effect of density of CpG islands is studied:

```
Category limits

       lower category        upper
1  0.000e+00  group.1 0.0000032500
2  3.250e-06  group.2 0.0000045000
3  4.500e-06  group.3 0.0000057500
4  5.750e-06  group.4 0.0000070000
5  7.000e-06  group.5 0.0000087500
6  8.750e-06  group.6 0.0000123500
7  1.235e-05  group.7 0.0000162500
8  1.625e-05  group.8 0.0000220500
9  2.205e-05  group.9 0.0000370000
10 3.700e-05 group.10 0.0001313264
```

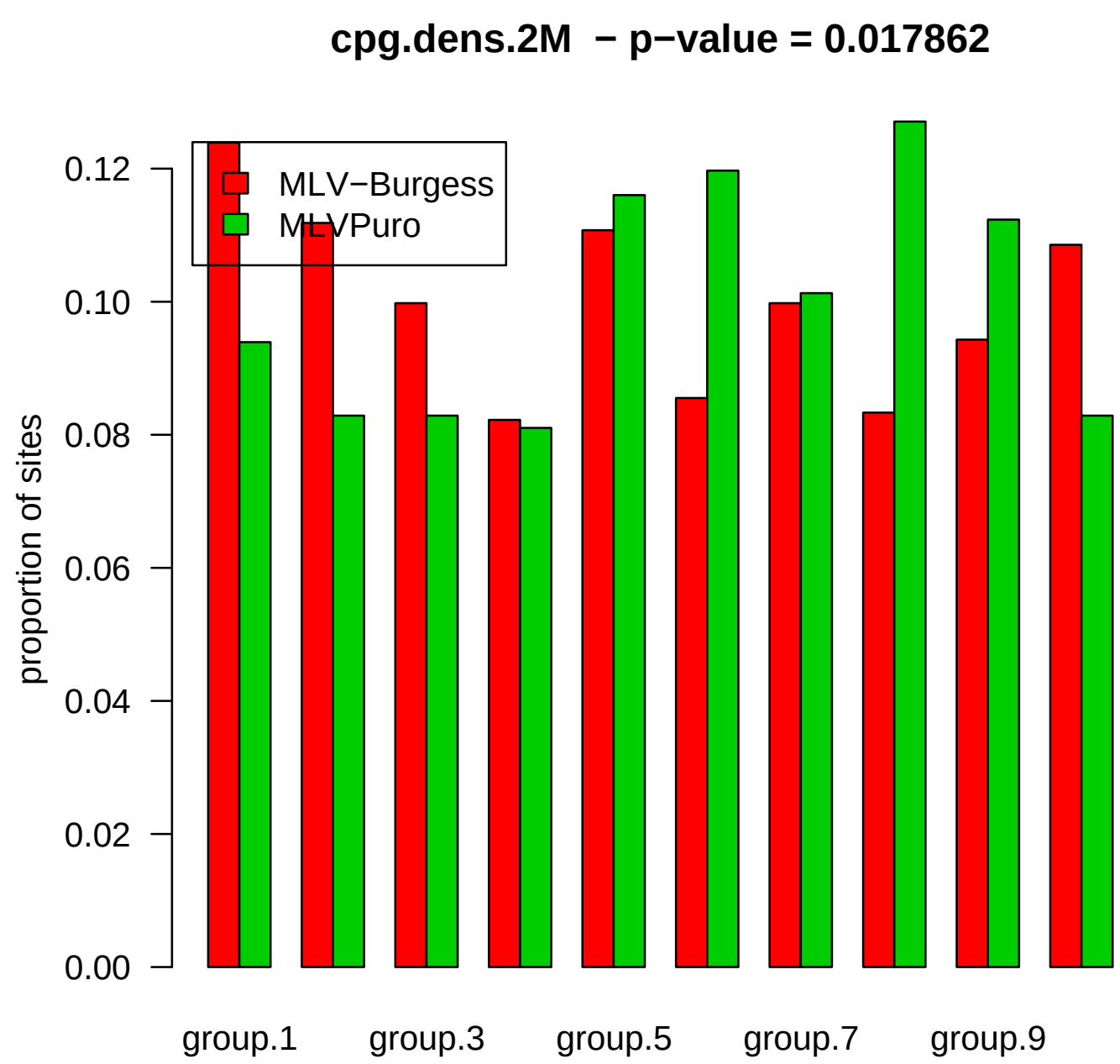

4.8 4 megaBase Window

In the barplot that follows we examine the association of insertion sites with expression density in a 4 megabase window surrounding each locus. First, we count just the number of genes on the represented on the chip.

```
 Category limits

          lower category        upper
1  0.000000e+00  group.1 1.462121e-06
2  1.462121e-06  group.2 2.162857e-06
3  2.162857e-06  group.3 2.758750e-06
4  2.758750e-06  group.4 3.453571e-06
5  3.453571e-06  group.5 4.229167e-06
6  4.229167e-06  group.6 5.488333e-06
7  5.488333e-06  group.7 7.319167e-06
8  7.319167e-06  group.8 9.755714e-06
9  9.755714e-06  group.9 1.441848e-05
10 1.441848e-05 group.10 3.462917e-05
```

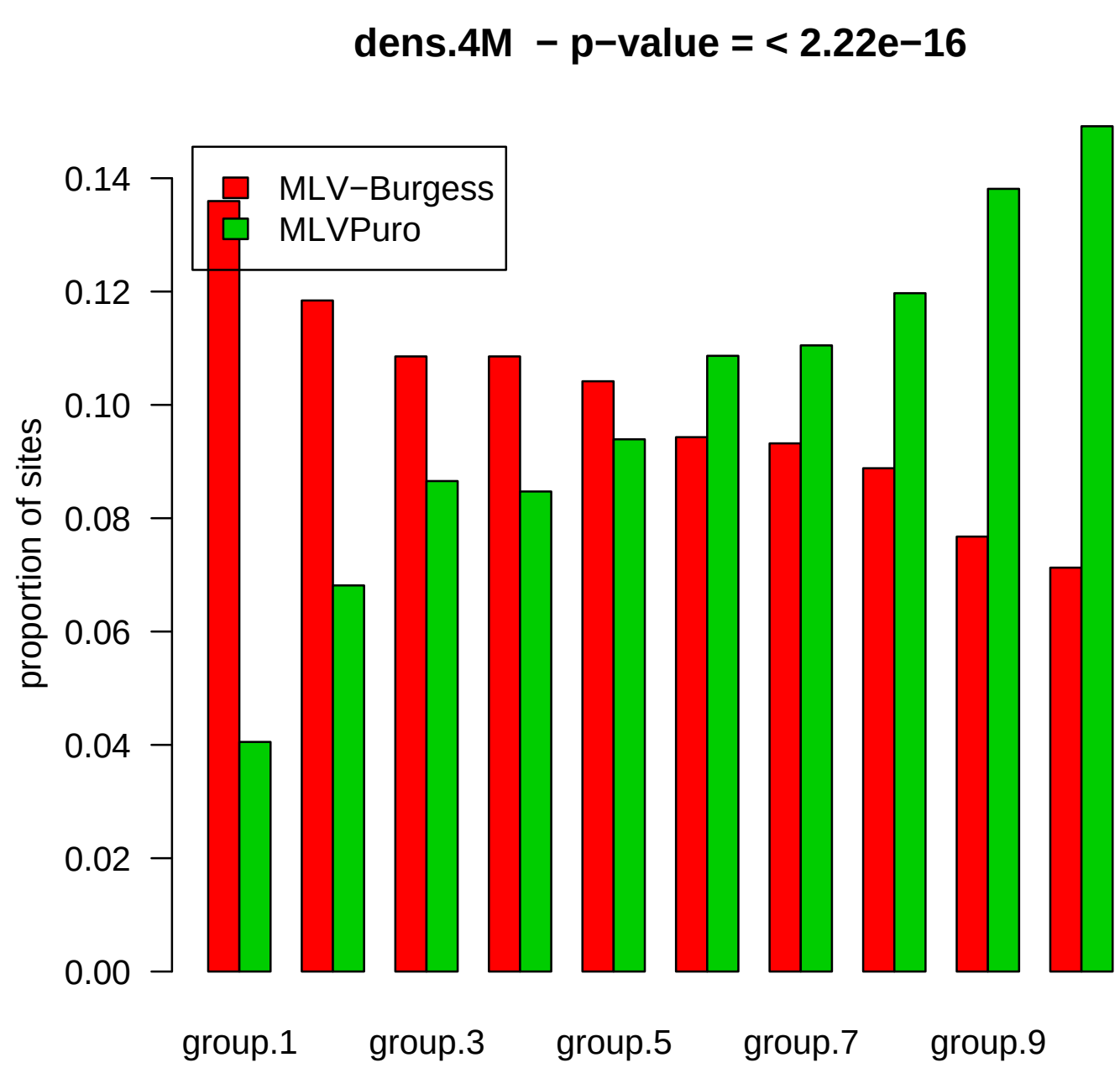

Here are the results for expression density. First, we count just genes that are in the upper half.

```
Category limits

          lower category        upper
1  0.000000e+00  group.1 5.955357e-07
2  5.955357e-07  group.2 9.583333e-07
3  9.583333e-07  group.3 1.208333e-06
4  1.208333e-06  group.4 1.562500e-06
5  1.562500e-06  group.5 2.000000e-06
6  2.000000e-06  group.6 2.614167e-06
7  2.614167e-06  group.7 3.550794e-06
8  3.550794e-06  group.8 4.844167e-06
9  4.844167e-06  group.9 6.855357e-06
10 6.855357e-06 group.10 1.792917e-05
```

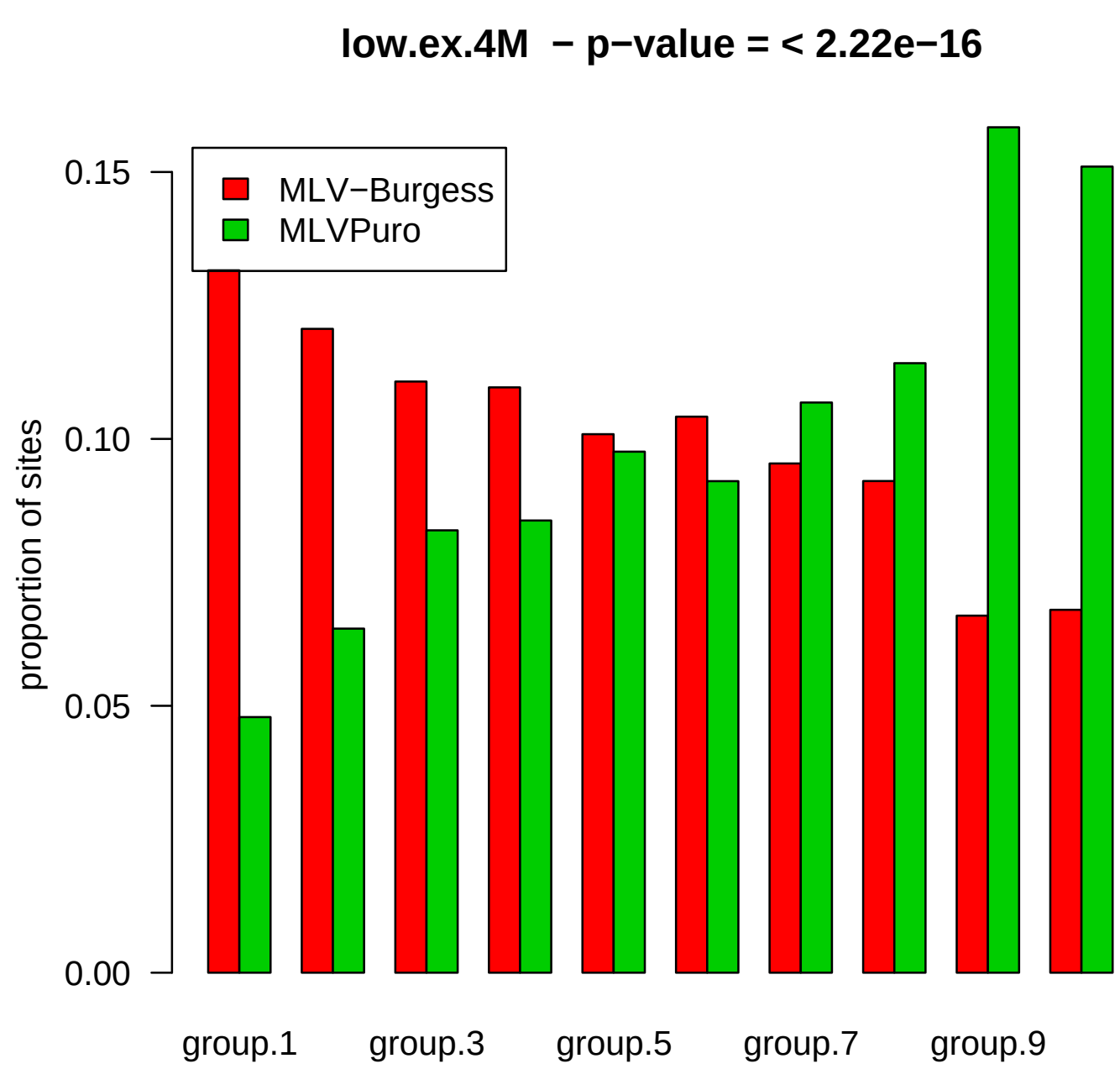

Now we count genes in the upper $1/8^{th}$:

```
Category limits

          lower  category        upper
1  0.000000e+00   group.1 2.500000e-07
2  2.500000e-07   group.2 4.154762e-07
3  4.154762e-07   group.3 5.938095e-07
4  5.938095e-07   group.4 7.708333e-07
5  7.708333e-07   group.5 1.041667e-06
6  1.041667e-06   group.6 1.360000e-06
7  1.360000e-06   group.7 1.854167e-06
8  1.854167e-06   group.8 2.550000e-06
9  2.550000e-06   group.9 3.588333e-06
10 3.588333e-06  group.10 9.337500e-06
```

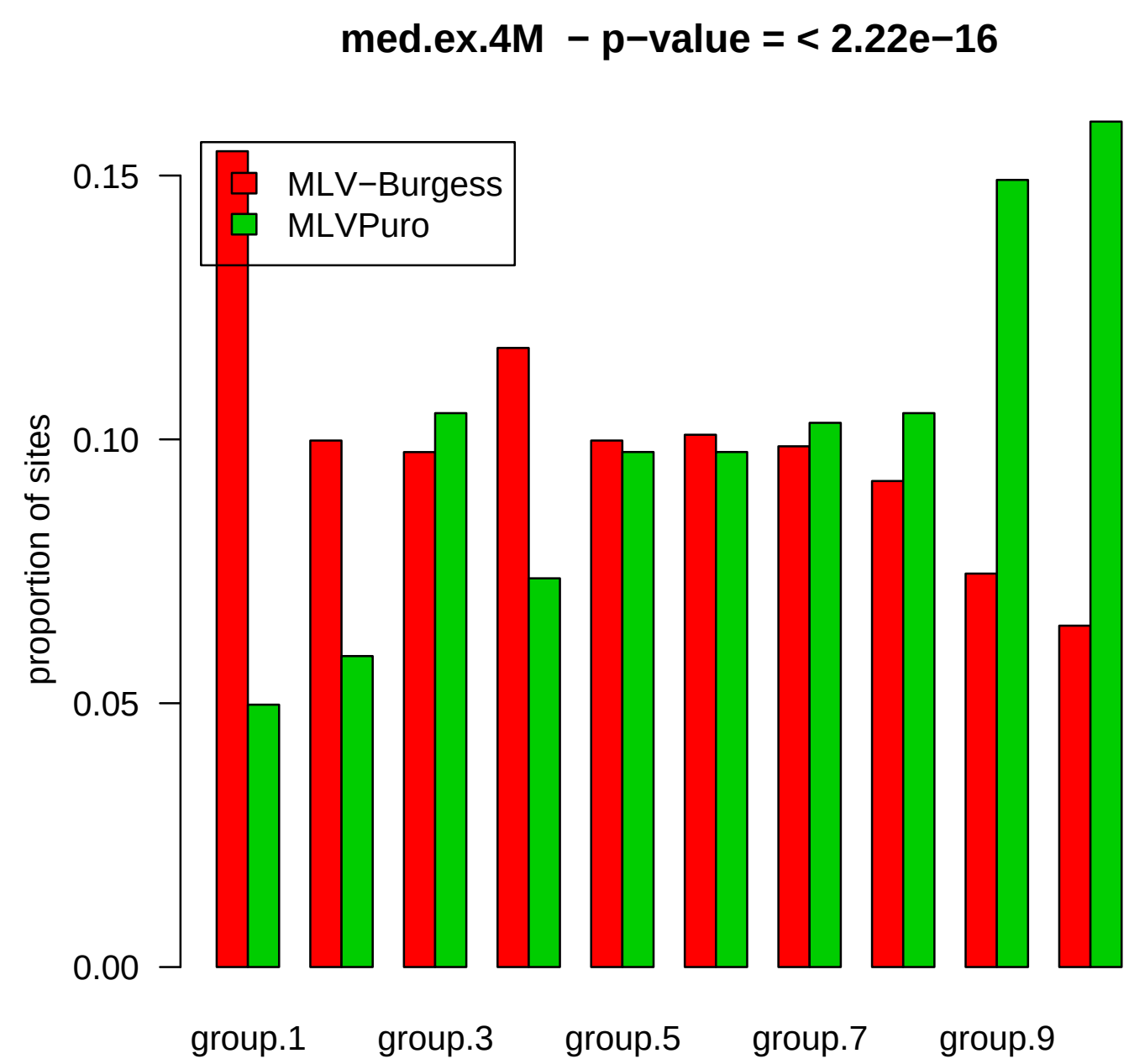

And here we count genes in the upper $1/16^{th}$:

```
Category limits

          lower category        upper
1  0.000000e+00  group.1 5.000000e-08
2  5.000000e-08  group.2 1.696429e-07
3  1.696429e-07  group.3 2.915793e-07
4  2.915793e-07  group.4 3.773061e-07
5  3.773061e-07  group.5 5.208333e-07
6  5.208333e-07  group.6 6.666667e-07
7  6.666667e-07  group.7 8.958333e-07
8  8.958333e-07  group.8 1.250000e-06
9  1.250000e-06  group.9 1.742760e-06
10 1.742760e-06 group.10 5.766667e-06
```

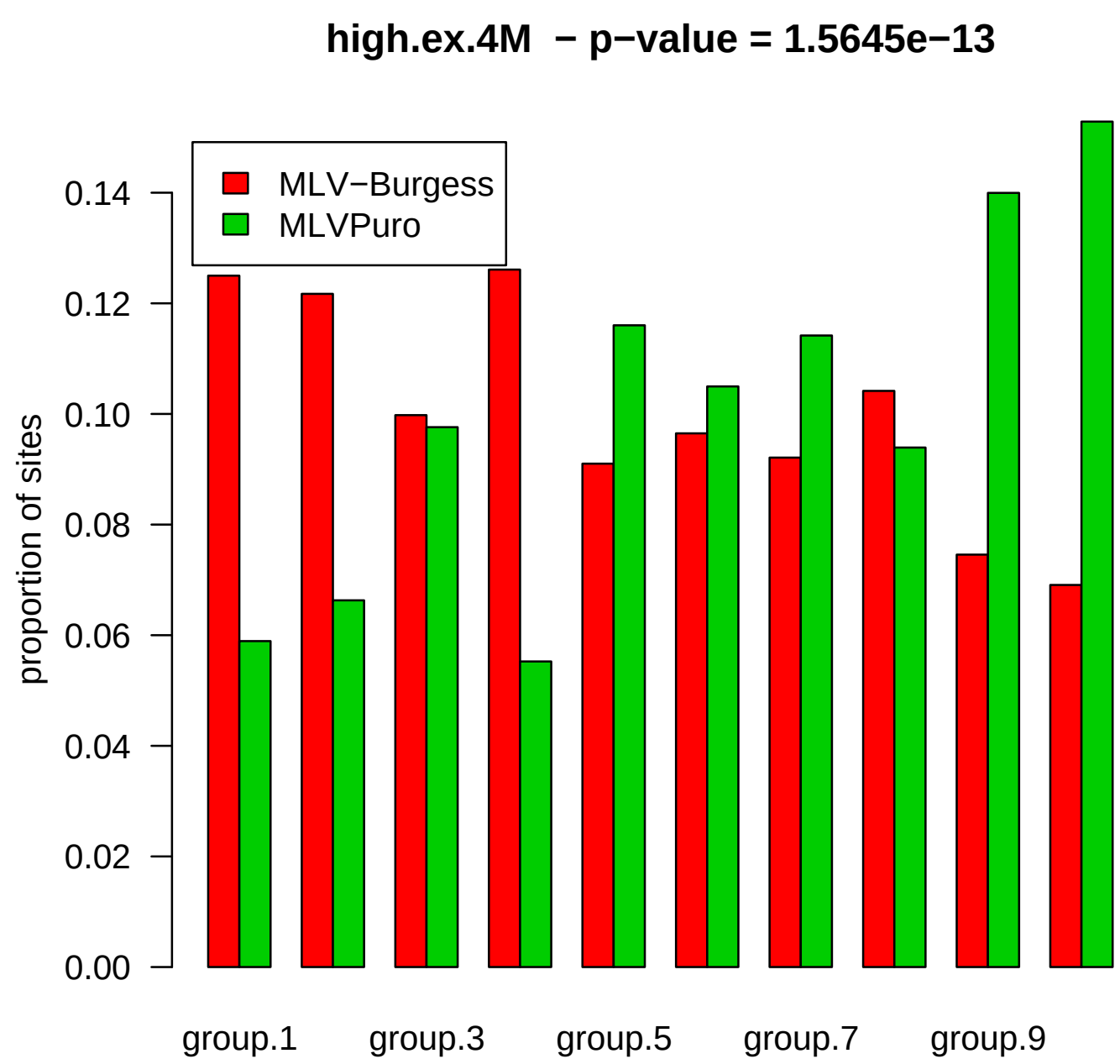

Here the effect of density of CpG islands is studied:

```
Category limits

          lower  category       upper
1  5.000000e-07   group.1 3.375000e-06
2  3.375000e-06   group.2 4.375000e-06
3  4.375000e-06   group.3 5.750000e-06
4  5.750000e-06   group.4 6.875000e-06
5  6.875000e-06   group.5 8.875000e-06
6  8.875000e-06   group.6 1.162500e-05
7  1.162500e-05   group.7 1.475000e-05
8  1.475000e-05   group.8 2.065000e-05
9  2.065000e-05   group.9 3.623310e-05
10 3.623310e-05  group.10 1.122037e-04
```

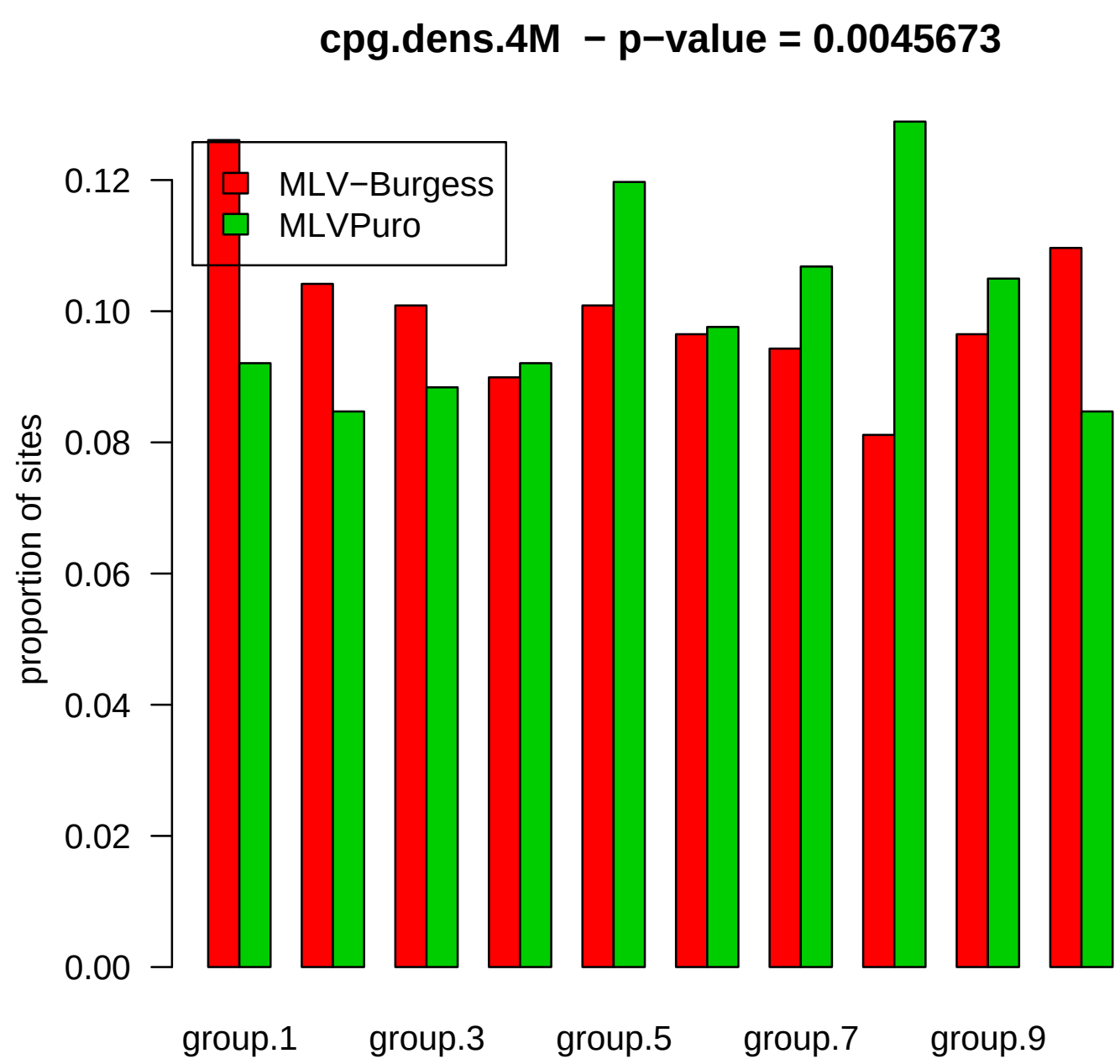

4.9 8 megaBase Window

In the barplot that follows we examine the association of insertion sites with expression density in a 8 megabase window surrounding each locus. First, we count just the number of genes on the represented on the chip.

```
 Category limits

          lower category        upper
1  2.083333e-07  group.1 1.636001e-06
2  1.636001e-06  group.2 2.194223e-06
3  2.194223e-06  group.3 2.689183e-06
4  2.689183e-06  group.4 3.294554e-06
5  3.294554e-06  group.5 4.201190e-06
6  4.201190e-06  group.6 5.327738e-06
7  5.327738e-06  group.7 6.808690e-06
8  6.808690e-06  group.8 8.450099e-06
9  8.450099e-06  group.9 1.359810e-05
10 1.359810e-05 group.10 2.813750e-05
```

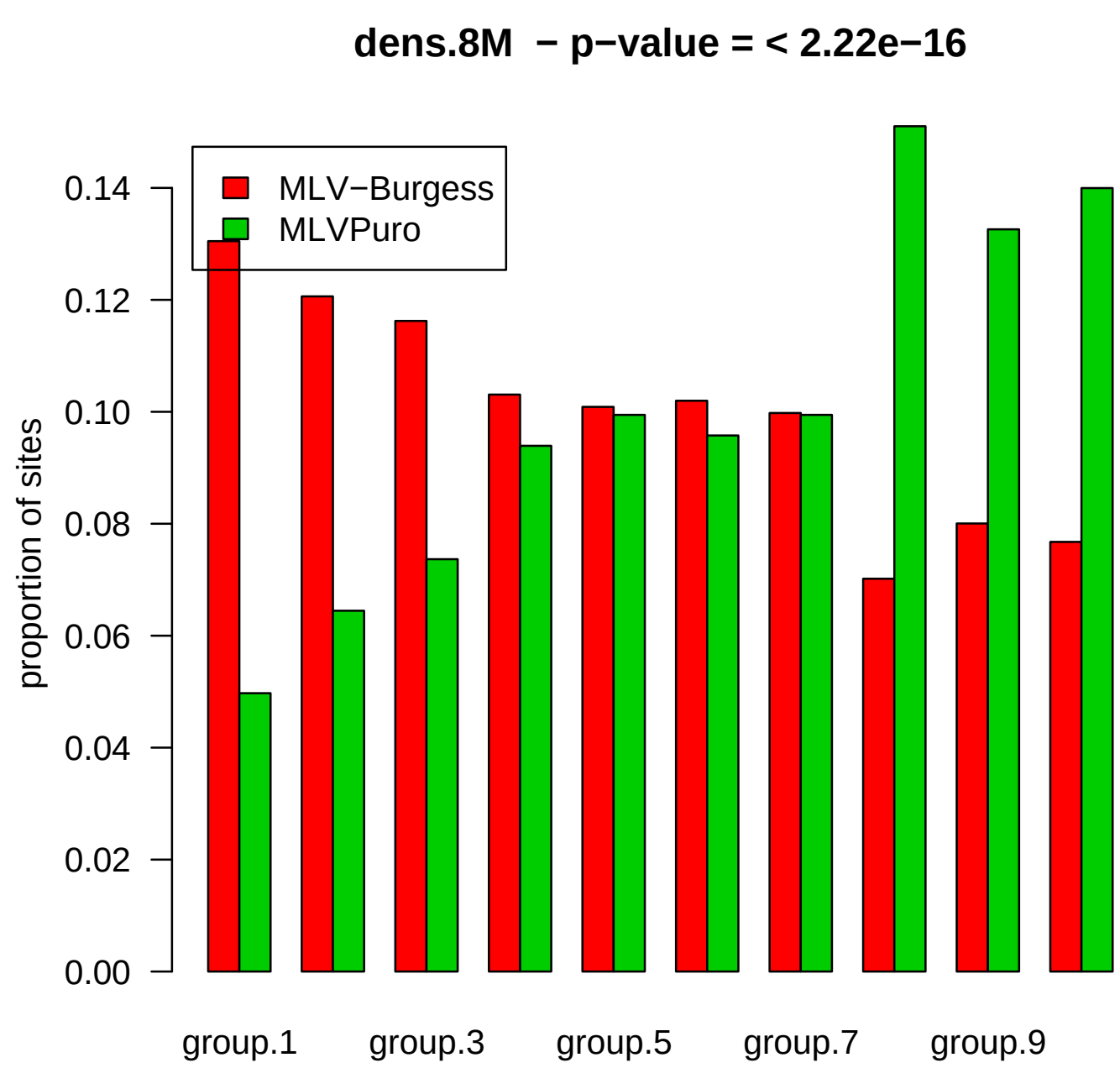

Here are the results for expression density. First, we count just genes that are in the upper half.

```
Category limits

           lower category        upper
1  6.250000e-08  group.1 7.002976e-07
2  7.002976e-07  group.2 8.975000e-07
3  8.975000e-07  group.3 1.178750e-06
4  1.178750e-06  group.4 1.489167e-06
5  1.489167e-06  group.5 1.870238e-06
6  1.870238e-06  group.6 2.560417e-06
7  2.560417e-06  group.7 3.239821e-06
8  3.239821e-06  group.8 4.160417e-06
9  4.160417e-06  group.9 6.275545e-06
10 6.275545e-06 group.10 1.238347e-05
```

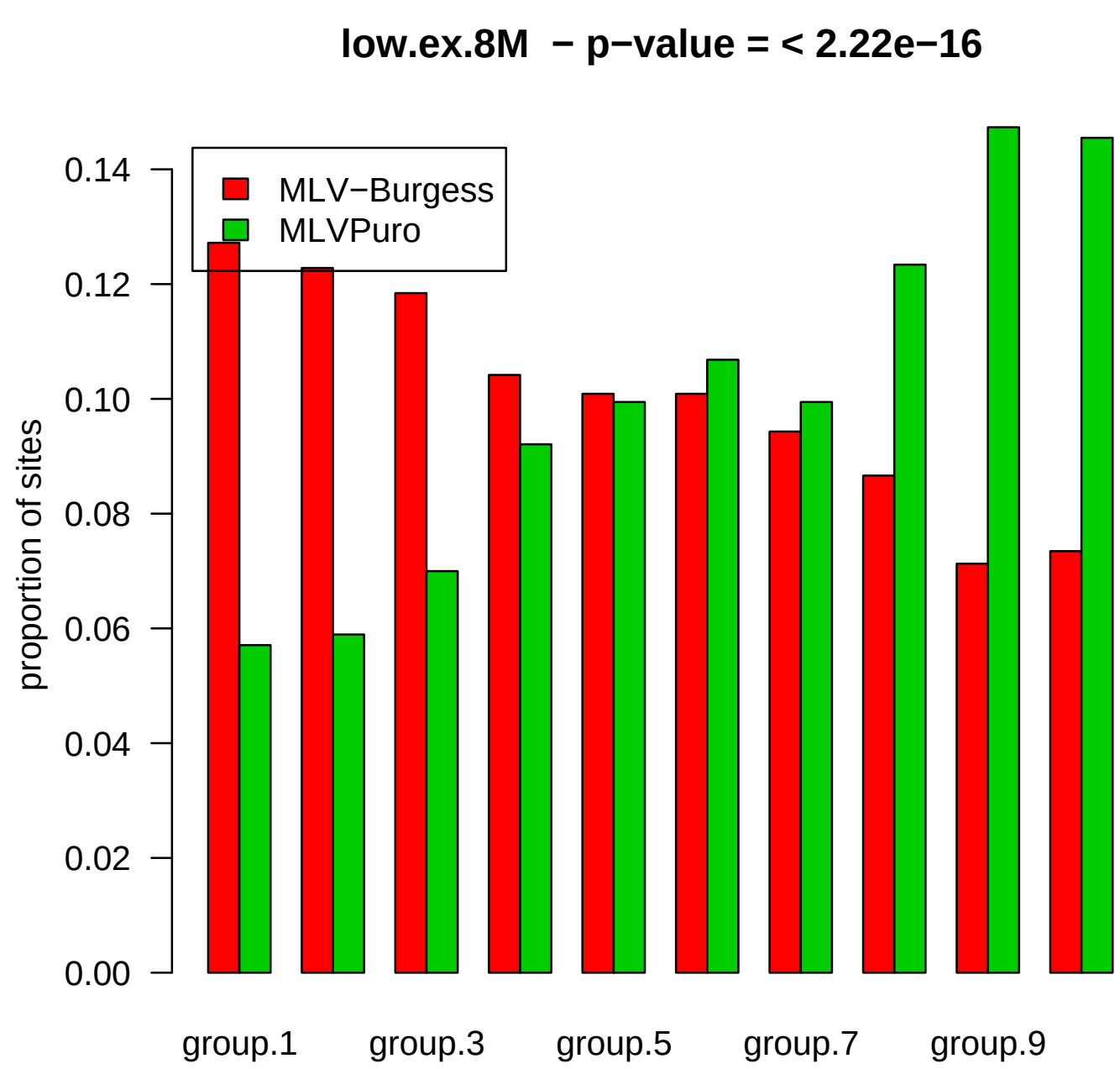

Now we count genes in the upper $1/8^{th}$:

```
Category limits

          lower  category        upper
1  0.000000e+00   group.1 2.812500e-07
2  2.812500e-07   group.2 4.410256e-07
3  4.410256e-07   group.3 5.937500e-07
4  5.937500e-07   group.4 7.908333e-07
5  7.908333e-07   group.5 1.008333e-06
6  1.008333e-06   group.6 1.300620e-06
7  1.300620e-06   group.7 1.674167e-06
8  1.674167e-06   group.8 2.164815e-06
9  2.164815e-06   group.9 3.285476e-06
10 3.285476e-06  group.10 6.927083e-06
```

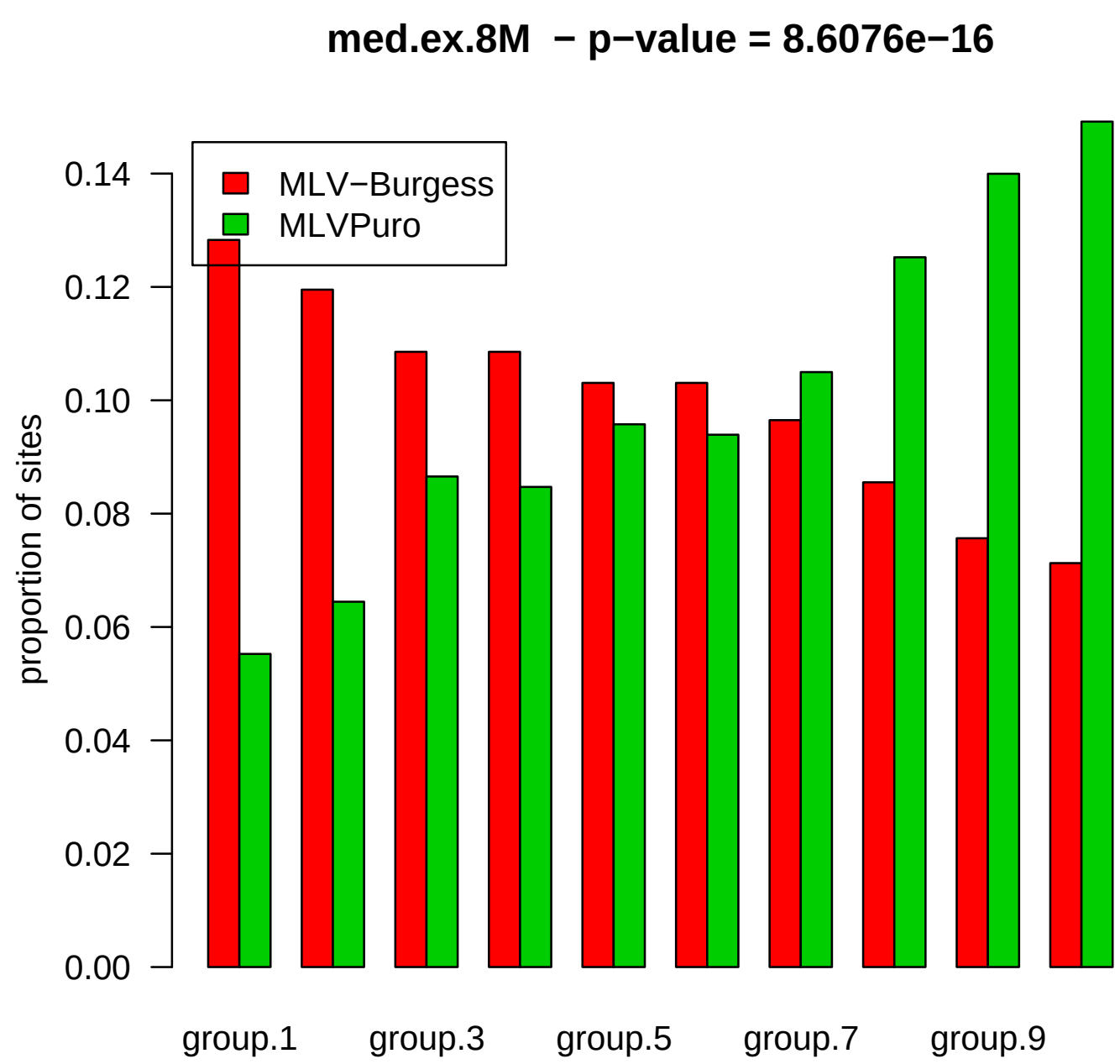

And here we count genes in the upper $1/16^{th}$:

```
Category limits

          lower category        upper
1  0.000000e+00  group.1 1.250000e-07
2  1.250000e-07  group.2 2.039881e-07
3  2.039881e-07  group.3 2.844290e-07
4  2.844290e-07  group.4 3.946429e-07
5  3.946429e-07  group.5 4.866071e-07
6  4.866071e-07  group.6 6.458333e-07
7  6.458333e-07  group.7 8.440164e-07
8  8.440164e-07  group.8 1.094014e-06
9  1.094014e-06  group.9 1.540278e-06
10 1.540278e-06 group.10 4.458333e-06
```

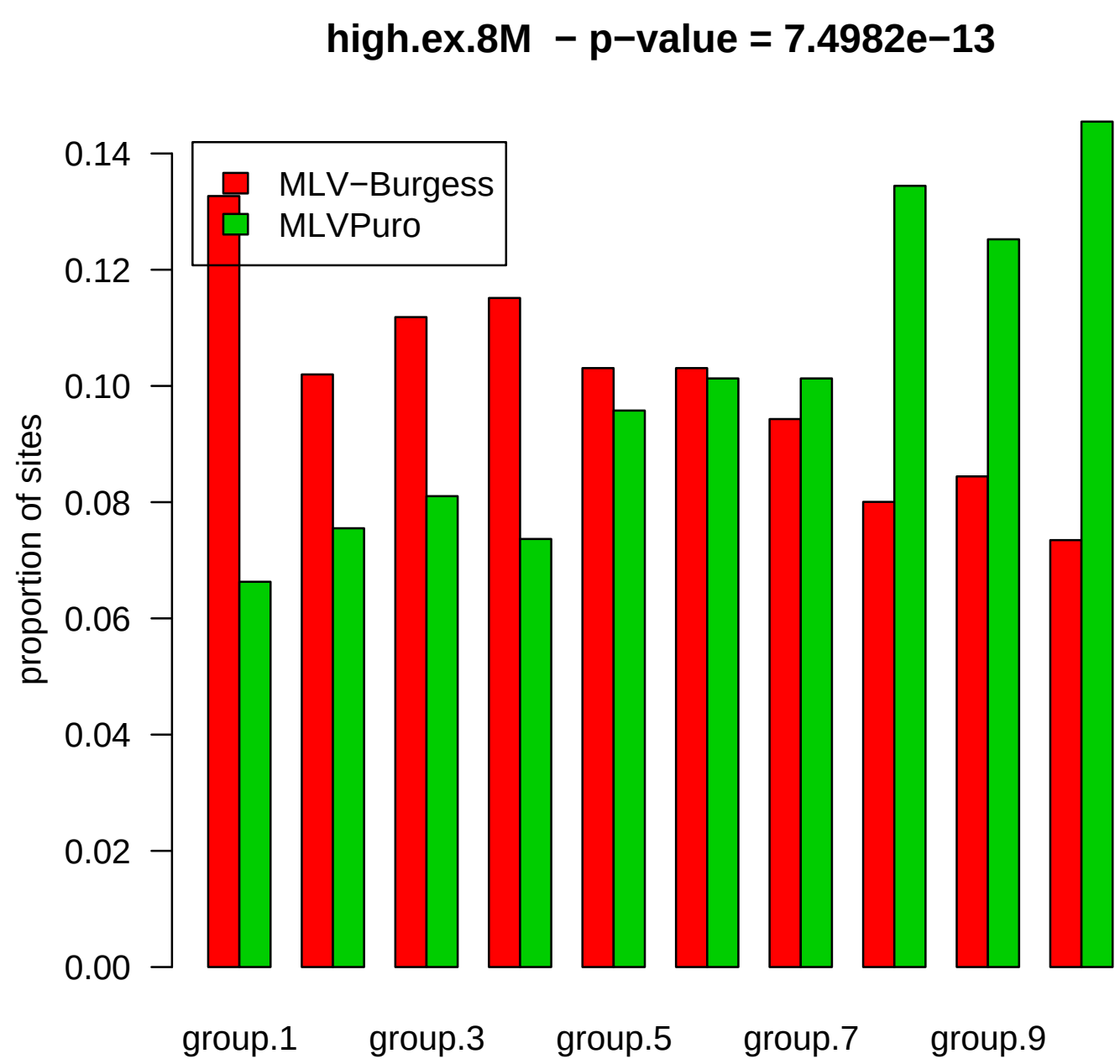

Here the effect of density of CpG islands is studied:

```
Category limits

           lower  category        upper
1  8.125000e-07   group.1 3.625000e-06
2  3.625000e-06   group.2 4.562500e-06
3  4.562500e-06   group.3 5.865175e-06
4  5.865175e-06   group.4 7.250000e-06
5  7.250000e-06   group.5 8.812500e-06
6  8.812500e-06   group.6 1.068750e-05
7  1.068750e-05   group.7 1.449859e-05
8  1.449859e-05   group.8 1.876250e-05
9  1.876250e-05   group.9 2.829574e-05
10 2.829574e-05 group.10 8.290307e-05
```

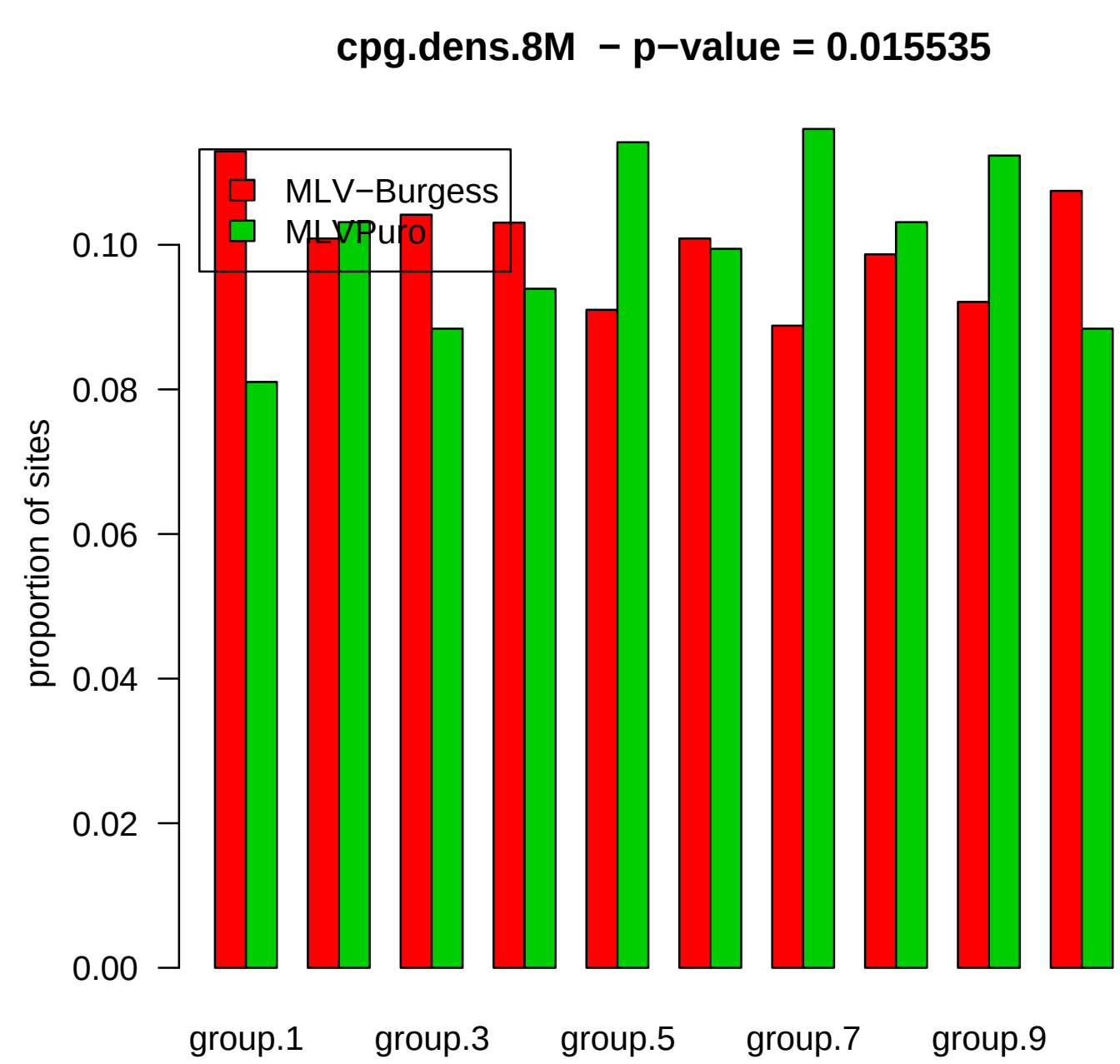

4.10 16 megaBase Window

In the barplot that follows we examine the association of insertion sites with expression density in a 16 megabase window surrounding each locus. First, we count just the number of genes on the represented on the chip.

```
 Category limits

          lower category        upper
1  6.166667e-07  group.1 1.857909e-06
2  1.857909e-06  group.2 2.288328e-06
3  2.288328e-06  group.3 2.728423e-06
4  2.728423e-06  group.4 3.297317e-06
5  3.297317e-06  group.5 3.984970e-06
6  3.984970e-06  group.6 4.808304e-06
7  4.808304e-06  group.7 6.038077e-06
8  6.038077e-06  group.8 7.897006e-06
9  7.897006e-06  group.9 1.119436e-05
10 1.119436e-05 group.10 1.928929e-05
```

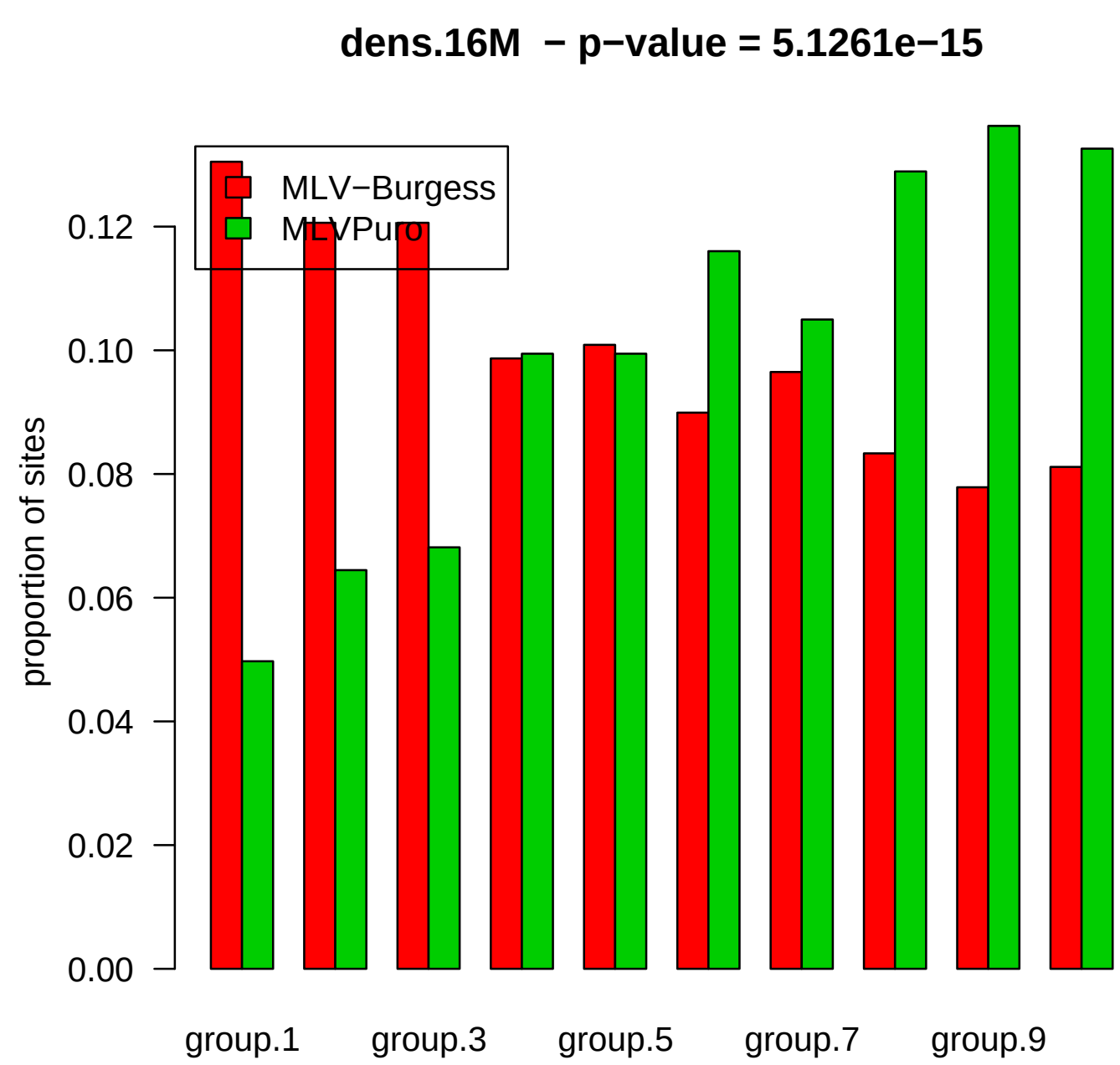

Here are the results for expression density. First, we count just genes that are in the upper half.

```
 Category limits

           lower category        upper
1   1.666667e-07  group.1 7.923160e-07
2   7.923160e-07  group.2 9.843750e-07
3   9.843750e-07  group.3 1.193839e-06
4   1.193839e-06  group.4 1.406250e-06
5   1.406250e-06  group.5 1.859598e-06
6   1.859598e-06  group.6 2.321042e-06
7   2.321042e-06  group.7 2.761047e-06
8   2.761047e-06  group.8 3.579107e-06
9   3.579107e-06  group.9 5.516101e-06
10  5.516101e-06 group.10 9.091874e-06
```

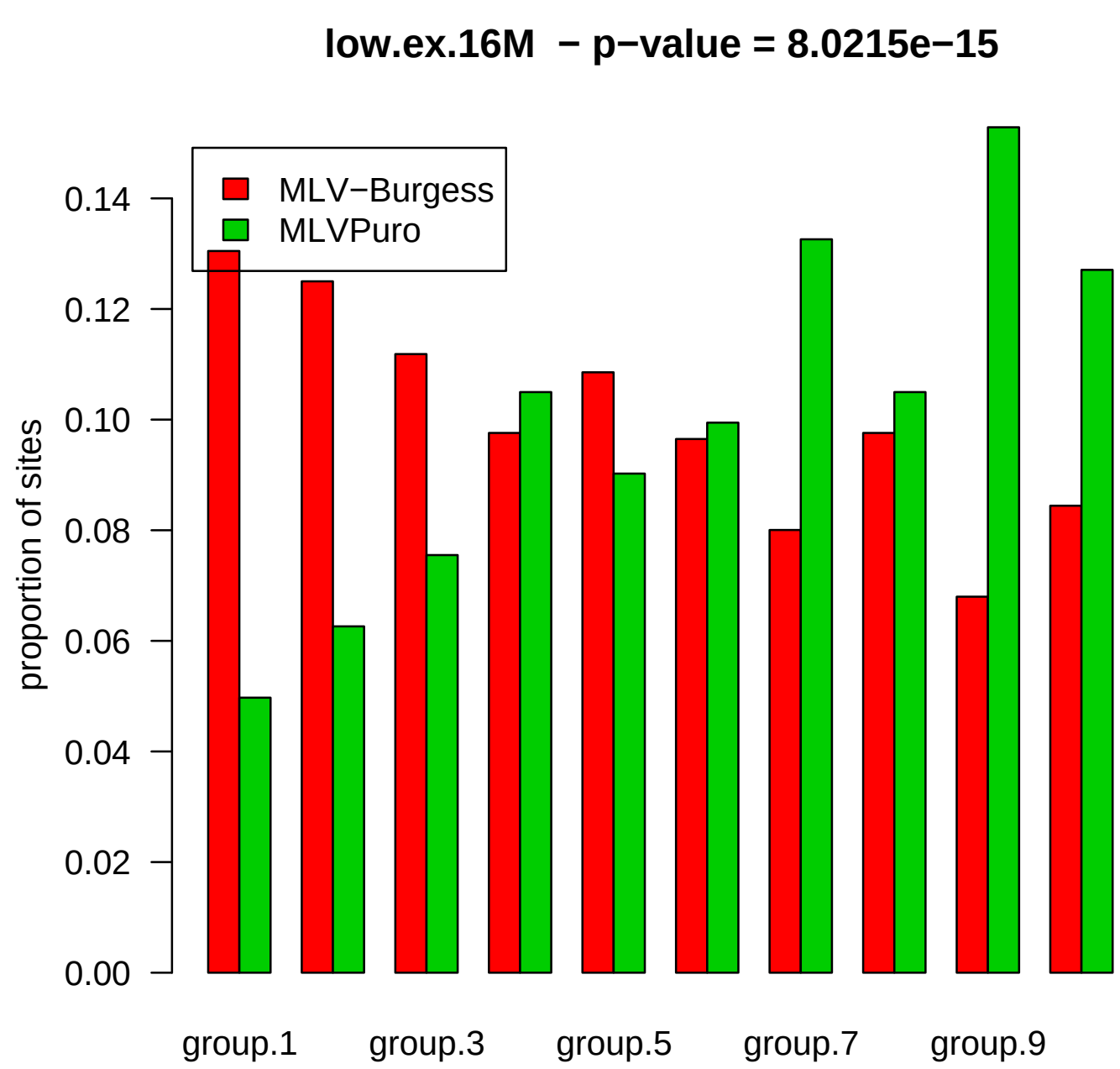

Now we count genes in the upper $1/8^{th}$:

```
Category limits

          lower category        upper
1  6.250000e-08  group.1 3.498512e-07
2  3.498512e-07  group.2 4.889583e-07
3  4.889583e-07  group.3 6.076306e-07
4  6.076306e-07  group.4 7.614583e-07
5  7.614583e-07  group.5 9.724702e-07
6  9.724702e-07  group.6 1.186458e-06
7  1.186458e-06  group.7 1.431250e-06
8  1.431250e-06  group.8 1.899306e-06
9  1.899306e-06  group.9 2.853750e-06
10 2.853750e-06 group.10 4.848850e-06
```

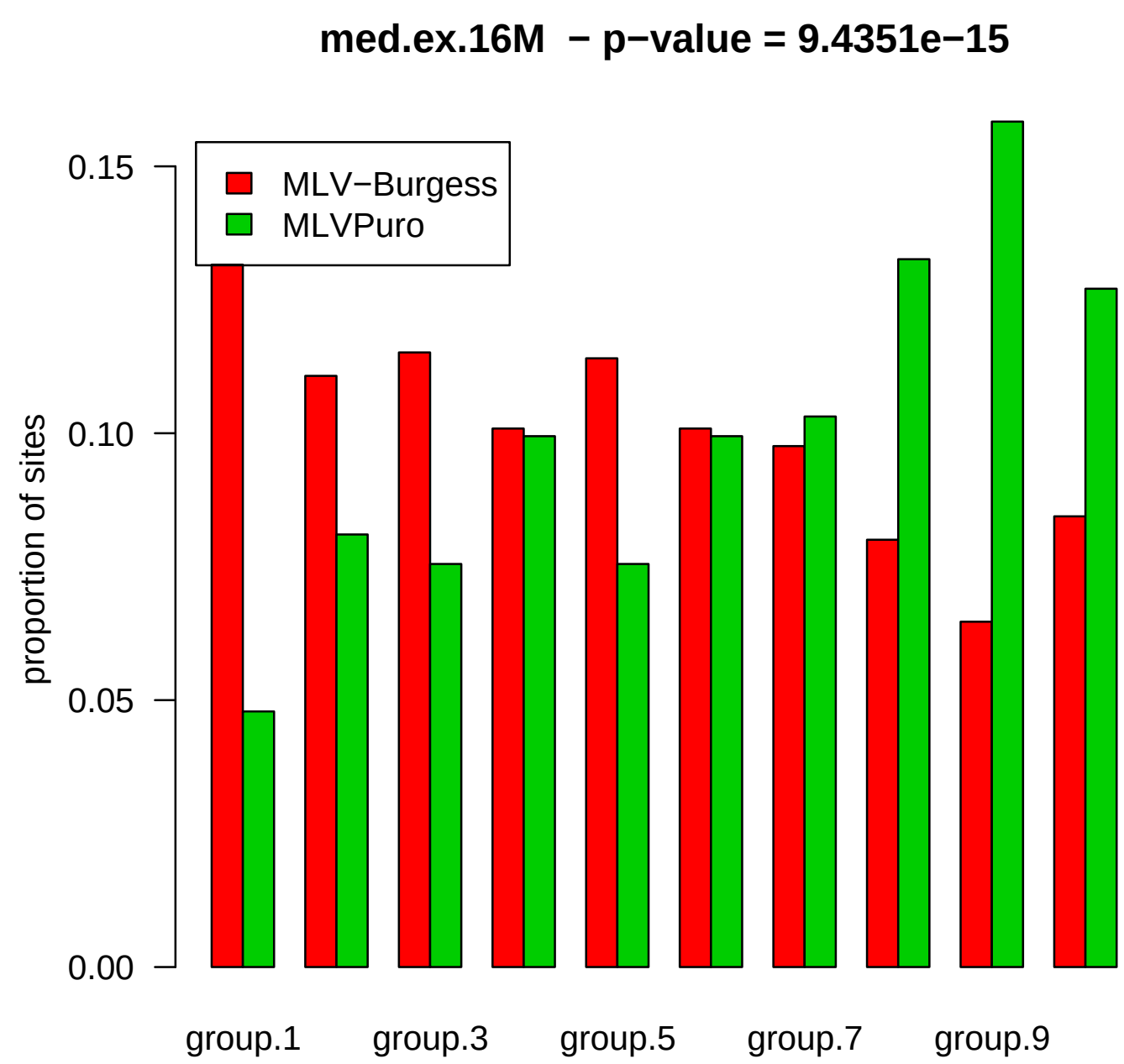

And here we count genes in the upper $1/16^{th}$:

```
Category limits

          lower category        upper
1  0.000000e+00  group.1 1.583333e-07
2  1.583333e-07  group.2 2.447917e-07
3  2.447917e-07  group.3 3.160417e-07
4  3.160417e-07  group.4 3.906250e-07
5  3.906250e-07  group.5 4.812500e-07
6  4.812500e-07  group.6 5.795833e-07
7  5.795833e-07  group.7 7.750000e-07
8  7.750000e-07  group.8 9.510417e-07
9  9.510417e-07  group.9 1.339583e-06
10 1.339583e-06 group.10 2.718750e-06
```

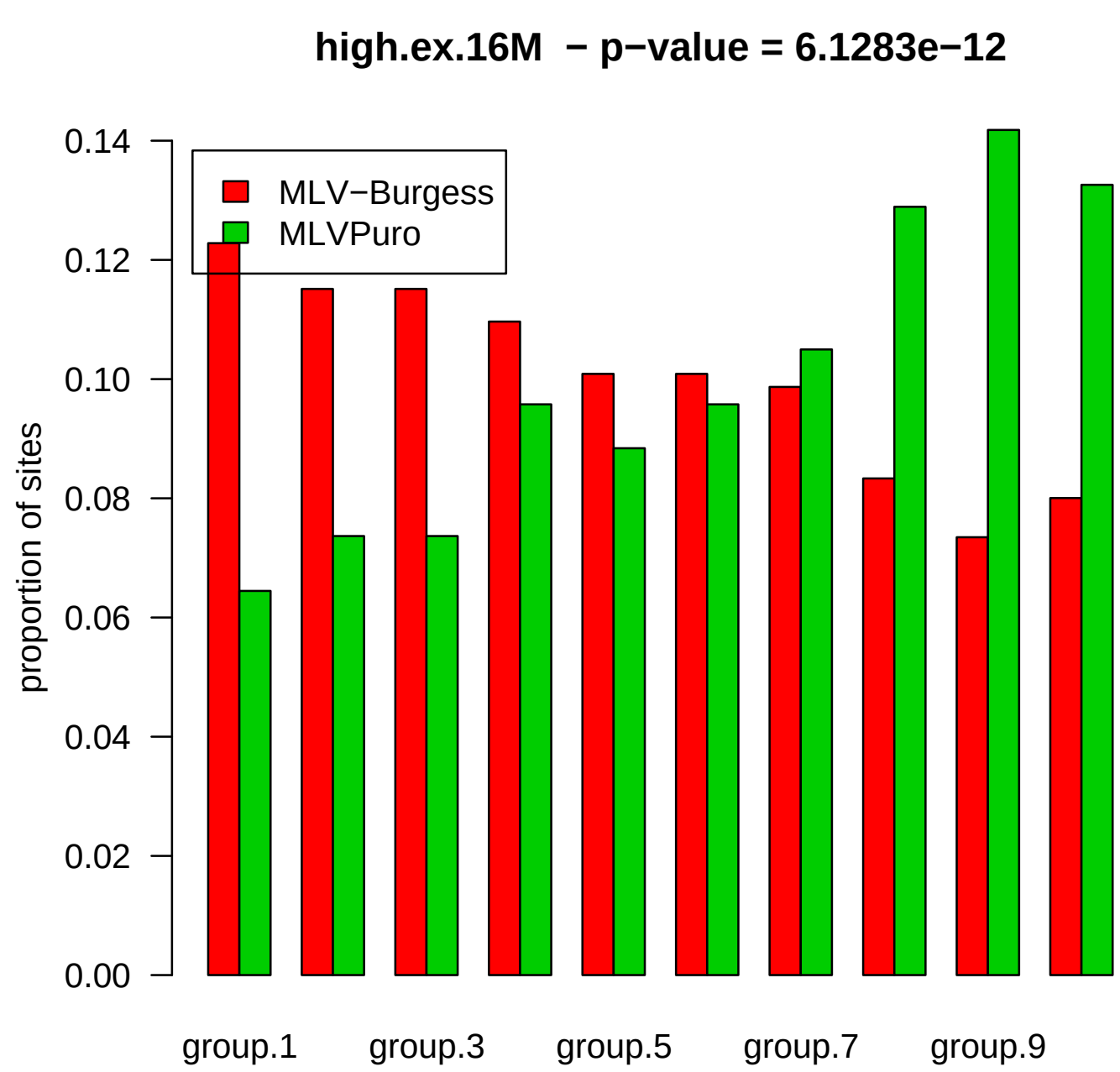

Here the effect of density of CpG islands is studied:

```
Category limits

          lower  category        upper
1  1.312500e-06   group.1 4.020451e-06
2  4.020451e-06   group.2 5.156250e-06
3  5.156250e-06   group.3 6.093750e-06
4  6.093750e-06   group.4 7.250000e-06
5  7.250000e-06   group.5 8.638967e-06
6  8.638967e-06   group.6 1.065000e-05
7  1.065000e-05   group.7 1.324375e-05
8  1.324375e-05   group.8 1.729446e-05
9  1.729446e-05   group.9 2.161923e-05
10 2.161923e-05  group.10 6.622230e-05
```

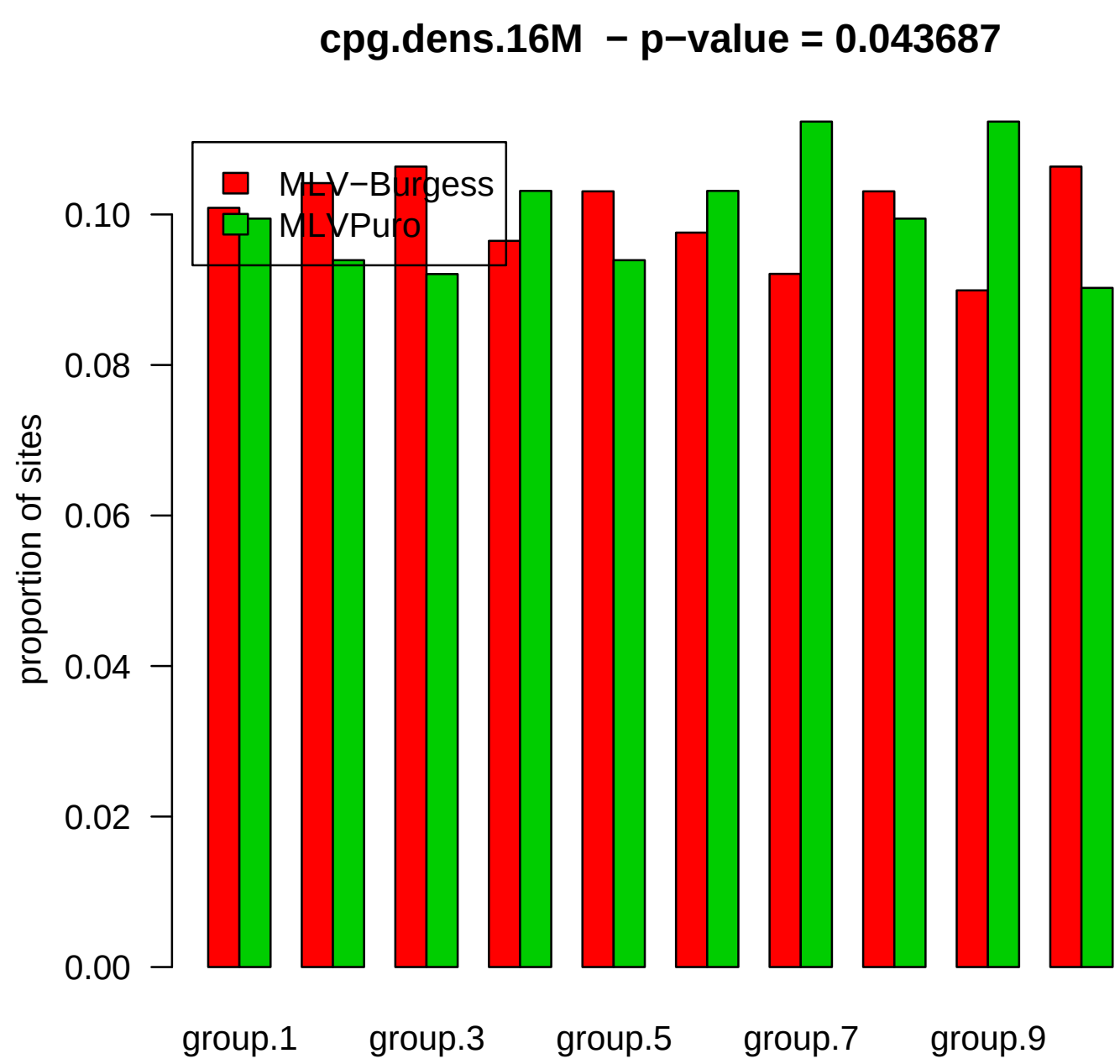

4.11 32 megaBase Window

In the barplot that follows we examine the association of insertion sites with expression density in a 32 megabase window surrounding each locus. First, we count just the number of genes on the represented on the chip.

```
 Category limits

          lower category        upper
1  6.308649e-07  group.1 1.911353e-06
2  1.911353e-06  group.2 2.404606e-06
3  2.404606e-06  group.3 2.775409e-06
4  2.775409e-06  group.4 3.330213e-06
5  3.330213e-06  group.5 3.968424e-06
6  3.968424e-06  group.6 4.736310e-06
7  4.736310e-06  group.7 5.246540e-06
8  5.246540e-06  group.8 6.878760e-06
9  6.878760e-06  group.9 8.908811e-06
10 8.908811e-06 group.10 1.858283e-05
```

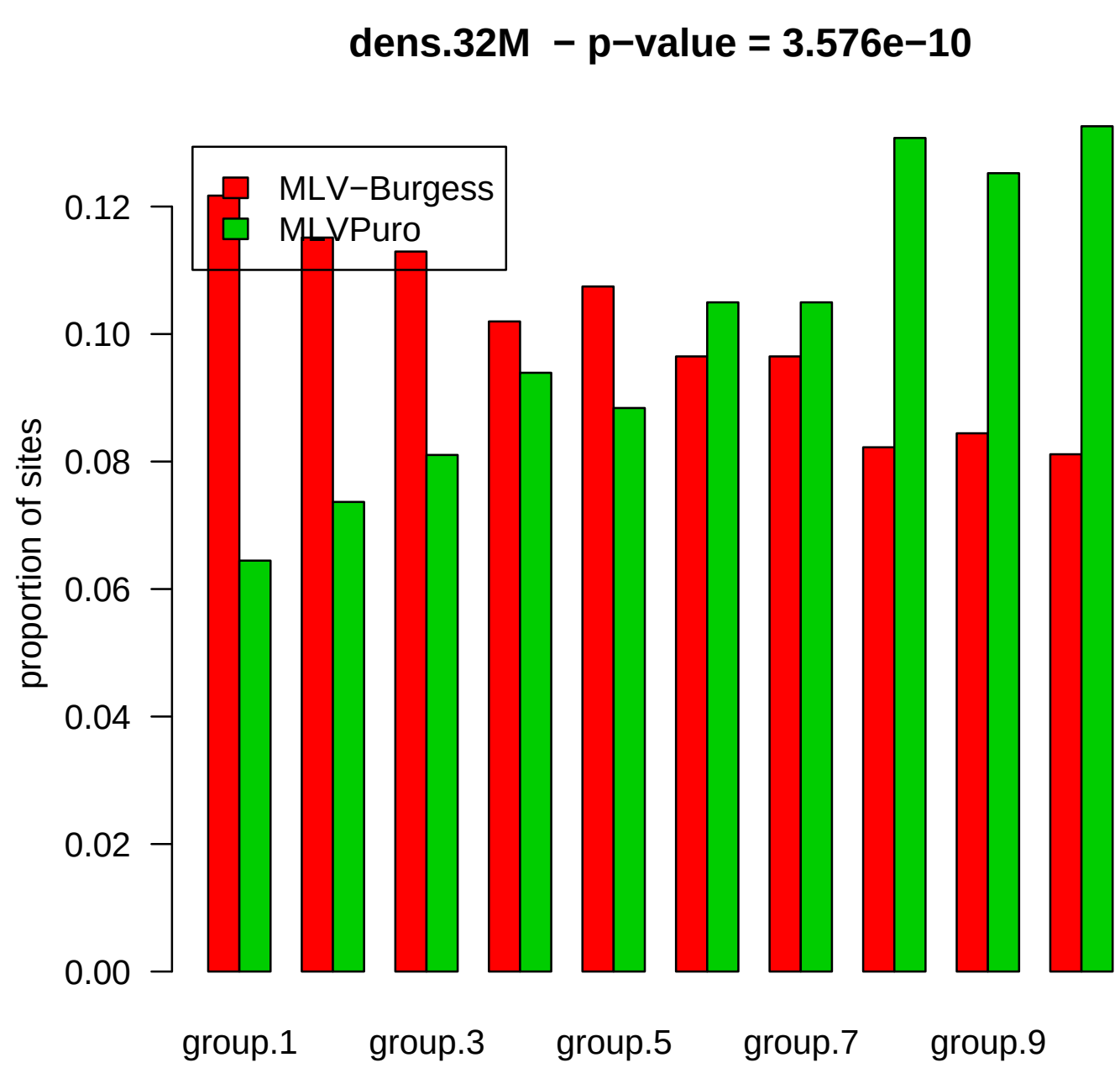

Here are the results for expression density. First, we count just genes that are in the upper half.

```
 Category limits

          lower category        upper
1  2.794834e-07  group.1 8.289446e-07
2  8.289446e-07  group.2 1.060074e-06
3  1.060074e-06  group.3 1.252016e-06
4  1.252016e-06  group.4 1.472961e-06
5  1.472961e-06  group.5 1.736979e-06
6  1.736979e-06  group.6 2.105536e-06
7  2.105536e-06  group.7 2.529449e-06
8  2.529449e-06  group.8 3.353331e-06
9  3.353331e-06  group.9 4.258287e-06
10 4.258287e-06 group.10 8.892661e-06
```

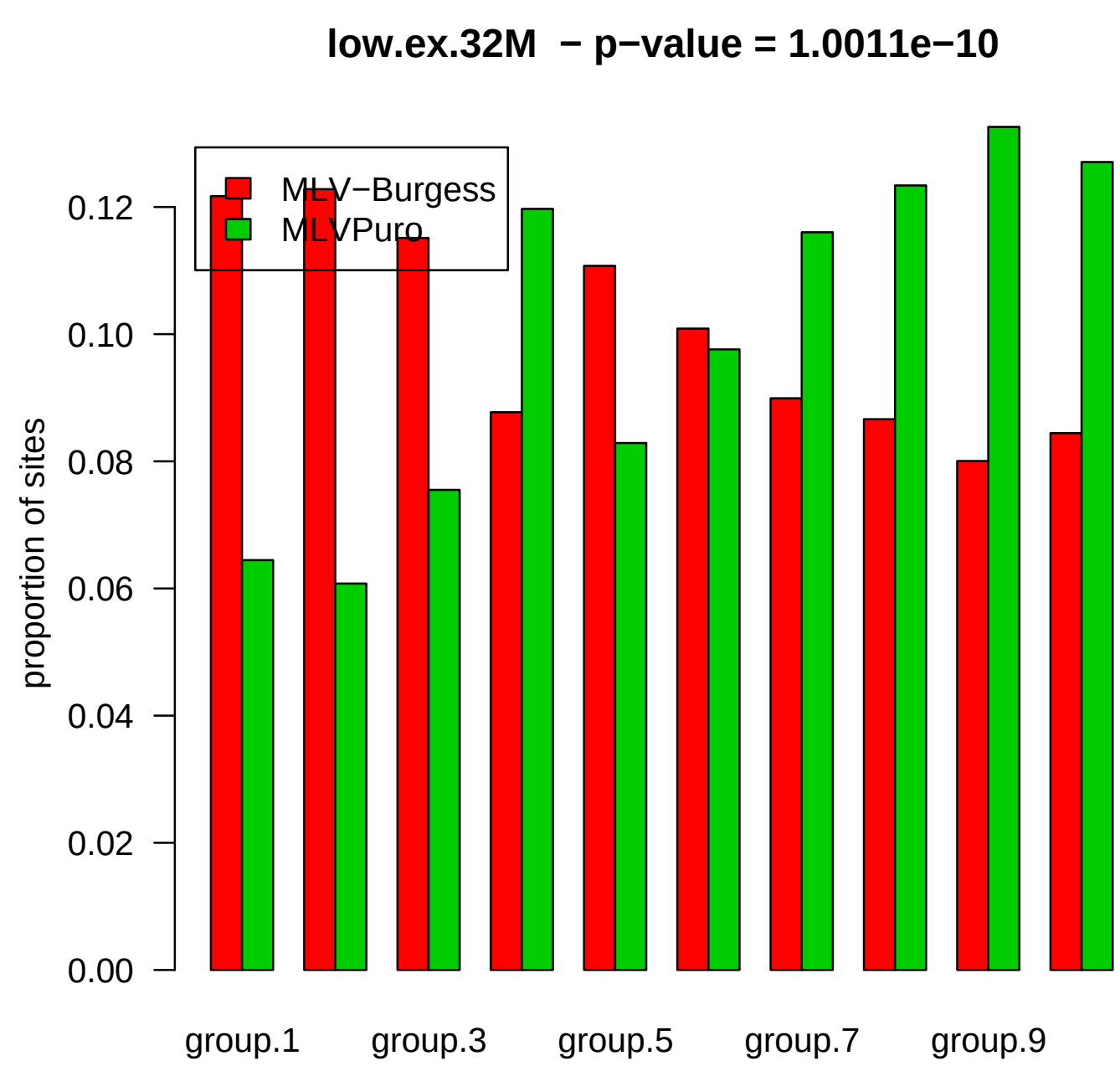

Now we count genes in the upper $1/8^{th}$:

```
Category limits

          lower  category        upper
1  1.551484e-07   group.1 4.047619e-07
2  4.047619e-07   group.2 5.375000e-07
3  5.375000e-07   group.3 6.435583e-07
4  6.435583e-07   group.4 7.867708e-07
5  7.867708e-07   group.5 9.072917e-07
6  9.072917e-07   group.6 1.078542e-06
7  1.078542e-06   group.7 1.315626e-06
8  1.315626e-06   group.8 1.793056e-06
9  1.793056e-06   group.9 2.168327e-06
10 2.168327e-06  group.10 4.529922e-06
```

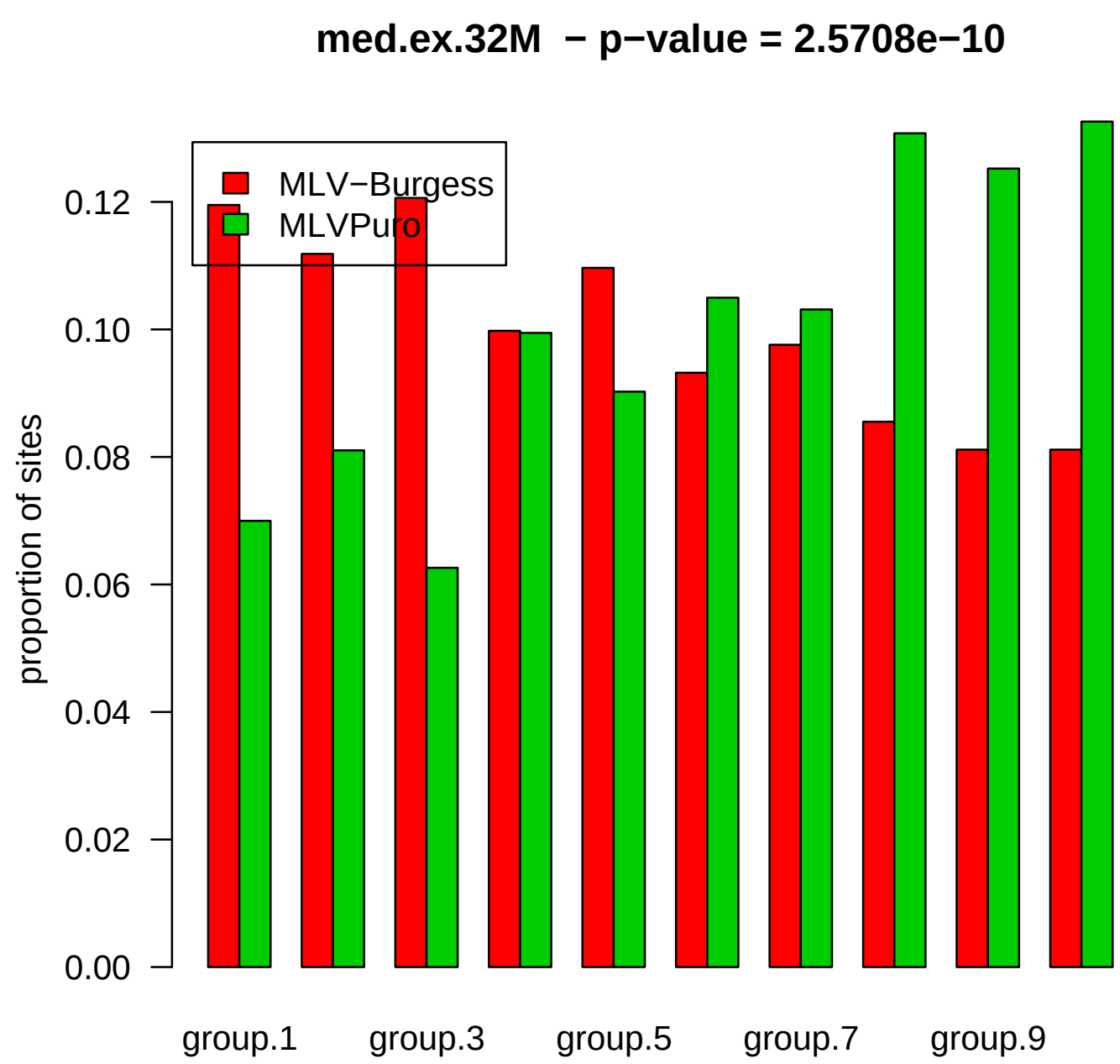

And here we count genes in the upper $1/16^{th}$:

```
Category limits

          lower  category         upper
1  5.202368e-08   group.1 2.008088e-07
2  2.008088e-07   group.2 2.716076e-07
3  2.716076e-07   group.3 3.322716e-07
4  3.322716e-07   group.4 3.939286e-07
5  3.939286e-07   group.5 4.685497e-07
6  4.685497e-07   group.6 5.973958e-07
7  5.973958e-07   group.7 7.083723e-07
8  7.083723e-07   group.8 8.227806e-07
9  8.227806e-07   group.9 9.995536e-07
10 9.995536e-07  group.10 2.177009e-06
```

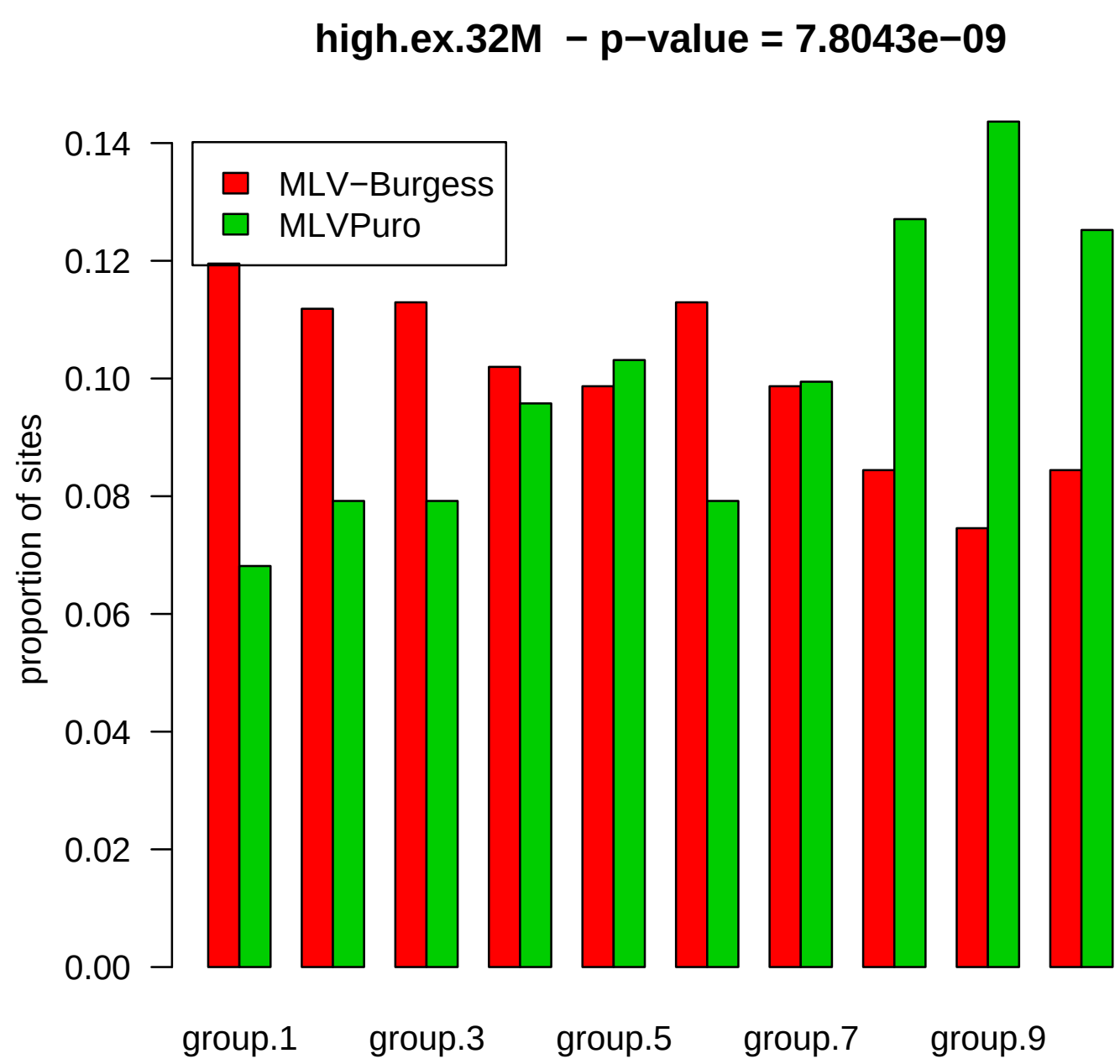

Here the effect of density of CpG islands is studied:

```
Category limits

          lower category        upper
1  3.000000e-06  group.1 4.609375e-06
2  4.609375e-06  group.2 5.531250e-06
3  5.531250e-06  group.3 6.406250e-06
4  6.406250e-06  group.4 7.179893e-06
5  7.179893e-06  group.5 7.896610e-06
6  7.896610e-06  group.6 9.633538e-06
7  9.633538e-06  group.7 1.148158e-05
8  1.148158e-05  group.8 1.508771e-05
9  1.508771e-05  group.9 1.917786e-05
10 1.917786e-05 group.10 3.888930e-05
```

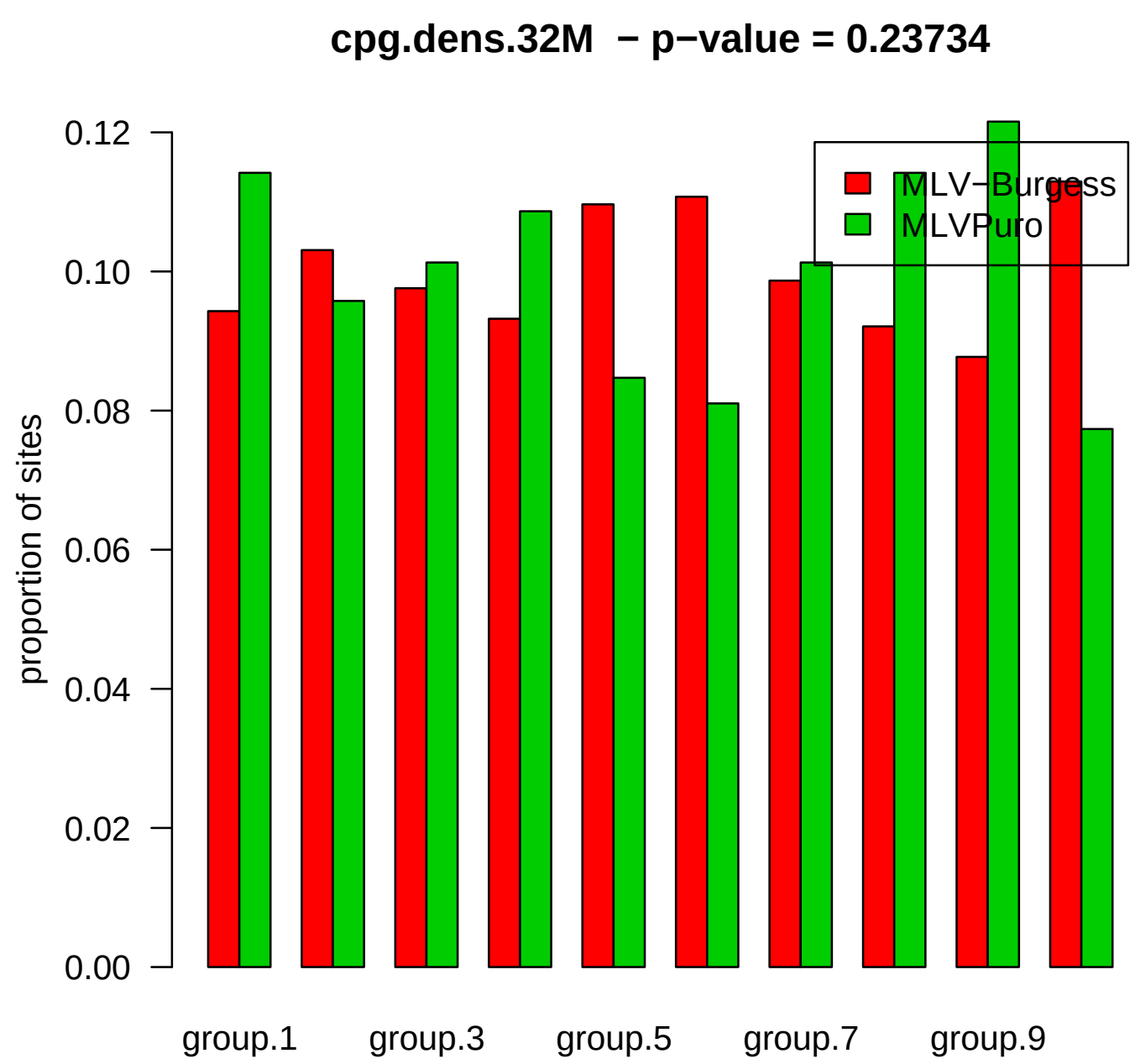

5 Juxtaposition with Gene Start and End Positions

5.1 Acembly Annotations

In this section we study the effect of juxtaposition in terms of gene start and end positions. The first barplot shows the effect of gene width for those insertions that are located within an Acembly gene.

```
 Category limits

      lower category     upper
1     326.0  group.1    8389.5
2    8389.5  group.2   18419.0
3   18419.0  group.3   28768.0
4   28768.0  group.4   44126.0
5   44126.0  group.5   66097.5
6   66097.5  group.6   94890.0
7   94890.0  group.7  130663.0
8  130663.0  group.8  197164.0
9  197164.0  group.9  310763.0
10 310763.0 group.10 1468602.0
```

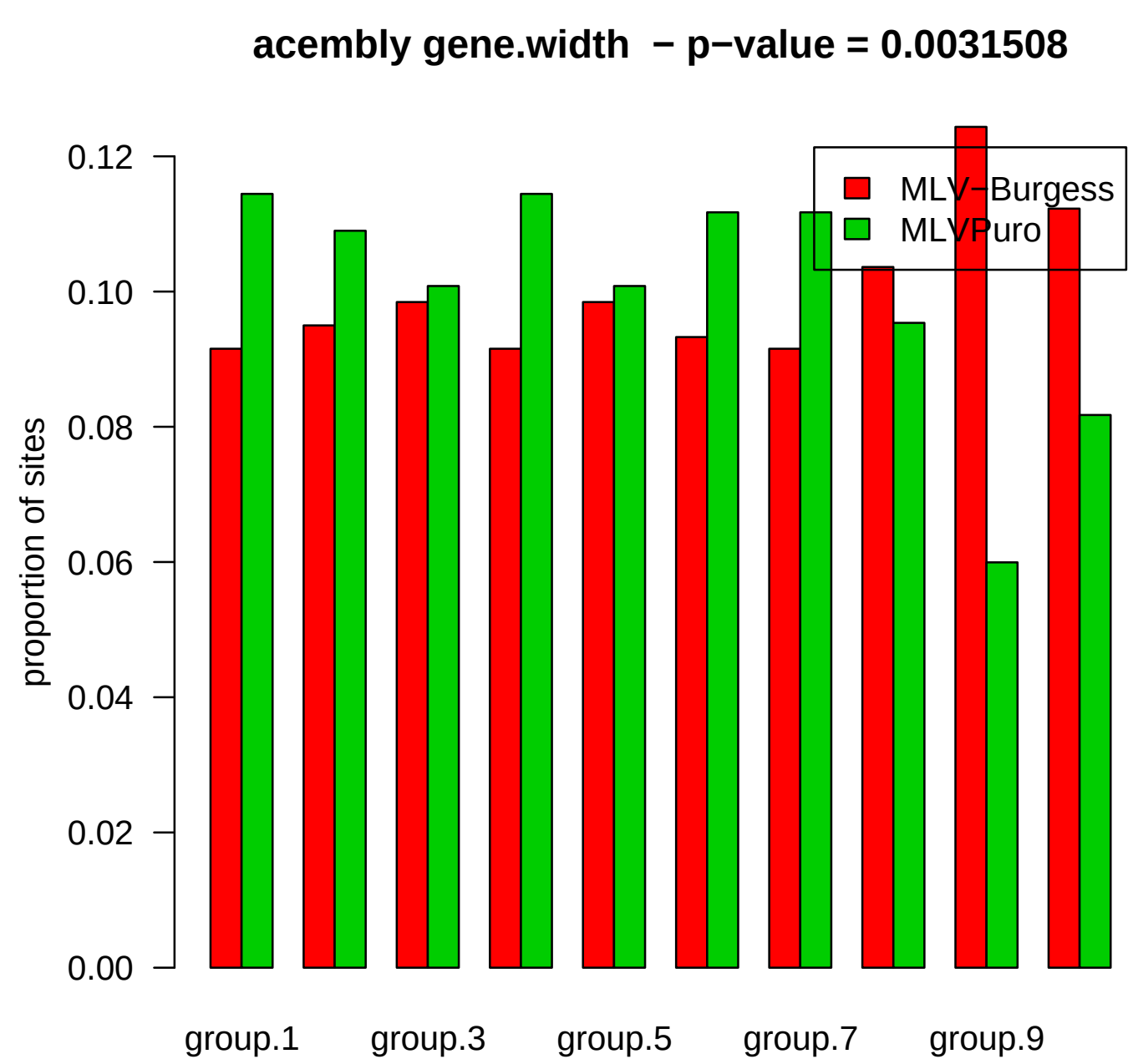

The next plot uses the width of a non-gene region for insertions that fall into such regions.

```
 Category limits

      lower category    upper
1     598.0  group.1   5814.0
2    5814.0  group.2  14692.0
3   14692.0  group.3  23137.8
4   23137.8  group.4  31492.4
5   31492.4  group.5  41490.0
6   41490.0  group.6  65738.0
7   65738.0  group.7  87563.0
8   87563.0  group.8 136570.4
9  136570.4  group.9 214759.0
10 214759.0 group.10 733739.0
```

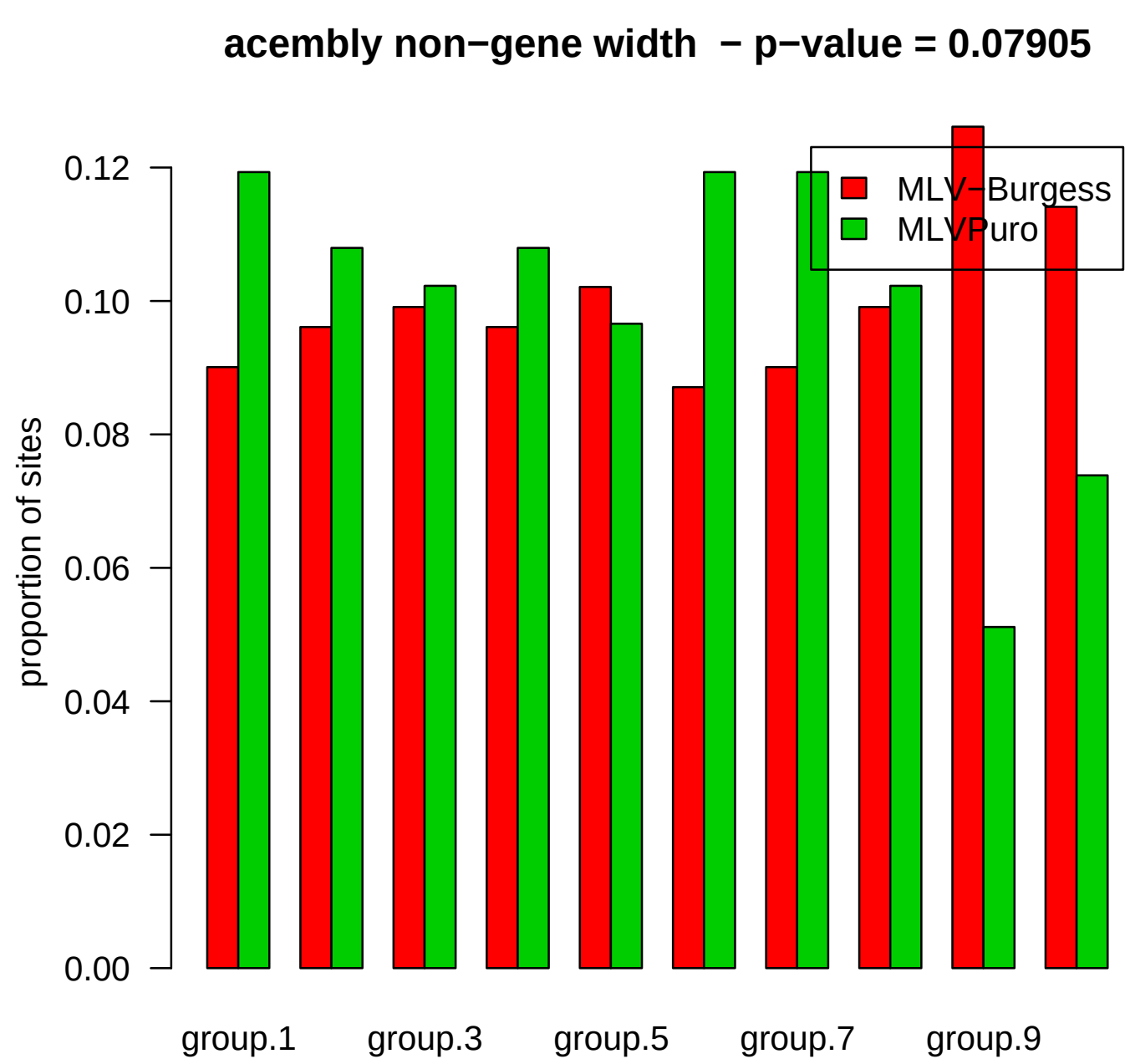

The next plot studies the distance to the nearest boundary between a gene and a non-gene region. The distance is expressed as a fraction of the length of the region. Thus, '0.25' refers to one quarter of the distance from the site to nearest boundary divided by the total width of the region.

```
 Category limits

         lower category      upper
1  0.0001629966  group.1 0.02422400
2  0.0242239990  group.2 0.06169855
3  0.0616985504  group.3 0.10831464
4  0.1083146372  group.4 0.15879184
5  0.1587918380  group.5 0.21012944
6  0.2101294427  group.6 0.26253887
7  0.2625388740  group.7 0.32510730
8  0.3251073039  group.8 0.38227968
9  0.3822796821  group.9 0.43986921
10 0.4398692059 group.10 0.49962244
```

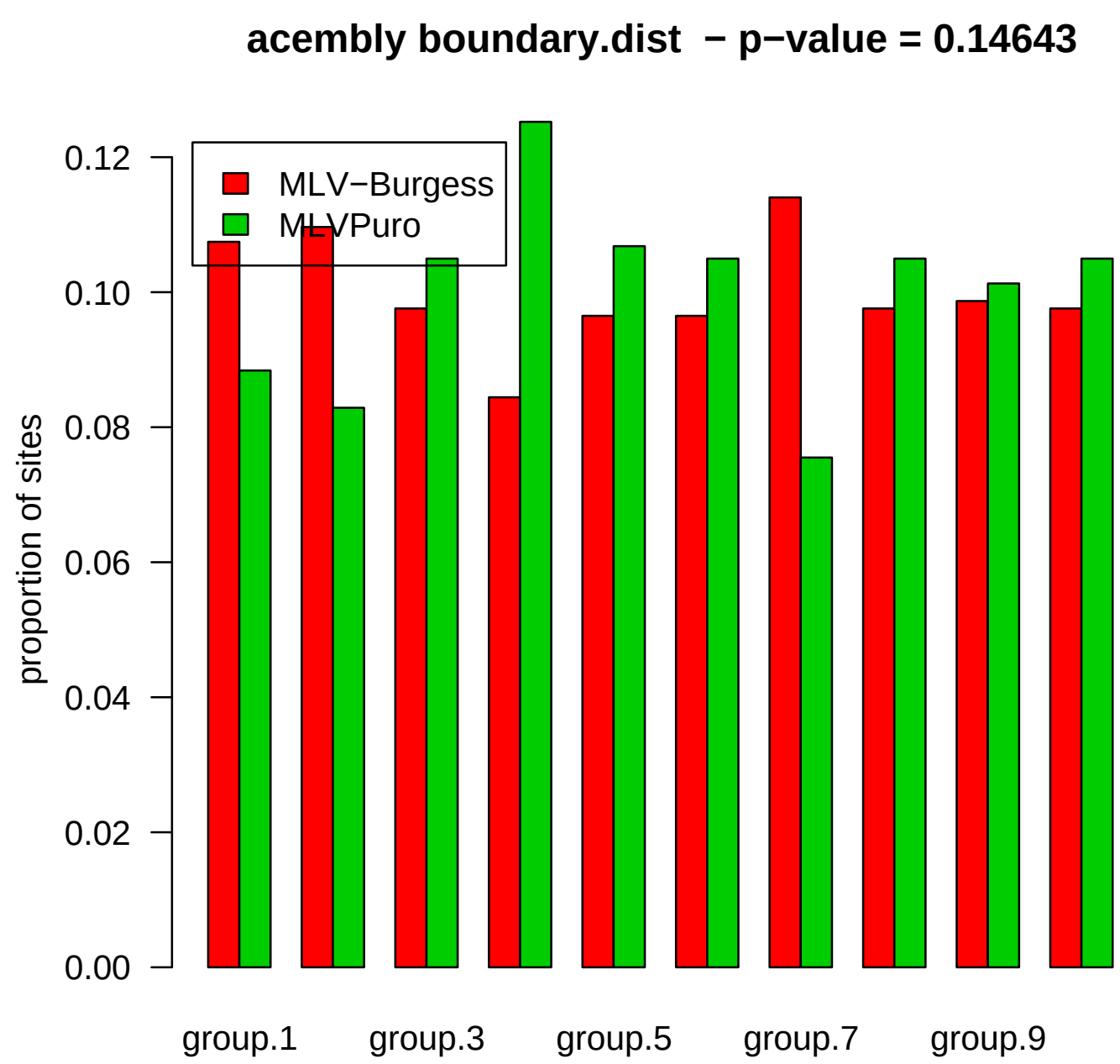

This plot studies the effect of nearness to the beginning of a transcript. For sites in genes, it is the distance to the start of the gene divided by the width of the gene. For other sites it is the distance from the site to the nearer gene if that gene boundary is also a transcription starting point. Locations near '0' are relatively near the beginning of transcription, while those near '1' are near the termination of the transcript.

```
 Category limits

          lower category      upper
1  0.0001629966  group.1 0.03194402
2  0.0319440186  group.2 0.10429321
3  0.1042932149  group.3 0.18197543
4  0.1819754324  group.4 0.27825009
5  0.2782500880  group.5 0.36999400
6  0.3699939971  group.6 0.47428155
7  0.4742815470  group.7 0.60099307
8  0.6009930664  group.8 0.74663747
9  0.7466374667  group.9 0.86910984
10 0.8691098371 group.10 0.99970222
```

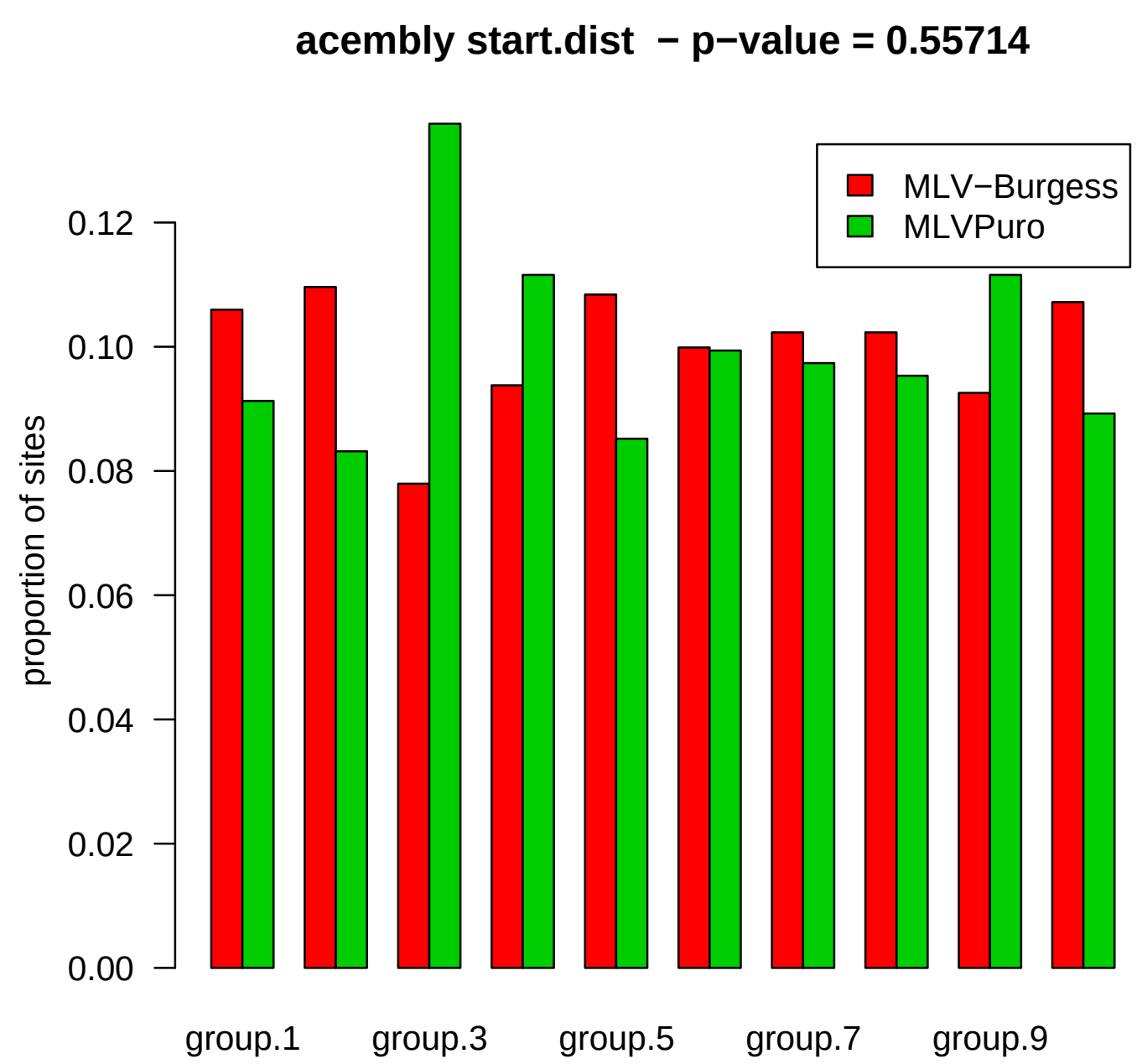

5.2 RefSeq Annotations

Category limits

	lower	category	upper
1	437.0	group.1	15657.2
2	15657.2	group.2	29811.6
3	29811.6	group.3	53185.2
4	53185.2	group.4	73121.2
5	73121.2	group.5	102946.0
6	102946.0	group.6	137449.4
7	137449.4	group.7	183002.4
8	183002.4	group.8	248874.0
9	248874.0	group.9	388421.4
10	388421.4	group.10	1468599.0

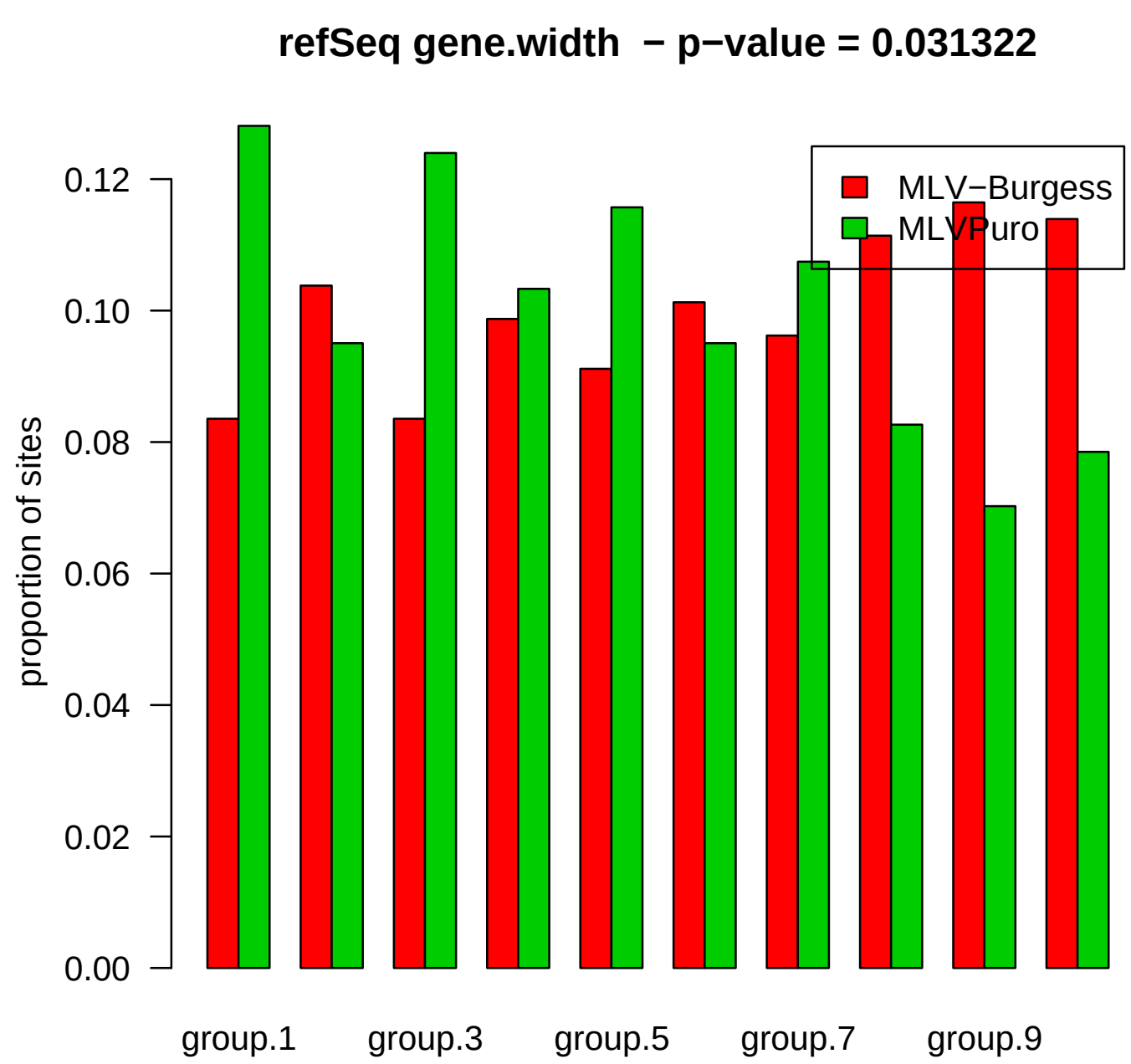

Category limits

	lower	category	upper
1	720.0	group.1	16279.7
2	16279.7	group.2	36052.6
3	36052.6	group.3	60577.1
4	60577.1	group.4	90361.2

5	90361.2	group.5	142155.5
6	142155.5	group.6	245814.6
7	245814.6	group.7	382797.0
8	382797.0	group.8	619811.6
9	619811.6	group.9	1274970.3
10	1274970.3	group.10	5474543.0

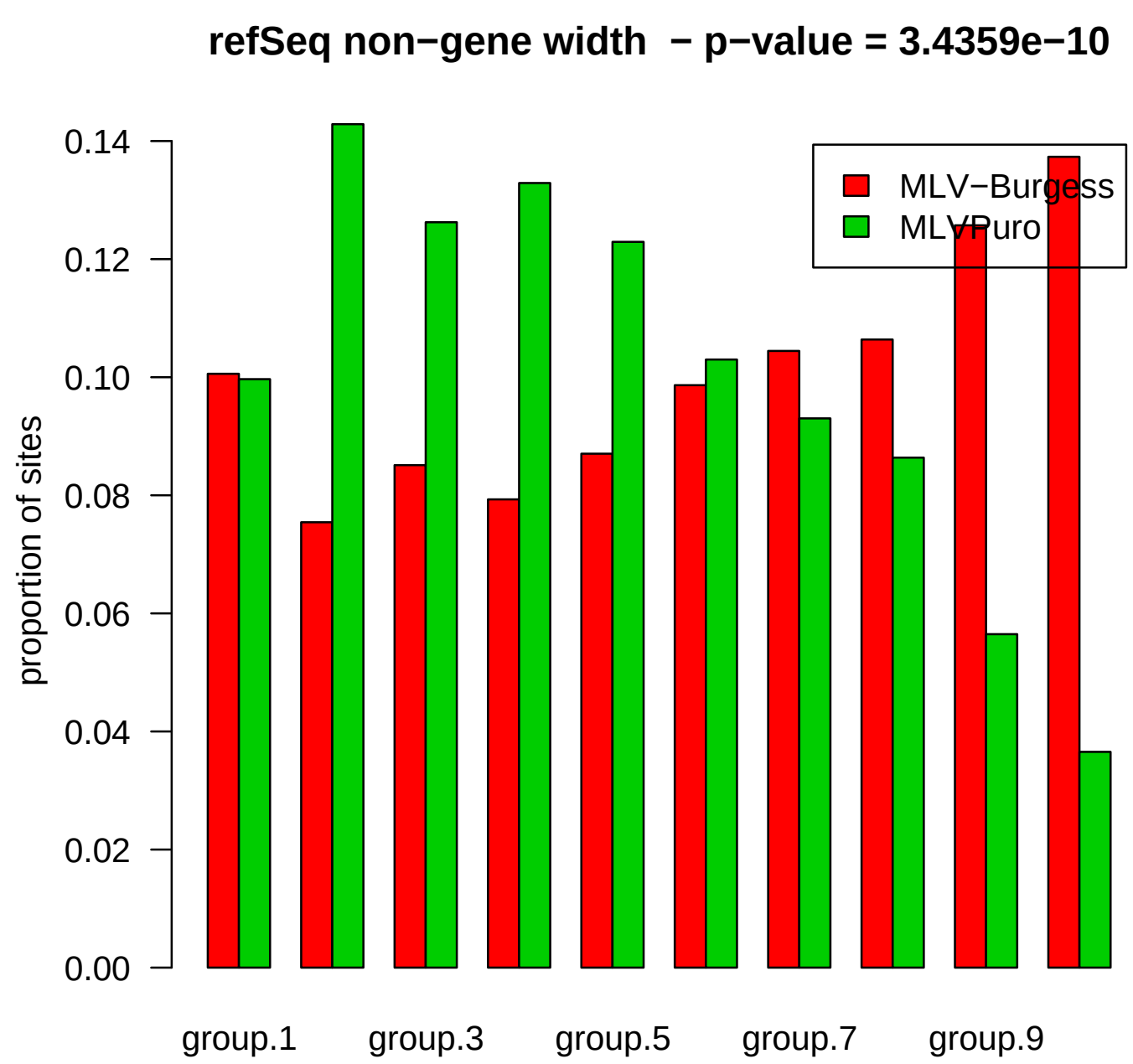

Category limits

	lower	category	upper
1	0.0001676227	group.1	0.02314041
2	0.0231404114	group.2	0.05673877
3	0.0567387687	group.3	0.10346024
4	0.1034602352	group.4	0.15794733
5	0.1579473307	group.5	0.20902820
6	0.2090282002	group.6	0.27251156
7	0.2725115616	group.7	0.33133189
8	0.3313318905	group.8	0.38102827
9	0.3810282654	group.9	0.44537610
10	0.4453760992	group.10	0.49986464

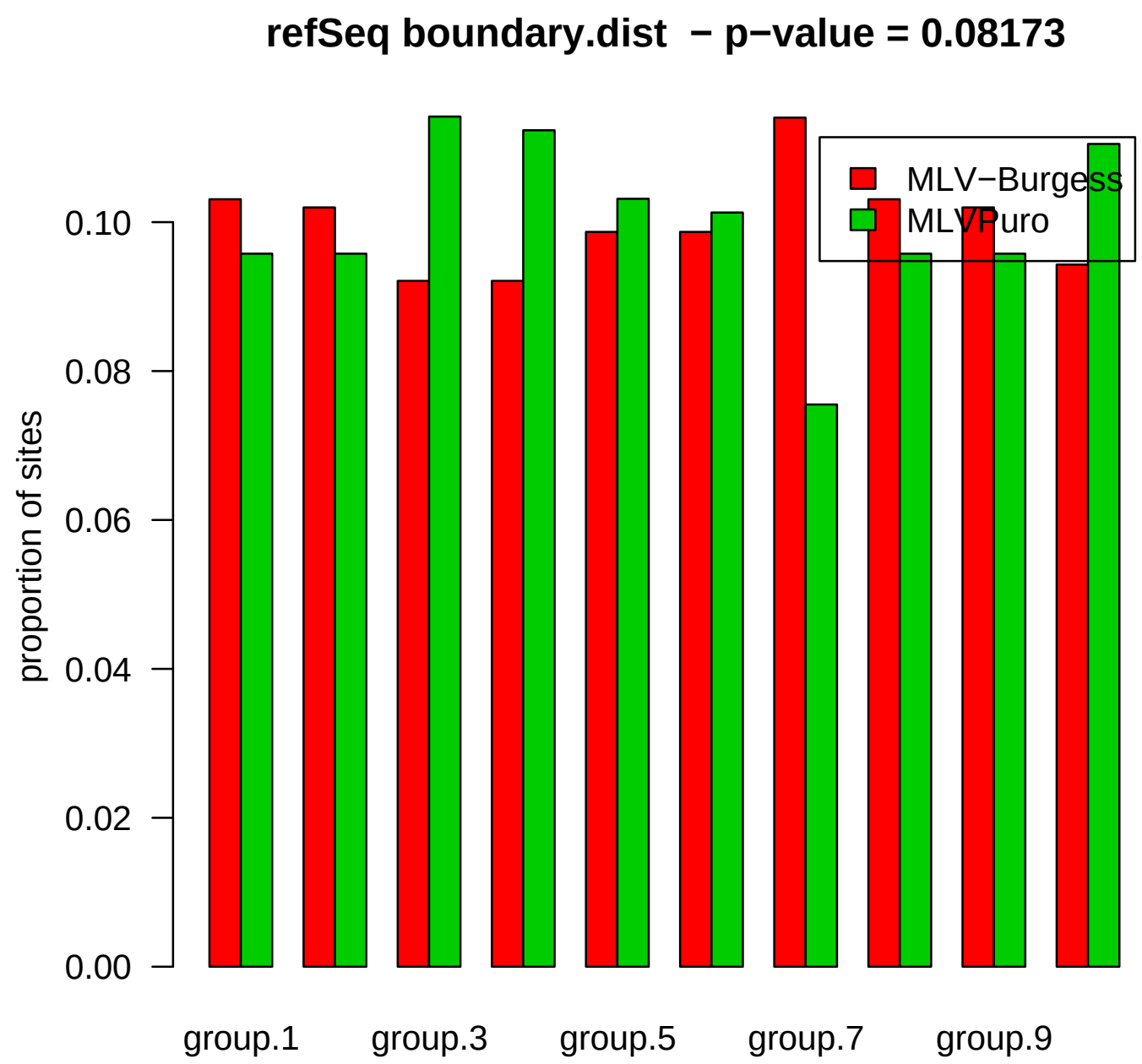

Category limits

	lower	category	upper
1	0.001022040	group.1	0.0424551
2	0.042455097	group.2	0.1183958
3	0.118395781	group.3	0.2124120
4	0.212412046	group.4	0.3155718
5	0.315571838	group.5	0.4104853
6	0.410485296	group.6	0.5073843
7	0.507384290	group.7	0.6460025
8	0.646002524	group.8	0.7796176
9	0.779617614	group.9	0.9022631
10	0.902263084	group.10	0.9998324

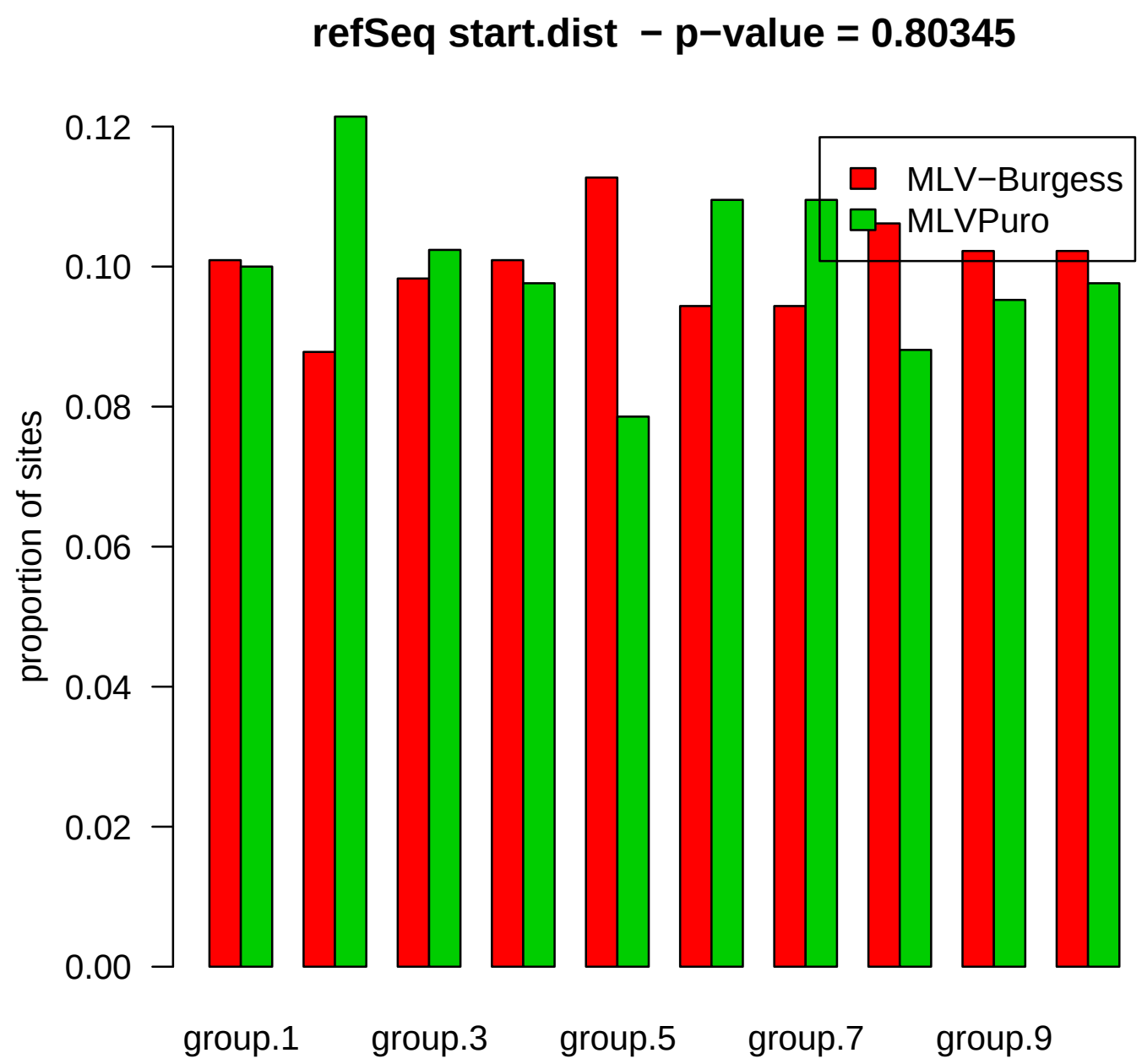
refSeq start.dist − p−value = 0.80345
proportion of sites
0.12
0.10
0.08
0.06
0.04
0.02
0.00
MLV−Burgess
MLVPuro
group.1
group.3
group.5
group.7
group.9

5.3 genScan Annotations

Category limits

	lower	category	upper
1	528.0	group.1	18950.5
2	18950.5	group.2	32159.0
3	32159.0	group.3	44187.0
4	44187.0	group.4	59619.0
5	59619.0	group.5	77935.0
6	77935.0	group.6	95755.0
7	95755.0	group.7	120931.5
8	120931.5	group.8	161743.0
9	161743.0	group.9	221476.0
10	221476.0	group.10	646561.0

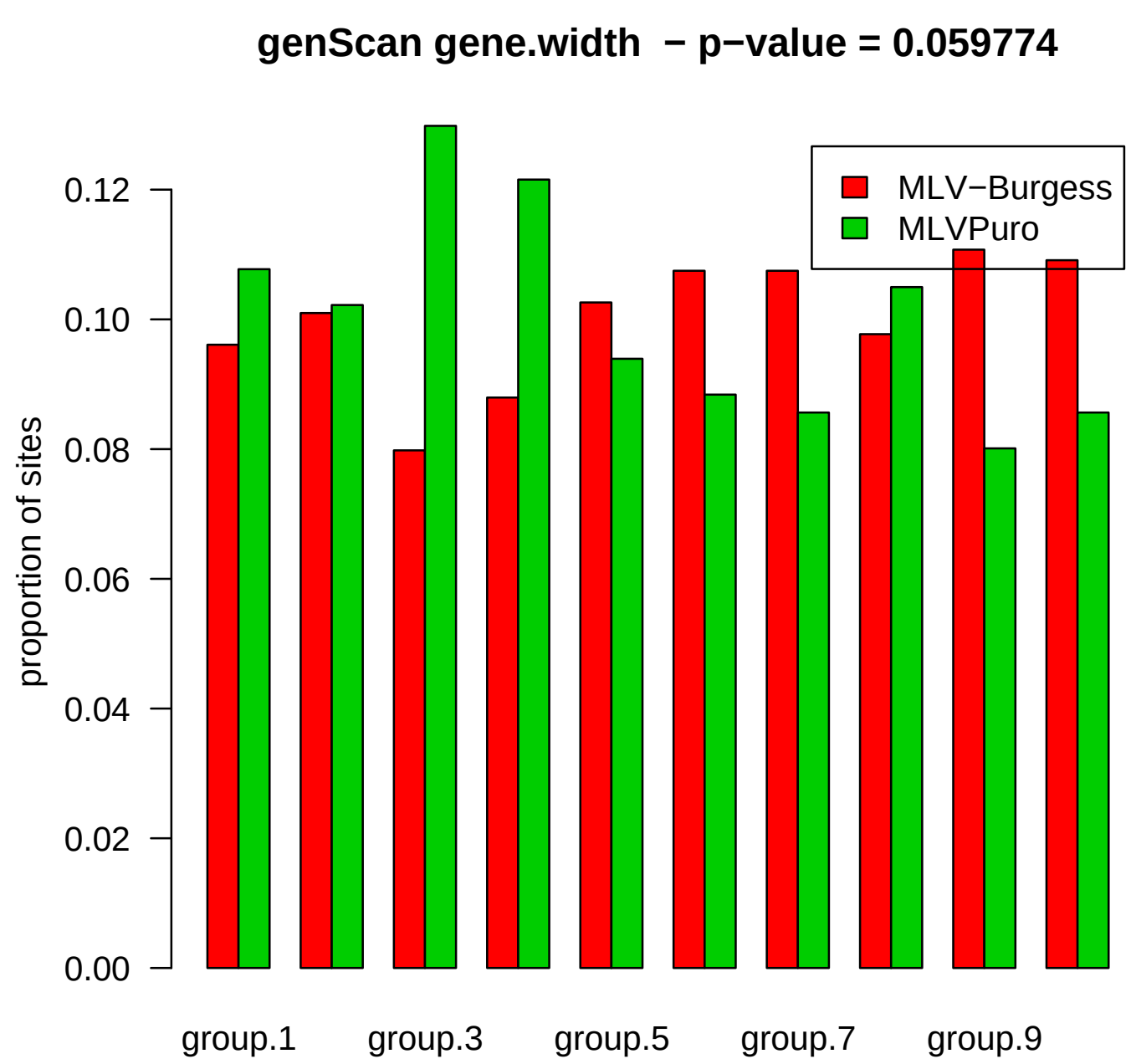

Category limits

	lower	category	upper
1	778.0	group.1	6125.6
2	6125.6	group.2	9893.2
3	9893.2	group.3	16013.2
4	16013.2	group.4	20307.8

5	20307.8	group.5	22644.0
6	22644.0	group.6	29514.0
7	29514.0	group.7	37045.6
8	37045.6	group.8	55001.6
9	55001.6	group.9	81036.0
10	81036.0	group.10	283599.0

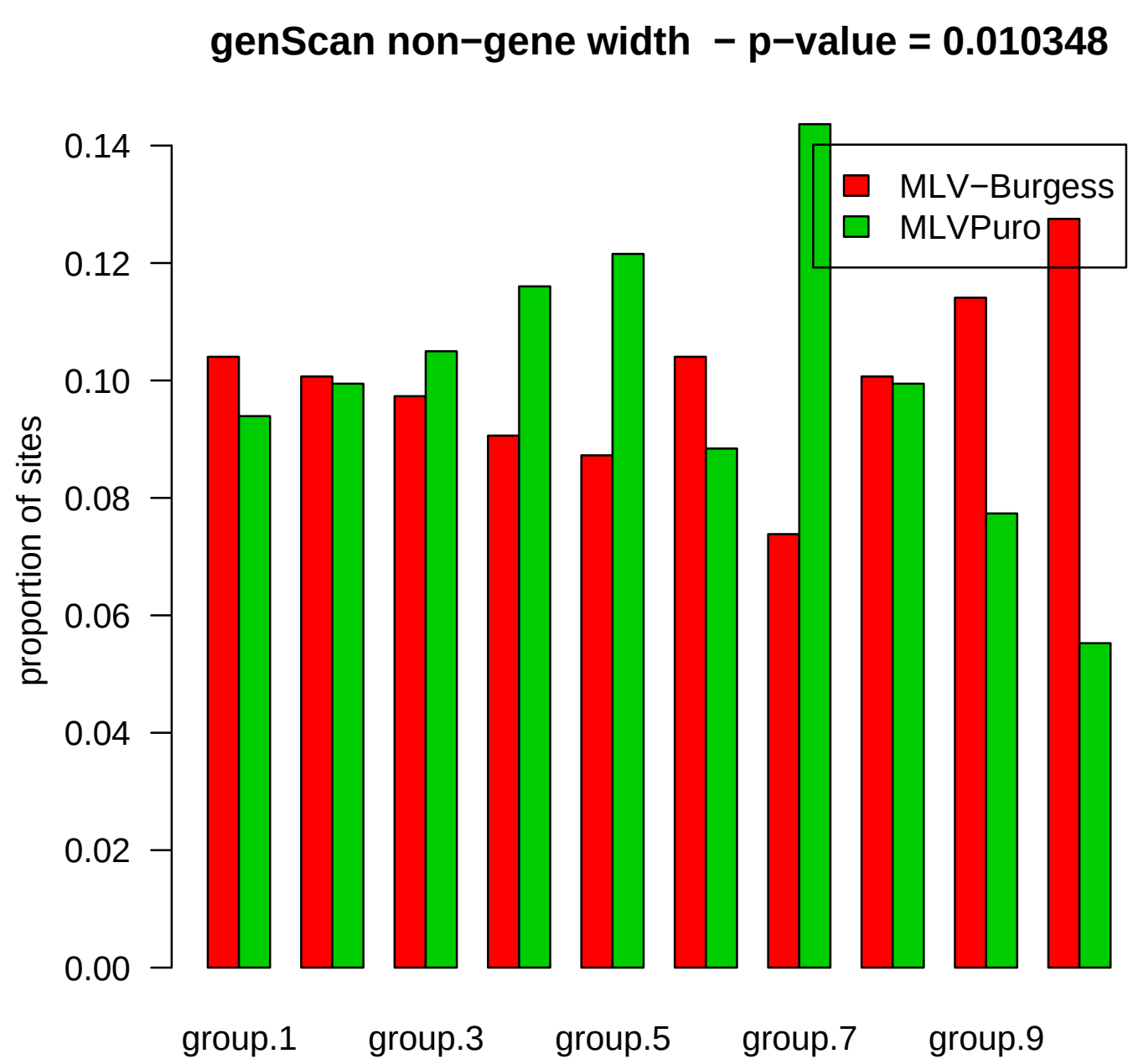

Category limits

	lower	category	upper
1	0.0002773669	group.1	0.03163856
2	0.0316385574	group.2	0.07389331
3	0.0738933124	group.3	0.12543881
4	0.1254388101	group.4	0.17225923
5	0.1722592265	group.5	0.22848296
6	0.2284829603	group.6	0.27822067
7	0.2782206676	group.7	0.33569052
8	0.3356905161	group.8	0.38824016
9	0.3882401608	group.9	0.44344987
10	0.4434498657	group.10	0.49997099

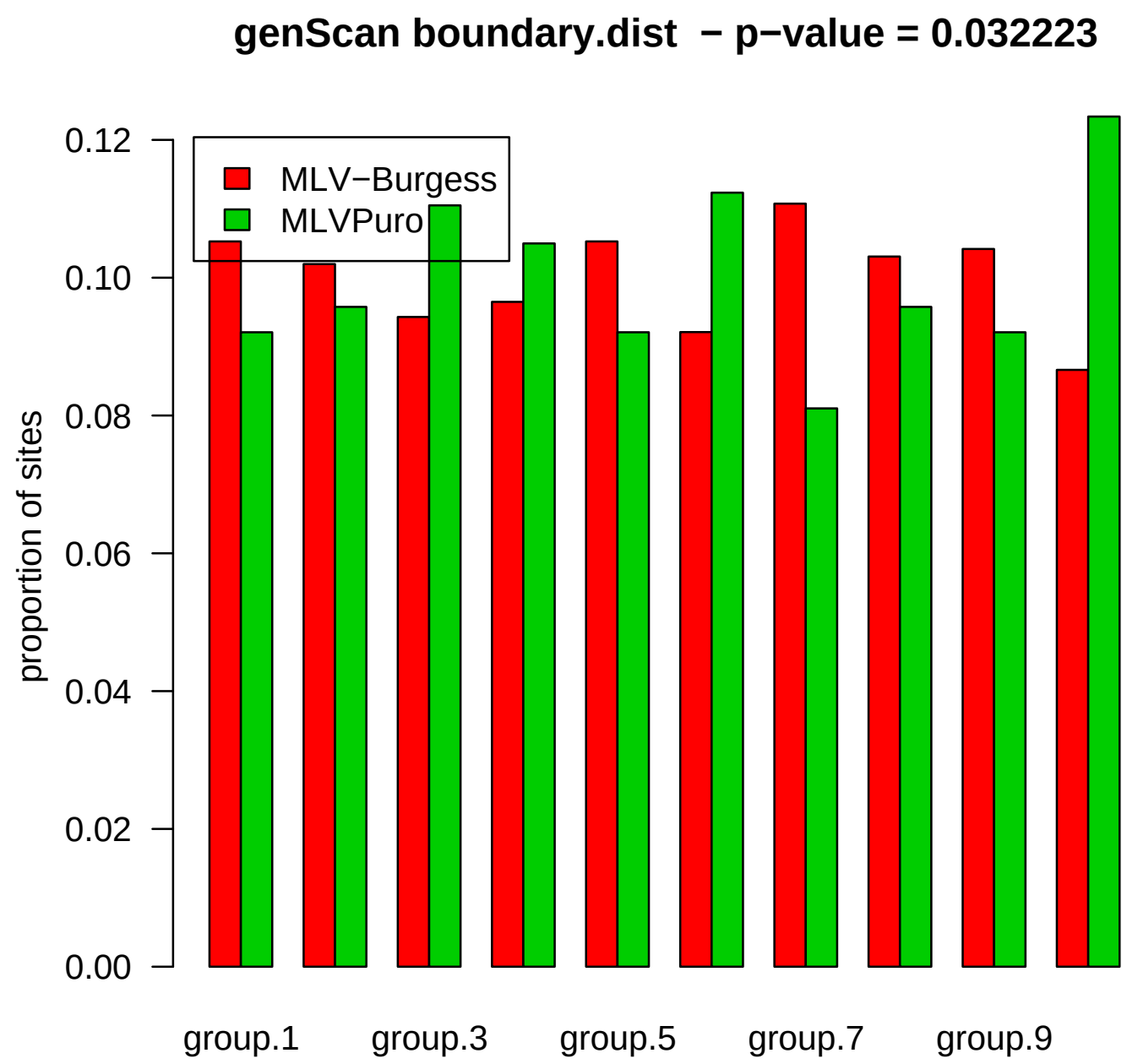

Category limits

	lower	category	upper
1	0.0002773669	group.1	0.04896258
2	0.0489625790	group.2	0.14423475
3	0.1442347542	group.3	0.24692466
4	0.2469246614	group.4	0.33433309
5	0.3343330876	group.5	0.42112499
6	0.4211249898	group.6	0.53551710
7	0.5355171033	group.7	0.64369593
8	0.6436959281	group.8	0.78417772
9	0.7841777172	group.9	0.89494284
10	0.8949428355	group.10	0.99897901

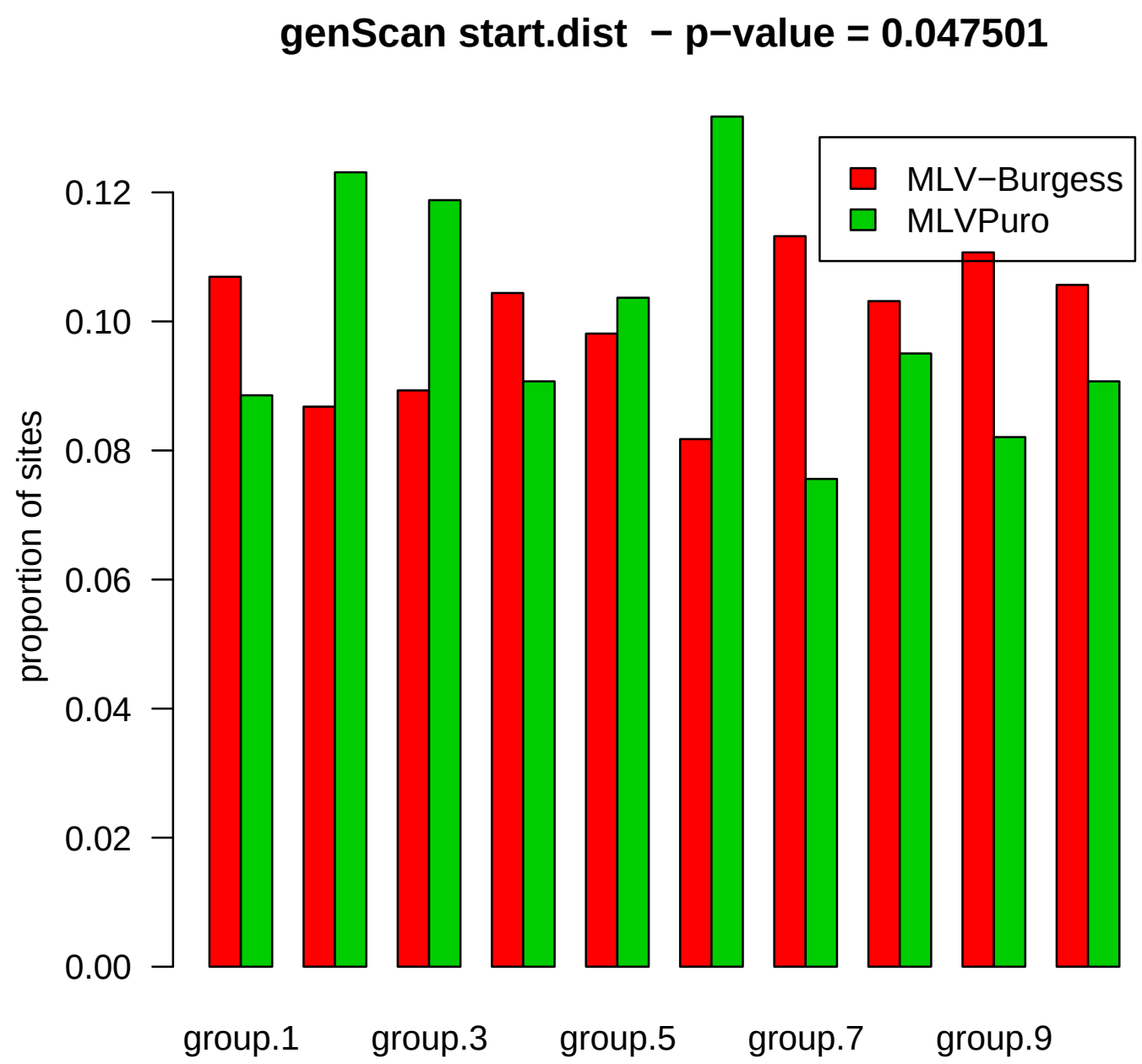
genScan start.dist − p−value = 0.047501
MLV−Burgess
MLVPuro
proportion of sites
0.00
0.02
0.04
0.06
0.08
0.10
0.12
group.1
group.3
group.5
group.7
group.9

5.4 uniGene Annotations

```
 Category limits

      lower category    upper
1      528.0  group.1  18950.5
2    18950.5  group.2  32159.0
3    32159.0  group.3  44187.0
4    44187.0  group.4  59619.0
5    59619.0  group.5  77935.0
6    77935.0  group.6  95755.0
7    95755.0  group.7 120931.5
8   120931.5  group.8 161743.0
9   161743.0  group.9 221476.0
10  221476.0 group.10 646561.0
```

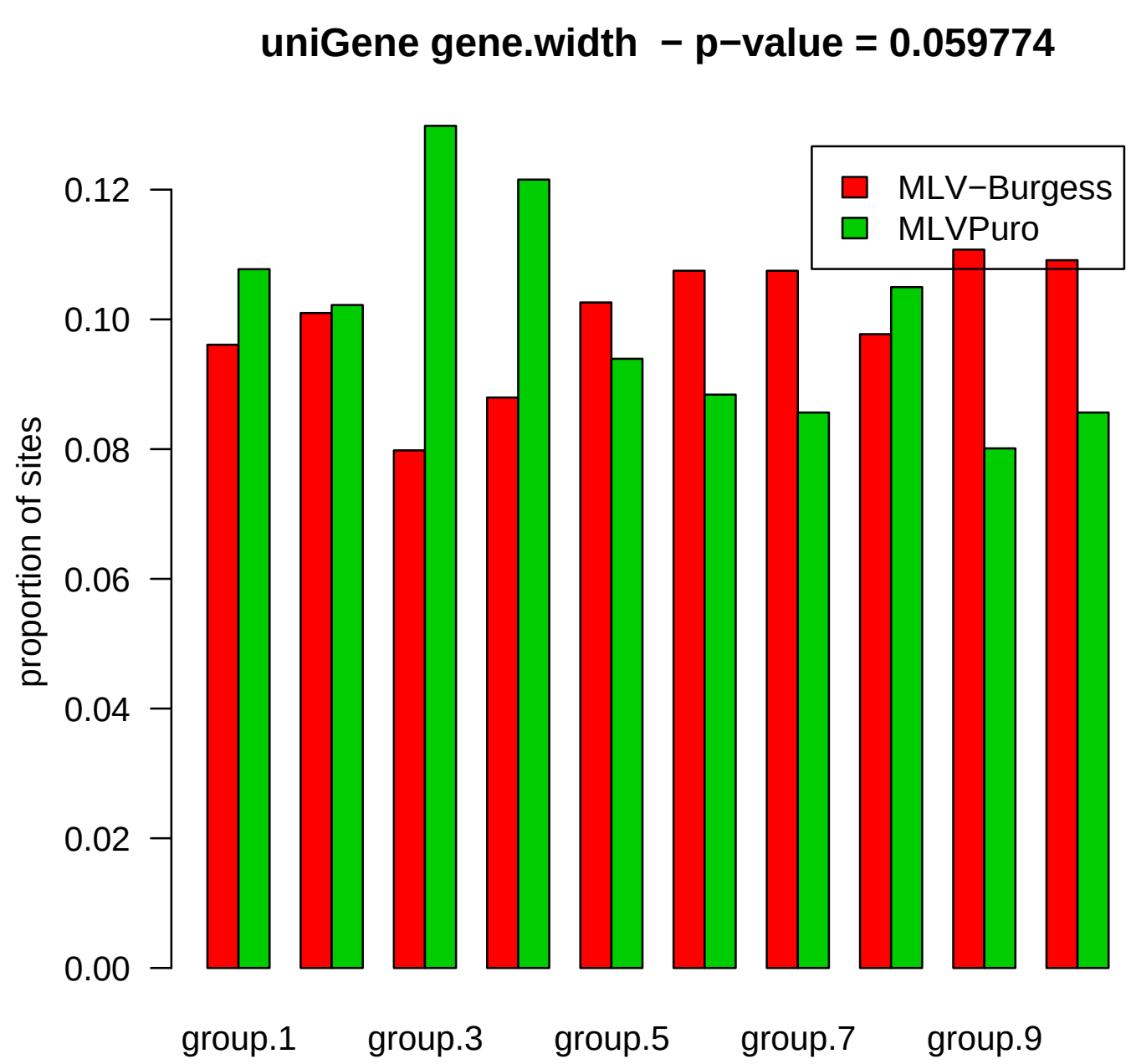


```
 Category limits

      lower category    upper
1     778.0  group.1   6125.6
2    6125.6  group.2   9893.2
3    9893.2  group.3  16013.2
4   16013.2  group.4  20307.8
```

5	20307.8	group.5	22644.0
6	22644.0	group.6	29514.0
7	29514.0	group.7	37045.6
8	37045.6	group.8	55001.6
9	55001.6	group.9	81036.0
10	81036.0	group.10	283599.0

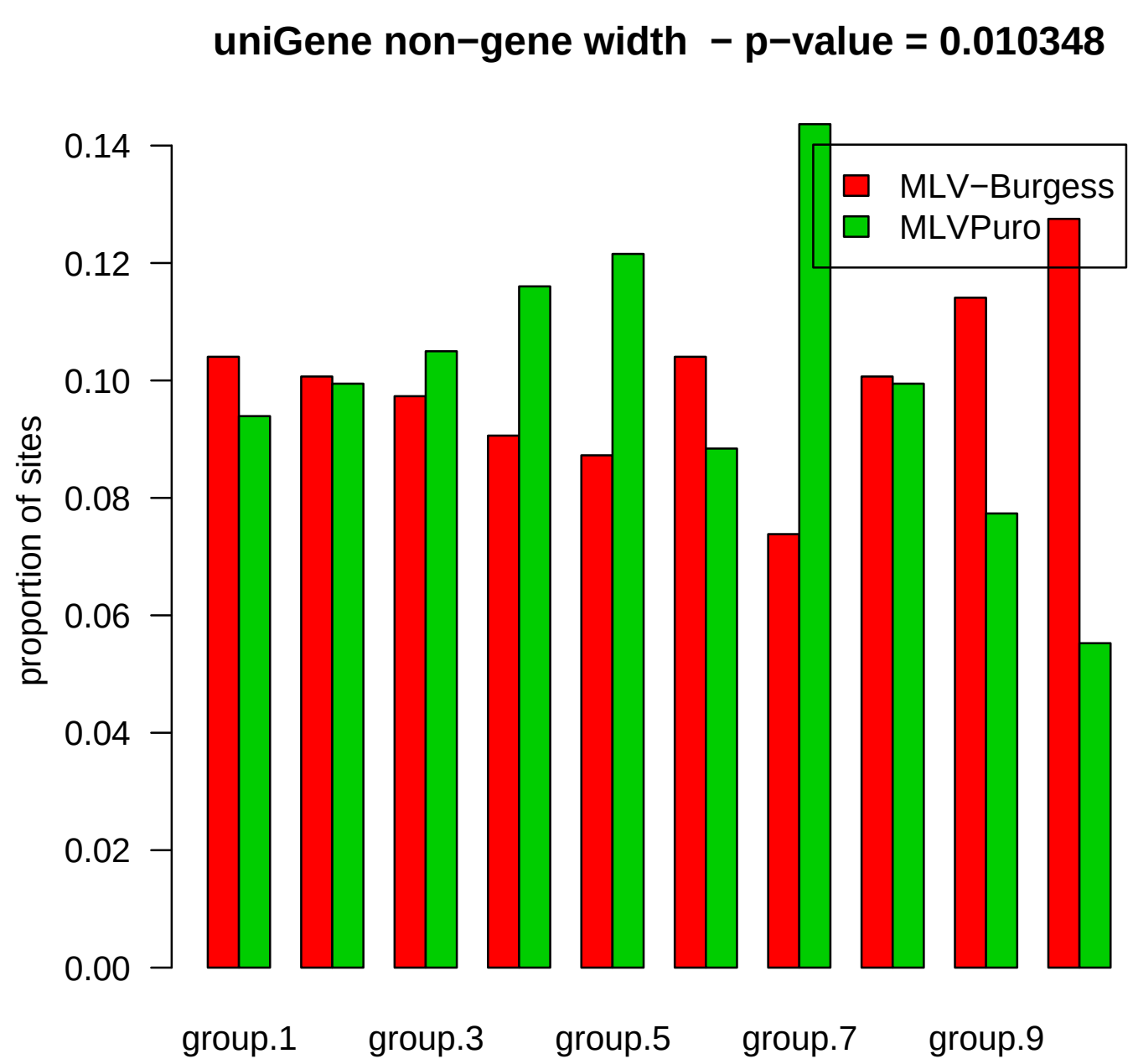

Category limits

	lower	category	upper
1	0.0002773669	group.1	0.03163856
2	0.0316385574	group.2	0.07389331
3	0.0738933124	group.3	0.12543881
4	0.1254388101	group.4	0.17225923
5	0.1722592265	group.5	0.22848296
6	0.2284829603	group.6	0.27822067
7	0.2782206676	group.7	0.33569052
8	0.3356905161	group.8	0.38824016
9	0.3882401608	group.9	0.44344987
10	0.4434498657	group.10	0.49997099

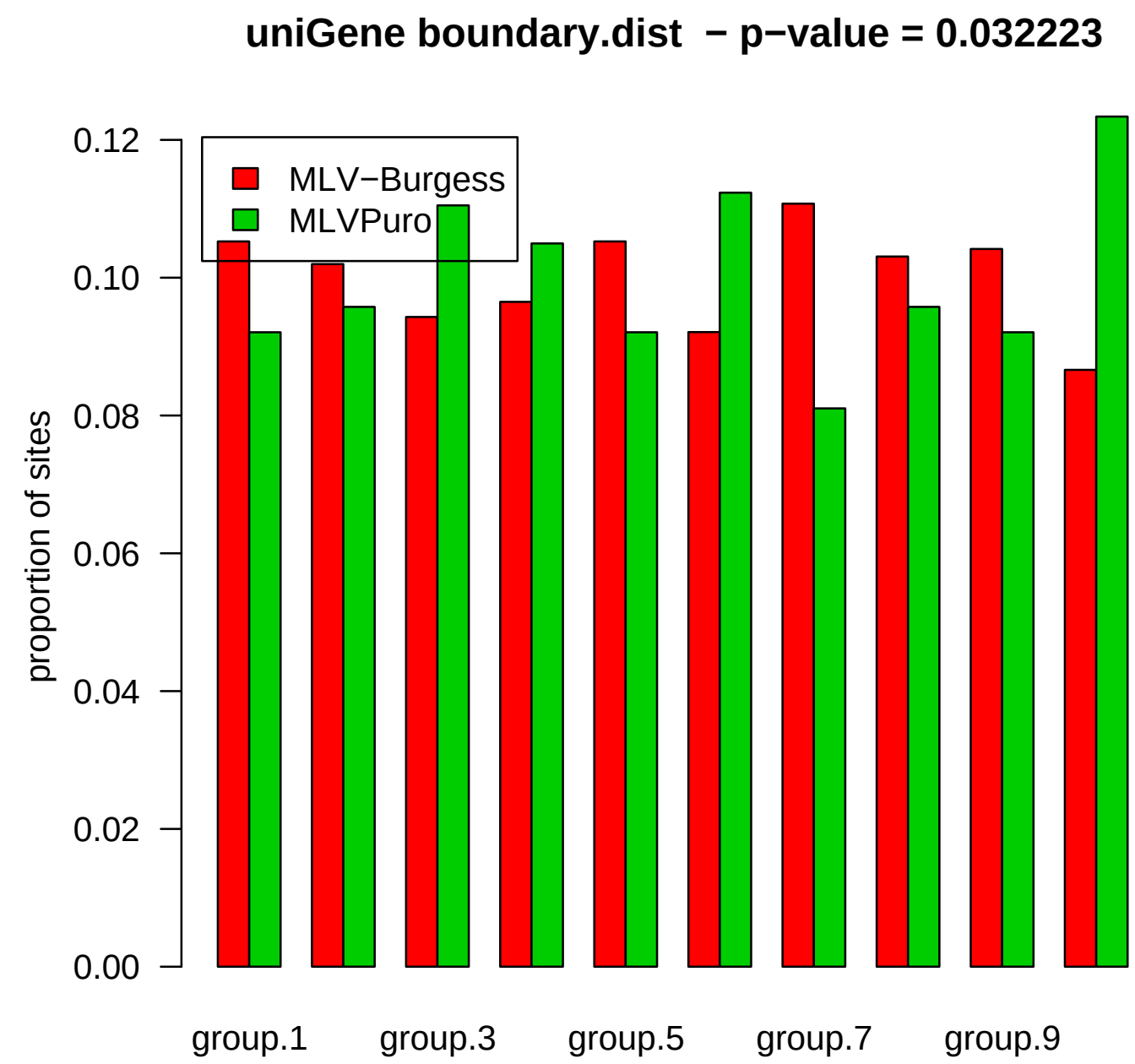

Category limits

	lower	category	upper
1	0.0002773669	group.1	0.04896258
2	0.0489625790	group.2	0.14423475
3	0.1442347542	group.3	0.24692466
4	0.2469246614	group.4	0.33433309
5	0.3343330876	group.5	0.42112499
6	0.4211249898	group.6	0.53551710
7	0.5355171033	group.7	0.64369593
8	0.6436959281	group.8	0.78417772
9	0.7841777172	group.9	0.89494284
10	0.8949428355	group.10	0.99897901

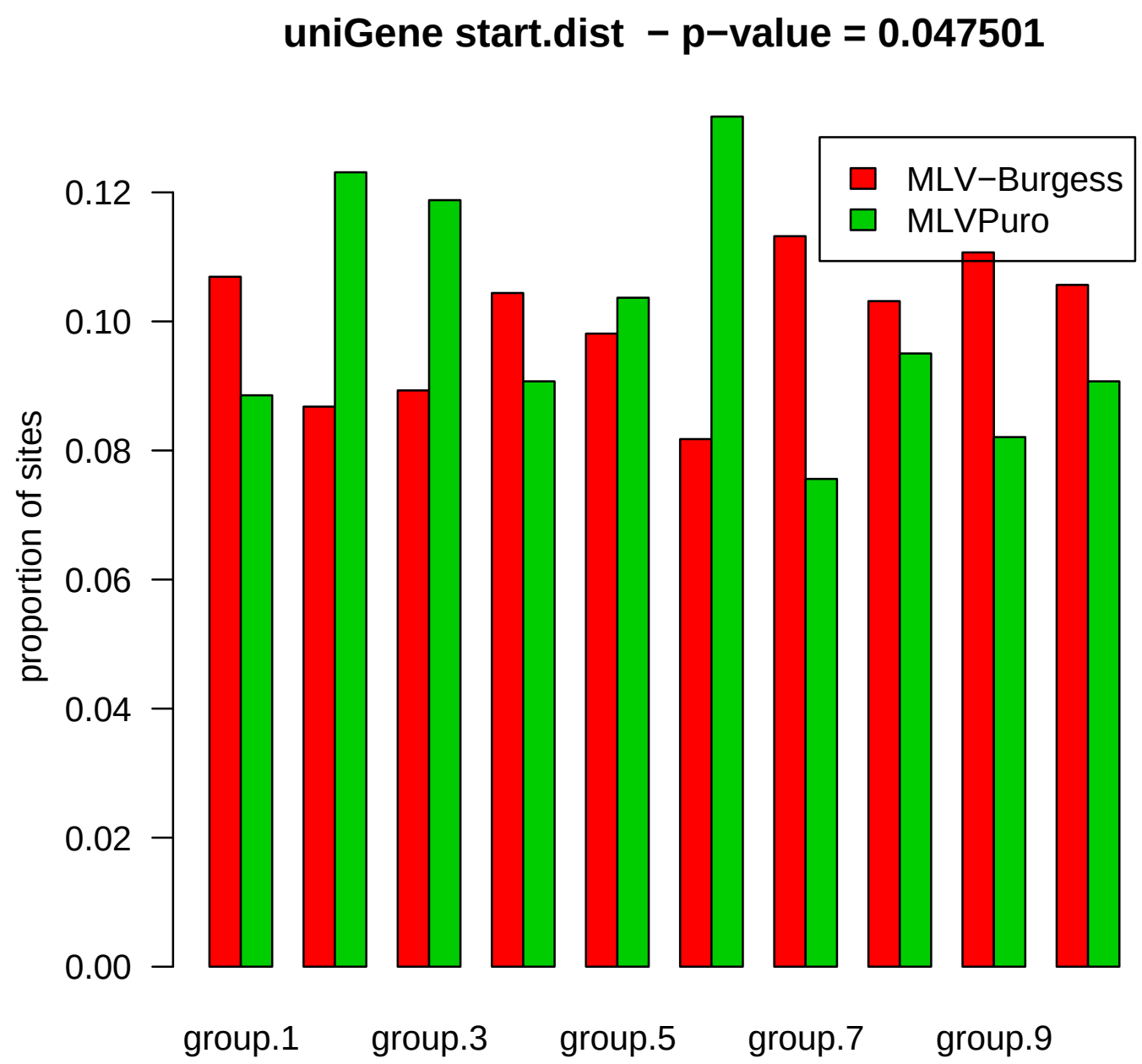
uniGene start.dist − p−value = 0.047501
MLV−Burgess
MLVPuro
proportion of sites
0.00
0.02
0.04
0.06
0.08
0.10
0.12
group.1
group.3
group.5
group.7
group.9

6 GC content

Here we study the effect of GC content on insertion. The GC content is taken from the Human Genome Draft at GoldenPath from the table
`http://genome.ucsc.edu/goldenPath/hg17/database/gc5Base.txt.gz`.

Following the plot is a table of fitted coefficients based on splitting the GC percent data at the median.

Category limits

	lower	category	upper
1	28.88672	group.1	36.07422
2	36.07422	group.2	38.51562
3	38.51562	group.3	40.50781
4	40.50781	group.4	42.01172
5	42.01172	group.5	43.82812
6	43.82812	group.6	45.72266
7	45.72266	group.7	47.98828
8	47.98828	group.8	50.33203
9	50.33203	group.9	54.10156
10	54.10156	group.10	67.44141

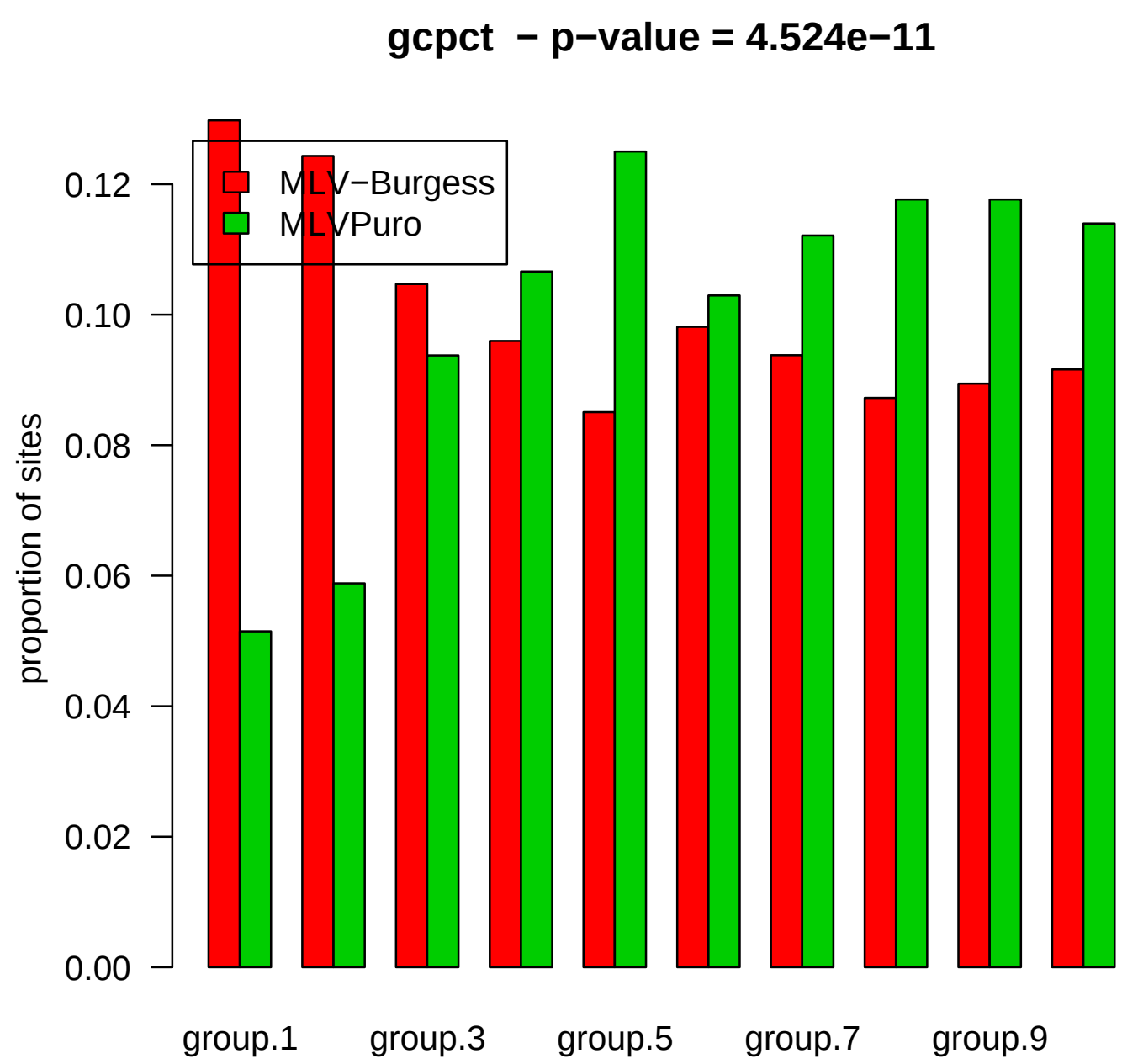

7 Cytobands

Here we study the association of cytoband with insertion intensity. The data are obtained from
`http://genome.ucsc.edu/goldenPath/hg17/database/cytoBand.txt.gz`.

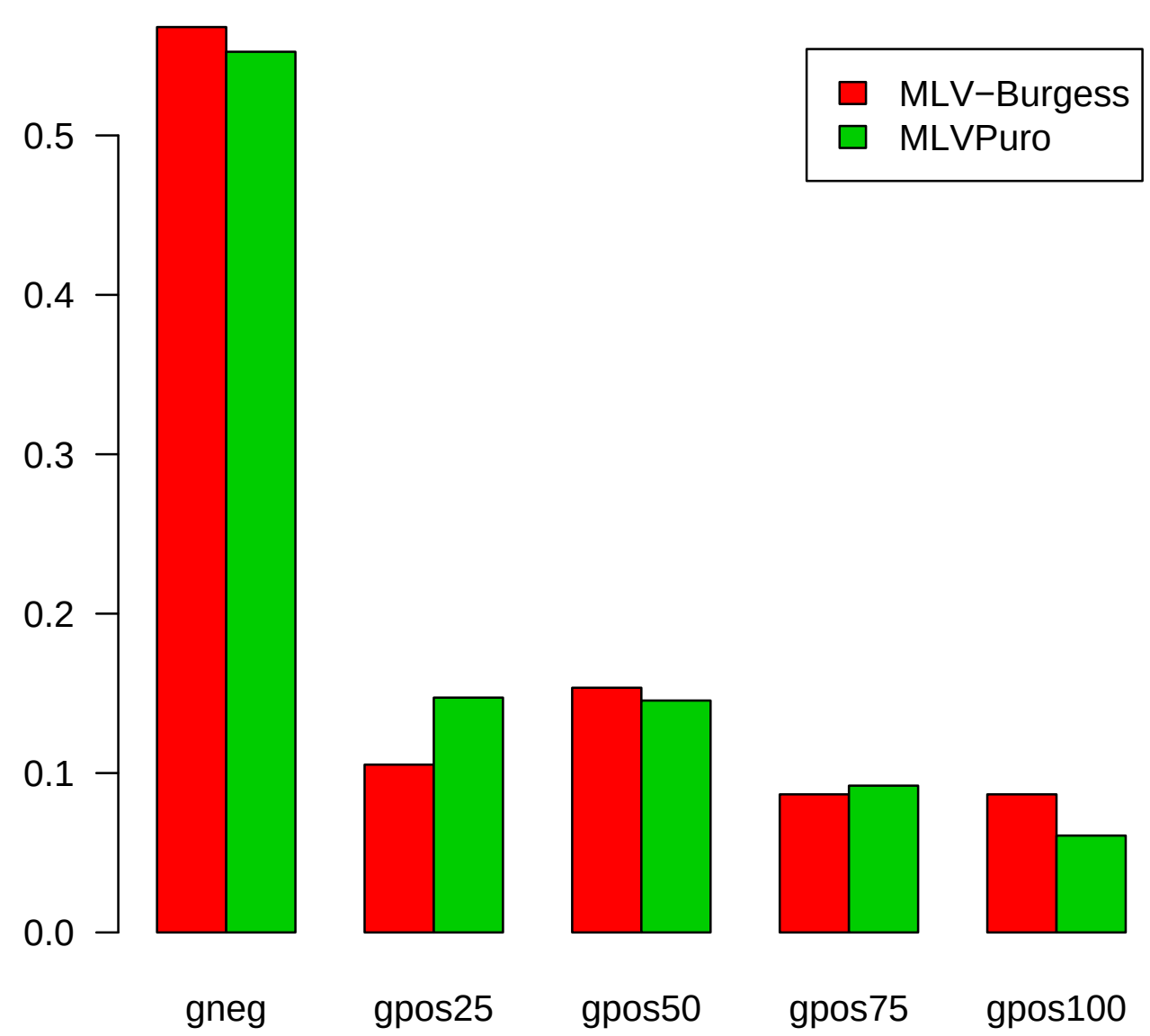

A formal test of significance attains a p-value of 0.080486.

References

[1] P. McCullagh and John A. Nelder. *Generalized linear models.* (Chapman & Hall ltd, 1999).

[2] Xiaolin Wu, Yuan Li, Bruce Crise, Shawn M. Burgess "Transcription Start Regions in the Human Genome Are Favored Targets for MLV Integration," *Science,* **300**(5626), (June 2003): 1749-1751.
